# Supplementary material for: Conformational flexibility of HIV-1 envelope glycoproteins modulates transmitted/founder sensitivity to broadly neutralizing antibodies
Source: Nat Commun. 2024 Aug 26;15:7334. doi: 10.1038/s41467-024-51656-4 (PMC11347675; doi:10.1038/s41467-024-51656-4)
Supplement: Supplementary file 1 — Supplementary Information [file 41467_2024_51656_MOESM1_ESM.pdf]

Supplementary Information

Conformational flexibility of HIV-1 envelope glycoproteins modulates transmitted / founder sensitivity to broadly neutralizing antibodies

Durgadevi Parthasarathy[1,11], Karunakar Reddy Pothula[2,11], Sneha Ratnapriya[1], Héctor Cervera Benet[1], Ruth Parsons[2,3], Xiao Huang[2], Salam Sammour[2], Katarzyna Janowska[2], Miranda Harris[1], Joseph Sodroski[4,5], Priyamvada Acharya[2,3,6] and Alon Herschhorn[1,7,8,9,10]*

[1]Division of Infectious Diseases and International Medicine, Department of Medicine, University of Minnesota, Minneapolis, MN, USA

[2]Duke Human Vaccine Institute, Duke University, Durham, NC, USA

[3]Department of Biochemistry, Duke University, Durham, NC, USA

[4]Department of Cancer Immunology and Virology, Dana-Farber Cancer Institute, Boston, MA, USA

[5]Department of Microbiology, Harvard Medical School, Boston, MA, USA

[6]Department of Surgery, Duke University, Durham, NC, USA

[7]Institute for Molecular Virology, University of Minnesota, Minneapolis, MN, USA

[8]Microbiology, Immunology, and Cancer Biology Graduate Program, University of Minnesota, Minneapolis, MN, USA

[9]The College of Veterinary Medicine Graduate Program, University of Minnesota, Minneapolis, MN, USA

[10]Molecular Pharmacology and Therapeutics Graduate Program, University of Minnesota, Minneapolis, MN, USA

[11]These authors contributed equally: Durgadevi Parthasarathy and Karunakar Reddy Pothula

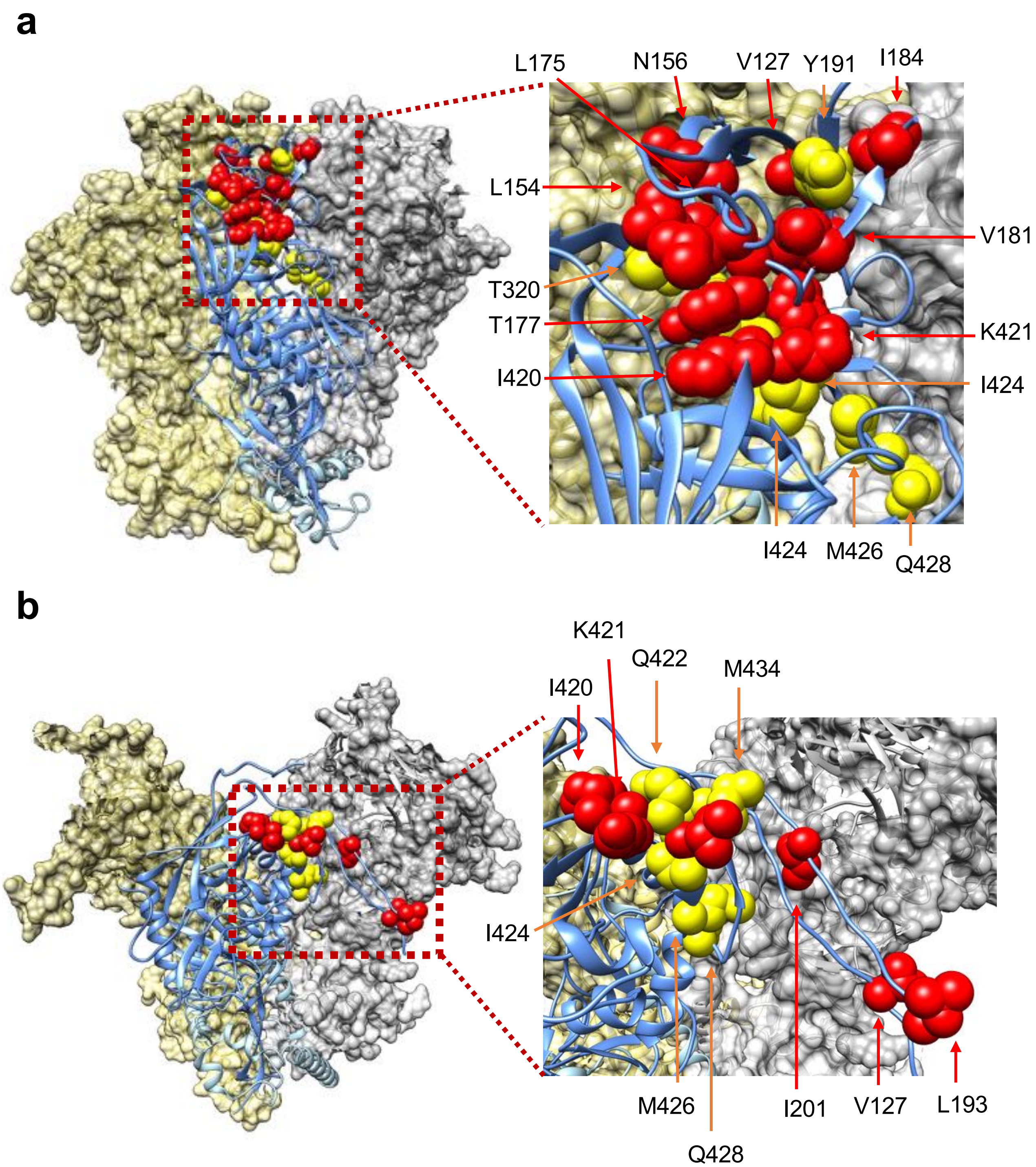

Supplementary Figure 1. Env residue mapping. We mapped Env residues that contribute to maintenance of Env closed conformation on available SOSIP Env structures. Residues that were resolved and in which changes resulted in hypersensitivity to multiple internal-epitope Env ligands (red) or only one internal-epitope Env ligand (yellow) are labeled (neutralization data and ligands tested are shown in Fig. 1 and Supplementary table 1). a, We used the crystal structure of BG505 SOSIP bound to BMS806, which blocks conformational changes of HIV-1 Env on virions (pdb entry 6mtj). b, We used the model of B41 SOSIP bound to sCD4 and 17b (pdb entry 5vn3). Left panels - SOSIP trimers with 2 of the protomers shown as surfaces and one front protomer shown as ribbon in which specific residues were mapped. Right - enhanced view of specific residues. Figure was prepared using the Chimera program (https://www.cgl.ucsf.edu/chimera/).

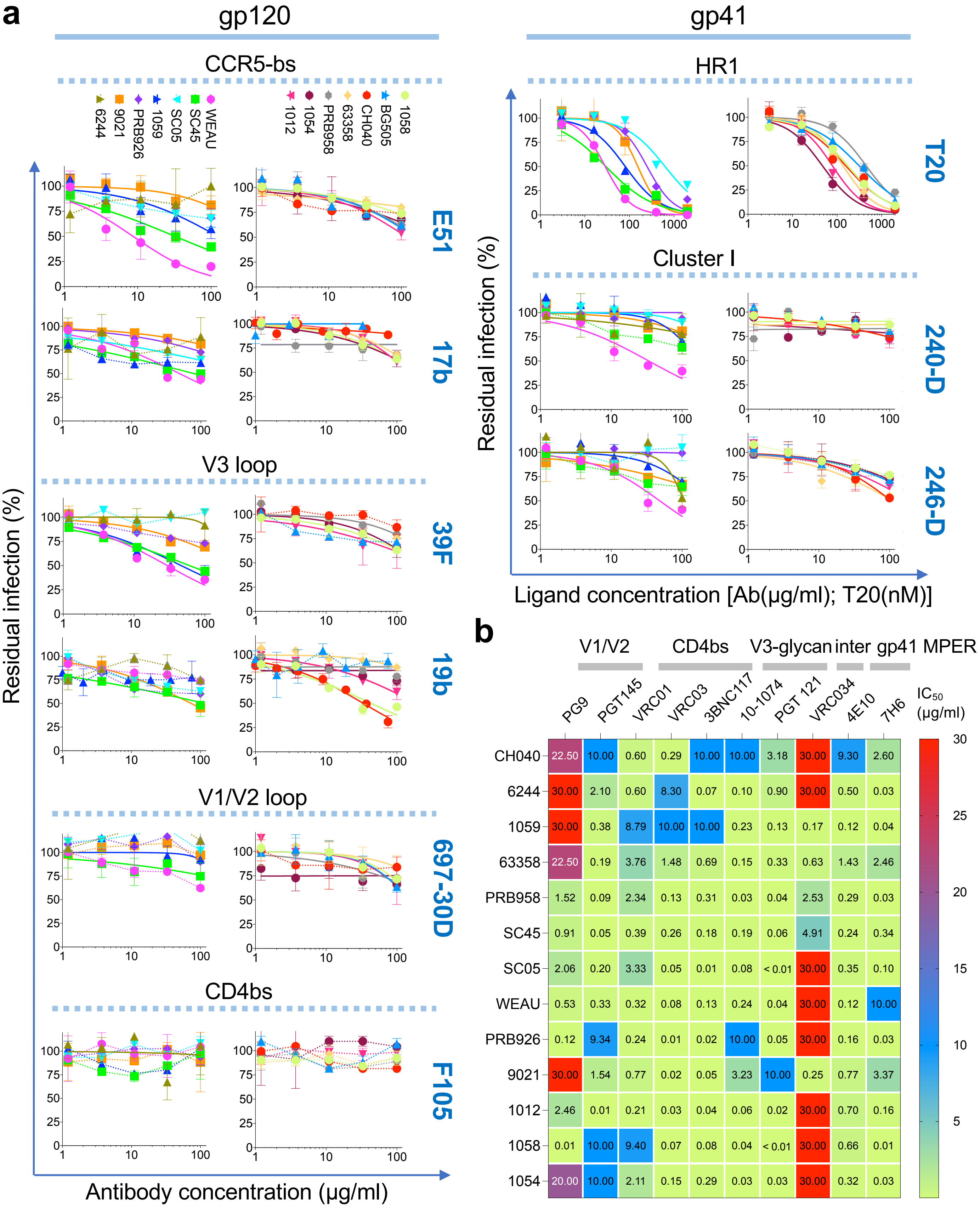

Supplementary Figure 2. Sensitivity of HIV-1 pseudotyped with transmitted/founder (T/F) Envs to different ligands. **a,** Sensitivity to ligands preferentially recognizing internal epitopes. BG505, a control. Dashed lines connect data that could not be fitted to a standard inhibition curve. Data are mean values ± s.d.; n = 2 or 4 technically independent experiments except for 6244 data, which represent one of 2 independent experiments, each performed in duplicate. Additional repeats of independent experiments for selected Envs and Abs are shown in Fig. 1. **b,** We calculated the sensitivity (IC_{50} values) of 13 T/F Envs to bnAbs of all known classes from dose response curves (representative curves are shown in Supplementary Fig. 3). 10.0 or 30.0 values represent values >10 or >30 μg/ml, respectively; inter, gp120-gp41 interface.

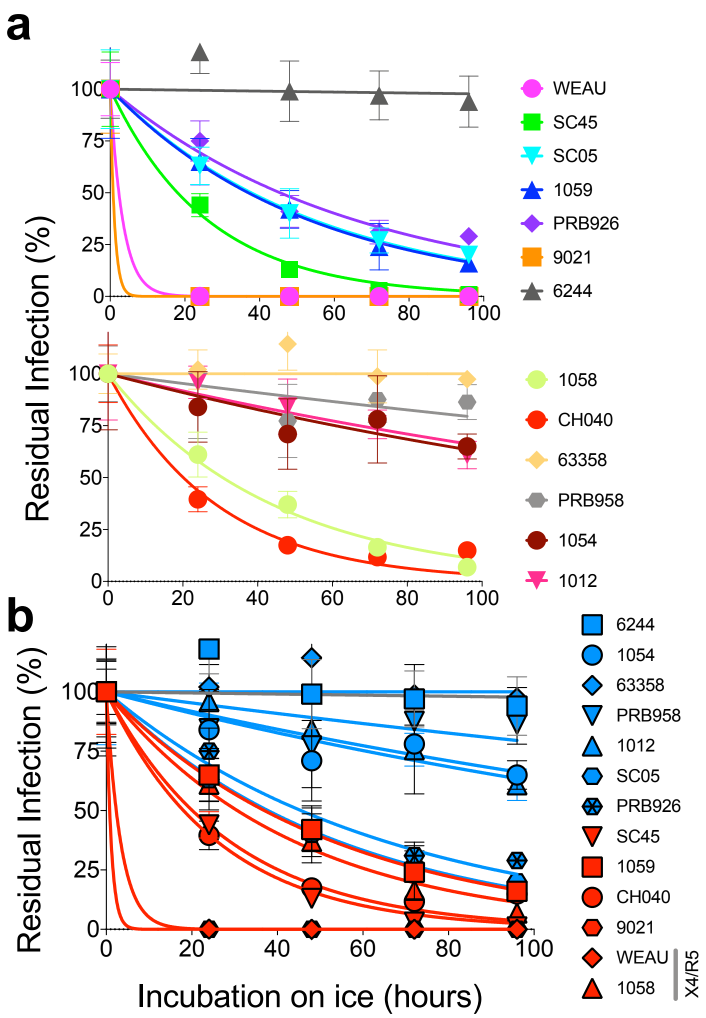

c

T/F Envs	sCD4 IC_{50} (nM)	s.e.
PRB926	55.95	6.116
6244	425.7	133.3
1059	95.7	44.54
1054	58.19	24.25
9021	5.453	3.656
1058	114.2	35.29
CH040	51.96	3.42
63358	463	107.2
PRB958	719.1	222.9
SC45	49.48	15.81
SC05	82.04	22.97
WEAU	31.14	12.54
1012	260.6	64.24

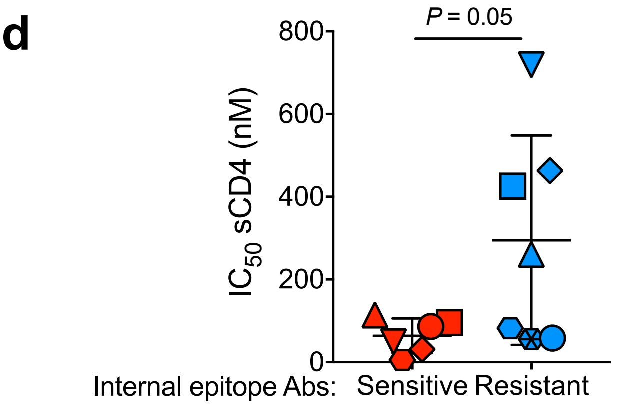

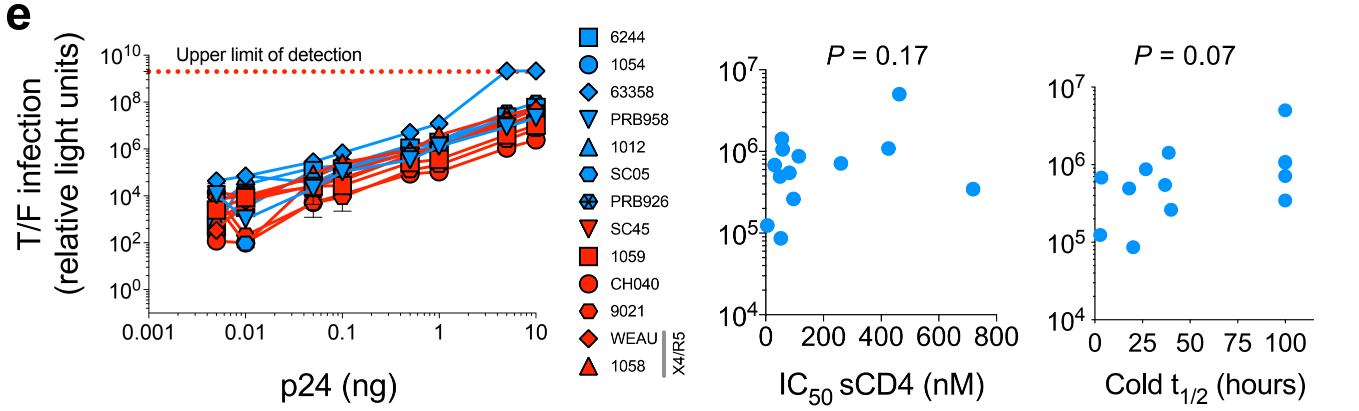

Supplementary Figure 3. HIV-1 T/F Env sensitivity to cold exposure and soluble CD4 (sCD4). a, We assessed sensitivity of 13 T/F Envs to cold exposure by measuring viral (pseudoviruses) infectivity after pre-incubation of the pseudoviruses on ice for the indicated times. **b,** Similar to (**a**) but T/F Envs were plotted on a single plot and color coded according to sensitivity to internal-epitope antibodies. Blue, resistant Envs (up to 100 μg/ml); red, Envs that were moderately inhibited by at least one internal-epitope antibody. Two dual-tropic (X4/R5) T/F Envs in this group are indicated in panel (**b**) legend. **c,** Sensitivity of HIV-1 pseudotyped with 13 T/F Envs to sCD4. Half maximal inhibitory concentrations (IC_{50}s) were calculated from dose response curves of pseudoviruses to increasing concentration of sCD4. **d,** Statistical analysis of difference between sCD4 sensitivity of T/F Envs that are sensitive and those that are resistant to internal-epitope antibodies. **e, Controls.** Left - we measured comparable infection levels of Cf2Th-CD4/CCR5 target cells by HIV-1 pseudotyped with 13 T/F Envs regardless of their internal-epitope exposure. Right - we did not detect statistically significant correlation between T/F viral infectivity and sCD4 or cold sensitivity. Color codes in panels **d** and **e (left)** are identical to the code in panel **b**. *P,* calculated two-tailed Student's t-test *P* value (panel d) and two-tailed Spearman correlation *P* value (panel e). Data are mean ± s.d. of representative (a, b, and e) or average (d) of n = at least 2 independent experiments, each performed with 2 or 4 replicates.

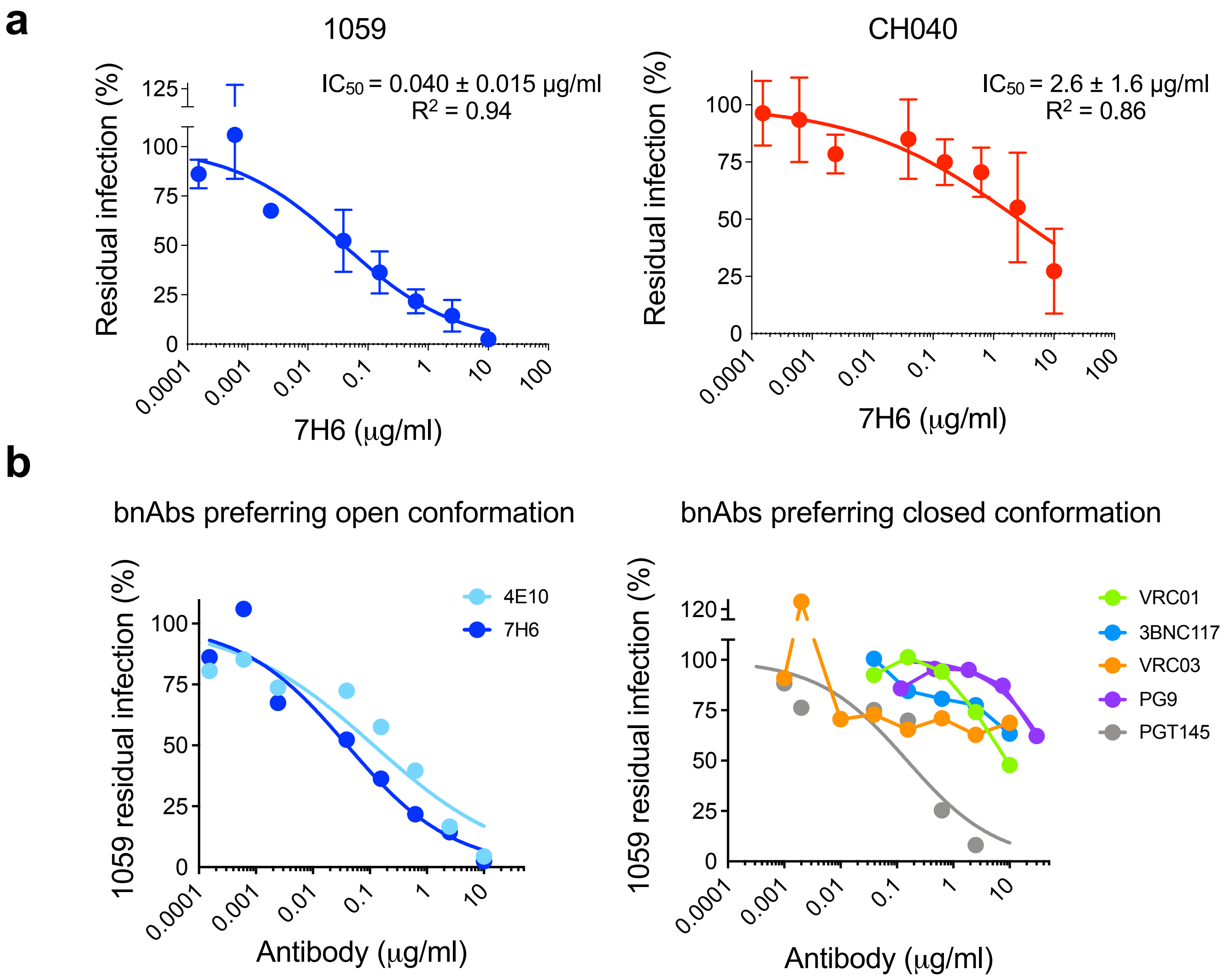

Supplementary Figure 4. HIV-1 T/F Env sensitivity to bnAbs. a, Sensitivity of viral entry, mediated by two T/F Envs, to the gp41-directed 7H6 bnAb. **b,** Sensitivity of viral entry mediated by 1059 Envs to bnAbs that prefer specific Env conformations. Data of 1059 sensitivity to 7H6 from panel **a** (left) is shown also in panel **b** (left) for reference. Data are mean values + s.e.m. (**a**) or mean values (**b**) of n = 2 independent experiments, each performed at least in duplicate. Calculated IC_{50} values of T/F Env sensitivity to all bnAbs tested are reported in **Supplementary Fig. 10** (exact values indicated on a heat map).

a

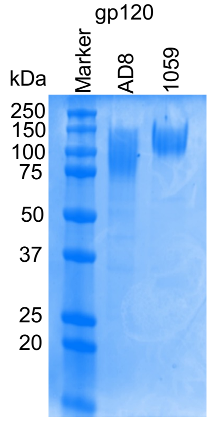

c

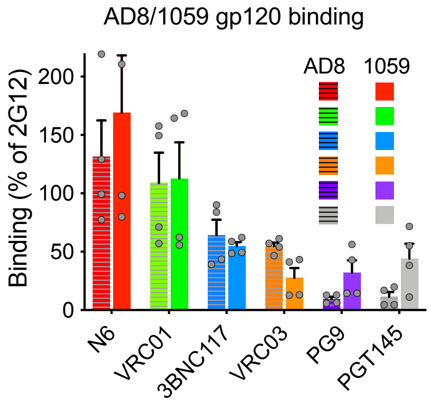

b

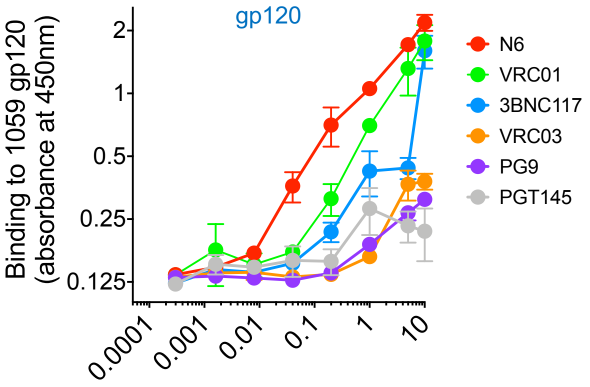

d

bnAb	1059 Envs		AD8 Envs	
	IC50 (μg/ml)	s.e.m.	IC50 (μg/ml)	s.e.m.
N6	1.2580	0.9458	0.1821	0.0587
VRC01	8.7980	1.6620	0.7173	0.1014
3BNC117	>10	n/a	0.0387	0.0070
VRC03	>10	n/a	0.0765	0.0130
PG9	>30	n/a	1.6280	0.3548
PGT145	0.1514	0.0743	0.1060	0.0193

e

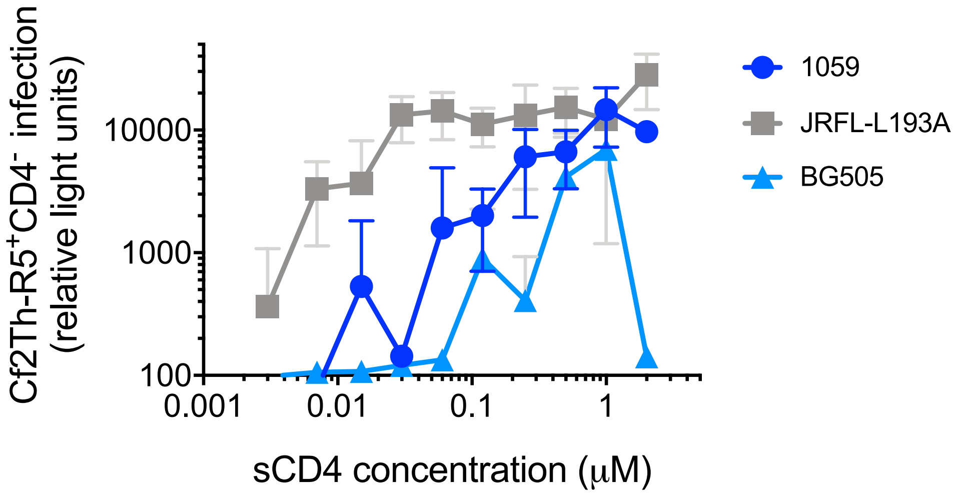

Supplementary Figure 5. Antibody binding to soluble gp120 and CD4 dependence of HIV-1 Env-mediated entry. a, SDS-PAGE of purified 1059 gp120. Gel is a representative of n = 2 independent experiments. **b**, Binding of CD4bs and V1V2 bnAbs to soluble 1059 gp120. **c**, Comparison of CD4bs and V1V2 bnAbs binding (1 μg/ml) to soluble gp120 of AD8 and 1059. **d,** Comparison of AD8 and 1059 pseudovirus neutralization by bnAbs from (**c**). Data in panels **b-d** are mean values + s.e.m. of n = 2 independent experiments, each performed at least in duplicate. **e**, We incubated recombinant pseudoviruses carrying the different Envs with CD4-negative, CCR5-expressing Cf2Th cells in the presence of indicated concentrations of sCD4. Between 48-72 hours post infection we measured the activity of firefly luciferase reporter protein (relative light units) to assess viral entry. Data are mean values ± s.d. of a representative from n = 2 independent experiments, each performed in triplicate.

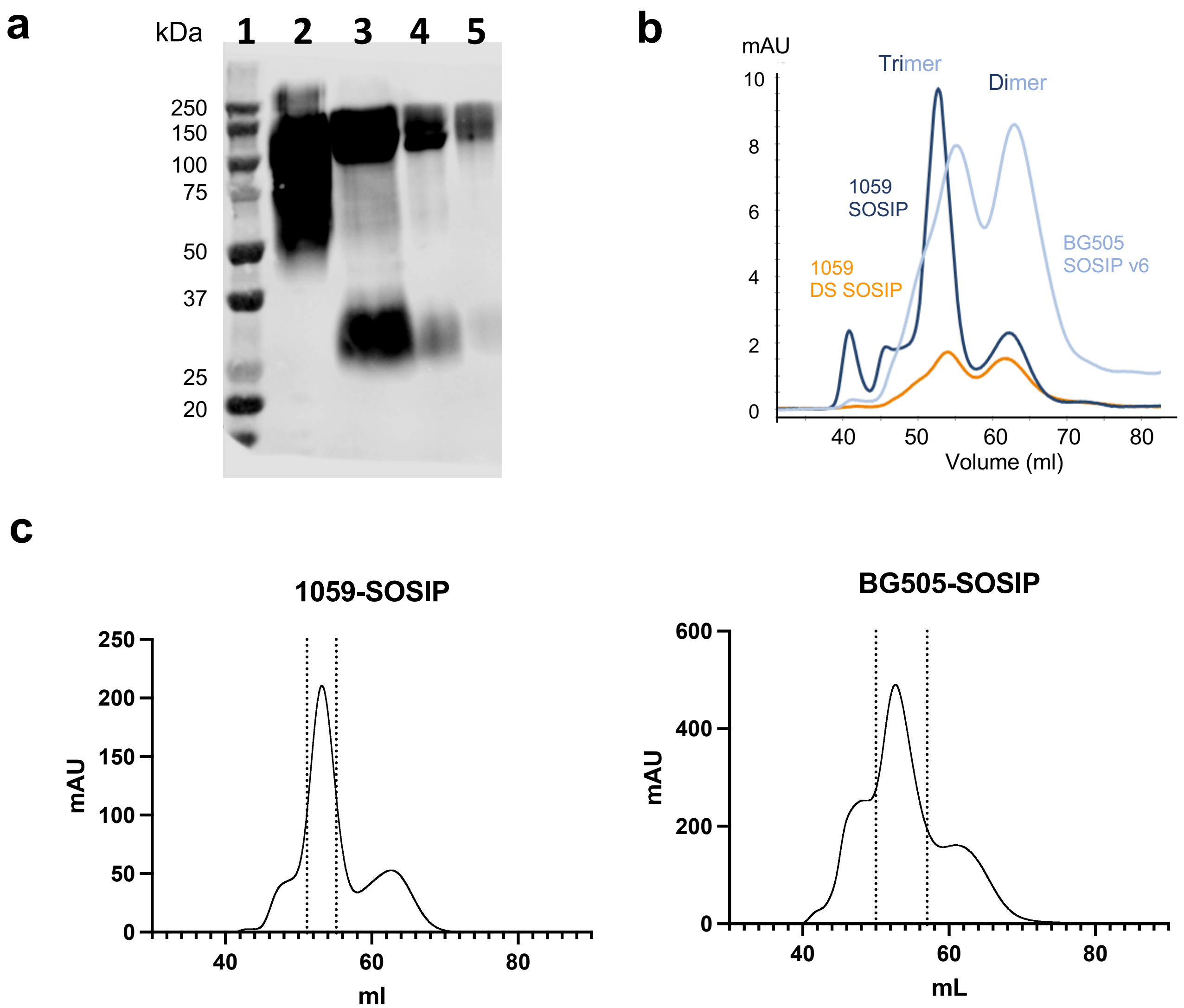

Supplementary Figure 6. Soluble 1059-SOSIP Env variants. a, Western blot of different 1059-SOSIP variants expressed in 293F cells for 4 days and analyzed directly from cell supernatants. We detected the SOSIP Env proteins using 1:10,000 dilution of serum of PLWH + 0.5μg/ml of JR52 antibody, which recognizes the D7324 epitope, followed by anti-human (1:10,000 dilution) + anti-mouse (1:20,000 dilution), both conjugated to horseradish peroxidase (HRP). Lanes: 1, ladder; 2, HIV-1_{AD8} gp120 control; 3, TPA WT; 4, TPA P22A (contains amino acid Alanine at position 22 of the signal peptide); 5, DS. TPA, signal peptide of the Tissue Plasminogen Activator; DS, 1059 SOSIP that contains the amino acid changes 201C and 433C (used only for the gel filtration in panel **b**). TPA-1059 SOSIP (lane 3) that includes only SOSIP mutations exhibited the highest expression levels and was used in all studies described in the main text. Blot is a representative of n = 2 independent experiments except for the TPA P22A variant that was tested only once. **b,** Comparison of size exclusion chromatography (SEC) profile of 1059 and BG505 SOSIP v6 trimers (both contained the signal peptide derived from the TPA). **c,** SEC profiles of 1059-SOSIP and BG505-SOSIP preparations used for determining structures of the unliganded Envs. The dashed vertical lines indicate the fractions that were pooled for downstream studies.

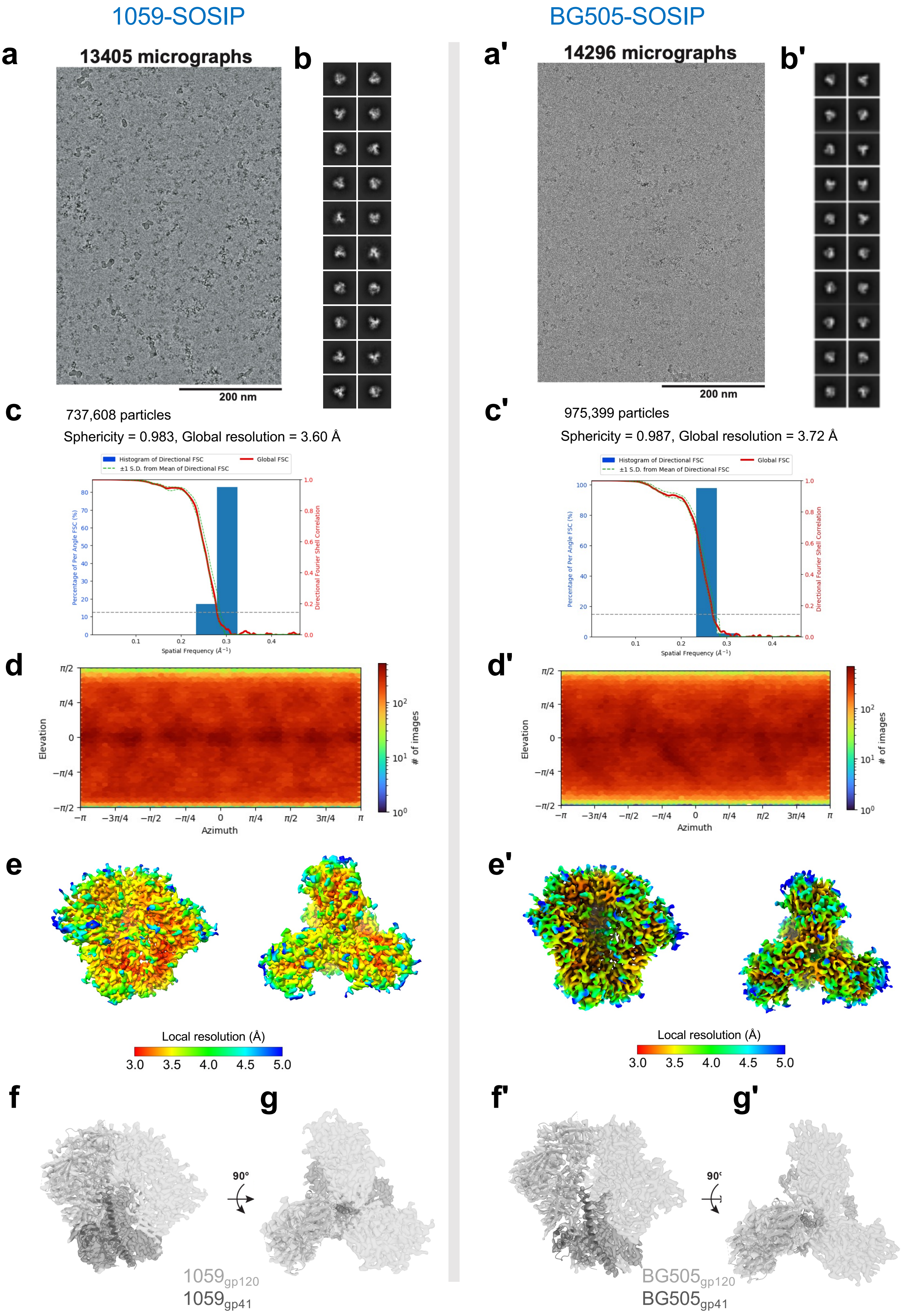
1059-SOSIP
BG505-SOSIP
a
13405 micrographs
200 nm
b
a'
14296 micrographs
200 nm
b'
c
737,608 particles
Sphericity = 0.983, Global resolution = 3.60 Å
c'
975,399 particles
Sphericity = 0.987, Global resolution = 3.72 Å
Histogram of Directional FSC
±1 S.D. from Mean of Directional FSC
Global FSC
Percentage of Per Angle FSC (%)
Directional Fourier Shell Correlation
Spatial Frequency (Å^{-1})
d
d'
Elevation
Azimuth
# of images
e
e'
Local resolution (Å)
3.0 3.5 4.0 4.5 5.0
f
g
f'
g'
90°
1059$_{gp120}$
1059$_{gp41}$
BG505$_{gp120}$
BG505$_{gp41}$

Supplementary Figure 7. Summary of cryo-EM data processing and validation of unliganded 1059 (left) and reference control unliganded BG505 (right) SOSIPs. a,a', Representative micrograph and **b,b',** Cryo-EM 2D class averages for 1059 or BG505 SOSIP cryo-EM data collection. **c,c'**, Gold-standard Fourier shell correlation (FSC) plot for the map. Estimate is reciprocal of spatial frequency at Fourier shell correlation value of 0.143 (horizontal dashed line). **d,d',** Viewing direction distribution plot generated by CryoSPARC v4.4.1 for the final reconstruction of 1059 SOSIP (**d**) and BG505 SOSIP (**d'**). **e,e',** Local resolution estimation of the 1059 / BG505 SOSIPs. **f-g,f'-g',** Side and top views of rigid body fit for one protomer of an Env trimer from the BG505-IOMA-10-1074 model (PDB 5T3Z; IOMA and 10-1074 Fabs removed) shown in cartoon and SOSIP cryo-EM densities.

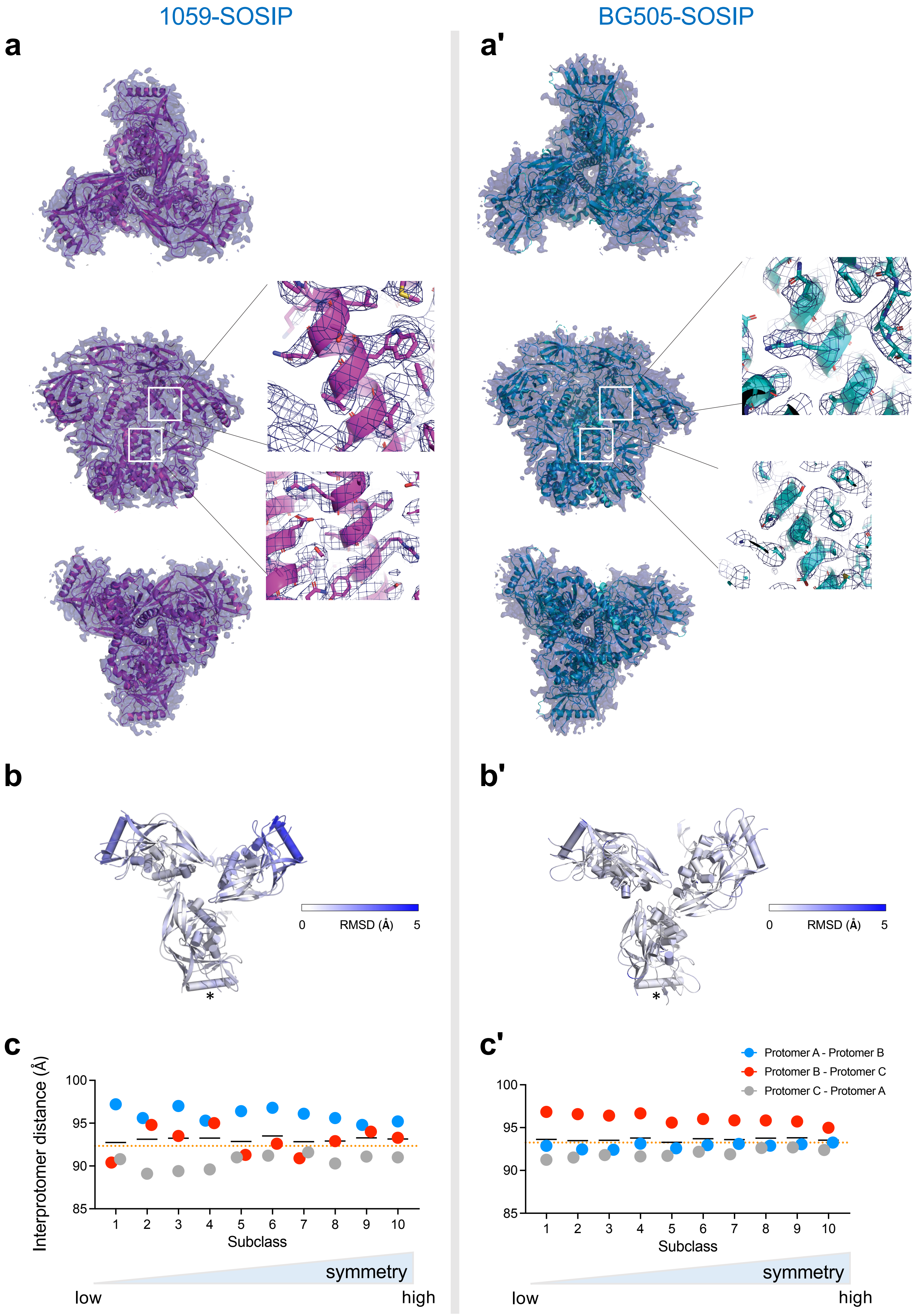
1059-SOSIP
BG505-SOSIP
a
a'
b
b'
0
RMSD (Å)
5
*
c
c'
Interprotomer distance (Å)
100
95
90
85
1 2 3 4 5 6 7 8 9 10
Subclass
Protomer A - Protomer B
Protomer B - Protomer C
Protomer C - Protomer A
symmetry
low
high

Supplementary Figure 8. Conformational flexibility and asymmetry of 1059-SOSIP. a,a', Coordinate models fit in density maps for 1059 (left) and BG505 (right) SOSIP cryo-EM datasets. Views from the gp120 (top), side (middle) and gp41 (bottom) are shown. Two helices from each are shown zoomed in with side chains shown as sticks. **b,b'**, We assessed motions of protomers in 1059- and BG505-SOSIP Envs by measuring the distance between each residue in the 2 most distanced subclasses (out of 10 shown in Fig. 2b) of each SOSIP (i.e. 2 subclasses with the highest C**α** root mean square deviation between them). The Env residues were colored according to the distance between same residue in the two chosen conformations. Data processing for generating these subclasses in described in the Methods section. Number of particles used, and resolution of each sub-class model are provided in Extended Data Table 4. **c,c'**, Interprotomer distance analysis of the 10 sub-classes shown in Fig. 2b. Distances was measured between residue 343 (HXBc2 numbering corresponding to Arg in 1059-SOSIP or Gly in BG505-SOSIP) of different protomers, and the sub-classes were ordered according to their symmetry based on the sum of differences of the interprotomer distances from the geometric mean of the interprotomer distances as described in the Methods section.

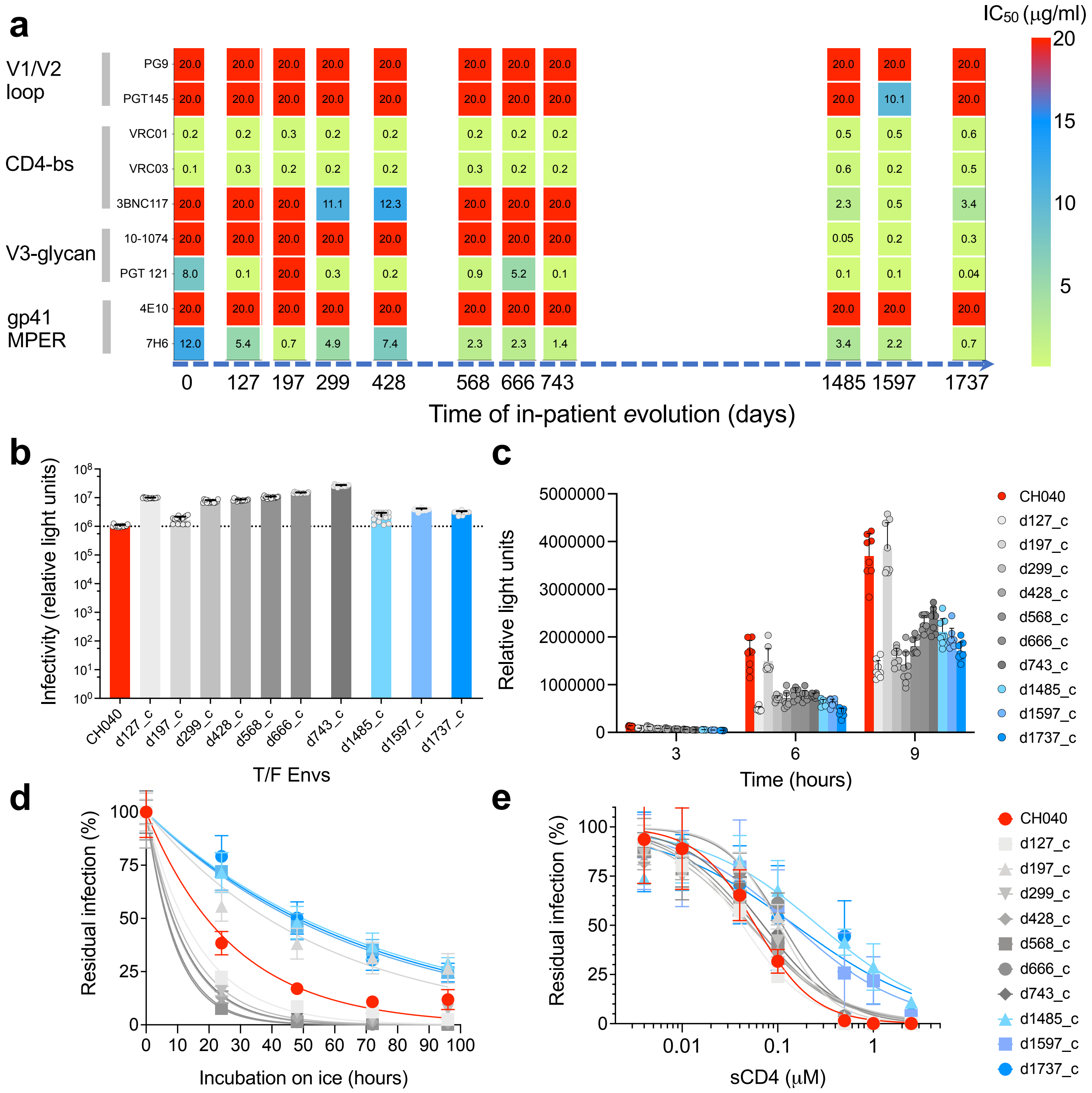

Supplementary Figure 9. Reconstructing the evolutionary pathways of (preexisting) multi-bnAb resistant T/F strain (CH040) in infected individual. a, Sensitivity of viruses pseudotyped with reconstructed consensus Envs to bnAbs targeting the five known sites of Env vulnerability. Envs from 10 different time points (day 127 to day 1737) were reconstructed based on consensus sequences after alignment and analysis of all available sequences for each time point (see Extended Data Table 2 and Supplementary Table 2). Indicated values are IC_{50s} in μg/ml and were used for Fig. 3d plots. **b,** Infectivity levels of reconstructed-Env pseudoviruses; 2ng p24 of each pseudovirus were used to infect Cf2Th-CD4/CCR5 target cells. For CH040, 2.5 ng p24 was used and the measurements were normalized to 2 ng p24. **c,** Cell-cell fusion activity. Env-expression plasmids of specified Envs were co-transfected with Tat-expression plasmid (5:1 Env:Tat ratio) into 293T cells and after 48-hours transfected cells were detached with PBS/5mM EDTA and incubated with TZM-bl cells. Extent of fusion was measured by firefly luciferase activity, which is expressed in the fused cells by Tat-mediated activation and used as a reporter. **d-e,** Sensitivity of reconstructed-Env pseudoviruses to cold exposure (**d**) and to sCD4 (**e**). Color codes for panels (**d**) and (**e**) are identical and shown on the right. Data are mean (relative light units or residual infection) ± s.d. of n = average (**a**, **d** and **e**) or representative (**b** and **c**) of 2 independent experiments each performed with 2-16 replicates.

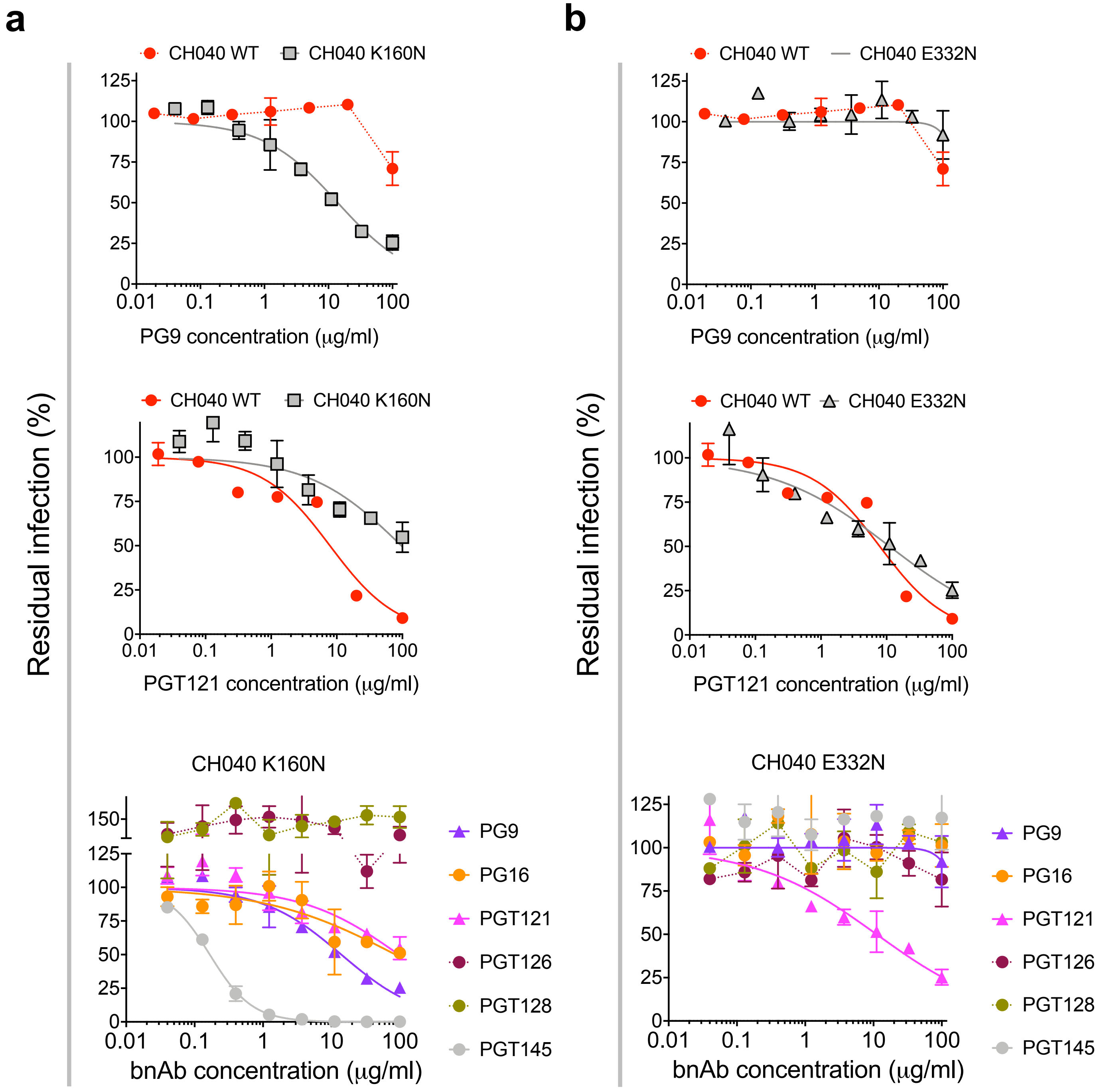

Supplementary Figure 10. Sensitivity of K160N and E332N variants of CH040 Env to different bnAbs. Effects of bnAbs targeting gp120 V1/V2 loop (PG9, PG16, and PGT145) and bnAbs targeting gp120 V3 glycan (PGT 121, PGT126, and PGT128) on the entry of pseudoviruses displaying the K160N (**a**) and E332N (**b**) CH040 Env variants. Data are mean values ± s.d. of representatives from n = 2 independent experiments performed in duplicates.

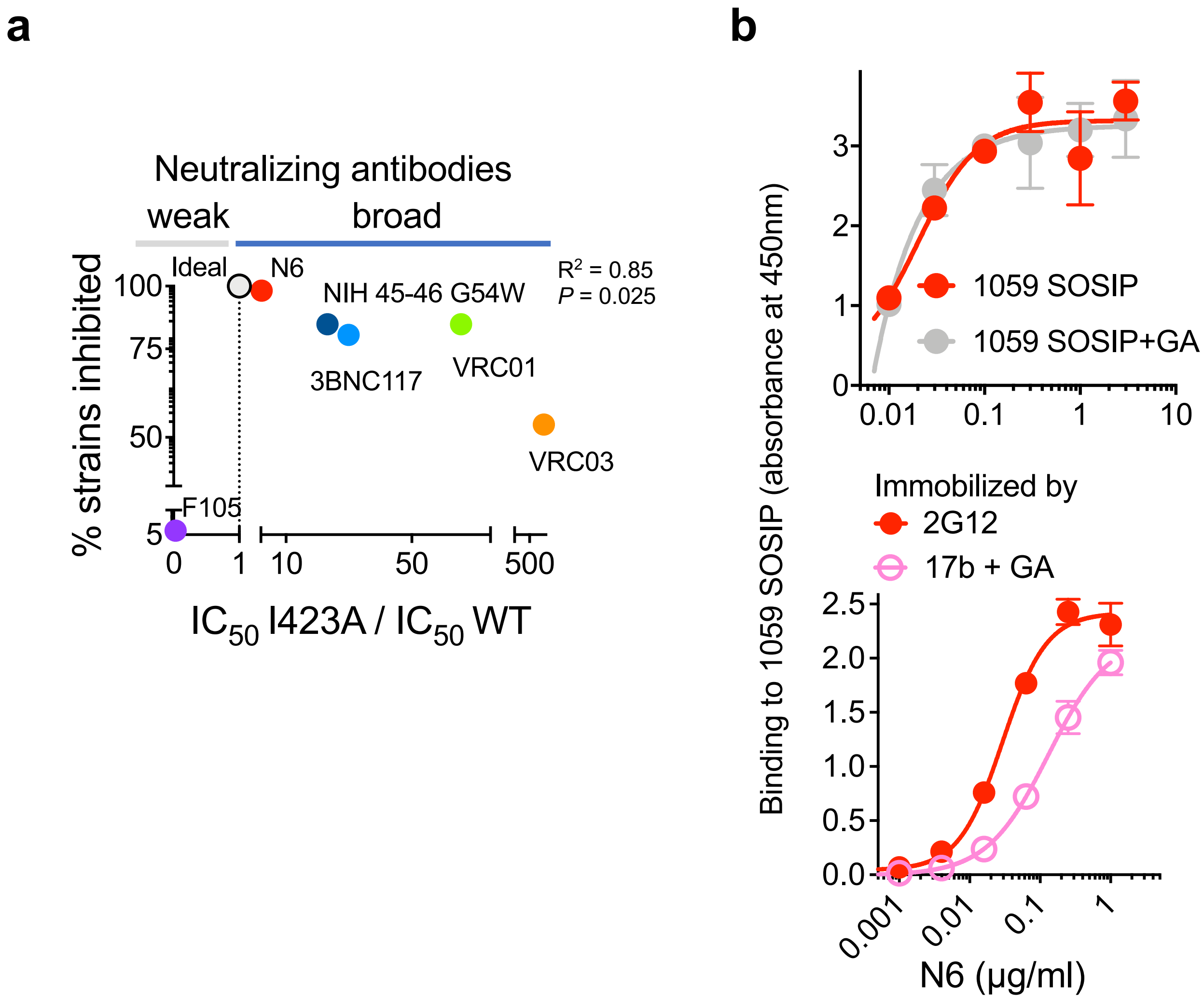

Supplementary Figure 11. Recognition of multiple HIV-1 Env conformations by CD4bs antibodies. a, Comparison between breadth and efficiency to neutralize both HIV-1_{JRFL} WT and intermediate HIV-1_{JRFL} I423A conformations by broadly and weakly CD4bs neutralizing antibodies. This plot extends the panel f of Fig. 4 (right) and show the position of an ideal bnAb and of the weakly neutralizing antibody F105. P, two-tailed P value of Pearson correlation (only for the bnAbs: N6, NIH45/46, 3BNC117, VRC01, and VRC03). **b, Top,** Comparison of N6 binding to 1059 SOSIP that was crosslinked by glutaraldehyde (GA) to restrict Env transitions and the binding to non-crosslinked 1095 SOSIP. **Bottom,** Comparison of N6 binding to 1059 SOSIP that was immobilized via 2G12 antibody and 1059 SOSIP immobilized by 17b antibody and further crosslinked by GA to stabilize open Env conformation. Data are mean values ± s.d. from n = 2 - 4 technically independent experiments.

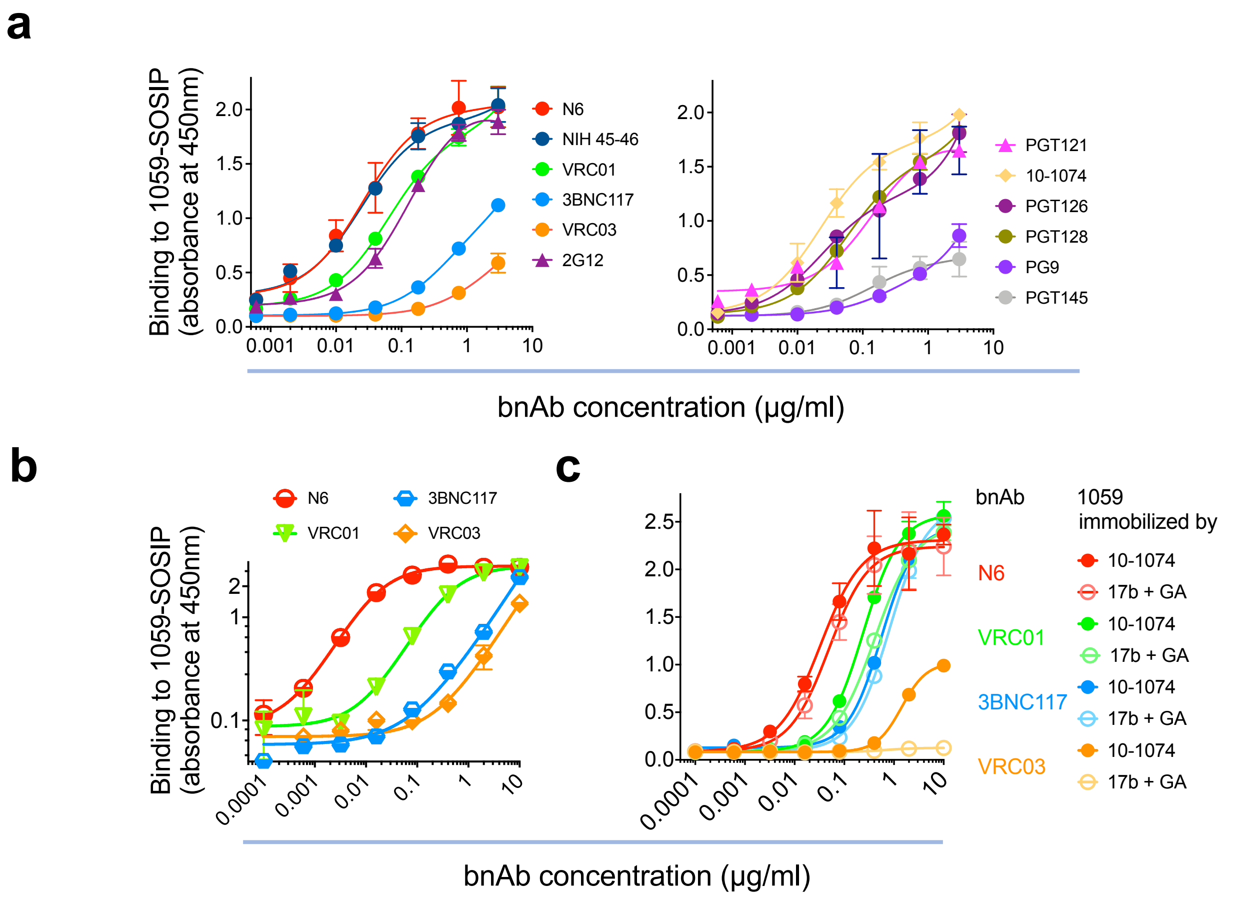

Supplementary Figure 12. Antibody binding to soluble SOSIP trimers. a, We measured bnAb binding to directly immobilized soluble 1059-SOSIP trimer by ELISA using anti-human IgG conjugated to HRP. **b-c**, Binding of CD4bs bnAbs to 1059 SOSIP immobilized by *Galanthus nivalis* lectin (**b**), 10-1074 (**c;** closed circles) or 17b and crossed linked (**c** open circles). Data are mean values ± s.d. from n = 2 - 4 technically independent experiments.

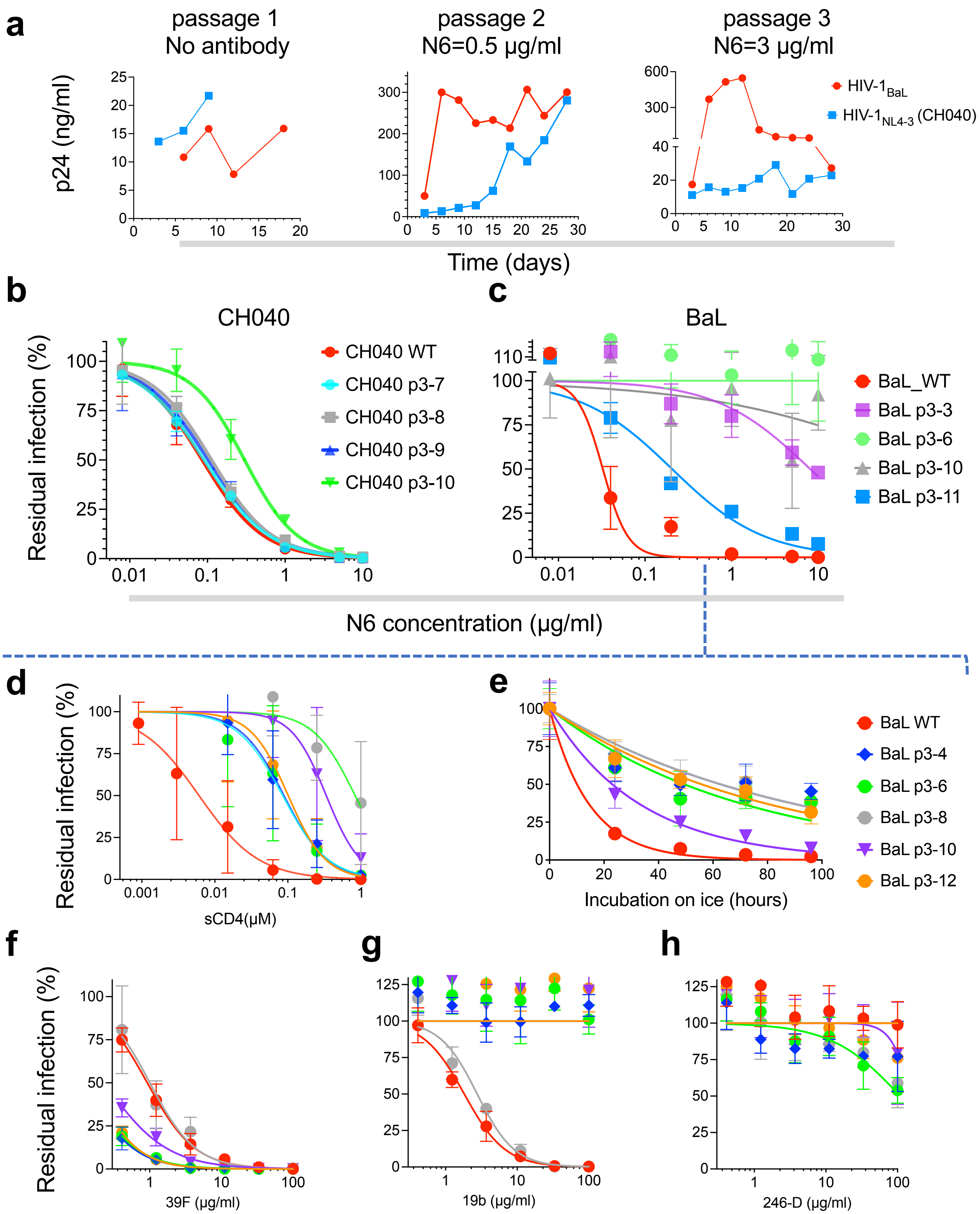

Supplementary Figure 13. *in vitro* adaptation of HIV-1 to N6. **a,** Levels of p24 in the supernatant of SupT1.R5 cells infected with HIV-1_{NL4-3} (CH040) or HIV-1_{BaL} during HIV-1 replication (passage 1), and adaptation to 0.5 µg/ml (passage 2) or 3 µg/ml (passage 3) N6. **b,c,** Sensitivity of Envs from single clones of adapted HIV-1_{NL4-3} (CH040) (**b**) or HIV-1_{BaL} (**c**) to N6 measured using a single-round infection assay. **d-h,** Sensitivity of the most N6-resistant Envs, isolated as single clones of N6-adapted HIV-1_{BaL}, to different Env ligands that preferentially recognize intermediate and open Env conformations (**d,f-h**), and to exposure to cold (**e**). Color code of the different clones in panels **d-h** are shown on the right of panel **e**. Data are mean values of 2-technically independent experiments (**a**) or mean values ± s.d. of n = 2 independent experiments, each performed in duplicates (**b-h**), except for the BaL p3-3 and BaL p3-11 clones in panel **c** that were still sensitive to N6 and were tested only once in duplicate.

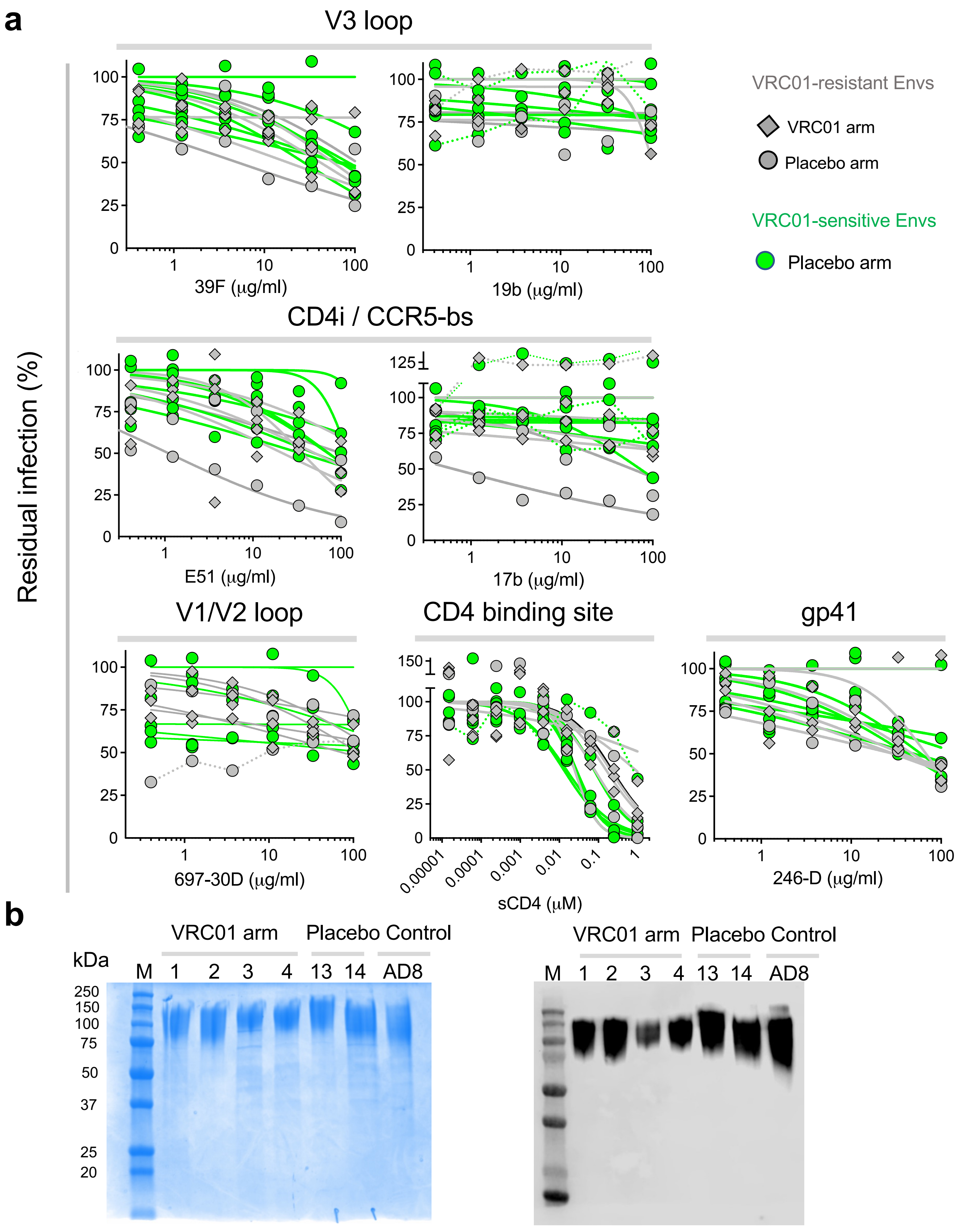

Supplementary Figure 14. Sensitivity of HIV-1 Envs, isolated from the antibody (VRC01)-mediated prevention (AMP) trial, to different Env ligands that preferentially neutralize Env open conformation and gp120 expression of Envs from VRC01-resistant strains. a, Sensitivity of VRC01-resistant (grey) and VRC01-sensitive Envs (green) to internal-epitope antibodies and soluble CD4. Data are mean values; n = 2-5 independent experiments, each performed with 2-4 replicates. **b,** SDS-PAGE (left) and western blot (right) of gp120 of VRC01-resistant HIV-1 strains. gp120 IDs are the same as those used in Fig. 5f-g. Gel and blot are representatives of *n* = 2 independent experiments.

Supplementary Table 1. Sensitivity of WT and intermediates HIV-1_{JRFL} Envs to internal-epitope ligands

	Relative infectivity (%)	IC_{50}					Counts (only single Env element <10%)				
		sCD4 (nM)	19b (µg/ml)	17b (µg/ml)	902090 (µg/ml)	T20 (nM)					
JR-FL*		Average	Average	Average	Average	Average	sCD4<2	19b<5	17b<15	902090<10	T20<0.23
WT	100	19.04	50	150	100	2.3	0	0	0	0	0
V127A	3	1.39	0.11	76.03	100	1.29	0	0	0	0	0
I154A	51	1.09	2.33	0.33	17.89	4.16	0	0	0	0	0
N156A	19	1.02	0.56	31.2	~5	85.01	0	0	0	0	0
L175A	172	0.91	2.52	60.18	5.21	4.25	0	0	0	0	0
Y177A	56	1.66	0.67	21.26	100	2.95	0	0	0	0	0
V181I	280	41.86	50	150	100	0.67	0	0	0	0	0
V181I+L193A	130	0.39	0.05	0.43	17.1	1.28	0	0	0	0	0
I184L+L193A	60	0.82	0.018	0.44	16.6	3.29	0	0	0	0	0
Y191A	87	1	6.75	65.35	26.43	3.76	1	0	0	0	0
Y191A+I423A	33	0.82	0.044	11.9	100	1.32	0	0	0	0	0
Y191A+Q428A	38	0.64	0.67	19.69	2.27	0.79	0	0	0	0	0
L193A	16	1.98	0.19	0.65	7.56	5.15	0	0	0	0	0
L193A + I201W	36	1.27	0.13	3.67	9.26	1.22	0	0	0	0	0
L193A + I423V	1012	0.67	0.04	0.29	3.9	2.1	0	0	0	0	0
L193A + I423V-D674N	63	0.37	0.027	0.18	7.49	1.33	0	0	0	0	0
L193A + I423V-Δ733	1571	2.00	0.38	7.39	25.6	7.16	0	0	0	0	0
L193A + I423V-Δ765	655	0.68	0.09	3.3	12.78	1.45	0	0	0	0	0
L193G	25	0.76	0.11	0.76	0.84	0.72	0	0	0	0	0
L193K	17	0.75	0.02	0.09	1.20	2.69	0	0	0	0	0
L193I	47	11.42	50.00	150	100	5.48	0	0	0	0	0
L193V	70	5.21	13.25	150	55.96	2.36	0	0	0	0	0
L193E	43	0.61	0.11	1.05	1.60	0.39	0	0	0	0	0
L193D	7	0.32	0.09	1.34	3.01	3.50	0	0	0	0	0
L193M	39	2.60	50.00	150	43.96	0.27	0	0	0	0	0
L193S	16	0.40	0.08	0.60	2.60	1.82	0	0	0	0	0
L193R	18	0.23	0.02	0.26	0.83	0.67	0	0	0	0	0
L193W	69	0.22	1.16	2.26	13.53	3.75	0	0	0	0	0
L193Q	18	1.17	0.12	0.66	3.58	2.12	0	0	0	0	0
L193F	70	3.20	23.84	150	11.24	2.71	0	0	0	0	0
L193H	8	0.33	0.06	0.81	3.72	0.83	0	0	0	0	0
L193T	22	0.93	0.05	0.98	0.68	1.87	0	0	0	0	0
L193Y	17	0.37	0.14	0.29	4.98	1.10	0	0	0	0	0
L193P	16	0.41	0.13	0.97	5.27	4.86	0	0	0	0	0
T320R	76	3.14	0.28	41.95	100	3.92	0	1	0	0	0
S375W	9	6.24	50	150	100	0.33	0	0	0	0	0
I420A	159	60.24	3.08	150	2.03	0.66	0	0	0	0	0
K421A	199	1.05	4.8	9.59	27.19	0.79	0	0	0	0	0
Q422A	72	1.11	2.73	150	22.76	2.14	0	0	0	0	0
Q422D	7	0.89	0.24	5.94	6.56	0.67	0	0	0	0	0
Q422N	7	6.59	1.12	45.86	28.18	0.73	0	1	0	0	0
I423A	696	1.22	0.1	39.22	1.57	0.21	0	0	0	0	0
I423V	943	19.92	47.8	150	100	1.88	0	0	0	0	0
I424A	27	21.74	0.013	150	16.06	2.73	0	1	0	0	0
N425A	590	335.4	50	150	100	0.27	0	0	0	0	0
M426A	214	254.2	50	150	100	0.22	0	0	0	0	1
Q428A	532	135.4	50	150	100	0.18	0	0	0	0	1
E429A	1127	13.7	50	150	24.65	2.9	0	0	0	0	0
V430A	18	500	50	150	68.32	1.35	0	0	0	0	0
G431A	752	25.27	50	150	59.34	2.13	0	0	0	0	0
K432A	579	29.89	50	150	100	2.56	0	0	0	0	0
A433G	2084	14.46	9.49	150	21.15	1.45	0	0	0	0	0
M434A	10	7.2	0.84	69.58	22.51	1.77	0	1	0	0	0
Y435A	9	46.27	0.71	150	15.87	1.29	0	1	0	0	0

Average WT infectivity (relative light units)	1907095.3
Average infectivity (%)	243.3
Range infectivity (%)	3 - 2084

	%					
	sCD4	19b (V3)	17b (B20-B21)	902090 (V1/V2)	T20 (gp41 HR1)	Total
Criteria	< 2nM	< 5 ug/ml	< 15 ug/ml	< 10 ug/ml	< 0.23 nM	
Number of variants out of 53 in each group	31.0	37.0	24.0	20.0	3.0	115
% of variants meeting criteria in each group (Number of varaints in each group / 53 *100)	58.5	69.8	45.3	37.7	5.7	
% of variants in each group from the total (Number of varaints in each group / 115 *100)	27.0	32.2	20.9	17.4	2.6	100

	Number of single site exposures				
	sCD4	19b (V3)	17b (B20-B21)	902090 (V1/V2)	T20 (gp41 HR1)
Criteria (only one of the following)	< 2nM	< 5 ug/ml	< 15 ug/ml	< 10 ug/ml	< 0.23 nM
Number of variants	1	5	0	0	2

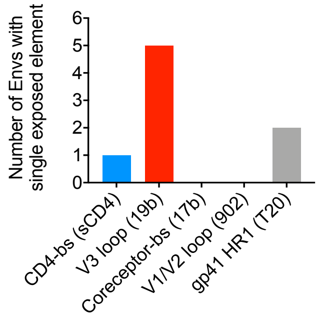

* Distribution of exposed elements among 53 functional Env intermediates. We selected HIV-1_{JRFL} Env variants, which are enriched in intermediates based on prior studies (this is not a random selection). WT and 53 Env mutants were analyzed for sensitivity to ligands that preferentially recognize open Env conformations (17b, 19b, sCD4, 902 directed against the V1/V2 loop, and T20). Env mutants that were > 10 times more sensitive than WT (IC_{50} mutant / IC_{50} WT < 0.1) were identified and their prevalence was calculated. IC_{50} of some Env mutants has been published previously (Herschhorn et al. mBio 2016; Nat Commun 2017).

* Env variants that showed >10 fold hypersensitivity to any of the ligands (17b, 19b, sCD4, 902 directed against the V1/V2 loop, and T20) are in blue.

Supplementary Table 2. HIV-1 transmitted/founder Envs

#	Env ID*	Env clone**	Tier	Fiebig Stage	Accession Number
1	1012	p1012.TC21.3257	1B or 2	III	EU289184
2		p1006_11.C3.1601	2	III	EU289183
3	1054	p1054.TC4.1499	2	II	EU289185
4		p1056.TA11.1826	1B or 2	II	EU289186
5	1058	p1058_11.B11.1550	2	IV	EU289187
6	1059	p1059_09.A4.1460	2	III	EU289188
7		p62357_14.D3.4589	2	II	EU289189
8	6244	p6244_13.B5.4567	2	II	EU289191
9		p6240_08.TA5.4622	2	II	EU289190
10	63358	p63358.p3.4013	2	II	EU289192
11	CH040***	p700010040.C9.4520	2	II	EU289193
12		p700010058.A4.4375	2	III	EU289194
13		p9014_01.TB1.4769	2	II	EU289195
14	9021	p9021_14.B2.4571	2	II	EU289196
15	PRB926	pPRB926_04.A9.4237	2	II	EU289197
16		pPRB931_06.TC3.4930	2	III	EU289198
17	PRB958	pPRB958_06.TB1.4305	2	III	EU289199
18	SC05	pSC05.8C11.2344	2	II	EU289200
19	SC45	pSC45.4B5.2631	2	II	EU289201
20	WEAU	pWEAUd15.410.5017	2	II	EU289202

* Env ID is shown for the 13 T/F Env clones that were selected for testing in the current study
** All Env-expressing plasmids (Env clones) of T/F strains were kindly provide by Drs. Beatrice H. Hahn, Brandon F. Keele and George M. Shaw through the NIH AIDS Reagent Program
*** An infectious molecular clone is available (pCH040.c/2625; Catalog number 11740); subject identifier 700010040

Supplementary Table 3. Neutralization preferences of different ligands to Env conformations and the effects of experimental condition on HIV-1 entry

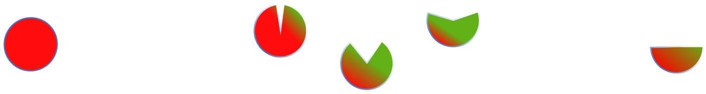

	Closed	Functional intermediates*	Open
Cold exposure	Resistant	Sensitive	Sensitive
sCD4 / CD4 mimetics	Moderately sensitive	Sensitive	Hypersensitive
Conformational blockers	Hypersensitive	Moderately resistant	Resistant
CD4-independent infection	Low	Moderate	Relatively high
VRC01	Sensitive	Relatively resistant	Resistant**
VRC03	Sensitive	Relatively resistant	Resistant**
3BNC117	Sensitive	Relatively resistant	Resistant**
4E10	Moderately sensitive	Sensitive	Hypersensitive
7H6	Sensitive	Sensitive	Hypersensitive
PGT151	Sensitive	Sensitive	Hypersensitive
VRC034	Relatively sensitive	Sensitive	Sensitive
PG9	Sensitive	Relatively resistant	Resistant
17b	Resistant	Relatively sensitive	Sensitive
19b	Resistant	Relatively sensitive	Sensitive
Patient serum	Generally resistant	Sensitive	Hypersensitive
T20	Sensitive	Sensitive	Hypersensitive

*By definition an intermediate phenotype can result from intermediate sensitivity to Env ligands that target internal epitopes or to cold, or it can result from sensitivity to some of these ligands/cold. In addition, resistance to a specific inhibitor can be the result of changes in the epitope/binding site and not necessary changes in Env conformation but resistance of multiple molecules targeting the same site is likely related to conformational changes.

** lab-adapted HIV-1 Envs are sensitive to these bnAbs, but Envs of primary strains that are engineered to be open could potentially be resistant.

Supplementary Table 4. Asymmetry calculation

1059 SOSIP sub-classes

AB	BC	CA	sum	geomean
97.2	90.4	90.8	278.4	92.75
95.6	94.8	89.1	279.5	93.12
97	93.5	89.4	279.9	93.25
95.3	95	89.6	279.9	93.26
96.4	91.3	91	278.7	92.87
96.8	92.6	91.2	280.6	93.50
96.1	90.9	91.6	278.6	92.84
95.6	92.9	90.3	278.8	92.91
94.8	94	91.1	279.9	93.29
95.2	93.3	91	279.5	93.15

**GeoMean 1059**	**∑(abs(X-gmean))**
92.47	8.47
	8.83
	8.63
	8.23
	6.57
	5.73
	6.07
	5.73
	5.23
	5.03
Average	6.85
GeoMean	6.70
STDEV	1.52

BG505 SOSIP sub classes

AB	BC	CA	sum	geomean
92.88	96.84	91.24	280.96	93.62
92.45	96.57	91.5	280.52	93.48
92.42	96.4	91.8	280.62	93.52
93.12	96.66	91.64	281.42	93.78
92.59	95.59	91.71	279.89	93.28
92.96	96.01	92.18	281.15	93.70
93.1	95.85	91.89	280.84	93.60
92.89	95.84	92.63	281.36	93.78
93.06	95.72	92.69	281.47	93.81
93.25	94.98	92.39	280.62	93.53

**GeoMean BG505**	**∑(abs(X-gmean))**
93.17	5.89
	5.79
	5.35
	5.07
	4.46
	4.04
	4.03
	3.49
	3.14
	2.67
Average	3.10
GeoMean	3.08
STDEV	0.42

Different SOSIP structures

		AB	BC	CA	sum	geomean
	4ZMJ	91.7	91.7	91.7	275.1	91.70
	5TZ3	91.9	91.9	91.9	275.7	91.90
	5V8L	92.1	92.1	92.1	276.3	92.10
	5ACO	91.8	91.8	91.7	275.3	91.77
	5VN3	91.6	91.6	91.5	274.7	91.57
	5U1F	94.8	94.4	94.4	283.6	94.53
*	**BG505-Env**	**95.64**	**92.27**	**91.66**	**183.93**	**93.17**
*	**1059-Env**	**95.26**	**92.53**	**89.7**	**277.49**	**92.47**
	5FUU	95.9	91	89.4	276.3	92.06

* Current study

Supplementary Table 5. Subclassification of unliganded 1059- and BG505-SOSIP cryo-EM datasets. The table shows the number of particles in each class and the resolution of each of the 10 structures after subclassification.

Unliganded 1059-SOSIP			Unliganded BG505-SOSIP		
Class ID	Particles	Resolution (Å)	Class ID	Particles	Resolution (Å)
1	53,177	3.93	1	84,089	4.16
2	71,533	3.79	2	64,582	4.22
3	57,181	3.82	3	69,147	4.24
4	57,278	3.96	4	81,171	4.14
5	45,804	4.04	5	278,726	3.93
6	69,801	3.74	6	86,261	4.11
7	57,124	4.06	7	85,273	4.14
8	50,430	4.01	8	79,022	4.22
9	55,435	4.12	9	81,500	4.18
10	219,825	3.72	10	65,628	4.23
Total	737,588		Total	975,399	

Supplementary Table 6. Constructing consensus HIV-1 Env sequences of 10 time points during in-patient evolution of CH040. Analysis of the dominant changes in consensus sequences for each time point during evolution of HIV-1 Envs in patient CH040.

	Time points (days)*															
	<16	d16	d32	d61	d127**	d197	d299	d428	d568	d666	d743	d1485	d1513	d1569	d1597	d1737
# seq available	7	22	33	28	67	32	39	37	34	45	25	30			35	41
Dominant changes (>50% strains except when indicated)	none	none	none	none												
									G135E	G135E						
									N136K	N136K						
										V137G (49%)						
								N139T			N139T	N139T				N139T
																N142M
																S143G
							N144K		N144T							
									G146E			G146E			G146E	G146E
					E147K	E147G		E147T	E147K		E147T	E147T			E147T	E147T
								M148L			M148L	M148L			M148L (inset)	
												K160R			K160R	K160R
									I165V			I165M			I165M	I165M
												K166Q			K166Q	K166Q
															R-to-S (188?)	
												I234N			I234N	I234N
							T295N		T295N	T295N	T295N	T295N			T295N	T295N
							N300H		N300H	N300H	N300H					
												P308H			P308H	P308H
												D321A			D321G	D321A
								T323I	T323I	T323I	T323I	T323I			T323I	T323I
						R327K										
								Y330S				Y330H			Y330H	Y330H
						E332K		E332K				E332N			E332N	E332N
												N334S			N334S	N334S
															S340N	
										E347G						
										Q352K						
															R363H	R363H
															W401G	
												K405E			K405N	K405N
															D409N	
								G412D		G412D						
								I415M		I415M		I415T			I415T	I415T
									K419R	K419R	K419R	K419R			K419R	K419R
																G429E
								K442I	I422E (42%)	I442Q (48%)	K442I	K442I			K442I	K442I
												R444K			R444K	R444K
												E463K			E463K	E463K
															D636N	D636N
															L663W	
															L721F	
							A754T	A754T	A754T	A754T		A754T			A754T	A754T
															V778A	
												R770H				
															N809K	
							I820T	I820T	I820T	I820T						
												C837G			C837G	C837G
												L841R			L841R	L841R

* Sequences from 10 time points highlighted in yellow were used for reconstruction of consensus viruses
** Consensus of sequences from day 127 and day 148 contained a single change and they were combined

Supplementary Table 7. Env sequences used for building consensus sequences for 10 time points: day 127 (d127) - day 1737 (d1737)

	d127*	d197	d299	d428	d568	d666	d743	d1485	d1597	d1737
# seq available	67	32	39	37	34	45	25	30	35	41
Dominant changes (>50% strains except when indicated)										
					G135E	G135E				
					N136K	N136K				
						V137G (49%)				
				N139T			N139T	N139T		N139T
										N142M
										S143G
			N144K		N144T					
					G146E			G146E	G146E	G146E
	E147K	E147G		E147T	E147K		E147T	E147T	E147T	E147T
				M148L			M148L	M148L	M148L (inset)	
								K160R	K160R	K160R
					I165V			I165M	I165M	I165M
								K166Q	K166Q	K166Q
									R-to-S (188?)	
								I234N	I234N	I234N
			T295N		T295N	T295N	T295N	T295N	T295N	T295N
			N300H		N300H	N300H	N300H			
								P308H	P308H	P308H
								D321A	D321G	D321A
				T323I	T323I	T323I	T323I	T323I	T323I	T323I
		R327K								
				Y330S				Y330H	Y330H	Y330H
		E332K		E332K				E332N	E332N	E332N
								N334S	N334S	N334S
									S340N	
						E347G				
						Q352K				
									R363H	R363H
									W401G	
								K405E	K405N	K405N
									D409N	
				G412D		G412D				
				I415M		I415M		I415T	I415T	I415T
					K419R	K419R	K419R	K419R	K419R	K419R
										G429E
				K442I	I422E (42%)	I442Q (48%)	K442I	K442I	K442I	K442I
								R444K	R444K	R444K
								E463K	E463K	E463K
									D636N	D636N
									L663W	
									L721F	
			A754T	A754T	A754T	A754T		A754T	A754T	A754T
									V778A	
								R770H		
									N809K	
			I820T	I820T	I820T	I820T				
								C837G	C837G	C837G
								L841R	L841R	L841R

* Consensus of sequences from day 127 and day 148 contained a single change and they were combined

Aligned using an external algorithm

Consensus

1. B.US.2006.700010040_C9_4520.EU289193
2. B.US.2006.CH0040_3_d0127_ipe032_27_49.MG900133
3. B.US.2006.CH0040_3_d0127_ipe032_2_02.MG900134
4. B.US.2006.CH0040_3_d0127_ipe032_2_03.MG900135
5. B.US.2006.CH0040_3_d0127_ipe032_2_04.MG900136
6. B.US.2006.CH0040_3_d0127_ipe032_2_06.MG900137
7. B.US.2006.CH0040_3_d0127_ipe032_2_10.MG900138
8. B.US.2006.CH0040_3_d0127_ipe032_2_11.MG900139
9. B.US.2006.CH0040_3_d0127_ipe032_2_13.MG900140
10. B.US.2006.CH0040_3_d0127_ipe032_2_14.MG900141
11. B.US.2006.CH0040_3_d0127_ipe032_2_15.MG900142
12. B.US.2006.CH0040_3_d0127_ipe032_2_17.MG900143
13. B.US.2006.CH0040_3_d0127_ipe032_2_18.MG900144
14. B.US.2006.CH0040_3_d0127_ipe032_2_19.MG900145
15. B.US.2006.CH0040_3_d0127_ipe032_2_20.MG900146
16. B.US.2006.CH0040_3_d0127_ipe032_2_21.MG900147
17. B.US.2006.CH0040_3_d0127_ipe032_2_22.MG900148
18. B.US.2006.CH0040_3_d0127_ipe032_2_24.MG900149
19. B.US.2006.CH0040_3_d0127_ipe032_2_26.MG900150
20. B.US.2006.CH0040_3_d0127_ipe032_2_28.MG900151
21. B.US.2006.CH0040_3_d0127_ipe032_2_29.MG900152
22. B.US.2006.CH0040_3_d0127_ipe032_2_31.MG900153
23. B.US.2006.CH0040_3_d0127_ipe032_2_32.MG900154
24. B.US.2006.CH0040_3_d0127_ipe032_2_36.MG900155
25. B.US.2006.CH0040_3_d0127_ipe032_2_38.MG900156
26. B.US.2006.CH0040_3_d0127_ipe032_2_39.MG900157
27. B.US.2006.CH0040_3_d0127_ipe032_2_41.MG900158
28. B.US.2006.CH0040_3_d0127_ipe032_2_42.MG900159
29. B.US.2006.CH0040_3_d0127_ipe032_2_43.MG900160
30. B.US.2006.CH0040_3_d0127_ipe032_2_46.MG900161
31. B.US.2006.CH0040_3_d0127_ipe032_2_51.MG900162
32. B.US.2006.CH0040_3_d0127_ipe032_2_52.MG900163
33. B.US.2006.CH0040_3_d0127_ipe032_2_54.MG900164
34. B.US.2006.CH0040_3_d0127_ipe032_2_56.MG900165
35. B.US.2006.CH0040_3_d0127_ipe032_2_57.MG900166
36. B.US.2006.CH0040_3_d0127_ipe032_2_58.MG900167
37. B.US.2006.CH0040_3_d0127_ipe032_2_59.MG900168
38. B.US.2006.CH0040_3_d0127_ipe032_2_61.MG900169
39. B.US.2006.CH0040_3_d0127_ipe032_3_01.MG900170
40. B.US.2006.CH0040_3_d0127_ipe032_81_50.MG900171
41. B.US.2006.CH0040_3_d0148_ipe032_2_01.MG900172
42. B.US.2006.CH0040_3_d0148_ipe032_2_02.MG900173
43. B.US.2006.CH0040_3_d0148_ipe032_2_03.MG900174
44. B.US.2006.CH0040_3_d0148_ipe032_2_05.MG900175
45. B.US.2006.CH0040_3_d0148_ipe032_2_08.MG900176
46. B.US.2006.CH0040_3_d0148_ipe032_2_11.MG900177
47. B.US.2006.CH0040_3_d0148_ipe032_2_13.MG900178
48. B.US.2006.CH0040_3_d0148_ipe032_2_14.MG900179
49. B.US.2006.CH0040_3_d0148_ipe032_2_15.MG900180
50. B.US.2006.CH0040_3_d0148_ipe032_2_16.MG900181
51. B.US.2006.CH0040_3_d0148_ipe032_2_18.MG900182
52. B.US.2006.CH0040_3_d0148_ipe032_2_21.MG900183
53. B.US.2006.CH0040_3_d0148_ipe032_2_32.MG900184
54. B.US.2006.CH0040_3_d0148_ipe032_2_35.MG900185
55. B.US.2006.CH0040_3_d0148_ipe032_2_36.MG900186
56. B.US.2006.CH0040_3_d0148_ipe032_2_43.MG900187
57. B.US.2006.CH0040_3_d0148_ipe032_2_46.MG900188
58. B.US.2006.CH0040_3_d0148_ipe032_2_48.MG900189
59. B.US.2006.CH0040_3_d0148_ipe032_2_51.MG900190
60. B.US.2006.CH0040_3_d0148_ipe032_2_52.MG900191
61. B.US.2006.CH0040_3_d0148_ipe032_2_53.MG900192
62. B.US.2006.CH0040_3_d0148_ipe032_2_55.MG900193
63. B.US.2006.CH0040_3_d0148_ipe032_2_58.MG900194
64. B.US.2006.CH0040_3_d0148_ipe032_2_61.MG900195

65. B.US.2006.CH0040_3_d0148_ipe032_2_64.MG900196
66. B.US.2006.CH0040_3_d0148_ipe032_2_65.MG900197
67. B.US.2006.CH0040_3_d0148_ipe032_2_66.MG900198
68. B.US.2006.CH0040_3_d0148_ipe032_9_42.MG900199

	MRVMGIRKNYQHLWREGILLLGILMICSAADNLWVTVYYGVPVWREATTTLFCASDAKAY	
1	MRVMGIRKNYQHLWREGILLLGILMICSAADNLWVTVYYGVPVWREATTTLFCASDAKAY	60
2	MRVMGIRKNYQHLWREGILLLGILMICSAADNLWVTVYYGVPVWREATTTLFCASDAKAY	60
3	MRVMGIRKNYQHLWREGILLLGILMICSAADNLWVTVYYGVPVWREATTTLFCASDAKAY	60
4	MRVMGIRKNYQHLWREGILLLGILMICSAADNLWVTVYYGVPVWREATTTLFCASDAKAY	60
5	MRVMGIRKNYQHLWREGILLLGILMICSAADNLWVTVYYGVPVWREATTTLFCASDAKAY	60
6	MRVMGIRKNYQHLWREGILLLGILMICSAADNLWVTVYYGVPVWREATTTLFCASDAKAY	60
7	MRVMGIRKNYQHLWREGILLLGILMICSAADNLWVTVYYGVPVWREATTTLFCASDAKAY	60
8	MRVMGIRKNYQHLWREGILLLGILMICSAADNLWVTVYYGVPVWREATTTLFCASDAKAY	60
9	MRVMGIRKNYQHLWREGILLLGILMICSAADNLWVTVYYGVPVWREATTTLFCASDAKAY	60
10	MRVMGIRKNYQHLWREGILLLGILMICSAADNLWVTVYYGVPVWREATTTLFCASDAKAY	60
11	MRVMGIRKNYQHLWREGILLLGILMICSAADNLWVTVYYGVPVWREATTTLFCASDAKAY	60
12	MRVMGIRKNYQHLWREGILLLGILMICSAADNLWVTVYYGVPVWREATTTLFCASDAKAY	60
13	MRVMGIRKNYQHLWREGILLLGILMICSAADNLWVTVYYGVPVWREATTTLFCASDAKAY	60
14	MRVMGIRKNYQHLWREGILLLGILMICSAADNLWVTVYYGVPVWREATTTLFCASDAKAY	60
15	MRVMGIRKNYQHLWREGILLLGILMICSAADNLWVTVYYGVPVWREATTTLFCASDAKAY	60
16	MRVMGIRKNYQHLWREGILLLGILMICSAADNLWVTVYYGVPVWKEATTTLFCASDAKAY	60
17	MRVMGIRKNYQHLWREGILLLGILMICSAADNLWVTVYYGVPVWREATTTLFCASDAKAY	60
18	MRVMGIRKNYQHLWREGILLLGILMICSAADNLWVTVYYGVPVWREATTTLFCASDAKAY	60
19	MRVMGIRKNYQHLWREGILLLGILMICSAADNLWVTVYYGVPVWREATTTLFCASDAKAY	60
20	MRVMGIRKNYQHLWREGILLLGILMICSAADNLWVTVYYGVPVWREATTTLFCASDAKAY	60
21	MRVMGIRKNYQHLWREGILLLGILMICSAADNLWVTVYYGVPVWREATTTLFCASDAKAY	60
22	MRVMGIRKNYQHLWREGILLLGILMICSAADNLWVTVYYGVPVWREATTTLFCASDAKAY	60
23	MRVMGIRKNYQHLWREGILLLGILMICSAADNLWVTVYYGVPVWREATTTLFCASDAKAY	60
24	MRVMGIRKNYQHLWREGILLLGILMICSAADNLWVTVYYGVPVWREATTTLFCASDAKAY	60
25	MRVMGIRKNYQHLWREGILLLGILMICSAADNLWVTVYYGVPVWREATTTLFCASDAKAY	60
26	MRVMGIRKNYQHLWREGILLLGILMICSAADNLWVTVYYGVPVWREATTTLFCASDAKAY	60
27	MRVMGIRKNYQHLWREGILLLGILMICSAADNLWVTVYYGVPVWREATTTLFCASDAKAY	60
28	MRVMGIRKNYQHLWREGILLLGILMICSAADNLWVTVYYGVPVWREATTTLFCASDAKAY	60
29	MRVMGIRKNYQHLWREGILLLGILMICSAADNLWVTVYYGVPVWREATTTLFCASDAKAY	60
30	MRVMGIRKNYQHLWREGILLLGILMICSAADNLWVTVYYGVPVWREATTTLFCASDAKAY	60
31	MRVMGIRKNYQHLWREGILLLGILMICSAADNLWVTVYYGVPVWREATTTLFCASDAKAY	60
32	MRVMGIRKNYQHLWREGILLLGILMICSAADNLWVTVYYGVPVWREATTTLFCASDAKAY	60
33	MRVMGIRKNYQHLWREGILLLGILMICSAADNLWVTVYYGVPVWREATTTLFCASDAKAY	60
34	MRVMGIRKNYQHLWREGILLLGILMICSAADNLWVTVYYGVPVWREATTTLFCASDAKAY	60
35	MRVMGIRKNYQHLWREGILLLGILMICSAADNLWVTVYYGVPVWREATTTLFCASDAKAY	60
36	MRVMGIRKNYQHLWREGILLLGILMICSAADNLWVTVYYGVPVWREATTTLFCASDAKAY	60
37	MRVMGIRKNYQHLWREGILLLGILMICSAADNLWVTVYYGVPVWREATTTLFCASDAKAY	60
38	MRVMGIRKNYQHLWREGILLLGILMICSAADNLWVTVYYGVPVWREATTTLFCASDAKAY	60
39	MRVMGIRKNYQHLWREGILLLGILMICSAAGNLWVTVYYGVPVWREATTTLFCASDAKAY	60
40	MRVMGIRKNYQHLWREGILLLGILMICSAADNLWVTVYYGVPVWREATTTLFCASDAKAY	60
41	MRVMGIRKNYQHLWREGILLLGILMICSAADNLWVTVYYGVPVWREATTTLFCASDAKAY	60
42	MRVMGIRKNYQHLWREGILLLGILMICSAADNLWVTVYYGVPVWREATTTLFCASDAKAY	60
43	MRVMGIRKNYQHLWREGILLLGILMICSAADNLWVTVYYGVPVWREATTTLFCASDAKAY	60
44	MRVMGIRKNYQHLWREGILLLGILMICSAADNLWVTVYYGVPVWREATTTLFCASDAKAY	60
45	MRVMGIRKNYQHLWREGILLLGILMICSAADNLWVTVYYGVPVWREATTTLFCASDAKAY	60
46	MRVMGIRKNYQHLWREGILLLGILMICSAADNLWVTVYYGVPVWREATTTLFCASDAKAY	60
47	MRVMGIRKNYQHLWREGILLLGILMICSAADNLWVTVYYGVPVWREATTTLFCASDAKAY	60
48	MRVMGIRKNYQHLWREGILLLGILMICSAADNLWVTVYYGVPVWREATTTLFCASDAKAY	60
49	MRVMGIRKNYQHLWREGILLLGILMICSAADNLWVTVYYGVPVWREATTTLFCASDAKAY	60
50	MRVMGIRKNYQHLWREGILLLGILMICSAADNLWVTVYYGVPVWREATTTLFCASDAKAY	60
51	MRVMGIRKNYQHLWREGILLLGILMICSAADNLWVTVYYGVPVWREATTTLFCASDAKAY	60
52	MRVMGIRKNYQHLWREGILLLGILMICSAADNLWVTVYYGVPVWREATTTLFCASDAKAY	60

53	MRVMGIRKNYQHLWREGILLLGILMICSAADNLWVTVYYGVPVWREATTTLFCASDAKAY	60
54	MRVMGIRKNYQHLWREGILLLGILMICSAADNLWVTVYYGVPVWREATTTLFCASDAKAY	60
55	MRVMGIRKNYQHLWREGILLLGILMICSAADNLWVTVYYGVPVWREATTTLFCASDAKAY	60
56	MRVMGIRKNYQHLWREGILLLGILMICSAADNLWVTVYYGVPVWREATTTLFCASDAKAY	60
57	MRVMGIRKNYQHLWREGILLLGILMICSAADNLWVTVYYGVPVWREATTTLFCASDAKAY	60
58	MRVMGIRKNYQHLWREGILLLGILMICSAADNLWVTVYYGVPVWREATTTLFCASDAKAY	60
59	MRVMGIRKNYQHLWREGILLLGILMICSAADNLWVTVYYGVPVWREATTTLFCASDAKAY	60
60	MRVMGIRKNYQHLWREGILLLGILMICSAADNLWVTVYYGVPVWREATTTLFCASDAKAY	60
61	MRVMGIRKNYQHLWREGILLLGILMICSAADNLWVTVYYGVPVWREATTTLFCASDAKAY	60
62	MRVMGIRKNYQHLWREGILLLGILMICSAADNLWVTVYYGVPVWREATTTLFCASDAKAY	60
63	MRVMGIRKNYQHLWREGILLLGILMICSAADNLWVTVYYGVPVWREATTTLFCASDAKAY	60
64	MRVMGIRKNYQHLWREGILLLGILMICSAADNLWVTVYYGVPVWREATTTLFCASDAKAY	60
65	MRVMGIRKNYQHLWREGILLLGILMICSAADNLWVTVYYGVPVWREATTTLFCASDAKAY	60
66	MRVMGIRKNYQHLWREGILLLGILMICSAADNLWVTVYYGVPVWREATTTLFCASDAKAY	60
67	MRVMGIRKNYQHLWREGILLLGILMICSAADNLWVTVYYGVPVWREATTTLFCASDAKAY	60
68	MRVMGIRKNYQHLWREGILLLGILMICSAADNLWVTVYYGVPVWREATTTLFCASDAKAY	60

DTEAHNVWATHACVPTDPNPQEVELKNVTENFNMWENNMVEQMHEDIISLWDQSLKPCVK

1	DTEAHNVWATHACVPTDPNPQEVELKNVTENFNMWENNMVEQMHEDIISLWDQSLKPCVK	120
2	DTEAHNVWATHACVPTDPNPQEVELKNVTENFNMWENNMVEQMHEDIISLWDQSLKPCVK	120
3	DTEAHNVWATHACVPTDPNPQEVELKNVTENFNMWENNMVEQMHEDIISLWDQSLKPCVK	120
4	DTEAHNVWATHACVPTDPNPQEVELKNVTENFNMWENNMVEQMHEDIISLWDQSLKPCVK	120
5	DTEAHNVWATHACVPTDPNPQEVELKNVTENFNMWENNMVEQMHEDIISLWDQSLKPCVK	120
6	DTEAHNVWATHACVPTDPNPQEVELKNVTENFNMWENNMVEQMHEDIISLWDQSLKPCVK	120
7	DTEAHNVWATHACVPTDPNPQEVELKNVTENFNMWENNMVEQMHEDIISLWDQSLKPCVK	120
8	DTEAHNVWATHACVPTDPNPQEVELKNVTENFNMWENNMVEQMHEDIISLWDQSLKPCVK	120
9	DTEAHNVWATHACVPTDPNPQEVKLKNVTENFNMWENNMVEQMHEDIISLWDQSLKPCVK	120
10	DTEAHNVWATHACVPTDPNPQEVELKNVTENFNMWENNMVEQMHEDIISLWDQSLKPCVK	120
11	DTEAHNVWATHACVPTDPNPQEVELKNVTENFNMWENNMVEQMHEDIISLWDQSLKPCVK	120
12	DTEAHNVWATHACVPTDPNPQEVELKNVTENFNMWENNMVEQMHEDIISLWDQSLKPCVK	120
13	DTEAHNVWATHACVPTDPNPQEVELKNVTENFNMWENNMVEQMHEDIISLWDQSLKPCVK	120
14	DTEAHNVWATHACVPTDPNPQEVELKNVTENFNMWENNMVEQMHEDIISLWDQSLKPCVK	120
15	DTEAHNVWATHACVPTDPNPQEVELKNVTENFNMWENNMVEQMHEDIISLWDQSLKPCVK	120
16	DTEAHNVWATHACVPTDPNPQEVELKNVTENFNMWENNMVEQMHEDIISLWDQSLKPCVK	120
17	DTEAHNVWATHACVPTDPNPQEVELKNVTENFNMWENNMVEQMHEDIISLWDQSLKPCVK	120
18	DTEAHNVWATHACVPTDPNPQEVELKNVTENFNMWENNMVEQMHEDIISLWDQSLKPCVK	120
19	DTEAHNVWATHACVPTDPNPQEVELKNVTENFNMWENNMVEQMHEDIISLWDQSLKPCVK	120
20	DTEAHNVWATHACVPTDPNPQEVELKNVTENFNMWENNMVEQMHEDIISLWDQSLKPCVK	120
21	DTEAHNVWATHACVPTDPNPQEVELKNVTENFNMWENNMVEQMHEDIISLWDQSLKPCVK	120
22	DTEA---------------PQEVELKNVTENFNMWENNMVEQMHEDIISLWDQSLKPCVK	105
23	DTEAHNVWATHACVPTDPNPQEVELKNVTENFNMWENNMVEQMHEDIISLWDQSLKPCVK	120
24	DTEAHNVWATHACVPTDPNPQEVELKNVTENFNMWENNMVEQMHEDIISLWDQSLKPCVK	120
25	DTEAHNVWATHACVPTDPNPQEVELKNVTENFNMWENNMVEQMHEDIISLWDQSLKPCVK	120
26	DTEAHNVWATHACVPTDPNPQEVELKNVTENFNMWENNMVEQMHEDIISLWDQSLKPCVK	120
27	DTEAHNVWATHACVPTDPNPQEVELKNVTENFNMWENNMVEQMHEDIISLWDQSLKPCVK	120
28	DTEAHNVWATHACVPTDPNPQEVELKNVTENFNMWENNMVEQMHEDIISLWDQSLKPCVK	120
29	DTEAHNVWATHACVPTDPNPQEVELKNVTENFNMWENNMVEQMHEDITSLWDQSLKPCVK	120
30	DTEAHNVWATHACVPTDPNPQEVELKNVTENFNMWENNMVEQMHEDIISVWDQSLKPCVK	120
31	DTEAHNVWATHACVPTDPNPQEVELKNVTENFNMWENNMVEQMHEDIISLWDQSLKPCVK	120
32	DTEAHNVWATHACVPTDPNPQEVELKNVTENFNMWENNMVEQMHEDIISLWDQSLKPCVK	120
33	DTEAHNVWATHACVPTDPNPQEVELKNVTENFNMWENNMVEQMHEDIISLWDQSLKPCVK	120
34	DTEAHNVWATHACVPTDPNPQEVELKNVTENFNMWENNMVEQMHEDIISLWDQSLKPCVK	120

```
35  DTEAHNVWATHACVPTDPNPQEVELKNVTENFNMWENNMVEQMHEDIISLWDQSLKPCVK  120
36  DTEAHNVWATHACVPTDPNPQEVELKNVTENFNMWENNMVEQMHEDIISLWDQSLKPCVK  120
37  DTEAHNVWATHACVPTDPNPQEVELKNVTENFNMWENNMVEQMHEDIISLWDQSLKPCVK  120
38  DTEAHNVWATHACVPTDPNPQEVELKNVTENFNMWENNMVEQMHEDIISLWDQSLKPCVK  120
39  DTEAHNVWATHACVPTDPNPQEVELKNVTENFNMWENNMVEQMHEDIISLWDQSLKPCVK  120
40  DTEAHNVWATHACVPTDPNPQEVELKNVTENFNMWENNMVEQMHEDIISLWDQSLKPCVK  120
41  DTEAHNVWATHACVPTDPNPQEVELKNVTENFNMWENNMAEQMHEDIISLWDQSLKPCVK  120
42  DTEAHNVWATHACVPTDPNPQEVELKNVTENFNMWENNMVEQMHEDIISLWDQSLKPCVK  120
43  DTEAHNVWATHACVPTDPNPQEVELKNVTENFNMWENNMVEQMHEDIISLWDQSLKPCVK  120
44  DTEAHNVWATHACVPTDPNPQEVGLKNVTENFNMWENNMVEQMHEDIISLWDQSLKPCVK  120
45  DTEAHNVWATHACVPTDPNPQEVELKNVTENFNMWENNMVEQMHEDIISLWDQSLKPCVK  120
46  DTEAHNVWATHACVPTDPNPQEVELKNVTENFNMWENNMVEQMHEDIISLWDQSLKPCVK  120
47  DTEAHNVWATHACVPTDPNPQEVELKNVTENFNMWENNMVEQMHEDIISLWDQSLKPCVK  120
48  DTEAHNVWATHACVPTDPNPQEVELKNVTENFNMWENNMVEQMHEDIISLWDQSLKPCVK  120
49  DTEAHNVWATHACVPTDPNPQEVELKNVTENFNMWENNMVEQMHEDIISLWDQSLKPCVK  120
50  DTEAHNVWATHACVPTDPNPQEVELKNVTENFNMWENNMVEQMHEDIISLWDQSLKPCVK  120
51  DTEAHNVWATHACVPTDPNPQEVELKNVTENFNMWENNMVEQMHEDIISLWDQSLKPCVK  120
52  DTEAHNVWATHACVPTDPNPQEVELKNVTENFNMWENNMVEQMHEDIISLWDQSLKPCVK  120
53  DTEAHNVWATHACVPTDPNPQEVELKNVTENFNMWENNMVEQMHEDIISLWDQSLKPCVK  120
54  DTEAHNVWATHACVPTDPNPQEVELKNVTENFNMWENNMVEQMHEDIISLWDQSLKPCVK  120
55  DTEAHNVWATHACVPTDPNPQEVELKNVTENFNMWENNMVEQMHEDIISLWDQSLKPCVK  120
56  DTEAHNVWATHACVPTDPNPQEVELKNVTENFNMWENNMVEQMHEDIISLWDQSLKPCVK  120
57  DTEAHNVWATHACVPTDPNPQEVELKNVTENFNMWENNMVEQMHEDIISLWDQSLKPCVK  120
58  DTEAHNVWATHACVPTDPNPQEVELKNVTENFNMWENNMVEQMHEDIISLWDQSLKPCVK  120
59  DTEAHNVWATHACVPTDPNPQEVELKNVTENFNMWENNMVEQMHEDIISLWDQSLKPCVK  120
60  DTEAHNVWATHACVPTDPNPQEVELKNVTENFNMWENNMVEQMHEDIISLWDQSLKPCVK  120
61  DTEAHNVWATHACVPTDPNPQEVELKNVTENFNMWENNMVEQMHEDIISLWDQSLKPCVK  120
62  DTEAHNVWATHACVPTDPNPQEVELKNVTENFNMWENNMVEQMHEDIISLWDQSLKPCVK  120
63  DTEAHNVWATHACVPTDPNPQEVELKNVTENFNMWENNMVEQMHEDIISLWDQSLKPCVK  120
64  DTEAHNVWATHACVPTDPNPQEVELKNVTENFNMWENNMVEQMHEDIISLWDQSLKPCVK  120
65  DTEAHNVWATHACVPTDPNPQEVELKNVTENFNMWENNMVEQMHEDIISLWDQSLKPCVK  120
66  DTEAHNVWATHACVPTDPNPQEVELKNVTENFNMWENNMVEQMHEDIISLWDQSLKPCVK  120
67  DTEAHNVWATHACVPTDPNPQEVELKNVTENFNMWENNMVEQMHEDIISLWDQSLKPCVK  120
68  DTEAHNVWATHACVPTDPNPQEVELKNVTENFNMWENNMVEQMHEDIISLWDQSLKPCVK  120
```

```
    LTPLCVTLNCTDLGNVTNTT---NSNGKMMEKGEVKNCSFKITTDIKDRTRKEYALFYKL

1   LTPLCVTLNCTDLGNVTNTT---NSNGEMMEKGEVKNCSFKITTDIKDRTRKEYALFYKL  177
2   LTPLCVTLNCTDLGKVTNTT---NSNGKMMEKGDV-NCSFKITTDIKDRTRKEYALFYKL  176
3   LTPLCVTLNCTDLGNVTNTT---NSNGKMMEKGEVKNCSFKITTDIKDRTRKEYALFYKL  177
4   LTPLCVTLNCTDLGNVTNTT---NSNGKMMEKGEVKNCSFKITTDIKDRTRKEYALFYKL  177
5   LTPLCVTLNCTDLGNVTNTT---NSNGKMMEKGEVKNCSFKITTDIKDRTRKEYALFYKL  177
6   LTPLCVTLNCTDLGNVTNTT---NSNGKMMEKGEVKNCSFKITTDIKDRTRKEYALFYKL  177
7   LTPLCVTLNCTDLGNVTNTT---NSNGKMMEKGEVKNCSFKITTDIKDRTRKEYALFYKL  177
8   LTPLCVTLNCTDLGNVTNTT---NSNGGMMEKGEVKNCSFKITTDIKDRTRKEYALFYKL  177
9   LTPLCVTLNCTDLGNVTNTT---NSNGGMMEKGEVKNCSFKITTDIKDRTRKEYALFYKL  177
10  LTPLCVTLNCTDLGNVTNTT---NSNGKMMEKGEVKNCSFKITTDIKDRTRKEYALFYKL  177
11  LTPLCVTLNCTDLGNVTNTT---NSNGKMMEKGEVKNCSFKITTDIKDRTRKEYALFYKL  177
12  LTPLCVTLNCTDLGNVTNTT---NSNGKMMEKGEVKNCSFKITTDIKDRTRKEYALFYKL  177
13  LTPLCVTLNCTDLGNVTNTT---NSNGKMMEKGEVKNCSFKITTDIKDRTRKEYALFYKL  177
14  LTPLCVTLNCTDLGNVTNTT-----NSKMMEKGEVKNCSFKITTDIKDRTRKEYALFYKL  175
15  LTPLCVTLNCTDLGNVTNTT---NSNGKMMEKGEVKNCSFKITTDIKDRTRKEYALFY--  175
16  LTPLCVTLNCTDLGNVTNTT---NSNGKMMEKGEVKNCSFKITTDIKDRTRKEYALFYKL  177
```

17 LTPLCVTLNCTDLGNVTNTT---NSNGKMMEKGEVKNCSFKITTDIKDRTRKEYALFYKL 177
18 LTPLCVTLNCTDLGNVTNTT---NSNGKMMEKGEVKNCSFKITTDIKDRTRKEYALFYKL 177
19 LTPLCVTLNCTDLGNVTNTT---NSNGKMMEKGEVKNCSFKITTDIKDRTRKEYALFYKL 177
20 LTPLCVTLNCTDLGNVTNTT-----NSKMMEKGEVKNCSFKITTDIKDRTRKEYALFYKL 175
21 LTPLCVTLNCTDLGNVTNTT---NSNGGMMEKGEVKNCSFKITTDIKDRTRKEYALFYKL 177
22 LTPLCVTLNCTDLGNVTNTT-----NSKMMEKGEVKNCSFKITTDIKDRTRKEYALFYKL 160
23 LTPLCVTLNCTDLGNVTNTT---NSNGKMMEKGEVKNCSFKITTDIKDRTRKEYALFYKL 177
24 LTPLCVTLNCTDLGKVTNTT---NSNGKMMEKGEVKNCSFKITTDIKDRTRKEYALFYKL 177
25 LTPLCVTLNCTDLGNVTNTT---NSNGKMMEKGEVKNCSFKITTDIKDRTRKEYALFYKL 177
26 LTPLCVTLNCTDLGNVTNTT---NSNGKMMEKGEVKNCSFKITTDIKDRTRKEYALFYKL 177
27 LTPLCVTLNCTDLGNVTNTT---NSNGKMMEKGEVKNCSFKITTDIKDRTRKEYALFYKL 177
28 LTPLCVTLNCTDLGNVTNTT---NSNGKMMEKGEVKNCSFKITTDIKDRTRKEYALFYKL 177
29 LTPLCVTLNCTDLGNVTNTT---NSNGKMMEKGEVKNCSFKITTDIKDRTRKEYALFYKL 177
30 LTPLCVTLNCTDLGNVTNTT---NSNGKMMEKGEVKNCSFKITTDIKDRTRKEYALFYKL 177
31 LTPLCVTLNCTDLGNVTNTT---NSNGKMMEKGEVKNCSFKITTDIKDRTRKEYALFYKL 177
32 LTPLCVTLNCTDLGNVTNTT---NSNGKMMEKGEVKNCSFKITTDIKDRTRKEYALFYKL 177
33 LTPLCVTLNCTDLGNVTNTT---NSNGKMMEKGEVKNCSFKITTDIKDRTRKEYALFYKL 177
34 LTPLCVTLNCTDLGNVTNTT---NSNGKMMEKGEVKNCSFKITTDIKDRTRKEYALFYKL 177
35 LTPLCVTLNCTDLGNVTNTT---NSNGKMMEKGEVKNCSFKITTDIKDRTRKEYALFYKL 177
36 LTPLCVTLNCTDLGKVTNTT---NSNGKMMEKGEVKNCSFKITTDIKDRTRKEYALFYKL 177
37 LTPLCVTLNCTDLGKVTNTT---NSNGKMMEKGEVKNCSFKITTDIKDRTRKEYALFYKL 177
38 LTPLCVTLNCTDLGNVTNTT-----NSKMMEKGEVKNCSFKITTDIKDRTRKEYALFYKL 175
39 LTPLCVTLNCTDLGNVTNTT---NSNREMMEKGEVKNCSFKITTDIKDRTRKEYALFYKL 177
40 LTPLCVTLNCTDLGNVTNTT---NSNGKMMEKGEVKNCSFKITTDIKDRTRKEYALFYKL 177
41 LTPLCVTLNCTDLGNVTNTT---NSNGKMMEKGEVKNCSFKITTDIKDRTRKEYALFYKL 177
42 LTPLCVTLNCTDLGNVTNTT---NSNGKMMEKGEVKNCSFKITTDIKDRTRKEYALFYKL 177
43 LTPLCVTLNCTDLGNVTNTT---NSNGKMMEKGEVKNCSFKITTDIKDRTRKEYALFYKL 177
44 LTPLCVTLNCTDLGNVTNTT---NSNGKMMEKGEVKNCSFKITTDIKDRTRKEYALFYKL 177
45 LTPLCVTLNCTDLGNVTNTT---NSNGKMMEKGEVKNCSFKITTDIKDRTRKEYALFYKL 177
46 LTPLCVTLNCTDLGNVTNTT---NSNGKMMEKGEVKNCSFKITTDIKDRTRKEYALFYKL 177
47 LTPLCVTLNCTDLGNVTNTT---NSNGGMMEKGEVKNCSFKITTDIKDRTRKEYALFYKL 177
48 LTPLCVTLNCTDLGNVTNTT---NSNGKMMEKGEVKNCSFKITTDIKDRTRKEYALFYKL 177
49 LTPLCVTLNCTDLGNVTNTT---NSNGKMMEKGEVKNCSFKITTDIKDRTRKEYALFYKL 177
50 LTPLCVTLNCTDLGNVTNTT---NSNGGMMEKGEVKNCSFKITTDIKDRTRKEYALFYKL 177
51 LTPLCVTLNCTDLGNVTNTT---NSNGKMMEKGEVKNCSFKITTDIKDRTRKEYALFYKL 177
52 LTPLCVTLNCTDLGNVTNTT---NSNGKMMEKGEVKNCSFKITTDIKDRTRKEYALFYKL 177
53 LTPLCVTLNCTDLGNVTNTT---NSNGGMMEKGEVKNCSFKITTDIKDRTRKEYALFYKL 177
54 LTPLCVTLNCTDLGNVTNTT---NSNGGRMEKGEVKNCSFKITTDIKDRTRKEYALFYKL 177
55 LTPLCVTLNCTDLGNVTNTT---NSNGKMMEKGEVKNCSFKITTDIKDRTRKEYALFYKL 177
56 LTPLCVTLNCTDLGNVTNTT---NSNGKMMEKGEVKNCSFKITTDIKDRTRKEYALFYKL 177
57 LTPLCVTLNCTDLGNVTNTT---NSNGGMMEKGEVKNCSFKITTDIKDRTRKEYALFYKL 177
58 LTPLCVTLNCTDLGNVTNTT---NSNGKMMEKGEVKNCSFKITTDIKDRTRKEYALFYKL 177
59 LTPLCVTLNCTDLGNVTNTT-----NSNGMEKGEVKNCSFKITTDIKDRTRKEYALFYKL 175
60 LTPLCVTLNCTDLGNVTNTT---NSNGKMMEKGEVKNCSFKITTDIKDRTRKEYALFYKL 177
61 LTPLCVTLNCTDLGNVTNTT---NSNGGMMEKGEVKNCSFKITTDIKDRTRKEYALFYKL 177
62 LTPLCVTLNCTDLGNVTNTT---NSNGKMMEKGEVKNCSFKITTDIKDRTRKEYALFYKL 177
63 LTPLCVTLNCTDLGNVTNTT---NSNGKMMEKGEVKNCSFKITTDIKDRTRKEYALFYKL 177
64 LTPLCVTLNCTDLGNVTNTT---NSNGKMMEKGEVKNCSFKITTDIKDRTRKEYALFYKL 177
65 LTPLCVTLNCTDLGNVTNTT---NSNGKMMEKGEVKNCSFKITTDIKDRTRKEYALFYKL 177
66 LTPLCVTLNCTDLGNVTNTT---NSNGKMMEKGEVKNCSFKITTDIKDRTRKEYALFYKL 177
67 LTPLCVTLNCTDLGNVTNTT-----NSNGEMMGEVKNCSFKITTDIKDRTRKEYALFYKL 175
68 LTPLCVTLNCTDLGNVTNTT---NSNGKMMEKGEVKNCSFKITTDIKDRTRKEYALFYKL 177

	DVVPIND-------TRYRLVSCNTSVITQACPKVSFEPIPIHYCAPAGFAILKCNDKQFI	
1	DVVPIND-------TRYRLVSCNTSVITQACPKVSFEPIPIHYCAPAGFAILKCNDKQFI	230
2	DVVPIND-------TRYRLVSCNTSVITQACPKVSFEPIPIHYCAPAGFAILKCNDKQFI	229
3	DVVPIND-------TRYRLVSCNTSVITQACPKVSFEPIPIHYCAPAGFAILKCNDKQFI	230
4	DVVPIND-------TRYRLVSCNTSVITQACPKVSFEPIPIHYCAPAGFAILKCNDKQFI	230
5	DVVPIND-------TRYRLVSCNTSVITQACPKVSFEPIPIHYCAPAGFAILKCNDKQFI	230
6	DVVPIND-------TRYRLVSCNTSVITQACPKVSFEPIPIHYCAPAGFAILKCNDKQFI	230
7	DVVPIND-------TRYRLVSCNTSVITQACPKVSFEPIPIHYCAPAGFAILKCNDKQFI	230
8	DVVPIND-------TRYRLVSCNTSVITQACPKVSFEPIPIHYCAPAGFAILKCNDKQFI	230
9	DVVPIND-------TRYRLVSCNTSVITQACPKVSFEPIPIHYCAPAGFAILKCNDKQFI	230
10	DVVPIND-------TRYRLVSCNTSVITQACPKVSFEPIPIHYCAPAGFAILKCNDKQFI	230
11	DVVPIND-------TRYRLVSCNTSVITQACPKVSFEPIPIHYCAPAGFAILKCNDKQFI	230
12	DVVPIND-------TRYRLVSCNTSVITQACPKVSFEPIPIHYCAPAGFAILKCNDKQFI	230
13	DVVPIND-------TRYRLVSCNTSVITQACPKVSFEPIPIHYCAPAGFAILKCNDKQFI	230
14	DVVPIND-------TRYRLVSCNTSVITQACPKVSFEPIPIHYCAPAGFAILKCNDKQFI	228
15	-------------------------------------PIPIHYCAPAGFAILKCNDKQFI	198
16	DVVPIND-------TRYRLVSCNTSVITQACPKVSFEPIPIHYCAPAGFAILKCNDKQFI	230
17	DVVPIND-------TRYRLVSCNTSVITQACPKVSFEPIPIHYCAPAGFAILKCNDKQFI	230
18	DVVPIND-------TRYRLVSCNTSVITQACPKVSFEPIPIHYCAPAGFAILKCNDKQFI	230
19	DVVPIND-------TRYRLVSCNTSVITQACPKVSFEPIPIHYCAPAGFAILKCNDKQFI	230
20	DVVPIND-------TRYRLVSCNTSVITQACPKVSFEPIPIHYCAPAGFAILKCNDKQFI	228
21	DVVPIND-------TRYRLVSCNTSVITQACPKVSFEPIPIHYCAPAGFAILKCNDKQFI	230
22	DVVPIND-------TRYRLVSCNTSVITQACPKVSFEPIPIHYCAPAGFAILKCNDKQFI	213
23	DVVPIND-------TRYRLVSCNTSVITQACPKVSFEPIPIHYCAPAGFAILKCNDKQFI	230
24	DVVPIND-------TRYRLVSCNTSVITQACPKVSFEPIPIHYCAPAGFAILKCNDKQFI	230
25	DVVPIND-------TRYRLVSCNTSVITQACPKVSFEPIPIHYCAPAGFAILKCNDKQFI	230
26	DVVPIND-------TRYRLVSCNTSVITQACPKVSFEPIPIHYCAPAGFAILKCNDKQFI	230
27	DVVPIND-------TRYRLVSCNTSVITQACPKVSFEPIPIHYCAPAGFAILKCNDKQFI	230
28	DVVPIND-------TRYRLVSCNTSVITQACPKVSFEPIPIHYCAPAGFAILKCNDKQFI	230
29	DVVPIND-------TRYRLVSCNTSVITQACPKVSFEPIPIHYCAPAGFAILKCNDKQFI	230
30	DVVPIND-------TRYRLVSCNTSVITQACPKVSFEPIPIHYCAPAGFAILKCNDKQFI	230
31	DVVPIND-------TRYRLVSCNTSVITQACPKVSFEPIPIHYCAPAGFAILKCNDKQFI	230
32	DVVPIND-------TRYRLVSCNTSVITQACPKVSFEPIPIHYCAPAGFAILKCNDKQFI	230
33	DVVPIND-------TRYRLVSCNTSVITQACPKVSFEPIPIHYCAPAGFAILKCNDKQFI	230
34	DVVPIND-------TRYRLVSCNTSVITQACPKVSFEPIPIHYCAPAGFAILKCNDKQFI	230
35	DVVPIND-------TRYRLVSCNTSVITQACPKVSFEPIPIHYCAPAGFAILKCNDKQFI	230
36	DVVPIND-------TRYRLVSCNTSVITQACPKVSFEPIPIHYCAPAGFAILKCNDKQFI	230
37	DVVPIND-------TRYRLVSCNTSVITQACPKVSFEPIPIHYCAPAGFAILKCNDKQFI	230
38	DVVPIND-------TRYRLVSCNTSVITQACPKVSFEPIPIHYCAPAGFAILKCNDKQFI	228
39	DVVPIND-------TRYRLVSCNTSVITQACPKVSFEPIPIHYCAPAGFAILKCNDKQFI	230
40	DVVPIND-------TRYRLVSCNTSVITQACPKVSFEPIPIHYCAPAGFAILKCNDKQFI	230
41	DVVPIND-------TRYRLVSCNTSVITQACPKVSFEPIPIHYCAPAGFAILKCNDKQFI	230
42	DVVPIND-------TRYRLVSCNTSVITQACPKVSFEPIPIHYCAPAGFAILKCNDKQFI	230
43	DVVPIND-------TRYRLVSCNTSVITQACPKVSFEPIPIHYCAPAGFAILKCNDKQFI	230
44	DVVPIND-------TRYRLVSCNTSVITQACPKVSFEPIPIHYCAPAGFAILKCNDKQFI	230
45	DVVPIND-------TRYRLVSCNTSVITQACPKVSFEPIPIHYCAPAGFAILKCNDKQFI	230
46	DVVPIND-------TRYRLVSCNTSVITQACPKVSFEPIPIHYCAPAGFAILKCNDKQFI	230
47	DVVPIND-------TRYRLVSCNTSVITQACPKVSFEPIPIHYCAPAGFAILKCNDKQFI	230
48	DVVPIND-------TRYRLVSCNTSVITQACPKVSFEPIPIHYCAPAGFAILKCNDKQFI	230
49	DVVPIND-------TRYRLVSCNTSVITQACPKVSFEPIPIHYCAPAGFAILKCNDKQFI	230
50	DVVPIDD-------TRYRLVSCNTSVITQACPKVSFEPIPIHYCAPAGFAILKCNDKQFI	230
51	DVVPIND-------TRYRLVSCNTSVITQACPKVSFEPIPIHYCAPAGFAILKCNDKQFI	230
52	DVVPIND-------TRYRLVSCNTSVITQACPKVSFEPIPIHYCAPAGFAILKCNDKQFI	230
53	DVVPIND-------TRYRLVSCNTSVITQACPKVSFEPIPIHYCAPAGFAILKCNDKQFI	230

54	DVVPIND-------TRYRLVSCNTSVITQACPKVSFEPIPIHYCAPAGFAILKCNDKQFI	230
55	DVVPIND-------TRYRLVSCNTSVITQACPKVSFEPIPIHYCAPAGFAILKCNDKQFI	230
56	DVVPIND-------TRYRLVSCNTSVITQACPKVSFEPIPIHYCAPAGFAILKCNDKQFI	230
57	DVVPIND-------TRYRLVSCNTSVITQACPKVSFEPIPIHYCAPAGFAILKCNDKQFI	230
58	DVVPIND-------TRYRLVSCNTSVITQACPKVSFEPIPIHYCAPAGFAILKCNDKQFI	230
59	DVVPIND-------TRYRLVSCNTSVITQACPKVSFEPIPIHYCAPAGFAILKCNDKQFI	228
60	DVVPIND-------TRYRLVSCNTSVITQACPKVSFEPIPIHYCAPAGFAILKCNDKQFI	230
61	DVVPIND-------TRYRLVSCNTSVITQACPKISFEPIPIHYCAPAGFAILKCNDKQFI	230
62	DVVPIND-------TRYRLVSCNTSVITQACPKVSFEPIPIHYCAPAGFAILKCNDKQFI	230
63	DVVPIND-------TRYRLVSCNTSVITQACPKVSFEPIPIHYCAPAGFAILKCNDKQFI	230
64	DVVPIND-------TRYRLVSCNTSVITQACPKVSFEPIPIHYCAPAGFAILKCNDKQFI	230
65	DVVPIND-------TRYRLVSCNTSVITQACPKVSFEPIPIHYCAPAGFAILKCNDKQFI	230
66	DVVPIND-------TRYRLVSCNTSVITQACPKVSFEPIPIHYCAPAGFAILKCNDKQFI	230
67	DVVPIND-------TRYRLVSCNTSVITQACPKVSFEPIPIHYCAPAGFAILKCNDKQFI	228
68	DVVPIND-------TRYRLVSCNTSVITQACPKVSFEPIPIHYCAPAGFAILKCNDKQFI	230

	**GTGPCTNVSTVQCTHGIRPVVSTQLLLNGSLAEEEVVIRSVNFSDNAKTIIVQLNKSVEI**	
1	GTGPCTNVSTVQCTHGIRPVVSTQLLLNGSLAEEEVVIRSVNFSDNAKTIIVQLNKSVEI	290
2	GTGPCTNVSTVQCTHGIRPVVSTQLLLNGSLAEEEVVIRSVNFSDNAKTIIVQLNKSVEI	289
3	GTGPCTNVSTVQCTHGIRPVVSTQLLLNGSLAEEEVVIRSVNFSDNAKTIIVQLNKSVKI	290
4	GTGPCTNVSTVQCTHGIRPVVSTQLLLNGSLAEEEVVIRSVNFSDNAKTIIVQLNKSVEI	290
5	GTGPCTNVSTVQCTHGIRPVVSTQLLLNGSLAEEEVVIRSVNFSDNAKTIIVQLNKSVKI	290
6	GTGPCTNVSTVQCTHGIRPVVSTQLLLNGSLAEEEVVIRSVNFSDNAKTIIVQLNKSVEI	290
7	GTGPCTNVSTVQCTHGIRPVVSTQLLLNGSLAEEEVVIRSVNFSDNAKTIIVQLNKSVEI	290
8	GTGPCTNVSTVQCTHGIRPVVSTQLLLNGSLAEEEVVIRSVNFSDNAKTIIVQLNKSVEI	290
9	GTGPCTNVSTVQCTHGIRPVVSTQLLLNGSLAEEEVVIRSVNFSDNAKTIIVQLNKSVEI	290
10	GTGPCTNVSTVQCTHGIRPVVSTQLLLNGSLAEEEVVIRSVNFSDNAKTIIVQLNKSVEI	290
11	GTGPCTNVSTVQCTHGIRPVVSTQLLLNGSLAEEEVVIRSVNFSDNAKTIIVQLNKSVEI	290
12	GTGPCTNVSTVQCTHGIRPVVSTQLLLNGSLAEEEVVIRSVNFSDNAKTIIVQLNKSVEI	290
13	GTGPCTNVSTVQCTHGIRPVVSTQLLLNGSLAEEEVVIRSVNFSDNAKTIIVQLNKSVEI	290
14	GTGPCTNVSTVQCTHGIRPVVSTQLLLNGSLAEEEVVIRSVNFSDNAKTIIVQLNKSVEI	288
15	GTGPCTNVSTVQCTHGIRPVVSTQLLLNGSLAEEEVVIRSVNFSDNAKTIIVQLNKSVKI	258
16	GTGPCTNVSTVQCTHGIRPVVSTQLLLNGSLAEEEVVIRSVNFSDNAKTIIVQLNKSVEI	290
17	GTGPCTNVSTVQCTHGIRPVVSTQLLLNGSLAEEEVVIRSVNFSDNAKTIIVQLNKSVKI	290
18	GTGPCTNVSTVQCTHGIRPVVSTQLLLNGSLAEEEVVIRSVNFSDNAKTIIVQLNKSVEI	290
19	GTGPCTNVSTVQCTHGIRPVVSTQLLLNGSLAEEEVVIRSVNFSDNAKTIIVQLNKSVEI	290
20	GTGPCTNVSTVQCTHGIRPVVSTQLLLNGSLAEEEVVIRSVNFSDNAKTIIVQLNKSVEI	288
21	GTGPCTNVSTVQCTHGIRPVVSTQLLLNGSLAEEEVVIRSVNFSDNAKTIIVQLNKSVEI	290
22	GTGPCTNVSTVQCTHGIRPVVSTQLLLNGSLAEEEVVIRSVNFSDNAKTIIVQLNKSVEI	273
23	GTGPCTNVSTVQCTHGIRPVVSTQLLLNGSLAEEEVVIRSVNFSDNAKTIIVQLNKSVEI	290
24	GTGPCTNVSTVQCTHGIRPVVSTQLLLNGSLAEEEVVIRSVNFSDNAKTIIV-LNKSVEI	289
25	GTGPCTNVSTVQCTHGIRPVVSTQLLLNGSLAEEEVVIRSVNFSDNAKTIIVQLNKSVEI	290
26	GTGPCTNVSTVQCTHGIRPVVSTQLLLNGSLAEEEVVIRSVNFSDNAKTIIVQLNKSVEI	290
27	GTGPCTNVSTVQCTHGIRPVVSTQLLLNGSLAEEEVVIRSVNFSDNAKTIIVQLNKSVKI	290
28	GTGPCTNVSTVQCTHGIRPVVSTQLLLNGSLAEEEVVIRSVNFSDNAKTIIVQLNKSVKI	290
29	GTGPCTNVSTVQCTHGIRPVVSTQLLLNGSLAEEEVVIRSVNFSDNAKTIIVQLNKSVKI	290
30	GTGPCTNVSTVQCTHGIRPVVSTQLLLNGSLAEEEVVIRSVNFSDNAKTIIVQLNKSVKI	290
31	GTGPCTNVSTVQCTHGIRPVVSTQLLLNGSLAEEEVVIRSVNFSDNAKTIIVQLNKSVEI	290
32	GTGPCTNVSTVQCTHGIRPVVSTQLLLNGSLAEEEVVIRSVNFSDNAKTIIVQLNKSVEI	290
33	GTGPCTNVSTVQCTHGIRPVVSTQLLLNGSLAEEEVVIRSVNFSDNAKTIIVQLNKSVEI	290
34	GTGPCTNVSTVQCTHGIRPVVSTQLLLNGSLAEEEVVIRSVNFSDNAKTIIVQLNKSVKI	290
35	GTGPCTNVSTVQCTHGIRPVVSTQLLLNGSLAEEEVVIRSVNFSDNAKTIRVQLNKSVEI	290

36	GTGPCTNVSTVQCTHGIRPVVSTQLLLNGSLAEEEVVIRSVNFSDNAKTIIVQLNKSVEI	290
37	GTGPCTNVSTVQCTHGIRPVVSTQLLLNGSLAEEEVVIRSVNFSDNAKTIIVQLNKSVEI	290
38	GTGPCTNVSTVQCTHGIRPVVSTQLLLNGSLAEEEVVIRSVNFSDNAKTIIVQLNKSVEI	288
39	GTGPCTNVSTVQCTHGIRPVVSTQLLLNGSLAEEEVVIRSVNFSDNAKTIIVQLNKSVEI	290
40	GTGPCTNVSTVQCTHGIRPVVSTQLLLNGSLAEEEVVIRSVNFSDNAKTIIVQLNKSVKI	290
41	GTGPCTNVSTVQCTHGIRPVVSTQLLLNGSLAEEEVVIRSVNFSDNAKTIIVQLNKSVKI	290
42	GTGPCTNVSTVQCTHGIRPVVSTQLLLNGSLAEEEVVIRSVNFSDNAKTIIVQLNKSVEI	290
43	GTGPCTNVSTVQCTHGIRPVVSTQLLLNGSLAEEEVVIRSVNFSDNAKTIIVQLNKSVEI	290
44	GTGPCTNVSTVQCTHGIRPVVSTQLLLNGSLAEEEVVIRSVNFSDNAKTIIVQLNKSVEI	290
45	GTGPCTNVSTVQCTHGIRPVVSTQLLLNGSLAEEEVVIRSVNFSDNAKTIIVQLNKSVEI	290
46	GTGPCTNVSTVQCTHGIRPVVSTQLLLNGSLAEEEVVIRSVNFSDNAKTIIVQLNKSVKI	290
47	GTGPCTNVSTVQCTHGIRPVVSTQLLLNGSLAEEEVVIRSVNFSDNAKTIIVQLNKSVEI	290
48	GTGPCTNVSTVQCTHGIRPVVSTQLLLNGSLAEEEVVIRSVNFSDNAKTIIVQLNKSVEI	290
49	GTGPCTNVSTVQCTHGIRPVVSTQLLLNGSLAEEEVVIRSVNFSDNAKTIIVQLNKSVEI	290
50	GTGPCTNVSTVQCTHGIRPVVSTQLLLNGSLAEEEVVIRSVNFSDNAKTIIVQLNKSVEI	290
51	GTGPCTNVSTVQCTHGIRPVVSTQLLLNGSLAEEEVVIRSVNFSDNAKTIIVQLNKSVEI	290
52	GTGPCTNVSTVQCTHGIRPVVSTQLLLNGSLAEEEVVIRSVNFSDNAKTIIVQLNKSVEI	290
53	GTGPCTNVSTVQCTHGIRPVVSTQLLLNGSLAEEEVVIRSVNFSDNAKTIIVQLNKSVEI	290
54	GTGPCTNVSTVQCTHGIRPVVSTQLLLNGSLAEEEVVIRSVNFSDNAKTIIVQLNKSVEI	290
55	GTGPCTNVSTVQCTHGIRPVVSTQLLLNGSLAEEEVVIRSVNFSDNAKTIIVQLNKSVEI	290
56	GTGPCTNVSTVQCTHGIRPVVSTQLLLNGSLAEGEVVIRSVNFSDNAKTIIVQLNKSVEI	290
57	GTGPCTNVSTVQCTHGIRPVVSTQLLLNGSLAEEEVVIRSVNFSDNAKTIIVQLNKSVEI	290
58	GTGPCTNVSTVQCTHGIRPVVSTQLLLNGSLAEEEVVIRSVNFSDNAKTIIVQLNKSVEI	290
59	GTGPCTNVSTVQCTHGIRPVVSTQLLLNGSLAEEEVVIRSVNFSDNAKTIIVQLNKSVEI	288
60	GTGPCTNVSTVQCTHGIRPVVSTQLLLNGSLAKEEVVIRSVNFSDNAKTIIVQLNKSVEI	290
61	GTGPCTNVSTVQCTHGIRPVVSTQLLLNGSLAEEEVVIRSVNFSDNAKTIIVQLNKSVEI	290
62	GTGPCTNVSTVQCTHGIRPVVSTQLLLNGSLAEEEVVIRSVNFSDNAKTIIVQLNKSVEI	290
63	GTGPCTNVSTVQCTHGIRPVVSTQLLLNGSLAEEEVVIRSVNFSDNAKTIIVQLNKSVEI	290
64	GTGPCTNVSTVQCTHGIRPVVSTQLLLNGSLAEEEVVIRSVNFSDNAKTIIVQLNKSVEI	290
65	GTGPCTNVSTVQCTHGIRPVVSTQLLLNGSLAEEEVVIRSVNFSDNAKTIIVQLNKSVEI	290
66	GTGPCTNVSTVQCTHGIRPVVSTQLLLNGSLAEEEVVIRSVNFSDNAKTIIVQLNKSVEI	290
67	GTGPCTNVSTVQCTHGIRPVVSTQLLLNGSLAEEEVVIRSVNFSDNAKTIIVQLNKSVEI	288
68	GTGPCTNVSTVQCTHGIRPVVSTQLLLNGSLAEEEVVIRSVNFSDNAKTIIVQLNKSVEI	290

	**TCTRPNNNTRKSIPMGPGKAFYARGDITGDIRKAYCEINGTEWHSTLKLVVEKLREQY-N**	
1	TCTRPNNNTRKSIPMGPGKAFYARGDITGDIRKAYCEINGTEWHSTLKLVVEKLREQY-N	349
2	TCTRPNNNTRKSIPMGPGKAFYARGDITGDIRKAYCEINGTEWHSTLKLVVEKLREQY-N	348
3	TCTRPNNNTRKSIPMGPGKAFYARGDITGDIRKAYCEINGTEWHSTLKLVVEKLREQY-N	349
4	TCTRPNNNTRKSIPMGPGKAFYARGDITGDIRKAYCEINGTEWHSTLKLVVEKLREQY-N	349
5	TCTRPNNNTRKSIPMGPGKAFYARGDITGDIRKAYCEINGTEWHSTLKLVVEKLREQY-N	349
6	TCTRPNNNTRKSIPMGPGKAFYARGDITGDIRKAYCEINGTEWHSTLKLVVEKLREQY-N	349
7	TCTRPNNNTRKSIPIGPGKAFYARGDITGDIRKTYCEINGTEWHSTLKLVVEKLREQY-N	349
8	TCTRPNNNTRKSIPMGPGKAFYARGDITGDIRKAYCEINGTEWHSTLKLVVEKLREQY-N	349
9	TCTRPNNNTRKSIPMGPGKAFYARGDITGDIRKAYCEINGTEWHSTLKLVVEKLREQY-N	349
10	TCTRPNNNTRKSIPMGPGKAFYARGDITGDIRKAYCEINGTEWHSTLKLVVEKLREQY-N	349
11	TCTRPNNNTRKSIPMGPGKAFYARGDITGDIRKAYCEINGTEWHSTLKLVVEKLREQY-N	349
12	TCTRPNNNTRKSIPMGPGKAFYARGDITGDIRRAYCEINGTEWHSTLKLVVEKLREQY-N	349
13	TCTRPNNNTRKSIPMGPGKAFYARGDITGDIRKAYCEINGTEWHSTLKLVVEKLREQY-N	349
14	TCTRPNNNTRKSIPMGPGKAFYARGDITGDIRKAYCEINGTEWHSTLKLVVEKLREQY-N	347
15	TCTRPNNNTRKSIPMGPGKAFYARGDITGDIRKAYCEINGTEWHSTLKLVVEKLREQY-N	317
16	TCTRPNNNTRKSIPMGPGKAFYARGDITGDIRKAYCEINGTEWHSTLKLVVEKLREQY-N	349
17	TCTRPNNNTRKSIPMGPGKAFYARGDITGDIRKAYCEINGTEWHSTLKLVVEKLREQY-N	349

18	TCTRPNNNTRKSIPMGPGKAFYARGDITGDIRKAYCEINGTEWHSTLKLVVEKLREQY-N	349
19	TCTRPNNNTRKSIPMGPGKAFYARGDITGDIRKAYCEINGTEWHSTLKLVVEKLREQY-N	349
20	TCTRPNNNTRKSIPMGPGKAFYARGDITGDIRKAYCEINGTEWHSTLKLVVEKLREQY-N	347
21	TCTRPNNNTRKSIPMGPGKAFYARGDITGDIRKAYCEINGTEWHSTLKLVVEKLREQY-N	349
22	TCTRPNNNTRKSIPMGPGKAFYARGDITGDIRKAYCEINGTEWHSTLKLVVEKLREQY-N	332
23	TCTRPNNNTRKSIPMGPGKAFYARGDITGDIRKAYCEINGTEWHSTLKLVVEKLREQY-N	349
24	TCTRPNNNTRKSIPMGPGKAFYARGDITGDIRKAYCEINGTEWHSTLKLVVEKLREQY-N	348
25	TCTRPNNNTRKSIPMGPGKAFYARGDITGDIRKAYCEINGTEWHSTLKLVVEKLREQY-N	349
26	TCTRPNNNTRKSIPMGPGKAFYARGDITGDIRKAYCEINGTEWHSTLKLVVEKLREQY-N	349
27	TCTRPNNNTRKSIPMGPGKAFYARGDITGDIRKAYCEINGTEWHSTLKLVVEKLREQY-N	349
28	TCTRPNNNTRKSIPMGPGKAFYARGDITGDIRKAYCEINGTEWHSTLKLVVEKLREQY-N	349
29	TCTRPNNNTRKSIPMGPGKAFYARGDITGDIRKAYCEINGTEWHSTLKLVVEKLREQY-N	349
30	TCTRPNNNTRKSIPMGPGKAFYARGDITGDIRKAYCEINGTEWHSTLKLVVEKLREQY-N	349
31	TCTRPNNNTRKSIPMGPGKAFYARGDITGDIRKAYCEINGTEWHSTLKLVVEKLREQY-N	349
32	TCTRPNNNTRKSIPMGPGKAFYARGDITGDIRKAYCEINGTEWHSTLKLVVEKLREQY-N	349
33	TCTRPNNNTRKSIPMGPGKAFYARGDITGDIRKAYCEINGTEWHSTLKLVVEKLREQY-N	349
34	TCTRPNNNTRKSIPMGPGKAFYARGDITGDIRKAYCEINGTEWHSTLKLVVEKLREQY-N	349
35	TCTRPNNNTRKSIPMGPGKAFYARGDITGDIRKAYCEINGTEWHSTLKLVVEKLREQY-N	349
36	TCTRPNNNTRKSIPMGPGKAFYARGDITGDIRKAYCEINGTEWHSTLELVVEKLREQY-N	349
37	TCTRPNNNTRKSIPMGPGKAFYARGDITGDIRKAYCEINGTEWHSTLKLVVEKLREQY-N	349
38	TCTRPNNNTRKSIPMGPGKAFYARGDITGDIRKAYCEINGTEWHSTLKLVVEKLREQY-N	347
39	TCTRPNNNTRKSIPMGPGKAFYARGDITGDIRKASCEINGTEWHSTLKLVVEKLREQY-N	349
40	TCTRPNNNTRKSIPMGPGKAFYARGDITGDIRKAYCEINGTEWHSTLKLVVEKLREQY-N	349
41	TCTRPNNNTRKSIPMGPGKAFYARGDITGDIRKAYCEINGTEWHSTLKLVVEKLREQY-N	349
42	TCTRPNNNTRKSIPMGPGKAFYARGDITGDIRKAYCEINGTEWHSTLKLVVEKLREQY-N	349
43	TCTRPNNNTRKSIPMGPGKAFYARGDITGDIRKAYCEINGTEWHSTLKMVVEKLREQY-N	349
44	TCTRPNNNTRKSIPMGPGKAFYARGDITGDIRKAYCEINGTEWHSTLKLVVEKLREQY-N	349
45	TCTRPNNNTRKSIPMGPGKAFYARGDITGDIRKAYCEINGTEWHSTLKLVVEKLREQY-N	349
46	TCTRPNNNTRKSIPMGPGKAFYARGDITGDIRKAYCEINGTEWHSTLKLVVEKLREQY-N	349
47	TCTRPNNNTRKSIPMGPGKAFYARGDITGDIRKAYCEINGTEWHSTLKLVVEKLREQY-N	349
48	TCTRPNNNTRKSIPMGPGKAFYARGDITGDIRKAYCEINGTEWHSTLKLVVEKLREQY-N	349
49	NCTRPNNNTRKSIPMGPGKAFYARGDITGDIRKAYCEINGTEWHSTLKLVVEKLREQY-N	349
50	TCTRPNNNTRKSIPMGPGKAFYARGDITGDIKKACCEINGTEWHSTLKLVVEKLREQY-N	349
51	TCIRPNNNTRKSIPMGPGKAFYARGDITGDIKKAYCEINGTEWHSTLKLVVEKLREQY-N	349
52	TCTRPNNNTRKSIPMGPGKAFYARGDITGDIRKAYCEINGTEWHSTLKLVVEKLREQY-N	349
53	TCTRPNNNTRKSIPMGPGKAFYARGDITGDIKKAYCEINGTEWHSTLKLVVEKLREQY-N	349
54	TCTRPNNNTRKSIPMGPGKAFYARGDITGDIRKAYCKINGTEWHSTLKLVVKKLREQY-N	349
55	TCTRPNNNTRKSIPMGPGKAFYARGDITGDIRKAYCEINGTEWHSTLKLVVEKLREQY-N	349
56	TCTRPNNNTRKSIPMGPGKAFYARGDITGDIRKAYCEINGTEWHSTLKLVVEKLREQY-N	349
57	TCTRPNNNTRKSIPMGPGKAFYARGDITGDIRKAYCEINGTEWHSTLKLVVEKLREQY-N	349
58	TCTRPNNNTRKSIPMGPGKAFYARGDITGDIRKAYCEINGTEWHSTLKLVVEKLREQY-N	349
59	TCTRPNNNTRKSIPMGPGKAFYARGDITGDIRKAYCEINGTEWHSTLKLVVEKLREQY-N	347
60	TCTRPNNNTRKSIPMGPGKAFYARGDITGDIRKAYCEINGTEWHSTLKLVVEKLREQY-N	349
61	TCTRPNNNTRKSIPMGPGKAFYARGDITGDIRKAYCGINGTEWHSTLKLVVEKLREQY-N	349
62	NCTRPNNNTRKSIPMGPGKAFYARGDITGDIRKAYCEINGTEWHSTLKLVVEKLREQY-N	349
63	TCTRPNNNTRKSIPMGPGKAFYARGDITGDIRKAYCKINGTEWHSTLKLVVEKLREQY-N	349
64	TCTRPNNNTRKSIPMGPGKAFYARGDITGDIRKAYCEINGTEWHSTLKLVVEKLREQY-N	349
65	TCTRPNNNTRKSIPMGPGKAFYARGDITGDIRKAYCEINGTEWHSTLKLVVEKLREQY-N	349
66	TCTRPNNNTRKSIPMGPGKAFYARGDITGDIRKAYCEINGTEWHSTLKLVVEKLREQY-N	349
67	TCTRPNNNTRKSIPMGPGKAFYARGDITGDIRKAYCEINGTEWHSTLKLVVEKLREQY-N	347
68	TCTRPNNNTRKSIPMGPGKAFYARGDITGDIRKAYCEINGTEWHSTLKLVVEKLREQY-N	349

	KTIVFNRSSGGDPEIVMYSFNCGGEFFYCNSTKLFNSTWPWND-TKGSHDTNGTLILPCK	
1	KTIVFNRSSGGDPEIVMYSFNCGGEFFYCNSTKLFNSTWPWND-TKGSHDTNGTLILPCK	408
2	KTIVFNRSSGGDPEIVMYSFNCGGEFFYCNSTRLFNSTWPWND-TKGSHDTNGTLILPCK	407
3	KTIVFNRSSGGDPEIVMYSFNCGGEFFYCNSTKLFNSTWPWND-TKGSHDTNGTLILPCK	408
4	KTIVFNRSSGGDPEIVMYSFNCGGEFFYCNSTKLFNSTWPWND-TKGSHDTNGTLILPCK	408
5	KTIVFNRSSGGDPEIVMYSFNCGGEFFYCNSTKLFNSTWPWND-TKGSHDTNGTLILPCK	408
6	KTIVFNRSSGGDPEIVMYSFNCGGEFFYCNSTKLFNSTWPWND-TKGSHDTNGTLILPCK	408
7	KTIVFNRSSGGDPEIVMYSFNCGGEFFYCNSTKLFNSTWPWND-TKGSHDTNGTLILPCK	408
8	KTIVFNRSSGGDPEIVMYSFNCGGEFFYCNSTKLFNSTWPWND-TKGSHDTNGTLILPCK	408
9	KTIVFNRSSGGDPEIVMYSFNCGGEFFYCNSTKLFNSTWPNDT--KGSHDTNDTLILPCK	407
10	KTIVFNRSSGGDPEIVMYSFNCGGEFFYCNSTKLFNSTWPWND-TKGSHDTNGTLILPCK	408
11	KTIVFNRSSGGDPEIVMYSFNCGGEFFYCNSTKLFNSTWPWND-TKGSHDTNGTLILPCK	408
12	KTIVFNRSSGGDPEIVMYSFNCGGEFFYCNSTKLFNSTWPWND-TKGSHDTNGTLILPCK	408
13	KTIVFNRSSGGDPEIVMYSFNCGGEFFYCNSTKLFNSTWPWND-TKGSHDTNGTLILPCK	408
14	KTIVFNRSSGGDPEIVMYSFNCGGEFFYCNSTKLFNSTWPWND-TKGSHDTNGTLILPCK	406
15	KTIVFNRSSGGDPEIVMYSFNCGGEFFYCNSTKLFNSTWPWND-TKGSHDTNGTLILPCK	376
16	KTIVFNRSSGGDPEIVMYSFNCGGEFFYCNSTKLFNSTWPWND-TKGSHDTNGTLILPCK	408
17	KTIVFNRSSGGDPEIVMYSFNCGGEFFYCNSTKLFNSTWPWND-TKGSHDTNGTLILPCK	408
18	KTIVFNRSSGGDPEIVMYSFNCGGEFFYCNSTKLFNSTWPWND-TKGSHDTNGTLILPCK	408
19	KTIVFNRSSGGDPEIVMYSFNCGGEFFYCNSTKLFNSTWPWND-TKGSHDTNGTLILPCK	408
20	KTIVFNRSSGGDPEIVMYSFNCGGEFFYCNSTKLFNSTWPWND-TKGSHDTNGTLILPCK	406
21	KTIVFNRSSGGDPEIVMYSFNCGGEFFYCNSTKLFNSTWPWND-TKGSHDTNGTLRLPCK	408
22	KTIVFNRSSGGDPEIVMYSFNCGGEFFYCNSTKLFNSTWPWND-TKGSHDTNGTLILPCK	391
23	KTIVFNRSSGGDPEIVMYSFNCGGEFFYCNSTKLFNSTWPWND-TKGSHDTNGTLILPCK	408
24	KTIVFNRSSGGDPEIVMYSFNCGGEFFYCNSTKLFNSTWPWND-TKGSHDTNGTLILPCK	407
25	KTIVFNRSSGGDPEIVMYSFNCGGEFFYCNSTKLFNSTWPWND-TKGSHDTNGTLILPCK	408
26	KTIVFNRSSGGDPEIVMYSFNCGGEFFYCNSTKLFNSTWPWND-TKGSHDTNGTLILPCK	408
27	KTIVFNRSSGGDPEIVMYSFNCGGEFFYCNSTKLFNSTWPWND-TKGSHDTNGTLILPCK	408
28	KTIVFNRSSGGDPEIVMYSFNCGGEFFYCNSTKLFNSTWPWND-TKGSHDTNGTLILPCK	408
29	KTIVFNRSSGGDPEIVMYSFNCGGEFFYCNSTKLFNSTWPWND-TKGSHDTNGTLILPCK	408
30	KTIVFNRSSGGDPEIVMYSFNCGGEFFYCNSTKLFNSTWPWND-TKGSHDTNGTLILPCK	408
31	KTIVFNRSSGGDPEIVMYSFNCGGEFFYCNSTKLFNSTWPWND-TKGSHDTNGTLILPCK	408
32	KTIVFNRSSGGDPEIVMYSFNCGGEFFYCNSTKLFNSTWPWND-TKGSHDTNGTLILPCK	408
33	KTIVFNRSSGGDPEIVMYSFNCGGEFFYCNSTKLFNSTWPWND-TKGSHDTNGTLILPCK	408
34	KTIVFNRSSGGDPEIVMYSFNCGGEFFYCNSTKLFNSTWPWND-TKGSHDTNGTLILPCK	408
35	KTIVFNRSSGGDPEIVMYSFNCGGEFFYCNSTKLFNSTWPWND-TKGSHDTNGTLILPCK	408
36	KTIVFNRSSGGDPEIVMYSFNCGGEFFYCNSTKLFNSTWHWND-TKGSHDTNGTLILPCK	408
37	KTIVFNRSSGGDPEIVMYSFNCGGEFFYCNSTKLFNSTWPWND-TKGSHDTNGTLILPCK	408
38	KTIVFNRSSGGDPEIVMYSFNCGGEFFYCNSTKLFNSTWPWND-TKGSHDTNGTLILPCK	406
39	KTIVFNRSSGGDPEIVMYSFNCGGEFFYCNSTKLFNSTWPWND-TKGSHDTNGTLILPCK	408
40	KTIVFNRSSGGDPEIVMYSFNCGGEFFYCNSTKLFNSTWPWND-TKGSHDTNGTLILPCK	408
41	KTIVFNRSSGGDPEIVMYSFNCGGEFFYCNSTKLFNSTWPWND-TKGSHDTNGTLILPCK	408
42	KTIVFNRSSGGDPEIVMYSFNCGGEFFYCNSTKLFNSTWPWND-TKGSHDTNDTLMLPCK	408
43	KTIVFNRSSGGDPEIVMYSFNCGGEFFYCNSTKLFNSTWPWND-TKGSHDTNDTLMLPCK	408
44	KTIVFNRSSGGDPEIVMYSFNCGGEFFYCNSTKLFNSTWPWND-TKGSHDTNGTLTLPCK	408
45	KTIVFNRSSGGDPEIVMYSFNCGGEFFYCNSTKLFNSTWPWND-TKGSHDTNDTLMLPCK	408
46	KTIVFNRSSGGDPEIVMYSFNCGGEFFYCNSTKLFNSTWPWND-TKGSHDTNGTLILPCK	408
47	KTIVFNRSSGGDPEIVMYSFNCGGEFFYCNSTKLFNSTWPWND-TKGSHDTNGTLTLPCK	408
48	KTIVFNRSSGGDPEIVMYSFNCGGEFFYCNSTKLFNSTWPWND-TKGSHDTNDTLMLPCK	408
49	KTIVFNRSSGGDPEIVMYSFNCGGEFFYCNSTKLFNSTWLWND-TKGSHDTNGTLILPCK	408
50	KTIVFNRSSGGDPEIVMYSFNCGGEFFYCNSTKLFNSTWPWND-TKGSHDTNGTLILPCK	408
51	KTIVFNRSSGGDPEIVMYSFNCGGEFFYCNSTKLFNSTWPWND-TKGSHDTNGTLILPCK	408
52	KTIVFNRSSGGDPEIVMYSFNCGGEFFYCNSTKLFNSTWPWND-TKGSHDTNDTLMLPCK	408
53	KTIVFNRSSGGDPEIVMYSFSCGGEFFYCNSTKLFNSTWPWND-TKGSHDTNGTLRLPCK	408
54	KTIVFNRSSGGDPEIVMYSFNCGGEFFYCNSTKLFNSTWPWND-TKGSHDTNGTLILPCK	408

55	KTIVFNRSSGGDPEIVMYSFNCGGEFFYCNSTKLFNSTWPWND-TKGSHDTNGTLILPCK	408
56	KTIVFNRSSGGDPEIVMYSFNCGGEFFYCNSTKLFNSTWPWND-TKGSHDTNDTLMLPCK	408
57	KTIVFNRSSGGDPEIVMYSFNCGGEFFYCNSTKLFNSTWPWND-TKGSHDTNGTLTLPCK	408
58	KTIVFNRSSGGDPEIVMYSFNCGGEFFYCNSTKLFNSTWPWND-TKGSHDTNGTLKLPCK	408
59	KTIVFNRSSGGDPEIVMYSFNCGGEFFYCNSTKLFNSTWPWND-TKGSHDTNGTLMLPCK	406
60	KTIVFNRSSGGDPEIVMYSFNCGGEFFYCNSTKLFNSTWPWND-TKGSHDTNDTLMLPCK	408
61	KTIVFNRSSGGDPEIVMYSFNCGGEFFYCNSTKLFNSTWPWND-TKGSHDTNGTLILPCK	408
62	KTIVFNRSSGGDPEIVMYSFNCGGEFFYCNSTKLFNSTWPWND-TKGSHDTNGTLILPCK	408
63	KTIVFNRSSGGDPEIVMYSFNCGGEFFYCNSTKLFNSTWPWND-TKGSHDTNGTLILPCK	408
64	KTIVFNRSSGGDPEIVMYSFNCGGEFFYCNSTKLFNSTWPWND-TKGSHDTNDTLMLPCK	408
65	KTIVFNRSSGGDPEIVMYSFNCGGEFFYCNSTKLFNSTWPWND-TKGSHDTNDTLMLPCK	408
66	KTIVFNRSSGGDPEIVMYSFNCGGEFFYCNSTKLFNSTWPWND-TKGSHDTNDTLMLPCK	408
67	KTIVFNRSSGGDPEIVMYSFNCGGEFFYCNSTKLFNSTWPWND-TKGSHDTNGTLILPCK	406
68	KTIVFNRSSGGDPEIVMYSFNCGGEFFYCNSTKLFNSTWPWND-TKGSHDTNDTLMLPCK	408

	**IKQIINMWQGVGKAMYAPPIEGKIRCSSNITGLLLTRDGG-YESNETDEIFRPGGGDMRD**	
1	IKQIINMWQGVGKAMYAPPIEGKIRCSSNITGLLLTRDGG-YESNETDEIFRPGGGDMRD	467
2	IKQIINMWQGVGKAMYAPPIEGKIRCSSNITGLLLTRDGG-YESNETDEIFRPGGGDMRD	466
3	IKQIINMWQGVGKAMYAPPIEGKIRCSSNITGLLLTRDGG-YESNETDEIFRPGGGDMRD	467
4	IKQIINMWQGVGKAMYAPPIEGKIRCSSNITGLLLTRDGG-YESNETDEIFRPGGGDMRD	467
5	IKQIINMWQGVGKAMYAPPIEGKIRCSSNITGLLLTRDGG-YESNETDEIFRPGGGDMRD	467
6	IKQIINMWQGVGKAMYAPPIEGKIRCSSNITGLLLTRDGG-YESNETDEIFRPGGGDMRD	467
7	IKQIINMWQGVGKAMYAPPIEGKIRCSSNITGLLLTRDGG-YESNETDEIFRPGGGDMRD	467
8	IKQIINMWQGVGKAMYAPPIEGKIRCSSNITGLLLTRDGG-YESNETDEIFRPGGGDMRD	467
9	IKQIINMWQGVGKAMYAPPIEGKIRCSSNITGLLLTRDGG-YESNETDEIFRPGGGDMRD	466
10	IKQIINMWQGVGKAMYAPPIEGKIRCSSNITGLLLTRDGG-YESNETDEIFRPGGGDMRD	467
11	IKQIINMWQGVGKAMYAPPIEGKIRCSSNITGLLLTRDGG-YESNETDEIFRPGGGDMRD	467
12	IKQIINMWQGVGKAMYAPPIEGKIRCSSNITGLLLTRDGG-YESNETDEIFRPGGGDMRD	467
13	IKQIINMWQGVGKAMYAPPIEGKIRCSSNITGLLLTRDGG-YESNETDEIFRPGGGDMRD	467
14	IKQIINMWQGVGKAMYAPPIEGKIRCSSNITGLLLTRDGG-YESNETDEIFRPGGGDMRD	465
15	IKQIINMWQGVGKAMYAPPIEGKIRCSSNITGLLLTRDGG-YESNETDEIFRPGGGDMRD	435
16	IKQIINMWQGVGKAMYAPPIEGKIRCSSNITGLLLTRDGG-YESNETDEIFRPGGGDMRD	467
17	IKQIINMWQGVGKAMYAPPIEGKIRCSSNITGLLLTRDGG-YESNETDEIFRPGGGDMRD	467
18	IKQIINMWQGVGKAMYAPPIEGKIRCSSNITGLLLTRDGG-YESNETDEIFRPGGGDMRD	467
19	IKQIINMWQGVGKAMYAPPIEGKIRCSSNITGLLLTRDGG-YESNETDEIFRPGGGDMRD	467
20	IKQIINMWQGVGKAMYAPPIEGKIRCSSNITGLLLTRDGG-YESNETDEIFRPGGGDMRD	465
21	IKQIINMWQGVGKAMYAPPIEGKIRCSSNITGLLLTRDGG-YESNETDEIFRPGGGDMRD	467
22	IKQIINMWQGVGKAMYAPPIEGKIRCSSNITGLLLTRDGG-YESNETDEIFRPGGGDMRD	450
23	IKQIINMWQGVGKAMYAPPIEGKIRCSSNITGLLLTRDGG-YESNETDEIFRPGGGDMRD	467
24	IKQIINMWQGVGKAMYAPPIEGKIRCSSNITGLLLTRDGG-YESNETDEIFRPGGGDMRD	466
25	IKQIINMWQGVGKAMYAPPIEGKIRCSSNITGLLLTRDGG-YESNETDEIFRPGGGDMRD	467
26	IKQIINMWQGVGKAMYAPPIEGKIRCSSNITGLLLTRDGG-YESNETDEIFRPGGGDMRD	467
27	IKQIINMWQGVGKAMYAPPIEGKIRCSSNITGLLLTRDGG-YESNETDEIFRPGGGDMRD	467
28	IKQIINMWQGVGKAMYAPPIEGKIRCSSNITGLLLTRDGG-YESNETDEIFRPGGGDMRD	467
29	IKQIINMWQGVGKAMYAPPIEGKIRCSSNITGLLLTRDGG-YESNETDEIFRPGGGDMRD	467
30	IKQIINMWQGVGKAMYAPPIEGKIRCSSNITGLLLTRDGG-YESNETDEIFRPGGGDMRD	467
31	IKQIINMWQGVGKAMYAPPIEGKIRCSSNITGLLLTRDGG-YESNETDEIFRPGGGDMRD	467
32	IKQIINMWQGVGKAMYAPPIEGKIRCSSNITGLLLTRDGG-YESNETDEIFRPGGGDMRD	467
33	IKQIINMWQGVGKAMYAPPIEGKIRCSSNITGLLLTRDGG-YESNETDEIFRPGGGDMRD	467
34	IKQIINMWQGVGKAMYAPPIEGKIRCSSNITGLLLTRDGG-YESNETDEIFRPGGGDMRD	467
35	IKQIINMWQGVGKAMYAPPIEGKIRCSSNITGLLLTRDGG-YESNETDEIFRPGGGDMRD	467
36	IKQIINMWQGVGKAMYAPPIEGKIRCSSNITGLLLTRDGG-YESNETDEIFRPGGGDMRD	467

37	IKQIINMWQGVGKAMYAPPIEGKIRCSSNITGLLLTRDGG-YESNETDEIFRPGGGDMRD	467
38	IKQIINMWQGVGKAMYAPPIEGKIRCSSNITGLLLTRDGG-YESNETDEIFRPGGGDMRD	465
39	IKQIINMWQGVGKAMYAPPIEGKIRCSSNITGLLLTRDGG-YESNETDEIFRPGGGDMRD	467
40	IKQIINMWQGVGKAMYAPPIEGKIRCSSNITGLLLTRDGG-YESNETDEIFRPGGGDMRD	467
41	IKQIINMWQGVGKAMYAPPIEGKIRCSSNITGLLLTRDGG-YESNETDEIFRPGGGDMRD	467
42	IKQIINMWQGVGKAMYAPPIEGKIRCSSNITGLLLTRDGG-YESNETDEIFRPGGGDMRD	467
43	IKQIINMWQGVGKAMYAPPIEGKIRCSSNITGLLLTRDGG-YESNETDEIFRPGGGDMRD	467
44	IKQIINMWQGVGKAMYAPPIEGKIRCSSNITGLLLTRDGG-YESNETDEIFRPGGGDMRD	467
45	IKQIINMWQGVGKAMYAPPIEGKIRCSSNITGLLLTRDGG-YESNETDEIFRPGGGDMRD	467
46	IKQIINMWQGVGKAMYAPPIEGKIRCSSNITGLLLTRDGG-YESNETDEIFRPGGGDMRD	467
47	IKQIINMWQGVGKAMYAPPIEGKIRCSSNITGLLLTRDGG-YESNETDEIFRPGGGDMRD	467
48	IKQIINMWQGVGKAMYAPPIEGKIRCSSNITGLLLTRDGG-YESNETDEIFRPGGGDMRD	467
49	IKQIINMWQGVGKAMYAPPIEGKIRCSSNITGLLLTRDGG-YESNETDEIFRPGGGDMRD	467
50	IKQIINMWQGVGKAMYAPPIEGKIRCSSNITGLLLTRDGG-YESNETDEIFRPGGGDMRD	467
51	IKQIINMWQGVGKAMYAPPIEGKIRCSSNITGLLLTRDGG-YESNETDEIFRPGGGDMRD	467
52	IKQIINMWQGVGKAMYAPPIEGKIRCSSNITGLLLTRDGG-YESNETDEIFRPGGGDMRD	467
53	IKQIINMWQGVGKAMYAPPIEGKIRCSSNITGLLLTRDGG-YESNETDEIFRPGGGDMRD	467
54	IKQIINMWQGVGKAMYAPPIEGKIRCSSNITGLLLTRDGG-YESNETDEIFRPGGGDMRD	467
55	IKQIINMWQGVGKAMYAPPIEGKIRCSSNITGLLLTRDGG-YESNETDEIFRPGGGDMRD	467
56	IKQIINMWQGVGKAMYAPPIEGKIRCSSNITGLLLTRDGG-YESNETDEIFRPGGGDMRD	467
57	IKQIINMWQGVGKAMYAPPIEGKIRCSSNITGLLLTRDGG-YESNETDEIFRPGGGDMRD	467
58	IKQIINMWQGVGKAMYAPPIEGKIRCSSNITGLLLTRDGG-YESNETDEIFRPGGGDMRD	467
59	IKQIINMWQGVGKAMYAPPIEGKIRCSSNITGLLLTRDGG-YESNETDEIFRPGGGDMRD	465
60	IKQIINMWQGVGKAMYAPPIEGKIRCSSNITGLLLTRDGG-YESNETDEIFRPGGGDMRD	467
61	IKQIINMWQGVGKAMYAPPIEGKIRCSSNITGLLLTRDGG-YESNETDEIFRPGGGDMRD	467
62	IKQIINMWQGVGKAMYAPPIEGKIRCSSNITGLLLTRDGG-YESNETDEIFRPGGGDMRD	467
63	IKQIINMWQGVGKAMYAPPIEGKIRCSSNITGLLLTRDGG-YESNETDEIFRPGGGDMRD	467
64	IKQIINMWQGVGKAMYAPPIEGKIRCSSNITGLLLTRDGG-YESNETDEIFRPGGGDMRD	467
65	IKQIINMWQGVGKAMYAPPIEGKIRCSSNITGLLLTRDGG-YESNETDEIFRPGGGDMRD	467
66	IKQIINMWQGVGKAMYAPPIEGKIRCSSNITGLLLTRDGG-YESNETDEIFRPGGGDMRD	467
67	IKQIINMWQGVGKAMYAPPIEGKIRCSSNITGLLLTRDGG-YESNETDEIFRPGGGDMRN	465
68	IKQIINMWQGVGKAMYAPPIEGKIRCSSNITGLLLTRDGG-YESNETDEIFRPGGGDMRD	467

NWRSELYKYKVVKIEPLGVAPTKAKRRVVQREKRAFG-LGAVFLGFLGAAGSTMGAASIT

1	NWRSELYKYKVVKIEPLGVAPTKAKRRVVQREKRAFG-LGAVFLGFLGAAGSTMGAASIT	526
2	NWRSELYKYKVVKIEPLGVAPTKAKRRVVQREKRAFG-LGAVFLGFLGAAGSTMGAASIT	525
3	NWRSELYKYKVVKIEPLGVAPTKAKRRVVQREKRAFG-LGAVFLGFLGAAGSTMGAASIT	526
4	NWRSELYKYKVVKIEPLGVAPTKAKRRVVQREKRAFG-LGAVFLGFLGAAGSTMGAASIT	526
5	NWRSELYKYKVVKIEPLGVAPTKAKRRVVQREKRAFG-LGAVFLGFLGAAGSTMGAASIT	526
6	NWRSELYKYKVVKIEPLGVAPTKAKRRVVQREKRAFG-LGAVFLGFLGAAGSTMGAASIT	526
7	NWRSELYKYKVVKIEPLGVAPTKAKRRVVQREKRAFG-LGAVFLGFLGAAGSTMGAASIT	526
8	NWRSELYKYKVVKIEPLGVAPTKAKRRVVQREKRAFG-LGAVFLGFLGAAGSTMGAASIT	526
9	NWRSELYKYKVVKIEPLGVAPTKAKRRVVQREKRAFG-LGAVFLGFLGAAGSTMGAASIT	525
10	NWRSELYKYKVVKIEPLGVAPTKAKRRVVQREKRAFG-LGAVFLGFLGAAGSTMGAASIT	526
11	NWRSELYKYKVVKIEPLGVAPTKAKRRVVQREKRAFG-LGAVFLGFLGAAGSTMGAASIT	526
12	NWRSELYKYKVVKIEPLGVAPTKAKRRVVQREKRAFG-LGAVFLGFLGAAGSTMGAASIT	526
13	NWRSELYKYKVVKIEPLGVAPTKAKRRVVQREKRAFG-LGAVFLGFLGAAGSTMGAASIT	526
14	NWRSELYKYKVVKIEPLGVAPTKAKRRVVQREKRAFG-LGAVFLGFLGAAGSTMGAASIT	524
15	NWRSELYKYKVVKIEPLGVAPTKAKRRVVQREKRAFG-LGAVFLGFLGAAGSTMGAASIT	494
16	NWRSELYKYKVVKIEPLGVAPTKAKRRVVQREKRAFG-LGAVFLGFLGAAGSTMGAASIT	526
17	NWRSELYKYKVVKIEPLGVAPTKAKRRVVQREKRAFG-LGAVFLGFLGAAGSTMGAASIT	526
18	NWRSELYKYKVVKIEPLGVAPTKAKRRVVQREKRAFG-LGAVFLGFLGAAGSTMGAASIT	526

19	NWRSELYKYKVVKIEPLGVAPTKAKRRVVQREKRAFG-LGAVFLGFLGAAGSTMSAASIT	526
20	NWRSELYKYKVVKIEPLGVAPTKAKRRVVQREKRAFG-LGAVFLGFLGAAGSTMGAASIT	524
21	NWRSELYKYKVVKIEPLGVAPTKAKRRVVQREKRAFG-LGAVFLGFLGAAGSTMGAASIT	526
22	NWRSELYKYKVVKIEPLGVAPTKAKRRVVQREKRAFG-LGAVFLGFLGAAGSTMGAASIT	509
23	NWRSELYKYKVVKIEPLGVAPTKAKRRVVQREKRAFG-LGAVFLGFLGAAGSTMGAASIT	526
24	NWRSELYKYKVVKIEPLGVAPTKAKRRVVQREKRAFG-LGAVFLGFLGAAGSTMGAASIT	525
25	NWRSELYKYKVVKIEPLGVAPTKAKRRVVQREKRAFG-LGAVFLGFLGAAGSTMGAASIT	526
26	NWRSELYKYKVVKIEPLGVAPTKAKRRVVQREKRAFG-LGAVFLGFLGAAGSTMGAASIT	526
27	NWRSELYKYKVVKIEPLGVAPTKAKRRVVQREKRAFG-LGAVFLGFLGAAGSTMGAASIT	526
28	NWRSELYKYKVVKIEPLGVAPTKAKRRVVQREKRAFG-LGAVFLGFLGAAGSTMGAASIT	526
29	NWRSELYKYKVVKIEPLGVAPTKAKRRVVQREKRAFG-LGAVFLGFLGAAGSTMGAASIT	526
30	NWRSELYKYKVVKIEPLGVAPTKAKRRVVQREKRAFG-LGAVFLGFLGAAGSTMGAASIT	526
31	NWRSELYKYKVVKIEPLGVAPTKAKRRVVQREKRAFG-LGAVFLGFLGAAGSTMGAASIT	526
32	NWRSELYKYKVVKIEPLGVAPTKAKRRVVQREKRAFG-LGAVFLGFLGAAGSTMGAASIT	526
33	NWRSELYKYKVVKIEPLGVAPTKAKRRVVQREKRAFG-LGAVFLGFLGAAGSTMGAASIT	526
34	NWRSELYKYKVVKIEPLGVAPTKAKRRVVQREKRAFG-LGAVFLGFLGAAGSTMGAASIT	526
35	NWRSELYKYKVVKIEPLGVAPTKAKRRVVQREKRAFG-LGAVFLGFLGAAGSTMGAASIT	526
36	NWRSELYKYKVVKIEPLGVAPTKAKRRVVQREKRAFG-LGAVFLGFLGAAGSTMGAASIT	526
37	NWRSELYKYKVVKIEPLGVAPTKAKRRVVQREKRAFG-LGAVFLGFLGAAGSTMGAASIT	526
38	NWRSELYKYKVVKIEPLGVAPTKAKRRVVQREKRAFG-LGAVFLGFLGAAGSTMGAASIT	524
39	NWRSELYKYKVVKIEPLGVAPTKAKRRVVQREKRAFG-LGAVFLGFLGAAGSTMGAASIT	526
40	NWRSELYKYKVVKIEPLGVAPTKAKRRVVQREKRAFG-LGAVFLGFLGAAGSTMGAASIT	526
41	NWRSELYKYKVVKIEPLGVAPTKAKRRVVQREKRAFG-LGAVFLGFLGAAGSTMGAASIT	526
42	NWRSELYKYKVVKIEPLGVAPTKAKRRVVQREKRAFG-LGAVFLGFLGAAGSTMGAASIT	526
43	NWRSELYKYKVVKIEPLGVAPTKAKRRVVQREKRAFG-LGAVFLGFLGAAGSTMGAASIT	526
44	NWRSELYKYKVVKIEPLGVAPTKAKRRVVQREKRAFG-LGAVFLGFLGAAGSTMGAASIT	526
45	NWRSELYKYKVVKIEPLGVAPTKAKRRVVQREKRAFG-LGAVFLGFLGAAGSTMGAASIT	526
46	NWRSELYKYKVVKIEPLGVAPTKAKRRVVQREKRAFG-LGAVFLGFLGAAGSTMGAASIT	526
47	NWRSELYKYKVVKIEPLGVAPTKAKRRVVQREKRAFG-LGAVFLGFLGAAGSTMSAASIT	526
48	NWRSELYKYKVVKIEPLGVAPTKAKRRVVQREKRAFG-LGAVFLGFLGAAGSTMGAASIT	526
49	NWRSELYKYKVVKIEPLGVAPTKAKRRVVQREKRAFG-LGAVFLGFLGAAGSTMGAASIT	526
50	NWRSELYKYKVVKIEPLGVAPTKAKRRVVQREKRAFG-LGAVFLGFLGAAGSTMGAASIT	526
51	NWRSELYKYKVVKIEPLGVAPTKAKRRVVQREKRAFG-LGAVFLGFLGAAGSTMGAASIT	526
52	NWRSELYKYKVVKIEPLGVAPTKAKRRVVQREKRAFG-LGAVFLGFLGAAGSTMGAASIT	526
53	NWRSELYKYKVVKIEPLGVAPTKAKRRVVQREKRAFG-LGAVFLGFLGAAGSTMGAASIT	526
54	NWRSELYKYKVVKIEPLGVAPTKAKRRVVQREKRAFG-LGAVFLGFLGAAGSTMGAASIT	526
55	NWRSELYKYKVVKIEPLGVAPTKAKRRVVQREKRAFG-LGAVFLGFLGAAGSTMGAASIT	526
56	NWRSELYKYKVVKIEPLGVAPTKAKRRVVQREKRAFG-LGAVFLGFLGAAGSTMGAASIT	526
57	NWRSELYKYKVVKIEPLGVAPTKAKRRVVQREKRAFG-LGAVFLGFLGAAGSTMSAASIT	526
58	NWRSELYKYKVVKIEPLGVAPTKAKRRVVQREKRAFG-LGAVFLGFLGAAGSTMGAASIT	526
59	NWRSELYKYKVVKIEPLGVAPTKAKRRVVQREKRAFG-LGAVFLGFLGAAGSTMGAASIT	524
60	NWRSELYKYKVVKIEPLGVAPTKAKRKVVQREKRAFG-LGAVFLGFLGAAGSTMGAASIT	526
61	NWRSELYKYKVVKIEPLGVAPTKAKRRVVQREKRAFG-LGAVFLGFLGAAGSTMGAASIT	526
62	NWRSELYKYKVVKIEPLGVAPTKAKRRVVQREKRAFG-LGAVFLGFLGAAGSTMGAASIT	526
63	NWRSELYKYKVVKIEPLGVAPTKAKRRVVQREKRAFG-LGAVFLGFLGAAGSTMGAASIT	526
64	NWRSELYKYKVVKIEPLGVAPTKAKRRVVQREKRAFG-LGAVFLGFLGAAGSTMGAASIT	526
65	NWRSELYKYKVVKIEPLGVAPTKAKRRVVQREKRAFG-LEAVFLGFLGAAGSTMGAASIT	526
66	NWRSELYKYKVVKIEPLGVAPTKAKRRVVQREKRAFG-LGAVFLGFLGAAGSTMGAASIT	526
67	NWRSELYKYKVVKIEPLGVAPTKAKRRVVQREKRAFG-LGAVFLGFLGAAGSTMGAASIT	524
68	NWRSELYKYKVVKIEPLGVAPTKAKRRVVQREKRAFG-LGAVFLGFLGAAGSTMGAASIT	526

LTVQARQLLSGIVQQQNNLLRAIEAQQHLLQLTVWGIKQLQARVLAVERYLKDQQLLGIW

1	LTVQARQLLSGIVQQQNNLLRAIEAQQHLLQLTVWGIKQLQARVLAVERYLKDQQLLGIW	586
2	LTVQARQLLSGIVQQQNNLLRAIEAQQHLLQLTVWGIKQLQARVLAVERYLKDQQLLGIW	585
3	LTVQARQLLSGIVQQQNNLLRAIEAQQHLLQLTVWGIKQLQARVLAVERYLKDQQLLGIW	586
4	LTVQARQLLSGIVQQQNNLLRAIEAQQHLLQLTVWGIKQLQTRVLAVERYLKDQQLLGIW	586
5	LTVQARQLLSGIVQQQNNLLRAIEAQQHLLQLTVWGIKQLQARVLAVERYLKDQQLLGIW	586
6	LTVQARQLLSGIVQQQNNLLRAIEAQQHLLQLTVWGIKQLQARVLAVERYLKDQQLLGIW	586
7	LTVQARQLLSGIVQQQNNLLRAIEAQQHLLQLTVWGIKQLQARVLAVERYLKDQQLLGIW	586
8	LTVQARQLLSGIVQQQNNLLRAIEAQQHLLQLTVWGIKQLQARVLAVERYLKDQQLLGIW	586
9	LTVQARQLLSGIVQQQNNLLRAIEAQQHLLQLTVWGIKQLQARVLAVERYLKDQQLLGIW	585
10	LTVQARQLLSGIVQQQNNLLRAIEAQQHLLQLTVWGIKQLQARVLAVERYLKDQQLLGIW	586
11	LTVQARQLLSGIVQQQNNLLRAIEAQQHLLQLTVWGIKQLQARVLAVERYLKDQQLLGIW	586
12	LTVQARQLLSGIVQQQNNLLRAIEAQQHLLQLTVWGIKQLQARVLAVERYLKDQQLLGIW	586
13	LTVQARQLLSGIVQQQNNLLRAIEAQQHLLQLTVWGIKQLQARVLAVERYLKDQQLLGIW	586
14	LTVQARQLLSGIVQQQNNLLRAIEAQQHLLQLTVWGIKQLQARVLAVERYLKDQQLLGIW	584
15	LTVQARQLLSGIVQQQNNLLRAIEAQQHLLQLTVWGIKQLQARVLAVERYLKDQQLLGIW	554
16	LTVQARQLLSGIVQQQNNLLRAIEAQQHLLQLTVWGIKQLQARVLAVERYLKDQQLLGIW	586
17	LTVQARQLLSGIVQQQNNLLRAIEAQQHLLQLTVWGIKQLQARVLAVERYLKDQQLLGIW	586
18	LTVQARQLLSGIVQQQNNLLRAIEAQQHLLQLTVWGIKQLQARVLAVERYLKDQQLLGIW	586
19	LTVQARQLLSGIVQQQNNLLRAIEAQQHLLQLTVWGIKQLQARVLAVERYLKDQQLLGIW	586
20	LTVQARQLLSGIVQQQNNLLRAIEAQQHLLQLTVWGIKQLQARVLAVERYLKDQQLLGIW	584
21	LTVQARQLLSGIVQQQNNLLRAIEAQQHLLQLTVWGIKQLQARVLAVERYLKDQQLLGIW	586
22	LTVQARQLLSGIVQQQNNLLRAIEAQQHLLQLTVWGIKQLQARVLAVERYLKDQQLLGIW	569
23	LTVQARQLLSGIVQQQNNLLRAIEAQQHLLQLTVWGIKQLQARVLAVERYLKDQQLLGIW	586
24	LTVQARQLLSGIVQQQNNLLRAIEAQQHLLQLTVWGIKQLQARVLAVERYLKDQQLLGIW	585
25	LTVQARQLLSGIVQQQNNLLRAIEAQQHLLQLTVWGIKQLQARVLAVERYLKDQQLLGIW	586
26	LTVQARQLLSGIVQQQNNLLRAIEAQQHLLQLTVWGIKQLQARVLAVERYLKDQQLLGIW	586
27	LTVQARQLLSGIVQQQNNLLRAIEAQQHLLQLTVWGIKQLQARVLAVERYLKDQQLLGIW	586
28	LTVQARQLLSGIVQQQNNLLRAIEAQQHLLQLTVWGIKQLQARVLAVERYLKDQQLLGIW	586
29	LTVQARQLLSGIVQQQNNLLRAIEAQQHLLQLTVWGIKQLQARVLAVERYLKDQQLLGIW	586
30	LTVQARQLLSGIVQQQNNLLRAIEAQQHLLQLTVWGIKQLQARVLAVERYLKDQQLLGIW	586
31	LTVQARQLLSGIVQQQNNLLRAIEAQQHLLQLTVWGIKQLQARVLAVERYLKDQQLLGIW	586
32	LTVQARQLLSGIVQQQNNLLRAIEAQQHLLQLTVWGIKQLQARVLAVERYLKDQQLLGIW	586
33	LTVQARQLLSGIVQQQNNLLRAIEAQQHLLQLTVWGIKQLQARVLAVERYLKDQQLLGIW	586
34	LTVQARQLLSGIVQQQNNLLRAIEAQQHLLQLTVWGIKQLQARVLAVERYLKDQQLLGIW	586
35	LTVQARQLLSGIVQQQNNLLRAIEAQQHLLQLTVWGIKQLQARVLAVERYLKDQQLLGIW	586
36	LTVQARQLLSGIVQQQNNLLRAIEAQQHLWQLTVWGIKQLQARVLAVERYLKDQQLLGIW	586
37	LTVQARQLLSGIVQQQNNLLRAIEAQQHLLQLTVWGIKQLQARVLAVERYLKDQQLLGIW	586
38	LTVQARQLLSGIVQQQNNLLRAIEAQQHLLQLTVWGIKQLQARVLAVERYLKDQQLLGIW	584
39	LTVQARQLLSGIVQQQNNLLRAIEAQQHLLQLTVWGIKQLQARVLAVERYLKDQQLLGIW	586
40	LTVQARQLLSGIVQQQNNLLRAIEAQQHLLQLTVWGIKQLQARVLAVERYLKDQQLLGIW	586
41	LTVQARQLLSGIVQQQNNLLRAIEAQQHLLQLTVWGIKQLQARVLAVERYLKDQQLLGIW	586
42	LTVQARQLLSGIVQQQNNLLRAIEAQQHLLQLTVWGIKQLQARVLAVERYLKDQQLLGIW	586
43	LTVQARQLLSGIVQQQNNLLRAIEAQQHLLQLTVWGIKQLQARVLAVERYLKDQQLLGIW	586
44	LTVQARQLLSGIVQQQNNLLRAIEAQQHLLQLTVWGIKQLQARVLAVERYLKDQQLLGIW	586
45	LTVQARQLLSGIVQQQNNLLRAIEAQQHLLQLTVWGIKQLQARVLAVERYLKDQQLLGIW	586
46	LTVQARQLLSGIVQQQNNLLRAIEAQQHLLQLTVWGIKQLQARVLAVERYLKDQQLLGIW	586
47	LTVQARQLLSGIVQQQNNLLRAIEAQQHLLQLTVWGIKQLQARVLAVERYLKDQQLLGIW	586
48	LTVQARQLLSGIVQQQNNLLRAIEAQQHLLQLTVWGIKQLQARVLAVERYLKDQQLLGIW	586
49	LTVQARQLLSGIVQQQNNLLRAIEAQQHLLQLTVWGIKQLQARVLAVERYLKDQQLLGIW	586
50	LTVQARQLLSGIVQQQNNLLRAIEAQQHLLQLTVWGIKQLQARVLAVERYLKDQQLLGIW	586
51	LTVQARQLLSGIVQQQNNLLRAIEAQQHLLQLTVWGIKQLQARVLAVERYLKDQQLLGIW	586
52	LTVQARQLLSGIVQQQNNLLRAIEAQQHLLQLTVWGIKQLQARVLAVERYLKDQQLLGIW	586
53	LTVQARQLLSGIVQQQNNLLRAIEAQQHLLQLTVWGIKQLQARVLAVERYLKDQQLLGIW	586
54	LTVQARQLLSGIVQQQNNLLRAIEAQQHLLQLTVWGIKQLQARVLAVERYLKDQQLLGIW	586
55	LTVQARQLLSGIVQQQNNLLRAIEAQQHLLQLTVWGIKQLQARVLAVERYLKDQQLLGIW	586

56	LTVQARQLLSGIVQQQNNLLRAIEAQQHLLQLTVWGIKQLQARVLAVERYLKDQQLLGIW	586
57	LTVQARQLLSGIVQQQNNLLRAIEAQQHLLQLTVWGIKQLQARVLAVERYLKDQQLLGIW	586
58	LTVQARQLLSGIVQQQNNLLRAIEAQQHLLQLTVWGIKQLQARVLAVERYLKDQQLLGIW	586
59	LTVQARQLLSGIVQQQNNLLRAIEAQQHLLQLTVWGIKQLQARVLAVERYLKDQQLLGIW	584
60	LTVQARQLLSGIVQQQNNLLRAIEAQQHLLQLTVWGIKQLQARVLAVERYLKDQQLLGIW	586
61	LTVQARQLLSGIVQQQNNLLRAIEAQQHLLQLTVWGIKQLQARVLAVERYLKDQQLLGIW	586
62	LTVQARQLLSGIVQQQNNLLRAIEAQQHLLQLTVWGIKQLQARVLAVERYLKDQQLLGIW	586
63	LTVQARQLLSGIVQQQNNLLRAIEAQQHLLQLTVWGIKQLQARVLAVERYLKDQQLLGIW	586
64	LTVQARQLLSGIVQQQNNLLRAIEAQQHLLQLTVWGIKQLQARVLAVERYLKDQQLLGIW	586
65	LTVQARQLLSGIVQQQNNLLRAIEAQQHLLQLTVWGIKQLQARVLAVERYLKDQQLLGIW	586
66	LTVQARQLLSGIVQQQNNLLRAIEAQQHLLQLTVWGIKQLQARVLAVERYLKDQQLLGIW	586
67	LTVQARQLLSGIVQQQNNLLRAIEAQQHLLQLTVWGIKQLQARVLAVERYLKDQQLLGIW	584
68	LTVQARQLLSGIVQQQNNLLRAIEAQQHLLQLTVWGIKQLQARVLAVERYLKDQQLLGIW	586

GCSGKLICTTTVPWNTSWSNKSLEQIWDNMTWMEWEREIDNYTGYIYQLIEESQNQQEKN

1	GCSGKLICTTTVPWNTSWSNKSLEQIWDNMTWMEWEREIDNYTGYIYQLIEESQNQQEKN	646
2	GCSGKLICTTTVPWNTSWSNKSLEQIWDNMTWMEWEREIDNYTGYIYQLIEESQNQQEKN	645
3	GCSGKLICTTTVPWNTSWSNKSLEQIWDNMTWMEWEREIDNYTGYIYQLIEESQNQQEKN	646
4	GCSGKLICTTTVPWNTSWSNKSLEQIWDNMTWMEWEREIDNYTGYIYQLIEESQNQQEKN	646
5	GCSGKLICTTTVPWNTSWSNKSLEQIWDNMTWMEWEREIDNYTGYIYQLIEESQNQQEKN	646
6	GCSGKLICTTTVPWNTSWSNKSLEQIWDNMTWMEWEREIDNYTGYIYQLIEESQNQQEKN	646
7	GCSGKLICTTTVPWNTSWSNKSLEQIWDNMTWMEWEREIDNYTGYIYQLIEESQNQQEKN	646
8	GCSGKLICTTTVPWNTSWSNKSLEQIWDNMTWMEWEREIDNYTGYIYQLIEESQNQQEKN	646
9	GCSGKLICTTTVPWNTSWSNKSLEQIWDNMTWMEWEREIDNYTGYIYQLIEESQNQQEKN	645
10	GCSGKLICTTTVPWNTSWSNKSLEQIWDNMTWMEWEREIDNYTGYIYQLIEESQNQQEKN	646
11	GCSGKLICTTTVPWNTSWSNKSLEQIWDNMTWMEWEREIDNYTGYIYQLIEESQNQQEKN	646
12	GCSGKLICTTTVPWNTSWSNKSLEQIWDNMTWMEWEREIDNYTGYIYQLIEESQNQQEKN	646
13	GCSGKLICTTTVPWNTSWSNKSLEQIWDNMTWMEWEREIDNYTGYIYQLIEESQNQQEKN	646
14	GCSGKLICTTTVPWNTSWSNKSLEQIWDNMTWMEWEREIDNYTGYIYQLIEESQNQQEKN	644
15	GCSGKLICTTTVPWNTSWSNKSLEQIWDNMTWMEWEREIDNYTGYIYQLIEKSQNQQEKN	614
16	GCSGKLICTTTVPWNTSWSNKSLEQIWDNMTWMEWEREIDNYTGYIYQLIEESQNQQEKN	646
17	GCSGKLICTTTVPWNTSWSNKSLEQIWDNMTWMEWEREIDNYTGYIYQLIEESQNQQEKN	646
18	GCSGKLICTTTVPWNTSWSNKSLEQIWDNMTWMEWEREIDNYTGYIYQLIEESQNQQEKN	646
19	GCSGKLICTTTVPWNTSWSNKSLEQIWDNMTWMEWEREIDNYTGYIYQLIEESQNQQEKN	646
20	GCSGKLICTTTVPWNTSWSNKSLEQIWDNMTWMEWEREIDNYTGYIYQLIEESQNQQEKN	644
21	GCSGKLICTTTVPWNTSWSNKSLEQIWDNMTWMEWEREIDNYTGYIYQLIEESQNQQEKN	646
22	GCSGKLICTTTVPWNTSWSNKSLEQIWDNMTWMEWEREIDNYTGYIYQLIEESQNQQEKN	629
23	GCSGKLICTTTVPWNTSWSNKSLEQIWDNMTWMEWEREIDNYTGYIYQLIEESQNQQEKN	646
24	GCSGKLICTTTVPWNTSWSNKSLEQIWDNMTWMEWEREIDNYTGYIYQLIEESQNQQEKN	645
25	GCSGKLICTTTVPWNTSWSNKSLEQIWDNMTWMEWEREIDNYTGYIYQLIEESQNQQEKN	646
26	GCSGKLICTTTVPWNTSWSNKSLEQIWDNMTWMEWEREIDNYTGYIYQLIEESQNQQEKN	646
27	GCSGKLICTTTVPWNTSWSNKSLEQIWDNMTWMEWEREIDNYTGYIYQLIEESQNQQEKN	646
28	GCSGKLICTTTVPWNTSWSNKSLEQIWDNMTWMEWEREIDNYTGYIYQLIEESQNQQEKN	646
29	GCSGKLICTTTVPWNTSWSNKSLEQIWDNMTWMEWEREIDNYTGYIYQLIEESQNQQEKN	646
30	GCSGKLICTTTVPWNTSWSNKSLEQIWDNMTWMEWEREIDNYTGYIYQLIEESQNQQEKN	646
31	GCSGKLICTTTVPWNTSWSNKSLEQIWDNMTWMEWEREIDNYTGYIYQLIEESQNQQEKN	646
32	GCSGKLICTTTVPWNTSWSNKSLEQIWDNMTWMEWEREIDNYTGYIYQLIEESQNQQEKN	646
33	GCSGKLICTTTVPWNTSWSNKSLEQIWDNMTWMEWEREIDNYTGYIYQLIEESQNQQEKN	646
34	GCSGKLICTTTVPWNTSWSNKSLEQIWDNMTWMEWEREIDNYTGYIYQLIEESQNQQEKN	646
35	GCSGKLICTTTVPWNTSWSNKSLEQIWDNMTWMEWEREIDNYTGYIYQLIEESQNQQEKN	646
36	GCSGKLICTTTVPWNTSWSNKSLEQIWDNMTWMEWEREIDNYTGYIYQLIEESQNQQEKN	646
37	GCSGKLICTTTVPWNTSWSNKSLEQIWDNMTWMEWEREIDNYTGYIYQLIEESQNQQEKN	646

38	GCSGKLICTTTVPWNTSWSNKSLEQIWDNMTWMEWEREIDNYTGYIYQLIEESQNQQEKN	644
39	GCSGKLICTTTVPWNTSWSNKSLEQIWDNMTWMEWEREIDNYTGYIYQLIEESQNQQEKN	646
40	GCSGKLICTTTVPWNTSWSNKSLEQIWDNMTWMEWEREIDNYTGYIYQLIEESQNQQEKN	646
41	GCSGKLICTTTVPWNTSWSNKSLEQIWDNMTWMEWEREIDNYTGYIYQLIEESQNQQEKN	646
42	GCSGKLICTTTVPWNTSWSNKSLEQIWDNMTWMEWEREIDNYTGYIYQLIEESQNQQEKN	646
43	GCSGKLICTTTVPWNTSWSNKSLEQIWDNMTWMEWEREIDNYTGYIYQLIEESQNQQEKN	646
44	GCSGKLICTTTVPWNTSWSNKSLEQIWDNMTWMEWEREIDNYTGYIYQLIEESQNQQEKN	646
45	GCSGKLICTTTVPWNTSWSNKSLEQIWDNMTWMEWEREIDNYTGYIYQLIEESQNQQEKN	646
46	GCSGKLICTTTVPWNTSWSNKSLEQIWDNMTWMEWEREIDNYTGYIYQLIEESQNQQEKN	646
47	GCSGKLICTTTVPWNTSWSNKSLEQIWDNMTWMEWEREIDNYTGYIYQLIEESQNQQEKN	646
48	GCSGKLICTTTVPWNTSWSNKSLEQIWDNMTWMEWEREIDNYTGYIYQLIEESQNQQEKN	646
49	GCSGKLICTTTVPWNTSWSNKSLEQIWDNMTWMEWEREIDNYTGYIYQLIEESQNQQEKN	646
50	GCSGKLICTTTVPWNTSWSNKSLEQIWDNMTWMEWEREIDNYTGYIYQLIEESQNQQEKN	646
51	GCSGKLICTTTVPWNTSWSNKSLEQIWDNMTWMEWEREIDNYTGYIYQLIEESQNQQEKN	646
52	GCSGKLICTTTVPWNTSWSNKSLEQIWDNMTWMEWEREIDNYTGYIYQLIEESQNQQEKN	646
53	GCSGKLICTTTVPWNTSWSNKSLEQIWDNMTWMEWEREIDNYTGYIYQLIEESQNQQEKN	646
54	GCSGKLICTTTVPWNTSWSNKSLEQIWDNMTWMEWEREIDNYTGYIYQLIEESQNQQEKN	646
55	GCSGKLICTTTVPWNTSWSNKSLEQIWDNMTWMEWEREIDNYTGYIYQLIEESQNQQEKN	646
56	GCSGKLICTTTVPWNTSWSNKSLEQIWDNMTWMEWEREIDNYTGYIYQLIEESQNQQEKN	646
57	GCSGKLICTTTVPWNTSWSNKSLEQIWDNMTWMEWEREIDNYTGYIYQLIEESQNQQEKN	646
58	GCSGKLICTTTVPWNTSWSNKSLEQIWDNMTWMEWEREIDNYTGYIYQLIEESQNQQEKN	646
59	GCSGKLICTTTVPWNTSWSNKSLEQIWDNMTWMEWEREIDNYTGYIYQLIEESQNQQEKN	644
60	GCSGKLICTTTVPWNTSWSNKSLEQIWDNMTWMEWEREIDNYTGYIYQLIEESQNQQEKN	646
61	GCSGKLICTTTVPWNTSWSNKSLEQIWDNMTWMEWEREIDNYTGYIYQLIEESQNQQEKN	646
62	GCSGKLICTTTVPWNTSWSNKSLEQIWDNMTWMEWEREIDNYTGYIYQLIEESQNQQEKN	646
63	GCSGKLICTTTVPWNTSWSNKSLEQIWDNMTWMEWEREIDNYTGYIYQLIEESQNQQEKN	646
64	GCSGKLICTTTVPWNTSWSNKSLEQIWDNMTWMEWEREIDNYTGYIYQLIEESQNQQEKN	646
65	GCSGKLICTTTVPWNTSWSNKSLEQIWDNMTWMEWEREIDNYTGYIYQLIEESQNQQEKN	646
66	GCSGKLICTTTVPWNTSWSNKSLEQIWDNMTWMEWEREIDNYTGYIYQLIEESQNQQEKN	646
67	GCSGKLICTTTVPWNTSWSNKSLEQIWDNMTWMEWEREIDNYTGYIYQLIEESQNQQEKN	644
68	GCSGKLICTTTVPWNTSWSNKSLEQIWDNMTWMEWEREIDNYTGYIYQLIEESQNQQEKN	646

EQELLALDKWASLWNWFDITNWLWYIKIFIMIVGGLIGLRIVFTVLSIVNRVRQGYSPLS

1	EQELLALDKWASLWNWFDITNWLWYIKIFIMIVGGLIGLRIVFTVLSIVNRVRQGYSPLS	706
2	EQELLALDKWASLWNWFDITNWLWYIKIFIMIVGGLIGLRIVFTVLSIVNRVRQGYSPLS	705
3	EQELLALDKWASLWNWFDITNWLWYIKIFIMIVGGLIGLRIVFTVLSIVNRVRQGYSPLS	706
4	EQELLALDKWASLWNWFDITNWLWYIKIFIMIVGGLIGLRIVFTVLSIVNRVRQGYSPLS	706
5	EQELLALDKWASLWNWFDITNWLWYIKIFIMMVGGLIGLRIVFTVLSIVNRVRQGYSPLS	706
6	EQELLALDKWASLWNWFDITNWLWYIKIFIMIVGGLIGLRIVFTVLSIVNRVRQGYSPLS	706
7	EQELLALDKWASLWNWFDITNWLWYIKIFIMIVGGLIGLRIVFTVLSIVNRVRQGYSPLS	706
8	EQELLALDKWASLWNWFDITNWLWYIKIFIMIVGGLIGLRIVFTVLSIVNRVRQGYSPLS	706
9	EQELLALDKWASLWNWFDITNWLWYIKIFIMIVGGLIGLRIVFTVLSIVNRVRQGYSPLS	705
10	EQELLALDKWASLWNWFDITNWLWYIKIFIMIVGGLIGLRIVFTVLSIVNRVRQGYSPLS	706
11	EQELLALDKWASLWNWFDITNWLWYIKIFIMIVGGLIGLRIVFTVLSIVNRVRQGYSPLS	706
12	EQELLALDKWASLWNWFDITNWLWYIKIFIMIVGGLIGLRIVFTVLSIVNRVRQGYSPLS	706
13	EQELLALDKWASLWNWFDITNWLWYIKIFIMIVGGLIGLRIVFTVLSIVNRVRQGYSPLS	706
14	EQELLALDKWASLWNWFDITNWLWYIKIFIMIVGGLIGLRIVFTVLSIVNRVRQGYSPLS	704
15	EQELLALDKWASLWNWFDITNWLWYIKIFIMIVGGLIGLRIVFTVLSIVNRVRQGYSPLS	674
16	EQELLALDKWASLWNWFDITNWLWYIKIFIMIVGGLIGLRIVFTVLSIVNRVRQGYSPLS	706
17	EQELLALDKWASLWNWFDITNWLWYIKIFIMIVGGLIGLRIVFTVLSIVNRVRQGYSPLS	706
18	EQELLALDEWASLWNWFDITNWLWYIKIFIMIVGGLIGLRIVFTVLSIVNRVRQGYSPLS	706
19	EQELLALDKWASLWNWFDITNWLWYIKIFIMIVGGLIGLRIVFTVLSIVNRVRQGYSPLS	706

20	EQELLALDKWASLWNWFDITNWLWYIKIFIMIVGGLIGLRIVFTVLSIVNRVRQGYSPLS	704
21	EQELLALDKWASLWNWFDITNWLWYIKIFIMIVGGLIGLRIVFTVLSIVNRVRQGYSPLS	706
22	EQELLALDKWASLWNWFDITNWLWYIKIFIMIVGGLIGLRIVFTVLSIVNRVRQGYSPLS	689
23	EQELLALDKWASLWNWFDITNWLWYIKIFIMIVGGLIGLRIVFTVLSIVNRVRQGYSPLS	706
24	EQELLALDKWASLWNWFDITNWLWYIKIFIMIVGGLIGLRIVFTVLSIVNRVRQGYSPLS	705
25	EQELLALDKWASLWNWFDITNWLWYIKIFIMIVGGLIGLRIVFTVLSIVNRVRQGYSPLS	706
26	EQELLALDKWASLWNWFDITNWLWYIKIFIMIVGGLIGLRIVFTVLSIVNRVRQGYSPLS	706
27	EQELLALDKWASLWNWFDITNWLWYIKIFIMIVGGLIGLRIVFTVLSIVNRVRQGYSPLS	706
28	EQELLALDKWASLWNWFDITNWLWYIKIFIMMVGGLIGLRIVFTVLSIVNRVRQGYSPLS	706
29	EQELLALDKWASLWNWFDITNWLWYIKIFIMIVGGLIGLRIVFTVLSIVNRVRQGYSPLS	706
30	EQELLALDKWASLWNWFDITNWLWYIKIFIMIVGGLIGLRIVFTVLSIVNRVRQGYSPLS	706
31	EQELLALDKWASLWNWFDITNWLWYIKIFIMIVGGLIGLRIVFTVLSIVNRVRQGYSPLS	706
32	EQELLALDKWASLWNWFDITNWLWYIKIFIMIVGGLIGLRIVFTVLSIVNRVRQGYSPLS	706
33	EQELLALDKWASLWNWFDITNWLWYIKIFIMIVGGLIGLRIVFTVLSIVNRVRQGYSPLS	706
34	EQELLALDKWASLWNWFDITNWLWYIKIFIMIVGGLIGLRIVFTVLSIVNRVRQGYSPLS	706
35	EQELLALDKWASLWNWFDITNWLWYIKIFIMIVGGLIGLRIVFTVLSIVNRVRQGYSPLS	706
36	EQELLALDKWASLWNWFDITNWLWYIKIFIMIVGGLIGLRIVFTVLSIVNRVRQGYSPLS	706
37	EQELLALDKWASLWNWFDITNWLWYIKIFIMIVGGLIGLRVVFTVLSIVNRVRQGYSPLS	706
38	EQELLALDKWASLWNWFDITNWLWYIKIFIMIVGGLIGLRIVFTVLSIVNRVRQGYSPLS	704
39	EQELLALDKWASLWNWFDITNWLWYIKIFIMIVGGLIGLRIVFTVLSIVNRVRQGYSPLS	706
40	EQELLALDKWASLWNWFDITNWLWYIKIFIMIVGGLIGLRIVFTVLSIVNRVRQGYSPLS	706
41	EQELLALDKWASLWNWFDITNWLWYIKIFIMIVGGLIGLRIVFTVLSIVNRVRQGYSPLS	706
42	EQELLALDKWASLWNWFDITNWLWYIKIFIMIVGGLIGLRIVFTVLSIVNRVRQGYSPLS	706
43	EQELLALDKWASLWNWFDITNWLWYIKIFIMIVGGLIGLRIVFTVLSIVNRVRQGYSPLS	706
44	EQELLALDKWASLWNWFDITNWLWYIKIFIMIVGGLIGLRIVFTVLSIVNRVRQGYSPLS	706
45	EQELLALDKWASLWNWFDITNWLWYIKIFIMIVGGLIGLRIVFTVLSIVNRVRQGYSPLS	706
46	EQELLALDKWASLWNWFDITNWLWYIKIFIMIVGGLIGLRIVFTVLSIVNRVRQGYSPLS	706
47	EQELLALDKWASLWNWFDITNWLWYIKIFIMIVGGLIGLRIVFTVLSIVNRVRQGYSPLS	706
48	EQELLALDKWASLWNWFDITNWLWYIKIFIMIVGGLIGLRIVFTVLSIVNRVRQGYSPLS	706
49	EQELLALDKWASLWNWFDITNWLWYIKIFIMIVGGLIGLRIVFTVLSIVNRVRQGYSPLS	706
50	EQELLALDKWASLWNWFDITDWLWYIKIFIMIVGGLIGLRIVFTVLSIVNRVRQGYSPLS	706
51	EQELLALDKWASLWNWFDITNWLWYIKIFIMIVGGLIGLRIVFTVLSIVNRVRQGYSPLS	706
52	EQELLALDKWASLWNWFDITNWLWYIKIFIMIVGGLIGLRIVFTVLSIVNRVRQGYSPLS	706
53	EQELLALDKWASLWNWFDITNWLWYIKIFIMIVGGLIGLRIVFTVLSIVNRVRQGYSPLS	706
54	EQELLALDKWASLWNWFDITNWLWYIKIFIMIVGGLIGLRIVFTVLSIVNRVRQGYSPLS	706
55	EQELLALDKWASLWNWFDITNWLWYIKIFIMIVGGLIGLRIVFTVLSIVNRVRQGYSPLS	706
56	EQELLALDKWASLWNWFDITNWLWYIKIFIMIVGGLIGLRIVFTVLSIVNRVRQGYSPLS	706
57	EQELLALDKWASLWNWFDITNWLWYIKIFIMIVGGLIGLRIVFTVLSIVNRVRQGYSPLS	706
58	EQELLALDKWASLWNWFDITNWLWYIKIFIMIVGGLIGLRIVFTVLSIVNRVRQGYSPLS	706
59	EQELLALDKWASLWNWFDITNWLWYIKIFIMIVGGLLGLRIVFTVLSIVNRVRQGYSPLS	704
60	EQELLALDKWASLWNWFDITNWLWYIKIFIMIVGGLIGLRIVFTVLSIVNRVRQGYSPLS	706
61	EQELLALDKWASLWNWFDITNWLWYIKIFIMIVGGLIGLRIVFTVLSIVNRVRQGYSPLS	706
62	EQELLALDKWASLWNWFDITNWLWYIKIFIMIVGGLIGLRIVFTVLSIVNRVRQGYSPLS	706
63	EQELLALDKWASLWNWFDITNWLWYIKIFIMIVGGLIGLRIVFTVLSIVNRVRQGYSPLS	706
64	EQELLALDKWASLWNWFDITNWLWYIKIFIMIVGGLIGLRIVFTVLSIVNRVRQGYSPLS	706
65	EQELLALDKWASLWNWFDITNWLWYIKIFIMIVGGLIGLRIVFTVLSIVNRVRQGYSPLS	706
66	EQELLALDKWASLWN-FDITNWLWYIKIFIMIVGGLIGLRIVFTVLSIVNRVRQGYSPLS	705
67	EQELLALDKWASLWNWFDITNWLWYIKIFIMIVGGLIGLRIVFTVLSIVNRVRQGYSPLS	704
68	EQELLALDKWASLWNWFDITNWLWYIKIFIMIVGGLIGLRIVFTVLSIVNRVRQGYSPLS	706

FQTHLPAQRGPDRPEGIGEEGGERDRDRSDPLVNGFLALIWSDLRSLCLFSYHRLRDLLL

1	FQTHLPAQRGPDRPEGIGEEGGERDRDRSDPLVNGFLALIWSDLRSLCLFSYHRLRDLLL	766

2	FQTHLPAQRGPDRPEGIGEEGGERDRDRSDPLVNGFLALIWSDLRSLCLFSYHRLRDLLL	765
3	FQTHLPAQRGPDRPEGIGEEGGERDRDKSDPLVNGFLALIWSDLRSLCLFSYHRLRDLLL	766
4	FQTHLPAQRGPDRPEGIGEEGGERDRDRSDPLVNGFLALIWSDLRSLCLFSYHRLRDLLL	766
5	FQTHLPAQRGPDRPEGIGEEGGERDRDKSDPLVNGFLALIWSDLRSLCLFSYHRLRDLLL	766
6	FQTHLPAQRGPDRPEGIGEEGGERDRDRSDPLVNGFLALIWSDLRSLCLFSYHRLRDLLL	766
7	FQTHLPAQRGPDRPEGIGEEGGERDRDRSDPLVNGFLALIWSDLRSLCLFSYHRLRDLLL	766
8	FQTHLPAQRGPDRPEGIGEEGGERDRDRSDPLVNGFLALIWSDLRSLCLFSYHRLRDLLL	766
9	FQTHLPAQRGPDRPEGIGEEGGERDRDRSDPLVNGFLALIWSDLRSLCLFSYHRLRDLLL	765
10	FQTHLPAQRGPDRPEGIGEEGGERDRDRSDPLVNGFLALIWSDLRSLCLFSYHRLRDLLL	766
11	FQTHLPAQRGPDRPEGIGEEGGERDRDRSDPLVNGFLALIWSDLRSLCLFSYHRLRDLLL	766
12	FQTHLPAQRGPDRPEGIGEEGGERDRDRSDPLVNGFLALIWSDLRSLCLFSYHRLRDLLL	766
13	FQTHLPAQRGPDRPEGIGEEGGERDRDRSDPLVNGFLALIWSDLRSLCLFSYHRLKDLLL	766
14	FQTHLPAQRGPDRPEGIGEEGGERDRDRSDPLVNGFLALIWSDLRSLCLFSYHRLRDLLL	764
15	FQTHLPAQRGPDRPEGIGEEGGERDRDKSDPLVNGFLALIWSDLRSLCLFSYHRLRDLLL	734
16	FQTHLPAQRGPDRPEGIGEEGGERDRDRSDPLVNGFLALIWSDLRSLCLFSYHRLRDLLL	766
17	FQTHLPAQRGPDRPEGIGEEGGERDRDKSDPLVNGFLALIWSDLRSLCLFSYHRLRDLLL	766
18	FQTHLPAQRGPDRPEGIGEEGGERDRDRSDPLVNGFLALIWSDLRSLCLFSYHRLRDLLL	766
19	FQTHLPAQRGPDRPEGIGEEGGERDRDRSDPLVNGFLALIWSDLRSLCLFSYHRLRDLLL	766
20	FQTHLPAQRGPDRPEGIGEEGGERDRDRSDPLVNGFLALIWSDLRSLCLFSYHRLRDLLL	764
21	FQTHLPAQRGPDRPEGIGEEGGERDRDRSDPLVNGFLALIWSDLRSLCLFSYHRLRDLLL	766
22	FQTHLPAQRGPDRPEGIGEEGGERDRDRSDPLVNGFLALIWSDLRSLCLFSYHRLRDLLL	749
23	FQTHLPAQRGPDRPEGIGEEGGERDRDRSDPLVNGFLALIWSDLRSLCLFSYHRLRDLLL	766
24	FQTHLPAQRGPDRPEGIGEEGGERDRDRSDPLVNGFLALIWSDLRSLCLFSYHRLRDLLL	765
25	FQTHLPAQRGPDRPEGIGEEGGERDRDRSDPLVNGFLALIWSDLRSLCLFSYHRLRDLLL	766
26	FQTHLPAQRGPDRPEGIGEEGGERDRDRSDPLVNGFLALIWSDLRSLCLFSYHRLRDLLL	766
27	FQTHLPAQRGPDRPEGIGEEGGERDRDKSDPLVNGFLALIWSDLRSLCLFSYHRLRDLLL	766
28	FQTHLPAQRGPDRPEGIGEEGGERDRDKSDPLVNGFLALIWSDLRSLCLFSCHRLRDLLL	766
29	FQTHLPAQRGPDRPEGIGEEGGERDRDKSDPLVNGFLALIWSDLRSLCLFSYHRLRDLLL	766
30	FQTHLPAQRGPDRPEGIGEEGGERDRDKSDPLVNGFLALIWSDLRSLCLFSYHRLRDLLL	766
31	FQTHLPAQRGPDRPEGIGEEGGERDRDRSDPLVNGFLALIWSDLRSLCLFSYHRLRDLLL	766
32	FQTHLPAQRGPDRPEGIGEEGGERDRDRSDPLVNGFLALIWSDLRSLCLFSYHRLRDLLL	766
33	FQTHLPAQRGPDRPEGIGEEGGERDRDRSDPLVNGFLALIWSDLRSLCLFSYHSLRDLLL	766
34	FQTHLPAQRGPDRPEGIGEEGGERDRDKSDPLVNGFLALIWSDLRSLCLFSYHRLRDLLL	766
35	FQTHLPAQRGPDRPEGIGEEGGERDRDRSDPLVNGFLALIWSDLRSLCLFSYHRLRDLLL	766
36	FQTHLPAQRGPDRPEGIGEEGGERDRDRSDPLVNGFLALIWSDLRSLCLFSYHRLRDLLL	766
37	FQTHLPAQRGPDRPEGIGEEGGERDRDRSDPLVNGFLALIWSDLRSLCLFSYHRLRDLLL	766
38	FQTHLPAQRGPDRPEGIGEEGGERDRDRSDPLVNGFLALIWSDLRSLCLFSYHRLRDLLL	764
39	FQTHLPAQRGPDRPEGIGEEGGERDRDRSDPLVNGFLALIWSDLRSLCLFSYHRLRDLLL	766
40	FQTHLPAQRGPDRPEGIGEEGGERDRDKSDPLVNGFLALIWSDLRSLCLFSYHRLRDLLL	766
41	FQTHLPAQRGPDRPEGIGEEGGERDRDKSDPLVNGFLALIWSDLRSLCLFSYHRLRDLLL	766
42	FQTHLPAQRGPDRPEGIGEEGGERDRDRSDPLVNGFLTLIWSDLRSLCLFSYHRLRDLLL	766
43	FQTHLPAQRGPDRPEGIGEEGGERDRDRSDPLVNGFLTLIWSDLRSLCLFSYHRLRDLLL	766
44	FQTHLPAQRGPDRPEGIGEEGGERDRDRSDPLVNGFLALIWSDLRSLCLFSYHRLRDLLL	766
45	FQTHLPAQRGPDRPEGIGEEGGERDRDRSDPLVNGFLTLIWSDLRSLCLFSYHRLRDLLL	766
46	FQTHLPAQRGPDRPEGIGEEGGERDRDKSDPLVNGFLALIWSDLRSLCLFSYHRLRDLLL	766
47	FQTHLPAQRGPDRPEGIGEEGGERDRDRSDPLVNGFLALIWSDLRSLCLFSYHRLRDLLL	766
48	FQTHLPAQRGPDRPEGIGEEGGERDRDRSDPLVNGFLTLIWSDLRSLCLFSYHRLRDLLL	766
49	FQTHLPAQRGPDRPEGIGEEGGERDRDRSDPLVNGFLALIWSDLRSLCLFSYHRLRDLLL	766
50	FQTHLPAQRGPDRPEGIGEEGGERDRDRSDPLVNGFLALIWSDLRSLCLFSYHRLRDLLL	766
51	FQTHLPAQRGPDRPEGIGEEGGERDRDRSDPLVNGFLALIWSDLRSLCLFSYHRLRDLLL	766
52	FQTHLPAQRGPDRPEGIGEEGGERDRDRSDPLVNGFLTLIWSDLRSLCLFSYHRLRDLLL	766
53	FQTHLPAQRGPDRPEGIGEEGGERDRDRSDPLVNGFLALIWSDLRSLCLFSYHRLRDLLL	766
54	FQTHLPAQRGPDRPEGIGEEGGERDRDRSDPLVNGFLALIWSDLRSLCLFSYHRLRDLLL	766
55	FQTHLPAQRGPDRPEGIGEEGGERDRNRSDPLVNGFLALIWSDLRSLCLFSYHRLRDLLL	766
56	FQTHLPAQRGPDRPEGIGEEGGERDRDRSDPLVNGFLTLIWSDLRSLCLFSYHRLRDLLL	766

```
57  FQTHLPAQRGPDRPEGIGEEGGERDRDRSDPLVNGFLALIWSDLRSLCLFSYHRLRDLLL  766
58  FQTHLPAQRGPDRPERIGEEGGERDRDRSDPLVNGFLALIWSDLRSLCLFSYHRLRDLLL  766
59  FQTHLPAQRGPDRPEGIGEEGGERDRDRSDPLVNGFLALIWSDLRSLCLFSYHRLRDLLL  764
60  FQTHLPAQRGPDRPEGIGEEGGERDRDRSDPLVNGFLTLIWSDLRSLCLFSYHRLRDLLL  766
61  FQTHLPAQRGPDRPEGIGEEGGERDRDRSNPLVNGFLALIWSDLRSLCLFSYHRLRDLLL  766
62  FQTHLPAQRGPDRPEGIGEEGGERDRDRSDPLVNGFLALIWSDLRSLCLFSYHRLRDLLL  766
63  FQTHLPAQRGPDRPEGIGEEGGERDRDRSDPLVNGFLALIWSDLRSLCLFSYHRLRDLLL  766
64  FQTHLPAQRGPDRPEGIGEEGGERDRDRSDPLVNGFLTLIWSDLRSLCLFSYHRLRDLLL  766
65  FQTHLPAQRGPDRPEGIGEEGGERDRDRSDPLVNGFLTLIWSDLRSLCLFSYHRLRDLLL  766
66  FQTHLPAQRGPDRPEGIGEEGGERDRDRSDPLVNGFLTLIWSDLRSLCLFSYHRLRDLLL  765
67  FQTHLPAQRGPDRPEGIGEEGGERDRDRSDPLVNGFLALIWSDLRSLCLFSYHRLRDLLL  764
68  FQTHLPAQRGPDRPEGIGEEGGERDRDRSDPLVNGFLTLIWSDLRSLCLFSYHRLRDLLL  766

    IVTRIVELLGR-------RGWEVLKYWWNLLQYWSQELKNSAVSLLNATAIAVAEGTDRV

1   IVTRIVELLGR-------RGWEVLKYWWNLLQYWSQELKNSAVSLLNATAIAVAEGTDRV  819
2   IVTRIVELLGR-------RGWEVLKYWWNLLQYWSQELKNSAVSLLNATAIAVAEGTDRV  818
3   IVTRIVELLGR-------RGWEVLKYWWNLLQYWSQELKNSAVSLLNATAIAVAEGTDRV  819
4   IVTRIVELLGR-------RGWEVLKYWWNLLQYWSQELKNSAVSLLNATAIAVAEGTDRV  819
5   IVTRIVELLGR-------RGWEVLKYWWNLLQYWSQELKNSAVSLLNATAIAVAEGTDRV  819
6   IVTRIVELLGR-------RGWEVLKYWWNLLQYWSQELKNSAVSLLNATAIAVAEGTDRV  819
7   IVTRIVELLGR-------RGWEVLKYWWNLLQYWSQELKNSAVSLLNATAIAVAEGTDRV  819
8   ---RIVELLGR-------RGWEVLKYWWNLLQYWSQELKNSAVSLLNATAIAVAEGTDRV  816
9   IVTRIVELLGR-------RGWEVLKYWWNLLQYWSQELKNSAVSLLNATAIAVAEGTDRV  818
10  IVTRIVELLGR-------RGWEVLKYWWNLLQYWSQELKNSAVSLLNATAIAVAEGTDRV  819
11  IVTRIVELLGR-------RGWEVLKYWWNLLQYWSQELKNSAVSLLNATAIAVAEGTDRV  819
12  IVTRIVELLGR-------RGWEVLKYWWNLLQYWSQELKNSAVSLLNATAIAVAEGTDRV  819
13  IVTRIVELLGR-------RGWEVLKYWWNLLQYWSQELKNSAVSLLNATAIAVAEGTDRV  819
14  IVTRIVELLGR-------RGWEVLKYWWNLLQYWSQELKNSAVSLLNATAIAVAEGTDRV  817
15  IVTRIVELLGR-------RGWEVLKYWWNLLQYWSQELKNSAVSLLNATAIAVAEGTDRV  787
16  IVTRIVELLGR-------RGWEVLKYWWNLLQYWSQELKNSAVSLLNATAIAVAEGTDRV  819
17  IVTRIVELLGR-------RGWEVLKYWWNLLQYWSQELKNSAVSLLNATAIAVAEGTDRV  819
18  IVTRIVELLGR-------RGWEVLKYWWNLLQYWSQELKNSAVSLLNATAIAVAEGTDRV  819
19  IVTRIVELLGR-------RGWEVLKYWWNLLQYWSQELKNSAVSLLNATAIAVAEGTDRV  819
20  IVTRIVELLGR-------RGWEVLKYWWNLLQYWSQELKNSAVSLLNATAIAVAEGTDRV  817
21  IVTRIVELLGR-------RGWEVLKYWWNLLQYWSQELKNSAVSLLNATAIAVAEGTDRV  819
22  IVTRIVELLGR-------RGWEVLKYWWNLLQYWSQELKNSAVSLLNATAIAVAEGTDRV  802
23  IVTRIVELLGR-------RGWEVLKYWWNLLQYWSQELKNSAVSLLNATAIAVAEGTDRV  819
24  IVTRIVELLGR-------RGWEVLKYWWNLLQYWSQELKNSAVSLLNATAIAVAEGTDRV  818
25  IVTRIVELLGR-------RGWEVLKYWWNLLQYWSQELKNSAVSLLNATAIAVAEGTDRV  819
26  IVTRIVELLGR-------RGWEVLKYWWNLLQYWSQELKNSAVSLLNATAIAVAEGTDRV  819
27  IVTRIVELLGR-------RGWEVLKYWWNLLQYWSQELKNSAVSLLNATAIAVAEGTDRV  819
28  IVTRIVELLGR-------RGWEVLKYWWNLLQYWSQELKNSAVSLLNATAIAVAEGTDRV  819
29  IATRIVELLGR-------RGWEVLKYWWNLLQYWSQELKNSAVSLLNATAIAVAEGTDRV  819
30  IVTRIVELLGR-------RGWEVLKYWWNLLQYWSQELKNSAVSLLNATAIAVAEGTDRV  819
31  IVTRIVELLGR-------RGWEVLKYWWNLLQYWSQELKNSAVSLLNATAIAVAEGTDRV  819
32  IVTRIVELLGR-------RGWEVLKYWWNLLQYWSQELKNSAVSLLNATAIAVAEGTDRV  819
33  IVTRIVELLGR-------RGWEVLKYWWNLLQYWSQELKNSAVSLLNATAIAVAEGTDRV  819
34  IVTRIVELLGR-------RGWEVLKYWWNLLQYWSQELKNSAVSLLNATAIAVAEGTDRV  819
35  IVTRIVELLGR-------RGWEVLKYWWNLLQYWSQELKNSAVSLLNATAIAVAEGTDRV  819
36  IVTRIVELLGR-------RGWEVLKYWWNLLQYWSQELKNSAVSLLNATAIAVAEGTDRV  819
37  IVTRIVELLGR-------RGWEVLKYWWNLLQYWSQELKNSAVSLLNATAIAVAEGTDRV  819
38  IVTRIVELLGR-------RGWEVLKYWWNLLQYWSQELKNSAVSLLNATAIAVAEGTDRV  817
```

```
39  IVTRIVELLGR-------RGWEVLKYWWNLLQYWSQELKNSAVSLLNATAIAVAEGTDRV  819
40  IVTRIVELLGR-------RGWEVLKYWWNLLQYWSQELKNSAVSLLNATAIAVAEGTDRV  819
41  IVTRIVELLGR-------RGWEVLKYWWNLLQYWSQELKNSAVSLLNATAIAVAEGTDRV  819
42  IVTRIVELLGR-------RGWEVLKYWWNLLQYWSQELKNSAVSLLNATAIAVAEGTDRV  819
43  IVTRIVELLGR-------RGWEVLKYWWNLLQYWSQELKNSAVSLLNATAIAVAEGTDRV  819
44  IVTRIVELLGR-------RGWEVLKYWWNLLQYWSQELKNSAVSLLNATAIAVAEGTDRV  819
45  IVTRIVELLGR-------RGWEVLKYWWNLLQYWSQELKNSAVSLLNATAIAVAEGTDRV  819
46  IVTRIVELLGR-------RGWEVLKYWWNLLQYWSQELKNSAVSLLNATAIAVAEGTDRV  819
47  IVTRIVELLGR-------RGWEVLKYWWNLLQYWSQELKNSAVSLLNATAIAVAEGTDRV  819
48  IVTRIVELLGR-------RGWEVLKYWWNLLQYWSQELKNSAVSLLNATAIAVAEGTDRV  819
49  IVTRIVELLGR-------RGWEVLKYWWNLLQYWSQELKNSAVSLLNATAIAVAEGTDRV  819
50  IVTRIVELLGR-------RGWEVLKYWWNLLQYWSQELKNSAVSLLNATAIAVAEGTDRV  819
51  IVTRIVELLGR-------RGWEVLKYWWNLLQYWSQKLKNSAVSLLNATAIAVAEGTDRV  819
52  IVTRIVELLGR-------RGWEVLKYWWNLLQYWSQELKNSAVSLLNATAIAVAEGTDRV  819
53  IVTRIVELLGR-------RGWEVLKYWWNLLQYWSQELKNSAVSLLNATAIAVAEGTDRV  819
54  IVTRIVELLGR-------RGWEVLKYWWNLLQYWSQELKNSAVSLLNATAIAVAEGTDRV  819
55  IVTRIVELLGR-------RGWEVLKYWWNLLQYWSQELKNSAVSLLNATAIAVAEGTDRV  819
56  IVTRIVELLGR-------RGWEVLKYWWNLLQYWSQELKNSAVSLLNATAIAVAEGTDRV  819
57  IVTRIVELLGR-------RGWEVLKYWWNLLQYWSQELKNSAVSLLNATAIAVAEGTDRV  819
58  IVTRIVELLGR-------RGWEVLKYWWNLLQYWSQELKNSAVSLLNATAIAVAEGTDRV  819
59  IVTRIVEPLGR-------RGWEVLKYWWNLLQYWSQELKNSAVSLLNATAIAVAEGTDRV  817
60  IVTRIVELLGR-------RGWEVLKYWWNLLQYWSQELKNSAVNLLNATAIAVAEGTDRV  819
61  IVTRIVELLGR-------RGWEVLKYWWNLLQYWSQELKNSAVSLLNATAIAVAEGTDRV  819
62  IVTRIVELLGR-------RGWEVLKYWWNLLQYWSQELKNSAVSLLNATAIAVAEGTDRV  819
63  IVTRIVELLGR-------RGWEVLKYWWNLLQYWSQELKNSAVSLLNATAIAVAEGTDRV  819
64  IVTRIVELLGR-------RGWEILKYWWNLLQYWSQELKNSAVSLLNATAIAVAEGTDRV  819
65  IVTRIVELLGR-------RGWEVLKYWWNLLQYWSQELKNSAVSLLNATAIAVAEGTDRV  819
66  IVTRIVKLLGR-------RGWEVLKYWWNLLQYWSQELKNSAVSLLNATAIAVAEGTDRV  818
67  IVTRIVELLGR-------RGWEVLKYWWNLLQYWSQELKNSAVSLLNATAIAVAEGTDRV  817
68  IVTRIVELLGR-------RGWEVLKYWWNLLQYWSQELKNSAVSLLNATAIAVAEGTDRV  819
```

```
    IEVVQRACRAILHIPRRIRQGLERALL
1   IEVVQRACRAILHIPRRIRQGLERALL  846
2   IEVVQRACRAILHIPRRIRQGLERALL  845
3   IEVVQRACRAILHIPRRIRQGLERALL  846
4   IEVVQRACRAILHIPRRIGQGLERALL  846
5   IEVVQRACRAILHIPRRIRQGLERALL  846
6   IEVVQRACRAILHIPRRIRQGLERALL  846
7   IEVVQRACRAILHIPRRIRQGLERALL  846
8   IEVVQRACRAILHIPRRIRQGLERALL  843
9   IEVVQRACRAILHIPRRIRQGLERALL  845
10  IEVVQRACRAILHIPRRIRQGLERALL  846
11  IEVVQRACRAILHIPRRIRQGLERALL  846
12  IEVVQRACRAILHIPRRIRQGLERALL  846
13  IEVVQRACRAILHIPRRIRQGLERALL  846
14  IEVVQRACRAILHIPRRIRQGLERALL  844
15  IEVVQRACRAILHIPRRIRQGLERALL  814
16  IEVVQRACRAILHIPRRIRQGLERALL  846
17  IEVVQRACRAILHIPRRIRQGLERALL  846
18  IEVVQRACRATLHIPRRIRQGLERALL  846
19  IEVVQRACRAILHIPRRIRQGLERALL  846
20  IEVVQRACRAILHIPRRIRQGLERALL  844
```

21	IEVVQRACRAILHIPRRIRQGLERALL	846
22	IEVVQRACRAILHIPRRIRQGLERALL	829
23	IEVVQRACRAILHIPRRIRQGLERALL	846
24	IEVVQRACRAILHIPRRIRQGLERALL	845
25	IEVVQRACRAILHIPRRIRQGLERALL	846
26	IEVVQRACRAILHIPRRIRQGLERALL	846
27	IEVVQRACRAILHIPRRIRQGLERALL	846
28	IEVVQRACRAILHIPRRIRQGLERALL	846
29	IEVVQRACRAILHIPRRIRQGLERALL	846
30	IEVVQRACRAILHIPRRIRQGLERALL	846
31	IEVVQRACRAILHIPRRIRQGLERALL	846
32	IEVVQRACRAILHIPRRIRQGLERALL	846
33	IEVVQRACRAILHIPRRIRQGLERALL	846
34	IEVVQRACRAILHIPRRIRQGLERALL	846
35	IEVVQRACRAILHTPRRIRQGLERALL	846
36	IEVVQRACRAILHIPRRIRQGLERALL	846
37	IEVVQRACRAILHIPRRIRQGLERALL	846
38	IEVVQRACRAILHIPRRIRQGLERALL	844
39	IEVVQRACRAILHIPRRIRQGLERALL	846
40	IEVVQRACRAILHIPRRIRQGLERALL	846
41	IEVVQRACRAILHIPRRIRQGLERALL	846
42	IEVVQRACRATLHIPRRIRQGLERALL	846
43	IEVVQRACRATLHIPRRIRQGLERALL	846
44	IEVVQRACRAIIHIPRRIRQGLERALL	846
45	IEVVQRACRATLHIPRRIRQGLERALL	846
46	IEVVQRACRAILHIPRRIRQGLERALL	846
47	IEVVQRACRAILHIPRRIRQGLERALL	846
48	IEVVQRACRATLHIPRRIRQGLERALL	846
49	IEVVQRACRAILHIPRRIRQGLERALL	846
50	IEVVQRACRAILHIPRRIRQGLERALL	846
51	IEVVQRACRAILHIPRRIRQGLERALL	846
52	IEVVQRACRATLHIPRRIRQGLERALL	846
53	IEVVQRACRAILHIPRRIRQGLERALL	846
54	IEVVQRACRAILHIPRRIRQGLERALL	846
55	IEVVQRACRAILHIPRRIRQGLERALL	846
56	IEVVQRACRATLHIPRRIRQGLERALL	846
57	IEVVQRACRAILHIPRRIRQGLERALL	846
58	IEVVQRACRAILHIPRRIRQGLERALL	846
59	IEVVQRACRAILHIPRRIRQGLERALL	844
60	IEVVQRACRATLHIPRRIRQGLERALL	846
61	IEVVQRACRAILHIPIRIRQGLERALL	846
62	IEVVQRACRAILHIPRRIRQGLERALL	846
63	IEVVQRACRAILHTPRRIRQGLERALL	846
64	IEVVQRACRATLHIPRRIRQGLERALL	846
65	IEVVQRACRATLHIPRRIRQGLERALL	846
66	IEVVQRACRATLHIPRRIRQGLERALL	845
67	IEVVQRACRAILHIPRRIRQGLERALL	844
68	IEVVQRACRATLHIPRRIRQGLERALL	846

Consensus

1. B.US.2006.700010040_C9_4520.EU289193
2. B.US.2007.CH0040_3_d0197_ipe032_27_50.MG900200
3. B.US.2007.CH0040_3_d0197_ipe032_2_03.MG900201
4. B.US.2007.CH0040_3_d0197_ipe032_2_04.MG900202
5. B.US.2007.CH0040_3_d0197_ipe032_2_10.MG900203
6. B.US.2007.CH0040_3_d0197_ipe032_2_12.MG900204
7. B.US.2007.CH0040_3_d0197_ipe032_2_13.MG900205
8. B.US.2007.CH0040_3_d0197_ipe032_2_14.MG900206
9. B.US.2007.CH0040_3_d0197_ipe032_2_17.MG900207
10. B.US.2007.CH0040_3_d0197_ipe032_2_18.MG900208
11. B.US.2007.CH0040_3_d0197_ipe032_2_33.MG900209
12. B.US.2007.CH0040_3_d0197_ipe032_2_35.MG900210
13. B.US.2007.CH0040_3_d0197_ipe032_2_36.MG900211
14. B.US.2007.CH0040_3_d0197_ipe032_2_38.MG900212
15. B.US.2007.CH0040_3_d0197_ipe032_2_39.MG900213
16. B.US.2007.CH0040_3_d0197_ipe032_2_42.MG900214
17. B.US.2007.CH0040_3_d0197_ipe032_2_43.MG900215
18. B.US.2007.CH0040_3_d0197_ipe032_3_46.MG900216
19. B.US.2007.CH0040_3_d0197_ipe032_3_52.MG900217
20. B.US.2007.CH0040_3_d0197_ipe032_3_54.MG900218
21. B.US.2007.CH0040_3_d0197_ipe032_3_55.MG900219
22. B.US.2007.CH0040_3_d0197_ipe032_3_56.MG900220
23. B.US.2007.CH0040_3_d0197_ipe032_3_59.MG900221
24. B.US.2007.CH0040_3_d0197_ipe032_3_60.MG900222
25. B.US.2007.CH0040_3_d0197_ipe032_3_61.MG900223
26. B.US.2007.CH0040_3_d0197_ipe032_3_64.MG900224
27. B.US.2007.CH0040_3_d0197_ipe032_3_65.MG900225
28. B.US.2007.CH0040_3_d0197_ipe032_3_66.MG900226
29. B.US.2007.CH0040_3_d0197_ipe032_3_68.MG900227
30. B.US.2007.CH0040_3_d0197_ipe032_3_69.MG900228
31. B.US.2007.CH0040_3_d0197_ipe032_3_70.MG900229
32. B.US.2007.CH0040_3_d0197_ipe032_3_74.MG900230
33. B.US.2007.CH0040_3_d0197_ipe032_3_75.MG900231

	**MRVMGIRKNYQHLWREGILLLGILMICSAADNLWVTVYYGVPVWREATTTLFCASDAKAY**	
1	MRVMGIRKNYQHLWREGILLLGILMICSAADNLWVTVYYGVPVWREATTTLFCASDAKAY	60
2	MRVMGIRKNYQHLWREGILLLGILMICSAADNLWVTVYYGVPVWREATTTLFCASDAKAY	60
3	MRVMGIRKNYQHLWREGILLLGILMICSAADNLWVTVYYGVPVWREATTTLFCASDAKAY	60
4	MRVMGIRKNYQHLWREGILLLGILMICSAADNLWVTVYYGVPVWREATTTLFCASDAKAY	60
5	MRVMGIRKNYQHLWREGILLLGILMICSAADNLWVTVYYGVPVWREATTTLFCASDAKAY	60
6	MRVMGIRKNYQHLWREGILLLGILMICSAADNLWVTVYYGVPVWREATTTLFCASDAKAY	60
7	MRVMGIRKNYQHLWREGILLLGILMICSAADNLWVTVYYGVPVWREATTTLFCASDAKAY	60
8	MRVMGIRKNYQHLWREGILLLGILMICSAADNLWVTVYYGVPVWREATTTLFCASDAKAY	60
9	MRVMGIRKNYQHLWREGILLLGILMICSAADNLWVTVYYGVPVWREATTTLFCASDAKAY	60
10	MRVMGIRKNYQHLWREGILLLGILMICSAADNLWVTVYYGVPVWREATTTLFCASDAKAY	60
11	MRVMGIRKNYQHLWREGILLLGILMICSAADNLWVTVYYGVPVWREATTTLFCASDAKAY	60
12	MRVMGIRKNYQHLWREGILLLGILMICSAADNLWVTVYYGVPVWREATTTLFCASDAKAY	60
13	MRVMGIRKNYQHLWREGILLLGILMICSAADNLWVTVYYGVPVWREATTTLFCASDAKAY	60
14	MRVMGIRKNYQHLWREGILLLGILMICSAADNLWVTVYYGVPVWREATTTLFCASDAKAY	60
15	MRVMGIRKNYQHLWREGILLLGILMICSAADNLWVTVYYGVPVWREATTTLFCASDAKAY	60
16	MRVMGIRKNYQHLWREGILLLGILMICSAADNLWVTVYYGVPVWREATTTLFCASDAKAY	60
17	MRVMGIRKNYQHLWREGILLLGILMICSAADNLWVTVYYGVPVWREATTTLFCASDAKAY	60
18	MRVMGIRKNYQHLWREGILLLGILMICSAADNLWVTVYYGVPVWREATTTLFCASDAKAY	60
19	MRVMGIRKNYQHLWREGILLLGILMICSAADNLWVTVYYGVPVWREATTTLFCASDAKAY	60
20	MRVMGIRKNYQHLWREGILLLGILMICSAADNLWVTVYYGVPVWREATTTLFCASDAKAY	60
21	MRVMGIRKNYQHLWREGILLLGILMICSAADNLWVTVYYGVPVWREATTTLFCASDAKAY	60
22	MRVMGIRKNYQHLWREGILLLGILMICSAADNLWVTVYYGVPVWREATTTLFCASDAKAY	60
23	MRVMGIRKNYQHLWREGILLLGILMICSAADNLWVTVYYGVPVWREATTTLFCASDAKAY	60
24	MRVMGIRKNYQHLWREGILLLGILMICSAADNLWVTVYYGVPVWREATTTLFCASDAKAY	60
25	MRVMGIRKNYQHLWREGILLLGILMICSAADNLWVTVYYGVPVWREATTTLFCASDAKAY	60
26	MRVMGIRKNYQHLWREGILLLGILMICSAADNLWVTVYYGVPVWREATTTLFCASDAKAY	60
27	MRVMGIRKNYQHLWREGILLLGILMICSAADNLWVTVYYGVPVWREATTTLFCASDAKAY	60
28	MRVMGIRKNYQHLWREGILLLGILMICSAADNLWVTVYYGVPVWREATTTLFCASDAKAY	60
29	MRVMGIRKNYQHLWREGILLLGILMICSAADNLWVTVYYGVPVWREATTTLFCASDAKAY	60
30	MRVMGIRKNYQHLWREGILLLGILMICSAADNLWVTVYYGVPVWREATTTLFCASDAKAY	60
31	MRVMGIRKNYQHLWREGILLLGILMICSAADNLWVTVYYGVPVWREATTTLFCASDAKAY	60
32	MRVMGIRKNYQHLWREGILLLGILMICSAADNLWVTVYYGVPVWREATTTLFCASDAKAY	60
33	MRVMGIRKNYQHLWREGILLLGILMICSAADNLWVTVYYGVPVWREATTTLFCASDAKAY	60

DTEAHNVWATHACVPTDPNPQEVELKNVTENFNMWENNMVEQMHEDIISLWDQSLKPCVK

1	DTEAHNVWATHACVPTDPNPQEVELKNVTENFNMWENNMVEQMHEDIISLWDQSLKPCVK	120
2	DTEAHNVWATHACVPTDPNPQEVELKNVTENFNMWENNMVEQMHEDIISLWDQSLKPCVK	120
3	DTEAHNVWATHACVPTDPNPQEVELKNVTENFNMWENNMVEQMHEDIISLWDQSLKPCVK	120
4	DTEAHNVWATHACVPTDPNPQEVELKNVTENFNMWENNMVEQMHEDIISLWDQSLKPCVK	120
5	DTEAHNVWATHACVPTDPNPQEVELKNVTENFNMWENNMVEQMHEDIISLWDQSLKPCVK	120
6	DTEAHNVWATHACVPTDPNPQEVELKNVTENFNMWENNMVEQMHEDIISLWDQSLKPCVK	120
7	DTEAHNVWATHACVPTDPNPQEVELKNVTENFNMWENNMVEQMHEDIISLWDQSLKPCVK	120
8	DTEAHNVWATHACVPTDPNPQEVELKNVTENFNMWENNMVEQMHEDIISLWDQSLKPCVK	120
9	DTEAHNVWATHACVPTDPNPQEVELKNVTENFNMWENNMVEQMHEDIISLWDQSLKPCVK	120
10	DTEAHNVWATHACVPTDPNPQEVELKNVTENFNMWENNMVEQMHEDIISLWDQSLKPCVK	120
11	DTEAHNVWATHACVPTDPNPQEVELKNVTENFNMWENNMVEQMHEDIISLWDQSLKPCVK	120
12	DTEAHNVWATHACVPTDPNPQEVELKNVTENFNMWENNMVEQMHEDIISLWDQSLKPCVK	120
13	DTEAHNVWATHACVPTDPNPQEVELKNVTENFNMWENNMVEQMHEDIISLWDQSLKPCVK	120
14	DTEAHNVWATHACVPTDPNPQEVELKNVTENFNMWENNMVEQMHEDIISLWDQSLKPCVK	120
15	DTEAHNVWATHACVPTDPNPQEVELKNVTENFNMWENNMVEQMHEDIISLWDQSLKPCVK	120
16	DTEAHNVWATHACVPTDPNPQEVELKNVTENFNMWENNMVEQMHEDIISLWDQSLKPCVK	120
17	DTEAHNVWATHACVPTDPNPQEVELKNVTENFNMWENNMVEQMHEDIISLWDQSLKPCVK	120
18	DTEAHNV-ATHACVPTDPNPQEVELKNVTENFNMWENNMVEQMHEDIISLWDQSLKPCVK	119
19	DTEAHNVWATHACVPTDPNPQEVELKNVTENFNMWENNMVEQMHEDIISLWDQSLKPCVK	120
20	DTEAHNVWATHACVPTDPNPQEVELKNVTENFNMWKNNMVEQMHEDIISLWDQSLKPCVK	120
21	DTEAHNVWATHACVPTDPNPQEVELKNVTENFNMWENNMVEQMHEDIISLWDQSLKPCVK	120
22	DTEAHNVWATHACVPTDPNPQEVELKNVTENFNMWENNMVEQMHEDIISLWDQSLKPCVK	120
23	DTEAHNVWATHACVPTDPNPQEVELKNVTENFNMWENNMVEQMHEDIISLWDQSLKPCVK	120
24	DTEAHNVWATHACVPTDPNPQEVELKNVTENFNMWENNMVEQMHEDIISLWDQSLKPCVK	120
25	DTEAHNVWATHACVPTDPNPQEVELKNVTENFNMWENNMVEQMHEDIISLWDQSLKP-VK	119
26	DTEAHNVWATHACVPTDPNPQEVELKNVTENFNMWENNMVEQMHEDIISLWDQSLKPCVK	120
27	DTEAHNVWATHACVPTDPNPQEVELKNVTENFNMWENNMVEQMHEDIISLWDQSLKPCVK	120
28	DTEAHNVWATHACVPTDPNPQEVELKNVTENFNMWENNMVEQMHEDIISLWDQSLKPCVK	120
29	DTEAHNVWATHACVPTDPNPQEVELKNVTENFNMWENNMVEQMHEDIISLWDQSLKPCVK	120
30	DTEAHNVWATHACVPTDPNPQEVELKNVTENFNMWENNMVEQMHEDIISLWDQSLKPCVK	120
31	DTEAHNVWATHACVPTDPNPQEVELKNVTENFNMWENNMVEQMHEDIISLWDQSLKPCVK	120
32	DTEAHNVWATHACVPTDPNPQEVELKNVTENFNMWENNMVEQMHEDIISLWDQSLKPCVK	120
33	DTEAHNVWATHACVPTDPNPQEVELKNVTENFNMWENNMVEQMHEDIISLWDQSLKPCVK	120

	LTPLCVTLNCTDLGNVTNTT---NSNGGMMEKGEVKNCSFKITTDIKDRTRKEYALFYKL	
1	LTPLCVTLNCTDLGNVTNTT---NSNGEMMEKGEVKNCSFKITTDIKDRTRKEYALFYKL	177
2	LTPLCVTLNCTDLGNVTNTT---NSNGGMMEKGEVKNCSFKITTDIKDRTRKEYALFYKL	177
3	LTPLCVTLNCTDLGNVTNTT---NSNGGMMEKGEVKNCSFKITTDIKDRTRKEYALFYKL	177
4	LTPLCVTLNCTDLGNVTNTT---NSNGGMMEKGEVKNCSFKITTDIKDRTRKEYALFYKL	177
5	LTPLCVTLNCTDLGNVTNTT---NSNGGMMEKGEVKNCSFKITTDIKDRTRKEYALFYKL	177
6	LTPLCVTLNCTDLGNVTNTT---NSNGGMMEKGEVKNCSFKITTDIKDRTRKEYALFYKL	177
7	LTPLCVTLNCTDLGNVTNTT---NSNGKMMEKGEVKNCSFKITTDIKDRTRKEYALFYKL	177
8	LTPLCVTLNCTDLGNVTNTT---NSNGGMMEKGEVKNCSFKITTDIKDRTRKEYALFYKL	177
9	LTPLCVTLNCTDLGNVTNTT---NSNEEMMEKGEVKNCSFKITTDIKDRTRKEYALFYKL	177
10	LTPLCVTLNCTDLGNVTNTT---NSNGGMMEKGEVKNCSFKITTDIKDRTRKEYALFYKL	177
11	LTPLCVTLNCTDLGNVTNTT---NSNGGMMEKGEVKNCSFKITTDIKDRTRKEYALFYKL	177
12	LTPLCVTLNCTDLGNVTNTT---NSNGGMMEKGEVKNCSFKITTDIKDRTRKEYALFYKL	177
13	LTPLCVTLNCTDLGNVTNTT---NSNGKMMEKGEVKNCSFKITTDIKDRTRKEYALFYKL	177
14	LTPLCVTLNCTDLGNVTNTT---NSNGGMMEKGEVKNCSFKITTDIKDRTRKEYALFYKL	177
15	LTPLCVTLNCTDLGNVTNIT---NSNGGMMEKGEVKNCSFKITTDIKDRTRKEYALFYKL	177
16	LTPLCVTLNCTDLGNVTNTT---NSNGGMMEKGEVKNCSFKITTDIKDRTRKEYALFYKL	177
17	LTPLCVTLNCTDLGNVTNTT---NSNGGMMEKGEVKNCSFKITTDIKDRTRKEYALFYKL	177
18	LTPLCVTLNCTDLGNVTNTT---NSKGEMMEKGEVKNCSFKITTDIKDRTRKEYALFYKL	176
19	LTPLCVTLNCTDLGNVTNTT---NSNGGMMEKGEVKNCSFKITTDIKDRTRKEYALFYKL	177
20	LTPLCVTLNCTDLGNVTNTT---NSNGKMMEKGEVKNCSFKITTDIKDRTRKEYALFYKL	177
21	LTPLCVTLNCTDLGNVTNTT---NSNGGMMEKGEVKNCSFKITTDIKDRTRKEYALFYKL	177
22	LTPLCVTLNCTDLGNVTNTT---NSNGGMMEKGEVKNCSFKITTDIKDRTRKEYALFYKL	177
23	LTPLCVTLNCTDLGNVTNTT---NSNGGMMEKGEVKNCSFKITTDIKDRTRKEYALFYKL	177
24	LTPLCVTLNCTDLGNVTNTT---NSNGGMMEKGEVKNCSFKITTDIKDRTRKEYALFYKL	177
25	LTPLCVTLNCTDLGNVTNTT---NSNGGMMEKGEVKNCSFKITTDIKDRTRKEYALFYKL	176
26	LTPLCVTLNCTDLGNVTNTT---NSNGGMMEKGEVKNCSFKITTDIKDRTRKEYALFYKL	177
27	LTPLCVTLNCTDLGNVTNTT---NSNGKMMEKGEVKNCSFKITTDIKDRTRKEYALFYKL	177
28	LTPLCVTLNCTDLGNVTNTT---NSNGGMMEKGEVKNCSFKITTDIKDRTRKEYALFYKL	177
29	LTPLCVTLNCTDLGNVTNTT---NSNGKMMEKGEVKNCSFKITTDIKDRTRKEYALFYKL	177
30	LTPLCVTLNCTDLGNVTNTT---NSKGEMMEKGEVKNCSFKITTDIKDRTRKEYALFYKL	177
31	LTPLCVTLNCTDLGNVTNTT---NSNGKMMEKGEVKNCSFKITTDIKDRTRKEYALFYKL	177
32	LTPLCVTLNCTDLGNVTNTT---NSNGGMMEKGEVKNCSFKITTDIKDRTRKEYALFYKL	177
33	LTPLCVTLNCTDLGNVTNTT---NSNGGMMEKGEVKNCSFKITTDIKDRTRKEYALFYKL	177

	**DVVPIND-------TRYRLVSCNTSVITQACPKVSFEPIPIHYCAPAGFAILKCNDKQFI**	
1	DVVPIND-------TRYRLVSCNTSVITQACPKVSFEPIPIHYCAPAGFAILKCNDKQFI	230
2	DVVPIND-------TRYRLVSCNTSVITQACPKVSFEPIPIHYCAPAGFAILKCNDKQFI	230
3	DVVPIND-------TRYRLVSCNTSVITQACPKVSFEPIPIHYCAPAGFAILKCNDKQFI	230
4	DVVPIND-------TRYRLVSCNTSVITQACPKVSFEPIPIHYCAPAGFAILKCNDKQFI	230
5	DVVPIND-------TRYRLVSCNTSVITQACPKVSFEPIPIHYCAPAGFAILKCNDKQFI	230
6	DVVPIND-------TRYRLVSCNTSVITQACPKVSFEPIPIHYCAPAGFAILKCNDKQFI	230
7	DVVPIND-------TRYRLVSCNTSVITQACPKVSFEPIPIHYCAPAGFAILKCNDKQFI	230
8	DVVPIND-------TRYRLVSCNTSVITQACPKVSFEPIPIHYCAPAGFAILKCNGKQFI	230
9	DVVPIND-------TRYRLVSCNTSVITQACPKVSFEPIPIHYCAPAGFAILKCNDKQFI	230
10	DVVPIND-------TRYRLVSCNTSVITQACPKVSFEPIPIHYCAPAGFAILKCNDKQFI	230
11	DVVPIND-------TRYRLVSCNTSVITQACPKVSFEPIPIHYCAPAGFAILKCNDKQFI	230
12	DVVPIND-------TRYRLVSCNTSVITQACPKVSFEPIPIHYCAPAGFAILKCNDKQFI	230
13	DVVPIND-------TRYRLVSCNTSVITQACPKVSFEPIPIHYCAPAGFAILKCNDKQFI	230
14	DVVPIND-------TRYRLVSCNTSVITQACPKVSFEPIPIHYCAPAGFAILKCNDKQFI	230
15	DVVPIND-------TRYRLVSCNTSVITQACPKVSFEPIPIHYCAPAGFAILKCNDKQFI	230
16	DVVPIND-------TRYRLVSCNTSVITQACPKVSFEPIPIHYCAPAGFAILKCNDKQFI	230
17	DVVPIND-------TRYRLVSCNTSVITQACPKVSFEPIPIHYCAPAGFAILKCNDKQFI	230
18	DVVPIND-------TRYRLVSCNTSVITQACPKVSFEPIPIHYCAPAGFAILKCNDKQFI	229
19	DVVPIND-------TRYRLVSCNTSVITQACPKVSFEPIPIHYCAPAGFAILKCNDKQFI	230
20	DVVPIND-------TRYRLVSCNTSVITQACPKVSFEPIPIHYCAPAGFAILKCNDKQFI	230
21	DVVPIND-------TRYRLVSCNTSVITQACPKVSFEPIPIHYCAPAGFAILKCNDKQFI	230
22	DVVPIND-------TRYRLVSCNTSVITQACPKVSFEPIPIHYCAPAGFAILKCNDKQFI	230
23	DVVPIND-------TRYRLVSCNTSVITQACPKVSFEPIPIHYCAPAGFAILKCNDKQFI	230
24	DVVPIND-------TRYRLVSCNTSVITQACPKVSFEPIPIHYCAPAGFAILKCNDKQFI	230
25	DVVPIND-------TRYRLVSCNTSVITQACPKVSFEPIPIHYCAPAGFAILKCNDKQFI	229
26	DVVPIND-------TRYRLVSCNTSVITQACPKVSFEPIPIYYCAPAGFAILKCNDKQFI	230
27	DVVPIND-------TRYRLVSCNTSVITQACPKVSFEPIPIHYCAPAGFAILKCNDKQFI	230
28	DVVPIND-------TRYRLVSCNTSVITQACPKVSFEPIPIHYCAPAGFAILKCNDKQFI	230
29	DVVPIND-------TRYRLVSCNTSVITQACPKVSFEPIPIHYCAPAGFAILKCNDKQFI	230
30	DVVPIND-------TRYRLVSCNTSVITQACPKVSFEPIPIHYCAPAGFAILKCNDKQFI	230
31	DVVPIND-------TRYRLVSCNTSVITQACPKVSFEPIPIHYCAPAGFAILKCNDKQFI	230
32	DVVPIND-------TRYRLVSCNTSVITQACPKVSFEPIPIHYCAPAGFAILKCNDKQFI	230
33	DVVPIND-------TRYRLVSCNTSVITQACPKVSFEPIPIHYCAPAGFAILKCNDKQFI	230

	**GTGPCTNVSTVQCTHGIRPVVSTQLLLNGSLAEEEVVIRSVNFSDNAKTIIVQLNKSVEI**	
1	GTGPCTNVSTVQCTHGIRPVVSTQLLLNGSLAEEEVVIRSVNFSDNAKTIIVQLNKSVEI	290
2	GTGPCTNVSTVQCTHGIRPVVSTQLLLNGSLAEEEVVIRSVNFSDNAKTIIVQLNKSVEI	290
3	GTGPCTNVSTVQCTHGIRPVVSTQLLLNGSLAEEEVVIRSVNFSDNAKTIIVQLNKSVEI	290
4	GTGPCTNVSTVQCTHGIRPVVSTQLLLNGSLAEEEVVIRSVNFSDNAKTIIVQLNKSVEI	290
5	GTGPCTNVSTVQCTHGIRPVVSTQLLLNGSLAEEEVVIRSVNFSDNAKTIIVQLNKSVEI	290
6	GTGPCTNVSTVQCTHGIRPVVSTQLLLNGSLAEEEVVIRSVNFSDNAKTIIVQLNKSVEI	290
7	GTGPCTNVSTVQCTHGIRPVVSTQLLLNGSLAEEEVVIRSVNFSDNAKTIIVQLNKSVEI	290
8	GTGPCTNVSTVQCTHGIRPVVSTQLLLNGSLAEEEVVIRSVNFSDNAKTIIVQLNKSVEI	290
9	GTGPCTNVSTVQCTHGIRPVVSTQLLLNGSLAEEEVVIRSVNFSDNAKTIIVQLNKSVEI	290
10	GTGPCTNVSTVQCTHGIRPVVSTQLLLNGSLAEEEVVIRSVNFSDNAKTIIVQLNKSVEI	290
11	GTGPCTNVSTVQCTHGIRPVVSTQLLLNGSLAEEEVVIRSVNFSDNAKTIIVQLNKSVEI	290
12	GTGPCTNVSTVQCTHGIRPVVSTQLLLNGSLAEEEVVIRSVNFSDNAKTIIVQLNKSVEI	290
13	GTGPCTNVSTVQCTHGIRPVVSTQLLLNGSLAEEEVVIRSVNFSDNAKTIIVQLNKSVEI	290
14	GTGPCTNVSTVQCTHGIRPVVSTQLLLNGSLAEEEVVIRSVNFSDNAKTIIVQLNKSVEI	290
15	GTGPCTNVSTVQCTHGIRPVVSTQLLLNGSLAEEEVVIRSVNFSDNAKTIIVQLNKSVEI	290
16	GTGPCTNVSTVQCTHGIRPVVSTQLLLNGSLAEEEVVIRSVNFSDNAKTIIVQLNKSVEI	290
17	GTGPCTNVSTVQCTHGIRPVVSTQLLLNGSLAEEEVVIRSVNFSDNAKTIIVQLNKSVEI	290
18	GTGPCTNVSTVQCTHGIRPVVSTQLLLNGSLAEEEVVIRSVNFSDNAKTIIVQLNKSVEI	289
19	GTGPCTNVSTVQCTHGIRPVVSTQLLLNGSLAEEEVVIRSVNFSDNAKTIIVQLNKSVEI	290
20	GTGPCTNVSTVQCTHGIRPVVSTQLLLNGSLAEEEVVIRSVNFSDNAKTIIVQLNKSVEI	290
21	GTGPCTNVSTVQCTHGIRPVVSTQLLLNGSLAEEEVVIRSVNFSDNAKTIIVQLNKSVEI	290
22	GTGPCTNVSTVQCTHGIRPVVSTQLLLNGSLAEEEVVIRSVNFSDNAKTIIVQLNKSVEI	290
23	GTGPCTNVSTVQCTHGIRPVVSTQLLLNGSLAEEEVVIRSVNFSDNAKTIIVQLNKSVEI	290
24	GTGPCTNVSTVQCTHGIRPVVSTQLLLNGSLAEEEVVIRSVNFSDNAKTIIVQLNKSVEI	290
25	GTGPCTNVSTVQCTHGIRPVVSTQLLLNGSLAEEEVVIRSVNFSDNAKTIIVQLNKSVEI	289
26	GTGPCTNVSTVQCTHGIRPVVSTQLLLNGSLAEEEVVIRSVNFSDNAKTIIVQLNKSVEI	290
27	GTGPCTNVSTVQCTHGIRPVVSTQLLLNGSLAEEEVVIRSVNFSDNAKTIIVQLNKSVEI	290
28	GTGPCTNVSTVQCTHGIRPVVSTQLLLNGSLAEEEVVIRSVNFSDNAKTIIVQLNKSVEI	290
29	GTGPCTNVSTVQCTHGIRPVVSTQLLLNGSLAEEEVVIRSVNFSDNAKTIIVQLNKSVEI	290
30	GTGPCTNVSTVQCTHGIRPVVSTQLLLNGSLAEEEVVIRSVNFSDNAKTIIVQLNKSVEI	290
31	GTGPCTNVSTVQCTHGIRPVVSTQLLLNGSLAEEEVVIRSVNFSDNAKTIIVQLNKSVKI	290
32	GTGPCTNVSTVQCTHGIRPVVSTQLLLNGSLAEEEVVIRSVNFSDNAKTIIVQLNKSVEI	290
33	GTGPCTNVSTVQCTHGIRPVVSTQLLLNGSLAEEEVVIRSVNFSDNAKTIIVQLNKSVEI	290

	TCTRPNNNTRKSIPMGPGKAFYARGDITGDIKKAYCKINGTEWHSTLKLVVEKLREQY-N	
1	TCTRPNNNTRKSIPMGPGKAFYARGDITGDIRKAYCEINGTEWHSTLKLVVEKLREQY-N	349
2	TCTRPNNNTRKSIPMGPGKAFYARGDITGDIKKAYCKINGTEWHSTLKLVVEKLREQY-N	349
3	TCTRPNNNTRKSIPMGPGKAFYARGDITGDIKKAYCKINGTEWHSTLKLVVEKLREQY-N	349
4	TCTRPNNNTRKSIPMGPGKAFYARGDITGDIKKAYCEINGTEWHSTLKLVVEKLREQY-N	349
5	TCTRPNNNTRKSIPMGPGKAFYARGDITGDIKKAYCKINGTEWHSTLKLVVEKLREQY-N	349
6	TCTRPNNNTRKSIPMGPGKAFYARGDITGDIKKAYCKINGTEWHSTLKLVVEKLREQY-N	349
7	TCIRPNNNTRKSIPMGPGKAFYARGDITGDIKKAYCEINGTEWHSTLKLVVEKLREQY-N	349
8	TCTRPNNNTRKSIPMGPGKAFYARGDITGDIKKAYCKINGTEWHSTLKLVVEKLREQY-N	349
9	TCTRPHNNTRKSIPMGPGKAFYARGDITGDIRKAYCEINGTEWHSTLKLVVEKLREQY-N	349
10	TCTRPNNNTRKSIPMGPGKAFYARGDITGDIKKAYCKINGTEWHSTLKLVVEKLREQY-N	349
11	TCTRPNNNTRKSIPMGPGKAFYARGDITGDIKKAYCKINGTEWHSTLKLVVEKLREQY-N	349
12	TCTRPNNNTRKSIPMGPGKAFYARGDITGDIKKAYCKINGTEWHSTLKLVVEKLREQY-N	349
13	TCTRPNNNTRKSIPMGPGKAFYARGDITGDIKKAYCEINGTEWHSTLKLVVEKLREQY-N	349
14	TCTRPNNNTRKSIPMGPGKAFYARGDITGDIKKAYCKINGTEWHSTLKLVVEKLREQY-N	349
15	TCTRPNNNTRKSIPMGPGKAFYARGDITGDIKKAYCKINGTEWHSTLKLVVEKLREQY-N	349
16	TCIRPNNNTRKSIPMGPGKAFYARGDITGDIKKAYCEINGTEWHSTLKLVVEKLREQY-N	349
17	TCTRPNNNTRKSIPMGPGKAFYARGDITGDIKKAYCKINGTEWHSTLKLVVEKLREQY-N	349
18	TCTRPHNNTRKSIPMGPGKAFYARGDITGDIRKAYCEINGTEWHSTLKLVVEKLREQY-N	348
19	TCTRPNNNTRKSIPMGPGKAFYARGDITGDI-KAYCKINGTEWHSTLKLVVEKLREQY-N	348
20	TCTRPNNNTRKSIPMGPGKAFYARGDITGDIRKAYCEINGTEWHSTLKLVVEKLREQY-N	349
21	TCTRPNNNTRKSIPMGPGKAFYARGDITGDIKKAYCKINGTEWHSTLKLVVEKLREQY-N	349
22	TCTRPNNNTRKSIPMGPGKAFYARGDITGDIKKASCEINGTEWHSTLKLVVEKLREQY-N	349
23	TCTRPNNNTRKSIPMGPGKAFYARGDITGDIKKAYCKINGTEWHSTLKLVVEKLREQY-N	349
24	TCTRPNNNTRKSIPMGPGKAFYARGDITGDIKKAYCKINGTEWHSTLKLVVEKLREQY-N	349
25	TCTRPNNNTRKSIPMGPGKAFYARGDITGDIKKAYCKINGTEWHSTLKLVVEKLREQY-N	348
26	TCTRPNNNTRKSIPMGPGKAFYARGDITGDIKKAYCKINGTEWHSTLKLVVEKLREQY-N	349
27	TCTRPNNNTRKSIPMGPGKAFYARGDITGDIRKAYCEINGTEWHSTLKLVVEKLREQY-N	349
28	TCTRPNNNTRKSIPMGPGKAFYARGDITGDIKKAYCKINGTEWHSTLKLVVEKLREQY-N	349
29	TCTRPNNNTRKSIPMGPGKAFYARGDITGDIRKAYCEINGTEWHSTLKLVVEKLREQY-N	349
30	TCTRPHNNTRKSIPMGPGKAFYARGDITGDIRKAYCEINGTEWHSTLKLVVEKLREQY-N	349
31	TCTRPHNNTRKSIPMGPGKAFYARGDITGDIRKAYCEINGTEWHSTLKLVVEKLREQY-N	349
32	TCTRPNNNTRKSIPMGPGKAFYARGDITGDIKKAYCKINGTEWHSTLKLVVEKLREQY-N	349
33	TCTRPNNNTRKSIPMGPGKAFYARGDITGDIKKAYCKINGTEWHSTLKLVVEKLREQY-N	349

	Sequence	
	KTIVFNRSSGGDPEIVMYSFNCGGEFFYCNSTKLFNSTWPWND-TKGSHDTNGTLILPCK	
1	KTIVFNRSSGGDPEIVMYSFNCGGEFFYCNSTKLFNSTWPWND-TKGSHDTNGTLILPCK	408
2	KTIVFNRSSGGDPEIVMYSFNCGGEFFYCNSTKLFNSTWPWND-AKGSHDTNGTLILPCK	408
3	KTIVFNRSSGGDPEIVMYSFNCGGEFFYCNSTKLFNSTWPWND-TKGSHDTNGTLILPCK	408
4	KTIVFNRSSGGDPEIVMYSFNCGGEFFYCNSTKLFNSTWPWND-TKGSHDTNGTLRLPCK	408
5	KTIVFNRSSGGDPEIVMYSFNCGGEFFYCNSTKLFNSTWPWND-TKGSHDTNGTLILPCK	408
6	KTIVFNRSSGGDPEIVMYSFNCGGEFFYCNSTKLFNSTWPWND-TKGSHDTNGTLILPCK	408
7	KTIVFNRSSGGDPEIVMYSFNCGGEFFYCNSTKLFNSTWPWND-TKGSHDTNGTLMLPCK	408
8	KTIVFNRSSGGDPEIVMYSFNCGGEFFYCNSTKLFNSTWPWND-TKGSHDTNGTLILPCK	408
9	KTIVFNRSSGGDPEIVMYSFNCGGEFFYCNSTKLFNSTWPWND-TKGSHDTNGTLILPCK	408
10	KTIVFNRSSGGDPEIVMYSFNCGGEFFYCNSTKLFNSTWPWND-TKGSHDTNGTLILPCK	408
11	KTIVFNRSSGGDPEIVMYSFNCGGEFFYCNSTKLFNSTWPWND-TKGSHDTNGTLILPCK	408
12	KTIVFNRSSGGDPEIVMYSFNCGGEFFYCNSTKLFNSTWPWND-TKGSHDTNGTLILPCK	408
13	KTIVFNRSSGGDPEIVMYSFNCGGEFFYCNSTKLFNSTWPWND-TKGSHDTNGTLMLPCK	408
14	KTIVFNRSSGGDPEIVMYSFNCGGEFFYCNSTKLFNSTWPWND-TKGSHDTNGTLILPCK	408
15	KTIVFNRSSGGDPEIVMYSFNCGGEFFYCNSTKLFNSTWPWND-TKGSHDTNGTLILPCK	408
16	KTIVFNRSSGGDPEIVMYSFNCGGEFFYCNSTKLFNSTWPWND-TKGSHDTNDTLRLPCK	408
17	KTIVFNRSSGGDPEIVMYSFNCGGEFFYCNSTKLFNSTWPWND-TKGSHDTNGTLILPCK	408
18	KTIVFNRSSGGDPEIVMYSFNCGGEFFYCNSTKLFNSTWPWND-TKGSHDTNGTLILPCK	407
19	KTIVFNRSSGGDPEIVMYSFNCGGEFFYCNSTKLFNSTWPWND-AKGSHDTNGTLILPCK	407
20	KTIVFNRSSGGDPEIVMYSFNCGGEFFYCNSTKLFNSTWPWND-TKGSHDTNDTLMLPCK	408
21	KTIVFNRSSGGDPEIVMYSFNCGGEFFYCNSTKLFNSTWPWND-TKGSHDTNGTLILPCK	408
22	KTIVFNRSSGGDPEIVMYSFNCGGEFFYCNSTKLFNSTWPWND-TKGSHDTNGTLILPCK	408
23	KTIVFNRSSGGDPEIVMYSFNCGGEFFYCNSTKLFNSTWPWND-AKGSHDTNGTLILPCK	408
24	KTIVFNRSSGGDPEIVMYSFNCGGEFFYCNSTKLFNSTWPWND-TKGSHDTNGTLILPCK	408
25	KTIVFNRSSGGDPEIVMYSFNCGGEFFYCNSTKLFNSTWPWND-TKGSHDTNGTLILPCK	407
26	KTIVFNRSSGGDPEIVMYSFNCGGEFFYCNSTKLFNSTWPWND-TKGSHDTNGTLILPCK	408
27	KTIVFNRSSGGDPEIVMYSFNCGGEFFYCNSTKLFNSTWPWND-TKGSHDTNDTLMLPCK	408
28	KTIVFNRSSGGDPEIVMYSFNCGGEFFYCNSTKLFNSTWPWND-AKGSHDTNGTLILPCK	408
29	KTIVFNRSSGGDPEIVMYSFNCGGEFFYCNSTKLFNSTWPWND-TKGSHDTNDTLMLPCK	408
30	KTIVFNRSSGGDPEIVMYSFNCGGEFFYCNSTKLFNSTWPWND-TKGSHDTNGTLILPCK	408
31	KTIVFNRSSGGDPEIVMYSFNCGGEFFYCNSTKLFNSTWPWND-TKGSHDTNGTLILPCK	408
32	KTIVFNRSSGGDPEIVMYSFNCGGEFFYCNSTKLFNSTWPWND-TKGSHDTNGTLILPCK	408
33	KTIVFNRSSGGDPEIVMYSFNCGGEFFYCNSTKLFNSTWPWND-TKGSHDTNGTLILPCK	408

	IKQIINMWQGVGKAMYAPPIEGKIRCSSNITGLLLTRDGG-YESNETDEIFRPGGGDMRD	
1	IKQIINMWQGVGKAMYAPPIEGKIRCSSNITGLLLTRDGG-YESNETDEIFRPGGGDMRD	467
2	IKQIINMWQGVGKAMYAPPIEGKIRCSSNITGLLLTRDGG-YESNETDEIFRPGGGDMRD	467
3	IKQIINMWQGVGKAMYAPPIEGKIRCSSNITGLLLTRDGG-YESNETDEIFRPGGGDMRD	467
4	IKQIINMWQGVGKAMYAPPIEGKIRCSSNITGLLLTRDGG-YESNETDEIFRPGGGDMRD	467
5	IKQIINMWQGVGKAMYAPPIEGKIRCSSNITGLLLTRDGG-YESNETDEIFRPGGGDMRD	467
6	IKQIINMWQGVGKAMYAPPIEGKIRCSSNITGLLLTRDGG-YESNETDEIFRPGGGDMRD	467
7	IKQIINMWQGVGKAMYAPPIEGKIRCSSNITGLLLTRDGG-YESNETDEIFRPGGGDMRD	467
8	IKQIINMWQGVGKAMYAPPIEGKIRCSSNITGLLLTRDGG-YESNETDEIFRPGGGDMRD	467
9	IKQIINMWQGVGKAMYAPPIEGKIRCSSNITGLLLTRDGG-YESNETDEIFRPGGGDMRD	467
10	IKQIINMWQGVGKAMYAPPIEGKIRCSSNITGLLLTRDGG-YESNETDEIFRPGGGDMRD	467
11	IKQIINMWQGVGKAMYAPPIEGKIRCSSNITGLLLTRDGG-YESNETDEIFRPGGGDMRD	467
12	IKQIINMWQGVGKAMYAPPIEGKIRCSSNITGLLLTRDGG-YESNETDEIFRPGGGDMRD	467
13	IKQIINMWQGVGKAMYAPPIEGKIRCSSNITGLLLTRDGG-YESNETDEIFRPGGGDMRD	467
14	IKQIINMWQGVGKAMYAPPIEGKIRCSSNITGLLLTRDGG-YESNETDEIFRPGGGDMRD	467
15	IKQIINMWQGVGKAMYAPPIEGKIRCSSNITGLLLTRDGG-YESNETDEIFRPGGGDMRD	467
16	IKQIINMWQGVGKAMYAPPIEGKIRCSSNITGLLLTRDGG-YESNETDEIFRPGGGDMRD	467
17	IKQIINMWQGVGKAMYAPPIEGKIRCSSNITGLLLTRDGG-YESNETDEIFRPGGGDMRD	467
18	IKQIINMWQGVGKAMYAPPIEGKIRCSSNITGLLLARDGG-YESNETDEIFRPGGGDMRD	466
19	IKQIINMWQGVGKAMYAPPIEGKIRCSSNITGLLLTRDGG-YESNETDEIFRPGGGDMRD	466
20	IKQIINMWQGVGKAMYAPPIEGQIRCSSNITGLLLTRDGG-YESNETDEIFRPGGG-MRD	466
21	IKQIINMWQGVGKAMYAPPIEGKIRCSSNITGLLLTRDGG-YESNETDEIFRPGGGDMRD	467
22	IKQIINMWQGVGKAMYAPPIEGKIRCSSNITGLLLTRDGG-YESNETDEIFRPGGGDMRD	467
23	IKQIINMWQGVGKAMYAPPIEGKIRCSSNITGLLLTRDGG-YESNETDEIFRPGGGDMRD	467
24	IKQIINMWQGVGKAMYAPPIEGKIRCSSNITGLLLTRDGG-YESNETDEIFRPGGGDMRD	467
25	IKQIINMWQGVGKAMYAPPIEGKIRCSSNITGLLLTRDGG-YENNETDEIFRPGGGDMRD	466
26	IKQIINMWQGVGKAMYAPPIEGKIRCSSNITGLLLTRDGG-YESNETDEIFRPGGGDMRD	467
27	IKQIINMWQGVGKAMYAPPIEGKIRCSSNITGLLLTRDGG-YESNETDEIFRPGGGDMRD	467
28	IKQIINMWQGVGKAMYAPPIEGKIRCSSNITGLLLTRDGG-YESNETDEIFRPGGGDMRD	467
29	IKQIINMWQGVGKAMYAPPIEGQIRCSSNITGLLLTRDGG-YESNETDEIFRPGGGDMRD	467
30	IKQIINMWQGVGKAMYAPPIEGKIRCSSNITGLLLARDGG-YESNETDEIFRPGGGDMRD	467
31	IKQIINMWQGVGKAMYAPPIEEKIRCSSNITGLLLTRDGG-YESNETDEIFRPGGGDMRD	467
32	IKQIINMWQGVGKAMYAPPIEGKIRCSSNITGLLLTRDGG-YESNETDEIFRPGGGDMRD	467
33	IKQIINMWQGVGKAMYAPPIEGKIRCSSNITGLLLTRDGG-YESNETDEIFRPGGGDMRD	467

	**NWRSELYKYKVVKIEPLGVAPTKAKRRVVQREKRAFG-LGAVFLGFLGAAGSTMGAASIT**	
1	NWRSELYKYKVVKIEPLGVAPTKAKRRVVQREKRAFG-LGAVFLGFLGAAGSTMGAASIT	526
2	NWRSELYKYKVVKIEPLGVAPTKAKRRVVQREKRAFG-LGAVFLGFLGAAGSTMGAASIT	526
3	NWRSELYKYKVVKIEPLGVAPTKAKRRVVQREKRAFG-LGAVFLGFLGAAGSTMGAASIT	526
4	NWRSELYKYKVVKIEPLGVAPTKAKRRVVQREKRAFG-LGAVFLGFLGAAGSTMGAASIT	526
5	NWRSELYKYKVVKIEPLGVAPTKAKRRVVQREKRAFG-LGAVFLGFLGAAGSTMGAASIT	526
6	NWRSELYKYKVVKIEPLGVAPTKAKRRVVQREKRAFG-LGAVFLGFLGAAGSTMGAASIT	526
7	NWRSELYKYKVVKIEPLGVAPTKAKRRVVQREKRAFG-LGAVFLGFLGAAGSTMGAASIT	526
8	NWRSELYKYKVVKIEPLGVAPTKAKRRVVQREKRAFG-LGAVFLGFLGAAGSTMGAASIT	526
9	NWRSELYKYKVVKIEPLGVAPTKAKRRVVQREKRAFG-LGAVFLGFLGAAGSTMGAASIT	526
10	NWRSELYKYKVVKIEPLGVAPTKAKRRVVQREKRAFG-LGAVFLGFLGAAGSTMGAASIT	526
11	NWRSELYKYKVVKIEPLGVAPTKAKRRVVQREKRAFG-LGAVFLGFLGAAGSTMGAASIT	526
12	NWRSELYKYKVVKIEPLGVAPTKAKRRVVQREKRAFG-LGAVFLGFLGAAGSTMGAASIT	526
13	NWRSELYKYKVVKIEPLGVAPTKAKRRVVQREKRAFG-LGAVFLGFLGAAGSTMGAASIT	526
14	NWRSELYKYKVVKIEPLGVAPTKAKRRVVQREKRAFG-LGAVFLGFLGAAGSTMGAASIT	526
15	NWRSELYKYKVVKIEPLGVAPTKAKRRVVQREKRAFG-LGAVFLGFLGAAGSTMGAASIT	526
16	NWRSELYKYKVVKIEPLGVAPTKAKRRVVQREKRAFG-LGAVFLGFLGAAGSTMGAASIT	526
17	NWRSELYKYKVVKIEPLGVAPTKAKRRVVQREKRAFG-LGAVFLGFLGAAGSTMGAASIT	526
18	NWRSELYKYKVVKIEPLGVAPTKAKRRVVQREK-AFG-LGAVFLGFLGAAGSTMGAASIT	524
19	NWRSELYKYKVVKIEPLGVAPTKAKRRVVQREKRAFG-LGAVFLGFLGAAGSTMGAASIT	525
20	NWRSELYKYKVVKIEPLGVAPTKAKRRVVQREKRAFG-LGAVFLGFLGAAGSTMGAASIT	525
21	NWRSELYKYKVVKIEPLGVAPTKAKRRVVQREKRAFG-LGAVFLGFLGAAGSTMGAASIT	526
22	NWRSELYKYKVVKIEPLGVAPTKAKRRVVQREKRAFG-LGAVFLGFLGAAGSTMGAASIT	526
23	NWRSELYKYKVVKIEPLGVAPTKAKRRVVQREKRAFG-LGAVFLGFLGAAGSTMGAASIT	526
24	NWRSELYKYKVVKIEPLGVAPTKAKRRVVQREKRAFG-LGAVFLGFLGAAGSTMGAASIT	526
25	NWRSELYKYKVVKIEPLGVAPTKAKRRVVQREKRAFG-LGAVFLGFLGAAGSTMGAASIT	525
26	NWRSELYKYKVVKIEPLGVAPTRAKRRVVQREKRAFG-LGAVFLGFLGAAGSTMGAASIT	526
27	NWRSELYKYKVVKIEPLGVAPTKAKRRVVQREKRAFG-LGAVFLGFLGAAGSTMGAASIT	526
28	NWRSELYKYKVVKIEPLGVAPTKAKRRVVQREKRAFG-LGAVFLGFLGAAGSTMGAASIT	526
29	NWRSELYKYKVVKIEPLGVAPTKAKRRVVQREKRAFG-LGAVFLGFLGAAGSTMGAASIT	526
30	NWRSELYKYKVVKIEPLGVAPTKAKRRVVQREKRAFG-LGAVFLGFLGAAGSTMGAASIT	526
31	NWRSELYKYKVVKIEPLGVAPTKAKRRVVQREKRAFG-LGAVFLGFLGAAGSTMGAASIT	526
32	NWRSELYKYKVVKIEPLGVAPTKAKRRVVQREKRAFG-LGAVFLGFLGAAGSTMGAASIT	526
33	NWRSELYKYKVVKIEPLGVAPTKAKRRVVQREKRAFG-LGAVFLGFLGAAGSTMGAASIT	526

LTVQARQLLSGIVQQQNNLLRAIEAQQHLLQLTVWGIKQLQARVLAVERYLKDQQLLGIW

1	LTVQARQLLSGIVQQQNNLLRAIEAQQHLLQLTVWGIKQLQARVLAVERYLKDQQLLGIW	586
2	LTVQARQLLSGIVQQQNNLLRAIEAQQHLLQLTVWGIKQLQARVLAVERYLKDQQLLGIW	586
3	LTVQARQLLSGIVQQQNNLLRAIEAQQHLLQLTVWGIKQLQARVLAVERYLKDQQLLGIW	586
4	LTVQARQLLSGIVQQQNNLLRAIEAQQHLLQLTVWGIKQLQARVLAVERYLKDQQLLGIW	586
5	LTVQARQLLSGIVQQQNNLLRAIEAQQHLLQLTVWGIKQLQARVLAVERYLKDQQLLGIW	586
6	LTVQARQLLSGIVQQQNNLLRAIEAQQHLLQLTVWGIKQLQARVLAVERYLKDQQLLGIW	586
7	LTVQARQLLSGIVQQQNNLLRAIEAQQHLLQLTVWGIKQLQARVLAVERYLKDQQLLGIW	586
8	LTVQARQLLSGIVQQQNNLLRAIEAQQHLLQLTVWGIKQLQARVLAVERYLKDQQLLGIW	586
9	LTVQARQLLSGIVQQQNNLLRAIEAQQHLLQLTVWGIKQLQARVLAVERYLKDQQLLGIW	586
10	LTVQARQLLSGIVQQQNNLLRAIEAQQHLLQLTVWGIKQLQARVLAVERYLKDQQLLGIW	586
11	LTVQARQLLSGIVQQQNNLLRAIEAQQHLLQLTVWGIKQLQARVLAVERYLKDQQLLGIW	586
12	LTVQARQLLSGIVQQQNNLLRAIEAQQHLLQLTVWGIKQLQARVLAVERYLKDQQLLGIW	586
13	LTVQARQLLSGIVQQQNNLLRAIEAQQHLLQLTVWGIKQLQARVLAVERYLKDQQLLGIW	586
14	LTVQARQLLSGIVQQQNNLLRAIEAQQHLLQLTVWGIKQLQARVLAVERYLKDQQLLGIW	586
15	LTVQARQLLSGIVQQQNNLLRAIEAQQHLLQLTVWGIKQLQARVLAVERYLKDQQLLGIW	586
16	LTVQARQLLSGIVQQQNNLLRAIEAQQHLLQLTVWGIKQLQARVLAVERYLKDQQLLGIW	586
17	LTVQARQLLSGIVQQQNNLLRAIEAQQHLLQLTVWGIKQLQARVLAVERYLKDQQLLGIW	586
18	LTVQARQLLSGIVQQQNNLLRAIEAQQHLLQLTVWGIKQLQARVLAVERYLKDQQLLGIW	584
19	LTVQARQLLSGIVQQQNNLLRAIEAQQHLLQLTVWGIKQLQARVLAVERYLKDQQLLGIW	585
20	LTVQARQLLSGIVQQQNNLLRAIEAQQHLLQLTVWGIKQLQARVLAVERYLKDQQLLGIW	585
21	LTVQARQLLSGIVQQQNNLLRAIEAQQHLLQLTVWGIKQLQARVLAVERYLKDQQLLGIW	586
22	LTVQARQLLSGIVQQQNNLLRAIEAQQHLLQLTVWGIKQLQARVLAVERYLKDQQLLGIW	586
23	LTVQARQLLSGIVQQQNNLLRAIEAQQHLLQLTVWGIKQLQARVLAVERYLKDQQLLGIW	586
24	LTVQARQLLSGIVQQQNNLLRAIEAQQHLLQLTVWGIKQLQARVLAVERYLKDQQLLGIW	586
25	LTVQARQLLSGIVQQQNNLLRAIEAQQHLLQLTVWGIKQLQARVLAVERYLKDQQLLGIW	585
26	LTVQARQLLSGIVQQQNNLLRAIEAQQHLLQLTVWGIKQLQARVLAVERYLKDQQLLGIW	586
27	LTVQARQLLSGIVQQQNNLLRAIEAQQHLLQLTVWGIKQLQARVLAVERYLKDQQLLGIW	586
28	LTVQARQLLSGIVQQQNNLLRAIEAQQHLLQLTVWGIKQLQARVLAVERYLKDQQLLGIW	586
29	LTVQARQLLSGIVQQQNNLLRAIETQQHLLQLTVWGIKQLQARVLAVERYLKDQQLLGIW	586
30	LTVQARQLLSGIVQQQNNLLRAIEAQQHLLQLTVWGIKQLQARVLAVERYLKDQQLLGIW	586
31	LTVQARQLLSGIVQQQNNLLRAIEAQQHLLQLTVWGIKQLQARVLAVERYLKDQQLLGIW	586
32	LTVQARQLLSGIVQQQNNLLRAIEAQQHLLQLTVWGIKQLQARVLAVERYLKDQQLLGIW	586
33	LTVQARQLLSGIVQQQNNLLRAIEAQQHLLQLTVWGIKQLQARVLAVERYLKDQQLLGIW	586

	**GCSGKLICTTTVPWNTSWSNKSLEQIWDNMTWMEWEREIDNYTGYIYQLIEESQNQQEKN**	
1	GCSGKLICTTTVPWNTSWSNKSLEQIWDNMTWMEWEREIDNYTGYIYQLIEESQNQQEKN	646
2	GCSGKLICTTTVPWNTSWSNKSLEQIWDNMTWMEWEREIDNYTGYIYQLIEESQNQQEKN	646
3	GCSGKLICTTTVPWNTSWSNKSLEQIWDNMTWMEWEREIDNYTGYIYQLIEESQNQQEKN	646
4	GCSGKLICTTTVPWNTSWSNKSLEQIWDNMTWMEWEREIDNYTGYIYQLIEESQNQQEKN	646
5	GCSGKLICTTTVPWNTSWSNKSLEQIWDNMTWMEWEREIDNYTGYIYQLIEESQNQQEKN	646
6	GCSGKLICTTTVPWNTSWSNKSLEQIWDNMTWMEWEREIDNYTGYIYQLIEESQNQQEKN	646
7	GCSGKLICTTTVPWNTSWSNKSLEQIWDNMTWMEWEREIDNYTGYIYQLIEESQNQQEKN	646
8	GCSGKLICTTTVPWNTSWSNKSLEQIWDNMTWMEWEREIDNYTGYIYQLIEESQNQQEKN	646
9	GCSGKLICTTTVPWNTSWSNKSLEQIWDNMTWMEWEREIDNYTGYIYQLIEESQNQQEKN	646
10	GCSGKLICTTTVPWNTSWSNKSLEQIWDNMTWMEWEREIDNYTGYIYQLIEESQNQQEKN	646
11	GCSGKLICTTTVPWNTSWSNKSLEQIWDNMTWMEWEREIDNYTGYIYQLIEESQNQQEKN	646
12	GCSGKLICTTTVPWNTSWSNKSLEQIWDNMTWMEWEREIDNYTGYIYQLIEESQNQQEKN	646
13	GCSGKLICTTTVPWNTSWSNKSLEQIWDNMTWMEWEREIDNYTGYIYQLIEESQNQQEKN	646
14	GCSGKLICTTTVPWNTSWSNKSLEQIWDNMTWMEWEREIDNYTGYIYQLIEESQNQQEKN	646
15	GCSGKLICTTTVPWNTSWSNKSLEQIWDNMTWMEWEREIDNYTGYIYQLIEESQNQQEKN	646
16	GCSGKLICTTTVPWNTSWSNKSLEQIWDNMTWMEWEREIDNYTGYIYQLIEESQNQQEKN	646
17	GCSGKLICTTTVPWNTSWSNKSLEQIWDNMTWMEWEREIDNYTGYIYQLIEESQNQQEKN	646
18	GCSGKLICTTTVPWNTSWSNKSLEQIWDNMTWMEWEREIDNYTGYIYQLIEESQNQQEKN	644
19	GCSGKLICTTTVPWNTSWSNKSLEQIWDNMTWMEWEREIDNYTGYIYQLIEESQNQQEKN	645
20	GCSGKLICTTTVPWNTSWSNKSLEQIWDNMTWMEWEREIDNYTGYIYQLIEESQNQQEKN	645
21	GCSGKLICTTTVPWNTSWSNKSLEQIWDNMTWMEWEREIDNYTGYIYQLIEESQNQQEKN	646
22	GCSGKLICTTTVPWNTSWSNKSLEQIWDNMTWMEWEREIDNYTGYIYQLIEESQNQQEKN	646
23	GCSGKLICTTTVPWNTSWSNKSLEQIWDNMTWMEWEREIDNYTGYIYQLIEESQNQQEKN	646
24	GCSGKLICTTTVPWNTSWSNKSLEQIWDNMTWMEWEREIDNYTGYIYQLIEESQNQQEKN	646
25	GCSGKLICTTTVPWNTSWSNKSLEQIWDNMTWMEWEREIDNYTGYIYQLIEESQNQQEKN	645
26	GCSGKLICTTTVPWNTSWSNKSLEQIWDNMTWMEWEREIDNYTGYIYQLIEESQNQQEKN	646
27	GCSGKLICTTTVPWNTSWSNKSLEQIWDNMTWMEWEREIDNYTGYIYQLIEESQNQQEKN	646
28	GCSGKLICTTTVPWNTSWSNKSLEQIWDNMTWMEWEREIDNYTGYIYQLIEESQNQQEKN	646
29	GCSGKLICTTTVPWNTSWSNKSLEQIWDNMTWMEWEREIDNYTGYIYQLIEESQNQQEKN	646
30	GCSGKLICTTTVPWNTSWSNKSLEQIWDNMTWMEWEREIDNYTGYIYQLIEESQNQQEKN	646
31	GCSGKLICTTTVPWNTSWSNKSLEQIWDNMTWMEWEREIDNYTGYIYQLIEESQNQQEKN	646
32	GCSGKLICTTTVPWNTSWSNKSLEQIWDNMTWMEWEREIDNYTGYIYQLIEESQNQQEKN	646
33	GCSGKLICTTTVPWNTSWSNKSLEQIWDNMTWMEWEREIDNYTGYIYQLIEESQNQQEKN	646

	**EQELLALDKWASLWNWFDITNWLWYIKIFIMIVGGLIGLRIVFTVLSIVNRVRQGYSPLS**	
1	EQELLALDKWASLWNWFDITNWLWYIKIFIMIVGGLIGLRIVFTVLSIVNRVRQGYSPLS	706
2	EQELLALDKWASLWNWFDITNWLWYIKIFIMIVGGLISLRIVFTVLSIVNRVRQGYSPLS	706
3	EQELLALDKWASLWNWFDITNWLWYIKIFIMIVGGLIGLRIVFTVLSIVNRVRQGYSPLS	706
4	EQELLALDKWASLWNWFDITNWLWYIKIFIMIVGGLIGLRIVFTVLSIVNRVRQGYSPLS	706
5	EQELLALDKWASLWNWFDITNWLWYIKIFIMIVGGLIGLRIVFTVLSIVNRVRQGYSPLS	706
6	EQELLALDKWASLWNWFDITNWLWYIKIFIMIVGGLIGLRIVFTVLSIVNRVRQGYSPLS	706
7	EQELLALDKWASLWNWFDITNWLWYIKIFIMIVGGLIGLRIVFTVLSIVNRVRQGYSPLS	706
8	EQELLAWDKWASLWNWFDITNWLWYIKIFIMIVGGLIGLRIVFTVLSIVNRVRQGYSPLS	706
9	EQELLALDKWASLWNWFDITNWLWYIKIFIMIVGGLIGLRIVFTVLSIVNRVRQGYSPLS	706
10	EQELLALDKWASLWNWFDITNWLWYIKIFIMIVGGLIGLRIVFTVLSIVNRVRQGYSPLS	706
11	EQELLALDKWASLWNWFDITNWLWYIKIFIMIVGGLIGLRIVFTVLSIVNRVRQGYSPLS	706
12	EQELLALDKWASLWNWFDITNWLWYIKIFIMIVGGLIGLRIVFTVLSIVNRVRQGYSPLS	706
13	EQELLALDKWASLWNWFDITNWLWYIKIFIMIVGGLIGLRIVFTVLSIVNRVRQGYSPLS	706
14	EQELLALDKWASLWNWFDITNWLWYIKIFIMIVGGLIGLRIVFTVLSIVNRVRQGYSPLS	706
15	EQELLALDKWASLWNWFDITNWLWYIKIFIMIVGGLIGLRIVFTVLSIVNRVRQGYSPLS	706
16	EQELLALDKWASLWNWFDITNWLWYIKIFIMIVGGLIGLRIVFTVLSIVNRVRQGYSPLS	706
17	EQELLALDKWAGLWNWFDITNWLWYIKIFIMIVGGLIGLRIVFTVLSIVNRVRQGYSPLS	706
18	EQELLALDKWASLWNWFDITNWLWYIKIFIMIVGGLIGLRIVFTVLSIVNRVRQGYSPLS	704
19	EQELLALDKWASLWNWFDITNWLWYIKIFIMIVGGLIGLRIVFTVLSIVNRVRQGYSPLS	705
20	EQELLALDKWASLWNWFDITNWLWYIKIFIMIVGGLIGLRIVFTVLSIVNRVRQGYSPLS	705
21	EQELLALDKWASLWNWFDITNWLWYIKIFIMIVGGLIGLRIVFTVLSIVNRVRQGYSPLS	706
22	EQELLALDKWASLWNWFDITNWLWYIKIFIMIVGGLIGLRIVFTVLSIVNRVRQGYSPLS	706
23	EQELLALDKWASLWNWFDITNWLWYIKIFIMIVGGLIGLRIVFTVLSIVNRVRQGYSPLS	706
24	EQELLAWDKWASLWNWFDITNWLWYIKIFIMIVGGLIGLRIVFTVLSIVNRVGQGYSPLS	706
25	EQELLAWDKWASLWNWFDITNWLWYIKIFIMIVGGLIGLRIVFTVLSIVNRVRQGYSPLS	705
26	EQELLALDKWASLWNWFDITNWLWYIKIFIMIVGGLIGLRIVFTVLSIVNRVRQGYSPLS	706
27	EQELLALDKWASLWNWFDITNWLWYIKIFIMIVGGLIGLRIVFTVLSIVNRVRQGYSPLS	706
28	EQELLALDKWASLWNWFDITHWLWYIKIFIMIVGGLIGLRIVFTVLSIVNRVRQGYSPLS	706
29	EQELLALDKWASLWNWFDITNWLWYIKIFIMIVGGLIGLRIVFTVLSIVNRVRQGYSPLS	706
30	EQELLALDKWASLWNWFDITNWLWYIKIFIMIVGGLIGLRIVFTVLSIVNRVRQGYSPLS	706
31	EQELLALDKWASLWNWFDITNWLWYIKIFIMIVGGLIGLRIVFTVLSIVNRVRQGYSPLS	706
32	EQELLALDKWASLWNWFDITNWLWYIKIFIMIVGGLIGLRIVFTVLSIVNRVRQGYSPLS	706
33	EQELLALDKWASLWNWFDITNWLWYIKIFIMIVGGLIGLRIVFTVLSIVNRVRQGYSPLS	706

	**FQTHLPAQRGPDRPEGIGEEGGERDRDRSDPLVNGFLALIWSDLRSLCLFSYHRLRDLLL**	
1	FQTHLPAQRGPDRPEGIGEEGGERDRDRSDPLVNGFLALIWSDLRSLCLFSYHRLRDLLL	766
2	FQTHLPAQRGPDRPEGIGEEGGERDRDRSDPLVNGFLALIWSDLRSLCLFSYHRLRDLLL	766
3	FQTHLPAQRGPDRPEGIGEEGGERDRDRSDPLVNGFLALIWSDLRSLCLFSYHRLRDLLL	766
4	FQTHLPAQRGPDRPEGIGEEGGERDRDRSDPLVNGFLALIWSDLRSLCLFSYHRLRDLLL	766
5	FQTHLPAQRGPDRPEGIGEEGGERDRDRSDPLVNGFLALIWSDLRSLCLFSYHRLRDLLL	766
6	FQTHLPAQRGPDRPEGIGEEGGERDRDRSDPLVNGFLALIWSDLRSLCLFSYHRLRDLLL	766
7	FQTHLPAQRGPDRPEGIGEEGGERDRDRSDPLVNGFLTLIWSDLRSLCLFSYHRLRDLLL	766
8	FQTHLPAQRGPDRPEGIGEEGGERDRDRSDPLVNGFLALIWSDLRSLCLFSYHRLRDLLL	766
9	FQTHLPAQRGPDRPEGIGEEGGERDRDRSDPLVNGFLALIWSDLRSLCLFSYHRLRDLLL	766
10	FQTHLPAQRGPDRPEGIGEEGGERDRDRSDPLVNGFLALIWSDLRSLCLFSYHRLRDLLL	766
11	FQTHLPAQRGPDRPEGIGEEGGERDRDRSDPLVNGFLALIWSDLRSLCLFSYHRLRDLLL	766
12	FQTHLPAQRGPDRPEGIGEEGGERDRDRSDPLVNGFLALIWSDLRSLCLFSYHRLRDLLL	766
13	FQTHLPAQRGPDRPEGIGEEGGERDRDRSDPLVNGFLALIWSDLRSLCLFSYHRLRDLLL	766
14	FQTHLPAQRGPDRPEGIGEEGGERDRDRSDPLVNGFLALIWSDLRSLCLFSYHRLRDLLL	766
15	FQTHLPAQRGPDRPEGIGEEGGERDRDRSDPLVNGFLALIWSDLRSLCLFSYHRLRDLLL	766
16	FQTHLPAQRGPDRPEGIGEEGGERDRDRSDPLVNGFLALIWSDLRSLCLFSYHRLRDLLL	766
17	FQTHLPAQRGPDRPEGIGEEGGERDRDRSDPLVNGFLALIWSDLRSLCLFSYHRLRDLLL	766
18	FQTHLPAQRGPDRPEGIGEEGGERDRDRSDPLVNGFLALIWSDLRSLCLFSYHRLRDLLL	764
19	FQTHLPAQRGPDRPEGIGEEGGERDRDRSDPLVNGFLALIWSDLRSLCLFSYHRLRDLLL	765
20	FQTHLPAQRGPDRPEGIGEEGGERDRDRSDPLVNGFLTLIWSDLRSLCLFSYHRLRDLLL	765
21	FQTHLPAQRGPDRPEGIGEEGGKRDRDRSDPLVNGFLALIWSDLRSLCLFSYHRLRDLLL	766
22	FQTHLPAQRGPDRPEGIGEEGGERDRDRSDPLVNGFLALIWSDLRSLCLFSYHRLRDLLL	766
23	FQTHLPAQRGPDRPEGIGEEGGERDRDRSDPLVNGFLALIWSDLRSLCLFSYHRLRDLLL	766
24	FQTHLPAQRGPDRPEGIGEEGGERDRDRSDPLVNGFLALIWSDLRSLCLFSYHRLRDLLL	766
25	FQTHLPAQRGPDRPEGIGEEGGERDRDRSDPLVNGFLALIWSDLRSLCLFSYHRLRDLLL	765
26	FQTHLPAQRGPDRPEGIGEEGGERDRDRSDPLVNGFLALIWSDLRSLCLFSYHRLRDLLL	766
27	FQTHLPAQRGPDRPEGIGEEGGERDRDRSDPLVNGFLTLIWSDLRSLCLFSYHRLRDLLL	766
28	FQTHLPAQRGPDRPEGIGEEGGERDRDRSDPLVNGFLALIWSDLRSLCLFSYHRLRDLLL	766
29	FQTHLPAQRGPDRPEGIGEEGGERDRDRSDPLVNGFLTLIWSDLRSLCLFSYHRLRDLLL	766
30	FQTHLPAQRGPDRPEGIGEEGGERDRDRSDPLVNGFLALIWSDLRSLCLFSYHRLRDLLL	766
31	FQTHLPAQRGPDRPEGIGEEGGERDRDRSDPLVNGFLTLIWSDLRSLCLFSYHRLRDLLL	766
32	FQTHLPAQRGPDRPEGIGEEGGERDRDRSDPLVNGFLALIWSDLRSLCLFSYHRLRDLLL	766
33	FQTHLPAQRGPDRPEGIGEEGGERDRDRSDPLVNGFLALIWSDLRSLCLFSYHRLRDLLL	766

	IVTRIVELLGR-------RGWEVLKYWWNLLQYWSQELKNSAVSLLNATAIAVAEGTDRV	
1	IVTRIVELLGR-------RGWEVLKYWWNLLQYWSQELKNSAVSLLNATAIAVAEGTDRV	819
2	IVTRIVELLGR-------RGWEVLKYWWNLLQYWSQELKNSAVSLLNATAIAVAEGTDRV	819
3	IVTRIVELLGR-------RGWEVLKYWWNLLQYWSQELKNSAVSLLNATAIAVAEGTDRV	819
4	IVTRIVELLGR-------RGWEVLKYWWNLLQYWSQELKNSAVSLLNATAIAVAEGTDRV	819
5	IVTRIVELLGR-------RGWEVLKYWWNLLQYWSQELKNSAVSLLNATAIAVAEGTDRV	819
6	IVTRIVELLGR-------RGWEVLKYWWNLLQYWSQELKNSAVSLLNATAIAVAEGTDRV	819
7	IVTRIVELLGR-------RGWEVLKYWWNLLQYWSQELKNSAVSLLNATAIAVAEGTDRV	819
8	IVTRIVELLGR-------RGWEVLKYWWNLLQYWSQELKNSAVSLLNATAIAVAEGTDRV	819
9	IVTRIVELLGR-------RGWEVLKYWWNLLQYWSQELKNSAVSLLNATAIAVAEGTDRV	819
10	IVTRIVELLGR-------RGWEVLKYWWNLLQYWSQELKNSAVSLLNATAIAVAEGTDRV	819
11	IVTRIVELLGR-------RGWEVLKYWWNLLQYWSQELKNSAVSLLNATAIAVAEGTDRV	819
12	IVTRIVELLGR-------RGWEVLKYWWNLLQYWSQELKNSAVSLLNATAIAVAEGTDRV	819
13	IVTRIVELLGR-------RGWEVLKYWWNLLQYWSQELKNSAVSLLNATAIAVAEGTDRV	819
14	IVTRIVELLGR-------RGWEVLKYWWNLLQYWSQELKNSAVSLLNATAIAVAEGTDRV	819
15	IVTRIVELLGR-------RGWEVLKYWWNLLQYWSQELKNSAVSLLNATAIAVAEGTDRV	819
16	IVTRIVELLGR-------RGWEVLKYWWNLLQYWSQELKNSAVSLLNATAIAVAEGTDRV	819
17	IVTRIVELLGR-------RGWEVLKYWWNLLQYWSQELKNSAVSLLNATAIAVAEGTDRV	819
18	IVTRIVELLGR-------RGWEVLKYWWNLLQYWSQELKNSAVSLLNATAIAVAEGTDRV	817
19	IVTRIVELLGR-------RGWEVLKYWWNLLQYWSQELKNSAVSLLNATAIAVAEGTDRV	818
20	IVTRIVELLGR-------RGWEVLKYWWNLLQYWSQELKNSAVSLLNATAIAVAEGTDRV	818
21	IVTRIVELLGR-------RGWEVLKYWWNLLQYWSQELKNSAVSLLNATAIAVAEGTDRV	819
22	IVTRIVELLGR-------RGWEVLKYWWNLLQYWSQELKNSAVSLLNATAIAVAEGTDRV	819
23	IVTRIVELLGR-------RGWEVLKYWWNLLQYWSQELKNSAVSLLNATAIAVAEGTDRV	819
24	IVTRIVELLGR-------RGWEVLKYWWNLLQYWSQELKNSAVSLLNATAIAVAEGTDRV	819
25	IVTRIVELLGR-------RGWEVLKYWWNLLQYWSQELKNSAVSLLNATAIAVAEGTDRV	818
26	IVTRIVELLGR-------RGWEVLKYWWNLLQYWSQELKNSAVSLLNATAIAVAEGTDRV	819
27	IVTRIVELLGR-------RGWEVLKYWWNLLQYWSQELKNSAVNLLNATAIAVAEGTDRV	819
28	IVTRIVELLGR-------RGWEVLKYWWNLLQYWSQELKNSAVSLLNATAIAVAEGTDRV	819
29	IVTRIVELLGR-------RGWEVLKYWWNLLQYWSQELKNSAVSLLNATAIAVAEGTDRV	819
30	IVTRIVELLGR-------RGWEVLKYWWNLLQYWSQELKNSAVSLLNATAIAVAEGTDRV	819
31	IVTRIVELLGR-------RGWEVLKYWWNLLQYWSQELKNSAVSLLNATAIAVAEGTDRV	819
32	IVTRIVELLGR-------RGWEVLKYWWNLLQYWSQELKNSAVSLLNATAIAVAEGTDRV	819
33	IVTRIVELLGR-------RGWEVLKYWWNLLQYWSQELKNSAVSLLNATAIAVAEGTDRV	819

IEVVQRACRAILHIPRRIRQGLERALL

1	IEVVQRACRAILHIPRRIRQGLERALL	846
2	IEVVQRACRAILHIPRRIRQGLERALL	846
3	IEVVQRACRAILHIPRRIRQGLERALL	846
4	IEVVQRACRAILHIPRRIRQGLERALL	846
5	VEVVQRACRAILHIPRRIRQGLERALL	846
6	IEVVQRACRAILHIPRRIRQGLERALL	846
7	IEVVQRACRAILHIPRRIRQGLERALL	846
8	IEVVQRACRAILHIPRRIRQGLERALL	846
9	IEVVQRACSAILHIPRRIRQGLERALL	846
10	IEVVQRACRAILHIPRRIRQGLERALL	846
11	IEVVQRACRAILHIPRRIRQGLERALL	846
12	IEVVQRACRAILHIPRRIRQGLERALL	846
13	IEVVQRACRAILHIPRRIRQGLERALL	846
14	IEVVQRACRAILHIPRRIRQGLERALL	846
15	IEVVQRACRAILHIPRRIRQGLERALL	846
16	IEVVQRACRAILHIPRRIRQGLERALL	846
17	IEVVQRACRAILHIPRRIRQGLERALL	846
18	IEVVQRACRAILHIPRRIRQGLERALL	844
19	IEVVQRACRAILHIPRRIRQGLERALL	845
20	IEVVQRACRATLHIPRRIRQGLERALL	845
21	IEVVQRACRAILHIPRRIRQGLERALL	846
22	IEVVQRACRAILHIPRRIRQGLERALL	846
23	IEVVQRACRAILHIPRRIRQGLERALL	846
24	IEVVQRACRAILHIPRRIRQGLERALL	846
25	IEVVQRACRAILHIPRRIRQGLERALL	845
26	IEVVQRACRAILHIPRRIRQGLERALL	846
27	IEVVQRACRATLHIPRRIRQGLERALL	846
28	IEVVQRACRAILHIPRRIRQGLERALL	846
29	IEVVQRACRATLHIPRRIRQGLERALL	846
30	IEVVQRACRAILHIPRRIRQGLERALL	846
31	IEVVQRACRAILHIPRRIRQGLERALL	846
32	IEVVQRACRAILHIPRRIRQGLERALL	846
33	IEVVQRACRAILHIPRRIRQGLERALL	846

Aligned using an external algorithm

Consensus

1. B.US.2006.700010040_C9_4520.EU289193
2. B.US.2007.CH0040_3_d0299_ipe032_2_01.MG900232
3. B.US.2007.CH0040_3_d0299_ipe032_2_04.MG900233
4. B.US.2007.CH0040_3_d0299_ipe032_2_05.MG900234
5. B.US.2007.CH0040_3_d0299_ipe032_2_06.MG900235
6. B.US.2007.CH0040_3_d0299_ipe032_2_08.MG900236
7. B.US.2007.CH0040_3_d0299_ipe032_2_09.MG900237
8. B.US.2007.CH0040_3_d0299_ipe032_2_10.MG900238
9. B.US.2007.CH0040_3_d0299_ipe032_2_13.MG900239
10. B.US.2007.CH0040_3_d0299_ipe032_2_16.MG900240
11. B.US.2007.CH0040_3_d0299_ipe032_2_18.MG900241
12. B.US.2007.CH0040_3_d0299_ipe032_2_19.MG900242
13. B.US.2007.CH0040_3_d0299_ipe032_2_20.MG900243
14. B.US.2007.CH0040_3_d0299_ipe032_2_21.MG900244
15. B.US.2007.CH0040_3_d0299_ipe032_2_23.MG900245
16. B.US.2007.CH0040_3_d0299_ipe032_2_24.MG900246
17. B.US.2007.CH0040_3_d0299_ipe032_2_25.MG900247
18. B.US.2007.CH0040_3_d0299_ipe032_2_27.MG900248
19. B.US.2007.CH0040_3_d0299_ipe032_2_29.MG900249
20. B.US.2007.CH0040_3_d0299_ipe032_2_30.MG900250
21. B.US.2007.CH0040_3_d0299_ipe032_2_33.MG900251
22. B.US.2007.CH0040_3_d0299_ipe032_2_35.MG900252
23. B.US.2007.CH0040_3_d0299_ipe032_2_37.MG900253
24. B.US.2007.CH0040_3_d0299_ipe032_2_38.MG900254
25. B.US.2007.CH0040_3_d0299_ipe032_2_39.MG900255
26. B.US.2007.CH0040_3_d0299_ipe032_2_42.MG900256
27. B.US.2007.CH0040_3_d0299_ipe032_2_43.MG900257
28. B.US.2007.CH0040_3_d0299_ipe032_2_45.MG900258
29. B.US.2007.CH0040_3_d0299_ipe032_2_47.MG900259
30. B.US.2007.CH0040_3_d0299_ipe032_2_48.MG900260
31. B.US.2007.CH0040_3_d0299_ipe032_2_49.MG900261
32. B.US.2007.CH0040_3_d0299_ipe032_2_51.MG900262
33. B.US.2007.CH0040_3_d0299_ipe032_2_53.MG900263
34. B.US.2007.CH0040_3_d0299_ipe032_2_55.MG900264
35. B.US.2007.CH0040_3_d0299_ipe032_2_57.MG900265
36. B.US.2007.CH0040_3_d0299_ipe032_2_58.MG900266
37. B.US.2007.CH0040_3_d0299_ipe032_2_59.MG900267
38. B.US.2007.CH0040_3_d0299_ipe032_2_60.MG900268
39. B.US.2007.CH0040_3_d0299_ipe032_2_61.MG900269
40. B.US.2007.CH0040_3_d0299_ipe032_3_40.MG900270

	**MRVMGIRKNYQHLWREGILLLGILMICSAADNLWVTVYYGVPVWREATTTLFCASDAKAY**	
1	MRVMGIRKNYQHLWREGILLLGILMICSAADNLWVTVYYGVPVWREATTTLFCASDAKAY	60
2	MRVMGIRKNYQHLWREGILLLRILMICSAADNLWVTVYYGVPVWREATTTLFCASDAKAY	60
3	MRVMGIRKNYQHLWREGILLLGILMICSAADNLWVTVYYGVPVWREATTTLFCASDAKAY	60
4	MRVMGIRKNYQHLWREGILLLGILMICSAADNLWVTVYYGVPVWREATTTLFCASDAKAY	60
5	MRVMGIRKNYQHLWREGILLLGILMICSAADNLWVTVYYGVPVWREATTTLFCASDAKAY	60
6	MRVMGIRKNYQHLWREGILLLGILMICSAADNLWVTVYYGVPVWREATTTLFCASDAKAY	60
7	MRVMGIRKNYQHLWREGILLLGILMICSAADNLWVTVYYGVPVWREATTTLFCASDAKAY	60
8	MRVMGIRKNYQHLWREGILLLGILMICSAADNLWVTVYYGVPVWREATTTLFCASDAKAY	60
9	MRVMGIRKNYQHLWREGILLLGILMICSAADNLWVTVYYGVPVWREATTTLFCASDAKAY	60
10	MRVMGIRKNYQHLWREGILLLGILMICSAADNLWVTVYYGVPVWREATTTLFCASDAKAY	60
11	MRVMGIRKNYQHLWREGILLLGILMICSAADNLWVTVYYGVPVWREATTTLFCASDAKAY	60
12	MRVMGIRKNYQHLWREGILLLGILMICSAADNLWVTVYYGVPVWREATTTLFCASDAKAY	60
13	MRVMGIRKNYQHLWREGILLLGILMICSAADNLWVTVYYGVPVWREATTTLFCASDAKAY	60
14	MRVMGIRKNYQHLWREGILLLGILMICSAADNLWVTVYYGVPVWREATTTLFCASDAKAY	60
15	MRVMGIRKNYQHLWREGILLLGILMICSAADNLWVTVYYGVPVWREATTTLFCASDAKAY	60
16	MRVMGIRKNYQHLWREGILLLGILMICSAADNLWVTVYYGVPVWREATTTLFCASDAKAY	60
17	MRVMGIRKNYQHLWREGILLLGILMICSAADNLWVTVYYGVPVWREATTTLFCASDAKAY	60
18	MRVMGIRKNYQHLWREGILLLGILMICSAADNLWVTVYYGVPVWREATTTLFCASDAKAY	60
19	MRVMGIRKNYQHLWREGILLLGILMICSAADNLWVTVYYGVPVWREATTTLFCASDAKAY	60
20	MRVMGIRKNYQHLWREGILLLGILMICSAADNLWVTVYYGVPVWREATTTLFCASDAKAY	60
21	MRVMGIRKNYQHLWREGILLLGILMICSAADNLWVTVYYGVPVWREATTTLFCASDAKAY	60
22	MRVMGIRKNYQHLWREGILLLGILMICSAADNLWVTVYYGVPVWREATTTLFCASDAKAY	60
23	MRVMGIRKNYQHLWREGILLLGILMICSAADNLWVTVYYGVPVWREATTTLFCASDAKAY	60
24	MRVMGIRKNYQHLWREGILLLGILMICSAADNLWVTVYYGVPVWREATTTLFCASDAKAY	60
25	MRVMGIRKNYQHLWREGILLLGILMICSAADNLWVTVYYGVPVWREATTTLFCASDAKAY	60
26	MRVMGIRKNYQHLWREGILLLGILMICSAADNLWVTVYYGVPVWREATTTLFCASDAKAY	60
27	MRVMGIRKNYQHLWREGILLLGILMICSAADNLWVTVYYGVPVWREATTTLFCASDAKAY	60
28	MRVMGIRKNYQHLWREGILLLGILMICSAADNLWVTVYYGVPVWREATTTLFCASDAKAY	60
29	MRVMGIRKNYQHLWREGILLLGILMICSAADNLWVTVYYGVPVWREATTTLFCASDAKAY	60
30	MRVMGIRKNYQHLWREGILLLGILMICSAADNLWVTVYYGVPVWREATTTLFCASDAKAY	60
31	MRVMGIRKNYQHLWREGILLLGILMICSAADNLWVTVYYGVPVWREATTTLFCASDAKAY	60
32	MRVMGIRKNYQHLWREGILLLGILMICSAADNLWVTVYYGVPVWREATTTLFCASDAKAY	60
33	MRVMGIRKNYQHLWREGILLLGILMICSAADNLWVTVYYGVPVWREATTTLFCASDAKAY	60
34	MRVMGIRKNYQHLWREGILLLGILMICSAADNLWVTVYYGVPVWREATTTLFCASDAKAY	60
35	MRVMGIRKNYQHLWREGILLLGILMICSAADNLWVTVYYGVPVWREATTTLFCASDAKAY	60
36	MRVMGIRKNYQHLWREGILLLGILMICSAADNLWVTVYYGVPVWREATTTLFCASDAKAY	60
37	MRVMGIRKNYQHLWREGILLLGILMICSAADNLWVTVYYGVPVWREATTTLFCASDAKAY	60
38	MRVMGIRKNYQHLWREGILLLGILMICSAADNLWVTVYYGVPVWREATTTLFCASDAKAY	60
39	MRVMGIRKNYQHLWREGILLLGILMICSAADNLWVTVYYGVPVWREATTTLFCASDAKAY	60
40	MRVMGIRKNYQHLWREGILLLGILMICSAADNLWVTVYYGVPVWREATTTLFCASDAKAY	60

	DTEAHNVWATHACVPTDPNPQEVELKNVTENFNMWENNMVEQMHEDIISLWDQSLKPCVK	
1	DTEAHNVWATHACVPTDPNPQEVELKNVTENFNMWENNMVEQMHEDIISLWDQSLKPCVK	120
2	DTEAHNV-ATHACVPTDPNPQEVELKNVTENFNMWENNMVEQMHEDIISLWDQSLKPCVK	119
3	DTEAHNVWATHACVPTDPNPQEVELKNVTENFNMWENNMVEQMHEDIISLWDQSLKPCVK	120
4	DTEAHNVWATHACVPTDPNPQEVELKNVTENFNMWENNMVEQMHEDIISLWDQSLKPCVK	120
5	DTEAHNVWATHACVPTDPNPQEVELKNVTENFNMWENNMVEQMHEDIISLWDQSLKPCVK	120
6	DTEAHNVWATHACVPTDPNPQEVELKNVTENFNMWENNMVEQMHEDIISLWDQSLKPCVK	120
7	DTEAHNVWATHACVPTDPNPQEVELKNVTENFNMWENNMVEQMHEDIISLWDQSLKPCVK	120
8	DTEAHNVWATHACVPTDPNPQEVELKNVTENFNMWENNMVEQMHEDIISLWDQSLKPCVK	120
9	DTEAHNVWATHACVPTDPNPQEVELKNVTENFNMWENNMVEQMHEDIISLWDQSLKPCVK	120
10	DTEAHNVWATHACVPTDPNPQEVELKNVTENFNMWENNMVEQMHEDIISLWDQSLKPCVK	120
11	DTEAHNVWATHACVPTDPNPQEVELKNVTENFNMWENNMVEQMHEDIISLWDQSLKPCVK	120
12	DTEAHNVWATHACVPTDPNPREVELKNVTENFNMWENNMVEQMHEDIISLWDQSLKPCVK	120
13	DTEAHNVWATHACVPTDPNPQEVELKNVTENFNMWENNMVEQMHEDIISLWDQSLKPCVK	120
14	DTEAHNVWATHACVPTDPNPQEVELKNVTENFNMWENNMVEQMHEDIISLWDQSLKPCVK	120
15	DTEAHNVWATHACVPTDPNPQEVELKNVTENFNMWENNMVEQMHEDIISLWDQSLKPCVK	120
16	DTEAHNVWATHACVPTDPNPQEVELKNVTENFNMWENNMVEQMHEDIISLWDQSLKPCVK	120
17	DTEAHNVWATHACVPTDPNPQEVELKNVTENFNMWENNMVEQMHEDIISLWDQSLKPCVK	120
18	DTEAHNVWATHACVPTDPNPQEVELKNVTENFNMWENNMVEQMHEDIISLWDQSLKPCVK	120
19	DTEAHNVWATHACVPTDPNPQEVELKNVTENFNMWENNMVEQMHEDIISLWDQSLKPCVK	120
20	DTEAHNVWATHACVPTDPNPQEVELKNVTENFNMWENNMVEQMHEDIISLWDQSLKPCVK	120
21	DTEAHNVWATHACVPTDPNPQEVELKNVTENFNMWENNMVEQMHEDIISLWDQSLKPCVK	120
22	DTEAHNVWATHACVPTDPNPQEVELKNVTENFNMWENNMVEQMHEDIISLWDQSLKPCVK	120
23	DTEAHNVWATHACVPTDPNPQEVELKNVTENFNMWENNMVEQMHEDIISLWDQSLKPCVK	120
24	DTEAHNVWATHACVPTDPNPQEVELKNVTENFNMWENNMVEQMHEDIISLWDQSLKPCVK	120
25	DTEAHNVWATHACVPTDPNPQEVELKNVTENFNMWENNMVEQMHEDIISLWDQSLKPCVK	120
26	DTEAHNVWATHACVPTDPNPQEVELKNVTENFNMWENNMVEQMHEDIISLWDQSLKPCVK	120
27	DTEAHNVWATHACVPTDPNPQEVELKNVTENFNMWENNMVEQMHEDIISLWDQSLKPCVK	120
28	DTEAHNVWATHACVPTDPNPQEVELKNVTENFNMWENNMVEQMHEDIISLWDQSLKPCVK	120
29	DTEAHNVWATHACVPTDPNPQEVELKNVTENFNMWENNMVEQMHEDIISLWDQSLKPCVK	120
30	DTEAHNVWATHACVPTDPNPQEVELKNVTENFNMWENNMVEQMHEDIISLWDQSLKPCVK	120
31	DTEAHNVWATHACVPTDPNPQEVELKNVTENFNMWENNMVEQMHEDIISLWDQSLKPCVK	120
32	DTEAHNVWATHACVPTDPNPQEVELKNVTENFNMWENNMVEQMHEDIISLWDQSLKPCVK	120
33	DTEAHNVWATHACVPTDPNPQEVELKNVTENFNMWENNMVEQMHEDIISLWDQSLKPCVK	120
34	DTEAHNVWATHACVPTDPNPQEVELKNVTENFNMWENNMVEQMHEDIISLWDQSLKPCVK	120
35	DTEAHNVWATHACVPTDPNPQEVELKNVTENFNMWENNMVEQMHEDIISLWDQSLKPCVK	120
36	DTEAHNVWATHACVPTDPNPQEVELKNVTENFNMWENNMVEQMHEDIISLWDQSLKPCVK	120
37	DTEAHNVWATHACVPTDPNPQEVELKNVTENFNMWENNMVEQMHEDIISLWDQSLKPCVK	120
38	DTEAHNVWATHACVPTDPNPQEVELKNVTENFNMWENNMVEQMHEDIISLWDQSLKPCVK	120
39	DTEAHNVWATHACVPTDPNPQEVELKNVTENFNMWENNMVEQMHEDIISLWDQSLKPCVK	120
40	DTEAHNVWATHACVPTDPNPQEVELKNVTENFNMWENNMVEQMHEDIISLWDQSLKPCVK	120

```
      LTPLCVTLNCTDLGNVTNTT---NSKGXMMEKGEVKNCSFKITTDIKDRTRKEYALFYKL

1     LTPLCVTLNCTDLGNVTNTT---NSNGEMMEKGEVKNCSFKITTDIKDRTRKEYALFYKL   177
2     LTPLCVTLNCTDLGNVTNTT---NSKGEMMEKGEVKNCSFKITTDIKDRTRKEYALFYKL   176
3     LTPLCVTLNCTDLGNVTNTT---NSKGEMMEKGEVKNCSFKITTDIKDRTRKEYALFYKL   177
4     LTPLCVTLNCTDLGNVTNTT---NSNGKMMEKGEVKNCSFKITTDIKDRTRKEYALFYKL   177
5     LTPLCVTLNCTDLGNVTNTT---NSNEKMMEKGEVKNCSFKITTDIKDRTRKEYALFYKL   177
6     LTPLCVTLNCTDLGNVTNTTN--SNGERMMEKGEVKNCSFKITTDIKDRTRKEYALFYKL   178
7     LTPLCVTLNCTDLGNVTNTT---NSKGEMMEKGEVKNCSFKITTDIKDRTRKEYALFYKL   177
8     LTPLCVTLNCTDLGNVTNTA---NSNGTMMEKGEVKNCSFKITTDIKDRTRKEYALFYKL   177
9     LTPLCVTLNCTDLENVTNTT---NSTEKMMEKGEVKNCSFKITTDIKDRTRKEYALFYKL   177
10    LTPLCVTLNCTDLENVTNTT---NSTEKMMEKGEVKNCSFKITTDIKDRTRKEYALFYKL   177
11    LTPLCVTLNCTDLGNVTNTT---NSKGEMMEKGEVKNCSFKITTDIKDRTRKEYALFYKL   177
12    LTPLCVTLNCTDLGNVTNTT---NSKGEMMEKGEVKNCSFKITTDIKDRTRKEYALFYKL   177
13    LTPLCVTLNCTDLENVTNTT---NSNEKMMEKGEVKNCSFKITTDIKDRTRKEYALFYKL   177
14    LTPLCVTLNCTDLGNVTNTT---NSKGEMMEKGEVKNCSFKITTDIKDRTRKEYALFYKL   177
15    LTPLCVTLNCTDLGNVTNTTN--SNGERMMEKGEVKNCSFKITTDIKDRTRKEYALFYKL   178
16    LTPLCVTLNCTDLGNVTNTT---NSKGEMMEKGEVKNCSFKITTDIKDRTRKEYALFYKL   177
17    LTPLCVTLNCTDLGNVTNTT---NSKGEMMEKGEVKNCSFKITTDIKDRTRKEYALFYKL   177
18    LTPLCVTLNCTDLGNVTNTT---NSKGEMMEKGEVKNCSFKITTDIKDRTRKEYALFYKL   177
19    LTPLCVTLNCTDLGNVTNTT---NSKGGMMEKGEVKNCSFKITTDIKDRTRKEYALFYKL   177
20    LTPLCVTLNCTDLENVTNTT---NSTEKMMEKGEVKNCSFKITTDIKDRTRKEYALFYKL   177
21    LTPLCVTLNCTDLGNVTNTT---NSKGGMMEKGEVKNCSFKITTDIKDRTRKEYALFYKL   177
22    LTPLCVTLNCTDLGNVTNTT---NSKGEMMEKGEVKNCSFKITTDIKDRTRKEYALFYKL   177
23    LTPLCVTLNCTDLGNVTNTT---NSKGEMMEKGEVKNCSFKITTDIKDRTRKEYALFYKL   177
24    LTPLCVTLNCTDLGNVTNTTN--SNGERMMEKGEVKNCSFKITTDIKDRTRKEYALFYKL   178
25    LTPLCVTLNCTDLGNVTNTT---NSKGEMMEKGEVKNCSFKITTDIKDRTRKEYALFYKL   177
26    LTPLCVTLNCTDLGNVTTTT---NSNGTLMEKGEVKNCSFKITTDIKDRTRKEYALFYKL   177
27    LTPLCVTLNCTDLGNVTNTT---NSKGEMMEKGEVKNCSFKITTDIKDRTRKEYALFYKL   177
28    LTPLCVTLNCTDLGNVTNTT---NSKGGMMEKGEVKNCSFKITTDIKDRTRKEYALFYKL   177
29    LTPLCVTLNCTDLGNVTNTT---NSKGGMMEKGEVKNCSFKITTDIKDRTRKEYALFYKL   177
30    LTPLCVTLNCTDLGNVTTTT---NSNGTLMEKGEVKNCSFKITTDIKDRTRKEYALFYKL   177
31    LTPLCVTLNCTDLGNVTNTT---NSKGGMMEKGEVKNCSFKITTDIKDRTRKEYALFYKL   177
32    LTPLCVTLNCTDLENVTNTT---NSNEKMMEKGEVKNCSFKITTDIKDRTRKEYALFYKL   177
33    LTPLCVTLNCTDLENVTNTT---NSTEKMMEKGEVKNCSFKITTDIKDRTRKEYALFYKL   177
34    LTPLCVTLNCTDLGNVTNTT---NSKGGMMEKGEVKNCSFKITTDIKDRTRKEYALFYKL   177
35    LTPLCVTLNCTDLGNVTNTT---NSNGKMMEKGEVKNCSFKITTDIKDRTRKEYALFYKL   177
36    LTPLCVTLNCTDLGNVTNTT---NSNGRMMEKGEVKNCSFKITTDIKDRTRKEYALFYKL   177
37    LTPLCVTLNCTDLGNVTNTT---NSKGEMMEKGEVKNCSFKITTDIKDRTRKEYALFYKL   177
38    LTPLCVTLNCTDLGNVTNTT---NSKGEMMEKGEVRNCSFKITTDIKDRTRKEYALFYKL   177
39    LTPLCVTLNCTDLGNVTNTT---NSKGEMMEKGEVKNCSFKITTDIKDRTRKEYALFYKL   177
40    LTPLCVTLNCTDLGNVTNTT---NSKGGMMEKGEVKNCSFKITTDIKDRTRKEYALFYKL   177
```

	DVVPIND-------TRYRLVSCNTSVITQACPKVSFEPIPIHYCAPAGFAILKCNDKQFI	
1	DVVPIND-------TRYRLVSCNTSVITQACPKVSFEPIPIHYCAPAGFAILKCNDKQFI	230
2	DVVPIND-------TRYRLVSCNTSVITQACPKVSFEPIPIHYCAPAGFAILKCNDKQFI	229
3	DVVPIND-------TRYRLVSCNTSVITQACPKVSFEPIPIHYCAPAGFAILKCNDKQFI	230
4	DVVPIND-------TRYRLVSCNTSVITQACPKVSFEPIPIHYCAPAGFAILKCNDKQFI	230
5	DVVPIND-------TRYRLVSCNTSVITQACPKVSFEPIPIHYCAPAGFAILKCNDKQFI	230
6	DVVPIND-------TRYRLVSCNTSVITQACPKVSFEPIPIHYCAPAGFAILKCNDKQFI	231
7	DVVPIND-------TRYRLVSCNTSVITQACPKVSFEPIPIHYCAPAGFAILKCNDKQFI	230
8	DVVPIND-------TRYRLVSCNTSVITQACPKVSFEPIPIHYCAPAGFAILKCNDKQFI	230
9	DVVPIND-------TRYRLVSCNTSVITQACPKVSFEPIPIHYCAPAGFAILKCNDKQFI	230
10	DVVPIND-------TRYRLVSCNTSVITQACPKVSFEPIPIHYCAPAGFAILKCNDKQFI	230
11	DVVPIND-------TRYRLVSCNTSVITQACPKVSFEPIPIHYCAPAGFAILKCNDKQFI	230
12	DVVPIND-------TRYRLVSCNTSVITQACPKVSFEPIPIHYCAPAGFAILKCNDKQFI	230
13	DVVPIND-------TRYRLVSCNTSVITQACPKVSFEPIPIHYCAPAGFAILKCNDKQFI	230
14	DVVPIND-------TRYRLVSCNTSVITQACPKVSFEPIPIHYCAPAGFAILKCNDKQFI	230
15	DVVPIND-------TRYRLVSCNTSVITQACPKVSFEPIPIHYCAPAGFAILKCNDKQFI	231
16	DVVPIND-------TRYRLVSCNTSVITQACPKVSFEPIPIHYCAPAGFAILKCNDKQFM	230
17	DVVPIND-------TRYRLVSCNTSVITQACPKVSFEPIPIHYCAPAGFAILKCNDKQFI	230
18	DVVPIND-------TRYRLVSCNTSVITQACPKVSFEPIPIHYCAPAGFAILKCNDKQFI	230
19	DVVPIND-------TRYRLVSCNTSVITQACPKVSFEPIPIHYCAPAGFAILKCNDKQFI	230
20	DVVPIND-------TRYRLVSCNTSVITQACPKVSFEPIPIHYCAPAGFAILKCNDKQFI	230
21	DVVPIND-------TRYRLVSCNTSVITQACPKVSFEPIPIHYCAPAGFAILKCNDKQFI	230
22	DVVPIND-------TRYRLVSCNTSVITQACPKVSFEPIPIHYCAPAGFAILKCNDKQFI	230
23	DVVPIND-------TRYRLVSCNTSVITQACPKVSFEPIPIHYCAPAGFAILKCNDKQFI	230
24	DVVPIND-------TRYRLVSCNTSVITQACPKVSFEPIPIHYCAPAGFAILKCNDKQFI	231
25	DVVPIND-------TRYRLVSCNTSVITQACPKVSFEPIPIHYCAPAGFAILKCNDKQFI	230
26	DVVPIND-------TRYRLVSCNTSVITQACPKVSFEPIPIHYCAPAGFAILKCNDKQFI	230
27	DVVPIND-------TRYRLVSCNTSVITQACPKVSFEPIPIHYCAPAGFAILKCNDKQFI	230
28	DVVPIND-------IRYRLVSCNTSVITQACPKVSFEPIPIHYCAPAGFAILKCNDKQFI	230
29	DVVPIND-------TRYRLVSCNTSVITQACPKVSFEPIPIHYCAPAGFAILKCNDKQFI	230
30	DVVPIND-------TRYRLVSCNTSVITQACPKVSFEPIPIHYCAPAGFAILKCNDKQFI	230
31	DVVPIND-------TRYRLVSCNTSVITQACPKVSFEPIPIHYCAPAGFAILKCNDKQFI	230
32	DVVPIND-------TRYRLVSCNTSVITQACPKVSFEPIPIHYCAPAGFAILKCNDKQFI	230
33	DVVPIND-------TRYRLVSCNTSVITQACPKVSFEPIPIHYCAPAGFAILKCNDKQFI	230
34	DVVPIND-------TRYRLVSCNTSVITQACPKVSFEPIPIHYCAPAGFAILKCNDKQFI	230
35	DVVPIND-------TRYRLVSCNTSVITQACPKVSFEPIPIHYCAPAGFAILKCNDKQFI	230
36	DVVPIND-------TRYRLVSCNTSVITQACPKVSFEPIPIHYCAPAGFAILKCNDKQFI	230
37	DVVPIND-------TRYRLVSCNTSVITQACPKVSFEPIPIHYCAPAGFAILKCNDKQFI	230
38	DVVPIND-------TRYRLVSCNTSVITQACPKVSFEPIPIHYCAPAGFAILKCNDKQFI	230
39	DVVPIND-------TRYRLVSCNTSVITQACPKVSFEPIPIHYCAPAGFAILKCNDKQFI	230
40	DVVPIND-------TRYRLVSCNTSVITQACPKVSFEPIPIHYCAPAGFAILKCNDKQFI	230

	**GTGPCTNVSTVQCTHGIRPVVSTQLLLNGSLAEEEVVIRSVNFSDNAKTIIVQLNKSVEI**	
1	GTGPCTNVSTVQCTHGIRPVVSTQLLLNGSLAEEEVVIRSVNFSDNAKTIIVQLNKSVEI	290
2	GTGPCTNVSTVQCTHGIRPVVSTQLLLNGSLAEEEVVIRSVNFSDNAKTIIVQLNKSVEI	289
3	GTGPCTNVSTVQCTHGIRPVVSTQLLLNGSLAEEEVVIRSVNFSDNAKTIIVQLNKSVEI	290
4	GTGPCTNVSTVQCTHGIRPVVSTQLLLNGSLAEEEVVIRSVNFSDNAKTIIVQLNKSVEI	290
5	GTGPCTNVSTVQCTHGIRPVVSTQLLLNGSLAEEEVVIRSVNFSDNAKTIIVQLNKSVEI	290
6	GTGPCTNVSTVQCTHGIRPVVSTQLLLNGSLAEEEVVIRSVNFSDNAKTIIVQLNKSVEI	291
7	GTGPCTNVSTVQCTHGIRPVVSTQLLLNGSLAEEEVVIRSVNFSDNAKTIIVQLNKSVEI	290
8	GTGPCTNVSTVQCTHGIRPVVSTQLLLNGSLAEEEVVIRSVNFSDNAKTIIVQLNKSVEI	290
9	GTGPCTNVSTVQCTHGIRPGVSTQLLLNGSLAEEEVVIRSVNFSDNAKTIIVQLNKSVEI	290
10	GTGPCTNVSTVQCTHGIRPVVSTQLLLNGSLAEEEVVIRSVNFSDNAKTIIVQLNKSVEI	290
11	GTGPCTNVSTVQCTHGIRPVVSTQLLLNGSLAEEEVVIRSVNFSDNAKTIIVQLNKSVEI	290
12	GTGPCTNVSTVQCTHGIRPVVSTQLLLNGSLAEEEVVIRSVNFSDNAKTIIVQLNKSVEI	290
13	GTGPCTNVSTVQCTHGIRPVVSTQLLLNGSLAEEEVVIRSVNFSDNAKTIIVQLNKSVEI	290
14	GTGPCTNVSTVQCTHGIRPVVSTQLLLNGSLAEEEVVIRSVNFSDNAKTIIVQLNKSVEI	290
15	GTGPCTNVSTVQCTHGIRPVVSTQLLLNGSLAEEEVVIRSVNFSDNAKTIIVQLNKSVEI	291
16	GTGPCTNVSTVQCTHGIRPVVSTQLLLNGSLAEEEVVIRSVNFSDNAKTIIVQLNKSVEI	290
17	GTGPCTNVSTVQCTHGIRPVVSTQLLLNGSLAEEEVVIRSVNFSDNAKTIIVQLNKSVEI	290
18	GTGPCTNVSTVQCTHGIRPVVSTQLLLNGSLAEEEVVIRSVNFSDNAKTIIVQLNKSVEI	290
19	GTGPCTNVSTVQCTHGIRPVVSTQLLLNGSLAEEEVVIRSVNFSDNAKTIIVQLNKSVEI	290
20	GTGPCTNVSTVQCTHGIRPVVSTQLLLNGSLAEEEVVIRSVNFSDNAKTIIVQLNKSVEI	290
21	GTGPCTNVSTVQCTHGIRPVVSTQLLLNGSLAEEEVVIRSVNFSDNAKTIIVQLNKSVEI	290
22	GTGPCTNVSTVQCTHGIRPVVSTQLLLNGSLAEEEVVIRSVNFSDNAKTIIVQLNKSVEI	290
23	GTGPCTNVSTVQCTHGIRPVVSTQLLLNGSLAEEEVVIRSVNFSDNAKTIIVQLNKSVEI	290
24	GTGPCTNVSTVQCTHGIRPVVSTQLLLNGSLAEEEVVIRSVNFSDNAKTIIVQLNKSVEI	291
25	GTGPCTNVSTVQCTHGIRPVVSTQLLLNGSLAEEEVVIRSVNFSDNAKTIIVQLNKSVEI	290
26	GTGPCTNVSTVQCTHGIRPVVSTQLLLNGSLAEEEVVIRSVNFSDNAKTIIVQLNKSVEI	290
27	GTGPCTNVSTVQCTHGIRPVVSTQLLLNGSLAEEEVVIRSVNFSDNAKTIIVQLNKSVEI	290
28	GTGPCTNVSTVQCTHGIRPVVSTQLLLNGSLAEEEVVIRSVNFSDNAKTIIVQLNKSVEI	290
29	GTGPCTNVSTVQCTHGIRPVVSTQLLLNGSLAEEEVVIRSVNFSDNAKTIIVQLNKSVEI	290
30	GTGPCTNVSTVQCTHGIRPVVSTQLLLNGSLAEEEVVIRSVNFSDNAKTIIVQLNKSVEI	290
31	GTGPCTNVSTVQCTHGIRPVVSTQLLLNGSLAEEEVVIRSVNFSDNAKTIIVQLNKSVEI	290
32	GTGPCTNVSTVQCTHGIRPVVSTQLLLNGSLAEEEVVIRSVNFSDNAKTIIVQLNKSVEI	290
33	GTGPCTNVSTVQCTHGIRPVVSTQLLLNGSLAEEEVVIRSVNFSDNAKTIIVQLNKSVEI	290
34	GTGPCTNVSTVQCTHGIRPVVSTQLLLNGSLAEEEVVIRSVNFSDNAKTIIVQLNKSVEI	290
35	GTGPCTNVSTVQCTHGIRPVVSTQLLLNGSLAEEEVVIRSVNFSDNAKTIIVQLNKSVEI	290
36	GTGPCTNVSTVQCTHGIRPVVSTQLLLNGSLAEEEVVIRSVNFSDNAKTIIVQLNKSVEI	290
37	GTGPCTNVSTVQCTHGIRPVVSTQLLLNGSLAEEEVVIRSVNFSDNAKTIIVQLNKSVEI	290
38	GTGPCTNVSTVQCTHGIRPVVSTQLLLNGSLAEEEVVIRSVNFSDNAKTIIVQLNKSVEI	290
39	GTGPCTNVSTVQCTHGIRPVVSTQLLLNGSLAEEEVVIRSVNFSDNAKTIIVQLNKSVEI	290
40	GTGPCTNVSTVQCTHGIRPVVSTQLLLNGSLAEEEVVIRSVNFSDNAKTIIVQLNKSVEI	290

	NCTRPHNNTRKSIPMGPGKAFYARGDITGDIRKAYCEINGTEWHSTLKLVVEKLREQY-N	
1	TCTRPNNNTRKSIPMGPGKAFYARGDITGDIRKAYCEINGTEWHSTLKLVVEKLREQY-N	349
2	NCTRPHNNTRKSIPMGPGKAFYARGDIIGDIRKAYCEINGTEWHSTLKLVVEKLREQY-N	348
3	NCTRPHNNTRKSIPMGPGKAFYARGDIIGDIRKAYCEINGTEWHSTLKLVVEKLREQY-N	349
4	NCTRPHNNTRKSIPMGPGKAFYARGDITGDIRKAYCEINGTEWHSTLKLVVEKLREQY-N	349
5	NCTRPHNNTRKSIPMGPGKAFYARGDITGDIRKAYCEINGTEWHSTLKLVVEKLREQY-N	349
6	TCTRPNNNTRKSIPMGPGKAFYARGDIIGDIRKAYCKINGTEWHSTLKLVVEKLREQY-N	350
7	NCTRPHNNTRKSIPMGPGKAFYARGDIIGDIRKAYCEINGTEWHSTLKLVVEKLREQY-N	349
8	NCTRPNNNTRKSIPMGPGKAFYARGDITGDIRKAYCEINGTEWHSTLKLVVEKLREQY-N	349
9	NCTRPHNNTRKSIPMGPGKAFYARGDITGDIRKAYCEINGTEWHSTLKLVVEKLREQY-N	349
10	NCTRPHNNTRKSIPMGPGKAFYARGDITGDIRKAYCEINGTEWHSTLKLVVEKLREQY-N	349
11	NCTRPHNNTRKSIPMGPGKAFYARGDIIGDIRKAYCEINGTEWHSTLKLVVEKLREQY-N	349
12	NCTRPHNNTRKSIPMGPGKAFYARGDITGDIRKAYCEINGTEWHSTLKLVVEKLREQY-N	349
13	NCTRPHNNTRKSIPMGPGKAFYARGDITGDIRKAYCEINGTEWHSTLKLVVEKLREQY-N	349
14	NCTRPHNNTRKSIPMGPGKAFYARGDITGDIRKAYCEINGTEWHSTLKLVVEKLREQY-N	349
15	TCTRPNNNTRKSIPMGPGKAFYARGDIIGDIRKAYCKINGTEWHSTLKLVVEKLREQY-N	350
16	TCTRPNNNTRKSIPMGPGKAFYARGDIIGDIRKASCKINGTEWHSTLKLVVEKLREQY-N	349
17	NCTRPHNNTRKSIPMGPGKAFYARGDITGDIRKAYCEINGTEWHSTLKLVVEKLREQY-N	349
18	NCTRPHNNTRKSIPMGPGKAFYARGDIIGDIRKAYCEINGTEWHSTLKLVVEKLREQY-N	349
19	NCTRPHNNTRKSIPMGPGKAFYARGDITGDIRKAYCEINGTEWHSTLKLVVEKLREQY-N	349
20	NCTRPHNNTRKSIPMGPGKAFYARGDITGDIRKAYCEINGTEWHSTLKLVVEKLREQY-N	349
21	NCTRPHNNTRKSIPMGPGKAFYARGDITGDIRKAYCEINGTEWHSTLKLVVEKLREQY-N	349
22	NCTRPHNNTRKSIPMGPGKAFYARGDIIGDIRKAYCEINGTEWHSTLKLVVEKLREQY-N	349
23	NCTRPHNNTRKSIPMGPGKAFYARGDIIGDIRKAYCEINGTEWHSTLKLVVEKLREQY-N	349
24	TCTRPNNNTRKSIPMGPGKAFYARGDIIGDIRKAYCKINGTEWHSTLKLVVEKLREQY-N	350
25	NCTRPHNNTRKSIPMGPGKAFYARGDIIGDIRKAYCEINGTEWHSTLKLVVEKLREQY-N	349
26	NCTRPNNNTRKSIPMGPGKAFYARGDITGDIRKAYCEINGTEWHSTLKLVVEKLREQY-N	349
27	NCTRPHNNTRKSIPMGPGKAFYARGDITGDIRKAYCEINGTEWHSTLKLVVEKLREQY-N	349
28	NCTRPHNNTRKSIPMGPGKAFYARGDITGDIRKAYCEINGTEWHSTLKLVVEKLREQY-N	349
29	NCTRPHNNTRKSIPMGPGKAFYARGDITGDIRKAYCEINGTEWHSTLKLVVEKLREQY-N	349
30	NCTRPHNNTRKSIPMGPGKAFYARGDITGDIRKAYCEINGTEWHSTLKLVVEKLREQY-N	349
31	NCTRPHNNTRKSIPMGPGKAFYARGDITGDIRKAYCEINGTEWHSTLKLVVEKLREQY-N	349
32	NCTRPHNNTRKSIPMGPGKAFYARGDITGDIRKAYCEINGTEWHSTLKLVVEKLREQY-N	349
33	NCTRPHNNTRKSIPMGPGKAFYARGDITGDIRKAYCEINGTEWHSTLKLVVEKLREQY-N	349
34	NCTRPHNNTRKSIPMGPGKAFYARGDITGDIRKAYCEINGTEWHSTLKLVVEKLREQY-N	349
35	NCTRPNNNTRKSIPMGPGKAFYARGDITGDIRKAYCEINGTEWHSTLKLVVEKLREQY-N	349
36	TCTRPNNNTRKSIPMGPGKAFYARGDIIGDIRKASCEINGTEWHSTLKLVVEKLREQY-N	349
37	NCTRPHNNTRKSIPMGPGKAFYARGDIIGDIRKAYCEINGTEWHSTLKLVVEKLREQY-N	349
38	NCTRPHNNTRKSIPMGPGKAFYARGDIIGDIRKAYCEINGTEWHSTLKLVVEKLREQY-N	349
39	NCTRPHNNTRKSIPMGPGKAFYARGDITGDIRKAYCEINGTEWHSTLKLVVEKLREQY-N	349
40	NCTRPHNNTRKSIPMGPGKAFYARGDITGDIRKAYCEVNGTEWHSTLKLVVEKLREQY-N	349

	Sequence	
	KTIVFNRSSGGDPEIVMYSFNCGGEFFYCNSTKLFNSTWPWND-TKGSHDTNGTLILPCK	
1	KTIVFNRSSGGDPEIVMYSFNCGGEFFYCNSTKLFNSTWPWND-TKGSHDTNGTLILPCK	408
2	KTIVFNRSSGGDPEIVMYSFNCGGEFFYCNSTKLFNSTWPWND-TKGSHDTNGTLILPCK	407
3	KTIVFNRSSGGDPEIVMYSFNCGGEFFYCNSTKLFNSTWPWND-TKGSHDTNGTLILPCK	408
4	KTIVFNRSSGGDPEIVMYSFNCGGEFFYCNSTKLFNSTWPWND-TKGSHDTNDTLMLPCK	408
5	KTIVFNRSSGGDPEIVMYSFNCGGEFFYCNSTKLFNSTWPWND-TKGSHDTNDTLILPCK	408
6	KTIVFNRSSGGDPEIVMYSFNCGGEFFYCNSTKLFNSTWPWND-TKGSHDTNDTLMLPCK	409
7	KTIVFNRSSGGDPEIVMYSFNCGGEFFYCNSTKLFNSTWPWND-TKGSHDTNGTLILPCK	408
8	KTIVFNRSSGGDPEIVMYSFNCGGEFFYCNSTKLFNSTWPWND-TKGSHDTNGTLTLPCK	408
9	KTIVFNRSSGGDPEIVMYSFNCGGEFFYCNSTKLFNSTWPWND-TKGSHDTNGKLILPCK	408
10	KTIVFNRSSGGDPEIVMYSFNCGGEFFYCNSTKLFNSTWPWND-TKGSHDTNGKLILPCK	408
11	KTIVFNRSSGGDPEIVMYSFNCGGEFFYCNSTKLFNSTWPWND-TKGSHDTNGTLILPCK	408
12	KTIVFNRSSGGDPEIVMYSFNCGGEFFYCNSTKLFNSTWPWND-TKGSHDTNGTLILPCK	408
13	KTIVFNRSSGGDPEIVMYSFNCGGEFFYCNSTKLFNSTWPWND-TKGSHDTNGTLILPCK	408
14	KTIVFNRSSGGDPEIVMYSFNCGGEFFYCNSTKLFNSTWPWND-TKGSHDTNGTLILPCK	408
15	KTIVFNRSSGGDPEIVMYSFNCGGEFFYCNSTKLFNSTWPWND-TKGSHDTNDTLMLPCK	409
16	KTIVFNRSSGGDPEIVMYSFNCGGEFFYCNSTKLFNSTWPWND-TKGSHDTNGTLILPCK	408
17	KTIVFNRSSGGDPEIVMYSFNCGGEFFYCNSTKLFNSTWPWND-TKGSHDTNDTLILPCK	408
18	KTIVFNRSSGGDPEIVMYSFNCGGEFFYCNSTKLFNSTWPWND-TKGSHDTNGTLILPCK	408
19	KTIVFNRSSGGDPEIVMYSFNCGGEFFYCNSTKLFNSTWPWND-TKGSHDTNGTLILPCK	408
20	KTIVFNRSSGGDPEIVMHSFNCGGEFFYCNSTKLFNSTWPWND-TKGSHDTNGKLILPCK	408
21	KTIVFNRSSGGDPEIVMYSFNCGGEFFYCNSTKLFNSTWPWND-TKGSHDTNGTLILPCK	408
22	KTIVFNRSSGGDPEIVMYSFNCGGEFFYCNSTKLFNSTWPWND-TKGSHDTNGTLILPCK	408
23	KTIVFNRSSGGDPEIIMYSFNCGGEFFYCNSTKLFNSTWPWND-TKGSHDTNGTLILPCK	408
24	KTIVFNRSSGGDPEIVMYSFTCGGEFFYCNSTKLFNSTWPWND-TKGSHDTNDTLMLPCK	409
25	KTIVFNRSSGGDPEIVMYSFNCGGEFFYCNSTKLFNSTWPWND-TKGSHDTNGTLILPCK	408
26	KTIVFNRSSGGDPEIVMYSFNCGGEFFYCNSTKLFNSTWPWND-TKGSHDTNGTLILPCK	408
27	KTIVFNRSSGGDPEIVMYSFNCGGEFFYCNSTKLFNSTWPWND-TKGSHDTNGTLILPCK	408
28	KTIVFNRSSGGDPEIVMYSFNCGGEFFYCNSTKLFNSTWPWND-TKGSHDTNGTLILPCK	408
29	KTIVFNRSSGGDPEIVMYSFNYGGEFFYCNSTKLFNSTWPWND-TKGSHDTNGTLILPCK	408
30	KTIVFNRSSGGDPEIVMYSFNCGGEFFYCNSTKLFNSTWPWND-TKGSHDTNGTLILPCK	408
31	KTIVFNRSSGGDPEIVMYSFNCGGEFFYCNSTKLFNSTWPWND-TKGSHDTNGTLILPCK	408
32	KTIVFNRSSGGDPEIVMYSFNCGGEFFYCNSTKLFNSTWPWND-TKGSHDTNGTLILPCK	408
33	KTIVFNRSSGGDPEIVMYSFNCGGEFFYCNSTKLFNSTWPWND-TKGSHDTNGKLILPCK	408
34	KTIVFNRSSGGDPEIVMYSFNCGGEFFYCNSTKLFNSTWPWND-TKGSHDTNGTLILPCK	408
35	KTIVFNRSSGGDPEIVMYSFNCGGEFFYCNSTKLFNSTWPWND-TKGSHDTNDTLMLPCK	408
36	KTIVFNRSSGGDPEIVMYSFNCGGEFFYCNSTKLFNSTWPWND-TKGSHDTNGTLILPCK	408
37	KTIVFNRSSGGDPEIVMYSFNCGGEFFYCNSTKLFNSTWPWND-TKGSHDTNGTLILPCK	408
38	KTIVFNRSSGGDPEIVMYSFNCGGEFFYCNSTKLFNSTWPWND-TKGSHDTNGTLILPCK	408
39	KTIVFNRSSGGDPEIVMYSFNCGGEFFYCNSTKLFNSTWPWND-TKGSHDTNGTLILPCK	408
40	KTIVFNRSSGGDPEIVMYSFNCGGEFFYCNSTKLFNSTWPWND-TKGSHDTNGTLILPCK	408

```
   IKQIINMWQGVGKAMYAPPIEGKIRCSSNITGLLLTRDGG-YESNETDEIFRPGGGDMRD

1  IKQIINMWQGVGKAMYAPPIEGKIRCSSNITGLLLTRDGG-YESNETDEIFRPGGGDMRD 467
2  IKQIINMWQGVGKAMYAPPIEGKIRCSSNITGLLLTRDGG-YESNETDEIFRPGGGDMRD 466
3  IKQIINMWQGVGKAMYAPPIEGKIRCSSNITGLLLTRDGG-YESNETDEIFRPGGGDMRD 467
4  IKQIINMWQGVGKAMYAPPIEGKIRCSSNITGLLLTRDGG-YESNKTDEIFRPGGGDMRD 467
5  IKQIINMWQGVGKAMYAPPIEGKIRCSSNITGLLLTRDGG-YESNETDEIFRPGGGDMRD 467
6  IKQIINMWQGVGKAMYAPPIEGEIRCSSNITGLLLTRDGG-YESNETDEIFRPGGGDMRD 468
7  I-QIINMWQGVGKAMYAPPIEGKIRCSSNITGLLLTRDGG-YESNETDEIFRPGGGDMRD 466
8  IKQIINMWQGVGKAMYAPPIEGQIRCSSNITGLLLTRDGG-YESNETDEIFRPGGGDMRD 467
9  IKQIINMWQGVGKAMYAPPIEGKIRCSSNITGLLLTRDGGYESNETDDEIFRPGGGDMRD 468
10 IKQIINMWQGVGKAMYAPPIEGKIRCSSNITGLLLTRDGG-YESNETDEIFRPGGGDMRD 467
11 IKQIINMWQGVGKAMYAPPIEGKIRCSSNITGLLLTRDGG-YESNETDEIFRPGGGDMRD 467
12 IKQIINMWQGVGKAMYAPPIEGKIRCSSNITGLLLTRDGG-YESNETDEIFRPGGGDMRD 467
13 IKQIINMWQGVGKAMYAPPIEGKIRCSSNITGLLLTRDGG-YESNETDEIFRPGGGDMRD 467
14 IKQIINMWQGVGKAMYAPPIEGKIRCSSNITGLLLARDGG-YESNETDEIFRPGGGDMRD 467
15 IKQIINMWQGVGKAMYAPPIEGEIRCSSNITGLLLTRDGG-YESNETDEIFRPGGGDMRD 468
16 IKQIINMWQGVGKAMYAPPIEGEIRCSSNITGLLLTRDGG-YESNETDEIFRPGGGDMRD 467
17 IKQIINMWQGVGKAMYAPPIEGKIRCSSNITGLLLTRDGG-YESNETDEIFRPGGGDMRD 467
18 IKQIINMWQGVGKAMYAPPIEGKIRCSSNITGLLLTRDGG-YESNETDEIFRPGGGDMRD 467
19 IKQIINMWQGVGKAMYAPPIEGKIRCSSNITGLLLTRDGG-YESNETDEIFRPGGGDMRD 467
20 IKQIINMWQGVGKAMYAPPIEGKIRCSSNITGLLLTRDGG-YESNETDEIFRPGGGDMRD 467
21 IKQIINMWQGVGKAMYAPPIEGKIRCSSNITGLLLTRDGG-YESNETDEIFRPGGGDMRD 467
22 IKQIINMWQGVGKAMYAPPIEGKIRCSSNITGLLLTRDGG-YESNETDEIFRPGGGDMRD 467
23 IKQIINMWQGVGKAMYAPPIEGKIRCSSNITGLLLTRDGG-YESNETDEIFRPGGGDMRD 467
24 IKQIINMWQGVGKAMYAPPIEGEIRCSSNITGLLLTRDGG-YESNETDEIFRPGGGDMRD 468
25 IKQIINMWQGVGKAMYAPPIEGKIRCSSNITGLLLTRDGG-YESNETDEIFRPGGGDMRD 467
26 IKQIINMWQGVGKAMYAPPIEGKIRCSSNITGLLLTRDGG-YESNKTDEIFRPGGGDMRD 467
27 IKQIINMWQGVGKAMYAPPIEGKIRCSSNITGLLLARDGG-YESNETDEIFRPGGGDMRD 467
28 IKQIINMWQGVGKAMYAPPIEGKIRCSSNITGLLLTRDGG-YESNETDEIFRPGGGDMRD 467
29 IKQIINMWQGVGKAMYAPPIEGKIRCSSNITGLLLTRDGG-YESNETDEIFRPGGGDMRD 467
30 IKQIINMWQGVGKAMYAPPIEGKIRCSSNITGLLLARDGG-YESNETDEIFRPGGGDMRD 467
31 IKQIINMWQGVGKAMYAPPIEGKIRCSSNITGLLSTRDGG-YESNETDEIFRPGGGDMRD 467
32 IKQIINMWQGVGKAMYAPPIEGKIRCSSNITGLLLTRDGG-YESNETDEIFRPGGGDMRD 467
33 IKQIINMWQGVGKAMYAPPIEGKIRCSSNITGLLLTRDGG-YESNETDEIFRPGGGDMRD 467
34 IKQIINMWQGVGKAMYAPPIEGKIRCSSNITGLLSTRDGG-YESNETDEIFRPGGGDMRD 467
35 IKQIINMWQGVGKAMYAPPIEGIIRCSSNITGLLLTRDGG-YESNETDEIFRPGGGDMRD 467
36 IKQIINMWQGVGKAMYAPPIEEKIRCSSNITGLLLTRDGG-YESNETDEIFRPGGGDMRD 467
37 IKQIINMWQGVGKAMYAPPIEGKIRCSSNITGLLLTRDGG-YESNETDEIFRPGGGDMRD 467
38 IKQIINMWQGVGKAMYAPPIEGKIRCSSNITGLLLTRDGG-YESNETDEIFRPGGGDMRD 467
39 IKQIINMWQGVGKAMYAPPIEGKIRCSSNITGLLLARDGG-YESNETDEIFRPGGGDMRD 467
40 IKQIINMWQGVGKAMYAPPIEGKIRCSSNITGLLLTRDGG-YESNETDEIFRPGGGDMRD 467
```

	**NWRSELYKYKVVKIEPLGVAPTKAKRRVVQREKRAFG-LGAVFLGFLGAAGSTMGAASIT**	
1	NWRSELYKYKVVKIEPLGVAPTKAKRRVVQREKRAFG-LGAVFLGFLGAAGSTMGAASIT	526
2	NWRSELYKYKVVKIEPLGVAPTKAKRRVVQREKRAFG-LGAVFLGFLGAAGSTMGAASIT	525
3	NWRSELYKYKVVKIEPLGVAPTKAKRRVVQREKRAFG-LGAVFLGFLGAAGSTMGAASIT	526
4	NWRSELYKYKVVKIEPLGVAPTKAKRRVVQREKRAFG-LGAVFLGFLGAAGSTMGAASIT	526
5	NWRSELYKYKVVKIEPLGVAPTKAKRRVVQREKRAFG-LGAVFLGFLGAAGSTMGAASIT	526
6	NWRSELYKYKVVKIEPLGVAPTKAKRRVVQREKRAFG-LGAVFLGFLGAAGSTMGAASIT	527
7	NWRSELYKYKVVKIEPLGVAPTKAKRRVVQREKRAFG-LGAVFLGFLGAAGSTMGAASIT	525
8	NWRSELYKYKVVKIEPLGVAPTKAKRRVVQREKRAFG-LGAVFLGFLGAAGSTMGAASIT	526
9	NWRSELYKYKVVKIEPLGVAPTKAKRRVVQREKRAFG-LGAVFLGFLGAAGSTMGAASIT	527
10	NWRSELYKYKVVKIEPLGVAPTKAKRRVVQREKRAFG-LGAVFLGFLGAAGSTMGAASIT	526
11	NWRSELYKYKVVKIEPLGVAPTKAKRRVVQREKRAFG-LGAVFLGFLGAAGSTMGAASIT	526
12	NWRSELYKYKVVKIEPLGVAPTKAKRRVVQREKRAFG-LGAVFLGFLGAAGSTMGAASIT	526
13	NWRSELYKYKVVKIEPLGVAPTKAKRRVVQREKRAFG-LGAVFLGFLGAAGSTMGAASIT	526
14	NWRSELYKYKVVKIEPLGVAPTKAKRRVVQREKRAFG-LGAVFLGFLGAAGSTMGAASIT	526
15	NWRSELYKYKVVKIEPLGVAPTKAKRRVVQREKRAFG-LGAVFLGFLGAAGSTMGAASIT	527
16	NWRSELYKYKVVKIEPLGVAPTKAKRRVVQREKRAFG-LGAVFLGFLGAAGSTMGAASIT	526
17	NWRSELYKYKVVKIEPLGVAPTKAKRRVVQREKRAFG-LGAVFLGFLGAAGSTMGAASIT	526
18	NWRSELYKYKVVKIEPLGVAPTKAKRRVVQRE--AFG-LGAVFLGFLGAAGSTMGAASIT	524
19	NWRSELYKYKVVKIEPLGVAPTKAKRRVVQREKRAFG-LGAVFLGFLGAAGSTMGAASIT	526
20	NWRSELYKYKVVKIEPLGVAPTKAKRRVVQREKRAFG-LGAVFLGFLGAAGSTMGAASIT	526
21	NWRSELYKYKVVKIEPLGVAPTKAKRRVVQREKRAFG-LGAVFLGFLGAAGSTMGAASIT	526
22	NWRSELYKYKVVKIEPLGVAPTKAKRRVVQREKRAFG-LGAVFLGFLGAAGSTMGAASIT	526
23	NWRSELYKYKVVKIEPLGVAPTKAKRRVVQREKRAFG-LGAVFLGFLGAAGSTMGAASIT	526
24	NWRSELYKYKVVKIEPLGVAPTKAKRRVVQREKRAFG-LGAVFLGFLGAAGSTMGAASIT	527
25	NWRSELYKYKVVKIEPLGVAPTKAKRRVVQREKRAFG-LGAVFLGFLGAAGSTMGAASIT	526
26	NWRSELYKYKVVKIEPLGVAPTKAKRRVVQREKRAFG-LGAVFLGFLGAAGSTMGAASIT	526
27	NWRSELYKYKVVKIEPLGVAPTKAKRRVVQREKRAFG-LGAVFLGFLGAAGSTMGAASIT	526
28	NWRSELYKYKVVKIEPLGVAPTKAKRRVVQREKRAFG-LGAVFLGFLGAAGSTMGAASIT	526
29	NWRSELYKYKVVKIEPLGVAPTKAKRRVVQREKRAFG-LGAVFLGFLGAAGSTMGAASIT	526
30	NWRSELYKYKVVKIEPLGVAPTKAKRRVVQREKRAFG-LGAVFLGFLGAAGSTMGAASIT	526
31	NWRSELYKYKVVKIEPLGVAPTKAKRRVVQREKRAFG-LGAVFLGFLGAAGSTMGAASIT	526
32	NWRSELYKYKVVKIEPLGVAPTKAKRRVVQREKRAFG-LGAVFLGFLGAAGSTMGAASIT	526
33	NWRSELYKYKVVKIEPLGVAPTKAKRRVVQREKRAFG-LGAVFLGFLGAAGSTMGAASIT	526
34	NWRSELYKYKVVKIEPLGVAPTKAKRRVVQREKRAFG-LGAVFLGFLGAAGSTMGAASIT	526
35	NWRSELYKYKVVKIEPLGVAPTKAKRRVVQREKRAFG-LGAVFLGFLGAAGSTMGAASIT	526
36	NWRSELYKYKVVKIEPLGVAPTKAKRRVVQREKRAFG-LGAVFLGFLGAAGSTMGAASIT	526
37	NWRSELYKYKVVKIEPLGVAPTKAKRRVVQREKRAFG-LGAVFLGFLGAAGSTMGAASIT	526
38	NWRSELYKYKVVKIEPLGVAPTKAKRRVVQREKRAFG-LGAVFLGFLGAAGSTMGAASIT	526
39	NWRSELYKYKVVKIEPLGVAPTKAKRRVVQREKRAFG-LGAVFLGFLGAAGSTMGAASIT	526
40	NWRSELYKYKVVKIEPLGVAPTKAKRRVVQREKRAFG-LGAVFLGFLGAAGSTMGAASIT	526

LTVQARQLLSGIVQQQNNLLRAIEAQQHLLQLTVWGIKQLQARVLAVERYLKDQQLLGIW

1	LTVQARQLLSGIVQQQNNLLRAIEAQQHLLQLTVWGIKQLQARVLAVERYLKDQQLLGIW	586
2	LTVQARQLLSGIVQQQNNLLRAIEAQQHLLQLTVWGIKQLQARVLAVERYLKDQQLLGIW	585
3	LTVQARQLLSGIVQQQNNLLRAIEAQQHLLQLTVWGIKQLQARVLAVERYLKDQQLLGIW	586
4	LTVQARQLLSGIVQQQNNLLRAIEAQQHLLQLTVWGIKQLQARVLAVERYLKDQQLLGIW	586
5	LTVQARQLLSGIVQQQNNLLRAIEAQQHLLQLTVWGIKQLQARVLAVERYLKDQQLLGIW	586
6	LTVQARQLLSGIVQQQNNLLRAIEAQQHLLQLTVWGIKQLQARVLAVERYLKDQQLLGIW	587
7	LTVQARQLLSGIVQQQNNLLRAIEAQQHLLQLTVWGIKQLQARVLAVERYLKDQQLLGIW	585
8	LTVQARQLLSGIVQQQNNLLRAIEAQQHLLQLTVWGIKQLQARVLAVERYLKDQQLLGIW	586
9	LTVQARQLLSGIVQQQNNLLRAIEAQQHLLQLTVWGIKQLQARVLAVERYLKDQQLLGIW	587
10	LTVQARQLLSGIVQQQNNLLRAIEAQQHLLQLTVWGIKQLQARVLAVERYLKDQQLLGIW	586
11	LTVQARQLLSGIVQQQNNLLRAIEAQQHLLQLTVWGIKQLQARVLAVERYLKDQQLLGIW	586
12	LTVQARQLLSGIVQQQNNLLRAIEAQQHLLQLTVWGIKQLQARVLAVERYLKDQQLLGIW	586
13	LTVQARQLLSGIVQQQNNLLRAIEAQQHLLQLTVWGIKQLQARVLAVERYLKDQQLLGIW	586
14	LTVQARQLLSGIVQQQNNLLRAIEAQQHLLQLTVWGIKQLQARVLAVERYLKDQQLLGIW	586
15	LTVQARQLLSGIVQQQNNLLRAIEAQQHLLQLTVWGIKQLQARVLAVERYLKDQQLLGIW	587
16	LTVQARQLLSGIVQQQNNLLRAIEAQQHLLQLTVWGIKQLQARVLAVERYLKDQQLLGIW	586
17	LTVQARQLLSGIVQQQNNLLRAIEAQQHLLQLTVWGIKQLQARVLAVERYLKDQQLLGIW	586
18	LTVQARQLLSGIVQQQNNLLRAIEAQQHLLQLTVWGIKQLQARVLAVERYLKDQQLLGIW	584
19	LTVQARQLLSGIVQQQNNLLRAIEAQQHLLQLTVWGIKQLQARVLAVERYLKDQQLLGIW	586
20	LTVQARQLLSGIVQQQNNLLRAIEAQQHLLQLTVWGIKQLQARVLAVERYLKDQQLLGIW	586
21	LTVQARQLLSGIVQQQNNLLRAIEAQQHLLQLTVWGIKQLQARVLAVERYLKDQQLLGIW	586
22	LTVQARQLLSGIVQQQNNLLRAIEAQQHLLQLTVWGIKQLQARVLAVERYLKDQQLLGIW	586
23	LTVQARQLLSGIVQQQNNLLRAIEAQQHLLQLTVWGIKQLQARVLAVERYLKDQQLLGIW	586
24	LTVQARQLLSGIVQQQNNLLRAIEAQQHLLQLTVWGIKQLQARVLAVERYLKDQQLLGIW	587
25	LTVQARQLLSGIVQQQNNLLRAIEAQQHLLQLTVWGIKQLQARVLAVERYLKDQQLLGIW	586
26	LTVQARQLLSGIVQQQNNLLRAIEAQQHLLQLTVWGIKQLQARVLAVERYLKDQQLLGIW	586
27	LTVQARQLLSGIVQQQNNLLRAIEAQQHLLQLTVWGIKQLQARVLAVERYLKDQQLLGIW	586
28	LTVQARQLLSGIVQQQNNLLRAIEAQQHLLQLTVWGIKQLQARVLAVERYLKDQQLLGIW	586
29	LTVQARQLLSGIVQQQNNLLRAIEAQQHLLQLTVWGIKQLQARVLAVERYLKDQQLLGIW	586
30	LTVQARQLLSGIVQQQNNLLRAIEAQQHLLQLTVWGIKQLQARVLAVERYLKDQQLLGIW	586
31	LTVQARQLLSGIVQQQNNLLRAIEAQQHLLQLTVWGIKQLQARVLAVERYLKDQQLLGIW	586
32	LTVQARQLLSGIVQQQNNLLRAIEAQQHLLQLTVWGIKQLQARVLAVERYLKDQQLLGIW	586
33	LTVQARQLLSGIVQQQNNLLRAIEAQQHLLQLTVWGIKQLQARVLAVERYLKDQQLLGIW	586
34	LTVQARQLLSGIVQQQNNLLRAIEAQQHLLQLTVWGIKQLQARVLAVERYLKDQQLLGIW	586
35	LTVQARQLLSGIVQQQNNLLRAIEAQQHLLQLTVWGIKQLQARVLAVERYLKDQQLLGIW	586
36	LTVQARQLLSGIVQQQNNLLRAIEAQQHLLQLTVWGIKQLQARVLAVERYLKDQQLLGIW	586
37	LTVQARQLLSGIVQQQNNLLRAIEAQQHLLQLTVWGIKQLQARVLAVERYLKDQQLLGIW	586
38	LTVQARQLLSGIVQQQNNLLRAIEAQQHLLQLTVWGIKQLQARVLAVERYLKDQQLLGIW	586
39	LTVQARQLLSGIVQQQNNLLRAIEAQQHLLQLTVWGIKQLQARVLAVERYLKDQQLLGIW	586
40	LTVQARQLLSGIVQQQNNLLRAIEAQQHLLQLTVWGIKQLQARVLAVERYLKDQQLLGIW	586

	**GCSGKLICTTTVPWNTSWSNKSLEQIWDNMTWMEWEREIDNYTGYIYQLIEESQNQQEKN**	
1	GCSGKLICTTTVPWNTSWSNKSLEQIWDNMTWMEWEREIDNYTGYIYQLIEESQNQQEKN	646
2	GCSGKLICTTTVPWNTSWSNKSLEQIWDNMTWMEWEREIDNYTGYIYQLIEESQNQQEKN	645
3	GCSGKLICTTTVPWNTSWSNKSLEQIWDNMTWMEWEREIDNYTGYIYQLIEESQNQQEKN	646
4	GCSGKLICTTTVPWNTSWSNKSLEQIWDNMTWMEWEREIDNYTGYIYQLIEESQNQQEKN	646
5	GCSGKLICTTTVPWNTSWSNKSLEQIWDNMTWMEWEREIDNYTGYIYQLIEESQNQQEKN	646
6	GCSGKLICTTTVPWNTSWSNKSLEQIWDNMTWMEWEREIDNYTGYIYQLIEESQNQQEKN	647
7	GCSGKLICTTTVPWNTSWSNKSLEQIWDNMTWMEWEREIDNYTGYIYQLIEESQNQQEKN	645
8	GCSGKLICTTTVPWNTSWSNKSLEQIWDNMTWMEWEREIDNYTGYIYQLIEESQNQQEKN	646
9	GCSGKLICTTTVPWNTSWSNKSLEQIWDNMTWMEWEREIDNYTGYIYQLIEESQNQQEKN	647
10	GCSGKLICTTTVPWNTSWSNKSLEQIWDNMTWMEWEREIDNYTGYIYQLIEESQNQQEKN	646
11	GCSGKLICTTTVPWNTSWSNKSLEQIWDNMTWMEWEREIDNYTGYIYQLIEESQNQQEKN	646
12	GCSGKLICTTTVPWNTSWSNKSLEQIWDNMTWMEWEREIDNYTGYIYQLIEESQNQQEKN	646
13	GCSGKLICTTTVPWNTSWSNKSLEQIWDNMTWMEWEREIDNYTGYIYQLIEESQNQQEKN	646
14	GCSGKLICTTTVPWNTSWSNKSLEQIWDNMTWMEWEREIDNYTGYIYQLIEESQNQQEKN	646
15	GCSGKLICTTTVPWNTSWSNKSLEQIWDNMTWMEWEREIDNYTGYIYQLIEESQNQQEKN	647
16	GCSGKLICTTTVPWNTSWSNKSLEQIWDNMTWMEWEREIDNYTGYIYQLIEESQNQQEKN	646
17	GCSGKLICTTTVPWNTSWSNKSLEQIWDNMTWMEWEREIDNYTGYIYQLIEESQNQQEKN	646
18	GCSGKLICTTTVPWNTSWSNKSLEQIWDNMTWMEWEREIDNYTGYIYQLIEESQNQQEKN	644
19	GCSGKLICTTTVPWNTSWSNKSLEQIWDNMTWMEWEREIDNYTGYIYQLIEESQNQQEKN	646
20	GCSGKLICTTTVPWNTSWSNKSLEQIWDNMTWMEWEREIDNYTGYIYQLIEESQNQQEKN	646
21	GCSGKLICTTTVPWNTSWSNKSLEQIWDNMTWMEWEREIDNYTGYIYQLIEESQNQQEKN	646
22	GCSGKLICTTTVPWNTSWSNKSLEQIWDNMTWMEWKREIDNYTGYIYQLIEESQNQQEKN	646
23	GCSGKLICTTTVPWNTSWSNKSLEQIWDNMTWMEWEREIDNYTGYIYQLIEESQNQQEKN	646
24	GCSGKLICTTTVPWNTSWSNKSLEQIWDNMTWMEWEREIDNYTGYIYQLIEESQNQQEKN	647
25	GCSGKLICTTTVPWNTSWSNKSLEQIWDNMTWMEWEREIDNYTGYIYQLIEESQNQQEKN	646
26	GCSGKLICTTTVPWNTSWSNKSLEQIWDNMTWMEWEREIDNYTGYIYQLIEESQNQQEKN	646
27	GCSGKLICTTTVPWNTSWSNKSLEQIWDNMTWMEWEREIDNYTGYIYQLIEESQNQQEKN	646
28	GCSGKLICTTTVPWNTSWSNKSLEQIWDNMTWMEWEREIDNYTGYIYQLIEESQNQQEKN	646
29	GCSGKLICTTTVPWNTSWSNKSLEQIWDNMTWMEWEREIDNYTGYIYQLIEESQNQQEKN	646
30	GCSGKLICTTTVPWNTSWSNKSLEQIWDNMTWMEWEREIDNYTGYIYQLIEESQNQQEKN	646
31	GCSGKLICTTTVPWNTSWSNKSLEQIWDNMTWMEWEREIDNYTGYIYQLIEESQNQQEKN	646
32	GCSGKLICTTTVPWNTSWSNKSLEQIWDNMTWMEWEREIDNYTGYIYQLIEESQNQQEKN	646
33	GCSGKLICTTTVPWNTSWSNKSLEQIWDNMTWMEWEREIDNYTGYIYQLIEESQNQQEKN	646
34	GCSGKLICTTTVPWNTSWSNKSLEQIWDNMTWMEWEREIDNYTGYIYQLIEESQNQQEKN	646
35	GCSGKLICTTTVPWNTSWSNKSLEQIWDNMTWMEWEREIDNYTGYIYQLIEESQNQQEKN	646
36	GCSGKLICTTTVPWNTSWSNKSLEQIWDNMTWMEWEREIDNYTGYIYQLIEESQNQQEKN	646
37	GCSGKLICTTTVPWNTSWSNKSLEQIWDNMTWMEWEREIDNYTGYIYQLIEESQNQQEKN	646
38	GCSGKLICTTTVPWNTSWSNKSLEQIWDNMTWMEWEREIDNYTGYIYQLIEESQNQQEKN	646
39	GCSGKLICTTTVPWNTSWSNKSLEQIWDNMTWMEWEREIDNYTGYIYQLIEESQNQQEKN	646
40	GCSGKLICTTTVPWNTSWSNKSLEQIWDNMTWMEWEREIDNYTGYIYQLIEESQNQQEKN	646

	**EQELLALDKWASLWNWFDITNWLWYIKIFIMIVGGLIGLRIVFTVLSIVNRVRQGYSPLS**	
1	EQELLALDKWASLWNWFDITNWLWYIKIFIMIVGGLIGLRIVFTVLSIVNRVRQGYSPLS	706
2	EQELLALDKWASLWNWFDITNWLWYIKIFIMIVGGLIGLRIVFTVLSIVNRVRQGYSPLS	705
3	EQELLALDKWASLWNWFDITNWLWYIKIFIMIVGGLIGLRIVFTVLSIVNRVRQGYSPLS	706
4	EQELLALDKWASLWNWFDITNWLWYIKIFIMIVGGLIGLRIVFTVLSIVNRVRQGYSPLS	706
5	EQELLALDKWASLWNWFDITNWLWYIKIFIMIVGGLIGLRIVFTVLSIVNRVRQGYSPLS	706
6	EQELLALDKWASLWNWFDITNWLWYIKIFIMIVGGLIGLRIVFTVLSIVNRVRQGYSPLS	707
7	EQELLALDKWASLWNWFDITNWLWYIKIFIMIVGGLIGLRIVFTVLSIVNRVRQGYSPLS	705
8	EQELLALDKWASLWNWFDITNWLWYIKIFIMIVGGLIGLRIVFTVLSIVNRVRQGYSPLS	706
9	EQELLALDKWASLWNWFDITNWLWYIKIFIMIVGGLIGLRIVFTVLSIVNRVRQGYSPLS	707
10	EQELLALDKWASLWNWFDITNWLWYIKIFIMIVGGLIGLRIVFTVLSIVNRVRQGYSPLS	706
11	EQELLALDKWASLWNWFDITNWLWYIKIFIMIVGGLIGLRIVFTVLSIVNRVRQGYSPLS	706
12	EQELLALDKWASLWNWFDITNWLWYIKIFIMIVGGLIGLRIVFTVLSIVNRVRQGYSPLS	706
13	EQELLALDKWASLWNWFDITNWLWYIKIFIMIVGGLIGLRIVFTVLSIVNRVRQGYSPLS	706
14	EQELLALDKWASLWNWFDITNWLWYIKIFIMIVGGLIGLRIVFTVLSIVNRVRQGYSPLS	706
15	EQELLALDKWASLWNWFDITNWLWYIKIFIMIVGGLIGLRIVFTVLSIVNRVRQGYSPLS	707
16	EQELLALDKWASLWNWFDITNWLWYIKIFIMIVGGLIGLRIVFTVLSIVNRVRQGYSPLS	706
17	EQELLALDKWASLWNWFDITNWLWYIKIFIMIVGGLIGLRIVFTVLSIVNRVRQGYSPLS	706
18	EQELLALDKWASLWNWFDITNWLWYIKIFIMIVGGLIGLRIVFTVLSIVNRVRQGYSPLS	704
19	EQELLALDKWASLWNWFDITNWLWYIKIFIMIVGGLIGLRIVFTVLSIVNRVRQGYSPLS	706
20	EQELLALDKWASLWNWFDITNWLWYIKIFIMIVGGLIGLRIVFTVLSIVNRVRQGYSPLS	706
21	EQELLALDKWASLWNWFDITNWLWYIKIFIMIVGGLIGLRIVFTVLSIVNRVRQGYSPLS	706
22	EQELLALDKWASLWNWFDITNWLWYIKIFIMIVGGLIGLRIVFTVLSIVNRVRQGYSPLS	706
23	EQELLALDKWASLWNWFDITNWLWYIKIFIMIVGGLIGLRIVFTVLSIVNRVRQGYSPLS	706
24	EQELLALDKWASLWNWFDITNWLWYIKIFIMIVGGLIGLRIVFTVLSIVNRVRQGYSPLS	707
25	EQELLALDKWASLWNWFDITNWLWYIKIFIMIVGGLIGLRIVFTVLSIVNRVRQGYSPLS	706
26	EQELLALDKWASLWNWFDITNWLWYIKIFIMIVGGLIGLRIVFTVLSIVKRVRQGYSPLS	706
27	EQELLALDKWASLWNWFDITNWLWYIKIFIMIVGGLIGLRIVFTVLSIVNRVRQGYSPLS	706
28	EQELLALDKWASLWNWFDITNWLWYIKIFIMIVGGLIGLRIVFTVLSIVNRVRQGYSPLS	706
29	EQELLALDKWAGLWNWFDITNWLWYIKIFIMIVGGLIGLRIVFTVLSIVNRVRQGYSPLS	706
30	EQELLALDKWASLWNWFDITNWLWYIKIFIMIVGGLIGLRIVFTVLSIVNRVRQGYSPLS	706
31	EQELLALDKWASLWNWFDITNWLWYIKIFIMIVGGLIGLRIVFTVLSIVNRVRQGYSPLS	706
32	EQELLALDKWASLWNWFDITNWLWYIKIFIMIVGGLIGLRIVFTVLSIVNRVRQGYSPLS	706
33	EQELLALDKWASLWNWFDITNWLWYIKIFIMIVGGLIGLRIVFTVLSIVNRVRQGYSPLS	706
34	EQELLALDKWASLWNWFDITNWLWYIKIFIMIVGGLIGLRIVFTVLSIVNRVRQGYSPLS	706
35	EQELLALDKWASLWNWFDITNWLWYIKIFIMIVGGLIGLRIVFTVLSIVNRVRQGYSPLS	706
36	EQELLALDKWASLWNWFDITNWLWYIKIFIMIVGGLIGLRIVFTVLSIVNRVRQGYSPLS	706
37	EQELLALDKWASLWNWFDITNWLWYIKIFIMIIGGLIGLRIVFTVLSIVNRVRQGYSPLS	706
38	EQELLALDKWASLWNWFDITNWLWYIKIFIMIVGGLIGLRIVFTVLSIVNRVRQGYSPLS	706
39	EQELLALDKWASLWNWFDITNWLWYIKIFIMIVGGLIGLRIVFTVLSIVNRVRQGYSPLS	706
40	EQELLALDKWASLWNWFDITNWLWYIKIFIMIVGGLIGLRIVFTVLSIVNRVRQGYSPLS	706

	**FQTHLPAQRGPDRPEGIGEEGGERDRDRSDPLVNGFLTLIWSDLRSLCLFSYHRLRDLLL**	
1	FQTHLPAQRGPDRPEGIGEEGGERDRDRSDPLVNGFLALIWSDLRSLCLFSYHRLRDLLL	766
2	FQTHLPAQRGPDRPEGIGEEGGERDRDRSDPLVNGFLTLIWSDLRSLCLFSYHRLRDLLL	765
3	FQTHLPAQRGPDRPEGIGEEGGERDRDRSDPLVNGFLTLIWSDLRSLCLFSYHRLRDLLL	766
4	FQTHLPAQRGPDRPEGIGEEGGERDRDRSDPLVNGFLTLIWSDLRSLCLFSYHRLRDLLL	766
5	FQTHLPAQRGPDRPEGIGEEGGERDRDRSDPLVNGFLTLIWSDLRSLCLFSYHRLRDLLL	766
6	FQTHLPAQRGPDRPEGIGEEGGERDRDRSDPLVNGFLTLIWSDLRSLCLFSYHRLRDLLL	767
7	FQTHLPAQRGPDRPEGIGEEGGERDRDRSDPLVNGFLTLIWSDLRSLCLFSYHRLRDLLL	765
8	FQTHLPAQRGPDRPEGIGEEGGERDRDRSDPLVNGFLALIWSELRSLCLFSYHRLRDLLL	766
9	FQTHLPAQRGPDRPEGIGEEGGERDRDRSDPLVNGFLTLIWSDLRSLCLFSYHRLRDLLL	767
10	FQTHLPAQRGPDRPEGIGEEGGERDRDRSDPLVNGFLTLIWSDLRSLCLFSYHRLRDLLL	766
11	FQTHLPAQRGPDRPEGIGEEGGERDRDRSDPLVNGFLTLIWSDLRSLCLFSYHRLRDLLL	766
12	FQTHLPAQRGPDRPEGIGEEGGERDRDRSDPLVNGFLTLIWSDLRSLCLFSYHRLRDLLS	766
13	FQTHLPAQRGPDRPEGIGEEGGERDRDRSDPLVNGFLTLIWSDLRSLCLFSYHRLRDLLL	766
14	FQTHLPAQRGPDRPEGTGEEGGERDRDRSDPLVNGFLTLIWSDLRSLCLFSYHRLRDLLL	766
15	FQTHLPAQRGPDRPEGIGEEGGERDRDRSDPLVNGFLTLIWSDLRSLCLFSYHRLRDLLL	767
16	FQTHLPAQRGPDRPEGIGEEGGERDRDRSDPLVNGFLTLIWSDLRSLCLFSYHRLRDLLL	766
17	FQTHLPAQRGPDRPEGIGEEGGERDRDRSDPLVNGFLTLIWSDLRSLCLFSYHRLRDLLL	766
18	FQTHLPAQRGPDRPEGIGEEGGERDRDRSDPLVNGFLTLIWSDLRSLCLFSYHRLRDLLL	764
19	FQTHLPAQRGPDRPEGIGEEGGERDRDRSDPLVNGFLTLIWSDLRSLCLFSYHRLRDLLL	766
20	FQTHLPAQRGPDRPEGIGEEGGERDRDRSDPLVNGFLTLIWSDLRSLCLFSYHRLRDLLL	766
21	FQTHLPAQRGPDRPEGIGEEGGERDRDRSDPLVNGFLTLIWSDLRSLCLFSYHRLRDLLL	766
22	FQTHLPAQRGPDRPEGIGEEGGERDRDRSDPLVNGFLTLIWSDLRSLCLFSYHRLRDLLL	766
23	FQTHLPAQRGPDRPEGIGEEGGERDRDRSDPLVNGFLTLIWSDLRSLCLFSYHRLRDLLL	766
24	FQTHLPAQRGPDRPEGIGEEGGERDRDRSDPLVNGFLTLIWSDLRSLCLFSYHRLRDLLL	767
25	FQTHLPAQRGPDRPEGIGEEGGERDRDRSDPLVNGFLTLIWSDLRSLCLFSYHRLRDLLL	766
26	FQTHLPAQRGPDRPEGIGEEGGARDRDRSDPLVNGFLALIWSDLRSLCLFSYHRLRDLLL	766
27	FQTHLPAQRGPDRPEGIGEEGGERDRDRSDPLVNGFLALIWSDLRSLCLFSYHRLRDLLL	766
28	FQTHLPAQRGPDRPEGIGEEGGERDRDRSDPLVNGFLTLIWSDLRSLCLFSYHRLRDLLL	766
29	FQTHLPAQRGPDRPEGIGEEGGERDRDRSDPLVNGFLTLIWSDLRSLCLFSYHRLRDLLL	766
30	FQTHLPAQRGPDRPEGIGEEGGERDRDRSDPLVNGFLTLIWSDLRSLCLFSYHRLRDLLL	766
31	FQTHLPAQRGPDRPEGIGEEGGERDRDRSDPLVNGFLTLIWSDLRSLCLFSYHRLRDLLL	766
32	FQTHLPAQRGPDRPEGIGEEGGERDRDRSDPLVNGFLTLIWSDLRSLCLFSYHRLRDLLL	766
33	FQTHLPAQRGPDRPEGIGEEGGERDRDRSDPLVNGFLTLIWSDLRSLCLFSYHRLRDLLL	766
34	FQTHLPAQRGPDRPEGIGEEGGERDRDRSDPLVNGFLTLIWSDLRSLCLFSYHRLRDLLL	766
35	FQTHLPAQRGPDRPEGIGEEGGERDRDRSDPLVNGFLTLIWSDLRSLCLFSYHRLRDLLL	766
36	FQTHLPAQRGPDRPEGIGEEGGERDRDRSDPLVNGFLTLIWSDLRSLCLFSYHRLRDLLL	766
37	FQTHLPAQRGPDRPEGIGEEGGERDRDRSDPLVNGFLTLIWSDLRSLCLFSYHRLRDLLL	766
38	FQTHLPAQRGPDRPEGIGEEGGERDRDRSDPLVNGFLTLIWSDLRSLCLFSYHRLRDLLL	766
39	FQTHLPAQRGPDRPEGIGEEGGERDRDRSDPLVNGFLTLIWSDLRSLCLFSYHRLRDLLL	766
40	FQTHLPAQRGPDRPEGIGEEGGERDRDRSDPLVNGFLTLIWSDLRSLCLFSYHRLRDLLL	766

	IVTRIVELLGR-------RGWEVLKYWWNLLQYWSQELKNSAVSLLNATATAVAEGTDRV	
1	IVTRIVELLGR-------RGWEVLKYWWNLLQYWSQELKNSAVSLLNATAIAVAEGTDRV	819
2	IVTRIVELLGR-------RGWEVLKYWWNLLQYWSQELKNSAVSLLNATATAVAEGTDRV	818
3	IVTRIVELLGR-------RGWEVLKYWWNLLQYWSQELKNSAVSLLNATATAVAEGTDRV	819
4	IVTRIVELLGR-------RGWEVLKYWWNLLQYWSQELKNSAVSLLNATATAVAEGTDRV	819
5	IVTRIVELLGR-------RGWEVLKYWWNLLQYWSQELKNSAVSLLNATATAVAEGTDRV	819
6	IVTRIVELLGR-------RGWEVLKYWWNLLQYWSQELKNSAVSLLNATAIAVAEGTDRV	820
7	IVTRIVELLGR-------RGWEVLKYWWNLLQYWSQELKNSAVSLLNATATAVAEGTDRV	818
8	IVTRIVELLGR-------RGWEVLKYWWNLLQYWSQELKNSAVSLLNATAIAVAEGTDRV	819
9	IVTRIVELLGR-------RGWEVLKYWWNLLQYWSQELKNSAVSLLNATATAVAEGTDRV	820
10	IVTRIVELLGR-------RGWEVLKYWWNLLQYWSQELKNSAVSLLNATATAVAEGTDRV	819
11	IVTRIVELLGR-------RGWEVLKYWWNLLQYWSQELKNSAVSLLNATATAVAEGTDRV	819
12	IVTRIVELLGR-------RGWEVLKYWWNLLQYWSQELKNSAVSLLNATAIAVAEGTDRV	819
13	IVTRIVELLGR-------RGWEVLKYWWNLLQYWSQELKNSAVSLLNATAIAVAEGTDRV	819
14	IVTRIVELLGR-------RGWEVLKYWWNLLQYWSQELKNSAVSLLNATATAVAEGTDRV	819
15	IVTRIVELLGR-------RGWEVLKYWWNLLQYWSQELKNSAVSLLNATAIAVAEGTDRV	820
16	IVTRIVELLGR-------RGWEVLKYWWNLLQYWSQELKNSAVSLLNATATAVAEGTDRV	819
17	IVTRIVELLGR-------RGWEVLKYWWNLLQYWSQELKNSAVSLLNATATAVAEGTDRV	819
18	IVTRIVELLGR-------RGWEVLKYWWNLLQYWSQELKNSAVSLLNATATAVAEGTDRV	817
19	IVTRIVELLGR-------RGWEVLKYWWNLLQYWSQELKNSAVSLLNATATAVAEGTDRV	819
20	IVTRIVELLGR-------RGWEVLKYWWNLLQYWSQELKNSAVSLLNATATAVAEGTDRV	819
21	IVTRIVELLGR-------RGWEVLKYWWNLLQYWSQELKNSAVSLLNATATAVAEGTDRV	819
22	IVTRIVELLGR-------RGWEVLKYWWNLLQYWSQELKNSAVSLLNATATAVAEGTDRV	819
23	IVTRIVELLGR-------RGWEVLKYWWNLLQYWSQELKNSAVSLLNATATAVAEGTDRV	819
24	IVTRIVELLGR-------RGWEVLKYWWNLLQYWSQELKNSAVSLLNATAIAVAEGTDRV	820
25	IVTRIVELLGR-------RGWEVLKYWWNLLQYWSQELKNSAVSLLNATATAVAEGTDRV	819
26	IVTRIVELLGR-------RGWEVLKYWWNLLQYWSQELKNSAVSLLNATAIAVAEGTDRV	819
27	IVTRIVELLGR-------RGWEVLKYWWNLLQYWSQELKNSAVSLLNATAIAVAEGTDRV	819
28	IVTRIVELLGR-------RGWEVLKYWWNLLQYWSQELKNSAVSLLNATATAVAEGTDRV	819
29	IVTRIVELLGR-------RGWEVLKYWWNLLQYWSQELKNSAVSLLNATATAVAEGTDRV	819
30	IVTRIVELLGR-------RGWEVLKYWWNLLQYWSQELKNSAVSLLNATAIAVAEGTDRV	819
31	IVTRIVELLGR-------RGWEVLKYWWNLLQYWSQELKNSAVSLLNATATAVAEGTDRV	819
32	IVTRIVELLGR-------RGWEVLKYWWNLLQYWSQELKNSAVSLLNATATAVAEGTDRV	819
33	IVTRIVELLGR-------RGWEVLKYWWNLLQYWSQELKNSAVSLLNATATAVAEGTDRV	819
34	IVTRIVELLGR-------RGWEVLKYWWNLLQYWSQELKNSAVSLLNATATAVAEGTDRV	819
35	IVTRIVELLGR-------RGWEVLKYWWNLLQYWSQELKNSAVSLLNATAIAVAEGTDRV	819
36	IVTRIVELLGR-------RGWEVLKYWWNLLQYWSQELKNSAVSLLNATAIAVAEGTDRV	819
37	IVTRIVELLGR-------RGWEVLKYWWNLLQYWSQELKNSAVSLLNATATAVAEGTDRV	819
38	IVTRIVELLGR-------RGWEVLKYWWNLLQYWSQELKNSAVSLLNATATAVAEGTDRV	819
39	IVTRIVELLGR-------RGWEVLKYWWNLLQYWSQELKNSAVSLLNATATAVAEGTDRV	819
40	IVTRIVELLGR-------RGWEVLKYWWNLLQYWSQELKNSAVSLLNATATAVAEGTDRV	819

	IEVVQRACRAILHIPRRIRQGLERALL	
1	IEVVQRACRAILHIPRRIRQGLERALL	846
2	IEVVQRACRAILHIPRRIRQGLERALL	845
3	IEVVQRACRAILHIPRRIRQGLERALL	846
4	IEVVQRACRAILHIPRRIRQGLERALL	846
5	IEVVQRACRAILHIPRRIRQGLERALL	846
6	IEVVQGACRAILHIPRRIRQGLERALL	847
7	IEVVQRACRAILHIPRRIRQGLERALL	845
8	IEVVQRACRAILHIPRRIRQGLERALL	846
9	IEVVQRACRAILHIPRRIRQGLERALL	847
10	IEVVQRACRAILHIPRRIRQGLERALL	846
11	IEVVQRACRAILHIPRRIRQGLERALL	846
12	IEVVQRACRAILHIPRRIRQGLERALL	846
13	IEVVQRACRAILHIPRRIRQGLERALL	846
14	IEVVQRACRAILHIPRRIRQGLERALL	846
15	IEVVQGACRAILHIPRRIRQGLERALL	847
16	IEVVQRACRAILHIPRRIRQGLERALL	846
17	IEVVQRACRAILHIPRRIRQGLERALL	846
18	IEVVQRACRAILHIPRRIRQGLERALL	844
19	IEVVQRACRAILHIPRRIRQGLERALL	846
20	IEVVQRACRAILHIPRRIRQGLERALL	846
21	IEVVQRACRAILHIPRRIRQGLERALL	846
22	IEVVQRACRAILHIPRRIRQGLERALL	846
23	IEVVQRACRAILHIPRRIRQGLERALL	846
24	IEVVQGACRAILHIPRRIRQGLERALL	847
25	IEVVQRACRAILHIPRRIRQGLERALL	846
26	IEVVQRACRAILHIPRRIRQGLERALL	846
27	IEVVQRACRAILHIPRRIRQGLERALL	846
28	IEVVQRACRAILHIPRRIRQGLERALL	846
29	IEVVQRACRAILHIPRRIRQGLERALL	846
30	IEVVQRACRAIRHIPRRIRQGLERALL	846
31	IEVVQRACRAILHIPRRIRQGLERALL	846
32	IEVVQRACRAILHIPRRIRQGLERALL	846
33	IEVVQRACRAILHIPRRIRQGLERALL	846
34	IEVVQRACRAILHIPRRIRQGLERALL	846
35	IEVVQRACRAIPHIPRRIRQGLERALL	846
36	IEVVQRVGRAILHIPRRIRQGLERALL	846
37	IEVVQRACRAILHIPRRIRQGLERALL	846
38	IEVVQRACRAILHIPRRIRQGLERALL	846
39	IEVVQRACRAILHIPRRIRQGLERALL	846
40	IEVVQRACRAILHIPRRIRQGLERALL	846

Aligned using an external algorithm

Consensus

1. B.US.2006.700010040_C9_4520.EU289193
2. B.US.2007.CH0040_3_d0428_ipe032_10_17.MG900271
3. B.US.2007.CH0040_3_d0428_ipe032_10_19.MG900272
4. B.US.2007.CH0040_3_d0428_ipe032_10_20.MG900273
5. B.US.2007.CH0040_3_d0428_ipe032_10_22.MG900274
6. B.US.2007.CH0040_3_d0428_ipe032_10_23.MG900275
7. B.US.2007.CH0040_3_d0428_ipe032_10_25.MG900276
8. B.US.2007.CH0040_3_d0428_ipe032_10_26.MG900277
9. B.US.2007.CH0040_3_d0428_ipe032_10_27.MG900278
10. B.US.2007.CH0040_3_d0428_ipe032_10_28.MG900279
11. B.US.2007.CH0040_3_d0428_ipe032_10_29.MG900280
12. B.US.2007.CH0040_3_d0428_ipe032_10_32.MG900281
13. B.US.2007.CH0040_3_d0428_ipe032_10_33.MG900282
14. B.US.2007.CH0040_3_d0428_ipe032_10_34.MG900283
15. B.US.2007.CH0040_3_d0428_ipe032_10_35.MG900284
16. B.US.2007.CH0040_3_d0428_ipe032_10_36.MG900285
17. B.US.2007.CH0040_3_d0428_ipe032_10_37.MG900286
18. B.US.2007.CH0040_3_d0428_ipe032_10_39.MG900287
19. B.US.2007.CH0040_3_d0428_ipe032_10_40.MG900288
20. B.US.2007.CH0040_3_d0428_ipe032_10_41.MG900289
21. B.US.2007.CH0040_3_d0428_ipe032_10_45.MG900290
22. B.US.2007.CH0040_3_d0428_ipe032_10_46.MG900291
23. B.US.2007.CH0040_3_d0428_ipe032_10_48.MG900292
24. B.US.2007.CH0040_3_d0428_ipe032_10_49.MG900293
25. B.US.2007.CH0040_3_d0428_ipe032_10_50.MG900294
26. B.US.2007.CH0040_3_d0428_ipe032_27_15.MG900295
27. B.US.2007.CH0040_3_d0428_ipe032_27_16.MG900296
28. B.US.2007.CH0040_3_d0428_ipe032_2_01.MG900297
29. B.US.2007.CH0040_3_d0428_ipe032_2_05.MG900298
30. B.US.2007.CH0040_3_d0428_ipe032_3_01.MG900299
31. B.US.2007.CH0040_3_d0428_ipe032_3_06.MG900300
32. B.US.2007.CH0040_3_d0428_ipe032_3_07.MG900301
33. B.US.2007.CH0040_3_d0428_ipe032_3_08.MG900302
34. B.US.2007.CH0040_3_d0428_ipe032_3_10.MG900303
35. B.US.2007.CH0040_3_d0428_ipe032_3_11.MG900304
36. B.US.2007.CH0040_3_d0428_ipe032_3_12.MG900305
37. B.US.2007.CH0040_3_d0428_ipe032_3_13.MG900306
38. B.US.2007.CH0040_3_d0428_ipe032_3_14.MG900307

	**MRVMGIRKNYQHLWREGILLLGILMICSAADNLWVTVYYGVPVWREATTTLFCASDAKAY**	
1	MRVMGIRKNYQHLWREGILLLGILMICSAADNLWVTVYYGVPVWREATTTLFCASDAKAY	60
2	MRVMGIRKNYQHLWREGILLLGILMICSAADNLWVTVYYGVPVWREATTTLFCASDAKAY	60
3	MRVMGIRKNYQHLWREGILLLGILMICSAADNLWVTVYYGVPVWREATTTLFCASDAKAY	60
4	MRVMGIRKNYQHLWREGILLLGILMICSVADNLWVTVYYGVPVWREATTTLFCASDAKAY	60
5	MRVMGIRKNYQHLWREGILLLGILMICSAADNLWVTVYYGVPVWREATTTLFCASDAKAY	60
6	MRVMGIRKNYQHLWREGILLLGILMICSAADNLWVTVYYGVPVWREATTTLFCASDAKAY	60
7	MRVMGIRKNYQHLWREGILLLGILMICSAADNLWVTVYYGVPVWREATTTLFCASDAKAY	60
8	MRVMGIRKNYQHLWREGILLLGILMICSAADNLWVTVYYGVPVWREATTTLFCASDAKAY	60
9	MRVMGIRKNYQHLWREGILLLGILMICSAADNLWVTVYYGVPVWREATTTLFCASDAKAY	60
10	MRVMGIRKNYQHLWREGILLLGILMICSAADNLWVTVYYGVPVWREATTTLFCASDAKAY	60
11	MRVMGIRKNYQHLWREGILLLGILMICSAADNLWVTVYYGVPVWREATTTLFCASDAKAY	60
12	MRVMGIRKNYQHLWREGILLLGILMICSAADNLWVTVYYGVPVWREATTTLFCASDAKAY	60
13	MRVMGIRKNYQHLWREGILLLGILMICSAADNLWVTVYYGVPVWREATTTLFCASDAKAY	60
14	MRVMGIRKNYQHLWREGILLLGILMICSAADNLWVTVYYGVPVWREATTTLFCASDAKAY	60
15	MRVMGIRKNYQHLWREGILLLGILMICSAADNLWVTVYYGVPVWREATTTLFCASDAKAY	60
16	MRVMGIRKNYQHLWREGILLLGILMICSAADNLWVTVYYGVPVWREATTTLFCASDAKAY	60
17	MRVMGIRKNYQHLWREGILLLGILMICSAADNLWVTVYYGVPVWREATTTLFCASDAKAY	60
18	MRVMGIRKNYQHLWREGILLLGILMICSAADNLWVTVYYGVPVWREATTTLFCASDAKAY	60
19	MRVMGIRKNYQHLWREGILLLGILMICSAADNLWVTVYYGVPVWREATTTLFCASDAKAY	60
20	MRVMGIRKNYQHLWREGILLLGILMICSAADNLWVTVYYGVPVWREATTTLFCASDAKAY	60
21	MRVMGIRKNYQHLWREGILLLGILMICSAADNLWVTVYYGVPVWREATTTLFCASDAKAY	60
22	MRVMGIRKNYQHLWREGILLLGILMICSAADNLWVTVYYGVPVWREATTTLFCASDAKAY	60
23	MRVMGIRKNYQHLWREGILLLGILMICSAADNLWVTVYYGVPVWREATTTLFCASDAKAY	60
24	MRVMGIRKNYQHLWREGILLLGILMICSAADNLWVTVYYGVPVWREATTTLFCASDAKAY	60
25	MRVMGIRKNYQHLWREGILLLGILMICSAADNLWVTVYYGVPVWREATTTLFCASDAKAY	60
26	MRVMGIRKNYQHLWREGILLLGILMICSAADNLWVTVYYGVPVWREATTTLFCASDAKAY	60
27	MRVMGIRKNYQHLWREGILLLGILMICSAADNLWVTVYYGVPVWREATTTLFCASDAKAY	60
28	MRVMGIRKNYQHLWREGILLLGILMICSAADNLWVTVYYGVPVWREATTTLFCASDAKAY	60
29	MRVMGIRKNYQHLWREGILLLGILMICSAADNLWVTVYYGVPVWREATTTLFCASDAKAY	60
30	MRVMGIRKNYQHLWREGILLLGILMICSAADNLWVTVYYGVPVWREATTTLFCASDAKAY	60
31	MRVMGIRKNYQHLWREGILLLGILMICSAADNLWVTVYYGVPVWREATTTLFCASDAKAY	60
32	MRVMGIRKNYQHLWREGILLLGILMICSAADNLWVTVYYGVPVWREATTTLFCASDAKAY	60
33	MRVMGIRKNYQHLWREGILLLGILMICSAADNLWVTVYYGVPVWREATTTLFCASDAKAY	60
34	MRVMGIRKNYQHLWREGILLLGILMICSAADNLWVTVYYGVPVWREATTTLFCASDAKAY	60
35	MRVMGIRKNYQHLWREGILLLGILMICSAADNLWVTVYYGVPVWREATTTLFCASDAKAY	60
36	MRVMGIRKNYQHLWREGILLLGILMICSAADNLWVTVYYGVPVWREATTTLFCASDAKAY	60
37	MRVMGIRKNYQHLWREGILLLGILMICSAADNLWVTVYYGVPVWREATTTLFCASDAKAY	60
38	MRVMGIRKNYQHLWREGILLLGILMICSAADNLWVTVYYGVPVWREATTTLFCASDAKAY	60

DTEAHNVWATHACVPTDPNPQEVELKNVTENFNMWENNMVEQMHEDIISLWDQSLKPCVK

1	DTEAHNVWATHACVPTDPNPQEVELKNVTENFNMWENNMVEQMHEDIISLWDQSLKPCVK	120
2	DTEAHNVWATHACVPTDPNPQEVELKNVTENFNMWENNMVEQMHEDIISLWDQSLKPCVK	120
3	DTEAHNVWATHACVPTDPNPQEVELKNVTENFNMWENNMVEQMHEDIISLWDQSLKPCVK	120
4	DTEAHNVWATHACVPTDPNPQEVELKNVTENFNMWENNMVEQMHEDIISLWDQSLKPCVK	120
5	DTEAHNVWATHACVPTDPNPQEVELKNVTENFNMWENNMVEQMHEDIISLWDQSLKPCVK	120
6	DTEAHNVWATHACVPTDPNPQEVELKNVTENFNMWENNMVEQMHEDIISLWDQSLKPCVK	120
7	DTEAHNVWATHACVPTDPNPQEVELKNVTENFNMWENNMVEQMHEDIISLWDQSLKPCVK	120
8	DTEAHNVWATHACVPTDPNPQEVELKNVTENFNMWENNMVEQMHEDIISLWDQSLKPCVK	120
9	DTEAHNVWATHACVPTDPNPQEVELKNVTENFNMWENNMVEQMHEDIISLWDQSLKPCVK	120
10	DTEAHNVWATHACVPTDPNPQEVELKNVTENFNMWENNMVEQMHEDIISLWDQSLKPCVK	120
11	DTEAHNVWATHACVPTDPNPQEVELKNVTENFNMWENNMVEQMHEDIISLWDQSLKPCVK	120
12	DTEAHNVWATHACVPTDPNPQEVELKNVTENFNMWENNMVEQMHEDIISLWDQSLKPCVK	120
13	DTEAHNVWATHACVPTDPNPQEVELKNVTENFNMWENNMVEQMHEDIISLWDQSLKPCVK	120
14	DTEAHNVWATHACVPTDPNPQEVELKNVTENFNMWENNMVEQMHEDIISLWDQSLKPCVK	120
15	DTEAHNVWATHACVPTDPNPQEVELKNVTENFNMWENNMVEQMHEDIISLWDQSLKPCVK	120
16	DTEAHNVWATHACVPTDPNPQEVELKNVTENFNMWENNMVEQMHEDIISLWDQSLKPCVK	120
17	DTEAHNVWATHACVPTDPNPQEVELKNVTENFNMWENNMVEQMHEDIISLWDQSLKPCVK	120
18	DTEAHNVWATHACVPTDPNPQEVELKNVTENFNMWENNMVEQMHEDIISLWDQSLKPCVK	120
19	DTEAHNVWATHACVPTDPNPQEVELKNVTENFNMWENNMVEQMHEDIISLWDQSLKPCVK	120
20	DTEAHNVWATHACVPTDPNPQEVELKNVTENFNMWENNMVEQMHEDIISLWDQSLKPCVK	120
21	DTEAHNVWATHACVPTDPNPQEVELKNVTENFNMWENNMVEQMHEDIISLWDQSLKPCVK	120
22	DTEAHNVWATHACVPTDPNPQEVELKNVTENFNMWENNMVEQMHEDIISLWDQSLKPCVK	120
23	DTEAHNVWATHACVPTDPNPQEVELKNVTENFNMWENNMVEQMHEDIISLWDQSLKPCVK	120
24	DTEAHNVWATHACVPTDPNPQEVELKNVTENFNMWENNMVEQMHEDIISLWDQSLKPCVK	120
25	DTEAHNVWATHACVPTDPNPQEVELKNVTENFNMWENNMVEQMHDDIISLWDQSLKPCVK	120
26	DTEAHNVWATHACVPTDPNPQEVELKNVTENFNMWENNMVEQMHEDIISLWDQSLKPCVK	120
27	DTEAHNVWATHACVPTDPNPQEVELKNVTENFNMWENNMVEQMHEDIISLWDQSLKPCVK	120
28	DTEAHNVWATHACVPTDPNPQEVELKNVTENFNMWENNMVEQMHEDIISLWDQSLKPCVK	120
29	DTEAHNVWATHACVPTDPNPQEVELKNVTENFNMWENNMVEQMHEDIISLWDQSLKPCVK	120
30	DTEAHNVWATHACVPTDPNPQEVELKNVTENFNMWENNMVEQMHEDIISLWDQSLKPCVK	120
31	DTEAHNVWATHACVPTDPNPQEVELKNVTENFNMWKNNMVEQMHEDIISLWDQSLKPCVK	120
32	DTEAHNVWATHACVPTDPNPQEVELKNVTENFNMWENNMVEQMHEDIISLWDQSLKPCVK	120
33	DTEAHNVWATHACVPTDPNPQEVELKNVTENFNMWENNMVEQMHEDIISLWDQSLKPCVK	120
34	DTEAHNVWATHACVPTDPNPQEVELKNVTENFNMWENNMVEQMHEDIISLWDQSLKPCVK	120
35	DTEAHNVWATHACVPTDPNPQEVELKNVTENFNMWENNMVEQMHEDIISLWDQSLKPCVK	120
36	DTEAHNVWATHACVPTDPNPQEVELKNVTENFNMWENNMVEQMHEDIISLWDQSLKPCVK	120
37	DTEAHNVWATHACVPTDPNPQEVELKNVTENFNMWENNMVEQMHEDIISLWDQSLKPCVK	120
38	DTEAHNVWATHACVPTDPNPQEVELKNVTENFNMWENNMVEQMHEDIISLWDQSLKPCVK	120

	Sequence	
	LTPLCVTLNCTDLGNVTTTT---NSNGTLMEKGEVKNCSFKITTDIKDRTRKEYALFYKL	
1	LTPLCVTLNCTDLGNVTNTT---NSNGEMMEKGEVKNCSFKITTDIKDRTRKEYALFYKL	177
2	LTPLCVTLNCTDLGNVTTTT---NSNGTLMEKGEVKNCSFKITTDIKDRTRKEYALFYKL	177
3	LTPLCVTLNCTDLGNVTTTT---NSNGTLMEKGEVKNCSFKITTDIKDRTRKEYALFYKL	177
4	LTPLCVTLNCTDLGNVTTTT---NSNGTLMEKGEVKNCSFKITTDIKDRTRKEYALFYKL	177
5	LTPLCVTLNCTDLGNVTTTT---NSNGTLMEKGEVKNCSFKITTDIKDRTRKEYALFYKL	177
6	LTPLCVTLNCTDLGNVTNTT---NSNGGMMEKGEVKNCSFKITTDIKDRTRKEYALFYKL	177
7	LTPLCVTLNCTDLGNVTTTT---NSNGTLMEKGEVKNCSFKITTDIKDRTRKEYALFYKL	177
8	LTPLCVTLNCTDLGNVTTTT---NSNGTLMEKGEVKNCSFKITTDIKDRTRKEYALFYKL	177
9	LTPLCVTLNCTDLGNVTTTT---NSNGTLMEKGEVKNCSFKITTDIKDRTRKEYALFYKL	177
10	LTPLCVTLNCTDLGNVTTTT---NSNGTLMEKGEVKNCSFKITTDIKDRTRKEYALFYKL	177
11	LTPLCVTLNCTDLGNVTTTT---NSNGTLMEKGEVKNCSFKITTDIKDRTRKEYALFYKL	177
12	LTPLCVTLNCTDLGNVTTTT---NSNGTLMEKGEVKNCSFKITTDIKDRTRKEYALFYKL	177
13	LTPLCVTLNCTDLENVTNTT---NSNETMMEKGEVKNCSFKITTDIKDRTRKEYALFYKL	177
14	LTPLCVTLNCTDLGNVTTTT---NSNGTLMEKGEVKNCSFKITTDIKDRTRKEYALFYKL	177
15	LTPLCVTLNCTDLGNVTTTT---NSNGTLMEKGEVKNCSFKITTDIKDRTRKEYALFYKL	177
16	LTPLCVTLNCTDLGNVTTTT---NSNGTLMEKGEVKNCSFKITTDIKDRTRKEYALFYKL	177
17	LTPLCVTLNCTDLGNVTTTT---NSNGTLMEKGEVKNCSFKITTDIKDRTRKEYALFYKL	177
18	LTPLCVTLNCTDLGNVTNTT---NSNGGMMEKGEVKNCSFKITTDIKDRTRKEYALFYKL	177
19	LTPLCVTLNCTDLGNVTTTT---NSNGTLMEKGEVKNCSFKITTDIKDRTRKEYALFYKL	177
20	LTPLCVTLNCTDLGNVTTTT---NSNGTLMEKGEVKNCSFKITTDIKDRTRKEYALFYKL	177
21	LTPLCVTLNCTDLGNVTTTT---NSNGTLMEKGEVKNCSFKITTDIKDRTRKEYALFYKL	177
22	LTPLCVTLNCTDLGNVTNTT---NSNGGMMEKGEVKNCSFKITTDIKDRTRKEYALFYKL	177
23	LTPLCVTLNCTDLGNVTTTT---NSNGTLMEKGEVKNCSFKITTDIKDRTRKEYALFYKL	177
24	LTPLCVTLNCTDLGNVTTTT---NSNGTLMEKGEVKNCSFKITTDIKDRTRKEYALFYKL	177
25	LTPLCVTLNCTDLGNVTTTT---NSNGTLMEKGEVKNCSFKITTDIKDRTRKEYALFYKL	177
26	LTPLCVTLNCTDLGNVTTTT---NSNGTLMEKGEVKNCSFKIATDIKDRTRKEYALFYKL	177
27	LTPLCVTLNCTDLGNVTTTT---NSNGTLMEKGEVKNCSFKITTDIKDRTRKEYALFYKL	177
28	LTPLCVTLNCTDLGNVTTTT---NSNGTLMEKGEVKNCSFKITTDIKDRTRKEYALFYKL	177
29	LTPLCVTLNCTDLGNVTTTT---NSNGTLMEKGEVKNCSFKITTDIKDRTRKEYALFYKL	177
30	LTPLCVTLNCTDLGNVTTTT---NSNGTLMEKGEVKNCSFKITTDIKDRTRKEYALFYKL	177
31	LTPLCVTLNCTDLGNVTNTT---NSNGGMMEKGEVKNCSFKITTDIKDRTRKEYALFYKL	177
32	LTPLCVTLNCTDLGNVTTTT---NSNGTLMEKGEVKNCSFKITTDIKDRTRKEYALFYKL	177
33	LTPLCVTLNCTDLGNVTTTT---NSNGTLMEKGEVKNCSFKITTDIKDRTRKEYALFYKL	177
34	LTPLCVTLNCTDLGNVTTTT---NSNGTLMEKGEVKNCSFKITTDIKDRTRKEYALFYKL	177
35	LTPLCVTLNCTDLGNVTNTT---NSNGGMMEKGEVKNCSFKITTDIKDRTRKEYALFYKL	177
36	LTPLCVTLNCTDLGNVTTTT---NSNGTLMEKGEVKNCSFKITTDIKDRTRKEYALFYKL	177
37	LTPLCVTLNCTDLGNVTTTT---NSNGTLMEKGEVKNCSFKITTDIKDRTRKEYALFYKL	177
38	LTPLCVTLNCTDLGNVTTTT---NSNGTLMEKGEVKNCSFKITTDIKDRMRKEYALFYKL	177

	DVVPIND-------TRYRLVSCNTSVITQACPKVSFEPIPIHYCAPAGFAILKCNDKQFI	
1	DVVPIND-------TRYRLVSCNTSVITQACPKVSFEPIPIHYCAPAGFAILKCNDKQFI	230
2	DVVPIND-------TRYRLVSCNTSVITQACPKVSFEPIPIHYCAPAGFAILKCNDKQFI	230
3	DVVPIND-------TRYRLVSCNTSVITQACPKVSFEPIPIHYCAPAGFAILKCNDKQFM	230
4	DVVPIND-------TRYRLVSCNTSVITQACPKVSFEPIPIHYCAPAGFAILKCNDKQFM	230
5	DVVPIND-------TRYRLVSCNTSVITQACPKVSFEPIPIHYCAPAGFAILKCNDKQFI	230
6	DVVPIND-------TRYRLVSCNTSVITQACPKVSFEPIPIHYCAPAGFAILKCNDKQFI	230
7	DVVPIND-------TRYRLVSCNTSVITQACPKVSFEPIPIHYCAPAGFAILKCNDKQFM	230
8	DVVPIND-------TRYRLVSCNTSVITQACPKVSFEPIPIHYCAPAGFAILKCNDKQFI	230
9	DVVPIND-------TRYRLVSCNTSVITQACPKVSFEPIPIHYCAPAGFAILKCNDKQFM	230
10	DVVPIND-------TRYRLVSCNTSVITQACPKVSFEPIPIHYCAPAGFAILKCNDKQFM	230
11	DVVPIND-------TRYRLVSCNTSVITQACPKVSFEPIPIHYCAPAGFAILKCNDKQFM	230
12	DVVPIND-------TRYRLVSCNTSVITQACPKVSFEPIPIHYCAPAGFAILKCNDKQFI	230
13	DVVPIND-------TRYRLVSCNTSVITQACPKVSFEPIPIHYCAPAGFAILKCNDKQFI	230
14	DVVPIND-------TRYRLVSCNTSVITQACPKVSFEPIPIHYCAPAGFAILKCNDKQFI	230
15	DVVPIND-------TRYRLVSCNTSVIIQACPKVSFEPIPIHYCAPAGFAILKCNDKQFI	230
16	DVVPIND-------TRYRLVSCNTSVITQACPKVSFEPIPIHYCAPAGFAILKCNDKQFM	230
17	DVVPIND-------TRYRLVSCNTSVITQACPKVSFEPIPIHYCAPAGFAILKCNDKQFM	230
18	DVVPIND-------TRYRLVSCNTSVITQACPKVSFEPIPIHYCAPAGFAILKCNDKQFI	230
19	DVVPIND-------TRYRLVSCNTSVITQACPKVSFEPIPIHYCAPAGFAILKCNDKQFI	230
20	DVVPIND-------TRYRLVSCNTSVITQACPKVSFEPIPIHYCAPAGFAILKCNDKQFI	230
21	DVVPIND-------TRYRLVSCNTSVITQACPKVSFEPIPIHYCAPAGFAILKCNDKQFM	230
22	DVVPIND-------TRYRLVSCNTSVITQACPKVSFEPIPIHYCAPAGFAILKCNDKQFI	230
23	DVVPIND-------TRYRLVSCNTSVITQACPKVSFEPIPIHYCAPAGFAILKCNDKQFI	230
24	DVVPIND-------TRYRLVSCNTSVITQACPKVSFEPIPIHYCAPAGFAILKCNDKQFI	230
25	DVVPIND-------TRYRLVSCNTSVITQACPKVSFEPIPIHYCAPAGFAILKCNDKQFI	230
26	DVVPIND-------TRYRLVSCNTSVITQACPKVSFEPIPIHYCAPAGFAILKCNDKQFI	230
27	DVVPIND-------TRYRLVSCNTSVITQACPKVSFEPIPIHYCAPAGFAILKCNDKQFI	230
28	DVVPIND-------TRYRLVSCNTSVITQACPKVSFEPIPIHYCAPAGFAILKCNDKQFI	230
29	DVVPIND-------TRYRLVSCNTSVITQACPKVSFEPIPIHYCAPAGFAILKCNDKQFM	230
30	DVVPIND-------TRYRLVSCNTSVITQACPKVSFEPIPIHYCAPAGFAILKCNDKQFM	230
31	DVVPIND-------TRYRLVSCNTSVITQACPKVSFEPIPIHYCAPAGFAILKCNDKQFI	230
32	DVVPIND-------TRYRLVSCNTSVITQACPKVSFEPIPIHYCAPAGFAILKCNDKQFM	230
33	DVVPIND-------TRYRLVSCNTSVITQACPKVSFEPIPIHYCAPAGFAILKCNDKQFM	230
34	DVVPIND-------TRYRLVSCNTSVITQACPKVSFEPIPIHYCAPAGFAILKCNDKQFI	230
35	DVVPIND-------TRYRLVSCNTSVITQACPKVSFEPIPIHYCAPAGFAILKCNDKQFI	230
36	DVVPIND-------TRYRLVSCNTSVITQACPKVSFEPIPIHYCAPAGFAILKCNDKQFM	230
37	DVVPIND-------TRYRLVSCNTSVITQACPKVSFEPIPIHYCAPAGFAILKCNDKQFM	230
38	DVVPIND-------TRYRLVSCNTSVITQACPKVSFEPIPIHYCAPAGFAILKCNDKQFM	230

	GTGPCTNVSTVQCTHGIRPVVSTQLLLNGSLAEEEVVIRSVNFSDNAKTIIVQLNKSVEI	
1	GTGPCTNVSTVQCTHGIRPVVSTQLLLNGSLAEEEVVIRSVNFSDNAKTIIVQLNKSVEI	290
2	GTGPCTNVSTVQCTHGIRPVVSTQLLLNGSLAEEEVVIRSVNFSDNAKTIIVQLNKSVEI	290
3	GTGPCTNVSTVQCTHGIRPVVSTQLLLNGSLAEEEVVIRSVNFSDNAKTIIVQLNKSVEI	290
4	GTGPCTNVSTVQCTHGIRPVVSTQLLLNGSLAEEEVVIRSVNFSDNAKTIIVQLNKSVEI	290
5	GTGPCTNVSTVQCTHGIRPVVSTQLLLNGSLAEEEVVIRSVNFSDNAKTIIVQLNKSVEI	290
6	GTGPCTNVSTVQCTHGIRPVVSTQLLLNGSLAEEEVVIRSVNFSDNAKTIIVQLNKSVEI	290
7	GTGPCTNVSTVQCTHGIRPVVSTQLLLNGSLAEEEVVIRSVNFSDNAKTIIVQLNKSVEI	290
8	GTGPCTNVSTVQCTHGIRPVVSTQLLLNGSLAEEEVVIRSVNFSDNAKTIIVQLNKSVEI	290
9	GTGPCTNVSTVQCTHGIRPVVSTQLLLNGSLAEEEVVIRSVNFSDNAKTIIVQLNKSVEI	290
10	GTGPCTNVSTVQCTHGIRPVVSTQLLLNGSLAEEEVVIRSVNFSDNAKTIIVQLNKSVEI	290
11	GTGPCTNVSTVQCTHGIRPVVSTQLLLNGSLAEEEVVIRSVNFSDNAKTIIVQLNKSVEI	290
12	GTGPCTNVSTVQCTHGIRPVVSTQLLLNGSLAEEEVVIRSVNFSDNAKTIIVQLNKSVEI	290
13	GTGPCTNVSTVQCTHGIRPVVSTQLLLNGSLAEEEVVIRSVNFSDNAKTIIVQLNKSVEI	290
14	GTGPCTNVSTVQCTHGIRPVVSTQLLLNGSLAEEEVVIRSVNFSDNAKTIIVQLNKSVEI	290
15	GTGPCTNVSTVQCTHGIRPVVSTQLLLNGSLAEEEVVIRSVNFSDNAKTIIVQLNKSVEI	290
16	GTGPCTNVSTVQCTHGIRPVVSTQLLLNGSLAEEEVVIRSVNFSDNAKTIIVQLNKSVEI	290
17	GTGPCTNVSTVQCTHGIRPVVSTQLLLNGSLAEEEVVIRSVNFSDNAKTIIVQLNKSVEI	290
18	GTGPCTNVSTVQCTHGIRPVVSTQLLLNGSLAEEEVVIRSGNFSDNAKTIIVQLNKSVEI	290
19	GTGPCTNVSTVQCTHGIRPVVSTQLLLNGSLAEEEVVIRSVNFSDNAKTIIVQLNKSVEI	290
20	GTGPCTNVSTVQCTHGIRPVVSTQLLLNGSLAEEEVVIRSVNFSDNAKTIIVQLNKSVEI	290
21	GTGPCTNVSTVQCTHGIRPVVSTQLLLNGSLAEEEVVIRSVNFSDNAKTIIVQLNKSVEI	290
22	GTGPCTNVSTVQCTHGIRPVVSTQLLLNGSLAEEEVVIRSVNFSDNAKTIIVQLNKSVEI	290
23	GTGPCTNVSTVQCTHGIRPVVSTQLLLNGSLAEEEVVIRSVNFSDNAKTIIVQLNKSVEI	290
24	GTGPCTNVSTVQCTHGIRPVVSTQLLLNGSLAEEEVVIRSVNFSDNAKTIIVQLNKSVEI	290
25	GTGPCTNVSTVQCTHGIRPVVSTQLLLNGSLAEEEVVIRSVNFSDNAKTIIVQLNKSVEI	290
26	GTGPCTNVSTVQCTHGIRPVVSTQLLLNGSLAEEEVVIRSVNFSDNAKTIIVQLNKSVEI	290
27	GTGPCTNVSTVQCTHGIRPVVSTQLLLNGSLAEEEVVIRSVNFSDNAKTIIVQLNKSVEI	290
28	GTGPCTNVSTVQCTHGIRPVVSTQLLLNGSLAEEEVVIRSVNFSDNAKTIIVQLNKSVEI	290
29	GTGPCTNVSTVQCTHGIRPVVSTQLLLNGSLAEEEVVIRSVNFSDNAKTIIVQLNKSVEI	290
30	GTGPCTNVSTVQCTHGIRPVVSTQLLLNGSLAEEEVVIRSVNFSDNAKTIIVQLNKSVEI	290
31	GTGPCTNVSTVQCTHGIRPVVSTQLLLNGSLAEEEVVIRSVNFSDNAKTIIVQLNKSVEI	290
32	GTGPCTNVSTVQCTYGIRPVVSTQLLLNGSLAEEEVVIRSVNFSDNAKTIIVQLNKSVEI	290
33	GTGPCTNVSTVQCTHGIRPVVSTQLLLNGSLAEEEVVIRSVNFSDNAKTIIVQLNKSVEI	290
34	GTGPCTNVSTVQCTHGIRPVVSTQLLLNGSLAEEEVVIRSVNFSDNAKTIIVQLNKSVEI	290
35	GTGPCTNVSTVQCTHGIRPVVSTQLLLNGSLAEEEVVIRSVNFSDNAKTIIVQLNKSVEI	290
36	GTGPCTNVSTVQCTHGIRPVVSTQLLLNGSLAEEEVVIRSVNFSDNAKTIIVQLNKSVEI	290
37	GTGPCTNVSTVQCTHGIRPVVSTQLLLNGSLAEEEVVIRSVNFSDNAKTIIVQLNKSVEI	290
38	GTGPCTNVSTVQCTHGIRPVVSTQLLLNGSLAEEEVVIRSVNFSDNAKTIIVQLNKSVEI	290

	TCTRPNNNTRKSIPMGPGKAFYARGDIIGDIRKASCKINGTEWHSTLKLVVEKLREQY-N	
1	TCTRPNNNTRKSIPMGPGKAFYARGDITGDIRKAYCEINGTEWHSTLKLVVEKLREQY-N	349
2	TCTRPNNNTRKSIPMGPGKAFYARGDIIGDIRKASCKINGTEWHSTLKLVVEKLREQY-N	349
3	TCTRPNNNTRKSIPMGPGKAFYARGDIIGDIRKASCKINGTEWHSTLKLVVEKLREQY-N	349
4	TCTRPNNNTRKSIPMGPGKAFYARGDIIGDIRKASCKINGTEWHSTLKLVVEKLREQY-N	349
5	NCTRPNNNTRKSIPMGPGKAFYARGDIIGDIRKASCELNGTEWHSTLKLVVEKLREQY-N	349
6	NCTRPHNNTRKSIPMGPGKAFYARGDIIGDIRKAYCKINGTEWHSTLKLVVEKLREQY-N	349
7	TCTRPNNNTRKSIPMGPGKAFYARGDIIGDIRKASCKINGTEWHSTLKLVVEKLREQY-N	349
8	TCTRPNNNTRKSIPMGPGKAFYARGDIIGDIRKASCKINGTEWHSTLKLVVEKLREQY-N	349
9	TCTRPNNNTRKSIPMGPGKAFYARGDIIGDIRKASCKINGTEWHSTLKLVVEKLREQY-N	349
10	TCTRPNNNTRKSIPMGPGKAFYARGDIIGDIRKASCKINGTEWHSTLKLVVEKLREQY-N	349
11	TCTRPNNNTRKSIPMGPGKAFYARGDIIGDIRKASCKINGTEWHSTLKLVVEKLREQY-N	349
12	TCTRPNNNTRKSIPMGPGKAFYARGDIIGDIRKASCKINGTEWHSTLKLVVEKLREQY-N	349
13	NCTRPHNNTRKSIPMGPGKAFYARGDITGDIRKAYCEINGTEWHSTLKLVVEKLRGQY-N	349
14	TCTRPNNNTRKSIPMGPGKAFYARGDIIGDIRKASCKINGTEWHSTLKLVVEKLREQY-N	349
15	TCTRPNNNTRKSIPMGPGKAFYARGDIIGDIRKASCEINGTEWHSTLKLVVEKLREQY-N	349
16	TCTRPNNNTRKSIPMGPGKAFYARGDIIGDIRKASCKINGTEWHSTLKLVVEKLREQY-N	349
17	TCTRPNNNTRKSIPMGPGKAFYARGDIIGDIRKASCKINGTEWHSTLKLVVEKLREQY-N	349
18	NCTRPHNNTRKSIPMGPGKAFYARGDIIGDIRKAYCKINGTEWHSTLKLVVGKLREQY-N	349
19	TCTRPNNNTRKSIPMGPGKAFYARGDIIGDIRKASCKINGTEWHSTLKLVVEKLREQY-N	349
20	NCTRPNNNTRKSIPMGPGKAFYARGDIIGDIRKASCELNGTEWHSTLKLVVEKLREQY-N	349
21	TCTRPNNNTRKSIPMGPGKAFYARGDIIGDIRKASCKINGTEWHSTLKLVVEKLREQY-N	349
22	NCTRPHNNTRKSIPMGPGKAFYARGDIIGDIRKAYCKINGTEWHSTLKLVVEKLREQY-N	349
23	TCTRPNNNTRKSIPMGPGKAFYARGDIIGDIRKASCKINGTEWHSTLKLVVEKLREQY-N	349
24	TCTRPNNNTRKSIPMGPGKAFYARGDIIGDIRKASCKINGTEWHSTLKLVVEKLREQY-N	349
25	TCTRPNNNTRKSIPMGPGKAFYARGDIIGDIRKASCKINGTEWHSTLKLVVEKLREQY-N	349
26	TCTRPNNNTRKSIPMGPGKAFYARGDIIGDIRKASCKINGTEWHSTLKLVVEKLREQY-N	349
27	TCTRPNNNTRKSIPMGPGKAFYARGDIIGDIRKASCKINGTEWHSTLKLVVEKLREQY-N	349
28	TCTRPNNNTRKSIPMGPGKAFYARGDIIGDIRKASCKINGTEWHSTLKLVVEKLREQY-N	349
29	TCTRPNNNTRKSIPMGPGKAFYARGDIIGDIRKASCKINGTEWHSTLKLVVEKLREQY-N	349
30	TCTRPNNNTRKSIPMGPGKAFYARGDIIGDIRKASCKINGTEWHSTLKLVVEKLREQY-N	349
31	NCTRPHNNTRKSIPMGPGKAFYARGDIIGDIRKAYCKINGTEWHSTLKLVVEKLREQY-N	349
32	TCTRPNNNTRKSIPMGPGKAFYARGDIIGDIRKASCKINGTEWHSTLKLVVEKLREQY-N	349
33	TCTRPNNNTRKSIPMGPGKAFYARGDIIGDIRKASCKINGTEWHSTLKLVVEKLREQY-N	349
34	TCTRPNNNTRKSIPMGPGKAFYARGDIIGDIRKASCKINGTEWHSTLKLVVEKLREQY-N	349
35	NCTRPHNNTRKSIPMGPGKAFYARGDIIGDIRKAYCKINGTEWHSTLKLVVEKLREQY-N	349
36	TCTRPNNNTRKSIPMGPGKAFYARGDIIGDIRKASCKINGTEWHSTLKLVVEKLREQY-N	349
37	TCTRPNNNTRKSIPMGPGKAFYARGDIIGDIRKASCKINGTEWHSTLKLVVEKLREQY-N	349
38	TCTRPNNNTRKSIPMGPGKAFYARGDIIGDIRKASCKINGTEWHSTLKLVVEKLREQY-N	349

```
    KTIVFNRSSGGDPEIVMYSFNCGGEFFYCNSTKLFNSTWPWND-TKGSHDTNDTLMLPCK
1   KTIVFNRSSGGDPEIVMYSFNCGGEFFYCNSTKLFNSTWPWND-TKGSHDTNGTLILPCK  408
2   KTIVFNRSSGGDPEIVMYSFNCGGEFFYCNSTKLFNSTWPWND-TKGSHDTNDTLMLPCK  408
3   KTIVFNRSSGGDPEIVMYSFNCGGEFFYCNSTKLFNSTWPWND-TKGSHDTNDTLMLPCK  408
4   KTIVFNRSSGGDPEIVMYSFNCGGEFFYCNSTKLFNSTWPWND-TKGSHDTNDTLMLPCK  408
5   KTIVFNRSSGGDPEIVMYSFNCGGEFFYCNSTKLFNSTWPWND-TKGSHDTNGTLILPCR  408
6   KTIVFNRSSGGDPEIVMYSFNCGGEFFYCNSTKLFNSTWPWND-TKGSHDTNDTLMLPCK  408
7   KTIVFNRSSGGDPEIVMYSFNCGGEFFYCNSTKLFNSTWPWND-TKGSHDTNDTLMLPCK  408
8   KTIVFNRSSGGDPEIVMYSFNCGGEFFYCNSTKLFNSTWPWND-TKGSHDTNDTLMLPCK  408
9   KTIVFNRSSGGDPEIVMYSFNCGGEFFYCNSTKLFNSTWPWND-TKGSHDTNDTLMLPCK  408
10  KTIVFNRSSGGDPEIVMYSFNCGGEFFYCNSTKLFNSTWPWND-TKGSHDTNDTLMLPCK  408
11  KTIVFNRSSGGDPEIVMYSFNCGGEFFYCNSTKLFNSTWPWND-TKGSHDTNDTLMLPCK  408
12  KTIVFNRSSGGDPEIVMYSFNCGGEFFYCNSTKLFNSTWPWND-TKGSHDTNDTLMLPCK  408
13  KTIVFNRSSGGDPEIVMYSFNCGGEFFYCNSTKLFNSTWPWND-TKGSHDTNGTLILPCK  408
14  KTIVFNRSSGGDPEIVMYSFNCGGEFFYCNSTKLFNSTWPWND-TKGSHDTNDTLMLPCK  408
15  KTIVFNRSSGGDPEIVMYSFNCGGEFFYCNSTKLFNSTWPWND-TKGSHDTNGRLILPCR  408
16  KTIVFNRSSGGDPEIVMYSFNCGGEFFYCNSTKLFNSTWPWND-TKGSHDTNDTLMLPCK  408
17  KTIVFNRSSGGDPEIVMYSFNCGGEFFYCNSTKLFNSTWPWND-TKGSHDTNDTLMLPCK  408
18  KTIVFNRSSGGDPEIVMYSFNCGGEFFYCNSTKLFNSTWPWND-TKGSHDTNDTLMLPCK  408
19  KTIVFNRSSGGDPEIVMYSFNCGGEFFYCNSTKLFNSTWPWND-TKGSHDTNDTLMLPCK  408
20  KTIVFNRSSGGDPEIVMYSFNCGGEFFYCNSTKLFNSTWPWND-TKGSHDTNGTLILPCR  408
21  KTIVFNRSSGGDPEIVMYSFNCGGEFFYCNSTKLFNSTWPWND-TKGSHDTNDTLMLPCK  408
22  KTIVFNRSSGGDPEIVMYSFNCGGEFFYCNSTKLFNSTWPWND-TKGSHDTNDTLMLPCK  408
23  KTIVFNRSSGGDPEIVMYSFNCGGEFFYCNSTKLFNSTWPWND-TKGSHDTNDTLMLPCK  408
24  KTIVFNRSSGGDPEIVMYSFNCGGEFFYCNSTKLFNSTWPWND-TKGSHDTNDTLMLPCK  408
25  KTIVFNRSSGGDPEIVMYSFNCGGEFFYCNSTKLFNSTWPWND-TKGSHDTNDTLMLPCK  408
26  KTIVFNRSSGGDPEIVMYSFNCGGEFFYCN-TKLFNSTWPWND-TKGSHDTNDTLMLPCK  407
27  KTIVFNRSSGGDPEIVMYSFNCGGEFFYCNSTKLFNSTWPWND-TKGSHDTNDTLMLPCK  408
28  KTIVFNRSSGGDPEIVMYSFNCGGEFFYCNSTKLFNSTWPWND-TKGSHDTNDTLMLPCK  408
29  KTIVFNRSSGGDPEIVMYSFNCGGEFFYCNSTKLFNSTWPWND-TKGSHDTNDTLMLPCK  408
30  KTIVFNRSSGGDPEIVMYSFNCGGEFFYCNSTKLFNSTWPWND-TKGSHDTNDTLMLPCK  408
31  KTIVFNRSSGGDPEIVMYSFNCGGEFFYCNSTKLFNSTWPWND-TKGSHDTNDTLMLPCK  408
32  KTIVFNRSSGGDPEIVMYSFNCGGEFFYCNSTKLFNSTWPWND-TKGSHDTNDTLMLPCK  408
33  KTIVFNRSSGGDPEIVMYSFNCGGEFFYCNSTKLFNSTWPWND-TKGSHDTNDTLMLPCK  408
34  KTIVFNRSSGGDPEIVMYSFNCGGEFFYCNSTKLFNSTWPWND-TKGSHDTNDTLMLPCK  408
35  KTIVFNRSSGGDPEIVMYSFNCGGEFFYCNSTKLFNSTWPWND-TKGSHDTNDTLMLPCK  408
36  KTIVFNRSSGGDPEIVMYSFNCGGEFFYCNSTKLFNSTWPWND-TKGSHDTNDTLMLPCK  408
37  KTIVFNRSSGGDPEIVMYSFNCGGEFFYCNSTKLFNSTWPWND-TKGSHDTNDTLMLPCK  408
38  KTIVFNRSSGGDPEIVMYSFNCGGEFFYCNSTKLFNSTWPWND-TKGSHDTNDTLMLPCK  408
```

	IKQIINMWQGVGKAMYAPPIEGIIRCSSNITGLLLTRDGG-YESNETDEIFRPGGGDMRD	
1	IKQIINMWQGVGKAMYAPPIEGKIRCSSNITGLLLTRDGG-YESNETDEIFRPGGGDMRD	467
2	IKQIINMWQGVGKAMYAPPIEGIIRCSSNITGLLLTRDGG-YESNETDEIFRPGGGDMRD	467
3	IKQIINMWQGVGKAMYAPPIEGIIRCSSNITGLLLTRDGG-YESNETDEIFRPGGGDMRD	467
4	IKQIINMWQGVGKAMYAPPIEGIIRCSSNITGLLLTRDGG-YESNETDEIFRPGGGDMRD	467
5	IKQIINMWQGVGKAMYAPPIEGEIRCSSNITGLLLTRDGG-YESNETDEIFRPGGGDMRD	467
6	IKQIINMWQGVGKAMYAPPIEGQIRCSSNITGLLLTRDGG-YESNETDEIFRPGGGDMRD	467
7	IKQIINMWQGVGKAMYAPPIEGIIRCSSNITGLLLTRDGG-YESNETDEIFRPGGGDMRD	467
8	IKQIINMWQGVGKAMYAPPIEGIIRCSSNITGLLLTRDGG-YESNETDEIFRPGGGDMRD	467
9	IKQIINMWQGVGKAMYAPPIEGIIRCSSNITGLLLTRDGG-YESNETDEIFRPGGGDMRD	467
10	IKQIINMWQGVGKAMYAPPIEGIIRCSSNITGLLLTRDGG-YESNETDEIFRPGGGDMRD	467
11	IKQIINMWQGVGKAMYAPPIEGIIRCSSNITGLLLTRDGG-YESNETDEIFRPGGGDMRD	467
12	IKQIINMWQGVGKAMYAPPIEGIIRCSSNITGLLLTRDGG-YESNETDEIFRPGGGDMRD	467
13	IKQIINMWQGVGKAMYAPPIEGKIRCSSNITGLLLTRDGG-YESNETDEIFRPGGGDMRD	467
14	IKQIINMWQGVGKAMYAPPIEGIIRCSSNITGLLLTRDGG-YESNETDEIFRPGGGDMRD	467
15	IKQIINMWQGVGKAMYAPPIEGEIRCSSNITGLLLTRDGG-YESNETDEIFRPGGGDMRD	467
16	IKQIINMWQGVGKAMYAPPIEGIIRCSSNITGLLLTRDGG-YESNETDEIFRPGGGDMRD	467
17	IKQIINMWQGVGKAMYAPPIEGIIRCSSNITGLLLTRDGG-YESNETDEIFRPGGGDMRD	467
18	IKQIINMWQGVGKAMYAPPIEGQIRCSSNITGLLLTRDGG-YESNETDEIFRPGGGDMRD	467
19	IKQIINMWQGVGKAMYAPPIEGIIRCSSNITGLLLTRDGG-YESNETDEIFRPGGGDMRD	467
20	IKQIINMWQGVGKAMYAPPIEGEIRCSSNITGLLLTRDGG-YESNETDEIFRPGGGDMRD	467
21	IKQIINMWQGVGKAMYAPPIEGIIRCSSNITGLLLTRDGG-YESNETDEIFRPGGGDMRD	467
22	IKQIINMWQGVGKAMYAPPIEGQIRCSSNITGLLLTRDGG-YESNETDEIFRPGGGDMRD	467
23	IKQIINMWQGVGKAMYAPPIEGIIRCSSNITGLLLTRDGG-YESNETDEIFRPGGGDMRD	467
24	IKQIINMWQGVGKAMYAPPIEGIIRCSSNITGLLLTRDGG-YESNETDEIFRPGGGDMRD	467
25	IKQIINMWQGVGKAMYAPPIEGIIRCSSNITGLLLTRDGG-YESNETDEIFRPGGGDMRD	467
26	IKQIINMWQGVGKAMYAPPIEGIIRCSSNITGLLLTRDGG-YESNETDEIFRPGGGDMRD	466
27	IKQIINMWQGVGKAMYAPPIEGIIRCSSNITGLLLTRDGG-YESNETDEIFRPGGGDMRD	467
28	IKQIINMWQGVGKAMYAPPIEGIIRCSSNITGLLLTRDGG-YESNETDEIFRPGGGDMRD	467
29	IKQIINMWQGVGKAMYAPPIEGIIRCSSNITGLLLTRDGG-YESNETDEIFRPGGGDMRD	467
30	IKQIINMWQGVGKAMYAPPIEGIIRCSSNITGLLLTRDGG-YESNETDEIFRPGGGDMRD	467
31	IKQIINMWQGVGKAMYAPPIEGQIRCSSNITGLLLTRDGG-YESNETDEIFRPGGGDMRD	467
32	IKQIINMWQGVGKAMYAPPIEGIIRCSSNITGLLLTRDGG-YESNETEEIFRPGGGDMRD	467
33	IKQIINMWQGVGKAMYAPPIEGIIRCSSNITGLLLTRDGG-YESNETDEIFRPGGGDMRD	467
34	IKQIINMWQGVGKAMYAPPIEGIIRCSSNITGLLLTRDGG-YESNETDEIFRPGGGDMRD	467
35	IKQIINMWQGVGKAMYAPPIEGQIRCSSNITGLLLTRDGG-YESNETDEIFRPGGGDMRD	467
36	IKQIINMWQGVGKAMYAPPIEGIIRCSSNITGLLLTRDGG-YESNETDEIFRPGGGDMRD	467
37	IKQIINMWQGVGKAMYAPPIEGIIRCSSNITGLLLTRDGG-YESNETDEIFRPGGGDMRD	467
38	IKQIINMWQGVGKAMYAPPIEGIIRCSSNITGLLLTRDGG-YESNETDEIFRPGGGDMRD	467

	Sequence	
	**NWRSELYKYKVVKIEPLGVAPTKAKRRVVQREKRAFG-LGAVFLGFLGAAGSTMGAASIT**	
1	NWRSELYKYKVVKIEPLGVAPTKAKRRVVQREKRAFG-LGAVFLGFLGAAGSTMGAASIT	526
2	NWRSELYKYKVVKIEPLGVAPTKAKRRVVQREKRAFG-LGAVFLGFLGAAGSTMGAASIT	526
3	NWRSELYKYKVVKIEPLGVAPTRAKRRVVQREKRAFG-LGAVFLGFLGAAGSTMGAASIT	526
4	NWRSELYKYKVVKIEPLGVAPTKAKRRVVQREKRAFG-LGAVFLGFLGAAGSTMGAASIT	526
5	NWRSELYKYKVVKIEPLGVAPTKAKRRVVQREKRAFG-LGAVFLGFLGAAGSTMGAASIT	526
6	NWRSELYKYKVVKIEPLGVAPTKAKRRVVQREKRAFG-LGAVFLGFLGAAGSTMGAASIT	526
7	NWRSELYKYKVVKIEPLGVAPTRAKRRVVQREKRAFG-LGAVFLGFLGAAGSTMGAASIT	526
8	NWRSELYKYKVVKIEPLGVAPTKAKRRVVQREKRAFG-LGAVFLGFLGAAGSTMGAASIT	526
9	NWRSELYKYKVVKIEPLGVAPTRAKRRVVQREKRAFG-LGAVFLGFLGAAGSTMGAASIT	526
10	NWRSELYKYKVVKIEPLGVAPTRAKRRVVQREKRAFG-LGAVFLGFLGAAGSTMGAASIT	526
11	NWRSELYKYKVVKIEPLGVAPTRAKRRVVQREKRAFG-LGAVFLGFLGAAGSTMGAASIT	526
12	NWRSELYKYKVVKIEPLGVAPTKAKRRVVQREKRAFG-LGAVFLGFLGAAGSTMGAASIT	526
13	NWRSELYKYKVVKIEPLGVAPTKAKRRVVQREKRAFG-LGAVFLGFLGAAGSTMGAASIT	526
14	NWRSELYKYKVVKIEPLGVAPTKAKRRVVQREKRAFG-LGAVFLGFLGAAGSTMGAASIT	526
15	NWRSELYKYKVVKIEPLGVAPTKAKRRVVQREKRAFG-LGAVFLGFLGAAGSTMGAASIT	526
16	NWRSELYKYKVVKIEPLGVAPTRAKRRVVQREKRAFG-LGAVFLGFLGAAGSTMGAASIT	526
17	NWRSELYKYKVVKIEPLGVAPTRAKRRVVQREKRAFG-LGAVFLGFLGAAGSTMGAASIT	526
18	NWRSELYKYKVVKIEPLGVAPTKAKRRVVQREKRAFG-LGAVFLGFLGAAGSTMGAASIT	526
19	NWRSELYKYKVVKIEPLGVAPTKAKRRVVQREKRAFG-LGAVFLGFLGAAGSTMGAASIT	526
20	NWRSELYKYKVVKIEPLGVAPTKAKRRVVQREKRAFG-LGAVFLGFLGAAGSTMGAASIT	526
21	NWRSELYKYKVVKIEPLGVAPTKAKRRVVQREKRAFG-LGAVFLGFLGAAGSTMGAASIT	526
22	NWRSELYKYKVVKIEPLGVAPTKAKRRVVQREKRAFG-LGAVFLGFLGAAGSTMGAASIT	526
23	NWRSELYKYKVVKIEPLGVAPTKAKRRVVQREKRAFG-LGAVFLGFLGAAGSTMGAASIT	526
24	NWRSELYKYKVVKIEPLGVAPTKAKRRVVQREKRAFG-LGAVFLGFLGAAGSTMGAASIT	526
25	NWRSELYKYKVVKIEPLGVAPTKAKRRVVQREKRAFG-LGAVFLGFLGAAGSTMGAASIT	526
26	NWRSELYKYKVVKIEPLGVAPTKAKRRVVQREKRAFG-LGAVFLGFLRAAGSTMGAASIT	525
27	NWRSELYKYKVVKIEPLGVAPTKAKRRVVQREKRAFG-LGAVFLGFLGAAGSTMGAASIT	526
28	NWRSELYKYKVVKIEPLGVAPTKAKRRVVQREKRAFG-LGAVFLGFLGAAGSTMGAASIT	526
29	NWRSELYKYKVVKIEPLGVAPTRAKRRVVQREKRAFG-LGAVFLGFLGAAGSTMGAASIT	526
30	NWRSELYKYKVVKIEPLGVAPTRAKRRVVQREKRAFG-LGAVFLGFLGAAGSTMGAASIT	526
31	NWRSELYKYKVVKIEPLGVAPTKAKRRVVQREKRAFG-LGAVFLGFLGAAGSTMGAASIT	526
32	NWRSELYKYKVVKIEPLGVAPTRAKRRVVQREKRAFG-LGAVFLGFLGAAGSTMGAASIT	526
33	NWRSELYKYKVVKIEPLGVAPTRAKRRVVQREKRAFG-LGAVFLGFLGAAGSTMGAASIT	526
34	NWRSELYKYKVVKIEPLGVAPTKAKRRVVQREKRAFG-LGAVFLGFLGAAGSTMGAASIT	526
35	NWRSELYKYKVVKIEPLGVAPTKAKRRVVQREKRAFG-LGAVFLGFLGAAGSTMGAASIT	526
36	NWRSELYKYKVVKIEPLGVAPTRAKRRVVQREKRAFG-LGAVFLGFLGAAGSTMGAASIT	526
37	NWRSELYKYKVVKIEPLGVAPTRAKRRVVQREKRAFG-LGAVFLGFLGAAGSTMGAASIT	526
38	NWRSELYKYKVVKIEPLGVAPTRAKRRVVQREKRAFG-LGAVFLGFLGAAGSTMGAASIT	526

	**LTVQARQLLSGIVQQQNNLLRAIEAQQHLLQLTVWGIKQLQARVLAVERYLKDQQLLGIW**	
1	LTVQARQLLSGIVQQQNNLLRAIEAQQHLLQLTVWGIKQLQARVLAVERYLKDQQLLGIW	586
2	LTVQARQLLSGIVQQQNNLLRAIEAQQHLLQLTVWGIKQLQARVLAVERYLKDQQLLGIW	586
3	LTVQARQLLSGIVQQQNNLLRAIEAQQHLLQLTVWGIKQLQARVLAVERYLKDQQLLGIW	586
4	LTVQARQLLSGIVQQQNNLLRAIEAQQHLLQLTVWGIKQLQARVLAVERYLKDQQLLGIW	586
5	LTVQARQLLSGIVQQQNNLLRAIEAQQHLLQLTVWGIKQLQARVLAVERYLKDQQLLGIW	586
6	LTVQARQLLSGIVQQQNNLLRAIEAQQHLLQLTVWGIKQLQARVLAVERYLKDQQLLGIW	586
7	LTVQARQLLSGIVQQQNNLLRAIEAQQHLLQLTVWGIKQLQARVLAVERYLKDQQLLGIW	586
8	LTVQARQLLSGIVQQQNNLLRAIEAQQHLLQLTVWGIKQLQARVLAVERYLKDQQLLGIW	586
9	LTVQARQLLSGIVQQQNNLLRAIEAQQHLLQLTVWGIKQLQARVLAVERYLKDQQLLGIW	586
10	LTVQARQLLSGIVQQQNNLLRAIEAQQHLLQLTVWGIKQLQARVLAVERYLKDQQLLGIW	586
11	LTVQARQLLSGIVQQQNNLLRAIEAQQHLLQLTVWGIKQLQARVLAVERYLKDQQLLGIW	586
12	LTVQARQLLSGIVQQQNNLLRAIEAQQHLLQLTVWGIKQLQARVLAVERYLKDQQLLGIW	586
13	LTVQARQLLSGIVQQQNNLLRAIEAQQHLLQLTVWGIKQLQARVLAVERYLKDQQLLGIW	586
14	LTVQARQLLSGIVQQQNNLLRAIEAQQHLLQLTVWGIKQLQARVLAVERYLKDQQLLGIW	586
15	LTVQARQLLSGIVQQQNNLLRAIEAQQHLLQLTVWGIKQLQARVLAVERYLKDQQLLGIW	586
16	LTVQARQLLSGIVQQQNNLLRAIEAQQHLLQLTVWGIKQLQARVLAVERYLKDQQLLGIW	586
17	LTVQARQLLSGIVQQQNNLLRAIEAQQHLLQLTVWGIKQLQARVLAVERYLKDQQLLGIW	586
18	LTVQARQLLSGIVQQQNNLLRAIEAQQHLLQLTVWGIKQLQARVLAVERYLKDQQLLGIW	586
19	LTVQARQLLSGIVQQQNNLLRAIEAQQHLLQLTVWGIKQLQARVLAVERYLKDQQLLGIW	586
20	LTVQARQLLSGIVQQQNNLLRAIEAQQHLLQLTVWGIKQLQARVLAVERYLKDQQLLGIW	586
21	LTVQARQLLSGIVQQQNNLLRAIEAQQHLLQLTVWGIKQLQARVLAVERYLKDQQLLGIW	586
22	LTVQARQLLSGIVQQQNNLLRAIEAQQHLLQLTVWGIKQLQARVLAVERYLKDQQLLGIW	586
23	LTVQARQLLSGIVQQQNNLLRAIEAQQHLLQLTVWGIKQLQARVLAVERYLKDQQLLGIW	586
24	LTVQARQLLSGIVQQQNNLLRAIEAQQHLLQLTVWGIKQLQARVLAVERYLKDQQLLGIW	586
25	LTVQARQLLSGIVQQQNNLLRAIEAQQHLLQLTVWGIKQLQARVLAVERYLKDQQLLGIW	586
26	LTVQARQLLSGIVQQQNNLLRAIEAQQHLLQLTVWGIKQLQARVLAVERYLKDQQLLGIW	585
27	LTVQARQLLSGIVQQQNNLLRAIEAQQHLLQLTVWGIKQLQARVLAVERYLKDQQLLGIW	586
28	LTVQARQLLSGIVQQQNNLLRAIEAQQHLLQLTVWGIKQLQARVLAVERYLKDQQLLGIW	586
29	LTVQARQLLSGIVQQQNNLLRAIEAQQHLLQLTVWGIKQLQARVLAVERYLKDQQLLGIW	586
30	LTVQARQLLSGIVQQQNNLLRAIEAQQHLLQLTVWGIKQLQARVLAVERYLKDQQLLGIW	586
31	LTVQARQLLSGIVQQQNNLLRAIEAQQHLLQLTVWGIKQLQARVLAVERYLKDQQLLGIW	586
32	LTVQARQLLSGIVQQQNNLLRAIEAQQHLLQLTVWGIKQLQARVLAVERYLKDQQLLGIW	586
33	LTVQARQLLSGIVQQQNNLLRAIEAQQHLLQLTVWGIKQLQARVLAVERYLKDQQLLGIW	586
34	LTVQARQLLSGIVQQQNNLLRAIEAQQHLLQLTVWGIKQLQARVLAVERYLKDQQLLGIW	586
35	LTVQARQLLSGIVQQQNNLLRAIEAQQHLLQLTVWGIKQLQARVLAVERYLKDQQLLGIW	586
36	LTVQARQLLSGIVQQQNNLLRAIEAQQHLLQLTVWGIKQLQARVLAVERYLKDQQLLGIW	586
37	LTVQARQLLSGIVQQQNNLLRAIEAQQHLLQLTVWGIKQLQARVLAVERYLKDQQLLGIW	586
38	LTVQARQLLSGIVQQQNNLLRAIEAQQHLLQLTVWGIKQLQARVLAVERYLKDQQLLGIW	586

GCSGKLICTTTVPWNTSWSNKSLEQIWDNMTWMEWEREIDNYTGYIYQLIEESQNQQEKN

1	GCSGKLICTTTVPWNTSWSNKSLEQIWDNMTWMEWEREIDNYTGYIYQLIEESQNQQEKN	646
2	GCSGKLICTTTVPWNTSWSNKSLEQIWDNMTWMEWEREIDNYTGYIYQLIEESQNQQEKN	646
3	GCSGKLICTTTVPWNTSWSNKSLEQIWDNMTWMEWEREIDNYTGYIYQLIEESQNQQEKN	646
4	GCSGKLICTTTVPWNTSWSNKSLEQIWDNMTWMEWEREIDNYTGYIYQLIEESQNQQEKN	646
5	GCSGKLICPTTVPWNTSWSNKSLEQIWDNMTWMEWEREIDNYTGYIYQLIEESQNQQEKN	646
6	GCSGKLICTTTVPWNTSWSNKSLEQIWDNMTWMEWEREIDNYTDYIYQLIEESQNQQEKN	646
7	GCSGKLICTTTVPWNTSWSNKSLEQIWDNMTWMEWEREIDNYTGYIYQLIEESQNQQEKN	646
8	GCSGKLICTTTVPWNTSWSNKSLEQIWDNMTWMEWEREIDNYTGYIYQLIEESQNQQEKN	646
9	GCSGKLICTTTVPWNTSWSNKSLEQIWDNMTWMEWEREIDNYTGYIYQLIEESQNQQEKN	646
10	GCSGKLICTTTVPWNTSWSNKSLEQIWDNMTWMEWEREIDNYTGYIYQLIEESQNQQEKN	646
11	GCSGKLICTTTVPWNTSWSNKSLEQIWDNMTWMEWEREIDNYTGYIYQLIEESQNQQEKN	646
12	GCSGKLICTTTVPWNTSWSNKSLEQIWDNMTWMEWEREIDNYTGYIYQLIEESQNQQEKN	646
13	GCSGKLICTTTVPWNTSWSNKSLEQIWDNMTWMEWEREIDNYTGYIYQLIEESQNQQEKN	646
14	GCSGKLICTTTVPWNTSWSNKSLEQIWDNMTWMEWEREIDNYTGYIYQLIEESQNQQEKN	646
15	GCSGKLICTTTVPWNTSWSNKSLEQIWDNMTWMEWEREIDNYTGYIYQLIEESQNQQEKN	646
16	GCSGKLICTTTVPWNTSWSNKSLEQIWDNMTWMEWEREIDNYTGYIYQLIEESQNQQEKN	646
17	GCSGKLICTTTVPWNTSWSNKSLEQIWDNMTWMEWEREIDNYTGYIYQLIEESQNQQEKN	646
18	GCSGKLICTTTVPWNTSWSNKSLEQIWDNMTWMEWEREIDNYTGYIYQLIEESQNQQEKN	646
19	GCSGKLICTTTVPWNTSWSNKSLEQIWDNMTWMEWEREIDNYTGYIYQLIEESQNQQEKN	646
20	GCSGKLICPTTVPWNTSWSNKSLEQIWDNMTWMEWEREIDNYTGYIYQLIEESQNQQEKN	646
21	GCSGKLICTTAVPWNTSWSNKSLEQIWDNMTWMEWEREIDNYTGYIYQLIEESQNQQEKN	646
22	GCSGKLICTTTVPWNTSWSNKSLEQIWDNMTWMEWEREIDNYTDYIYQLIEESQNQQEKN	646
23	GCSGKLICTTTVPWNTSWSNKSLEQIWDNMTWMEWEREIDNYTGYIYQLIEESQNQQEKN	646
24	GCSGKLICTTTVPWNTSWSNKSLEQIWDNMTWMEWEREIDNYTGYIYQLIEESQNQQEKN	646
25	GCSGKLICTTTVPWNTSWSNKSLEQIWDNMTWMEWEREIDNYTGYIYQLIEESQNQQEKN	646
26	GCSGKLICTTTVPWNTSWSNKSLEQIWDNMTWMEWEREIDNYTGYIYQLIEESQNQQEKN	645
27	GCSGKLICTTTVPWNTSWSNKSLEQIWDNMTWMEWEREIDNYTGYIYQLIEESQNQQEKN	646
28	GCSGKLICTTTVPWNTSWSNKSLEQIWDNMTWMEWEREIDNYTGYIYQLIEESQNQQEKN	646
29	GCSGKLICTTTVPWNTSWSNKSLEQIWDNMTWMEWEREIDNYTGYIYQLIEESQNQQEKN	646
30	GCSGKLICTTTVPWNTSWSNKSLEQIWDNMTWMEWEREIDNYTGYIYQLIEESQNQQEKN	646
31	GCSGKLICTTTVPWNTSWSNKSLEQIWDNMTWMEWEREIDNYTGYIYQLIEESQNQQEKN	646
32	GCSGKLICTTTVPWNTSWSNKSLEQIWDNMTWMEWEREIDNYTGYIYQLIEESQNQQEKN	646
33	GCSGKLICTTTVPWNTSWSNKSLEQIWDNMTWMEWEREIDNYTGYIYQLIEESQNQQEKN	646
34	GCSGKLICTTTVPWNTSWSNKSLEQIWDNMTWMEWEREIDNYTGYIYQLIEESQNQQEKN	646
35	GCSGKLICTTTVPWNTSWSNKSLEQIWDNMTWMEWEREIDNYTGYIYQLIEESQNQQEKN	646
36	GCSGKLICTTTVPWNTSWSNKSLEQIWDNMTWMEWEREIDNYTGYIYQLIEESQNQQEKN	646
37	GCSGKLICTTTVPWNTSWSNKSLEQIWDNMTWMEWEREIDNYTGYIYQLIEESQNQQEKN	646
38	GCSGKLICTTTVPWNTSWSNKSLEQIWDNMTWMEWEREIDNYTGYIYQLIEESQNQQEKN	646

	**EQELLALDKWASLWNWFDITNWLWYIKIFIMIVGGLIGLRIVFTVLSIVNRVRQGYSPLS**	
1	EQELLALDKWASLWNWFDITNWLWYIKIFIMIVGGLIGLRIVFTVLSIVNRVRQGYSPLS	706
2	EQELLALDKWASLWNWFDITNWLWYIKIFIMIVGGLIGLRIVFTVLSIVNRVRQGYSPLS	706
3	EQELLALDKWASLWNWFDITNWLWYIKIFIMIVGGLIGLRIVFTVLSIVNRVRQGYSPLS	706
4	EQELLALDKWASLWNWFDITNWLWYIKIFIMIVGGLIGLRIVFTVLSIVNRVRQGYSPLS	706
5	EQELLALDKWASLWNWFDITNWLWYIKIFIMIVGGLIGLRIVFTVLSIVNRVRQGYSPLS	706
6	EQELLALDKWASLWNWFDITNWLWYIKIFIMIVGGLIGLRIVFTVLSIVNRVRQGYSPLS	706
7	EQELLALDKWASLWNWFDITNWLWYIKIFIMIVGGLIGLRIVFTVLSIVNRVRQGYSPLS	706
8	EQELLALDKWASLWNWFDITNWLWYIKIFIMIVGGLIGLRIVFTVLSIVNRVRQGYSPLS	706
9	EQELLALDKWASLWNWFDITNWLWYIKIFIMIVGGLIGLRIVFTVLSIVNRVRQGYSPLS	706
10	EQELLALDKWASLWNWFDITNWLWYIKIFIMIVGGLIGLRIVFTVLSIVNRVRQGYSPLS	706
11	EQELLALDKWASLWNWFDITNWLWYIKIFIMIVGGLIGLRIVFTVLSIVNRVRQGYSPLS	706
12	EQELLALDKWASLWNWFDITNWLWYIKIFIMIVGGLIGLRIVFTVLSIVNRVRQGYSPLS	706
13	EQELLALDKWASLWNWFDITNWLWYIKIFIMIVGGLIGLRIVFTVLSIVNRVRQGYSPLS	706
14	EQELLALDKWASLWNWFDITNWLWYIKIFIMIVGGLIGLRIVFTVLSIVNRVRQGYSPLS	706
15	EQELLALDKWASLWNWFDITNWLWYIKIFIMIVGGLIGLRIVFTVLSIVNRVRQGYSPLS	706
16	EQELLALDKWASLWNWFDITNWLWYIKIFIMIVGGLIGLRIVFTVLSIVNRVRQGYSPLS	706
17	EQELLALDKWASLWNWFDITNWLWYIKIFIMIVGGLIGLRIVFTVLSIVNRVRQGYSPLS	706
18	EQELLALDKWASLWNWFDITNWLWYIKIFIMIVGGLIGLRIVFTVLSIVNRVRQGYSPLS	706
19	EQELLALDKWASLWNWFDITNWLWYIKIFIMIVGGLIGLRIVFTVLSIVNRVRQGYSPLS	706
20	EQELLALDKWASLWNWFDITNWLWYIKIFIMIVGGLIGLRIVFTVLSIVNRVRQGYSPLS	706
21	EQELLALDKWASLWNWFDITNWLWYIKIFIMIVGGLIGLRIVFTVLSIVNRVRQGYSPLS	706
22	EQELLALDKWASLWNWFDITNWLWYIKIFIMIVGGLIGLRIVFTVLSIVNRVRQGYSPLS	706
23	EQELLALDKWASLWNWFDITNWLWYIKIFIMIVGGLIGLRIVFTVLSIVNRVRQGYSPLS	706
24	EQELLALDKWASLWNWFDITNWLWYIKIFIMIVGGLIGLRIVFTVLSIVNRVRQGYSPLS	706
25	EQELLALDKWASLWNWFDITNWLWYIKIFIMIVGGLIGLRIVFTVLSIVNRVRQGYSPLS	706
26	EQELLALDKWASLWNWFDITNWLWYIKIFIMIVGGLIGLRIVFTVLSIVNRVRQGYSPLS	705
27	EQELLALDKWASLWNWFDITNWLWYIKIFIMIVGGLIGLRIVFTVLSIVNRVRQGYSPLS	706
28	EQELLALDKWASLWNWFDITNWLWYIKIFIMIVGGLIGLRIVFTVLSIVNRVRQGYSPLS	706
29	EQELLALDKWASLWNWFDITNWLWYIKIFIMIVGGLIGLRIVFTVLSIVNRVRQGYSPLS	706
30	EQELLALDKWASLWNWFDITNWLWYIKIFIMIVGGLIGLRIVFTVLSIVNRVRQGYSPLS	706
31	EQELLALDKWASLWNWFDITNWLWYIKIFIMIVGGLIGLRIVFTVLSIVNRVRQGYSPLS	706
32	EQELLALDKWASLWNWFDITNWLWYIKIFIMIVGGLIGLRIVFTVLSIVNRVRQGYSPLS	706
33	EQELLALDKWASLWNWFDITNWLWYIKIFIMIVGGLIGLRIVFTVLSIVNRVRQGYSPLS	706
34	EQELLALDKWASLWNWFDITNWLWYIKIFIMIVGGLIGLRIVFTVLSIVNRVRQGYSPLS	706
35	EQELLALDKWASLWNWFDITNWLWYIKIFIMIVGGLIGLRIVFTVLSIVNRVRQGYSPLS	706
36	EQELLALDKWASLWNWFDITNWLWYIKIFIMIVGGLIGLRIVFTVLSIVNRVRQGYSPLS	706
37	EQELLALDKWASLWNWFDITNWLWYIKIFIMIVGGLIGLRIVFTVLSIVNRVRQGYSPLS	706
38	EQELLALDKWASLWNWFDITNWLWYIKIFIMIVGGLIGLRIVFTVLSIVNRVRQGYSPLS	706

	FQTHLPAQRGPDRPEGIGEEGGERDRDRSDPLVNGFLTLIWSDLRSLCLFSYHRLRDLLL	
1	FQTHLPAQRGPDRPEGIGEEGGERDRDRSDPLVNGFLALIWSDLRSLCLFSYHRLRDLLL	766
2	FQTHLPAQRGPDRPEGIGEEGGERDRDRSDPLVNGFLTLIWSDLRSLCLFSYHRLRDLLL	766
3	FQTHLPAQRGPDRPEGIGEEGGERDRDRSDPLVNGFLTLIWSDLRSLCLFSYHRLRDLLL	766
4	FQTHLPAQRGPDRPEGIGEEGGERDRDRSDPLVNGFLTLIWSDLRSLCLFSYHRLRDLLL	766
5	FQTHLPAQRGPDRPEGIGEEGGERDRDRSDPLVNGFLTLIWSDLRSLCLFSYHRLRDLLL	766
6	FQTHLPAQRGPDRPEGIGEEGGERDRDRSDPLVNGFLTLIWSDLRSLCLFSYHRLRDLLL	766
7	FQTHLPAQRGPDRPEGIGEEGGERDRDRSDPLVNGFLTLIWSDLRSLCLFSYHRLRDLLL	766
8	FQTHLPAQRGPDRPEGIGEEGGERDRDRSDPLVNGFLTLIWSDLRSLCLFSYHRLRDLLL	766
9	FQTHLPAQRGPDRPEGIGEEGGERDRDRSDPLVNGFLTLIWSDLRSLCLFSYHRLRDLLL	766
10	FQTHLPAQRGPDRPEGIGEEGGERDRDRSDPLVNGFLTLIWSDLRSLCLFSYHRLRDLLL	766
11	FQTHLPAQRGPDRPEGIGEEGGERDRDRSDPLVNGFLTLIWSDLRSLCLFSYHRLRDLLL	766
12	FQTHLPAQRGPDRPEGIGEEGGERDRDRSDPLVNGFLTLIWSDLRSLCLFSYHRLRDLLL	766
13	FQTHLPAQRGPDRPEGIGEEGGERDRDRSDPLVNGFLTLIWSDLRSLCLFSYHRLRDLLL	766
14	FQTHLPAQRGPDRPEGIGEEGGERDRDRSDPLVNGFLTLIWSDLRSLCLFSYHRLRDLLL	766
15	FQTHLPAQRGPDRPEGIGEEGGERDRDRSDPLVNGFLTLIWSDLRSLCLFSYHRLRDLLL	766
16	FQTHLPAQRGPDRPEGIGEEGGERDRDRSDPLVNGFLTLIWSDLRSLCLFSYHRLRDLLL	766
17	FQTHLPAQRGPDRPEGIGEEGGERDRDRSDPLVNGFLTLIWSDLRSLCLFSYHRLRDLLL	766
18	FQTHLPAQRGPDRPEGIGEEGGERDRDRSDPLVNGFLTLIWSDLRSLCLFSYHRLRDLLL	766
19	FQTHLPAQRGPDRPEGIGEEGGERDRDRSDPLVNGFLTLIWSDLRSLCLFSYHRLRDLLL	766
20	FQTHLPAQRGPDRPEGIGEEGGERDRDRSDPLVNGFLTLIWSDLRSLCLFSYHRLRDLLL	766
21	FQTHLPAQRGPDRPEGIGEEGGERDRDRSDPLVNGFLTLIWSDLRSLCLFSYHRLRDLLL	766
22	FQTHLPAQRGPDRPEGIGEEGGERDRDRSDPLVNGFLTLIWSDLRSLCLFSYHRLRDLLL	766
23	FQTHLPAQRGPDRPEGIGEEGGERDRDRSDPLVNGFLTLIWSDLRSLCLFSYHRLRDLLL	766
24	FQTHLPAQRGPDRPEGIGEEGGERDRDRSDPLVNGFLTLIWSDLRSLCLFSYHRLRDLLL	766
25	FQTHLPAQRGPDRPEGIGEEGGERDRDRSDPLVNGFLTLIWSDLRSLCLFSYHRLRDLLL	766
26	FQTHLPAQRGPDRPEGIGEEGGERDRDRSDPLVNGFLTLIWSDLRSLCLFSYHRLRDLLL	765
27	FQTHLPAQRGPDRPEGIGEEGGERDRDRSDPLVNGFLTLIWSDLRSLCLFSYHRLRDLLL	766
28	FQTHLPAQRGPDRPEGIGEEGGERDRDRSDPLVNGFLTLIWSDLRSLCLFSYHRLRDLLL	766
29	FQTHLPAQRGPDRPEGIGEEGGERDRDRSDPLVNGFLTLIWSDLRSLCLFSYHRLRDLLL	766
30	FQTHLPAQRGPDRPEGIGEEGGERDRDRSDPLVNGFLTLIWSDLRSLCLFSYHRLRDLLL	766
31	FQTHLPAQRGPDRPEGIGEEGGERDRDRSDPLVNGFLTLI-SDLRSLCLFSYHRLRDLLL	765
32	FQTHLPAQRGPDRPEGIGEEGGERDRDRSDPLVNGFLTLIWSDLRSLCLFSYHRLRDLLL	766
33	FQTHLPAQRGPDRPEGIGEEGGERDRDRSDPLVNGFLTLIWSDLRSLCLFSYHRLRDLLL	766
34	FQTHLPAQRGPDRPEGIGEEGGERDRDRSDPLVNGFLTLIWSDLRSLCLFSYHRLRDLLL	766
35	FQTHLPAQRGPDRPEGIGEEGGERDRDRSDPLVNGFLTLIWSDLRSLCLFSYHRLRDLLL	766
36	FQTHLPAQRGPDRPEGIGEEGGERDRDRSDPLVNGFLTLIWSDLRSLCLFSYHRLRDLLL	766
37	FQTHLPAQRGPDRPEGIGEEGGERDRDRSDPLVNGFLTLIWSDLRSLCLFSYHRLRDLLL	766
38	FQTHLPAQRGPDRPEGIGEEGGERDRDRSDPLVNGFLTLIWSDLRSLCLFSYHRLRDLLL	766

	IVTRIVELLGR-------RGWEVLKYWWNLLQYWSQELKNSAVSLLNATATAVAEGTDRV	
1	IVTRIVELLGR-------RGWEVLKYWWNLLQYWSQELKNSAVSLLNATAIAVAEGTDRV	819
2	IVTRIVELLGR-------RGWEVLKYWWNLLQYWSQELKNSAVSLLNATATAVAEGTDRV	819
3	IVTRIVELLGR-------RGWEVLKYWWNLLQYWSQELKNSAVSLLNATATAVAEGTDRV	819
4	IVTRIVELLGR-------RGWEILKYWWNLLQYWSQELKNSAVSLLNATATAVAEGTDRV	819
5	IVTRIVELLGR-------RGWEVLKYWWNLLQYWSQELKNSAVSLLNATAIAVAEGTDRV	819
6	IVTRIVELLGR-------RGWEVLKYWWNLLQYWSQELKNSAVSLLNATATAVAEGTDRV	819
7	IVTRIVELLGR-------RGWEVLKYWWNLLQYWSQELKNSAVSLLNATATAVAEGTDRV	819
8	IVTRIVELLGR-------RGWEVLKYWWNLLQYWSQELKNSAVSLLNATATAVAEGTDRV	819
9	IVTRIVELLGR-------RGWEVLKYWWNLLQYWSQELKNSAVSLLNATATAVAEGTDRV	819
10	IVTRIVELLGR-------RGWEVLKYWWNLLQYWSQELKNSAVSLLNATATAVAEGTDRV	819
11	IVTRIVELLGR-------RGWEVLKYWWNLLQYWSQELKNSAVSLLNATATAVAEGTDRV	819
12	IVTRIVELLGR-------RGWEVLKYWWNLLQYWSQELKNSAVSLLNATATAVAEGTDRV	819
13	IVTRIVELLGR-------RGWEVLKYWWNLLQYWSQELKNSAVSLLNATATAVAEGTDRV	819
14	IVTRIVELLGR-------RGWEVLKYWWNLLQYWSQELKNSAVSLLNATATAVAEGTDRV	819
15	IVTRIVELLGR-------RGWEVLKYWWNLLQYWSQELKNSAVSLLNATAIAVAEGTDRV	819
16	IVTRIVELLGR-------RGWEVLKYWWNLLQYWSQELKNSAVSLLNATATAVAEGTDRV	819
17	IVTRIVELLGR-------RGWEVLKYWWNLLQYWSQELKNSAVSLLNATATAVAEGTDRV	819
18	IVTRIVELLGR-------RGWEVLKY-WNLLQYWSQELKNSAVSLLNATATAVAEGTDRV	818
19	IVTRIVELLGR-------RGWEVLKYWWNLLQYWSQELKNSAVSLLNATATAVAEGTDRV	819
20	IVTRIVELLGR-------RGWEVLKYWWNLLQYWSQELKNSAVSLLNATAIAVAEGTDRV	819
21	IVTRIVELLGR-------RGWEVLKYWWNLLQYWSQELKNSAVSLLNATATAVAEGTDRV	819
22	IVTRIVELLGR-------RGWEVLKYWWNLLQYWSQELKNSAVSLLNATATAVAEGTDRV	819
23	IVTRIVELLGR-------RGWEVLKYWWNLLQYWSQELKNSAVSLLNATATAVAEGTDRV	819
24	IVTRIVELLGR-------RGWEVLKYWWNLLQYWSQELKNSAVSLLNATATAVAEGTDRV	819
25	IVTRIVELLGR-------RGWEVLKYWWNLLQYWSQELKNSAVSLLNATATAVAEGTDRV	819
26	IVTRIVELLGR-------RGWEVLKYWWNLLQYWSQELKNSAVSLLNATATAVAEGTDRV	818
27	IVTRIVELLGR-------RGWEVLKYWWNLLQYWSQELKNSAVSLLNATATAVAEGTDRV	819
28	IVTRIVELLGR-------RGWEVLKYWWNLLQYWSQELKNSAVSLLNATATAVAEGTDRV	819
29	IVTRIVELLGR-------RGWEVLKYWWNLLQYWSQELKNSAVSLLNATATAVAEGTDRV	819
30	IVTRIVELLGR-------RGWEVLKYWWNLLQYWSQELKNSAVSLLNATATAVAEGTDRV	819
31	IVTRIVELLGR-------RGWEVLKYWWNLLQYWSQELKNSAVSLLNATATAVAEGTDRV	818
32	IVTRIVELLGR-------RGWEVLKYWWNLLQYWSQELKNSAVSLLNATATAVAEGTDRV	819
33	IVTRIVELLGR-------RGWEVLKYWWNLLQYWSQELKNSAVSLLNATATAVAEGTDRV	819
34	IVTRIVELLGR-------RGWEVLKYWWNLLQYWSQELKNSAVSLLNATATAVAEGTDRV	819
35	IVTRIVELLGR-------RGWEVLKYWWNLLQYWSQELKNSAVSLLNATATAVAEGTDRV	819
36	IVTRIVELLGR-------RGWEVLKYWWNLLQYWSQELKNSAVSLLNATATAVAEGTDRV	819
37	IVTRIVELLGR-------RGWEVLKYWWNLLQYWSQELKNSAVSLLNATATAVAEGTDRV	819
38	IVTRIVELLGR-------RGWEVLKYWWNLLQYWSQELKNSAVSLLNATATAVAEGTDRV	819

	IEVVQRACRAILHIPRRIRQGLERALL	
1	IEVVQRACRAILHIPRRIRQGLERALL	846
2	IEVVQRACRAILHIPRRIRQGLERALL	846
3	IEVVQRACRAILHIPRRIRQGLERALL	846
4	VEVVQRACRAILHIPRRIRQGLERALL	846
5	IEVVQRACRAILHIPRRIRQGLERALL	846
6	IEVVQRACRAILHIPRRIRQGLERALL	846
7	IEVVQRACRAILHIPRRIRQGLERALL	846
8	IEVVQRACRAILHIPRRIRQGLERALL	846
9	IEVVQRACRAILHIPRRIRQGLERALL	846
10	IEVVQRACRAILHIPRRIRQGLERALL	846
11	IEVVQRACRAILHIPRRIRQGLERALL	846
12	IEVVQRACRAILHIPRRIRQGLERALL	846
13	IEVVQRACRAILHIPRRIRQGLERALL	846
14	IEVVQRACRAILHIPRRIRQGLERALL	846
15	IEVVQRACRAIRHIPRRIRQGLERALL	846
16	IEVVQRACRAILHIPRRIRQGLERALL	846
17	IEVVQRACRAILHIPRRIRQGLERALL	846
18	VEVVQRACRAILHIPRRIRQGLERALL	845
19	IEVVQRACRAILHIPRRIRQGLERALL	846
20	IEVVQRACRAILHIPRRIRQGLERALL	846
21	VEVVQRACRAILHIPRRIRQGLERALL	846
22	IEVVQRACRAILHIPRRIRQGLERALL	846
23	IEVVQRACRAILHIPRRIRQGLERALL	846
24	IEVVQRACRAILHIPRRIRQGLERALL	846
25	IEVVQRACRAILHIPRRIRQGLERALL	846
26	IEVVQRACRAILHIPRRIRQGLERALL	845
27	IEVVQRACRAILHIPRRIRQGLERALL	846
28	IEVVQRACRAILHIPRRIRQGLERALL	846
29	IEVVQRACRAILHIPRRIRQGLERALL	846
30	IEVVQRACRAILHIPRRIRQGLERALL	846
31	VEVVQRACRAILHIPRRIRQGLERALL	845
32	IEVVQRACRAILHIPRRIRQGLERALL	846
33	IEVVQRACRAILHIPRRIRQGLERALL	846
34	IEVVQRACRAILHIPRRIRQGLERALL	846
35	VEVVQRACRAILHIPRRIRQGLERALL	846
36	IEVVQRACRAILHIPRRIRQGLERALL	846
37	IEVVQRACRAILHIPRRIRQGLERALL	846
38	IEVVQRACRAILHIPRRIRQGLERALL	846

Consensus

1. B.US.2006.700010040_C9_4520.EU289193
2. B.US.2008.CH0040_3_d0568_ipe032_2_01.MG900308
3. B.US.2008.CH0040_3_d0568_ipe032_3_03.MG900309
4. B.US.2008.CH0040_3_d0568_ipe032_3_04.MG900310
5. B.US.2008.CH0040_3_d0568_ipe032_3_06.MG900311
6. B.US.2008.CH0040_3_d0568_ipe032_3_13.MG900312
7. B.US.2008.CH0040_3_d0568_ipe032_3_15.MG900313
8. B.US.2008.CH0040_3_d0568_ipe032_3_17.MG900314
9. B.US.2008.CH0040_3_d0568_ipe032_3_18.MG900315
10. B.US.2008.CH0040_3_d0568_ipe032_3_20.MG900316
11. B.US.2008.CH0040_3_d0568_ipe032_3_21.MG900317
12. B.US.2008.CH0040_3_d0568_ipe032_3_22.MG900318
13. B.US.2008.CH0040_3_d0568_ipe032_3_25.MG900319
14. B.US.2008.CH0040_3_d0568_ipe032_3_26.MG900320
15. B.US.2008.CH0040_3_d0568_ipe032_3_27.MG900321
16. B.US.2008.CH0040_3_d0568_ipe032_3_29.MG900322
17. B.US.2008.CH0040_3_d0568_ipe032_3_30.MG900323
18. B.US.2008.CH0040_3_d0568_ipe032_3_32.MG900324
19. B.US.2008.CH0040_3_d0568_ipe032_3_33.MG900325
20. B.US.2008.CH0040_3_d0568_ipe032_3_34.MG900326
21. B.US.2008.CH0040_3_d0568_ipe032_3_36.MG900327
22. B.US.2008.CH0040_3_d0568_ipe032_3_38.MG900328
23. B.US.2008.CH0040_3_d0568_ipe032_3_39.MG900329
24. B.US.2008.CH0040_3_d0568_ipe032_3_40.MG900330
25. B.US.2008.CH0040_3_d0568_ipe032_3_42.MG900331
26. B.US.2008.CH0040_3_d0568_ipe032_3_43.MG900332
27. B.US.2008.CH0040_3_d0568_ipe032_3_44.MG900333
28. B.US.2008.CH0040_3_d0568_ipe032_3_47.MG900334
29. B.US.2008.CH0040_3_d0568_ipe032_3_49.MG900335
30. B.US.2008.CH0040_3_d0568_ipe032_3_50.MG900336
31. B.US.2008.CH0040_3_d0568_ipe032_3_52.MG900337
32. B.US.2008.CH0040_3_d0568_ipe032_3_53.MG900338
33. B.US.2008.CH0040_3_d0568_ipe032_3_54.MG900339
34. B.US.2008.CH0040_3_d0568_ipe032_3_56.MG900340
35. B.US.2008.CH0040_3_d0568_ipe032_3_58.MG900341

	MRVMGIRKNYQHLWREGILLLGILMICSAADNLWVTVYYGVPVWREATTTLFCASDAKAY	
1	MRVMGIRKNYQHLWREGILLLGILMICSAADNLWVTVYYGVPVWREATTTLFCASDAKAY	60
2	MRVMGIRKNYQHLWREGILLLGILMICSAADNLWVTVYYGVPVWREATTTLFCASDAKAY	60
3	MRVMGIRKNYQHLWREGILLLGILMICSAADNLWVTVYYGVPVWREATTTLFCASDAKAY	60
4	MRVMGIRKNYQHLWREGILLLGILMICSAADNLWVTVYYGVPVWREATTTLFCASDAKAY	60
5	MRVMGIRKNYQHLWREGILLLGILMICSAADNLWVTVYYGVPVWREATTTLFCASDAKAY	60
6	MRVMGIRKNYQHLWREGILLLGILMICSAADNLRVTVYYGVPVWREATTTLFCASDAKAY	60
7	MRVMGIRKNYQHLWREGILLLGILMICSAADNLWVTVYYGVPVWREATTTLFCASDAKAY	60
8	MRVMGIRKNYQHLWREGILLLGILMICSAADNLWVTVYYGVPVWREATTTLFCASDAKAY	60
9	MRVMGIRKNYQHLWREGILLLGILMICSAADNLWVTVYYGVPVWREATTTLFCASDAKAY	60
10	MRVMGIRKNYQHLWREGILLVGILMICSAADNLWVTVYYGVPVWREATTTLFCASDAKAY	60
11	MRVMGIRKNYQHLWREGILLLGLLMICSAADNLWVTVYYGVPVWREATTTLFCASDAKAY	60
12	MRVMGIRKNYQHLWREGILLLGILMICSAADNLWVTVYYGVPVWREATTTLFCASDAKAY	60
13	MRVMGIRKNYQHLWKEGILLLGILMICSAADNLWVTVYYGVPVWREATTTLFCASDAKAY	60
14	MRVMGIRKNYQHLWREGILLLGILMICSAADNLWVTVYYGVPVWREATTTLFCASDAKAY	60
15	MRVMGIRKNYQHLWREGILLLGILMICSAADNLWVTVYYGVPVWREATTTLFCASDAKAY	60
16	MRVMGIRKNYQHLWREGILLLGILMICSAADNLWVTVYYGVPVWREATTTLFCASDAKAY	60
17	MRVMGIRKNYQHLWREGILLLGILMICSAADNLWVTVYYGVPVWREATTTLFCASDAKAY	60
18	MRVMGIRKNYQHLWREGILLLGILMICSAADNLWVTVYYGVPVWREATTTLFCASDAKAY	60
19	MRVMGIRKNYQHLWREGILLLGILMICSAADNLWVTVYYGVPVWREATTTLFCASDAKAY	60
20	MRVMGIRKNYQHLWREGILLLGILMICSAADNLWVTVYYGVPVWREATTTLFCASDAKAY	60
21	MRVMGIRKNYQHLWREGILLLGILMICSAADNLWVTVYYGVPVWREATTTLFCASDAKAY	60
22	MRVMGIRKNYQHLWREGILLLGILMICSAADNLWVTVYYGVPVWREATTTLFCASDAKAY	60
23	MRVMGIRKNYQHLWREGILLLGILMICSAADNLWVTVYYGVPVWREATTTLFCASDAKAY	60
24	MRVMGIRKNYQHLWREGILLLGILMICSAADNLWVTVYYGVPVWREATTTLFCASDAKAY	60
25	MRVMGIRKNYQHLWREGILLLGILMICSAADNLWVTVYYGVPVWREATTTLFCASDAKAY	60
26	MRVMGIRKNYQHLWREGILLLGILMICSAADNLWVTVYYGVPVWREATTTLFCASDAKAY	60
27	MRVMGIRKNYQHLWREGILLLGILMICSAADNLWVTVYYGVPVWREATTTLFCASDAKAY	60
28	MRVMGIRKNYQHLWREGILLLGILMICSAADNLWVTVYYGVPVWREATTTLFCASDAKAY	60
29	MRVMGIRKNYQHLWREGILLLGILMICSAADNLWVTVYYGVPVWREATTTLFCASDAKAY	60
30	MRVMGIRKNYQHLWREGILLLGILMICSAADNLWVTVYYGVPVWREATTTLFCASDAKAY	60
31	MRVMGIRKNYQHLWREGILLLGILMICSAADNLWVTVYYGVPVWREATTTLFCASDAKAY	60
32	MRVMGIRKNYQHLWREGILLLGILMICSAADNLWVTVYYGVPVWREATTTLFCASDAKAY	60
33	MRVMGIRKNYQHLWREGILLLGILMICSAADNLWVTVYYGVPVWREATTTLFCASDAKAY	60
34	MRVMGIRKNYQHLWREGILLLGILMICSAADNLWVTVYYGVPVWR---------------	45
35	MRVMGIRKNYQHLWREGILLLGILMICSAADNLWVTVYYGVPVWREATTTLFCASDAKAY	60

```
    DTEAHNVWATHACVPTDPNPQEVELKNVTENFNMWENNMVEQMHEDIISLWDQSLKPCVK
1   DTEAHNVWATHACVPTDPNPQEVELKNVTENFNMWENNMVEQMHEDIISLWDQSLKPCVK  120
2   DTEAHNVWATHACVPTDPNPQEVELKNVTENFNMWENNMVEQMHEDIISLWDQSLKPCVK  120
3   DTEAHNVWATHACVPTDPNPQEVELKNVTENFNMWENNMVEQMHEDIISLWDQSLKPCVK  120
4   DTEAHNVWATHACVPTDPNPQEVELKNVTENFHMWENNMVEQMHEDIISLWDQSLKPCVK  120
5   DTEAHNVWATHACVPTDPNPQEVELKNVTENFNMWENNMVEQMHEDIISLWDQSLKPCVK  120
6   DTEAHNVWATHACVPTDPNPQEVELKNVTENFNMWENNMVEQMHEDIISLWDQSLKPCVK  120
7   DTEAHNVWATHDCVPTDPNPQEVELKNVTENFNMWENNMVEQMHEDIISLWDQSLKPCVK  120
8   DTEAHNVWATHACVPTDPNPQEVELKNVTENFNMWENNMVEQMHEDIISLWDQSLKPCVK  120
9   DTEAHNVWATHACVPTDPNPQEVELKNVTENFNMWENNMVEQMHEDIISLWDQSLKPCVK  120
10  DTEAHNVWATHACVPTDPNPQEVELKNVTENFNMWENNMVEQMHEDIISLWDQSLKPCVK  120
11  DTEAHNVWATHACVPTDPNPQEVELKNVTENFNMWENNMVEQMHEDIISLWDQSLKPCVK  120
12  DTEAHNVWATHACVPTDPNPQEVELKNVTENFNMWENNMVEQMHEDIISLWDQSLKPCVK  120
13  DTEAHNVWATHACVPTDPNPQEVELKNVTENFNMWENNMVEQMHEDIISLWDQSLKPCVK  120
14  DTEAHNVWATHACVPTDPNPQEVELKNVTENFNMWENNMVEQMHEDIISLWDQSLKPCVK  120
15  DTEAHNVWATHACVPTDPNPQEVELKNVTENFNMWENNMVEQMHEDIISLWDQSLKPCVK  120
16  DTEAHNVWATHACVPTDPNPQEVELKNVTENFNMWENNMVEQMHEDIISLWDQSLKPCVK  120
17  DTEAHNVWATHACVPTDPNPQEVELKNVTENFNMWENNMVEQMHEDIISLWDQSLKPCVK  120
18  DTEAHNVWATHACVPTDPNPQEVELKNVTENFNMWENNMVEQMHEDIISLWDQSLKPCVK  120
19  DTEAHNVWATHACVPTDPNPQEVELKNVTENFNMWENNMVEQMHEDIISLWDQSLKPCVK  120
20  DTEAHNVWATHACVPTDPNPQEVELKNVTENFNMWENNMVEQMHEDIISLWDQSLKPCVK  120
21  DTEAHNVWATHACVPTDPNPQEVELKNVTENFNMWENNMVEQMHEDIISLWDQSLKPCVK  120
22  DTEAHNVWATHACVPTDPNPQEVELKNVTENFNMWENNMVEQMHEDIISLWDQSLKPCVK  120
23  DTEAHNVWATHACVPTDPNPQEVELKNVTENFNMWENNMVEQMHEDIISLWDQSLKPCVK  120
24  DTEAHNVWATHACVPTDPNPQEVELKNVTENFNMWENNMVEQMHEDIISLWDQSLKPCVK  120
25  DTEAHNVWATHACVPTDPNPQEVELKNVTENFNMWENNMVEQMHEDIISLWDQSLKPCVK  120
26  DTEAHNVWATHACVPTDPNPQEVELKNVTENFNMWENNMVEQMHEDIISLWDQSLKPCVK  120
27  DTEAHNVWATHACVPTDPNPQEVELKNVTENFNMWENNMVEQMHEDIISLWDQSLKPCVK  120
28  DTEAHNVWATHACVPTDPNPQEVELKNVTENFNMWENNMVEQMHEDIISLWDQSLKPCVK  120
29  DTEAHNVWATHACVPTDPNPQEVELKNVTENFNMWENNMVEQMHEDIISLWDQSLKPCVK  120
30  DTEAHNVWATHACVPTDPNPQEVELKNVTENFNMWENNMVEQMHEDIISLWDQSLKPCVK  120
31  DTEAHNVWATHACVPTDPNPQEVELKNVTENFNMWENNMVEQMHEDIISLWDQSLKPCVK  120
32  DTEAHNVWATHACVPTDPNPQEVELKNVTENFNMWENNMVEQMHEDIISLWDQSLKPCVK  120
33  DTEAHNVWATHACVPTDPNPQEVELKNVTENFNMWENNMVEQMHEDIISLWDQSLKPCVK  120
34  --------ATHACVPTDPNPQEVELKNVTENFNMWENNMVEQMHEDIISLWDQSLKPCVK  97
35  DTEAHNVWATHACVPTDPNPQEVELKNVTENFNMWENNMVEQMHEDIISLWDQSLKPCVK  120
```

	Sequence	
	**LTPLCVTLNCTDLEKVTNTT---NSTEKMMEKGEVKNCSFKITTDVKDRTRKEYALFYKL**	
1	LTPLCVTLNCTDLGNVTNTT---NSNGEMMEKGEVKNCSFKITTDIKDRTRKEYALFYKL	177
2	LTPLCVTLNCTDLEKVTNTT---NSTEKMMEKGEVKNCSFKITTDVKDRTRKEYALFYKL	177
3	LTPLCVTLNCTDLEKVTNTT---NSTEKMMEKGEVKNCSFKITTDVKDRTRKEYALFYKL	177
4	LTPLCVTLNCTDLEKVTNTT---NSTEKMMEKGEVKNCSFKITTDVKDRTRKEYALFYKL	177
5	LTPLCVTLNCTDLEKVANTT---NSTEKMMEKGEVKNCSFKITTDVKDRTRKEYALFYKL	177
6	LTPLCVTLNCTDLEKVTNTT---NSTEKMMEKGEVKNCSFKITTDVKDRTRKEYALFYKL	177
7	LTPLCVTLNCTDLENVTDTT---NSTEKMMEKGEVKNCSFKITTDLKDRTRKEYALFYKL	177
8	LTPLCVTLNCTDLEKVTNTT---NSTEKMMEKGEVKNCSFKITTDVKDRTRKEYALFYKL	177
9	LTPLCVTLNCTDLGNVTTTT---NSNGTLMEKGEVKNCSFKITTDTKDRTRKEYALFYKL	177
10	LTPLCVTLNCTDLEKVTNTT---NSTEKMMEKGEVKNCSFKITTDVKDRTRKEYALFYKL	177
11	LTPLCVTLNCTDLEKVTNTT---NSTEKMMEKGEVKNCSFKITTDVKDRTRKEYALFYKL	177
12	LTPLCVTLNCTDLEKVTNTT---NSTEKMMEKGEVKNCSFKITTDVKDRTRKEYALFYKL	177
13	LTPLCVTLNCTDLEKVTNTT---NSTEKMMEKGEVKNCSFKITTDMKDRTRKEYALFYKL	177
14	LTPLCVTLNCTDLEKVTNTT---NSTEKMMEKGEVKNCSFKITTDVKDRTRKEYALFYKL	177
15	LTPLCVTLNCTDLGNVTATT---NSNGTLMEKGEVKNCSFKITTDIKDRTRKEYALFYKL	177
16	LTPLCVTLNCTDLEKVTNTT---NSTEKMMEKGEVKNCSFKITTDVKDRTRKEYALFYKL	177
17	LTPLCVTLNCTDLEKVTNTT---NSTEKMMEKGEVKNCSFRITTDIKDRTRKEYALFYKL	177
18	LTPLCVTLNCTDLGNVTTTT---NSNGTLMEKGEVKNCSFKITTEIKDRTRKEYALFYKL	177
19	LTPLCVTLNCTDLGNVTTTT---NSNGTLMEKGEVKNCSFKITTDVKDRTRKEYALF---	174
20	LTPLCVTLNCTDLEKVTNTT---NSTEKMMEKGEVKNCSFKITTDVKDRTRKEYALFYKL	177
21	LTPLCVTLNCTDLEKVTNTT---NSTEKMMEKGEVKNCSFKITTDRKDRTRKEYALFYKL	177
22	LTPLCVTLNCTDLGNVTTTT---NSNGTLMEKGEVKNCSFKITTEIKDRTRKEYALFYKL	177
23	LTPLCVTLNCTDLEKVTNTT---NSTEKMMEKGEVKNCSFKITTDVKDRTRKEYALFYKL	177
24	LTPLCVTLNCTDLEKVTNTT---NSNETMMEKGEVKNCSFKITTDIKDRTRKEYALFYKL	177
25	LTPLCVTLNCTDLEKVTNTT---NSTEKMMEKGEVKNCSFKITTDVKDRTRKEYALFYKL	177
26	LTPLCVTLNCTDLGNVTTTT---NSNGTLMEKGEVKNCSFKITTDTKDRTRKEYALFYKL	177
27	LTPLCVTLNCTDLGNVTTTT---NSNGTLMEKGEVKNCSFKITTEIKDRTRKEYALFYKL	177
28	LTPLCVTLNCTDLEKVTNTT---NSTEKMMEKGEVKNCSFKITTDVKDRTRKEYALFYKL	177
29	LTPLCVTLNCTDLEKVTNTT---NSTEKMMEKGEVKNCSFKITTDVKDRTRKEYALFYKL	177
30	LTPLCVTLNCTDLEKVTNTT---NSTEKMMEKGEVKNCSFKITTDVKDRTRKEYALFYKL	177
31	LTPLCVTLNCTDLEKVTNTT---NSTEKMMEKGEVKNCSFKITTDVKDRTRKEYALFYKL	177
32	LTPLCVTLNCTDLEKVTNTT---NSTEKMMEKGEVKNCSFKITTDVKDRTRKEYALFYKL	177
33	LTPLCVTLNCTDLEKVTNTT---NSTEKMMEKGEVKNCSFKITTDVKDRTRKEYALFYKL	177
34	LTPLCVTLNCTDLGNVTTTT---NSNGTLMEKGEVKNCSFKITTDTKDRTRKEYALFYKL	154
35	LTPLCVTLNCTDLEKVTNTT---NSTEKMMEKGEVKNCSFKITTDVKDRTRKEYALFYKL	177

```
     DVVPIND-------TRYRLVSCNTSVITQACPKVSFEPIPIHYCAPAGFAILKCNDKQFI
1    DVVPIND-------TRYRLVSCNTSVITQACPKVSFEPIPIHYCAPAGFAILKCNDKQFI  230
2    DVVPIND-------TRYRLVSCNTSVITQACPKVSFEPIPIHYCAPAGFAILKCNDKQFI  230
3    DVVPIND-------TRYRLVSCNTSVITQACPKVSFEPIPIHYCAPAGFAILKCNDKQFI  230
4    DVVPIND-------TRYRLVSCNTSVITQACPKVSFEPIPIHYCAPAGFAILKCNDKQFI  230
5    DVVPIND-------TRYRLVSCNTSVITQACPKVSFEPIPIHYCAPAGFAILKCNDKQFI  230
6    DVVPIND-------TRYRLVSCNTSVITQACPKVSFEPIPIHYCAPAGFAILKCNDKQFN  230
7    DVVPIND-------TRYRLVSCNTSVITQACPKVSFEPIPIHYCAPAGFAILKCNDKQFI  230
8    DVVPIND-------TRYRLVSCNTSVITQACPKVSFEPIPIHYCAPAGFAILKCNDKQFI  230
9    DVVPIND-------TRYRLVSCNTSVITQACPKVSFEPIPIHYCAPAGFAILKCNDKQFI  230
10   DVVPIND-------TRYRLVSCNTSVITQACPKVSFEPIPIHYCAPAGFAILKCNDKQFI  230
11   DVVPIND-------TRYRLVSCNTSVITQACPKVSFEPIPIHYCAPAGFAILKCNDKQFI  230
12   DVVPIND-------TRYRLVSCNTSVITQACPKVSFEPIPIHYCAPAGFAILKCNDKQFI  230
13   DVVPIND-------TRYRLVSCNTSVITQACPKVSFEPIPIHYCAPAGFAILKCNDKQFI  230
14   DVVPIND-------TRYRLVSCNTSVITQACPKVSFEPIPIHYCAPAGFAILKCNDKQFI  230
15   DVVPIND-------TRYRLVSCNTSVITQACPKVSFEPIPIHYCAPAGFAILKCNDKQFM  230
16   DVVPIND-------TRYRLVSCNTSVITQACPKVSFEPIPIHYCAPAGFAILKCNDKQFI  230
17   DVVPIND-------TRYRLVSCNTSVITQACPKVSFEPIPIHYCAPAGFAILKCNDKQFI  230
18   DVVPIND-------TRYRLVSCNTSVITQACPKVSFEPIPIHYCAPAGFAILKCNDKQFI  230
19   --------------------SCNTSVITQACPKVSFEPIPIHYCAPAGFAILKCNDKQFI  214
20   DVVPIND-------TRYRLVSCNTSVITQACPKVSFEPIPIHYCAPAGFAILKCNDKQFI  230
21   DVVPIND-------TRYRLVSCNTSVITQACPKVSFEPIPIHYCAPAGFAILKCNDKQFM  230
22   DVVPIND-------TRYRLVSCNTSVITQACPKVSFEPIPIHYCAPAGFAILKCNDKQFI  230
23   DVVPIND-------TRYRLVSCNTSVITQACPKVSFEPIPIHYCAPAGFAILKCNDKQFI  230
24   DVVPIND-------TRYRLVSCNTSVITQACPKVSFEPIPIHYCAPAGFAILKCNDKQFI  230
25   DVVPIND-------TRYRLVSCNTSVITQACPKVSFEPIPIHYCAPAGFAILKCNDKQFI  230
26   DVVPIND-------TRYRLVSCNTSVITQACPKVSFEPIPIHYCAPAGFAILKCNDKQFI  230
27   DVVPIND-------TRYRLVSCNTSVITQACPKVSFEPIPIHYCAPAGFAILKCNDKQFI  230
28   DVVPIND-------TRYRLVSCNTSVITQACPKVSFEPIPIHYCAPAGFAILKCNDKQFI  230
29   DVVPIND-------TRYRLVSCNTSVITQACPKVSFEPIPIHYCAPAGFAILKCNDKQFI  230
30   DVVPIND-------TRYRLVSCNTSVITQACPKVSFEPIPIHYCAPAGFAILKCNDKQFI  230
31   DVVPIND-------TRYRLVSCNTSVITQACPKVSFEPIPIHYCAPAGFAILKCNDKQFI  230
32   DVVPIND-------TRYRLVSCNTSVITQACPKVSFEPIPIHYCAPAGFAILKCNDKQFI  230
33   DVVPIND-------TRYRLVSCNTSVITQACPKVSFEPIPIHYCAPAGFAILKCNDKQFI  230
34   DVVPIND-------TRYRLVSCNTSVITQACPKVSFEPIPIHYCAPAGFAILKCNDKQFI  207
35   DVVPIND-------TRYRLVSCNTSVITQACPKVSFEPIPIHYCAPAGFAILKCNDKQFI  230
```

	Sequence	
	**GTGPCTNVSTVQCTHGIRPVVSTQLLLNGSLAEEEVVIRSVNFSDNAKTIIVQLNKSVEI**	
1	GTGPCTNVSTVQCTHGIRPVVSTQLLLNGSLAEEEVVIRSVNFSDNAKTIIVQLNKSVEI	290
2	GTGPCTNVSTVQCTHGIRPVVSTQLLLNGSLAEEEVVIRSVNFRDNAKTIIVQLNKSVEI	290
3	GTGPCTNVSTVQCTHGIRPVVSTQLLLNGSLAEEEVVIRSVNFRDNAKTIIVQLNKSVEI	290
4	GTGPCTNVSTVQCTHGIRPVVSTQLLLNGSLAEEEVVIRSVNFRDNAKTIIVQLNKSVEI	290
5	GTGPCTNVSTVQCTHGIRPVVSTQLLLNGSLAEEEVVIRSVNFSDNAKTIIVQLNKSVEI	290
6	GTGPCTNVSTVQCTHGIRPVVSTQLLLNGSLAEEEVVIRSVNFSDNAKTIIVQLNKSVEI	290
7	GTGPCTNVSTVQCTHGIRPVVSTQLLLNGSLAEEEVVIRSVNFRDNAKTIIVQLNKSVEI	290
8	GTGPCTNVSTVQCTHGIRPVVSTQLLLNGSLAEEEVVIRSVNFRDNAKTIIVQLNKSVEI	290
9	GTGPCTNVSTVQCTHGIRPVVSTQLLLNGSLAEEEVVIRSVNFSDNAKTIIVQLNKSVEI	290
10	GTGPCTNVSTVQCTHGIRPVVSTQLLLNGSLAEEEVVIRSVNFSDNAKTIIVQLNKSVEI	290
11	GTGPCTNVSTVQCTHGIRPVVSTQLLLNGSLAEEEVVIRSVNFSDNAKTIIVQLNKSVEI	290
12	GTGPCTNVSTVQCTHGIRPVVSTQLLLNGSLAEEEVVIRSVNFSDNAKTIIVQLNKSVEI	290
13	GTGPCTNVSTVQCTHGIRPVVSTQLLLNGSLAEEEVVIRSVNFRDNAKTIIVQLNKSVEI	290
14	GTGPCTNVSTVQCTHGIRPVVSTQLLLNGSLAEEEVVIRSVNFSDNAKTIIVQLNKSVEI	290
15	GTGPCTNVSTVQCTHGIRPVVSTQLLLNGSLAEEEVVIRSVNFSDNAKTIIVQLNKSVEI	290
16	GTGPCTNVSTVQCTHGIRPVVSTQLLLNGSLAEEEVVIRSVNFRDNAKTIIVQLNKSVEI	290
17	GTGPCTNVSTVQCTHGIRPVVSTQLLLNGSLAEEEVVIRSVNFSDNAKTIIVQLNKSVEI	290
18	GTGPCTNVSTVQCTHGIRPVVSTQLLLNGSLAEEEVVIRSVNFRDNAKTIIVQLNKSVEI	290
19	GTGPCTNVSTVQCTHGIRPVVSTQLLLNGSLAEEEVVIRSVNFSDNAKTIIVQLNKSVEI	274
20	GTGPCTNVSTVQCTHGIRPVVSTQLLLNGSLAEEEVVIRSVNFRDNAKTIIVQLNKSVEI	290
21	GTGPCTNVSTVQCTHGIRPVVSTQLLLNGSLAEEEVVIRSVNFSDNAKTIIVQLNKSVEI	290
22	GTGPCTNVSTVQCTHGIRPVVSTQLLLNGSLAEEEVVIRSVNFRDNAKTIIVQLNKSVEI	290
23	GTGPCTNVSTVQCTHGIRPVVSTQLLLNGSLAEEEVVIRSVNFRDNAKTIIVQLNKSVEI	290
24	GTGPCTNVSTVQCTHGIRPVVSTQLLLNGSLAEEEVVIRSVNFSDNAKTIIVQLNKSVEI	290
25	GTGPCTNVSTVQCTHGIRPVVSTQLLLNGSLAEEEVVIRSVNFRDNAKTIIVQLNKSVEI	290
26	GTGPCTNVSTVQCTHGIRPVVSTQLLLNGSLAEEEVVIRSVNFSDNAKTIIVQLNKSVEI	290
27	GTGPCTNVSTVQCTHGIRPVVSTQLLLNGSLAEEEVVIRSVNFRDNAKTIIVQLNKSVEI	290
28	GTGPCTNVSTVQCTHGIRPVVSTQLLLNGSLAEEEVVIRSVNFRDNAKTIIVQLNKSVEI	290
29	GTGPCTNVSTV--------------------------IRSVNFSDNAKTIIVQLNKSVEI	264
30	GTGPCTNVSTVQCTHGIRPVVSTQLLLNGSLAEEEVVIRSVNFRDNAKTIIVQLNKSVEI	290
31	GTGPCTNVSTVQCTHGIRPVVSTQLLLNGSLAEEEVVIRSVNFSDNAKTIIVQLNKSVEI	290
32	GTGPCTNVSTVQCTHGIRPVVSTQLLLNGSLAEEEVVIRSVNFSDNAKTIIVQLNKSVEI	290
33	GTGPCTNVSTVQCTHGIRPVVSTQLLLNGSLAEEEVVIRSVNFSDNAKTIIVQLNKSVEI	290
34	GTGPCTNVSTVQCTHGIRPVVSTQLLLNGSLAEEEVVIRSVNFSDNAKTIIVQLNKSVEI	267
35	GTGPCTNVSTVQCTHGIRPVVSTQLLLNGSLAEEEVVIRSVNFRDNAKTIIVQLNKSVEI	290

	Sequence	
	NCTRPHNNTRKSIPMGPGKAFYARGDIIGDIRKAYCEINGTEWHSTLKLVVEKLREQY-N	
1	TCTRPNNNTRKSIPMGPGKAFYARGDITGDIRKAYCEINGTEWHSTLKLVVEKLREQY-N	349
2	NCTRPHNNTRKSIPMGPGKAFYARGDIIGDIRKAYCEVNGTEWHSTLKLVVEKLREQY-N	349
3	NCTRPHNNTRKSIPMGPGKAFYARGDIIGDIRKAYCEINGTEWHSTLKLVVEKLREQY-N	349
4	NCTRPHNNTRKSIPMGPGKAFYARGDIIGDIRKAYCEINGTEWHSTLKLVVEKLREQY-N	349
5	NCTRPHNNTRKSIPMGPGKAFYARGDIIGDIRKAYCEINGTEWHSTLKLVVEKLREKY-N	349
6	NCTRPHNNTRKSIPMGPGKAFYARGDIIGDIRKAYCNINGTEWHSTLKLVVEKLREQY-N	349
7	NCARPHNNTRKSIPMGPGKAFYARGDITGDIRKAYCEINGTEWHSTLKLVVEKLREQY-N	349
8	NCTRPHNNTRKSIPMGPGKAFYARGDIIGDIRKAYCEINGTEWHSTLKLVVEKLREQY-N	349
9	NCTRPNNNTRKSIPMGPGKAFYARGDIIGDIRKASCELNGTEWHSTLKLVVEKLRKQY-N	349
10	NCTRPHNNTRKSIPMGPGKAFYARGDIIGDIRKAYCEINGTEWHSTLKLVVGKLREKY-N	349
11	NCTRPNNNTRKSIPMGPGKAFYARGDIIGDIRKASCKINGTEWHSTLKLVVEKLREQY-N	349
12	NCTRPHNNTRKSIPMGPGKAFYARGDIIGDIRKAYCEINGTEWHSTLKLVVGKLREKY-N	349
13	NCTRPHNNTRKSIPMGPGKAFYARGDIIGDIRKAYCEINGTEWHSTLKLVVEKLREQY-N	349
14	NCTRPHNNTRKSIPMGPGKAFYARGDIIGDIRKAYCEINGTEWHSTLKLVVGKLREKY-N	349
15	TCTRPNNNTRKSIPMGPGKAFYARGDIIGDIRKASCKINGTEWHSTLKLVVEKLREQY-N	349
16	NCTRPHNNTRKSIPMGPGKAFYARGDIIGDIRKAYCEINGTEWHSTLKLVVEKLREQY-N	349
17	NCTRPHNNTRKSIPMGPGKAFYARGDIIGDIRKAYCEINGTEWHSTLKLVVEKLREQY-N	349
18	NCTRPHNNTRKSIPMGPGKAFYARGDIIGDIRKAYCTINGTEWHSTLKLVVEKLREQY-N	349
19	NCTRPHNNTRKSIPMGPGKAFYARGDIIGDIRKAYCKINGTEWHSTLKLVVEKLREQY-N	333
20	NCTRPHNNTRKSIPMGPGKAFYARGDIIGDIRKAYCEINGTEWHSTLKLVVEKLREQY-N	349
21	NCTRPHNNTRKSIPMGPGKAFYARGDITGDIRKAYCEINGTEWHSTLKLVVEKLREQY-N	349
22	NCTRPHNNTRKSIPMGPGKAFYARGDIIGNIRKAYCEINGTEWHSTLKLVVEKLREQY-N	349
23	NCTRPHNNTRKSIPMGPGKAFYARGDIIGDIRKAYCEINGTEWHSTLKLVVEKLREQY-N	349
24	TCTRPNNNTRKSIPMGPGKAFYARGDIIGDIRKASCKINGTEWHSTLKLVVEKLREKY-N	349
25	NCTRPHNNTRKSIPMGPGKAFYARGDIIGDIRKAYCEINGTEWHSTLKLVVEKLREQY-N	349
26	NCTRPNNNTRKSIPMGPGKAFYARGDIIGDIRKASCELDGTEWHSTLKLVVEKLRKQY-N	349
27	NCTRPHNNTRKSIPMGPGKAFYARGDIIGDIRKAYCEINGAEWHSTLKLVVEKLKEQY-N	349
28	NCTRPHNNTRKSIPMGPGKAFYARGDIIGDIRKAYCEINGTEWHSTLKLVVEKLREQY-N	349
29	NCTRPNNNTRKSIPMGPGKAFYARGDIIGDIRKASCKINGTEWHSTLKLVVEKLREQY-N	323
30	NCTRPHNNTRKSIPMGPGKAFYARGDIIGDIRKAYCEVNGTEWHSTLKLVVEKLREQY-N	349
31	NCTRPHNNTRKSIPMGPGKAFYARGDIIGDIRKAYCAINGTEWHSTLKLVVEKLKEQY-N	349
32	NCTRPNNNTRKSIPMGPGKAFYARGDIIGDIRKASCKINGTEWHSTLKLVVEKLREQY-N	349
33	NCTRPNNNTRKSIPMGPGKAFYARGDIIGDIRKASCKINGTEWHSTFKLVVEKLRAQY-N	349
34	NCTRPNNNTRKSIPMGPGKAFYARGDIIGDIRKASCELDGTEWHSTLKLVVEKLRKQY-N	326
35	NCTRPHNNTRKSIPMGPGKAFYARGDIIGDIRKAYCEINGTEWHSTLKLVVEKLREQY-N	349

```
    KTIVFNRSSGGDPEIVMYSFNCGGEFFYCNSTKLFNSTWPWND-TKGSHDTNGTLILPCR
1   KTIVFNRSSGGDPEIVMYSFNCGGEFFYCNSTKLFNSTWPWND-TKGSHDTNGTLILPCK  408
2   KTIVFNRSSGGDPEIVMYSFNCGGEFFYCNSTKLFNSTWPWND-TKGSHDTNGKLILPCR  408
3   KTIVFNRSSGGDPEIVMYSFNCGGEFFYCNSTKLFNSTWPWND-TKGSHDTNGKLILPCR  408
4   KTIVFNRSSGGDPEIVMYSFNCGGEFFYCNSTKLFNSTWPWND-TKGSHDTNGKLILPCR  408
5   KTIVFNRSSGGDPEIVMYSFNCGGEFFYCNSTKLFNSTWPWND-TKGSHDTNDTLMLPCK  408
6   KTIVFNRSSGGDPEIVMYSFNCGGEFFYCNSTKLFNSTWPWND-TKGSHDTNDTLMLPCK  408
7   KTIVFNRSSGGDPEIVMYSFNCGGEFFYCNSTKLFNSTWPWND-TKGSHDTNGKLILPCK  408
8   KTIVFNRSSGGDPEIVMYSFNCGGEFFYCNSTKLFNSTWPWND-TKGSHDTNGKLILPCR  408
9   KTIVFNRSSGGDPEIVMYSFNCGGEFFYCNSTKLFNSTWPWND-TKGSHDTNGTLILPCR  408
10  KTIVFNRSSGGDPEIVMYSFNCGGEFFYCNSTKLFNSTWPWND-TKGSHDTNDTLMLPCK  408
11  KTIVFNRSSGGDPEIVMYSFNCGGEFFYCNSTKLFNSTWPWND-TKGSHDTNDTLMLPCK  408
12  KTIVFNRSSGGDPEIVMYSFNCGGEFFYCNSTKLFNSTWPWND-TKGSHDTNDTLMLPCK  408
13  KTIVFNRSSGGDPEIVMYSFNCGGEFFYCNSTKLFNSTWPWND-TKGSHDTNDTLMLPCK  408
14  KTIVFNRSSGGDPEIVMYSFNCGGEFFYCNSTKLFNSTWPWSD-TKGSHDTNDTLMLPCK  408
15  KTIVFNRSSGGDPEIVMYSFNCGGEFFYCNSTKLFNSTWPWND-TKGSHDTNDTLMLPCK  408
16  KTIVFNRSSGGDPEIVMYSFNCGGEFFYCNSTKLFNSTWPWND-TKGSHDTNGKLILPCR  408
17  KTIVFNRSSGGDPEIVMYSFNCGGEFFYCNSTKLFNSTWPWND-TKGSHDTNDTLMLPCK  408
18  KTIVFNRSSGGDPEIVMYSFNCGGEFFYCNSTKLFNSTWPWND-TKGSHDTNDTLMLPCK  408
19  KTIVFNRSSGGDPEIVMYSFNCGGEFFYCNSTKLFNSTWPWND-TKGSHDTNDTLMLPCR  392
20  KTIVFNRSSGGDPEIVMYSFNCGGEFFYCNSTKLFNSTWPWND-TKGSHDTNGKLILPCR  408
21  KTIVFNRSSGGDPEIVMYSFNCGGEFFYCNSTKLFNSTWPWND-TKGSHDTNGKLILPCR  408
22  KTIVFNRSSGGDPEIVMYSFNCGGEFFYCNSTKLFNSTWPWND-TKGSHDTNGRLILPCR  408
23  KTIVFNRSSGGDPEIVMYSFNCGGEFFYCNSTKLFNSTWPWND-TKGSHDTNGKLILPCR  408
24  KTIVFNRSSGGDPEIVMYSFNCGGEFFYCNSTKLFNSTWPWSD-TKGSHDTNDTLMLPCR  408
25  KTIVFNRSSGGDPEIVMYSFNCGGEFFYCNSTKLFNSTWPWND-TKGSHDTNGSLILPCR  408
26  KTIVFNRSSGGDPEIVMYSFNCGGEFFYCNSTKLFNSTWPWND-TKGSHDTNGTLILPCR  408
27  KTIVFNRSSGGDPEIVMYSFNCGGEFFYCNSTKLFNSTWPWND-TKGSHDINGTLILPCK  408
28  KTIVFNRSSGGDPEIVMYSFNCGGEFFYCNSTKLFNSTWPWND-TKGSHDTNGKLILPCR  408
29  KTIVFNRSSGGDPEIVMYSFNCGGEFFYCNSTKLFNSTWPWND-TKGSHDTNDTLMLPCK  382
30  KTIVFNRSSGGDPEIVMYSFNCGGEFFYCNSTKLFNSTWPWND-TKGSHDTNGKLILPCR  408
31  KTIVFNRSSGGDPEIVMYSFNCGGEFFYCNSTKLFNSTWPWND-TKGSHDTNGTLILPCK  408
32  KTIVFNRSSGGDPEIVMYSFNCGGEFFYCNSTKLFNSTWPWND-TKGSHDTNDTLMLPCK  408
33  KTIVFSRSSGGDPEIVMYSFNCGGDFFYCNSTQLFNSTWPWND-TKGSHDTNDTLMLPCK  408
34  KTIVFNRSSGGDPEIVMYSFNCGGEFFYCNSTKLFNSTWPWND-TKGSHDTNGTLILPCR  385
35  KTIVFNRSSGGDPEIVMYSFNCGGEFFYCNSTKLFNSTWPWND-TKGSHDTNGKLILPCR  408
```

```
    IKQIINMWQGVGKAMYAPPIEGXIRCSSNITGLLLTRDGG-YESNETDEIFRPGGGDMRD
1   IKQIINMWQGVGKAMYAPPIEGKIRCSSNITGLLLTRDGG-YESNETDEIFRPGGGDMRD  467
2   IKQIINMWQGVGKAMYAPPIEGEIRCSSNITGLLLTRDGG-YESNKTDEIFRPGGGDMRD  467
3   IKQIINMWQGVGKAMYAPPIEGEIRCSSNITGLLLTRDGG-YESNKTDEIFRPGGGDMRD  467
4   IKQIINMWQGVGKAMYAPPIEGEIRCSSNITGLLLTRDGG-YESNKTDEIFRPGGGDMRD  467
5   IKQIINMWQGVGKAMYAPPIEGQIRCSSNITGLLLTRDGG-YESNETDEIFRPGGGDMRD  467
6   IKQIINMWQGVGKAMYAPPIEGQIRCSSNITGLLLTRDGG-YESNETDEIFRPGGGDMRD  467
7   IKQIINMWQGVGKAMYAPPIEGKIRCSSNITGLLLTRDGG-YESNETDEIFRPGGGDMRD  467
8   IKQIINMWQGVGKAMYAPPIEGEIRCSSNITGLLLTRDGG-YESNKTDEIFRPGGGDMRD  467
9   IKQIINMWQGVGKAMYAPPIEGEIRCSSNITGLLLTRDGG-YESNETDEIFRPGGGDMRD  467
10  IKQIINMWQGVGKAMYAPPIEGQIRCSSNITGLLLTRDGG-YESNETDEIFRPGGGDMRD  467
11  IKQIINMWQGVGKAMYAPPIEGIIRCSSNITGLLLTRDGG-YESNETDEIFRPGGGDMRD  467
12  IKQIINMWQGVGKAMYAPPIEGQIRCSSNITGLLLTRDGG-YESNETDEIFRPGGGDMRD  467
13  IKQIINMWQGVGKAMYAPPIEGQIRCSSNITGLLLTRDGG-YESNETDEIFRPGGGDMRD  467
14  IKQIINMWQGVGKAMYAPPIEGQIRCSSNITGLLLTRDGG-YESNETDEIFRPGGGDMRD  467
15  IKQI-NMWQGVGKAMYAPPIEGIISCSSNITGLLLTRDGG-YESNETDEIFRPGGGDMRD  466
16  IKQIINMWQGVGKAMYAPPIEGEIRCSSNITGLLLTRDGG-YESNKTDEIFRPGGGDMRD  467
17  IKQIINMWQGVGKAMYAPPIEGQIRCSSNITGLLLTRDGG-YESNETDEIFRPGGGDMRD  467
18  IKQIINMWQGVGKAMYAPPIEGQIRCSSNITGLLLTRDGG-YESNETDEIFRPGGGDMRD  467
19  IKQIINMWQGVGKAMYAPPIEGIIRCSSNITGLLLTRDGG-YESNETDEIFRPGGGDMRD  451
20  IKQIINMWQGVGKAMYAPPIEGEIRCSSNITGLLLTRDGG-YESNKTDEIFRPGGGDMRD  467
21  IKQIINMWQGVGKAMYAPPIEGQIRCSSNITGLLLTRDGG-YESNETDEIFRPGGGDMRD  467
22  IKQIINMWQGVGKAMYAPPIEGEIRCSSNITGLLLTRDGG-YESNKTDEIFRPGGGDMRD  467
23  IKQIINMWQGVGKAMYAPPIEGEIRCSSNITGLLLTRDGG-YESNKTDEIFRPGGGDMRD  467
24  IKQIINMWQGVGKAMYAPPIEGIIRCSSNITGLLLTRDGG-YESNETDEIFRPGGGDMRD  467
25  IKQIINMWQGVGKAMYAPPIEGEIRCSSNITGLLLTRDGG-YESNKTDEIFRPGGGDMRD  467
26  IKQIINMWQGVGKAMYAPPIEGEIRCSSNITGLLLTRDGG-YESNETDEIFRPGGGDMRD  467
27  IKQIINMWQGVGKAMYAPPIEGQIRCSSNITGLLLTRDGG-YESNKTDEIFRPGGGDMRD  467
28  IKQIINMWQGVGKAMYAPPIEGEIRCSSNITGLLLTRDGG-YESNKTDEIFRPGGGDMRD  467
29  IKQIINMWQGVGKAMYAPPIEGIIRCSSNITGLLLTRDGG-YESNETDEIFRPGGGDMRD  441
30  IKQIINMWQGVGKAMYAPPIEGEIRCSSNITGLLLTRDGG-YESNKTDEIFRPGGGDMRD  467
31  IKQIINMWQGVGKAMYAPPIEGQIRCSSNITGLLLTRDGG-YESNKTDEIFRPGGGDMRD  467
32  IKQIINMWQGVGKAMYAPPIEGIIRCSSNITGLLLTRDGG-YESNETDEIFRPGGGDRRD  467
33  IKQIINMWQGVGKAMYAPPIEGIIRCSSNITGLLLTRDGG-YESNETDEIFRPGGGDMRD  467
34  IKQIINMWQGVGKAMYAPPIEGEIRCSSNITGLLLTRDGG-YESNETDEIFRPGGGDMRD  444
35  IKQIINMWQGVGKAMYAPPIEGEIRCSSNITGLLLTRDGG-YESNKTDEIFRPGGGDMRD  467
```

	NWRSELYKYKVVKIEPLGVAPTKAKRRVVQREKRAFG-LGAVFLGFLGAAGSTMGAASIT	
1	NWRSELYKYKVVKIEPLGVAPTKAKRRVVQREKRAFG-LGAVFLGFLGAAGSTMGAASIT	526
2	NWRSELYKYKVVKIEPLGVAPTKAKRRVVQREKRAFG-LGAVFLGFLGAAGSTMGAASIT	526
3	NWRSELYKYKVVKIEPLGVAPTKAKRRVVQREKRAFG-LGAVFLGFLGAAGSTMGAASIT	526
4	NWRSELYKYKVVKIEPLGVAPTKAKRRVVQREKRAFG-LGAVFLGFLGAAGSTMGAASIT	526
5	NWRSELYKYKVVKIEPLGVAPTKAKRRVVQREKRAFG-LGAVFLGFLGAAGSTMGAASIT	526
6	NWRSELYKYKVVKIEPLGVAPTKAKRRVVQREKRAFG-LGAVFLGFLGAAGSTMGAASIT	526
7	NWRSELYKYKVVKIEPLGVAPTKAKRRVVQREKRAFG-LGAVFLGFLGAAGSTMGAASIT	526
8	NWRSELYKYKVVKIEPLGVAPTKAKRRVVQREKRAFG-LGAVFLGFLGAAGSTMGAASIT	526
9	NWRSELYKYKVVKIEPLGVAPTKAKRRVVQREKRAFG-LGAVFLGFLGAAGSTMGAASIT	526
10	NWRSELYKYKVVKIEPLGVAPTKAKRRVVQKEKRAFG-LGAVFLGFLGAAGSTMGAASIT	526
11	NWRSELYKYKVVKIEPLGVAPTKAKRRVVQREKRAFG-LGAVFLGFLGAAGSTMGAASIT	526
12	NWRSELYKYKVVKIEPLGVAPTKAKRRVVQREKRAFG-LGAVFLGFLGAAGSTMGAASIT	526
13	NWRSELYKYKVVKIEPLGVAPTKAKRRVVQREKRAFG-LGAVFLGFLGAAGSTMGAASIT	526
14	NWRSELYKYKVVKIKPLGVAPTKAKRRVVQREKRAFG-LGAVFLGFLGAAGSTMGAASIT	526
15	NWRSELYKYKVVKIEPLGVAPTRAKRRVVQREKRAFG-LGAVFLGFLGAAGSTMGAASIT	525
16	NWRSELYKYKVVKIEPLGVAPTKAKRRVVQREKRAFG-LGAVFLGFLGAAGSTMGAASIT	526
17	NWRSELYKYKVVKIEPLGVAPTKAKRRVVQREKRAFG-LGAVFLGFLGAAGSTMGAASIT	526
18	NWRSELYKYKVVKIEPLGVAPTKAKRRVVQREKRAFG-LGAVFLGFLGAAGSTMGAASIT	526
19	NWRSELYKYKVVKIEPLGVAPTKAKRRVVQREKRAFG-LGAVFLGFLGAAGSTMGAASIT	510
20	NWRSELYKYKVVKIEPLGVAPTKAKRRVVQREKRAFG-LGAVFLGFLGAAGSTMGAASIT	526
21	NWRSELYKYKVVKIEPLGVAPTKAKRRVVQREKRAFG-LGAVFLGFLGAAGSTMGAASIT	526
22	NWRSELYKYKVVKIEPLGVAPTKAKRRVVQREKRAFG-LGAVFLGFLGAAGSTMGAASIT	526
23	NWRSELYKYKVVKIEPLGVAPTKAKRRVVQREKRAFG-LGAVFLGFLGAAGSTMGAASIT	526
24	NWRSELYKYKVVKIEPLGVAPTKAKRRVVQREKRAFG-LGAVFLGFLGAAGSTMGAASIT	526
25	NWRSELYKYKVVKIEPLGVAPTKAKRRVVQREKRAFG-LGAVFLGFLGAAGSTMGAASIT	526
26	NWRSELYKYKVVKIEPLGVAPTKAKRRVVQREKRAFG-LGAVFLGFLGAAGSTMGAASIT	526
27	NWRSELYKYKVVKIEPLGVAPTKAKRRVVQREKRAFG-LGAVFLGFLGAAGSTMGAASIT	526
28	NWRSELYKYKVVKIEPLGVAPTKAKRRVVQREKRAFG-LGAVFLGFLGAAGSTMGAASIT	526
29	NWRSELYKYKVVKIEPLGVAPTKAKRRVVQREKRAFG-LGAVFLGFLGAAGSTMGAASIT	500
30	NWRSELYKYKVVKIEPLGVAPTKAKRRVVQREKRAFG-LGAVFLGFLGAAGSTMGAASIT	526
31	NWRSELYKYKVVKIEPLGVAPTKAKRRVVQREKRAFG-LGAVFLGFLGAAGSTMGAASIT	526
32	KWRSELYKHKVVKIEPLGVAPTKAKRRVVQREKRAFG-LGAVFLGFLGAAGSTMGAASIT	526
33	NWRSELYKYKVVKIEPLGVAPTKAKRRVVQREKRAFG-LGAVFLGFLGAAGSTMGAASIT	526
34	NWRSELYKYKVVKIEPLGVAPTKAKRRVVQREKRAFG-LGAVFLGFLGAAGSTMGAASIT	503
35	NWRSELYKYKVVKIEPLGVAPTKAKRRVVQREKRAFG-LGAVFLGFLGAAGSTMGAASIT	526

LTVQARQLLSGIVQQQNNLLRAIEAQQHLLQLTVWGIKQLQARVLAVERYLKDQQLLGIW

1	LTVQARQLLSGIVQQQNNLLRAIEAQQHLLQLTVWGIKQLQARVLAVERYLKDQQLLGIW	586
2	LTVQARQLLSGIVQQQNNLLRAIEAQQHLLQLTVWGIKQLQARVLAVERYLKDQQLLGIW	586
3	LTVQARQLLSGIVQQQNNLLRAIEAQQHLLQLTVWGIKQLQARVLAVERYLKDQQLLGIW	586
4	LTVQARQLLSGIVQQQNNLLRAIEAQQHLLQLTVWGIKQLQARVLAVERYLKDQQLLGIW	586
5	LTVQARQLLSGIVQQQNNLLRAIEAQQHLLQLTVWGIKQLQARVLAVERYLKDQQLLGIW	586
6	LTVQARQLLSGIVQQQNNLLRAIEAQQHLLQLTVWGIKQLQARVLAVERYLKDQQLLGIW	586
7	LTVQARQLLSGIVQQQNNLLRAIEAQQHLLQLTVWGIKQLQARILAVERYLKDQQLLGIW	586
8	LTVQARQLLSGIVQQQNNLLRAIEAQQHLLQLTVWGIKQLQARVLAVERYLKDQQLLGIW	586
9	LTVQARQLLSGIVQQQNNLLRAIEAQQHLLQLTVWGIKQLQARVLAVERYLKDQQLLGIW	586
10	LTVQARQLLSGIVQQQNNLLRAIEAQQHLLQLTVWGIKQLQARVLAVERYLKDQQLLGIW	586
11	LTVQARQLLSGIVQQQNNLLRAIEAQQHLLQLTVWGIKQLQARVLAVERYLKDQQLLGIW	586
12	LTVQARQLLSGIVQQQNNLLRAIEAQQHLLQLTVWGIKQLQARVLAVERYLKDQQLLGIW	586
13	LTVQARQLLSGIVQQQNNLLRAIEAQQHLLQLTVWGIKQLQARVLAVERYLKDQQLLGIW	586
14	LTVQARQLLSGIVQQQNNLLRAIEAQQHLLQLTVWGIKQLQARVLAVERYLKDQQLLGIW	586
15	LTVQARQLLSGIVQQQNNLLRAIEAQQHLLQLTVWGIKQLQARVLAVERYLKDQQLLGIW	585
16	LTVQARQLLSGIVQQQNNLLRAIEAQQHLLQLTVWGIKQLQARVLAVERYLKDQQLLGIW	586
17	LTVQARQLLSGIVQQQNNLLRAIEAQQHLLQLTVWGIKQLQARVLAVERYLKDQQLLGIW	586
18	LTVQARQLLSGIVQQQNNLLRAIEAQQHLLQLTVWGIKQLQARVLAVERYLKDQQLLGIW	586
19	LTVQARQLLSGIVQQQNNLLRAIEAQQHLLQLTVWGIKQLQARVLAVERYLKDQQLLGIW	570
20	LTVQARQLLSGIVQQQNNLLRAIEAQQHLLQLTVWGIKQLQARVLAVERYLKDQQLLGIW	586
21	LTVQARQLLSGIVQQQNNLLRAIEAQQHLLQLTVWGIKQLQARVLAVERYLKDQQLLGIW	586
22	LTVQARQLLSGIVQQQNNLLRAIEAQQHLLQLTVWGIKQLQARVLAVERYLKDQQLLGIW	586
23	LTVQARQLLSGIVQQQNNLLRAIEAQQHLLQLTVWGIKQLQARVLAVERYLKDQQLLGIW	586
24	LTVQARQLLSGIVQQQNNLLRAIEAQQHLLQLTVWGIKQLQARVLAVERYLKDQQLLGIW	586
25	LTVQARQLLSGIVQQQNNLLRAIEAQQHLLQLTVWGIKQLQARVLAVERYLKDQQLLGIW	586
26	LTVQARQLLSGIVQQQNNLLRAIEAQQHLLQLTVWGIKQLQARVLAVERYLKDQQLLGIW	586
27	LTVQARQLLSGIVQQQNNLLRAIEAQQHLLQLTVWGIKQLQARVLAVERYLKDQQLLGIW	586
28	LTVQARQLLSGIVQQQNNLLRAIEAQQHLLQLTVWGIKQLQARVLAVERYLKDQQLLGIW	586
29	LTVQARQLLSGIVQQQNNLLRAIEAQQHLLQLTVWGIKQLQARVLAVERYLKDQQLLGIW	560
30	LTVQARQLLSGIVQQQNNLLRAIEAQQHLLQLTVWGIKQLQARVLAVERYLKDQQLLGIW	586
31	LTVQARQLLSGIVQQQNNLLRAIEAQQHLLQLTVWGIKQLQARVLAVERYLKDQQLLGIW	586
32	LTVQARQLLSGIVQQQNNLLRAIEAQQHLLQLTVWGIKQLQARVLAVERYLKDQQLLGIW	586
33	LTVQARQLLSGIVQQQNNLLRAIEAQQHLLQLTVWGIKQLQARVLAVERYLKDQQLLGIW	586
34	LTVQARQLLSGIVQQQNNLLRAIEAQQHLLQLTVWGIKQLQARVLAVERYLKDQQLLGIW	563
35	LTVQARQLLSGIVQQQNNLLRAIEAQQHLLQLTVWGIKQLQARVLAVERYLKDQQLLGIW	586

	GCSGKLICTTTVPWNTSWSNKSLEQIWDNMTWMEWEREIDNYT----GYIYQLIEESQNQ	
1	GCSGKLICTTTVPWNTSWSNKSLEQIWDNMTWMEWEREIDNYT----GYIYQLIEESQNQ	642
2	GCSGKLICTTTVPWNTSWSNKSLEQIWDNMTWMEWEREIDNYT----GYIYQLIEESQNQ	642
3	GCSGKLICTTTVPWNTSWSNKSLEQIWDNMTWMEWEREIDNYT----GYIYQLIEESQNQ	642
4	GCSGKLICTTTVPWNTSWSNKSLEQIWDNMTWMEWEREIDNYTGYIYQLIYQLIEESQNQ	646
5	GCSGKLICTTTVPWNTSWSNKSLEQIWDNMTWMEWEREIDNYT----GYIYQLIEESQNQ	642
6	GCSGKLICTTTVPWNTSWSNKSLEQIWDNMTWMEWEREIDNYT----GYIYQLIEESQNQ	642
7	GCSGKLICTTTVPWNTSWSNKSLEQIWDNMTWMEWEREIDNYT----GYIYQLIEESQNQ	642
8	GCSGKLICTTTVPWNTSWSNKSLEQIWDNMTWMEWEREIDNYT----GYIYQLIEESQNQ	642
9	GCSGKLICPTTVPWNTSWSNKSLEQIWDNMTWMEWEREIDNYT----GYIYQLIEESQNQ	642
10	GCSGKLICTTTVPWNTSWSNKSLEQIWDNMTWMEWEREIDNYT----GYIYQLIEESQNQ	642
11	GCSGKLICTTTVPWNTSWSNKSLEQIWDNMTWMEWEREIDNYT----GYIYQLIEESQNQ	642
12	GCSGKLICTTTVPWNTSWSNKSLEQIWDNMTWMEWEREIDNYT----GYIYQLIEESQNQ	642
13	GCSGKLICTTTVPWNTSWSNKSLEQIWDNMTWMEWEREIDNYT----GYIYQLIEESQNQ	642
14	GCSGKLICTTTVPWNTSWSNKSLEQIWDNMTWMEWEREIDNYT----GYIYQLIEESQNQ	642
15	GCSGKLICTTTVPWNTSWSNKSLEQIWDNMTWMEWEREIDNYT----GYIYQLIEESQNQ	641
16	GCSGKLICTTTVPWNTSWSNKSLEQIWDNMTWMEWEREIDNYT----GYIYQLIEESQNQ	642
17	GCSGKLICTTTVPWNTSWSNKSLEQIWDNMTWMEWEREIDNYT----GYIYQLIEESQNQ	642
18	GCSGKLICTTTVPWNTSWSNKSLEQIWDNMTWMEWEREIDNYT----GYIYQLIEESQNQ	642
19	GCSGKLICTTTVPWNTSWSNKSLEQIWDNMTWMEWEREIDNYT----GYIYQLIEESQNQ	626
20	GCSGKLICTTTVPWNTSWSNKSLEQIWDNMTWMEWEREIDNYT----GYIYQLIEESQNQ	642
21	GCSGKLICTTTVPWNTSWSNKSLEQIWDNMTWMEWEREIDNYT----GYIYQLIEESQNQ	642
22	GCSGKLICTTTVPWNTSWSNKSLEQIWDNMTWMEWEREIDNYT----GYIYQLIEESQNQ	642
23	GCSGKLICTTTVPWNTSWSNKSLEQIWDNMTWMEWEREIDNYT----GYIYQLIEESQNQ	642
24	GCSGKLICTTTVPWNTSWSNKSLEQIWDNMTWMEWEREIDNYT----GYIYQLIEESQNQ	642
25	GCSGKLICTTTVPWNTSWSNKSLEQIWDNMTWMEWEREIDNYT----GYIYQLIEESQNQ	642
26	GCSGKLICPTTVPWNTSWSNKSLEQIWDNMTWMEWEREIDNYT----GYIYQLIEESQNQ	642
27	GCSGKLICTTTVPWNTSWSNKSLEQIWDNMTWMEWEREIDNYT----GYIYQLIEESQNQ	642
28	GCSGKLICTTTVPWNTSWSNKSLEQIWDNMTWMEWEREIDNYT----GYIYQLIEESQNQ	642
29	GCSGKLICTTTVPWNTSWSNKSLEQIWDNMTWMEWEREIDNYT----GYIYQLIEESQNQ	616
30	GCSGKLICTTTVPWNTSWSNKSLEQIWDNMTWMEWEREIDNYT----GYIYQLIEESQNQ	642
31	GCSGKLICTTTVPWNTSWSNKSLEQIWDNMTWMEWEREIDNYT----GYIYQLIEESQNQ	642
32	GCSGKLICTTTVPWNTSWSNKSLEQIWDNMTWMEWEREIDNYT----GYIYQLIEESQNQ	642
33	GCSGKLICTTTVPWNTSWSNKSLEQIWDNMTWMEWEREIDNYT----GYIYQLIEESQNQ	642
34	GCSGKLICPTTVPWNTSWSNKSLEQIWDNMTWMEWEREIDNYT----GYIYQLIEESQNQ	619
35	GCSGKLICTTTVPWNTSWSNKSLEQIWDNMTWMEWEREIDNYT----GYIYQLIEESQNQ	642

	**QEKNEQELLALDKWASLWNWFDITNWLWYIKIFIMIVGGLIGLRIVFTVLSIVNRVRQGY**	
1	QEKNEQELLALDKWASLWNWFDITNWLWYIKIFIMIVGGLIGLRIVFTVLSIVNRVRQGY	702
2	QEKNEQELLALDKWASLWNWFDITNWLWYIKIFIMIVGGLIGLRIVFTVLSIVNRVRQGY	702
3	QEKNEQELLALDKWASLWNWFDITNWLWYIKIFIMIVGGLIGLRIVFTVLSIVNRVRQGY	702
4	QEKNEQELLALDKWASLWNWFDITNWLWYIKIFIMIVGGLIGLRIVFTVLSIVNRVRQGY	706
5	QEKNEQELLALDKWASLWNWFDITNWLWYIKIFIMIVGGLIGLRIVFTVLSIVNRVRQGY	702
6	QEKNEQELLALDKWASLWNWFDITNWLWYIKIFIMIVGGLIGLRIVFTVLSIVNRVRQGY	702
7	QEKNEQELLALDKWASLWNWFDITNWLWYIKIFIMIVGGLIGLRIVFTVLSIVNRVRQGY	702
8	QEKNEQELLALDKWASLWNWFDITNWLWYIKIFIMIVGGLIGLRIVFTVLSIVNRVRQGY	702
9	QEKNEQELLALDKWASLWNWFDITNWLWYIKIFIMIVGGLIGLRIVFTVLSIVNRVRQGY	702
10	QEKNEQELLALDKWASLWNWFDITNWLWYIKIFIMIVGGLIGLRIVFTVLSIVNRVRQGY	702
11	QEKNEQELLALDKWASLWNWFDITNWLWYIKIFIMIVGGLIGLRIVFTVLSIVNRVRQGY	702
12	QEKNEQELLALDKWASLWNWFDITNWLWYIKIFIMIVGGLIGLRIVFTVLSIVNRVRQGY	702
13	QEKNEQELLALDKWASLWNWFDITNWLWYIKIFIMIVGGLIGLRIVFTVLSIVNRVRQGY	702
14	QEKNEQELLALDKWASLWNWFDITNWLWYIKIFIMIVGGLIGLRIVFTVLSIVNRVRQGY	702
15	QEKNEQELLALDKWASLWNWFDITNWLWYIKIFIMIVGGVIGLRIVFTVLSIVNRVRQGY	701
16	QEKNEQELLALDKWASLWNWFDITNWLWYIKIFIMIVGGLIGLRIVFTVLSIVNRVRQGY	702
17	QEKNEQELLALDKWASLWNWFDITNWLWYIKIFIMIVGGLIGLRIVFTVLSIVNRVRQGY	702
18	QEKNEQELLALDKWASLWNWFDITNWLWYIKIFIMIVGGLIGLRIVFTVLSIVNRVRQGY	702
19	QEKNEQELLALDKWASLWNWFDITNWLWYIKIFIMIVGGLIGLRIVFTVLSIVNRVRQGY	686
20	QEKNEQELLALDKWASLWNWFDITNWLWYIKIFIMIVGGLIGLRIVFTVLSIVNRVRQGY	702
21	QEKNEQELLALDKWASLWNWFDITNWLWYIKIFIMIVGGLIGLRIVFTVLSIVNRVRQGY	702
22	QEKNEQELLALDKWASLWNWFDITNWLWYIKIFIMIVGGLIGLRIVFTVLSIVNRVRQGY	702
23	QEKNEQELLALDKWASLWNWFDITNWLWYIKIFIMIVGGLIGLRIVFTVLSIVNRVRQGY	702
24	QEKNEQELLALDKWASLWNWFDITNWLWYIKIFIMIVGGLIGLRIVFTVLSIVNRVRQGY	702
25	QEKNEQELLALDKWASLWNWFDITNWLWYIKIFIMIVGGLIGLRIVFTVLSIVNRVRQGY	702
26	QEKNEQELLALDKWASLWNWFDITNWLWYIKIFIMIVGGLIGLRIVFTVLSIVNRVRQGY	702
27	QEKNEQELLALDKWASLWNWFDITNWLWYIKIFIMIVGGLIGLRIVFTVLSIVNRVRQGY	702
28	QEKNEQELLALDKWAGLWNWFDITNWLWYIKIFIMIVGGLIGLRIVFTVLSIVNRVRQGY	702
29	QEKNEQELLALDKWASLWNWFDITNWLWYIKIFIMIVGGLIGLRIVFTVLSIVNRVRQGY	676
30	QEKNEQELLALDKWASLWNWFDITNWLWYIKIFIMIVGGLIGLRIVFTVLSIVNRVRQGY	702
31	QEKNEQELLALDKWASLWNWFDITNWLWYIKIFIMIVGGLIGLRIVFTVLSIVNRVRQGY	702
32	QEKNEQELLALDKWASLWNWFDITNWLWYIKIFIMIVGGLIGLRIVFTVLSIVNRVRQGY	702
33	QEKNEQELLALDKWASLWNWFDITNWLWYIKIFIMIVGGLIGLRIVFTVLSIVNRVRQGY	702
34	QEKNEQELLALDKWASLWNWFDITNWLWYIKIFIMIVGGLIGLRIVFTVLSIVNRVRQGY	679
35	QEKNEQELLALDKWASLWNWFDITNWLWYIKIFIMIVGGLIGLRIVFTVLSIVNRVRQGY	702

	**SPLSFQTHLPAQRGPDRPEGIGEEGGERDRDRSDPLVNGFLTLIWSDLRSLCLFSYHRLR**	
1	SPLSFQTHLPAQRGPDRPEGIGEEGGERDRDRSDPLVNGFLALIWSDLRSLCLFSYHRLR	762
2	SPLSFQTHLPAQRGPDRPEGIGEEGGERDRDRSDPLVNGFLTLIWSDLRSLCLFSYHRLR	762
3	SPLSFQTHLPAQRGPDRPEGIGEEGGERDRDRSDPLVNGFLALIWSDLRSLCLFSYHRLR	762
4	SPLSFQTHLPAQRGPDRPEGIGEEGGERDRDRSDPLVNGFLTLIWSDLRSLCLFSYHRLR	766
5	SPLSFQTHLPAQRGPDRPEGIGEEGGERDRDRSDPLVNGFLTLIWSDLRSLCLFSYHRLR	762
6	SPLSFQTHLPAQRGPDRPEGIGEEGGERDRDRSDPLVNGFLTLIWSDLRSLCLFSYHRLR	762
7	SPLSFQTHLPAQRGPDRPEGIGEEGGERDRDRSDPLVNGFLTLIWSDLRSLCLFSYHRLR	762
8	SPLSFQTHLPAQRGPDRPEGIGEEGGERDRDRSDPLVNGFLTLIWSDLRSLCLFSYHRLR	762
9	SPLSFQTHLPAQRGPDRPEGIGEEGGERDRDRSDPLVNGFLTLIWSDLRSLCLFSYHRLR	762
10	SPLSFQTHLPAQRGPDRPEGIGEEGGERDRDRSDPLVNGFLTLIWSDLRSLCLFSYHRLR	762
11	SPLSFQTHLPAQRGPDRPEGIGEEGGERDRDRSDPLVNGFLTLIWSDLRSLCLFSYHRLR	762
12	SPLSFQTHLPAQRGPDRPEGIGEEGGERDRDRSDPLVNGFLTLIWSDLRSLCLFSYHRLR	762
13	SPLSFQTHLPAQRGPDRPEGIGEEGGERDRDRSDPLVNGFLALIWSDLRSLCLFSYHRLR	762
14	SPLSFQTHLPAQRGPDRPEGIGEEGGERDRDRSDPLVNGFLTLIWSDLRSLCLFSYHRLR	762
15	SPLSFQTHLPAQRGPDRPEGIGEEGGERDRDRSDPLVNGFLTLIWSDLRSLCLFSYHRLR	761
16	SPLSFQTHLPAQRGPDRPEGIGEEGGERDRDRSDPLVNGFLTLIWSDLRSLCLFSYHRLR	762
17	SPLSFQTHLPAQRGPDRPEGIGEEGGERDRDRSDPLVNGFLTLIWSDLRSLCLFSYHRLR	762
18	SPLSFQTHLPAQRGPDRPEGIGEEGGERDRDRSDPLVNGFLTLIWSDLRSLCLFSYHRLR	762
19	SPLSFQTHLPAQRGPDRPEGIGEEGGERDRDRSDPLVNGFLTLIWSDLRSLCLFSYHRLR	746
20	SPLSFQTHLPAQRGPDRPEGIGEEGGERDRDRSDPLVNGFLTLIWSDLRSLCLFSYHRLR	762
21	SPLSFQTHLPAQRGPDRPEGIGEEGGERDRDRSDPLVNGFLTLIWSDLRSLCLFSYHRLR	762
22	SPLSFQTHLPAQRGPDRPEGIGEEGGERDRDRSDPLVNGFLTLIWSDLRSLCLFSYHRLR	762
23	SPLSFQTHLPAQRGPDRPEGIGEEGGERDRDRSDPLVNGFLTLIWSDLRSLCLFSYHRLR	762
24	SPLSFQTHLPAQRGPDRPEGIGEEGGERDRDRSDPLVNGFLTLIWSDLRSLCLFSYHRLR	762
25	SPLSFQTHLPAQRGPDRPEGIGEEGGERDRDRSDPLVNGFLTLIWSDLRSLCLFSYHRLR	762
26	SPLSFQTHLPAQRGPDRPEGIGEEGGERDRDRSDPLVNGFLTLIWSDLRSLCLFSYHRLR	762
27	SPLSFQTHLPAQKGPDRPEGIGEEGGERDRDRSDPLVNGFLTLIWSDLRSLCLFSYHRLR	762
28	SPLSFQTHLPAQRGPDRPEGIGEEGGERDRDRSDPLVNGFLTLIWSDLRSLCLFSYHRLR	762
29	SPLSFQTHLPAQRGPDRPEGIGEEGGERDRDRSDPLVNGFLTLIWSDLRSLCLFSYHRLR	736
30	SPLSFQTHLPAQRGPDRPEGIGEEGGERDRDRSDPLVNGFLTLIWSDLRSLCLFSYHRLR	762
31	SPLSFQTHLPAQRGPDRPEGIGEEGGERDRDRSDPLVNGFLTLIWSDLRSLCLFSYHRLR	762
32	SPLSFQTHLPAQRGPDRPEGIGEEGGERDRDRSDPLVNGFLTLIWSDLRSLCLFSYHRLR	762
33	SPLSFQTHLPAQRGPDRPEGIGEEGGERDRDRSDPLVNGFLTLIWSDLRSLCLFSYHRLR	762
34	SPLSFQTHLPAQRGPDRPEGIGEEGGERDRDRSDPLVNGFLTLIWSDLRSLCLFSYHRLR	739
35	SPLSFQTHLPAQRGPDRPEGIGEEGGERDRDRSDPLVNGFLTLIWSDLRSLCLFSYHRLR	762

	Sequence	
	**DLLLIVTRIVELLGR-------RGWEVLKYWWNLLQYWSQELKNSAVSLLNATATAVAEG**	
1	DLLLIVTRIVELLGR-------RGWEVLKYWWNLLQYWSQELKNSAVSLLNATAIAVAEG	815
2	DLLLIVTRIVELLGR-------RGWEVLKYWWNLLQYWSQELKNSAVSLLNATATAVAEG	815
3	DLLLIVTRIVELLGR-------RGWEVLKYWWNLLQYWSQELKNSAVSLLNATATAVAEG	815
4	DLLLIVTRIVELMGR-------RGWEVLKYWWNLLQYWSQELKNSAVSLLNATATAVAEG	819
5	DLLLIVTRIVELLGR-------RGWEVLKYWWNLLQYWSQELKNSAVSLLNATAIAVAEG	815
6	DLLLIVTRIVELLGR-------RGWEVLKYWWNLLQYWSQELKNSAVSLLNATAIAVAEG	815
7	DLLLIVTRIVELLGR-------RGWEVLKYWWNLLQYWSQELKNSAVSLLNATATAVAEG	815
8	DLLLIVTRIVELLGR-------RGWEVLKYWWNLLRYWSQELKNSAVSLLNATATAVAEG	815
9	DLLLIVTRIVELLGR-------RGWEVLKYWWNLLQYWSQELKNSAVSLLNATAIAVAEG	815
10	DLLLIVTRIVELLGR-------RGWEVLKYWWNLLQYWSQELKNSAVSLLNATATAVAEG	815
11	DLLLIVTRIVELLGR-------RGWEVLKYWWNLLQYWSQELKNSAVSLLNATATAVAEG	815
12	DLLLIVTRIVELLGR-------RGWEVLKYWWNLLQYWSQELKNSAVSLLNATATAVAEG	815
13	DLLLIVTRIVELLGR-------RGWEVLKYWWNLLQYWSQELKNSAVSLLNATAIAVAEG	815
14	DLLLIVTRIVELLGR-------RGWEILKYWWNLLQYWSQELKNSAVSLLNATATAVAEG	815
15	DLLLIVTRIVELLGR-------RGWEVLKYWWNLLQYWSQELKNSAVSLLNATATAVAEG	814
16	DLLLIVTRIVELLGR-------RGWEVLKYWWNLLQYWSQELKNSAVSLLNATATAVAEG	815
17	DLLLIVTRIVELLGR-------RGWEVLKYWWNLLQYWSQELKNSAVSLLNATATAVAEG	815
18	DLLLIVTRIVELLGR-------RGWEVLKYWWNLLQYWSQELKNSAVSLLNATAIAVAEG	815
19	DLLLIVTRIVELLGR-------RGWEVLKYWWNLLQYWSQELKNSAVSLLNATAIAVAEG	799
20	DLLLIVTRIVELLGR-------RGWEVLKYWWNLLQYWSQELKNSAVSLLNATATAVAEG	815
21	DLLLIVTRIVELLGR-------RGWEVLKYWWNLLQYWSQELKNSAVSLLNATATAVAEG	815
22	DLLLIVTRIVELLGR-------RGWEVLKYWWNLLQYWSQELKNSAVSLLNATATAVAEG	815
23	DLLLIVTRIVELLGR-------RGWEVLKYWWNLLQYWSQELKNSAVSLLNATATAVAEG	815
24	DLLLIVTRIVELLGR-------RGWEVLKYWWNLLQYWSQELKNSAVSLLNATATAVAEG	815
25	DLLLIVTRIVELLGR-------RGWEVLKYWWNLLQYWSQELKNSAVSLLNATATAVAEG	815
26	DLLLIVTRIVELLGR-------RGWEVLKYWWNLLQYWSQELKNSAVSLLNATAIAVAEG	815
27	DLLLIVTRIVELLGR-------RGWEVLKYWWNLLQYWSQELKNSAVSLLNATATAVAEG	815
28	DLLLIVTRIVELLGR-------RGWEVLKYWWNLLQYWSQELKNSAVSLLNATATAVAEG	815
29	DLLLIVTRIVELLGR-------RGWEVLKYWWNLLQYWSQELKNSAVSLLNATATAVAEG	789
30	DLLLIVTRIVELLGR-------RGWEVLKYWWNLLQYWSQELKNSAVSLLNATATAVAEG	815
31	DLLLIVTRIVELLGR-------RGWEVLKYWWNLLQYWSQELKNSAVSLLNATATAVAEG	815
32	DLLLIVTRIVELLGR-------RGWEVLKYWWNLLQYWSQELKNSAVSLLNATATAVAEG	815
33	DLLLIVTRIVELLGR-------RGWEVLKYWWNLLQYWSQELKNSAVSLLNATATAVAEG	815
34	DLLLIVTRIVELLGR-------RGWEVLKYWWNLLQYWSQELKNSAVSLLNATAIAVAEG	792
35	DLLLIVTRIVELLGR-------RGWEVLKYWWNLLQYWSQELKNSAVSLLNATATAVAEG	815

	**TDRVIEVVQRACRAILHIPRRIRQGLERALL**	
1	TDRVIEVVQRACRAILHIPRRIRQGLERALL	846
2	TDRVIEVVQRACRAILHIPRRIRQGLERALL	846
3	TDRVIEVVQRACRAILHIPRRIRQGLERALL	846
4	TDRVIEVVQRACRAILHIPRRIRQGLERALL	850
5	TDRVIEVVQRACRAIRHIPRRIRQGLERALL	846
6	TDRVIEVVQGACRAILHIPRRIRQGLERALL	846
7	TDRVIEVVQRACRAILHIPRRIRQGLERALL	846
8	TDRVIEVVQRACRAILHIPRRIRQGLERALL	846
9	TDRVIEVVQRACRAILHIPRRIRQGLERALL	846
10	TDRVIEVVQRACRAILHIPRRIRQGLERALL	846
11	TDRVVEVVQRACRAILHIPRRIRQGLERALL	846
12	TDRVIEVVQRACRAILHIPRRIRQGLERALL	846
13	TDRVIEVVQRTCRAIRHIPRRIRQGLERALL	846
14	TDRVIEVVQRACRAILHIPRRIRQGLERALL	846
15	TDRVIEVVQRACRAILHIPRRIRQGLERALL	845
16	TDRVIEVVQRACRAILHIPRRIRQGLERALL	846
17	TDRVIEVVQRACRAILHIPRRIRQGLERALL	846
18	TDRVIEVVQRACRAIRHIPRRIRQGLERALL	846
19	TDRVIEVVQRACRAIRHIPRRIRQGLERALL	830
20	TDRVIEVVQRACRAILHIPRRIRQGLERALL	846
21	TDRVIEVVQRACRAILHIPRRIRQGLERALL	846
22	TDRVIEVVQRACRAILHIPRRIRQGLERALL	846
23	TDRVIEVVQRACRAILHIPRRIRQGLERALL	846
24	TDRVIEVVQRACRAILHIPRRIRQGLERALL	846
25	TDRVIEVVQRACRAILHIPRRIRQGLERALL	846
26	TDRVIEVVQRACRAILHIPRRIRQGLERALL	846
27	TDRVIEVVQRACRAILHIPRRIRQGLERALL	846
28	TDRVIEVVQRACRAILHIPRRIRQGLERALL	846
29	TDRVIEVVQRACRAILHIPRRIRQGLERALL	820
30	TDRVIEVVQRACRAILHIPRRIRQGLERALL	846
31	TDRVIEVVQRACRAILHIPRRIRQGLERALL	846
32	TDRVIEVVQRACRAILHIPRRIRQGLERALL	846
33	TDRVIEVVQRACRAILHIPRRIRQGLERALL	846
34	TDRVIEVVQRACRAILHIPRRIRQGLERALL	823
35	TDRVIEVVQRACRAILHIPRRIRQGLERALL	846

Consensus

1. B.US.2006.700010040_C9_4520.EU289193
2. B.US.2008.CH0040_3_d0666_ipe032_27_9.MG900342
3. B.US.2008.CH0040_3_d0666_ipe032_2_01.MG900343
4. B.US.2008.CH0040_3_d0666_ipe032_3_03.MG900344
5. B.US.2008.CH0040_3_d0666_ipe032_3_04.MG900345
6. B.US.2008.CH0040_3_d0666_ipe032_3_05.MG900346
7. B.US.2008.CH0040_3_d0666_ipe032_3_07.MG900347
8. B.US.2008.CH0040_3_d0666_ipe032_3_08.MG900348
9. B.US.2008.CH0040_3_d0666_ipe032_5_11.MG900349
10. B.US.2008.CH0040_3_d0666_ipe032_5_12.MG900350
11. B.US.2008.CH0040_3_d0666_ipe032_5_13.MG900351
12. B.US.2008.CH0040_3_d0666_ipe032_5_15.MG900352
13. B.US.2008.CH0040_3_d0666_ipe032_5_16.MG900353
14. B.US.2008.CH0040_3_d0666_ipe032_5_17.MG900354
15. B.US.2008.CH0040_3_d0666_ipe032_5_18.MG900355
16. B.US.2008.CH0040_3_d0666_ipe032_5_21.MG900356
17. B.US.2008.CH0040_3_d0666_ipe032_5_22.MG900357
18. B.US.2008.CH0040_3_d0666_ipe032_5_23.MG900358
19. B.US.2008.CH0040_3_d0666_ipe032_5_24.MG900359
20. B.US.2008.CH0040_3_d0666_ipe032_5_33.MG900360
21. B.US.2008.CH0040_3_d0666_ipe032_5_34.MG900361
22. B.US.2008.CH0040_3_d0666_ipe032_5_37.MG900362
23. B.US.2008.CH0040_3_d0666_ipe032_5_38.MG900363
24. B.US.2008.CH0040_3_d0666_ipe032_5_41.MG900364
25. B.US.2008.CH0040_3_d0666_ipe032_5_42.MG900365
26. B.US.2008.CH0040_3_d0666_ipe032_5_43.MG900366
27. B.US.2008.CH0040_3_d0666_ipe032_5_44.MG900367
28. B.US.2008.CH0040_3_d0666_ipe032_5_45.MG900368
29. B.US.2008.CH0040_3_d0666_ipe032_5_48.MG900369
30. B.US.2008.CH0040_3_d0666_ipe032_5_49.MG900370
31. B.US.2008.CH0040_3_d0666_ipe032_5_51.MG900371
32. B.US.2008.CH0040_3_d0666_ipe032_5_52.MG900372
33. B.US.2008.CH0040_3_d0666_ipe032_5_56.MG900373
34. B.US.2008.CH0040_3_d0666_ipe032_5_57.MG900374
35. B.US.2008.CH0040_3_d0666_ipe032_5_58.MG900375
36. B.US.2008.CH0040_3_d0666_ipe032_5_60.MG900376
37. B.US.2008.CH0040_3_d0666_ipe032_5_62.MG900377
38. B.US.2008.CH0040_3_d0666_ipe032_5_63.MG900378
39. B.US.2008.CH0040_3_d0666_ipe032_5_64.MG900379
40. B.US.2008.CH0040_3_d0666_ipe032_5_65.MG900380
41. B.US.2008.CH0040_3_d0666_ipe032_5_66.MG900381
42. B.US.2008.CH0040_3_d0666_ipe032_5_67.MG900382
43. B.US.2008.CH0040_3_d0666_ipe032_5_68.MG900383
44. B.US.2008.CH0040_3_d0666_ipe032_5_73.MG900384
45. B.US.2008.CH0040_3_d0666_ipe032_5_79.MG900385
46. B.US.2008.CH0040_3_d0666_ipe032_5_82.MG900386

MRVMGIRKNYQHLWREGILLLGILMICSAADNLWVTVYYGVPVWREATTTLFCASDAKAY

1	MRVMGIRKNYQHLWREGILLLGILMICSAADNLWVTVYYGVPVWREATTTLFCASDAKAY	60
2	MRVMGIRKNYQHLWREGILLLGILMICSAADNLWVTVYYGVPVWREATTTLFCASDAKAY	60
3	MRVMGIRKNYQHLWREGILLLGILMICSAADNLWVTVYYGVPVWREATTTLFCASDAKAY	60
4	MRVMGIRKNYQHLWREGILLLGILMICSAADNLWVTVYYGVPVWREATTTLFCASDAKAY	60
5	MRVMGIRKNYQHLWREGILLLGILMICSAADNLWVTVYYGVPVWREATTTLFCASDAKAY	60
6	MRVMGIRKNYQHLWREGILLLGILMICSAADNLWVTVYYGVPVWRKATTTLFCASDAKAY	60
7	MRVMGIRKNYQHLWREGILLLGILMICSAADNLWVTVYYGVPVWREATTTLFCASDAKAY	60
8	MRVMGIRKNYQHLWREGILLLGILMICSAADNLWVTVYYGVPVWREATTTLFCASDAKAY	60
9	MRVMGIRKNYQHLWREGILLLGILMICSAADNLWVTVYYGVPVWREATTTLFCASDAKAY	60
10	MRVMGIRKNYQHLWREGILLLGILMICSAADNLWVTVYYGVPVWREATTTLFCASDAKAY	60
11	MRVMGIRKNYQHLWREGILLLGILMICSAADNLWVTVYYGVPVWREATTTLFCASDAKAY	60
12	MRVMGIRKNYQHLWREGILLLGILMICSAADNLWVTVYYGVPVWREATTTLFCASDAKAY	60
13	MRVMGIRKNYQHLWREGILLLGILMICSAADNLWVTVYYGVPVWREATTTLFCASDAKAY	60
14	MRVMGIRKNYQHLWREGILLLGILMICSAADNLWVTVYYGVPVWREATTTLFCASDAKAY	60
15	MRVMGIRKNYQHLWREGILLLGILMICSAADNLWVTVYYGVPVWREATTTLFCASDAKAY	60
16	MRVMGIRKNYQHLWREGILLLGILMICSAADNLWVTVYYGVPVWREATTTLFCASDAKAY	60
17	MRVMGIRKNYQHLWREGILLLGILMICSAADNLWVTVYYGVPVWREATTTLFCASDAKAY	60
18	MRVMGIRKNYQHLWREGILLLGILMICSAADNLWVTVYYGVPVWREATTTLFCASDAKAY	60
19	MRVMGIRKNYQHLWREGILLLGILMICSAADNLWVTVYYGVPVWREATTTLFCASDAKAY	60
20	MRVMGIRKNYQHLWREGILLLGILMICSAADNLWVTVYYGVPVWREATTTLFCASDAKAY	60
21	MRVMGIRKNYQHLWREGILLLGILMICSAADNLWVTVYYGVPVWREATTTLFCASDAKAY	60
22	MRVMGIRKNYQHLWREGILLLGILMICSAADNLWVTVYYGVPVWREATTTLFCASDAKAY	60
23	MRVMGIRKNYQHLWREGILLLGILMICSAADNLWVTVYYGVPVWREATTTLFCASDAKAY	60
24	MRVMGIRKNYQHLWREGILLLGILMICSAADNLWVTVYYGVPVWREATTTLFCASDAKAY	60
25	MRVMGIRKNYQHLWREGILLLGILMICSAADNLWVTVYYGVPVWREATTTLFCASDAKAY	60
26	MRVMGIRKNYQHLWREGILLLGILMICSAADNLWVTVYYGVPVWREATTTLFCASDAKAY	60
27	MRVMGIRKNYQHLWREGILLLGILMICSAADNLWVTVYYGVPVWREATTTLFCASDAKAY	60
28	MRVMGIRKNYQHLWREGILLLGILMICSAADNLWVTVYYGVPVWREATTTLFCASDAKAY	60
29	MRVMGIRKNYQHLWREGILLLGILMICSAADNLWVTVYYGVPVWREATTTLFCASDAKAY	60
30	MRVMGIRKNYQHLWREGILLLGILMICSAADNLWVTVYYGVPVWREATTTLFCASDAKAY	60
31	MRVMGIRKNYQHLWREGILLLGILMICSAADNLWVTVYYGVPVWREATTTLFCASDAKAY	60
32	MRVMGIRKNYQHLWREGILLLGILMICSAADNLWVTVYYGVPVWREATTTLFCASDAKAY	60
33	MRVMGIRKNYQHLWREGILLLGILMICSAADNLWVTVYYGVPVWREATTTLFCASDAKAY	60
34	MRVMGIRKNYQHLWREGILLLGILMICSAADNLWVTVYYGVPVWREATTTLFCASDAKAY	60
35	MRVMGIRKNYQHLWREGILLLGILMICSAADNLWVTVYYGVPVWREATTTLFCASDAKAY	60
36	MRVMGIRKNYQHLWREGILLLGILMICSAADNLWVTVYYGVPVWREATTTLFCASDAKAY	60
37	MRVMGIRKNYQHLWRKGILLLGILMICSAADNLWVTVYYGVPVWREATTTLFCASDAKAY	60
38	MRVMGIRKNYQHLWREGILLLGILMICSAADNLWVTVYYGVPVWREATTTLFCASDAKAY	60
39	MRVMGIRKNYQHLWREGILLLGILMICSAADNLWVTVYYGVPVWREATTTLFCASDAKAY	60
40	MRVMGIRKNYQHLWREGILLLGILMICSAADNLWVTVYYGVPVWREATTTLFCASDAKAY	60
41	MRVMGIRKNYQHLWREGILLLGILMICSAADNLWVTVYYGVPVWREATTTLFCASDAKAY	60
42	MRVMGIRKNYQHLWREGILLLGILMICSAADNLWVTVYYGVPVWREATTTLFCASDAKAY	60
43	MRVMGIRKNYQHLWREGILLLGILMICSAADNLWVTVYYGVPVWREATTTLFCASDAKAY	60
44	MRVMGIRKNYQHLWREGILLLGILMICSAADNLWVTVYYGVPVWREATTTLFCASDAKAY	60
45	MRVMGIRKNYQHLWREGILLLGILMICSAADNLWVTVYYGVPVWREATTTLFCASDAKAY	60
46	MRVMGIRKNYQHLWREGILLLGILMICSAADNLWVTVYYGVPVWREATTTLFCASDAKAY	60

DTEAHNVWATHACVPTDPNPQEVELKNVTENFNMWENNMVEQMHEDIISLWDQSLKPCVK

1	DTEAHNVWATHACVPTDPNPQEVELKNVTENFNMWENNMVEQMHEDIISLWDQSLKPCVK	120
2	DTEAHNVWATHACVPTDPNPQEVELK----NFNMWENNMVEQMHEDIISLWDQSLKPCVK	116
3	DTEAHNVWATHACVPTDPNPQEVELKNVTENFNMWENNMVEQMHEDIISLWDQSLKPCVK	120
4	DTEAHNVWATHACVPTDPNPQEVELKNVTENFNMWENNMVEQMHEDIISLWDQSLKPCVK	120
5	DTEAHNVWATHACVPTDPNPQEVELKNVTENFNMWENNMVEQMHEDIISLWDQSLKPCVK	120
6	DTEAHNVWATHACVPTDPNPQEVELKNVTENFNMWENNMVEQMHEDIISLWDQSLKPCVK	120
7	DTEAHNVWATHACVPTDPNPQEVELKNVTENFNMWENNMVEQMHEDIISLWDQSLKPCVK	120
8	DTEAHNVWATHACVPTDPNPQEVELKNVTENFNMWENNMVEQMHEDIISLWDQSLKPCVK	120
9	DTEAHNVWATHACVPTDPNPQEVELKNVTENFNMWENNMVEQMHEDIISLWDQSLKPCVK	120
10	DTEAHNVWATHACVPTDPNPQEVELKNVTENFNMWENNMVEQMHEDIISLWDQSLKPCVK	120
11	DTEAHNVWATHACVPTDPNPQEVELKNVTENFNMWENNMVEQMHEDIISLWDQSLKPCVK	120
12	DTEAHNVWATHACVPTDPNPQEVELKNVTENFNMWENNMVEQMHEDIISLWDQSLKPCVK	120
13	DTEAHNVWATHACVPTDPNPQEVELKNVTENFNMWENNMVEQMHEDIISLWDQSLKPCVK	120
14	DTEAHNVWATHACVPTDPNPQEVELKNVTENFNMWENNMVEQMHEDIISLWDQSLKPCVK	120
15	DTEAHNVWATHACVPTDPNPQEVELKNVTENFNMWENNMVEQMHEDIISLWDQSLKPCVK	120
16	DTEAHNVWATHACVPTDPNPQEVELKNVTENFNMWENNMVEQMHEDIISLWDQSLKPCVK	120
17	DTEAHNVWATHACVPTDPNPQEVELKNVTENFNMWENNMVEQMHEDIISLWDQSLKPCVK	120
18	DTEAHNVWATHACVPTDPNPQEVELKNVTENFNMWENNMVEQMHEDIISLWDQSLKPCVK	120
19	DTEAHNVWATHACVPTDPNPQEVELKNVTENFNMWENNMVEQMHEDIISLWDQSLKPCVK	120
20	DTEAHNVWATHACVPTDPNPQEVELKNVTENFNMWENNMVEQMHEDIISLWDQSLKPCVK	120
21	DTEAHNVWATHACVPTDPNPQEVELKNVTENFNMWENNMVEQMHEDIISLWDQSLKPCVK	120
22	DTEAHNVWATHACVPTDPNPQEVELKNVTENFNMWENNMVEQMHEDIISLWDQSLKPCVK	120
23	DTEAHNVWATHACVPTDPNPQEVELKNVTENFNMWENNMVEQMHEDIISLWDQSLKPCVK	120
24	DTEAHNVWATHACVPTDPNPQEVELKNVTENFNMWENNMVEQMHEDIISLWDQSLKPCVK	120
25	DTEAHNVWATHACVPTDPNPQEVELKNVTENFNMWENNMVEQMHEDIISLWDQSLKPCVK	120
26	DTEAHNVWATHACVPTDPNPQEVELKNVTENFNMWENNMVEQMHEDIISLWDQSLKPCVK	120
27	DTEAHNVWATHACVPTDPNPQEVELKNVTENFNMWENNMVEQMHEDIISLWDQSLKPCVK	120
28	DTEAHNVWATHACVPTDPNPQEVELKNVTENFNMWENNMVEQMHEDIISLWDQSLKPCVK	120
29	DTEAHNVWATHACVPTDPNPQEVELKNVTENFNMWENNMVEQMHEDIISLWDQSLKPCVK	120
30	DTEAHNVWATHACVPTDPNPQEVELKNVTENFNMWENNMVEQMHEDIISLWDQSLKPCVK	120
31	DTEAHNVWATHACVPTDPNPQEVELKNVTENFNMWENNMVEQMHEDIISLWDQSLKPCVK	120
32	DTEAHNVWATHACVPTDPNPQEVELKNVTENFNMWENNMVEQMHEDIISLWDQSLKPCVK	120
33	DTEAHNVWATHACVPTDPNPQEVELKNVTENFNMWENNMVEQMHEDIISLWDQSLKPCVK	120
34	DTEAHNVWATHACVPTDPNPQEVELKNVTENFNMWENNMVEQMHEDIISLWDQSLKPCVK	120
35	DTEAHNVWATHACVPTDPNPQEVELKNVTENFNMWENNMVEQMHEDIISLWDQSLKPCVK	120
36	DTEAHNVWATHACVPTDPNPQEVELKNVTENFNMWENNMVEQMHEDIISLWDQSLKPCVK	120
37	DTEAHNVWATHACVPTDPNPQEVELKNVTENFNMWENNMVEQMHEDIISLWDQSLKPCVK	120
38	DTEAHNVWATHACVPTDPNPQEVELKNVTENFNMWENNMVEQMHEDIISLWDQSLKPCVK	120
39	DTEAHNVWATHACVPTDPNPQEVELKNVTENFNMWENNMVEQMHEDIISLWDQSLKPCVK	120
40	DTEAHNVWATHACVPTDPNPQEVELKNVTENFNMWENNMVEQMHEDIISLWDQSLKPCVK	120
41	DTEAHNVWATHACVPTDPNPQEVELKNVTENFNMWENNMVEQMHEDIISLWDQSLKPCVK	120
42	DTEAHNVWATHACVPTDPNPQEVELKNVTENFNMWENNMVEQMHEDIISLWDQSLKPCVK	120
43	DTEAHNVWATHACVPTDPNPQEVELKNVTENFNMWENNMVEQMHEDIISLWDQSLKPCVK	120
44	DTEAHNVWATHACVPTDPNPQEVELKNVTENFNMWKNNMVEQMHEDIISLWDQSLKPCVK	120
45	DTEAHNVWATHACVPTDPNPQEVELKNVTENFNMWENNMVEQMHEDIISLWDQSLKPCVK	120
46	DTEAHNVWATHACVPTDPNPQEVELKNVTENFNMWENNMVEQMHEDIISLWDQSLKPCVK	120

```
    LTPLCVTLNCTDLEKXTNTT---NSTEKMMEKGEVKNCSFKITTDXKDRTRKEYALFYKL
1   LTPLCVTLNCTDLGNVTNTT---NSNGEMMEKGEVKNCSFKITTDIKDRTRKEYALFYKL  177
2   LTPLCVTLNCTDLGNVTTTI---NSNGTLMEKGEVKNCSFKITTDTKDRTRKEYALFYKL  173
3   LTPLCVTLNCTDLGNVTTTT---NSNGTLMEKGEVKNCSFKITTDTKDRTRKEYALFYKL  177
4   LTPLCVTLNCTDLEKGTNTT---NSTEKMMEKGEVKNCSFRITTDIKDRTRKEYALFYKL  177
5   LTPLCVTLNCTDLENVTDTT---NSTEKMMEKGEVKNCSFKITTDLKDRTRKEYALFYKL  177
6   LTPLCVTLNCTDLEKGTNTT---NSTEKMMEKGEVKNCSFRITTDIKDRTRKEYALFYKL  177
7   LTPLCVTLNCTDLENVTNTT---NSTEKMMEKGEVKNCSFKITTDITDRTRKEYALFYKL  177
8   LTPLCVTLNCTDLEKGTNTT---NSTEKMMEKGEVKNCSFRITTDIKDRTRKEYALFYKL  177
9   LTPLCVTLNCTDLGNVTTTT---NSNGTLMEKGEVKNCSFKITTDTKDRTRKEYALFYKL  177
10  LTPLCVTLNCTDLEKGTNTT---NSTEKMMEKGEVKNCSFKITTDVKDRTRKEYALFYKL  177
11  LTPLCVTLNCTDLEKGTNTT---NSTEKMMEKGEVKNCSFKITTDMKDRTRKEYALFYKL  177
12  LTPLCVTLNCTDLEKGTNTT---NSTEKMMEKGEVKNCSFKITTDVKDRTRKEYALFYKL  177
13  LTPLCVTLNCTDLGKVTNTT---NSTEKMMEQGEVKNCSFKITTDVKDRTRKEYALFYKL  177
14  LTPLCVTLNCTDLEKGTNTT---NSTEKMMEKGEVKNCSFRITTDIKDRTRKEYALFYKL  177
15  LTPLCVTLNCTDLEKDTNTT---NSTEKMMEKGEVKNCSFKITTDMKDRTRKEYALFYKL  177
16  LTPLCVTLNCTDLEKGTNTT---NSTEKMMEKGEVKNCSFRITTDIKDRTRKEYALFYKL  177
17  LTPLCVTLNCTDLEKGTNTT---NSTEKMMEKGEVKNCSFRITTDIKDRTRKEYALFYKL  177
18  LTPLCVTLNCTDLGNVTTTT---NSNGTLMEKGEVKNCSFKITTDTKDRTRKEYALFYKL  177
19  LTPLCVTLNCTDLGNVTTTT---NSNGTLMEKGEVKNCSFKITTDTKDRTRKEYALFYKL  177
20  LTPLCVTLNCTDLGNVTTTT---NSNGTLMEKGEVKNCSFKITTDTKDRTRKEYALFYKL  177
21  LTPLCVTLNCTDLEKGTNTT---NSTEKMMEKGEVKNCSFRITTDIKDRTRKEYALFYKL  177
22  LTPLCVTLNCTDLGNVTTTT---NSNGTLMEKGEVKNCSFKITTEIKDRTRKEYALFYKL  177
23  LTPLCVTLKCTDLEKGTNTT---NSTEKMMEKGEVKNCSFKITTDVKDRTRKEYALFYKL  177
24  LTPLCVTLNCTDLEKGTNTT---NSTEKMMEKGEVKNCSFKITTDVKDRTRKEYALFYKL  177
25  LTPLCVTLNCTDLEKGTNTT---NSTEKMMEKGEVKNCSFRITTDIKDRTRKEYALFYKL  177
26  LTPLCVTLNCTDLEKGTNTT---NSTEKMMEKGEVKNCSFRITTDIKDRTRKEYALFYKL  177
27  LTPLCVTLNCTDLGNVTTTT---NSNGTLMEKGEVKNCSFKITTDTKDRTRKEYALFYKL  177
28  LTPLCVTLNCTDLEKDTNTT---NSTEKMMEKGEVKNCSFRITTDIKDRTRKEYALFYKL  177
29  LTPLCVTLNCTDLGNVTTTT---NSNGTLMEKGEVKNCSFKITTDTKDRTRKEYALFYKL  177
30  LTPLCVTLNCTDLGNVTTTT---NSNGTLMEKGEVKNCSFKITTDTKDRTRKEYALFYKL  177
31  LTPLCVTLNCTDLGNVTTTT---NSNGTLMEKGEVKNCSFKITTDTKDRTRKEYALFYKL  177
32  LTPLCVTLNCTDLGNVTTTT---NSNGTLMEKGEVKNCSFKITTDTKDRTRKEYALFYKL  177
33  LTPLCVTLNCTDLGNVTTTT---NSNGTLMEKGEVKNCSFKITTDTKDRTRKEYALFYKL  177
34  LTPLCVTLNCTDLEKGTNTT---NSTEKMMEKGEVKNCSFRITTDIKDRTRKEYALFYKL  177
35  LTPLCVTLNCTDLEKGTNTT---NSTEKMMEKGEVKNCSFRITTDIKDRTRKEYALFYKL  177
36  LTPLCVTLNCTDLGNVTTTT---NSNGTLMEKGEVKNCSFKITTEIKDRTRKEYALFYKL  177
37  LTPLCVTLNCTDLGNVTTTT---NSNGTLMEKGEVKNCSFKITTDVKDRTRKEYALFYKL  177
38  LTPLCVTLNCTDLEKGTNTT---NSTEKMMEKGEVKNCSFRITTDIKDRTRKEYALFYKL  177
39  LTPLCVTLNCTDLEKGTNTT---NSTEKMMEKGEVKNCSFRITTDIKDRTRKEYALFYKL  177
40  LTPLCVTLNCTDLEKGTNTT---NSTEKMMEKGEVKNCSFRITTDIKDRTRKEYALFYKL  177
41  LTPLCVTLNCTDLGNVTTTI---NSNGTLMEKGEVKNCSFKITTDTKDRTRKEYALFYKL  177
42  LTPLCVTLNCTDLGNVTTTT---NSNGTLMEKGEVKNCSFKITTDTKDRTRKEYALFYKL  177
43  LTPLCVTLNCTDLEKGTNTT---NSTEKMMEKGEVKNCSFRITTDIKDRTRKEYALFYKL  177
44  LTPLCVTLNCTDLGNVTTTT---NSNGTLMEKGEVKNCSFKITTEIKDRTRKEYALFYKL  177
45  LTPLCVTLNCTDLEKGTNTT---NSTEKMMEKGEVKNCSFKITTDVKDRTRKEYALFYKL  177
46  LTPLCVTLNCTDLEKGTNTT---NSTEKMMEKGEVKNCSFRITTDIKDRTRKEYALFYKL  177
```

	Sequence	
	DVVPIND-------TRYRLVSCNTSVITQACPKVSFEPIPIHYCAPAGFAILKCNDKQFI	
1	DVVPIND-------TRYRLVSCNTSVITQACPKVSFEPIPIHYCAPAGFAILKCNDKQFI	230
2	DVVPIND-------TRYRLVSCNTSVITQACPKVSFEPIPIHYCAPAGFAILKCNDKQFI	226
3	DVVPIND-------TRYRLVSCNTSVITQACPKVSFEPIPIHYCAPAGFAILKCNDKQFI	230
4	DVVPIND-------TRYRLVSCNTSVITQACPKVSFEPIPIHYCAPAGFAILKCNDKQFI	230
5	DVVPIND-------TRYRLVSCNTSVITQACPKVSFEPIPIHYCAPAGFAILKCNDKQFI	230
6	DVVPIND-------TRYRLVSCNTSVITQACPKVSFEPIPIHYCAPAGFAILKCNDKQFI	230
7	DVVPIND-------TRYRLVSCNTSVITQACPKVSFEPIPIHYCAPAGFAILKCNDKQFI	230
8	DVVPIND-------TRYRLVSCNTSVITQACPKVSFEPIPIHYCAPAGFAILKCNDKQFI	230
9	DVVPIND-------TRYRLVSCNTSVITQACPKVSFEPIPIHYCAPAGFAILKCNDKQFI	230
10	DVVPIND-------TRYRLVSCNTSVITQACPKVSFEPIPIHYCAPAGFAILKCNDKQFI	230
11	DVVPIND-------TRYRLVSCNTSVITQACPKVSFEPIPIHYCAPAGFAILKCNDKQFI	230
12	DVVPIND-------TRYRLVSCNTSVITQACPKVSFEPIPIHYCAPAGFAILKCNDKQFI	230
13	DVVPIND-------TRYRLVSCNTSVITQACPKVSFEPIPIHYCAPAGFAILKCNDKQFN	230
14	DVVPIND-------TRYRLVSCNTSVITQACPKVSFEPIPIHYCAPAGFAILKCNDKQFI	230
15	DVVPIND-------TRYRLVSCNTSVITQACPKVSFEPIPIHYCAPAGFAILKCNDKQFI	230
16	DVVPIND-------TRYRLVSCNTSVITQACPKVSFEPIPIHYCAPAGFAILKCNDKQFI	230
17	DVVPIND-------TRYRLVSCNTSVITQACPKVSFEPIPIHYCAPAGFAILKCNDKQFI	230
18	DVVPIND-------TRYRLVSCNTSVITQACPKVSFEPIPIHYCAPAGFAILKCNDKQFI	230
19	DVVPIND-------TRYRLVSCNTSVITRACPKVSFEPIPIHYCAPAGFAILKCNDKQFI	230
20	DVVPIND-------TRYRLVSCNTSVITQACPKVSFEPIPIHYCAPAGFAILKCNDKQFI	230
21	DVVPIND-------TRYRLVSCNTSVITQACPKVSFEPIPIHYCAPAGFAILKCNDKQFI	230
22	DVVPIND-------TGYRLVSCNTSVITQACPKVSFEPIPIHYCAPAGFAILKCNDKQFI	230
23	DVVPIND-------TRYRLVSCNTSVITQACPKVSFEPIPIHYCAPAGFAILKCNDKQFI	230
24	DVVPIND-------TRYRLVSCNTSVITQACPKVSFEPIPIHYCAPAGFAILKCNDKQFI	230
25	DVVPIND-------TRYRLVSCNTSVITQACPKVSFEPIPIHYCAPAGFAILKCNDKQFI	230
26	DVVPINE-------------SCNTSVITQACPKVSFEPIPIHYCAPAGFAILKCNDKQFI	224
27	DVVPIND-------TRYRLVSCNTSVITQACPKVSFEPIPIHYCAPAGFAILKCNDKQFI	230
28	DVVPIND-------TRYRLVSCNTSVITQACPKVSFEPIPIHYCAPAGFAILKCNDKQFI	230
29	DVVPIND-------TRYRLVSCNTSVITQACPKVSFEPIPIHYCAPAGFAILKCNDKQFI	230
30	DVVPIND-------TRYRLVSCNTSVITQACPKVSFEPIPIHYCAPAGFAILKCNDKQFI	230
31	DVVPIND-------TRYRLVSCNTSVITQACPKVSFEPIPIHYCAPAGFAILKCNDKQFI	230
32	DVVPIND-------TRYRLVSCNTSVITQACPKVSFEPIPIHYCAPAGFAILKCNDKQFI	230
33	DVVPIND-------TRYRLVSCNTSVITQACPKVSFEPIPIHYCAPAGFAILKCNDKQFI	230
34	DVVPIND-------TRYRLVSCNTSVITQACPKVSFEPIPIHYCAPAGFAILKCNDKQFI	230
35	DVVPIND-------TRYRLVSCNTSVITQACPKVSFEPIPIHYCAPAGFAILKCNDKQFI	230
36	DVVPIND-------TRYRLVSCNTSVITQACPKVSFEPIPIHYCAPAGFAILKCNDKQFI	230
37	DVVPIND-------TRYRLVSCNTSVITQACPKVSFEPIPIHYCAPAGFAILKCNDKQFI	230
38	DVVPIND-------TRYRLVSCNTSVITQACPKVSFEPIPIHYCAPAGFAILKCNDKQFI	230
39	DVVPIND-------TRYRLVSCNTSVITQACPKVSFEPIPIHYCAPAGFAILKCNDKQFI	230
40	DVVPIND-------TRYRLVSCNTSVITQACPKVSFEPIPIHYCAPAGFAILKCNDKQFI	230
41	DVVPIND-------TRYRLVSCNTSVITQACPKVSFEPIPIHYCAPAGFAILKCNDKQFI	230
42	DVVPIND-------TRYRLVSCNTSVITQACPKVSFEPIPIHYCAPAGFAILKCNDKQFI	230
43	DVVPIND-------TRYRLVSCNTSVITQACPKVSFEPIPIHYCAPAGFAILKCNDKQFI	230
44	DVVPIND-------TRYRLVSCNTSVITQACPKVSFEPIPIHYCAPAGFAILKCNDKQFI	230
45	DVVPIND-------TRYRLVSCNTSVITQACPKVSFEPIPIHYCAPAGFAILKCNDKQFI	230
46	DVVPIND-------TRYRLVSCNTSVITQACPKVSFEPIPIHYCAPAGFAILKCNDKQFI	230

	GTGPCTNVSTVQCTHGIRPVVSTQLLLNGSLAEEEVVIRSVNFSDNAKTIIVQLNKSVEI	
1	GTGPCTNVSTVQCTHGIRPVVSTQLLLNGSLAEEEVVIRSVNFSDNAKTIIVQLNKSVEI	290
2	GTGPCTNVSTVQCTHGIRPVVSTQLLLNGSLAEEEVVIRSVNFSDNAKTIIVQLNKSVEI	286
3	GTGPCTNVSTVQCTHGIRPVVSTQLLLNGSLAEEEVVIRSVNFSDNAKTIIVQLNKSVEI	290
4	GTGPCTNVSTVQCTHGIRPVVSTQLLLNGSLAEEEVVIRSVNFSDNAKTIIVQLNKSVEI	290
5	GTGPCTNVSTVQCTHGIRPVVSTQLLLNGSLAEEEVVIRSVNFSDNAKTIIVQLNKSVEI	290
6	GTGPCTNVSTVQCTHGIRPVVSTQLLLNGSLAEEEVVIRSVNFSDNAKTIIVQLNKSVEI	290
7	GTGPCTNVSTVQCTHGIRPVVSTQLLLNGSLAEEEVVIRSVNSSDNAKTIIVQLNKSVEI	290
8	GTGPCTNVSTVQCTHGIRPVVSTQLLLNGSLAEEEVVIRSVNFSDNAKTIIVQLNNSVEI	290
9	GTGPCTNVSTVQCTHGIRPVVSTQLLLNGSLAEEEVVIRSVNFSDNAKTIIVQLNKSVEI	290
10	GTGPCTNVSTVQCTHGIRPVVSTQLLLNGSLAEEEVVIRSVNFRDNAKTIIVQLNKSVEI	290
11	GTGPCTNVSTVQCTHGIRPVVSTQLLLNGSLAEEEVVIRSVNFRDNAKTIIVQLNKSVEI	290
12	GTGPCTNVSTVQCTHGIRPVVSTQLLLNGSLAEEEVVIRSVNFRDNAKTIIVQLNKSVEI	290
13	GTGPCTNVSTVQCTHGIRPVVSTQLLLNGSLAEEEVVIRSVNFSDNAKTIIVQLNKSVEI	290
14	GTGPCTNVSTVQCTHGIRPVVSTQLLLNGSLAEEEVVIRSVNFSDNAKTIIVQLNKSVEI	290
15	GTGPCTNVSTVQCTHGIRPVVSTQLLLNGSLAEEEVVIRSVNFSDNAKTIIVQLNKSVEI	290
16	GTGPCTNVSTVQCTHGIRPVVSTQLLLNGSLAEEEVVIRSVNFSDNAKTIIVQLNKSVEI	290
17	GTGPCTNVSTVQCTHGIRPVVSTQLLLNGSLAEEEVVIRSVNFSDNAKTIIVQLNKSVEI	290
18	GTGPCTNVSTVQCTHGIRPVVSTQLLLNGSLAEEEVVIRSVNFSDNAKTIIVQLNKSVEI	290
19	GTGPCTNVSTVQCTHGIRPVVSTQLLLNGSLAEEEVVIRSVNFSDNAKTIIVQLNKSVEI	290
20	GTGPCTNVSTVQCTHGIRPVVSTQLLLNGSLAEEEVVIRSVNFSDNAKTIIVQLNKSVEI	290
21	GTGPCTNVSTVQCTHGIRPVVSTQLLLNGSLAEEEVVIRSVNFSDNAKTIIVQLNKSVEI	290
22	GTGPCTNVSTVQCTHGIRPVVSTQLLLNGSLAEEEVVIRSVNFNDNAKTIIVQLNKSVEI	290
23	GTGPCTNVSTVQCTHGIRPVVSTQLLLNGSLAEEEVVIRSVNFRDNAKTIIVQLNKSVEI	290
24	GTGPCTNVSTVQCTHGIRPVVSTQLLLNGSLAEEEVVIRSVNFRDNAKTIIVQLNKSVEI	290
25	GTGPCTNVSTVQCTHGIRPVVSTQLLLNGSLAEEEVVIRSVNFSDNAKTIIVQLNKSVEI	290
26	GTGPCTNVSTVQCTHGIRPVVSTQLLLNGSLAEEEVVIRSVNFSDNAKTIIVQLNKSVEI	284
27	GTGPCTNVSTVQCTHGIRPVVSTQLLLNGSLAEEEVVIRSVNFSDNAKTIIVQLNKSVEI	290
28	GTGPCTNVSTVQCTHGIRPVVSTQLLLNGSLAEEEVVIRSVNFSDNAKTIIVQLNKSVEI	290
29	GTGPCTNVSTVQCTHGIRPVVSTQLLLNGSLAEEEVVIRSVNFSDNAKTIIVQLNKSVEI	290
30	GTGPCTNVSTVQCTHGIRPVVSTQLLLNGSLAEEEVVIRSVNFSDNAKTIIVQLNKSVEI	290
31	GTGPCTNVSTVQCTHGIRPVVSTQLLLNGSLAEEEVVIRSVNFSDNAKTIIVQLNKSVEI	290
32	GTGPCTNVSTVQCTHGIRPVVSTQLLLNGSLAEEEVVIRSVNFSDNAKTIIVQLNKSVEI	290
33	GTGPCTNVSTVQCTHGIRPVVSTQLLLNGSLAEEEVVIRSVNFSDNAKTIIVQLNKSVEI	290
34	GTGPCTNVSTVQCTHGIRPVVSTQLLLNGSLAEEEVVIRSVNFSDNAKTIIVQLNKSVEI	290
35	GTGPCTNVSTVQCTHGIRPVVSTQLLLNGSLAEEEVVIRSVNFSDNAKTIIVQLNKSVEI	290
36	GTGPCTNVSTVQCTHGIRPVVSTQLLLNGSLAEEEVVIRSVNFNDNAKTIIVQLNKSVEI	290
37	GTGPCTNVSTVQCTHGIRPVVSTQLLLNGSLAEEEVVIRSVNFSDNAKTIIVQLNKSVEI	290
38	GTGPCTNVSTVQCTHGIRPVVSTQLLLNGSLAEEEVVIRSVNFSDNAKTIIVQLNKSVEI	290
39	GTGPCTNVSTVQCTHGIRPVVSTQLLLNGSLAEEEVVIRSVNFSDNAKTIIVQLNKSVEI	290
40	GTGPCTNVSTVQCTHGIRPVVSTQLLLNGSLAEEEVVIRSVNFSDNAKTIIVQLNKSVEI	290
41	GTGPCTNVSTVQCTHGIRPVVSTQLLLNGSLAEEEVVIRSVNFSDNAKTIIVQLNKSVEI	290
42	GTGPCTNVSTVQCTHGIRPVVSTQLLLNGSLAEEEVVIRSVNFSDNAKTIIVQLNKSVEI	290
43	GTGPCTNVSTVQCTHGIRPVVSTQLLLNGSLAEEEVVIRSVNFSDNAKTIIVQLNKSVEI	290
44	GTGPCTNVSTVQCTHGIRPVVSTQLLLNGSLAEEEVVIRSVNFRDNAKTIIVQLNKSVEI	290
45	GTGPCTNVSTVQCTHGIRPVVSTQLLLNGSLAEEEVVIRSVNFRDNAKTIIVQLNKSVEI	290
46	GTGPCTNVSTVQCTHGIRPVVSTQLLLNGSLAEEEVVIRSVNFSDNAKTIIVQLNKSVEI	290

	NCTRPHNNTRKSIPMGPGKAFYARGDIIGDIRKAYCEINGTEWHSTLKLVVGKLREKY-N	
1	TCTRPNNNTRKSIPMGPGKAFYARGDITGDIRKAYCEINGTEWHSTLKLVVEKLREQY-N	349
2	NCTRPNNNTRKSIPMGPGKAFYARGDIIGDIRKASCELDGTEWHSTLKLVVEKLRKQY-N	345
3	NCTRPNNNTRKSIPMGPGKAFYARGDIIGDIRKASCELDGTEWHSTLKLVVEKLRKQY-N	349
4	NCTRPHNNTRKSIPMGPGKAFYARGDIIGDIRKAYCEINGTEWHSTLKLVVGKLREKY-N	349
5	NCTRPHNNTRKSIPMGPGKAFYARGDIIGDIRKAYCEINGTEWHSTLKLVVGKLREKY-N	349
6	NCTRPHNNTRKSIPMGPGKAFYARGDIIGDIRKAYCEINGTEWHSTLKLVVGKLREKY-N	349
7	NCTRPHNNTRKSIPMGPGKAFYARGDIIGDIRKAYCEINGTEWHSTLKLVVEKLREQY-N	349
8	NCTRPHNNTRKSIPMGPGKAFYARGDIIGDIRKAYCKINGTEWHSTLKLVVGKLREKY-N	349
9	NCTRPNNNTRKSIPMGPGKAFYARGDIIGDIRKASCELDGTEWHSTLKLVVEKLRKQY-N	349
10	NCTRPHNNTRKSIPMGPGKAFYARGDIIGDIRKAYCEINGTEWHSTLKLVVGKLREKY-N	349
11	NCTRPHNNTRKSIPMGPGKAFYARGDIIGDIRKAYCTINGTEWHSTLKLVVGKLREKY-N	349
12	NCTRPHNNTRKSIPMGPGKAFYARGDIIGDIRKAYCEINGTEWHSTLKLVVGKLREKY-N	349
13	NCTRPHNNTRKSIPMGPGKAFYARGDIIGDIRKAYCNINGTEWHSTLKLVVEKLREQY-N	349
14	NCTRPHNNTRKSIPMGPGKAFYARGDIIGDIRKAYCEINGTEWHSTLKLVVGKLREKY-N	349
15	NCTRPHNNTRKSIPMGPGKAFYARGDIIGDIRKAYCEINGTEWHSTLKLVVEKLREKY-N	349
16	NCTRPHNNTRKSIPMGPGKAFYARGDIIGDIRKAYCEINGTEWHSTLKLVVGKLREKY-N	349
17	NCTRPHNNTRKSIPMGPGKAFYARGDIIGDIRKAYCEINGTEWHSTLKLVVGKLREKY-N	349
18	NCTRPNNNTRKSIPMGPGKAFYARGDIIGDIRKASCELDGTEWHSTLKLVVEKLRKQY-N	349
19	NCTRPNNNTRKSIPMGPGKAFYARGDIIGDIRKASCELNGTEWHSTLKLVVEKLRKQY-N	349
20	NCTRPNNNTRKSIPMGPGKAFYARGDIIGDIRKASCELDGTEWHSTLKLVVEKLRKQY-N	349
21	NCTRPHNNTRKSIPMGPGKAFYARGDIIGDIRKAYCEINGTEWHSTLKLVVGKLRKI---	347
22	NCTRPHNNTRKSIPMGPGKAFYAKGDIIGDIRKAYCEINGTEWHSTLKLVVEKLRERY-N	349
23	NCTRPHNNTRKSIPMGPGKAFYARGDIIGDIRKAYCEINGTEWHSTLKLVVGKLREKY-N	349
24	NCTRPHNNTRKSIPMGPGKAFYARGDIIGDIRKAYCEINGTEWHSTLKLVVGKLREKY-N	349
25	NCTRPHNNTRKSIPMGPGKAFYARGDIIGDIRKAYCEINGTEWHSTLKLVVGKLREKY-N	349
26	NCTRPHNNTRKSIPMGPGKAFYARGDIIGDIRKAYCEINGTEWHSTLKLVVGKLREKY-N	343
27	SCTRPNNNTRKSIPMGPGKAFYARGDIIGDIRKASCELDGTEWHSTLKLVVEKLRKQY-N	349
28	NCTRPHNNTRKSIPMGPGKAFYARGDIIGDIRKAYCEINGTEWHSTLKLVVGKLREKY-N	349
29	NCTRPNNNTRKSIPMGPGKAFYARGDIIGDIRKASCELDGTEWHSTLKLVVEKLRKQY-N	349
30	NCTRPNNNTRKSIPMGPGKAFYARGDIIGDIRKASCELDGTEWHSTLKLVAEKLRKQY-N	349
31	NCTRPNNNTRKSIPMGPGKAFYARGDIIGDIRKASCELDGTEWHSTLKLVVEKLRKQY-N	349
32	NCTRPNNNTRKSIPMGPGKAFYARGDIIGDIRKASCELDGTEWHSTLKLVVEKLRKQY-N	349
33	NCTRPNNNTRKSIPMGPGKAFYARGDIIGDIRKASCELDGTEWHSTLKLVVEKLRKQY-N	349
34	NCTRPHNNTRKSIPMGPGKAFYARGDIIGDIRKAYCEINGTEWHSTLKLVVGKLREKY-N	349
35	NCTRPHNNTRKSIPMGPGKAFYARGDIIGDIRKAYCEINGTEWHSTLKLVVGKLREKY-N	349
36	NCTRPHNNTRKSIPMGPGKAFYAKGDIIGDIRKAYCEINGTEWHSTLKLVVEKLRERY-N	349
37	NCTRPHNNTRKSIPMGPGKAFYARGDIIGDIRKAYCKINGTEWHSTLKLVVEKLREQY-N	349
38	NCTRPHNNTRKSIPMGPGKAFYARGDIIGDIRKAYCEINGTEWHSTLKLVVGKLREKY-N	349
39	NCTRPHNNTRKSIPMGPGKAFYARGDIIGDIRKAYCEINGTEWHSTLKLVVGKLREKY-N	349
40	NCTRPHNNTRKSIPMGPGKAFYARGDIIGDIRKAYCEINGTEWHSTLKLVVGKLREKY-N	349
41	NCTRPNNNTRKSIPMGPGKAFYARGDIIGDIRKASCELDGTEWHSTLKLVVEKLRKQY-N	349
42	NCTRPNNNTRKSIPMGPGKAFYARGDIIGDIRKASCKLDGTEWHSTLKLVVEKLRKQY-N	349
43	NCTRPHNNTRKSIPMGPGKAFYARGDIIGDIRKAYCEINGTEWHSTLKLVVGKLREKY-N	349
44	NCTRPHNNTRKSIPMGPGKAFYARGDIIGNIRKAYCEINGTEWHSTLKLVVEKLREKY-N	349
45	NCTRPHNNTRKSIPMGPGKAFYARGDIIGDIRKAYCEINGTEWHSTLKLVVGKLREKY-N	349
46	NCTRPHNNTRKSIPMGPGKAFYARGDIIGDIRKAYCEINGTEWHSTLKLVVGKLREKY-N	349

```
    KTIVFNRSSGGDPEIVMYSFNCGGEFFYCNSTKLFNSTWPWND-TKGSHDTNDTLMLPCR

1   KTIVFNRSSGGDPEIVMYSFNCGGEFFYCNSTKLFNSTWPWND-TKGSHDTNGTLILPCK  408
2   KTIIFNRSSGGDPEIVMYSFNCGGEFFYCNSTKLFNSTWPWND-TKGSHDTNGTLILPCR  404
3   KTIVFNRSSGGDPEIVMYSFNCGGEFFYCNSTKLFNSTWPWND-TKGSHDTNGTLILPCR  408
4   KTIVFNRSSGGDPEIVMYSFNCGGEFFYCNSTKLFNSTWPWND-TKGSHDTNDTLMLPCK  408
5   KTIVFNRSSGGDPEIVMYSFNCGGEFFYCNSTKLFNSTWPWND-TKGSHDTNDTLMLPCK  408
6   KTIVFNRSSGGDPEIVMYSFNCGGEFFYCNSTKLFNSTWPWND-TKGSHDTNDTLMLPCK  408
7   KTIVFNRSSGGDPEIVMYSFNCGGEFFYCNSTKLFNSTWPWND-TKGSHDTNGTLILPCR  408
8   KTIVFNRSSGGDPEIVMYSFNCGGEFFYCNSTKLFNSTWPWND-TKGSHDTNDTLMLPCK  408
9   KTIVFNRSSGGDPEIVMYSFNCGGEFFYCNSTKLFNSTWPWND-TKGSHDTNGTLILPCR  408
10  KTIVFNRSSGGDPEIVMYSFNCGGEFFYCNSTKLFNSTWPWND-TKGSHDTNDTLMLPCR  408
11  KTIVFKRSSGGDPEIVMYSFNCGGEFFYCNSTKLFNSTWPWND-TKGSHDTNDTLMLPCK  408
12  KTIVFNRSSGGDPEIVMYSFNCGGEFFYCNSTKLFNSTWPWND-TKGSHDTNDTLMLPCR  408
13  KTIVFNRSSGGDPEIVMYSFNCGGEFFYCNSTKLFNSTWPWND-TKGSHDTNDTLMLPCK  408
14  KTIVFNRSSGGDPEIVMYSFNCGGEFFYCNSTKLFNSTWPWND-TKGSHDTNDTLMLPCK  408
15  KTIVFNRSSGGDPEIVMYSFNCGGEFFYCNSTKLFNSTWPWND-TKGSHDTNDTLMLPCK  408
16  KTIVFNRSSGGDPEIVMYSFNCGGEFFYCNSTKLFNSTWPWND-TKGSHDTNDTLMLPCK  408
17  KTIVFNRSSGGDPEIVMYSFNCGGEFFYCNSTKLFNSTWPWND-TKGSHDTNDTLMLPCK  408
18  KTTVFNRSSGGDPEIVMYSFNCGGEFFYCNSTKLFNSTWPWND-TKGSHDTNGTLILPCR  408
19  KTIVFNRSSGGDPEIVMYSFNCGGEFFYCNSTKLFNSTPWNDT--KGSHDTNGTLILPCR  407
20  KTIVFNRSSGGDPEIVMYSFNCGGEFFYCNSTKLFNSTWPWND-TKGSHDTNGTLILPCR  408
21  -------SSGGDPEIVMYSFNCGGEFFYCNSTKLFNSTWPWND-TKGSHDTNDTLMLPCK  399
22  KTIVFNRSSGGDPEIVMYSFNCGGEFFYCNSTKLFNSTWPWND-TKGSHDTNGTLTLPCR  408
23  KTIVFNRSSGGDPEIVMYSFNCGGEFFYCNSTKLFNSTWPWND-TKGSHDTNDTLMLPCR  408
24  KTIVFNRSSGGDPEIVMYSFNCGGEFFYCNSTKLFNSTWPWND-TKGSHDTNDTLMLPCR  408
25  KTIVFNRSSGGDPEIVMYSFNCGGEFFYCNSTKLFNSTWPWND-TKGSHDTNDTLMLPCK  408
26  KTIVFNRSSGGDPEIVMYSFNCGGEFFYCNSTKLFNSTWPWND-TKGSHDTNDTLMLPCK  402
27  KTIVFNRSSGGDPEIVMYSFNCGGEFFYCNSTKLFNSTWPWND-TKGSHDTNGTLILPCR  408
28  KTIVFNRSSGGDPEIVMYSFNCGGEFFYCNSTKLFNSTWPWND-TKGSHDTNDTLMLPCK  408
29  KTIVFNRSSGGDPEIVMYSFNCGGEFFYCNSTKLFNSTWPWND-TKGSHDTNGTLILPCR  408
30  KTIVFNRSSGGDPEIVMYSFNCGGEFFYCNSTKLFNSTWPWND-TKGSHDTNGTLILPCR  408
31  KTIVFNRSSGGDPEIVMYSFNCGGEFFYCNSTKLFNSTWPWND-TKGSHDTNGTLILPCR  408
32  KTIVFNRSSGGDPEIVMYSFNCGGEFFYCNSTKLFNSTWPWND-TKGSHDTNGTLILPCR  408
33  KTIVFNRSSGGDPEIVMYSFNCGGEFFYCNSTKLFNSTWPWND-TKGSHDTNGTLILPCR  408
34  KTIVFNRSSGGDPEIVMYSFNCGGEFFYCNSTKLFNSTWPWND-TKGSHDTNDTLMLPCK  408
35  KTIVFNRSSGGDPEIVMYSFNCGGEFFYCNSTKLFNSTWPWND-TKGSHDTNDTLMLPCK  408
36  KTIVFNRSSGGDPEIVMYSFNCGGEFFYCNSTKLFNSTWPWND-TKGSHDTNGTLTLPCR  408
37  KTIVFNRSSGGDPEIVMYSFNCGGEFFYCNSTKLFNSTWPWND-TKGSHDTNDTLMLPCR  408
38  KTIVFNRSSGGDPEIVMYSFNCGGEFFYCNSTKLFNSTWPWND-TKGSHDTNDTLMLPCK  408
39  KTIVFNRSSGGDPEIVMYSFNCGGEFFYCNSTKLFNSTWPWND-TKGSHDTNDTLMLPCK  408
40  KTIVFNRSSGGDPEIVMYSFNCGGEFFYCNSTKLFNSTWPWND-TKGSHDTNDTLMLPCK  408
41  KTIIFNRSSGGDPEIVMYSFNCGGEFFYCNSTKLFNSTWPWND-TKGSHDTNGTLILPCR  408
42  KTIVFNRSSGGDPEIVMYSFNCGGEFFYCNSTKLFNSTWPWND-TKGSHDTNGTLILPCR  408
43  KTIVFNRSSGGDPEIVMYSFNCGGEFFYCNSTKLFNSTWPWND-TKGSHDTNDTLMLPCK  408
44  KTIVFNRSSGGDPEIVMYSFNCGGEFFYCNSTKLFNSTWPWND-TKGSHDTNGTLILPCR  408
45  KTIVFNRSSGGDPEIVMYSFNCGGEFFYCNSTKLFNSTWPWND-TKGSHDTNDTLMLPCR  408
46  KTIVFNRSSGGDPEIVM-SFNCGGEFFYCNSTKLFNSTWPWND-TKGSHDTNDTLMLPCK  407
```

	IKQIINMWQGVGKAMYAPPIEGXIRCSSNITGLLLTRDGG-YESNETDEIFRPGGGDMRD	
1	IKQIINMWQGVGKAMYAPPIEGKIRCSSNITGLLLTRDGG-YESNETDEIFRPGGGDMRD	467
2	IKQIINMWQGVGKAMYAPPIEGEIRCSSNITGLLLTRDGG-YESNETDEIFRPGGGDMRD	463
3	IKQIINMWQGVGKAMYAPPIEGEIRCSSNITGLLLTRDGG-YESNETDEIFRPGGGDMRD	467
4	IKQIINMWQGVGKAMYAPPIEGQIRCSSNITGLLLTRDGG-YESNETDEIFRPGGGDMRD	467
5	IKQIINMWQGVGKAMYAPPIEGQIRCSSNITGLLLTRDGG-YESNETDEIFRPGGGDMRD	467
6	IKQIINMWQGVGKAMYAPPIEGQIRCSSNITGLLLTRDGG-YESNETDEIFRPGGGDMRD	467
7	IKQIINMWQGVGKAMYAPPIEGEIRCSSNITGLLLTRDGG-YESNKTDEIFRPGGGDMRD	467
8	IKQIINMWQGVGKAMYAPPIEGQIRCSSNITGLLLTRDGG-YESNETDEIFRPGGGDMRD	467
9	IKQIINMWQGVGKAMYAPPIEGEIRCS-----LLLTRDGG-YESNETDEIFRPGGGDMRD	462
10	IKQIINMWQGVGKAMYAPPIEGIIRCSSNITGLLLTRDGG-YESNETDEIFRPGGGDMRD	467
11	IKQIINMWQGVGKAMYAPPIEGQIRCSSNITGLLLTRDGG-YESNETDEIFRPGGGDMRD	467
12	IKQIINMWQGVGKVMYAPPIEGIIRCSSNITGLLLTRDGG-YESNETDEIFRPGGGDMRD	467
13	IKQIINMWQGVGKAMYAPPIEGQIRCSSNITGLLLTRDGG-YESNKTDEIFRPGGGDMRD	467
14	IKQIINMWQGVGKAMYAPPIEGQIRCSSNITGLLLTRDGG-YESNETDEIFRPGGGDMRD	467
15	IKQIINMWQGVGKAMYAPPIEGQIRCSSNITGLLLTRDGG-YESNETDEIFRPGGGDMRD	467
16	IKQIINMWQGVGKAMYAPPIEGQIRCSSNITGLLLTRDGG-YESNETDEIFRPGGGDMRD	467
17	IKQIINMWQGVGKAMYAPPIEGQIRCSSNITGLLLTRDGG-YESNETDEIFRPGGGDMRD	467
18	IKQIINMWQGVGKAMYAPPIEGEIRCSSNITGLLLTRDGG-YESNETDEIFRPGGGDMRD	467
19	IKQIINMWQGVGKAMYAPPIEGEIRCSSNITGLLLTRDGG-YESNETDEIFRPGGGDMRD	466
20	IKQIINMWQGVGKAMYAPPIEGEIRCSSNITGLLLTRDGG-YESNETDEIFRPGGGDMRD	467
21	IKQIINMWQGVGKAMYAPPIEGQIRCSSNITGLLLTRDGG-YESNETDEIFRPGGGDMRD	458
22	IKQIINMWQGVGKAMYAPPIEGKIRCSSNITGLLLTRDGG-YESNKTDEIFRPGGGDMRD	467
23	IKQIINMWQGVGKAMYAPPIEGIIRCSSNITGLLLTRDGG-YESNETDEIFRPGGGDMRD	467
24	IKQIINMWQGVGKAMYAPPIEGIIRCSSNITGLLLTRDGG-YESNETDEIFRPGGGDMRD	467
25	IKQIINMWQGVGKAMYAPPIEGQIRCSSNITGLLLTRDGG-YESNETDEIFRPGGGDMRD	467
26	IKQIINMWQGVGKAMYAPPIEGQIRCSSNITGLLLTRDGG-YESNETDEIFRPGGGDMRD	461
27	IKQIINMWQGVGKAMYAPPIEGEIRCSSNITGLLLTRDGG-YESNETDEIFRPGGGDMRD	467
28	IKQIINMWQGVGKAMYAPPIEGQIRCSSNITGLLLTRDGG-YESNETDEIFRPGGGDMRD	467
29	IKQIINMWQGVGKAMYAPPIEGEIRCSSNITGLLLTRDGG-YESNETDEIFRPGGGDMRD	467
30	IKQIINMWQGVGKAMYAPPIEGEIRCSSNITGLLLTRDGG-YESNETDEIFRPGGGDMRD	467
31	IKQIINMWQGVGKAMYAPPIEGEIRCSSNITGLLLTRDGG-YESNETDEIFRPGGGDMRD	467
32	IKQIINMWQGVGKAMYAPPIEGEIRCSSNITGLLLTRDGG-YESNETDEIFRPGGGDMRD	467
33	IKQIINMWQGVGKAMYAPPIEGEIRCSSNITGLLLTRDGG-YESNETDEIFRPGGGDMRD	467
34	IKQIINMWQGVGKAMYAPPIEGQIRCSSNITGLLLTRDGG-YESNETDEIFRPGG-----	462
35	IKQIINMWQGVGKAMYAPPIEGQIRCSSNITGLLLTRDGG-YESNETDEIFRPGGGDMRD	467
36	IKQIINMWQGVGKAMYAPPIEGKIRCSSNITGLLLTRDGG-YESNKTDEIFRPGGGDMRD	467
37	IKQIINMWQGVGKAMYAPPIEGIIRCSSNITGLLLTRDGG-YESNETDEIFRPGGGDMRD	467
38	IKQIINMWQGVGKAMYAPPIEGQIRCSSNITGLLLTRDGG-YESNETDEIFRPGGGDMRD	467
39	IKQIINMWQGVGKAMYAPPIEGQIRCSSNITGLLLTRDGG-YESNETDEIFRPGGGDMRD	467
40	IKQIINMWQGVGKAMYAPPIEGQIRCSSNITGLLLTRDGG-YESNETDEIFRPGGGDMRD	467
41	IKQIINMWQGVGKAMYAPPIEGEIRCSSNITGLLLTRDGG-YESNETDEIFRPGGGDMRD	467
42	IKQIINMWQGVGKAMYAPPIEGEIRCSSNITGLLLTRDGG-YESNETDEIFRPGGGDMRD	467
43	IKQIINMWQGVGKAMYAPPIEGQIRCSSNITGLLLTRDGG-YESNETDEIFRPGGGDMRD	467
44	IKQIINMWQGVGKAMYAPPIEGEIRCSSNITGLLLTRDGG-YESNETDEIFRPGGGDMRD	467
45	IKQIINMWQGVGKAMYAPPIEGIIRCSSNITGLLLTRDGG-YESNETDEIFRPGGGDMRD	467
46	IKQIINMWQGVGKAMYAPPIEGQIRCSSNITGLLLTRDGG-YESNETDEIFRPGGGDMRD	466

	NWRSELYKYKVVKIEPLGVAPTKAKRRVVQREKRAFG-LGAVFLGFLGAAGSTMGAASIT	
1	NWRSELYKYKVVKIEPLGVAPTKAKRRVVQREKRAFG-LGAVFLGFLGAAGSTMGAASIT	526
2	NWRSELYKYKVVKIEPLGVAPTKAKRRVVQREKRAFG-LGAVFLGFLGAAGSTMGAASIT	522
3	NWRSELYKYKVVKIEPLGVAPTKAKRRVVQREKRAFG-LGAVFLGFLGAAGSTMGAASIT	526
4	NWRSELYKYKVVKIEPLGVAPTKAKRRVVQREKRAFG-LGAVFLGFLGAAGSTMGAASIT	526
5	NWRSELYKYKVVKIEPLGVAPTKAKRRVVQREKRAFG-LGAVFLGFLGAAGSTMGAASIT	526
6	NWRSELYKYKVVKIEPLGVAPTKAKRRVVQREKRAFG-LGAVFLGFLGAAGSTMGAASIT	526
7	NWRSELYKYKVVKIEPLGVAPTKAKRRVVQREKRAFG-LGAVFLGFLGAAGSTMGAASIT	526
8	NWRSKLYKYKVVKIEPLGVAPTKAKRRVVQREKRAFE-LGAVFLGFLGAAGSTMGAASIT	526
9	NWRSELYKYKVVKIEPLGVAPTKAKRRVVQREKRAFG-LGAVFLGFLGAAGSTMGAASIT	521
10	NWRSELYKYKVVKIEPLGVAPTKAKRRVVQREKRAFG-LGAVFLGFLGAAGSTMGAASIT	526
11	NWRSELYKYKVVKIEPLGVAPTKAKRRVVQREKRAFG-LGAVFLGFLGAAGSTMGAASIT	526
12	NWRSELYKYKVVKIEPLGVAPTKAKRRVVQREKRAFG-LGAVFLGFLGAAGSTMGAASIT	526
13	NWRSELYKYKVVKIEPLGVAPTKAKRRVVQREKRAFG-LGAVFLGFLGAAGSTMGAASIT	526
14	NWRSELYKYKVVKIEPLGVAPTKAKRRVVQREKRAFG-LGAVFLGFLGAAGSTMGAASIT	526
15	NWRSELYKYKVVKIEPLGVAPTKAKRRVVQREKRAFG-LGAVFLGFLGAAGSTMGAASIT	526
16	NWRSELYKYKVVKIEPLGVAPTKAKRRVVQREKRAFG-LGAVFLGFLGAAGSTMGAASIT	526
17	NWRSELYKYKVVKIEPLGVAPTKAKRRVVQREKRAFG-LGAVFLGFLGAAGSTMGAASIT	526
18	NWRSELYKYKVVKIEPLGVAPTKAKRRVVQREKRAFG-LGAVFLGFLGAAGSTMGAASIT	526
19	NWRSELYKYKVVKIEPLGVAPTKAKRRVVQREKRAFG-LGAVFLGFLGAAGSTMGAASIT	525
20	NWRSELYKYKVVKIEPLGVAPTKAKRRVVQREKRAFG-LGAVFLGFLGAAGSTMGAASIT	526
21	NWRSELYKYKVVKIEPLGVAPTKAKRRVVQREKRAFG-LGAVFLGFLGAAGSTMGAASIT	517
22	NWRSELYKYKVVKIEPLGVAPTKAKRRVVQREKRAFG-LGAVFLGFLGAAGSTMGAASIT	526
23	NWRSELYKYKVVKIEPLGVAPTKAKRRVVQREKRAFG-LGAVFLGFLGAAGSTMGAASIT	526
24	NWRSELYKYKVVKIEPLGVAPTKAKRRVVQREKRAFG-LGAVFLGFLGAAGSTMGAASIT	526
25	NWRSELYKYKVVKIEPLGVAPTKAKRRVVQREKRAFG-LGAVFLGFLGAAGSTMGAASIT	526
26	NWRSELYKYKVVKIEPLGVAPTKAKRRVVQREKRAFG-LGAVFLGFLGAAGSTMGAASIT	520
27	NWRSELYKYKVVKIEPLGVAPTKAKRRVVQREKRAFG-LGAVFLGFLGAAGSTMGAASIT	526
28	NWRSELYKYKVVKIEPLGVAPTKAKRRVVQREKRAFG-LGAVFLGFLGAAGSTMGAASIT	526
29	NWRSELYKYKVVKIEPLGVAPTKAKRRVVQREKRAFG-LGAVFLGFLGAAGSTMGAASIT	526
30	NWRSELYKYKVVKIEPLGVAPTKAKRRVVQREKRAFG-LGAVFLGFLGAAGSTMGAASIT	526
31	NWRSELYKYKVVKIEPLGVAPTKAKRRVVQREKRAFG-LGAVFLGFLGAAGSTMGAASIT	526
32	NWRSELYKYKVVKIEPLGVAPTKAKRRVVQREKRAFG-LGAVFLGFLGAAGSTMGAASIT	526
33	NWRSELYKYKVVKIEPLGVAPTKAKRRVVQREKRAFG-LGAVFLGFLGAAGSTMGAASIT	526
34	---SELYKYKVVKIEPLGVAPTKAKRRVVQREKRAFG-LGAVFLGFLGAAGSTMGAASIT	518
35	NWRSELYKYKVVKIEPLGVAPTKAKRRVVQREKRAFG-LGAVFLGFLGAAGSTMGAASIT	526
36	NWRSELYKYKVVKIEPLGVAPTKAKRRVVQREKRAFG-LGAVFLGFLGAAGSTMGAASIT	526
37	NWRSELYKYKVVKIEPLGVAPTRAKRRVVQREKRAFG-LGAVFLGFLGAAGSTMGAASIT	526
38	NWRSELYKYKVVKIEPLGIAPTKAKRRVVQREKRAFG-LGAVFLGFLGAAGSTMGAASIT	526
39	NWRSELYKYKVVKIEPLGVAPTKAKRRVVQREK-AFG-LGAVFLGFLGAAGSTMGAASIT	525
40	NWRSELYKYKVVKIEPLGVAPTKAKRRVVQREKRAFG-LGAVFLGFLGAAGSTMGAASIT	526
41	NWRSELYKYKVVKIEPLGVAPTKAKRRVVQREKRAFG-LGAVFLGFLGAAGSTMGAASIT	526
42	NWRSELYKYKVVKIEPLGVAPTKAKRRVVQREKRAFG-LGAVFLGFLGAAGSTMGAASIT	526
43	NWRSELYKYKVVKIEPLGVAPTKAKRRVVQREKRAFG-LGAVFLGFLGAAGSTMGAASIT	526
44	NWRSELYKYKVVKIEPLGVAPTKAKRRVVQREKRAFG-LGAVFLGFLGAAGSTMGAASIT	526
45	NWRSELYKYKVVKIEPLGVAPTKAKRRVVQREKRAFG-LGAVFLGFLGAAGSTMGAASIT	526
46	NWRSELYKYKVVKIEPLGVAPTKAKRRVVQREKRAFG-LGAVFLGFLGAAGSTMGAASIT	525

	**LTVQARQLLSGIVQQQNNLLRAIEAQQHLLQLTVWGIKQLQARVLAVERYLKDQQLLGIW**	
1	LTVQARQLLSGIVQQQNNLLRAIEAQQHLLQLTVWGIKQLQARVLAVERYLKDQQLLGIW	586
2	LTVQARQLLSGIVQQQNNLLRAIEAQQHLLQLTVWGIKQLQARVLAVERYLKDQQLLGIW	582
3	LTVQARQLLSGIVQQQNNLLRAIEAQQHLLQLTVWGIKQLQARVLAVERYLKDQQLLGIW	586
4	LTVQARQLLSGIVQQQNNLLRAIEAQQHLLQLTVWGIKQLQARVLAVERYLKDQQLLGIW	586
5	LTVQARQLLSGIVQQQNNLLRAIEAQQHLLQLTVWGIKQLQARVLAVERYLKDQQLLGIW	586
6	LTVQARQLLSGIVQQQNNLLRAIEAQQHLLQLTVWGIKQLQARVLAVERYLKDQQLLGIW	586
7	LTVQARQLLSGIVQQQNNLLRAIEAQQHLLQLTVWGIKQLQARVLAVERYLKDQQLLGIW	586
8	LTVQARQLLSGIVQQQNNLLRAIEAQQHLLQLTVWGIKQLQARVLAVERYLKDQQLLGIW	586
9	LTVQARQLLSGIVQQQNNLLRAIEAQQHLLQLTVWGIKQLQARVLAVERYLKDQQLLGIW	581
10	LTVQARQLLSGIVQQQNNLLRAIEAQQHLLQLTVWGIKQLQARVLAVERYLKDQQLLGIW	586
11	LTVQARQLLSGIVQQQNNLLRAIEAQQHLLQLTVWGIKQLQARVLAVERYLKDQQLLGIW	586
12	LTVQARQLLSGIVQQQNNLLRAIEAQQHLLQLTVWGIKQLQARVLAVERYLKDQQLLGIW	586
13	LTVQARQLLSGIVQQQNNLLRAIEAQQHLLQLTVWGIKQLQARVLAVERYLKDQQLLGIW	586
14	LTVQARQLLSGIVQQQNNLLRAIEAQQHLLQLTVWGIKQLQARVLAVERYLKDQQLLGIW	586
15	LTVQARQLLSGIVQQQNNLLRAIEAQQHLLQLTVWGIKQLQARVLAVERYLKDQQLLGIW	586
16	LTVQARQLLSGIVQQQNNLLRAIEAQQHLLQLTVWGIKQLQARVLAVERYLKDQQLLGIW	586
17	LTVQARQLLSGIVQQQNNLLRAIEAQQHLLQLTVWGIKQLQARVLAVERYLKDQQLLGIW	586
18	LTVQARQLLSGIVQQQNNLLRAIEAQQHLLQLTVWGIKQLQARVLAVERYLKDQQLLGIW	586
19	LTVQARQLLSGIVQQQNNLLRAIEAQQHLLQLTVWGIKQLQARVLAVERYLKDQQLLGIW	585
20	LTVQARQLLSGIVQQQNNLLRAIEAQQHLLQLTVWGIKQLQARVLAVERYLKDQQLLGIW	586
21	LTVQARQLLSGIVQQQNNLLRAIEAQQHLLQLTVWGIKQLQARVLAVERYLKDQQLLGIW	577
22	LTVQARQLLSGIVQQQNNLLRAIEAQQHLLQLTVWGIKQLQARVLAVERYLKDQQLLGIW	586
23	LTVQARQLLSGIVQQQNNLLRAIEAQQHLLQLTVWGIKQLQARVLAVERYLKDQQLLGIW	586
24	LTVQARQLLSGIVQQQNNLLRAIEAQQHLLQLTVWGIKQLQARVLAVERYLKDQQLLGIW	586
25	LTVQARQLLSGIVQQQNNLLRAIEAQQHLLQLTVWGIKQLQARVLAVERYLKDQQLLGIW	586
26	LTVQARQLLSGIVQQQNNLLRAIEAQQHLLQLTVWGIKQLQARVLAVERYLKDQQLLGIW	580
27	LTVQARQLLSGIVQQQNNLLRAIEAQQHLLQLTVWGIKQLQARVLAVERYLKDQQLLGIW	586
28	LTVQARQLLSGIVQQQNNLLRAIEAQQHLLQLTVWGIKQLQARVLAVERYLKDQQLLGIW	586
29	LTVQARQLLSGIVQQQNNLLRAIEAQQHLLQLTVWGIKQLQARVLAVERYLKDQQLLGIW	586
30	LTVQARQLLSGIVQQQNNLLRAIEAQQHLLQLTVWGIKQLQARVLAVERYLKDQQLLGIW	586
31	LTVQARQLLSGIVQQQNNLLRAIEAQQHLLQLTVWGIKQLQARVLAVERYLKDQQLLGIW	586
32	LTVQARQLLSGIVQQQNNLLRAIEAQQHLLQLTVWGIKQLQARVLAVERYLKDQQLLGIW	586
33	LTVQARQLLSGIVQQQNNLLRAIEAQQHLLQLTVWGIKQLQARVLAVERYLKDQQLLGIW	586
34	LTVQARQLLSGIVQQQNNLLRAIEAQQHLLQLTVWGIKQLQARVLAVERYLKDQQLLGIW	578
35	LTVQARQLLSGIVQQQNNLLRAIEAQQHLLQLTVWGIKQLQARVLAVERYLKDQQLLGIW	586
36	LTVQARQLLSGIVQQQNNLLRAIEAQQHLLQLTVWGIKQLQARVLAVERYLKDQQLLGIW	586
37	LTVQARQLLSGIVQQQNNLLRAIEAQQHLLQLTVWGIKQLQARVLAVERYLKDQQLLGIW	586
38	LTVQARQLLSGIVQQQNNLLRAIEAQQHLLQLTVWGIKQLQARVLAVERYLKDQQLLGIW	586
39	LTVQARQLLSGIVQQQNNLLRAIEAQQHLLQLTVWGIKQLQARVLAVERYLKDQQLLGIW	585
40	LTVQARQLLSGIVQQQNNLLRAIEAQQHLLQLTVWGIKQLQARVLAVERYLKDQQLLGIW	586
41	LTVQARQLLSGIVQQQNNLLRAIEAQQHLLQLTVWGIKQLQARVLAVERYLKDQQLLGIW	586
42	LTVQARQLLSGIVQQQNNLLRAIEAQQHLLQLTVWGIKQLQARVLAVERYLKDQQLLGIW	586
43	LTVQARQLLSGIVQQQNNLLRAIEAQQHLLQLTVWGIKQLQARVLAVERYLKDQQLLGIW	586
44	LTVQARQLLSGIVQQQNNLLRAIEAQQHLLQLTVWGIKQLQARVLAVERYLKDQQLLGIW	586
45	LTVQARQLLSGIVQQQNNLLRAIEAQQHLLQLTVWGIKQLQARVLAVERYLKDQQLLGIW	586
46	LTVQARQLLSGIVQQQNNLLRAIEAQQHLLQLTVWGIKQLQARVLAVERYLKDQQLLGIW	585

GCSGKLICTTTVPWNTSWSNKSLEQIWDNMTWMEWEREIDNYTGYIYQLIEESQNQQEKN

1	GCSGKLICTTTVPWNTSWSNKSLEQIWDNMTWMEWEREIDNYTGYIYQLIEESQNQQEKN	646
2	GCSGKLICPTTVPWNTSWSNKSLEQIWDNMTWMEWEREIDNYTGYIYQLIEESQNQQEKN	642
3	GCSGKLICPTTVPWNTSWSNKSLEQIWDNMTWMEWEREIDNYTGYIYQLIEESQNQQEKN	646
4	GCSGKLICTTTVPWNTSWSNKSLEQIWDNMTWMEWEREIDNYTGYIYQLIEESQNQQEKN	646
5	GCSGKLICTTTVPWNTSWSNKSLEQIWDNMTWREWEREIDNYTGYIYQLIEESQNQQEKN	646
6	GCSGKLICTTTVPWNTSWSNKSLEQIWDNMTWMEWEREIDNYTGYIYQLIEESQNQQEKN	646
7	GCSGKLICTTTVPWNTSWSNKSLEQIWDNMTWMEWEREIDNYTGYIYQLIEESQNQQEKN	646
8	GCSGKLICTTTVPWNTSWSNKSLEQIWDNMTWMEWEREIDNYTGYIYQLIEESQNQQEKN	646
9	GCSGKLICPTTVPWNTSWSNKSLEQIWDNMTWMEWEREIDNYTGYIYQLIEESQNQQEKN	641
10	GCSGKLICTTTVPWNTSWSNKSLEQIWDNMTWMEWEREIDNYTGYIYQLIEESQNQQEKN	646
11	GCSGKLICTTTVPWNTSWSNKSLEQIWDNMTWMEWEREIDNYTGYIYQLIEESQNQQEKN	646
12	GCSGKLICTTTVPWNTSWSNKSLEQIWDNMTWMEWEREIDNYTGYIYQLIEESQNQQEKN	646
13	GCSGKLICTTTVPWNTSWSNKSLEQIWDNMTWMEWEREIDNYTGYIYQLIEESQNQQEKN	646
14	GCSGKLICTTTVPWNTSWSNKSLEQIWDNMTWMEWEREIDNYTGYIYQLIEESQNQQEKN	646
15	GCSGKLICTTTVPWNTSWSNKSLEQIWDNMTWREWEREIDNYTGYIYQLIEESQNQQEKN	646
16	GCSGKLICTTTVPWNTSWSNKSLEQIWDNMTWMEWEREIDNYTGYIYQLIEESQNQQEKN	646
17	GCSGKLICTTTVPWNTSWSNKSLEQIWDNMTWMEWEREIDNYTGYIYQLIEESQNQQEKN	646
18	GCSGKLICPTTVPWNTSWSNKSLEQIWDNMTWMEWEREIDNYTGYIYQLIEESQNQQEKN	646
19	GCSGKLICPTTVPWNTSWSNKSLEQIWDNMTWMEWEREIDNYTGYIYQLIEESQNQQEKN	645
20	GCSGKLICPTTVPWNTSWSNKSLEQIWDNMTWMEWEREIDNYTGYIYQLIEESQNQQEKN	646
21	GCSGKLICTTTVPWNTSWSNKSLEQIWDNMTWMEWEREIDNYTGYIYQLIEESQNQQEKN	637
22	GCSGKLICTTTVPWNTSWSNKSLEQIWDNMTWMEWEREIDNYTGYIYQLIEESQNQQEKN	646
23	GCSGKLICTTTVPWNTSWSNKSLEQIWDNMTWMEWEREIDNYTGYIYQLIEESQNQQEKN	646
24	GCSGKLICTTTVPWNTSWSNKSLEQIWDNMTWMEWEREIDNYTGYIYQLIEESQNQQEKN	646
25	GCSGKLICTTTVPWNTSWSNKSLEQIWDNMTWMEWEREIDNYTGYIYQLIEESQNQQEKN	646
26	GCSGKLICTTTVPWNTSWSNKSLEQIWDNMTWMEWEREIDNYTGYIYQLIEESQNQQEKN	640
27	GCSGKLICPTTVPWNTSWSNKSLEQIWDNMTWMEWEREIDNYTGYIYQLIEELQNQQEKN	646
28	GCSGKLICTTTVPWNTSWSNKSLEQIWDNMTWMEWEREIDNYTGYIYQLIEESQNQQEKN	646
29	GCSGKLICPTTVPWNTSWSNKSLEQIWDNMTWMEWEREIDNYTGYIYQLIEESQNQQEKN	646
30	GCSGKLICPTTVPWNTSWSNKSLEQIWDNMTWMEWEREIDNYTGYIYQLIEESQNQQEKN	646
31	GCSGKLICPTTVPWNTSWSNKSLEQIWDNMTWMEWEREIDNYTGYIYQLIEESQNQQEKN	646
32	GCSGKLICPTTVPWNTSWSNKSLEQIWDNMTWMEWEREIDNYTGYIYQLIEESQNQQEKN	646
33	GCSGKLICPTTVPWNTSWSNKSLEQIWDNMTWMEWEREIDNYTGYIYQLIEESQNQQEKN	646
34	GCSGKLICTTTVPWNTSWSNKSLEQIWDNMTWMEWEREIDNYTGYIYQLIEESQNQQEKN	638
35	GCSGKLICTTTVPWNTSWSNKSLEQIWDNMTWMEWEREIDNYTGYIYQLIEESQNQQEKN	646
36	GCSGKLICTTTVPWNTSWSNKSLEQIWDNMTWMEWEREIDNYTGYIYQLIEESQNQQEKN	646
37	GCSGKLICTTTVPWNTSWSNKSLEQIWDNMTWMEWEREIDNYTGYIYQLIEESQNQQEKN	646
38	GCSGKLICTTTVPWNTSWSNKSLEQIWDNMTWMEWEREIDNYTGYIYQLIEESQNQQEKN	646
39	GCSGKLICTTTVPWNTSWSNKSLEQIWDNMTWMEWEREIDNYTGYIYQLIEESQNQQEKN	645
40	GCSGKLICTTTVPWNTSWSNKSLEQIWDNMTWMEWEREIDNYTGYIYQLIEESQNQQEKN	646
41	GCSGKLICPTTVPWNTSWSNKSLEQIWDNMTWMEWEREIDNYTGYIYQLIEESQNQQEKN	646
42	GCSGKLICPTTVPWNTSWSNKSLEQIWDNMTWMEWEREIDNYTGYIYQLIEESQNQQEKN	646
43	GCSGKLICTTTVPWNTSWSNKSLEQIWDNMTWMEWEREIDNYTGYIYQLIEESQNQQEKN	646
44	GCSGKLICTTTVPWNTSWSNKSLEQIWDNMTWMEWEREIDNYTGYIYQLIEESQNQQEKN	646
45	GCSGKLICTTTVPWNTSWSNKSLEQIWDNMTWMEWEREIDNYTGYIYQLIEESQNQQEKN	646
46	GCSGKLICTTTVPWNTSWSNKSLEQIWDNMTWMEWEREIDNYTGYIYQLIEESQNQQEKN	645

	EQELLALDKWASLWNWFDITNWLWYIKIFIMIVGGLIGLRIVFTVLSIVNRVRQGYSPLS	
1	EQELLALDKWASLWNWFDITNWLWYIKIFIMIVGGLIGLRIVFTVLSIVNRVRQGYSPLS	706
2	EQELLALDKWASLWNWFDITNWLWYIKIFIMIVGGLIGLRIVFTVLSIVNRVRQGYSPLS	702
3	GQELLALDKWASLWNWFDITNWLWYIKIFIMIVGGLIGLRIVFTVLSIVNRVRQGYSPLS	706
4	EQELLALDKWASLWNWFDITNWLWYIKIFIMIVGGLIGLRIVFTVLSIVNRVRQGYSPLS	706
5	EQELLALDKWAGLWNWFDITNWLWYIKIFIMIVGGLIGLRIVFTVLSIVNRVRQGYSPLS	706
6	EQELLALDKWAGLWNWFDITNWLWYIKIFIMIVGGLIGLRIVFTVLSIVNRVRQGYSPLS	706
7	EQELLALDKWASLWNWFDITNWLWYIKIFIMIVGGLIGLRIVFTVLSIVNRVRQGYSPLS	706
8	EQELLALDKWASLWNWFDITNWLWYIKIFIMIVGGLIGLRIVFTVLSIVNRVRQGYSPLS	706
9	EQELLALDKWASLWNWFDITNWLWYIKIFIMIVGGLIGLRIVFTVLSIVNRVRQGYSPLS	701
10	EQELLALDKWAGLWNWFDITNWLWYIKIFIMIVGGLIGLRIVFTVLSIVNRVRQGYSPLS	706
11	EQELLALDKWASLWNWFDITNWLWYIKIFIMIVGGLIGLRIVFTVLSIVNRVRQGYSPLS	706
12	EQELLALDKWAGLWNWFDITNWLWYIKIFIMIVGGLIGLRIVFTVLSIVNRVRQGYSPLS	706
13	EQELLALDKWASLWNWFDITNWLWYIKIFIMIVGGLIGLRIVFTVLSIVNRVRQGYSPLS	706
14	EQELLALDKWASLWNWFDITNWLWYIKIFIMIVGGLIGLRIVFTVLSIVNRVRQGYSPLS	706
15	EQELLALDKWASLWNWFDITNWLWYIKIFIMIVGGLIGLRIVFTVLSIVNRVRQGYSPLS	706
16	EQELLALDKWASLWNWFDITNWLWYIKIFIMIVGGLIGLRIVFTVLSIVNRVRQGYSPLS	706
17	EQELLALDKWASLWNWFDITNWLWYIKIFIMIVGGLIGLRIVFTVLSIVNRVRQGYSPLS	706
18	EQELLALDKWASLWNWFDITNWLWYIKIFIMIVGGLIGLRIVFTVLSIVNRVRQGYSPLS	706
19	EQELLALDKWASLWNWFDITNWLWYIKIFIMIVGGLIGLRIVFTVLSIVNRVRQGYSPLS	705
20	EQELLALDKWASLWNWFDITNWLWYIKIFIMIVGGLIGLRIVFTVLSIVNRVRQGYSPLS	706
21	EQELLALDKWASLWNWFDITNWLWYIKIFIMIVGGLIGLRIVFTVLSIVNRVRQGYSPLS	697
22	EQELLALDKWASLWNWFDITNWLWYIKIFIMIVGGLIGLRIVFTVLSIVNRVRQGYSPLS	706
23	EQELLALDKWAGLWNWFDITNWLWYIKIFIMIVGGLIGLRIVFTVLSIVNRVRQGYSPLS	706
24	EQELLALDKWAGLWNWFDITNWLWYIKIFIMIVGGLIGLRIVFTVLSIVNRVRQGYSPLS	706
25	EQELLALDKWASLWNWFDITNWLWYIKIFIMIVGGLIGLRIVFTVLSIVNRVRQGYSPLS	706
26	EQELLALDKWASLWNWFDITNWLWYIKIFIMIVGGLIGLRIVFTVLSIVNRVRQGYSPLS	700
27	EQELLALDKWASLWNWFDITNWLWYIKIFIMIVGGLIGLRIVFTVLSIVNRVRQGYSPLS	706
28	EQELLALDKWASLWNWFDITNWLWYIKIFIMIVGGLIGLRIVFTVLSIVNRVRQGYSPLS	706
29	EQELLALDKWASLWNWFDITNWLWYIKIFIMIVGGLIGLRIVFTVLSIVNRVRQGYSPLS	706
30	EQELLALDKWASLWNWFDITNWLWYIKIFIMIVGGLIGLRIVFTVLSIVNRVRQGYSPLS	706
31	EQELLALDKWASLWNWFDITNWLWYIKIFIMIVGGLIGLRIVFTVLSIVNRVRQGYSPLS	706
32	EQELLALDKWASLWNWFDITNWLWYIKIFIMIVGGLIGLRIVFTVLSIVNRVRQGYSPLS	706
33	EQELLALDKWASLWNWFDITNWLWYIKIFIMIVGGLIGLRIVFTVLSIVNRVRQGYSPLS	706
34	EQELLALDKWASLWNWFDITNWLWYIKIFIMIVGGLIGLRIVFTVLSIVNRVRQGYSPLS	698
35	EQELLALDKWASLWNWFDITNWLWYIKIFIMIVGGLIGLRIVFTVLSIVNRVRQGYSPLS	706
36	EQELLALDKWASLWNWFDITNWLWYIKIFIMIVGGLIGLRIVFTVLSIVNRVRQGYSPLS	706
37	EQELLALDEWASLWNWFDITNWLWYIKIFIMIVGGLIGLRIVFTVLSIVNRVRQGYSPLS	706
38	EQELLALDKWASLWNWFDITNWLWYIKIFIMIVGGLIGLRIVFTVLSIVNRVRQGYSPLS	706
39	EQELLALDKWASLWNWFDITNWLWYIKIFIMIVGGLIGLRIVFTVLSIVNRVRQGYSPLS	705
40	EQELLALDKWASLWNWFDITNWLWYIKIFIMIVGGLIGLRIVFTVLSIVNRVRQGYSPLS	706
41	EQELLALDKWASLWNWFDITNWLWYIKIFIMIVG---GLRIVFTVLSIVNRVRQGYSPLS	703
42	EQELLALDKWASL-NWFDITNWLWYIKIFIMIVGGLIGLRIVFTVLSIVNRVRQGYSPLS	705
43	EQELLALDKWASLWNWFDITNWLWYIKIFIMIVGGLIGLRIVFTVLSIVNRVRQGYSPLS	706
44	EQELLALDKWASLWNWFDITNWLWYIKIFIMIVGGLIGLRIVFTVLSIVNRVRQGYSPLS	706
45	EQELLALDKWAGLWNWFDITNWLWYIKIFIMIVGGLIGLRIVFTVLSIVNRVRQGYSPLS	706
46	EQELLALDKWASLWNWFDITNWLWYIKIFIMIVGGLIGLRIVFTVLSIVNRVRQGYSPLS	705

	FQTHLPAQRGPDRPEGIGEEGGERDRDRSDPLVNGFLTLIWSDLRSLCLFSYHRLRDLLL	
1	FQTHLPAQRGPDRPEGIGEEGGERDRDRSDPLVNGFLALIWSDLRSLCLFSYHRLRDLLL	766
2	FQTHLPAQRGPDRPEGIGEEGGERDRDRSDPLVNGFLTLIWSDLRSLCLFSYHRLRDLLL	762
3	FQTHLPAQRGPDRPEGIGEEGGERDRDRSDPLVNGFLTLIWSDLRSLCLFSYHRLRDLLL	766
4	FQTHLPAQRGPDRPEGIGEEGGERDRDRSDPLVNGFLTLIWSDLRSLCLFSYHRLRDLLL	766
5	FQTHLPAQRGPDRPEGIGEEGGERDRDRSDPLVNGFLTLIWSDLRSLCLFSYHRLRDLLL	766
6	FQTHLPAQRGPDRPEGIGEEGGERDRDRSDPLVNGFLTLIWSDLRSLCLFSYHRLRDLLL	766
7	FQTHLPAQRGPDRPEGIGEEGGERDRDRSDPLVNGFLTLIWSDLRSLCLFSYHRLRDLLL	766
8	FQTHLPAQRGPDRPEGIGEEGGERDRDRSDPLVNGFLTLIWSDLRSLCLFSYHRLRDLLL	766
9	FQTHLPAQRGPDRPEGIGEEGGERDRDRSDPLVNGFLTLIWSDLRSLCLFSYHRLRDLLL	761
10	FQTHLPAQRGPDRPEGIGEEGGERDRDRSDPLVNGFLTLIWSDLRSLCLFSYHRLRDLLL	766
11	FQTHLPAQRGPDRPEGIGEEGGERDRDRSDPLVNGFLTLIWSDLRSLCLFSYHRLRDLLL	766
12	FQTHLPAQRGPDRPEGIGEEGGERDRDRSDPLVNGFLTLIWSDLRSLCLFSYHRLRDLLL	766
13	FQTHLPAQRGPDRPEGIGEEGGERDRDRSDPLVNGFLTLIWSDLRSLCLFSYHRLRDLLL	766
14	FQTHLPAQRGPDRPEGIGEEGGERDRDRSDPLVNGFLTLIWSDLRSLCLFSYHRLRDLLL	766
15	FQTHLPAQRGPDRPEGIGEEGGERDRDRSDPLVNGFLTLIWSDLRSLCLFSYHRLRDLLL	766
16	FQTHLPAQRGPDRPEGIGEEGGERDRDRSDPLVNGFLTLIWSDLRSLCLFSYHRLRDLLL	766
17	FQTHLPAQRGPDRPEGIGEEGGERDRDRSDPLVNGFLTLIWSDLRSLCLFSYHRLRDLLL	766
18	FQTHLPAQRGPDRPEGIGEEGGERDRDRSDPLVNGFLTLIWSDLRSLCLFSYHRLRDLLL	766
19	FQTHLPAQRGPDRPEGIGEEGGERDRDRSDPLVNGFLTLIWSDLRSLCLFSYHRLRDLLL	765
20	FQTHLPAQRGPDRPEGIGEEGGERDRDRSDPLVNGFLTLIWSDLRSLCLFSYHRLRDLLL	766
21	FQTHLPAQRGPDRPEGIGEEGGERDRDRSDPLVNGFLTLIWSDLRSLCLFSYHRLRDLLL	757
22	FQTHLPAQRGPDRPEGIGEEGGERDRDRSDPLVNGFLTLIWSDLRSLCLFSYHRLRDLLL	766
23	FQTHLPAQRGPDRPEGIGEEGGERDRDRSDPLVNGFLTLIWSDLRSLCLFSYHRLRDLLL	766
24	FQTHLPAQRGPDRPEGIGEEGGERDRDRSDPLVNGFLTLIWSDLRSLCLFSYHRLRDLLL	766
25	FQTHLPAQRGPDRPEGIGEEGGERDRDRSDPLVNGFLTLIWSDLRSLCLFSYHRLRDLLL	766
26	FQTHLPAQRGPDRPEGIGEEGGERDRDRSDPLVNGFLTLIWSDLRSLCLFSYHRLRDLLL	760
27	FQTHLPAQRGPDRPEGIGEEGGERDRDRSDPLVNGFLTLIWSDLRSLCLLSYHRLRDLLL	766
28	FQTHLPAQRGPDRPEGIGEEGGERDRDRSDPLVNGFLTLIWSDLRSLCLFSYHRLRDLLL	766
29	FQTHLPAQRGPDRPEGIGEEGGERDRDRSDPLVNGFLTLIWSDLRSLCLFSYHRLRDLLL	766
30	FQTHLPAQRGPDRPEGIGEEGGERDRDRSDPLVNGFLTLIWSDLRSLCLFSYHRLRDLLL	766
31	FQTHLPAQRGPDRPEGIGEEGGERDRDRSDPLVNGFLTLIWSDLRSLCLFSYHRLRDLLL	766
32	FQTHLPAQRGPDRPEGIGEEGGERDRDRSDPLVNGFLTLIWSDLRSLCLFSYHRLRDLLL	766
33	FQTHLPAQRGPDRPEGIGEEGGERDRDRSDPLVNGFLTLIWSDLRSLCLFSYHRLRDLLL	766
34	FQTHLPAQRGPDRPEGIGEEGGERDRDRSDPLVNGFLTLIWSDLRSLCLFSYHRLRDLLL	758
35	FQTHLPAQRGPDRPEGIGEEGGERDRDRSDPLVNGFLTLIWSDLRSLCLFSYHRLRDLLL	766
36	FQTHLPAQRGPDRPEGIGEEGGERDRDRSDPLVNGFLTLIWSDLRSLCLFSYHRLRDLLL	766
37	FQTHLPAQRGPDRPEGIGEEGGERDRDRSDPLVNGFLTLIWSDLRSLCLFSYHRLRDLLL	766
38	FQTHLPAQRGPDRPEGIGEEGGERDRDRSDPLVNGFLTLIWSDLRSLCLFSYHRLRDLLL	766
39	FQTHLPAQRGPDRPEGIGEEGGERDRDRSDPLVNGFLTLIWSDLRSLCLFSYHRLRDLLL	765
40	FQTHLPAQRGPDRPEGIGEEGGERDRDRSDPLVNGFLTLIWSDLRSLCLFSYHRLRDLLL	766
41	FQTHLPAQRGPDRPEGIGEEGGERDRDRSDPLVNGFLTLIWSDLRSLCLFSYHRLRDLLL	763
42	FQTHLPAQRGPDRPEGIGEEGGERDRDRSDPLVNGFLTLIWSDLRSLCLFSYHRLRDLLL	765
43	FQTHLPAQRGPDRPEGIGEEGGERDRDRSDPLVNGFLTLIWSDLRSLCLFSYHRLRDLLL	766
44	FQTHLPAQRGPDRPEGIGEEGGERDRDRSDPLVNGFLTLIWSDLRSLCLFSYHRLRDLLL	766
45	FQTHLPAQRGPDRPEGIGEEGGERDRDRSDPLVNGFLTLIWSDLRSLCLFSYHRLRDLLL	766
46	FQTHLPAQRGPDRPEGIGEEGGERDRDRSDPLVNGFLTLIWSDLRSLCLFSYHRLRDLLL	765

	IVTRIVELLGR-------RGWEVLKYWWNLLQYWSQELKNSAVSLLNATATAVAEGTDRV	
1	IVTRIVELLGR-------RGWEVLKYWWNLLQYWSQELKNSAVSLLNATAIAVAEGTDRV	819
2	I----VELLGR-------RGWEVLKYWWNLLQYWSQELKNSAVSLLNATAIAVAEGTDRV	811
3	IVTRIVELLGR-------RGWEVLKYWWNLLQYWSQELKNSAVSLLNATAIAVAEGTDRV	819
4	IVTRIVELLGR-------RGWEVLKYWWNLLQYWSQELKNSAVSLLNATATAVAEGTDRV	819
5	IVTRIVELLGR-------RGWEVLKYWWNLLQYWSQELKNSAVSLLNATATAVAEGTDRV	819
6	IVTRIVELLGR-------RGWEVLKYWWNLLQYWSQELKNSAVSLLNATATAVAEGTDRV	819
7	IVTRIVELLGR-------RGWEVLKYWWNLLQYWSQELKNSAVSLLNATATAVAEGTDRV	819
8	IVTRIVELLGR-------RGWEVLKYWWNLLQYWSQELKNSAVSLLNATATAVAEGTDRV	819
9	IVTRIVELLGR-------RGWEVLKYWWNLLQYWSQELKNSAVSLLNATAIAVAEGTDRV	814
10	IVTRIVELLGR-------RGWEVLKYWWNLLQYWSQELKNSAVSLLNATATAVAEGTDRV	819
11	IVTRIVELLGR-------RGWEVLKYWWNLLQYWSQELKNSAVSLLNATATAVAEGTDRV	819
12	IVTRIVELLGR-------RGWEVLKYWWNLLQYWSQELKNSAVSLLNATATAVAEGTDRV	819
13	IVTRIVELLGR-------RGWEVLKYWWNLLQYWSQELKNSAVSLLNATAIAVAEGTDRV	819
14	IVTRIVELLGR-------RGWEVLKYWWNLLQYWSQELKNSAVSLLNATATAVAEGTDRV	819
15	IVTRIVELLGR-------RGWEVLKYWWNLLQYWSQELKNSAVSLLNATATAVAEGTDRV	819
16	IVTRIVELLGR-------RGWEVLKYWWNLLQYWSQELKNSAVSLLNATATAVAEGTDRV	819
17	IVTRIVELLGR-------RGWEVLKYWWNLLQYWSQELKNSAVSLLNATATAVAEGTDRV	819
18	IVTRIVELLGR-------RGWEVLKYWWNLLQYWSQELKNSAVSLLNATAIAVAEGTDRV	819
19	IVTRIVELLGR-------RGWEVLKYWWNLLQYWSQELKNSAVSLLNATAIAVAEGTDRV	818
20	IVTRIVELLGR-------RGWEVLKYWWNLLQYWSQELKNSAVSLLNATAIAVAEGTDRV	819
21	IVTRIVELLGR-------RGWEVLKYWWNLLQYWSQELKNSAVSLLNATATAVAEGTDRV	810
22	IVTRIVELLGR-------RGWEVLKYWWNLLQYWSQELKNSAVSLLNATATAVAEGTDRV	819
23	IVTRIVELLGR-------RGWEVLKYWWNLLQYWSQELKNSAVSLLNATATAVAEGTDRV	819
24	IVTRIVELLGR-------RGWEVLKYWWNLLQYWSQELKNSAVSLLNATATAVAEGTDRV	819
25	IVTRIVELLGR-------RGWEVLKYWWNLLQYWSQELKNSAVSLLNATATAVAEGTDRV	819
26	IVTRIVELLGR-------RGWEVLKYWWNLLQYWSQELKNSAVSLLNATATAVAEGTDRV	813
27	IVTRIVELLGR-------RGWEVLKYWWNLLQYWSQELKNSAVSLLNATAIAVAEGTDRV	819
28	IVTRIVELLGR-------RGWEVLKYWWNLLQYWSQELKNSAVSLLNATATAVAEGTDRV	819
29	IVTRIVELLGR-------RGWEVLKYWWNLLQYWSQELKNSAVSLLNATAIAVAEGTDRV	819
30	IVTRIVELLGR-------RGWEVLKYWWNLLQYWSQELKNSAVSLLNATAIAVAEGTDRV	819
31	IVTRIVELLGR-------RGWEVLKYWWNLLQYWSQELKNSAVSLLNATAIAVAEGTDRV	819
32	IATRIVELLGR-------RGWEVLKYWWNLLQYWSQELKNSAVSLLNATAIAVAEGTDRV	819
33	IVTRIVELLGR-------RGWEVLKYWWNLLQYWSQELKNSAVSLLNATAIAVAEGTDRV	819
34	IVTRIVELLGR-------RGWEVLKYWWNLLQYWSQELKNSAVSLLNATATAVAEGTDRV	811
35	IVTRIVELLGR-------RGWEVLKYWWNLLQYWSQELKNSAVSLLNATATAVAEGTDRV	819
36	IVTRIVELLGR-------RGWEVLKYWWNLLQYWSQELKNSAVSLLNATATAVAEGTDRV	819
37	IVTRIVELLGR-------RGWEVLKYWWNLLQYWSQELKNSAVSLLNATAIAVAEGTDRV	819
38	IVTRIVELLGR-------RGWEVLKYWWNLLQYWSQELKNSAVSLLNATATAVAEGTDRV	819
39	IVTRIVELLGR-------RGWEVLKYWWNLLQYWSQELKNSAVSLLNATATAVAEGTDRV	818
40	IVTRIVELLGR-------RGWEVLKYWWNLLQYWSQELKNSAVSLLNATATAVAEGTDRV	819
41	I----VELLGR-------RGWEVLKYWWNLLQYWSQELKNSAVSLLNATAIAVAEGTDRV	812
42	IVTRIVELLGR-------RGWEVLKYWWNLLQYWSQELKNSAVSLLNATAIAVAEGTDRV	818
43	IVTRIVELLGR-------RGWEVLKYWWNLLQYWSQELKNSAVSLLNATATAVAEGTDRV	819
44	IVTRIVELLGR-------RGWEVLKYWWNLLQYWSQELKNSAVSLLNATATAVAEGTDRV	819
45	IVTRIVELLGR-------RGWEVLKYWWNLLQYWSQELKNSAVSLLNATATAVAEGTDRV	819
46	IVTRIVELLGR-------RGWEVLKYWWNLLQYWSQELKNSAVSLLNATATAVAEGTDRV	818

	IEVVQRACRAILHIPRRIRQGLERALL	
1	IEVVQRACRAILHIPRRIRQGLERALL	846
2	IEVVQRACRAILHIPRRIRQGLERALL	838
3	IEVVQRACRAILHIPRRIRQGLERALL	846
4	IEVVQRACRAILHIPRRIRQGLERALL	846
5	IEVVQRACRAILHIPRRIRQGLERALL	846
6	IEVVQRACRAILHIPRRIRQGLERALL	846
7	IEVVQRACRAILHIPRRIRQGLERALL	846
8	IEVVQRACRAILHIPRKIRQGLERALL	846
9	IEVVQRACRAILHIPRRIRQGLERALL	841
10	IEVVQRACRAILHIPRRIRQGLERALL	846
11	IEVVQRACRAILHIPRRIRQGLERALL	846
12	IEVVQRACRAILHIPRRIRQGLERALL	846
13	IEVVQGACRAILHIPRRIRQGLERALL	846
14	IEVVQRACRAILHIPRRIRQGLERALL	846
15	IEVVQRACRAILHIPRRIRQGLERALL	846
16	IEVVQRACRAILHIPRRIRQGLERALL	846
17	IEVVQRACRAILHIPRRIRQGLERALL	846
18	IEVVQRACRAILHIPRRIRQGLERALL	846
19	IEVVQRACRAILHIPRRIRQGLERALL	845
20	IEVVQRACRAILHIPRRIRQGLERALL	846
21	IEVVQRACRAILHIPRRIRQGLERALL	837
22	IEVVQRACRAILHIPRRIRQGLERALL	846
23	IEVVQRACRAILHIPRRIRQGLERALL	846
24	IEVVQRACRAILHIPRRIRQGLERALL	846
25	IEVVQRACRAILHIPRRIRQGLERALL	846
26	IEVVQRACRAILHIPRRIRQGLERALL	840
27	IEVVQRACRAILHIPRRIRQGLERALL	846
28	IEVVQRACRAILHIPRRIRQGLERALL	846
29	IEVVQRACRAILHIPRRIRQGLERALL	846
30	IEVVQRACRAILHIPRRIRQGLERALL	846
31	IEVVQRACRAILHIPRRIRQGLERALL	846
32	IEVVQRACRAILHIPRRIRQGLERALL	846
33	IEVVQRACRAILHIPRRIRQGLERALL	846
34	IEVVQRACRAILHIPRRIRQGLERALL	838
35	IEVVQRACRAILHIPRRIRQGLERALL	846
36	IEVVQRACRAILHIPRRIRQGLERALL	846
37	IEVVQRACRAIRHIPRRIRQGLERALL	846
38	IEVVQRACRAILHIPRRIRQGLERALL	846
39	IEVVQRACRAILHIPRRIRQGLERALL	845
40	IEVVQRACRAILHIPRRIRQGLERALL	846
41	IEVVQRACRAILHIPRRIRQGLERALL	839
42	IEVVQRACRAILHIPRRIRQGLERALL	845
43	IEVVQRACRAILHIPRRIRQGLERALL	846
44	IEVVQRACRAILHIPRRIRQGLERALL	846
45	IEVVQRACRAILHIPRRIRQGLERALL	846
46	IEVVQRACRAILHIPRRIRQGLERALL	845

Aligned using an external algorithm

Consensus

1. B.US.2006.700010040_C9_4520.EU289193
2. B.US.2008.CH0040_3_d0743_ipe032_2_03.MG900387
3. B.US.2008.CH0040_3_d0743_ipe032_2_04.MG900388
4. B.US.2008.CH0040_3_d0743_ipe032_2_05.MG900389
5. B.US.2008.CH0040_3_d0743_ipe032_2_07.MG900390
6. B.US.2008.CH0040_3_d0743_ipe032_2_08.MG900391
7. B.US.2008.CH0040_3_d0743_ipe032_2_10.MG900392
8. B.US.2008.CH0040_3_d0743_ipe032_2_11.MG900393
9. B.US.2008.CH0040_3_d0743_ipe032_2_12.MG900394
10. B.US.2008.CH0040_3_d0743_ipe032_2_13.MG900395
11. B.US.2008.CH0040_3_d0743_ipe032_2_14.MG900396
12. B.US.2008.CH0040_3_d0743_ipe032_2_15.MG900397
13. B.US.2008.CH0040_3_d0743_ipe032_2_16.MG900398
14. B.US.2008.CH0040_3_d0743_ipe032_2_18.MG900399
15. B.US.2008.CH0040_3_d0743_ipe032_2_19.MG900400
16. B.US.2008.CH0040_3_d0743_ipe032_2_20.MG900401
17. B.US.2008.CH0040_3_d0743_ipe032_2_22.MG900402
18. B.US.2008.CH0040_3_d0743_ipe032_2_25.MG900403
19. B.US.2008.CH0040_3_d0743_ipe032_2_26.MG900404
20. B.US.2008.CH0040_3_d0743_ipe032_2_27.MG900405
21. B.US.2008.CH0040_3_d0743_ipe032_2_28.MG900406
22. B.US.2008.CH0040_3_d0743_ipe032_2_29.MG900407
23. B.US.2008.CH0040_3_d0743_ipe032_2_30.MG900408
24. B.US.2008.CH0040_3_d0743_ipe032_2_32.MG900409
25. B.US.2008.CH0040_3_d0743_ipe032_2_33.MG900410
26. B.US.2008.CH0040_3_d0743_ipe032_3_24.MG900411

	**MRVMGIRKNYQHLWREGILLLGILMICSAADNLWVTVYYGVPVWREATTTLFCASDAKAY**	
1	MRVMGIRKNYQHLWREGILLLGILMICSAADNLWVTVYYGVPVWREATTTLFCASDAKAY	60
2	MRVMGIRKNYQHLWREGILLLGILMICSAADNLWVTVYYGVPVWREATTTLFCASDAKAY	60
3	MRVMGIRKNYQHLWREGILLLGILMICSAADNLWVTVYYGVPVWREATTTLFCASDAKAY	60
4	MRVMGIRKNYQHLWREGILLLGILMICSAADNLWVTVYYGVPVWREATTTLFCASDAKAY	60
5	MRVMGIRKNYQHLWREGILLLGILMICSAADNLWVTVYYGVPVWREATTTLFCASDAKAY	60
6	MRVMGIRKNYQHLWREGILLLGILMICSAADNLWVTVYYGVPVWREATTTLFCASDAKAY	60
7	MRVMGIRKNYQHLWREGILLLGILMICSAADNLWVTVYYGVPVWREATTTLFCASDAKAY	60
8	MRVMGIRKNYQHLWREGILLLGILMICSAADNLWVTVYYGVPVWREATTTLFCASDAKAY	60
9	MRVMGIRKNYQHLWREGILLLGILMICSAADNLWVTVYYGVPVWREATTTLFCASDAKAY	60
10	MRVMGIRKNYQHLWREGILLLGILMICSAADNLWVTVYYGVPVWREATTTLFCASDAKAY	60
11	MRVMGIRKNYQHLWREGILLLGILMICSAADNLWVTVYYGVPVWREATTTLFCASDAKAY	60
12	MRVMGIRKNYQHLWRKGILLLGILMICSAADNLWVTVYYGVPVWREATTTLFCASDAKAY	60
13	MRVMGIRKNYQHLWREGILLLGILMICSAADNLWVTVYYGVPVWREATTTLFCASDAKAY	60
14	MRVMGIRKNYQHLWREGILLLGILMICSAADNLWVTVYYGVPVWREATTTLFCASDAKAY	60
15	MRVMGIRKNYQHLWREGILLLGILMICSAADNLWVTVYYGVPVWREATTTLFCASDAKAY	60
16	MRVMGIRKNYQHLWREGILLLGILMICSAADNLWVTVYYGVPVWREATTTLFCASDAKAY	60
17	MRVMGIRKNYQHLWREGILLLGILMICSAADNLWVTVYYGVPVWREATTTLFCASDAKAY	60
18	MRVMGIRKNYQHLWREGILLLGILMICSAADNLWVTVYYGVPVWREATTTLFCASDAKAY	60
19	MRVMGIRKNYQHLWREGILLLGILMICSAADNLWVTVYYGVPVWREATTTLFCASDAKAY	60
20	MRVMGIRKNYQHLWRKGILLLGILMICSAADNLWVTVYYGVPVWREATTTLFCASDAKAY	60
21	MRVMGIRKNYQHLWREGILLLGILMICSAADNLWVTVYYGVPVWREATTTLFCASDAKAY	60
22	MRVMGIRKNYQHLWREGILLLGILMICSAADNLWVTVYYGVPVWREATTTLFCASDAKAY	60
23	MRVMGIRKNYQHLWREGILLLGILMICSAADNLWVTVYYGVPVWREATTTLFCASDAKAY	60
24	MRVMGIRKNYQHLWREGILLLGILMICSAADNLWVTVYYGVPVWREATTTLFCASDAKAY	60
25	MRVMGIRKNYQHLWREGILLLGILMICSAADNLWVTVYYGVPVWREATTTLFCASDAKAY	60
26	MRVMGIRKNYQHLWREGILLLEILMICSAADNLWVTVYYGVPVWREATTTLFCASDAKAY	60

	DTEAHNVWATHACVPTDPNPQEVELKNVTENFNMWENNMVEQMHEDIISLWDQSLKPCVK	
1	DTEAHNVWATHACVPTDPNPQEVELKNVTENFNMWENNMVEQMHEDIISLWDQSLKPCVK	120
2	DTEAHNVWATHACVPTDPNPQEVELKNVTENFNMWENNMVEQMHEDIISLWDQSLKPCVK	120
3	DTEAHNVWATHACVPTDPNPQEVELKNVTENFNMWENNMVEQMHEDIISLWDQSLKPCVK	120
4	DTEAHNVWATHACVPTDPNPQEVELKNVTENFNMWENNMVEQMHEDIISLWDQSLKPCVK	120
5	DTEAHNVWATHACVPTDPNPQEVELKNVTENFNMWENNMVEQMHEDIISLWDQSLKPCVK	120
6	DTEAHNVWATHACVPTDPNPQEVELKNVTENFNMWENNMVEQMHEDIISLWDQSLKPCVK	120
7	GTEAHNVWATHACVPTDPNPQEVELKNVTENFNMWENNMVEQMHEDIISLWDQSLKPCVK	120
8	DTEAHNVWATHACVPTDPNPQEVELKNVTENFNMWKNNMVEQMHEDIISLWDQSLKPCVK	120
9	DTEAHNVWATHACVPTDPNPQEVELKNVTENFNMWENNMVEQMHEDIISLWDQSLKPCVK	120
10	DTEAHNVWATHACVPTDPNPQEVELKNVTENFNMWENNMVEQMHEDIISLWDQSLKPCVK	120
11	DTEAHNVWATHACVPTDPNPQEVELKNVTENFNMWENNMVEQMHEDIISLWDQSLKPCVK	120
12	DTEAHNVWATHACVPTDPNPQEVELKNVTENFNMWENNMVEQMHEDIISLWDQSLKPCVK	120
13	DTEAHNVWATHACVPTDPNPQEVELKNVTENFNMWENNMVEQMHEDIISLWDQSLKPCVK	120
14	DTEAHNVWATHACVPTDPNPQEVELKNVTENFNMWENNMVEQMHEDIISLWDQSLKPCVK	120
15	DTEAHNVWATHACVPTDPNPQEVELKNVTENFNMWENNMVEQMHEDIISLWDQSLKPCVK	120
16	DTEAHNVWATHACVPTDPNPQEVELKNVTENFNMWENNMVEQMHEDIISLWDQSLKPCVK	120
17	DTEAHNVWATHACVPTDPNPQEVELKNVTENFNMWENNMVEQMHEDIISLWDQSLKPCVK	120
18	DTEAHNVWATHACVPTDPNPQEVELKNVTENFNMWENNMVEQMHEDIISLWDQSLKPCVK	120
19	DTEAHNVWATHACVPTDPNPQEVELKNVTENFNMWENNMVEQMHEDIISLWDQSLKPCVK	120
20	DTEAHNVWATHACVPTDPNPQEVELKNVTENFNMWENNMVEQMHEDIISLWDQSLKPCVK	120
21	DTEAHNVWATHACVPTDPNPQEVELKNVTENFNMWENNMVEQMHEDIISLWDQSLKPCVK	120
22	DTEAHNVWATHACVPTDPNPQEVELKNVTENFNMWENNMVEQMHEDIISLWDQSLKPCVK	120
23	DTEAHNVWATHACVPTDPNPQEVELKNVTENFNMWENNMVEQMHEDIISLWDQSLKPCVK	120
24	DTEAHNVWATHACVPTDPNPQEVELKNVTENFNMWKNNMVEQMHEDIISLWDQSLKPCVK	120
25	DTEAHNVWATHACVPTDPNPQEVELKNVTENFNMWENNMVEQMHEDIISLWDQSLKPCVK	120
26	DTEAHNVWATHACVPTDPNPQEVELKNVTENFNMWENNMVEQMHEDIISLWDQSLKPCVK	120

```
    LTPLCVTLNCTDLGNVTTTT-----------------NSNGTLMEKGEVKNCSFKITTDI

1   LTPLCVTLNCTDLGNVTNTT-----------------NSNGEMMEKGEVKNCSFKITTDI  163
2   LTPLCVTLNCTDLGNVTTTTNRNGTNCTDLGNVTTTTNRNGTLMEKGEVKNCSFRITTDI  180
3   LTPLCVTLNCTDLGNVTTTT-----------------NNNGTLMEKGEVKNCSFKITTDT  163
4   LTPLCVTLNCTDLGNVTTTTNRNGTNCTDLGNVTTTTNRNGTLMEKGEVKNCSFRITTDI  180
5   LTPLCVTLNCTDLGNVTTTT-----------------NSNGTLMEKGEVKNCSFKITTDT  163
6   LTPLCVTLNCTDLGNVTTTT-----------------NSNGTLMEKGEVKNCSFKITTDT  163
7   LTPLCVTLNCTDLGNVTTTTNRNGTNCTDLGNVTTTTNRNGTLMEKGEVKNCSFRITTDI  180
8   LTPLCVTLNCTDLGNVTTTT-----------------NSNGTLMEKGEVKNCSFKITTEI  163
9   LTPLCVTLNCTDLGNGTNTT-----------------NSNEKMMEKGEIKNCSFKITTDI  163
10  LTPLCVTLNCTDLGNDTTTT-----------------NSNGTLMEKGEVKNCSFKITTEI  163
11  LTPLCVTLNCTDLGNVTTTTNRNGTNCTDLGNVTTTTNRNGTLMEKGEVKNCSFRITTDI  180
12  LTPLCVTLNCTDLEKGTNTT-----------------NSTEKMMEKGEVKNCSFRITTDI  163
13  LTPLCVTLNCTDLGNVTTTTNRNGTNCTDLGNVTTTTNRNGTLMEKGEVKNCSFRITTDI  180
14  LTPLCVTLNCTDLGNVTTTT-----------------NSNGTLMEKGEVKNCSFKITTDT  163
15  LTPLCVTLNCTDLGNVTTTTNRNGTNCTDLGNVTTTTNRNGTLMEKGEVKNCSFKITTDV  180
16  LTPLCVTLNCTDLGNDTTTT-----------------NSNGTLMEKGEVKNCSFKITTEI  163
17  LTPLCVTLNCTDLGNDTTTT-----------------NSNGTLMEKGEVKNCSFKITTEI  163
18  LTPLCVTLNCTDLGNVTTTTNRNGTNCTDLGNVTTTTNRNGTLMEKGEVKNCSFRITTDI  180
19  LTPLCVTLNCTDLGNVTTTTNRNGTNCTDLGNVTTTTNRNGTLMEKGEVKNCSFKITTDV  180
20  LTPLCVTLNCTDLGNVTTTT-----------------NSNGTLMEKGEVKNCSFKITTDI  163
21  LTPLCVTLNCTDLGNDTTTT-----------------NSNGTLMEKGEVKNCSFKITTEI  163
22  LTPLCVTLNCTDLGNVTTTT-----------------NSNGTLMEKGEVKNCSFKITTDT  163
23  LTPLCVTLNCTDLGNDTTTT-----------------NSNGTLMEKGEVKNCSFKITTEI  163
24  LTPLCVTLNCTDLGNVTTTT-----------------NSNGTLMEKGEVKNCSFKITTEI  163
25  LTPLCVTLNCTDLGNVTTTTNRNGTNCTDLGNVTTTTNRNGTLMEKGEVKNCSFRITTDI  180
26  LTPLCVTLNCTDLGNVTNTT-----------------NSNGGMMEKGEVKNCSFKITADI  163
```

```
    KDRTRKEYALFYKLDVVPIND-------TRYRLVSCNTSVITQACPKVSFEPIPIHYCAP
1   KDRTRKEYALFYKLDVVPIND-------TRYRLVSCNTSVITQACPKVSFEPIPIHYCAP 216
2   KDRTRKEYALFYKLDVVPIND-------TRYRLVSCNTSVITQACPKVSFEPIPIHYCAP 233
3   KDRTRKEYALFYKLDVVPIND-------TRYRLVSCNTSVITQACPKVSFEPIPIHYCAP 216
4   KDRTRKEYALFYKLDVVPIND-------TRYRLVSCNTSVITQACPKVSFEPIPIHYCAP 233
5   KDRTRKEYALFYKLDVVPIND-------TRYRLVSCNTSVITQACPKVSFEPIPIHYCAP 216
6   KDRTRKEYALFYKLDVVPINH-------TRYRLVSCNTSVITQACPKVSFEPIPIHYCAP 216
7   KDRTRKEYALFYKLDVVPIND-------TRYRLVSCNTSVITQACPKVSFEPIPIHYCAP 233
8   KDRTRKEYALFYKLDVVPIND-------TRYRLVSCNTSVITQACPKVSFEPIPIHYCAP 216
9   RDRTRKEYALFYKLDVVPIND-------TRYRLVSCNTSVITQACPKVSFEPIPIHYCAP 216
10  TDRTRKEYALFYKLDVVPIND-------TRYRLVSCNTSVITQACPKVSFEPIPIHYCAP 216
11  KDRTRKEYALFYKLDVVPIND-------TRYRLVSCNTSVITQACPKVSFEPIPIHYCAP 233
12  KDRTRKEYALFYKLDVVPIND-------TRYRLVSCNTSVITQACPKVSFEPIPIHYCAP 216
13  KDRTRKEYALFYKLDVVPIND-------TRYRLVSCNTSVITQACPKVSFEPIPIHYCAP 233
14  KDRTRKEYALFYKLDVVPIND-------TRYRLVSCNTSVITQACPKVSFEPIPIHYCAP 216
15  KDRMRKEYALFYKLDVVPIND-------TRYRLVSCNTSVITQACPKVSFEPIPIHYCAP 233
16  TDRTRKEYALFYKLDVVPIND-------TRYRLVSCNTSVITQACPKVSFEPIPIHYCAP 216
17  KDRTRKEYALFYNLDVVPIND-------TRYRLVSCNTSVITQACPKVSFEPIPIHYCAP 216
18  KDRTRKEYALFYKLDVVPIND-------TRYRLVSCNTSVITQACPKVSFEPIPIHYCAP 233
19  KDRMRKEYALFYKLDVVPIND-------TRYRLVSCNTSVITQACPKVSFEPIPIHYCAP 233
20  KDRTRTEYALFYKLDVVPIND-------TRYRLVSCNTSVITQACPKVSFEPIPIHYCAP 216
21  KDRTRKEYALFYKLDVVPIND-------TRYRLVSCNTSVITQACPKVSFEPIPIHYCAP 216
22  KDRTRKEYALFYKLDVVPIND-------TRYRLVSCNTSVITQACPKVSFEPIPIHYCAP 216
23  KDRTRKEYALFYKLDVVPIND-------TRYRLVSCNTSVITQACPKVSFEPIPIHYCAP 216
24  KDRTRKEYALFYKLDVVPIND-------TRYRLVSCNTSVITQACPKVSFEPIPIHYCAP 216
25  KDRTRKEYALFYKLDVVPIND-------TRYRLVSCNTSVITQACPKVSFEPIPIHYCAP 233
26  KDRTRKEYALFYKLDVVPIND-------TRYRLVSCNTSVITQACPKVSFEPIPIHYCAP 216
```

AGFAILKCNDKQFIGTGPCTNVSTVQCTHGIRPVVSTQLLLNGSLAEEEVVIRSVNFSDN

1	AGFAILKCNDKQFIGTGPCTNVSTVQCTHGIRPVVSTQLLLNGSLAEEEVVIRSVNFSDN	276
2	AGFAILKCNDKQFNGTGPCTNVSTVQCTHGIRPVVSTQLLLNGSLAEEEVVIRSVNFSDN	293
3	AGFAILKCNDKQFIGTGPCTNVSTVQCTHGIRPVVSTQLLLNGSLAEEEVVIRSVNFSDN	276
4	AGFAILKCNDKQFIGTGPCTNVSTVQCTHGIRPVVSTQLLLNGSLAEEEVVIRSVNFSDN	293
5	AGFAILKCNDKQFIGTGPCTNVSTVQCTHGIRPVVSTQLLLNGSLAEEEVVIRSVNFSDN	276
6	AGFAILKCNDKQFIGTGPCTNVSTVQCTHGIRPVVSTQLLLNGSLAEEEVVIRSVNFSDN	276
7	AGFAILKCNDKQFIGTGPCTNVSTVQCTHGIRPVVSTQLLLNGSLAEEEVVIRSVNFSDN	293
8	AGFAILKCNDKQFNGTGPCTNVSTVQCTHGIRPVVSTQLLLNGSLAEEEVVIRSVNFRDN	276
9	AGFAILKCNDKQFNGTGPCTNVSTVQCTHGIRPVVSTQLLLNGSLAEEEVVIRSVNFSDN	276
10	AGFAILKCNDKQFNGTGPCTNVSTVQCTHGIRPVVSTQLLLNGSLAEEEVVIRSVNFSDN	276
11	AGFAILKCNDKQFNGTGPCTNVSTVQCTHGIRPVVSTQLLLNGSLAEEEVVIRSVNFSDN	293
12	AGFAILKCNDKQFIGTGPCTNVSTVQCTHGIRPVVSTQLLLNGSLAEEEVVIRSVNFSDN	276
13	AGFAILKCNDKQFNGTGPCTNVSTVQCTHGIRPVVSTQLLLNGSLAEEEVVIRSVNFSDN	293
14	AGFAILKCNDKQFIGTGPCTNVSTVQCTHGIRPVVSTQLLLNGSLAEEEVVIRSVNFSDN	276
15	AGFAILKCNDKQFNGTGPCTNVSTVQCTHGIRPVVSTQLLLNGSLAEEEVVIRSVNFSDN	293
16	AGFAILKCNDKQFNGTGPCTNVSTVQCTHGIRPVVSTQLLLNGSLAEEEVVIRSVNFSDN	276
17	AGFAILKCNDKQFNGTGPCTNVSTVQCTHGIRPVVSTQLLLNGSLAEEEVVIRSVNFSDN	276
18	AGFAILKCNDKQFIGTGPCTNVSTVQCTHGIRPVVSTQLLLNGSLAEEEVVIRSVNFSDN	293
19	AGFAILKCNDKQFNGTGPCTNVSTVQCTHGIRPVVSTQLLLNGSLAEEEVVIRSVNFSDN	293
20	AGFAILKCNDKQFIGTGPCTNVSTVQCTHGIRPVVSTQLLLNGSLAEEEVVIRSVNFSDN	276
21	AGFAILKCNDKQFNGTGPCTNVSTVQCTHGIRPVVSTQLLLNGSLAEEEVVIRSVNFSDN	276
22	AGFAILKCNDKQFIGTGPCTNVSTVQCTHGIRPVVSTQLLLNGSLAEEEVVIRSVNFSDN	276
23	AGFAILKCNDKQFNGTGPCTNVSTVQCTHGIRPVVSTQLLLNGSLAEEEVVIRSVNFSDN	276
24	AGFAILKCNDKQFIGTGPCTNVSTVQCTHGIRPVVSTQLLLNGSLAEEEVVIRSVNFNDN	276
25	AGFAILKCNDKQFIGTGPCTNVSTVQCTHGIRPVVSTQLLLNGSLAEEEVVIRSVNFSDN	293
26	AGFAILKCNDKQFIGTGPCTNVSTVQCTHGIRPVVSTQLLLNGSLAEEEVVIRSVNFSDN	276

	AKTIIVQLNKSVEINCTRPXNNTRKSIPMGPGKAFYARGDIIGDIRKAYCEINGTEWHST	
1	AKTIIVQLNKSVEITCTRPNNNTRKSIPMGPGKAFYARGDITGDIRKAYCEINGTEWHST	336
2	AKTIIVQLNKSVEINCTRPNNNTRKSIPMGPGKAFYARGDIIGDIRKASCEINGTEWHST	353
3	AKTIIVQLNKSVEINCTRPNNNTRKSIPMGPGKAFYARGDIIGNIRKASCELDGTEWHST	336
4	AKTIIVQLNKSVEINCTRPHNNTRKSIPMGPGKAFYARGDIIGDIRKAYCEINGTEWHST	353
5	AKTIIVQLNKSVEINCTRPNNNTRKSIPMGPGKAFYARGDIIGDIRKASCELDGTEWHST	336
6	AKTIIVQLNKSVEINCTRPNNNTRKSIPMGPGKAFYARGDIIGNIRKASCELDGTEWHST	336
7	AKTIIVQLNKSVEINCTRPHNNTRKSIPMGPGKAFYARGDIIGDIRKAYCEINGTEWHST	353
8	AKTIIVQLNKSVEINCTRPHNNTRKSIPMGPGKAFYARGDIIGNIRKAYCEINGTEWHST	336
9	AKTIIVQLNKSVEINCTRPHNNTRKSIPMGPGKAFYARGDIIGDIRKAYCNINGTEWHST	336
10	AKTIIVQLNKSVEINCTRPHNNTRKSIPMGPGKAFYARGDIIGNIRKAYCTINGTEWHST	336
11	AKTIIVQLNKSVEITCTRPHNNTRKSIPMGPGKAFYAKGDIIGDIRKAYCEINGTEWHST	353
12	AKTIIVQLNKSVEINCTRPHNNTRKSIPMGPGKAFYARGDIIGDIRKAYCEINGTEWHST	336
13	AKTIIVQLNKSVEINCTRPNNNTRKSIPMGPGKAFYARGDIIGDIRKASCEINGTEWHST	353
14	AKTIIVQLNKSVEINCTRPNNNTRKSIPMGPGKAFYARGDIIGDIRKASCELDGTEWHST	336
15	AKTIIVQLNKSVEMTCTRPNNNTRKSIPMGPGKAFYARGDIIGDIRKASCEINGTEWHST	353
16	AKTIIVQLNKSVEINCTRPHNNTRKSIPMGPGKAFYARGDIIGNIRKASCEINGTEWHST	336
17	AKTIIVQLNKSVEINCTRPHNNTRKSIPMGPGKAFYARGDIIGDIRKAYCTINGTEWHSA	336
18	AKTIIVQLNKSVEITCTRPNNNTRKSIPMGPGKAFYARGDIIGDIRKASCEINGTEWHST	353
19	AKTIIVQLNKSVEMTCTRPNNNTRKSIPMGPGKAFYARGDIIGDIRKASCEINGTEWHST	353
20	AKTIIVQLNKSVEINCTRPHNNTRKSIPMGPGKAFYAKGDIIGDIRKAYCEINGTEWHST	336
21	AKTIIVQLNKSVEINCTRPHNNTRKSIPMGPGKAFYARGDIIGDIRKAYCTINGTEWHST	336
22	AKTIIVQLNKSVEINCTRPNNNTRKSIPMGPGKAFYARGDIIGDIRKASCELDGTEWHST	336
23	AKTIIVQLNKSVEINCTRPHNNTRKSIPMGPGKAFYARGDIIGDIRKAYCTINGTEWHST	336
24	AKTIIVQLNKSVEINCTRPHNNTRKSIPMGPGKAFYARGDIIGNIRKAYCEINGTEWHST	336
25	AKTIIVQLNKSVEITCTRPNNNTRKSIPMGPGKAFYARGDIIGDIRKASCEINGTEWHST	353
26	AKTIIVQLNKSVEITCTRPNNNTRKSIPMGPGKAFYARGDITGDIKKAYCKINGTEWHST	336

	LKLVVEKLREQY-NKTIVFNRSSGGDPEIVMYSFNCGGEFFYCNSTKLFNSTWPWND-TK	
1	LKLVVEKLREQY-NKTIVFNRSSGGDPEIVMYSFNCGGEFFYCNSTKLFNSTWPWND-TK	394
2	LKLVVEKLREQY-NKTIVFNRSSGGDPEIVMYSFNCGGEFFYCNSTKLFNSTWPWND-TK	411
3	LKLVVEQLRKQY-NKTIVFNRSSGGDPEIVMYSFNCGGEFFYCNSTKLFNSTWPWND-TK	394
4	LKLVVGKLREKY-NKTIVFNRSSGGDPEIVMYSFNCGGEFFYCNSTKLFNSTWPWND-NK	411
5	LKLVVEQLRKQY-NKTIVFNRSSGGDPEIVMYSFNCGGEFFYCNSTKLFNSTWPWND-TK	394
6	LKLVVEKLRKQY-NKTIVFNRSSGGDPEIVMYSFNCGGEFFYCNSTKLFNSTWPWND-TK	394
7	LKLVVGKLREKY-NKTIVFNRSSGGDPEIVMYSFNCGGEFFYCNSTKLFNSTWPWND-TK	411
8	LKLVVEKLREKY-NKTIVFNRSSGGDPEIVMYSFNCGGEFFYCNSTKLFNSTWPWND-TK	394
9	LKLVVEKLREQY-NKTIVFNRSSGGDPEIVMYSFNCGGEFFYCNSTKLFNSTWPWND-TK	394
10	LKLVVEKLREQY-NKTIVFNRSSGGDPEIVMYSFNCGGEFFYCNSTKLFNSTWPWND-TK	394
11	LKLVVEKLRERY-NKTIVFNRSSGGDPEIVMYSFNCGGEFFYCNSTKLFNSTWPWND-TK	411
12	LKLVVGKLREKY-NKTIVFNRSSGGDPEIVMYSFNCGGEFFYCNSTKLFNSTWPWND-TK	394
13	LKLVVEKLREQY-NKTIVFNRSSGGDPEIVMYSFNCGGEFFYCNSTKLFNSTWPWND-TK	411
14	LKLVVEKLRKQY-NKTIVFNRSSGGDPEIVMYSFNCGGEFFYCNSTKLFNSTWPWND-TK	394
15	LKMVVEKLREQY-NKTIVFNRSSGGDPEIVMYSFNCGGEFFYCNSTKLFNSTWPWND-TK	411
16	LKLVVEKLREQY-NKTIVFNRSSGGDPEIVMYSFNCGGEFFYCNSTKLFNSTWPWND-TK	394
17	LKLVVEKLREQY-NKTIVFNRSSGGDPEIVMYSFNCGGEFFYCNSTKLFNSTWPWND-TK	394
18	LKLVVEKLREQY-NKTIVFNRSSGGDPEIVMYSFNCGGEFFYCNSTKLFNSTWPWND-TK	411
19	LKMVVEKLREQY-NKTIVFNRSSGGDPEIVMYSFNCGGEFFYCNSTKLFNSTWPWND-TK	411
20	LKLVVGKLRERY-NKTIVFNRSSGGDPEIVMYSFNCGGEFFYCNSTKLFNSTWPWND-TK	394
21	LKLVVEKLREQY-NKTIVFNRSSGGDPEIVMYSFNCGGEFFYCNSTKLFNSTWPWND-TK	394
22	LKLVVEKLRKQY-NKTIVFNRSSGGDPEIVMYSFNCGGEFFYCNSTKLFNSTWPWND-TK	394
23	LKLVVEKLREQY-NKTIVFNRSSGGDPEIVMYSFNCGGEFFYCNSTKLFNSTWPWND-TK	394
24	LKLVVEKLREKY-NKTIVFNRSSGGDPEIVMYSFNCGGEFFYCNSTKLFNSTWPWND-TK	394
25	LKLVVEKLREQY-NKTIVFNRSSGGDPEIVMYSFNCGGEFFYCNSTKLFNSTWPWND-TK	411
26	LKLVVEKLREQY-NKTIVFNRSSGGDPEIVMYSFNCGGEFFYCNSTKLFNSTWPWND-TK	394

	Sequence	Position
	GSHDTNGTLILPCRIKQIINMWQGVGKAMYAPPIEGXIRCSSNITGLLLTRDGG-YESNE	
1	GSHDTNGTLILPCKIKQIINMWQGVGKAMYAPPIEGKIRCSSNITGLLLTRDGG-YESNE	453
2	GSHDTNGKLILPCRIKQIINMWQGVGKAMYAPPIEGIIRCSSNITGLLLTRDGG-YESNE	470
3	GSHDTNGTLILPCRIKQIINMWQGVGKAMYAPPIEGEIRCSSNITGLLLTRDGG-YESNE	453
4	GSHDTNGKLILPCRIKQIINMWQGVGKAMYAPPIEGIIRCSSNITGLLLTRDGG-YESNE	470
5	GSHDTNGTLILPCRIKQIINMWQGVGKAMYAPPIEGEIRCSSSITGLLLTRDGG-YESNE	453
6	GSHDTNGTLILPCRIKQIINMWQGVGKAMYAPPIEGEIRCSSNITGLLLTRDGG-YESNE	453
7	GSHDTNGKLILPCRIKQIINMWQGVGKAMYAPPIEGIIRCSSNITGLLLTRDGG-YESNE	470
8	GSHDTNGTLILPCRIKQIINMWQGVGKAMYAPPIEGEIRCSSNITGLLLTRDGG-YESNE	453
9	GSHDTNDTLMLPCKIKQIINMWQGVGKAMYAPPIEGQISCSSNITGLLLTRDGG-YESNK	453
10	GSHDTNDTLMLPCKIKQIINMWQGVGKAMYAPPIEGQIRCSSNITGLLLTRDGG-YESNE	453
11	GSHDTNGTLTLPCRIKQIINMWQGVGKAMYAPPIEGKIRCSSNITGLLLTRDGG-YESNK	470
12	GSHDTNDTLMLPCKIKQIINMWQGVGKAMYAPPIEGQIRCSSNITGLLLTRDGG-YESNE	453
13	GSHDTNGKLILPCRIKQIINMWQGVGKAMYAPPIEGIIRCSSNITGLLLTRDGG-YESNE	470
14	GSHDTNGTLILPCRIKQIINMWQGVGKAMYAPPIEGEIRCSSNITGLLLTRDGG-YESNE	453
15	GSHDTNGKLILPCRIKQIINMWQGVGKAMYAPPIEGIIRCSSNITGLLLTRDGG-YESNE	470
16	GSHDTNGKLILPCRIKQIINMWQGVGKAMYAPPIEGIIRCSSNITGLLLTRDGG-YESNE	453
17	GSHDTNDTLMLPCKIKQIINMWQGVGKAMYAPPIEGQIRCSSNITGLLLTRDGG-YESNE	453
18	GSHDTNGKLILPCRIKQIINMWQGVGKAMYAPPIEGIIRCSSNITGLLLTRDGG-YESNE	470
19	GSHDTNGKLILPCRIKQIINMWQGVGKAMYAPPIEGIIRCSSNITGLLLTRDGG-YESNE	470
20	GSHDTNGTLTLPCRIKQIINMWQGVGKAMYAPPIEGKIRCSSNITGLLLTRDGG-YESNK	453
21	GSHDTNDTLMLPCKIKQIINMWQGVGKAMYAPPIEGQIRCSSNITGLLLTRDGG-YESNE	453
22	GSHDTNGTLILPCRIKQIINMWQGVGKAMYAPPIEGEIRCSSNITGLLLTRDGG-YESNE	453
23	GSHDTNDTLMLPCKIKQIINMWQGVGKAMYAPPIEGQIRCSSNITGLLLTRDGG-YESNE	453
24	GSHDTNGTLILPCRIKQIINMWQGVGKAMYAPPIEGEIRCSSNITGLLLTRDGG-YESNE	453
25	GSHDTNGKLILPCRIKQIINMWQGVGKAMYAPPIEGIIRCSSNITGLLLTRDGG-YESNE	470
26	GSHDTNGTLILPCKIKQIINMWQGVGKAMYAPPIEEKIRCSSNITGLLLTRDGG-YESNE	453

	TDEIFRPGGGDMRDNWRSELYKYKVVKIEPLGVAPTKAKRRVVQREKRAFG-LGAVFLGF	
1	TDEIFRPGGGDMRDNWRSELYKYKVVKIEPLGVAPTKAKRRVVQREKRAFG-LGAVFLGF	512
2	TDEIFRPGGGDMRDNWRSELYKYKVVKIEPLGVAPTKAKRRVVQREKRAFG-LGALFLGF	529
3	TDEIFRPGGGDMRDNWRSELYKYKVVKIEPLGVAPTKAKRRVVQREKRAFG-LGAVFLGF	512
4	TDKIFRPGGGDMRDNWRSELYKYKVVKIEPLGVAPTKAKRRVVQREKRAFG-LGAVFLGF	529
5	TDEIFRPGGGDMRDNWRSELYKYKVVKIEPLGVAPTKAKRRVVQREKRAFG-LGAVFLGF	512
6	TDEIFRPGGGDMRDNWRSELYKYKVVKIEPLGVAPTKAKRRVVQREKRAFG-LGAVFLGF	512
7	TDEIFRPGGGDMRDNWRSELYKYKVVKIEPLGVAPTKAKRRVVQREKRAFG-LGAVFLGF	529
8	TDEIFRPGGGDMRDNWRSELYKYKVVKIEPLGVAPTKAKRRVVQREKRAFG-LGAVFLGF	512
9	TDEIFRPGGGDMRDNWRSELYKYKVVKIEPLGVAPTKAKRRVVQREKRAFG-LGAVFLGF	512
10	TDEIFRPGGGDMRDNWRSELYKYKVVKIEPLGVAPTKAKRRMVQREKRAFG-LGAVFLGF	512
11	TDEIFRPGGGDMRDNWRSELYKYKVVKIEPLGVAPTKAKRRVVQREKRAFG-LGAVFLGF	529
12	TDEIFRPGGGDMRDNWRSELYKDKVVKIEPLGVAPTKAKRRVVQREKRAFG-LGAVFLGF	512
13	TDEIFRPGGGDMRDNWRSELYKYKVVKIEPLGVAPTKAKRRVVQREKRAFG-LGALFLGF	529
14	TDEIFRPGGGDMRDNWRSELYKYKVVKIEPLGVAPTKAKRRVVQREKRAFG-LGAVFLGF	512
15	TDEIFRPGGGDMRDNWRSELYKYKVVKIEPLGVAPTKAKRRVVQREKRAFG-LGAVFLGF	529
16	TDEIFRPGGGDMRDNWRSELYKYKVVKIEPLGVAPTKAKRRVVQREKRAFG-LGAVFLGF	512
17	TDEIFRPGGGDMRDNWRSELYKYKVVKIEPLGVAPTKAKRRVVQREKRAFG-LGAVFLGF	512
18	TDEIFRPGGGDMRDNWRSELYKYKVVKIEPLGVAPTKAKRRVVQREKRAFG-LGAVFLGF	529
19	TDEIFRPGGGDMRDNWRSELYKYKVVKIEPLGVAPTKAKRRVVQREKRAFG-LGAVFLGF	529
20	TDEIFRPGGGDMRDNWRSELYKYKVVKIEPLGVAPTKAKRRVVQREKRAFG-LGAVFLGF	512
21	TDEIFRPGGGDMRDNWRSELYKYKVVKIEPLGVAPTKAKRRVVQREKRAFG-LGAVFLGF	512
22	TDEIFRPGGGDMRDNWRSELYKYKVVKIEPLGVAPTKAKRRVVQREKRAFG-LGAVFLGF	512
23	TDEIFRPGGGDMRDNWRSELYKYKVVKIEPLGVAPTKAKRRVVQREKRAFG-LGAVFLGF	512
24	TDEIFRPGGGDMRDNWRSELYKYKVVKIEPLGVAPTKAKRRVVQREKRAFG-LGAVFLGF	512
25	TDEIFRPGGGDMRDNWRSELYKYKVVKIEPLGVAPTKAKRRVVQREKRAFG-LGAVFLGF	529
26	TDEIFRPGGGDMRDNWRSELYKYKVVKIEPLGVAPTKAKRRVVQREKRAFG-LGAVFLGF	512

	**LGAAGSTMGAASITLTVQARQLLSGIVQQQNNLLRAIEAQQHLLQLTVWGIKQLQARVLA**	
1	LGAAGSTMGAASITLTVQARQLLSGIVQQQNNLLRAIEAQQHLLQLTVWGIKQLQARVLA	572
2	LGAAGSTMGAASITLTVQARQLLSGIVQQQNNLLRAIEAQQHLLQLTVWGIKQLQARVLA	589
3	LGAAGSTMGAASITLTVQARQLLSGIVQQQNNLLRAIEAQQHLLQLTVWGIKQLQARVLA	572
4	LGAAGSTMGAASITLTVQARQLLSGIVQQQNNLLRAIEAQQHLLQLTVWGIKQLQARVLA	589
5	LGAAGSTMGAASITLTVQARQLLSGIVQQQNNLLRAIEAQQHLLQLTVWGIKQLQARVLA	572
6	LGAAGSTMGAASITLTVQARQLLSGIVQQQNNLLRAIEAQQHLLQLTVWGIKQLQARVLA	572
7	LGAAGSTMGAASITLTVQARQLLSGIVQQQNNLLRAIEAQQHLLQLTVWGIKQLQARVLA	589
8	LGAAGSTMGAASITLTVQARQLLSGIVQQQNNLLRAIEAQQHLLQLTVWGIKQLQARVLA	572
9	LGAAGSTMGAASITLTVQARQLLSGIVQQQNNLLRAIEAQQHLLQLTVWGIKQLQARVLA	572
10	LGAAGSTMGAASITLTVQARQLLSGIVQQQNNLLRAIEAQQHLLQLTVWGIKQLQARVLA	572
11	LGAAGSTMGAASITLTVQARQLLSGIVQQQNNLLRAIEAQQHLLQLTVWGIKQLQARVLA	589
12	LGAAGSTMGAASITLTVQARQLLSGIVQQQNNLLRAIEAQQHLLQLTVWGIKQLQARVLA	572
13	LGAAGSTMGAASITLTVQARQLLSGIVQQQNNLLRAIEAQQHLLQLTVWGIKQLQARVLA	589
14	LGAAGSTMGAASITLTVQARQLLSGIVQQQNNLLRAIEAQQHLLQLTVWGIKQLQARVLA	572
15	LGAAGSTMGAASITLTVQARQLLSGIVQQQNNLLRAIEAQQHLLQLTVWGIKQLQARVLA	589
16	LGAAGSTMGAASITLTVQARQLLSGIVQQQNNLLRAIEAQQHLLQLTVWGIKQLQARVLA	572
17	LGAAGSTMGAASITLTVQARQLLSGIVQQQNNLLRAIEAQQHLLQLTVWGIKQLQARVLA	572
18	LGAAGSTMGAASITLTVQARQLLSGIVQQQNNLLRAIEAQQHLLQLTVWGIKQLQARVLA	589
19	LGAAGSTMGAASITLTVQARQLLSGIVQQQNNLLRAIEAQQHLLQLTVWGIKQLQARVLA	589
20	LGAAGSTMGAASITLTVQARQLLSGIVQQQNNLLRAIEAQQHLLQLTVWGIKQLQARVLA	572
21	LGAAGSTMGAASITLTVQARQLLSGIVQQQNNLLRAIEAQQHLLQLTVWGIKQLQARVLA	572
22	LGAAGSTMGAASITLTVQARQLLSGIVQQQNNLLRAIEAQQHLLQLTVWGIKQLQARVLA	572
23	LGAAGSTMGAASITLTVQARQLLSGIVQQQNNLLRAIEAQQHLLQLTVWGIKQLQARVLA	572
24	LGAAGSTMGAASITLTVQARQLLSGIVQQQNNLLRAIEAQQHLLQLTVWGIKQLQARVLA	572
25	LGAAGSTMGAASITLTVQARQLLSGIVQQQNNLLRAIEAQQHLLQLTVWGIKQLQARVLA	589
26	LGAAGSTMGAASITLTVQARQLLSGIVQQQNNLLRAIEAQQHLLQLTVWGIKQLQARVLA	572

VERYLKDQQLLGIWGCSGKLICTTTVPWNTSWSNKSLEQIWDNMTWMEWEREIDNYTGYI

1	VERYLKDQQLLGIWGCSGKLICTTTVPWNTSWSNKSLEQIWDNMTWMEWEREIDNYTGYI	632
2	VERYLKDQQLLGIWGCSGKLICTTTVPWNNSWSNKSLEQIWDNMTWMEWEREIDNYTGYI	649
3	VERYLKDQQLLGIWGCSGKLICPTTVPWNTSWSNKSLEQIWDNMTWMEWEREIDNYTGYI	632
4	VERYLKDQQLLGIWGCSGKLICTTTVPWNTSWSNKSLEQIWDNMTWMEWEREIDNYTGYI	649
5	VERYLKDQQLLGIWGCSGKLICPTTVPWNTSWSNKSLEQIWDNMTWMEWEREIDNYTGYI	632
6	VERYLKDQQLLGIWGCSGKLICPTTVPWNTSWSNKSLEQIWDNMTWMEWEREIDNYTGYI	632
7	VERYLKDQQLLGIWGCSGKLICTTTVPWNTSWSNKSLEQIWDNMTWMEWEREIDNYTGYI	649
8	VERYLKDQQLLGIWGCSGKLICTTTVPWNTSWSNKSLEQIWDNMTWMEWEREIDNYTGYI	632
9	VERYLKDQQLLGIWGCSGKLICTTTVPWNTSWSNKSLEQIWDNMTWMEWEREIDNYTGYI	632
10	VERYLKDQQLLGIWGCSGKLICTTTVPWNTSWSNKSLEQIWDNMTWMEWEREIDNYTGYI	632
11	VERYLKDQQLLGIWGCSGKLICTTTVPWNTSWSNKSLEQIWDNMTWMEWEREIDNYTGYI	649
12	VERYLKDQQLLGIWGCSGKLICPTTVPWNTSWSNKSLEQIWDNMTWMEWEREIDNYTGYI	632
13	VERYLKDQQLLGIWGCSGKLICTTTVPWNNSWSNKSLEQIWDNMTWMEWEREIDNYTGYI	649
14	VERYLKDQQLLGIWGCSGKLICPTTVPWNTSWSNKSLEQIWDNMTWMEWEREIDNYTGYI	632
15	VERYLKDQQLLGIWGCSGKLICTTTVPWNTSWSNKSLEQIWDNMTWMEWEREIDNYTGYI	649
16	VERYLKDQQLLGIWGCSGKLICTTTVPWNTSWSNKSLEQIWDNMTWMEWEREIDNYTGYI	632
17	VERYLKDQQLLGIWGCSGKLICTTTVPWNTSWSNKSLEQIWDNMTWMEWEREIDNYTGYI	632
18	VERYLKDQQLLGIWGCSGKLICTTTVPWNTSWSNKSLEQIWDNMTWMEWEREIDNYTGYI	649
19	VERYLKDQQLLGIWGCSGKLICTTTVPWNTSWSNKSLEQIWDNMTWMEWEREIDNYTGYI	649
20	VERYLKDQQLLGIWGCSGKLICTTTVPWNTSWSNKSLEQIWDNMTWMEWEREIDNYTGYI	632
21	VERYLKDQQLLGIWGCSGKLICTTTVPWNTSWSNKSLEQIWDNMTWMEWEREIDNYTGYI	632
22	VERYLKDQQLLGIWGCSGKLICPTTVPWNTSWSNKSLEQIWDNMTWMEWEREIDNYTGYI	632
23	VERYLKDQQLLGIWGCSGKLICTTTVPWNTSWSNKSLEQIWDNMTWMEWEREIDNYTGYI	632
24	VERYLKDQQLLGIWGCSGKLICTTTVPWNTSWSNKSLEQIWDNMTWMEWEREIDNYTGYI	632
25	VERYLKDQQLLGIWGCSGKLICTTTVPWNTSWSNKSLEQIWDNMTWMEWEREIDNYTGYI	649
26	VERYLKDQQLLGIWGCSGKLICTTTVPWNTSWSNKSLEQIWDNMTWMEWEREIDNYTGYI	632

	Sequence	
	**YQLIEESQNQQEKNEQELLALDKWASLWNWFDITNWLWYIKIFIMIVGGLIGLRIVFTVL**	
1	YQLIEESQNQQEKNEQELLALDKWASLWNWFDITNWLWYIKIFIMIVGGLIGLRIVFTVL	692
2	YQLIEESQNQQEKNEQELLALDKWASLWNWFDITNWLWYIKIFIMIVGGLIGLRIVFTVL	709
3	YQLIEESQNQQEKNEQELLALDKWASLWNWFDITNWLWYIKIFIMIVGGLIGLRIVFTVL	692
4	YQLIEESQNQQEKNEQELLALDKWASLWNWFDITNWLWYIKIFIMIVGGLIGLRIVFTVL	709
5	YQLIEESQNQQEKNEQELLALDKWASLWNWFDITNWLWYIKIFIMIVGGLIGLRIVFTVL	692
6	YQLIEESQNQQEKNEQELLALDKWASLWNWFDITNWLWYIKIFIMIVGGLIGLRIVFTVL	692
7	YQLIEESQNQQEKNEQELLALDKWASLWNWFDITNWLWYIKIFIMIVGGLIGLRIVFTVL	709
8	YQLIEESQNQQEKNEQELLALDKWASLWNWFDITNWLWYIKIFIMIVGGLIGLRIVFTVL	692
9	YQLIEESQNQQEKNEQELLALDKWASLWNWFDITNWLWYIKIFIMIVGGLIGLRIVFTVL	692
10	YQLIEESQNQQEKNEQELLALDKWASLWNWFDITNWLWYIKIFIMIVGGLIGLRIVFTVL	692
11	YQLIEESQNQQEKNEQELLALDKWASLWNWFDITNWLWYIKIFIMIVGGLIGLRIVFTVL	709
12	YQLIEESQNQQEKNEQELLALDKWASLWNWFDITNWLWYIKIFIMIVGGLIGLRIVFTVL	692
13	YQLIEESQNQQEKNEQELLALDKWASLWNWFDITNWLWYIKIFIMIVGGLIGLRIVFTVL	709
14	YQLIEESQNQQEKNEQELLALDKWASLWNWFDITNWLWYIKIFIMIVGGLIGLRIVFTVL	692
15	YQLIEESQNQQEKNEQELLALDKWASLWNWFDITNWLWYIKIFIMIVGGLIGLRIVFTVL	709
16	YQLIEESQNQQEKNEQELLALDKWASLWNWFDITNWLWYIKIFIMIVGGLIGLRIVFTVL	692
17	YQLIEESQNQQEKNEQELLALDKWASLWNWFDITNWLWYIKIFIMIVGGLIGLRIVFTVL	692
18	YQLIEESQNQQEKNEQELLALDKWASLWNWFDITNWLWYIKIFIMIVGGLIGLRIVFTVL	709
19	YQLIEESQNQQEKNEQELLALDKWASLWNWFDITNWLWYIKIFIMIVGGLIGLRLVFTVL	709
20	YQLIEESQNQQEKNEQELLALDKWASLWNWFDITNWLWYIKIFIMIVGGLIGLRIVFTVL	692
21	YQLIEESQNQQEKNEQELLALDKWASLWNWFDITNWLWYIKIFIMIVGGLIGLRIVFTVL	692
22	YQLIEESQNQQEKNEQELLVLDKWASLWNWFDITNWLWYIKIFIMIVGGLIGLRIVFTVL	692
23	YQLIEESQNQQEKNEQELLALDKWASLWNWFDITNWLWYIKIFIMIVGGLIGLRIVFTVL	692
24	YQLIEESQNQQEKNEQELLALDKWASLWNWFDITNWLWYIKIFIMIVGGLIGLRIVFTVL	692
25	YQLIEESQNQQEKNEQELLALDKWASLWNWFDITNWLWYIKIFIMIVGGLIGLRIVFTVL	709
26	YQLIEESQNQQEKNEQELLALDKWASLWNWFDITNWLWYIKIFIMIVGGLIGLRIVFTVL	692

SIVNRVRQGYSPLSFQTHLPAQRGPDRPEGIGEEGGERDRDRSDPLVNGFLTLIWSDLRS

1	SIVNRVRQGYSPLSFQTHLPAQRGPDRPEGIGEEGGERDRDRSDPLVNGFLALIWSDLRS	752
2	SIVNRVRQGYSPLSFQTHLPAQRGPDRPEGIGEEGGERDRDRSDPLVNGFLTLIWSDLRS	769
3	SIVNRVRQGYSPLSFQTHLPAQRGPDRPEGIGEEGGERDRDRSDPLVNGFLTLIWSDLRS	752
4	SIVNRVRQGYSPLSFQTHLPAQRGPDRPEGIGEEGGERDRDRSDPLVNGFLTLIWSDLRS	769
5	SIVNRVRQGYSPLSFQTHLPAQRGPDRPEGIGEEGGERDRDRSDPLVNGFLTLIWSDLRS	752
6	SIVNRVRQGYSPLSFQTHLPAQRGPDRPEGIGEEGGERDRDRSDPLVNGFLTLIWSDLRS	752
7	SIVNRVRQGYSPLSFQTHLPAQRGPDRPEGIGEEGGERDRDRSDPLVNGFLTLIWSDLRS	769
8	SIVNRVRQGYSPLSFQTHLPAQRGPDRPEGIGEEGGERDRDRSDPLVNGFLTLIWSDLRS	752
9	SIVNRVRQGYSPLSFQTHLPAQRGPDRPEGIGEEGGERDRDRSDPLVNGFLTLIWSDLRS	752
10	SIVNRVRQGYSPLSFQTHLPAQRGPDRPEGIGEEGGERDRDRSDPLVNGFLTLIWSDLRS	752
11	SIVNRVRQGYSPLSFQTHLPAQRGPDRPEGIGEEGGERDRDRSDPLVNGFLTLIWSDLRS	769
12	SIVNRVRQGYSPLSFQTHLPAQRGPDRPEGIGEEGGERDRDRSDPLVNGFLTLIWSDLRS	752
13	SIVNRVRQGYSPLSFQTHLPAQRGPDRPEGIGEEGGERDRDRSDPLVNGFLTLIWSDLRS	769
14	AIVNRVRQGYAPLSFQTHLPAQRGPDRPEGIGEEGGERDRDRSDPLVNGFLTLIWSDLRS	752
15	SIVNRVRQGYSPLSFQTHLPAQRGPDRPEGIGEEGGERDRDRSDPLVNGFLTLIWSDLRS	769
16	SIVNRVRQGYSPLSFQTHLPAQRGPDRPEGIGEEGGERDRDRSDPLVNGFLTLIWSDLRS	752
17	SIVNRVRQGYSPLSFQTHLPAQRGPDRPEGIGEEGGERDRDRSDPLVNGFLTLIWSDLRS	752
18	SIVNRVRQGYSPLSFQTHLLAQRGPDRPEGIGEEGGERDRDRSDPLVNGFLTLIWSDLRS	769
19	SIVNRVRQGYSPLSFQTHLPAQRGPDRPEGIGEEGGERDRDRSDPLVNGFLTLIWSDLRS	769
20	SIVNRVRQGYSPLSFQTHLPAQRGPDRPEGIGEEGGERDRDRSDPLVNGFLTLIWSDLRS	752
21	SIVNRVRQGYSPLSFQTHLPAQRGPDRPEGIGEEGGERDRDRSDPLVNGFLTLIWSDLRS	752
22	SIVNRVRQGYSPLSFQTHLPAQRGPDRPEGIGEEGGERDRDRSDPLVNGFLTLIWSDLRS	752
23	SIVNRVRQGYSPLSFQTHLPAQRGPDRPEGIGEEGGERDRDRSDPLVNGFLTLIWSDLRS	752
24	SIVNRVRQGYSPLSFQTHLPAQRGPDRPEGIGEEGGERDRDRSDPLVNGFLTLIWSDLRS	752
25	SIVNRVRQGYSPLSFQTHLPAQRGPDRPEGIGEEGGERDRDRSDPLVNGFLTLIWSDLRS	769
26	SIVNRVRQGYSPLSFQTHLPAQRGPDRPEGIGEEGGKRDRDRSDPLVNGFLALIWSDLRS	752

	Sequence	
	LCLFSYHRLRDLLLIVTRIVELLGR-------RGWEVLKYWWNLLQYWSQELKNSAVSLL	
1	LCLFSYHRLRDLLLIVTRIVELLGR-------RGWEVLKYWWNLLQYWSQELKNSAVSLL	805
2	LCLFSYHRLRDLLLIVTRIVELLGR-------RGWEVLKYWWNLLQYWSQELKNSAVSLL	822
3	LCLFSYHRLRDLLLIVTRIVELLGR-------RGWEVLKYWWNLLQYWSQELKNSAVSLL	805
4	LCLFSYHRLRDLLLIVTRIVELLGR-------RGWEVLKYWWNLLQYWSQELKNSAVSLL	822
5	LCLFSYHRLRDLLLIVTRIVELLGR-------RGWEVLKYWWNLLQYWSQELKNSAVSLL	805
6	LCLFSYHRLRDLLLI----VELLGR-------RGWEVLKYWWNLLQYWSQELKNSAVSLL	801
7	LCLFSYHRLRDLLLIVTRIVELLGR-------RGWEVLKYWWNLLQYWSQELKNSAVSLL	822
8	LCLFSYHRLRDLLLIVTRIVELLGR-------RGWEVLKYWWNLLQYWSQELKNSAVSLL	805
9	LCLFSYHRLRDLLLIVTRIVELLGR-------RGWEVLKYWWNLLQYWSQELKNSAVSLL	805
10	LCLFSYHRLRDLLLIVTRIVELLGR-------RGWEVLKYWWNLLQYWSQELKNSAVSLL	805
11	LCLFSYHRLRDLLLIVTRIVELLGR-------RGWEVLKYWWNLLQYWSQELKNSAVSLL	822
12	LCLFSYHHLRDLLLIVTRIVELLGR-------RGWEVLKYWWNLLQYWSQELKNSAVSLL	805
13	LCLFSYHRLRDLLLIVTRIVELLGR-------RGWEVLKYWWNLLQYWSQELKNSAVSLL	822
14	LCLFSYHRLRDLLLIVTRIVELLGR-------RGWEVLKYWWNLLQYWSQELKNSAVSLL	805
15	LCLFSYHRLRDLLLIVTRIVELLGR-------RGWEVLKYWWNLLQYWSQELKNSAVSLL	822
16	LCLFSYHSLRDLLLIVTRIVELLGR-------RGWEVLKYWWNLLQYWSQELKNSAVSLL	805
17	LCLFSYHRLRDLLLIVTRIVELLGR-------RGWEVLKYWWNLLQYWSQELKNSAVSLL	805
18	LCLFSYHRLRDLLLIVTRIVELLGR-------RGWEVLKYWWNLLQYWSQELKNSAVSLL	822
19	LCLFSYHRLRDLLLIVTRIVELLGR-------RGWEVLKYWWNLLQYWSQELKNSAVSLL	822
20	LCLFSYHRLRDLLLIVTRIVELLGR-------RGWEVLKYWWNLLQYWSQELKNSAVSLL	805
21	LCLFSYHRLRDLLLIVTRIVELLGR-------RGWEVLKYWWNLLQYWSQELKNSAVSLL	805
22	LCLFSYHRLRDLLLIVTRIVELLGR-------RGWEVLKYWWNLLQYWSQELKNSAVSLL	805
23	LCLFSYHRLRDLLLIVTRIVELLGR-------RGWEVLKYWWNLLQYWSQELKNSAVSLL	805
24	LCLFSYHRLRDLLLIVTRIVELLGR-------RGWEVLKYWWNLLQYWSQELKNSAVSLL	805
25	LCLFSYHRLGDLLLIVTRIVELLGR-------RGWEVLKYWWNLLQYWSQELKNSAVSLL	822
26	LCLFSYHRLRDLLLIVTRIVELLGR-------RGWEVLKYWWNLLQYWSQELKNSAVSLL	805

	NATAIAVAEGTDRVIEVVQRACRAILHIPRRIRQGLERALL	
1	NATAIAVAEGTDRVIEVVQRACRAILHIPRRIRQGLERALL	846
2	NATAIAVAEGTDRVIEVVQGACRAILHIPRRIRQGLERALL	863
3	NATAIAVAEGTDRVIEVVQRACRAILHIPRRIRQGLERALL	846
4	NATATAVAEGTDRVIEVVQRACRAILHIPRRIRQGLERALL	863
5	NATAIAVAEGTDRVIEVVQRACRAILHIPRRIRQGLERALL	846
6	NATAIAVAEGTDRVIEVVQRACRAILHIPRRIRQGLERALL	842
7	NATAIAVAEGTDRVIEVVQRACRAIRHIPRRIRQGLERALL	863
8	NATATAVAEGTDRVIEVVQRACRAILHIPRRIRQGLERALL	846
9	NATAIAVAEGTDRVIEVVQGACRAILHIPRRIRQGLERALL	846
10	NATAIAVAEGTDRVIEVVQRAGRAIRHIPRRIRQGLERALL	846
11	NTTAIAVAEGTDRVIEVVQGACRAILHIPRRIRQGLERALL	863
12	NATAIAVAEGTDRVIEVVQRACRAILHIPRRIRQGLERALL	846
13	NATAIAVAEGTDRVIEVVQGACRAILHIPRRIRQGLERALL	863
14	NATAIAVAEGTDRVIEVVQRACRAILHIPRRIRQGLERALL	846
15	NATAIAVAEGTDRVIEVVQGACRAILHIPRRIRQGLERALL	863
16	NATAIAVAEGTDRVIEVVQRAGRAIRHIPRRIRQGLERALL	846
17	NATAIAVAEGTDRVIEVVQRAGRAIRHIPRRIRQGLERALL	846
18	NATAIAVAEGTDRVIEVVQGACRAILHIPRRIRQGLERALL	863
19	NATAIAVAEGTDRVIEVVQGACRAILHIPRRIRQGLERALL	863
20	NATATAVAEGTDRVIEVVQRACRAILHIPRRIRQGLERALL	846
21	NATAIAVAEGTDRVIEVVQRAGRAIRHIPRRIRQGLERALL	846
22	NATAIAVAEGTDRVIEVVQRACRAILHIPRRIRQGLERALL	846
23	NATAIAVAEGTDRVIEVVQRAGRAIRHIPRRIRQGLERALL	846
24	NATATAVAEGTDRVIEVVQRACRAILHIPRRIRQGLERALL	846
25	NATAIAVAEGTDRVIEVVQRACRAICHIPRRIRQGLERALL	863
26	NATAIAVAEGTDRVIEVVQRACRAILHIPRRIRQGLERALL	846

Consensus

1. B.US.2006.700010040_C9_4520.EU289193
2. B.US.2010.CH0040_3_d1485_ipe032_15_08.MG900412
3. B.US.2010.CH0040_3_d1485_ipe032_15_09.MG900413
4. B.US.2010.CH0040_3_d1485_ipe032_15_10.MG900414
5. B.US.2010.CH0040_3_d1485_ipe032_15_11.MG900415
6. B.US.2010.CH0040_3_d1485_ipe032_15_13.MG900416
7. B.US.2010.CH0040_3_d1485_ipe032_15_16.MG900417
8. B.US.2010.CH0040_3_d1485_ipe032_15_18.MG900418
9. B.US.2010.CH0040_3_d1485_ipe032_15_19.MG900419
10. B.US.2010.CH0040_3_d1485_ipe032_15_24.MG900420
11. B.US.2010.CH0040_3_d1485_ipe032_15_25.MG900421
12. B.US.2010.CH0040_3_d1485_ipe032_15_27.MG900422
13. B.US.2010.CH0040_3_d1485_ipe032_15_28.MG900423
14. B.US.2010.CH0040_3_d1485_ipe032_15_29.MG900424
15. B.US.2010.CH0040_3_d1485_ipe032_15_30.MG900425
16. B.US.2010.CH0040_3_d1485_ipe032_15_31.MG900426
17. B.US.2010.CH0040_3_d1485_ipe032_15_32.MG900427
18. B.US.2010.CH0040_3_d1485_ipe032_15_33.MG900428
19. B.US.2010.CH0040_3_d1485_ipe032_15_37.MG900429
20. B.US.2010.CH0040_3_d1485_ipe032_15_38.MG900430
21. B.US.2010.CH0040_3_d1485_ipe032_15_39.MG900431
22. B.US.2010.CH0040_3_d1485_ipe032_15_41.MG900432
23. B.US.2010.CH0040_3_d1485_ipe032_15_42.MG900433
24. B.US.2010.CH0040_3_d1485_ipe032_15_45.MG900434
25. B.US.2010.CH0040_3_d1485_ipe032_15_46.MG900435
26. B.US.2010.CH0040_3_d1485_ipe032_15_47.MG900436
27. B.US.2010.CH0040_3_d1485_ipe032_15_48.MG900437
28. B.US.2010.CH0040_3_d1485_ipe032_27_06.MG900438
29. B.US.2010.CH0040_3_d1485_ipe032_2_49.MG900439
30. B.US.2010.CH0040_3_d1485_ipe032_2_50.MG900440
31. B.US.2010.CH0040_3_d1485_ipe032_9_03.MG900441

	Sequence	
	**MRVMGIRKNYQHLWREGILLLGILMICSAADNLWVTVYYGVPVWREATTTLFCASDAKAY**	
1	MRVMGIRKNYQHLWREGILLLGILMICSAADNLWVTVYYGVPVWREATTTLFCASDAKAY	60
2	MRVMGIRKNYQHLWREGILLLGILMICSAADNLWVTVYYGVPVWREATTTLFCASDAKAY	60
3	MRVMGIRKNYQHLWREGILLLGILMICSAADNLWVTVYYGVPVWREATTTLFCASDAKAY	60
4	MRAMGIRKNYQHLWREGILLLGILMICSAADNLWVTVYYGVPVWREATTTLFCASDAKAY	60
5	MRVMGIRKNYQHWWREGILLLGILMICSAADKLWVTVYYGVPVWREATTTLFCASDAKAY	60
6	MRVMGIRKNYQHLWREGILLLGILMICSAADNLWVTVYYGVPVWREATTTLFCASDAKAY	60
7	MRVMGIRKNYQHLWREGILLLGILMICSAADNLWVTVYYGVPVWREATTTLFCASDAKAY	60
8	MRVMGIRKNYQHLWREGILLLGILMICSAADNLWVTVYYGVPVWREATTTLFCASDAKAY	60
9	MRVMGIRKNYQHLWREGILLLGILMICSAADNLWVTVYYGVPVWREATTTLFCASDAKAY	60
10	MRVMGIRKNYQHLWREGILLLGILMICSAADNLWVTVYYGVPVWREATTTLFCASDAKAY	60
11	MRVMGIRKNYQHLWREGILLLGILMICSAADNLWVTVYYGVPVWREATTTLFCASDAKAY	60
12	MRVMGIRKNYQHLWRKGILLLGILMICSAADNLWVTVYYGVPVWREATTTLFCASDAKAY	60
13	MRVMGIRKNYQHLWREGILLLGILMICSAADNLWVTVYYGVPVWREATTTLFCASDAKAY	60
14	MRVMGIRKNYQHLWRKGILLLGILMICSAADNLWVTVYYGVPVWREATTTLFCASDAKAY	60
15	MRVMGIRKNYQHLWREGILLLGILMICSAADNLWVTVYYGVPVWREATTTLFCASDAKAY	60
16	MRVMGIRKNYQHLWREGILLLGILMICSAADNLWVTVYYGVPVWREATTTLFCASDAKAY	60
17	MRVMGIRKNYQHLWREGILLLGILMICSAADNLWVTVYYGVPVWREATTTLFCASDAKAY	60
18	MRVMGIRKNYQHLWREGILLLGILMICSAADNLWVTVYYGVPVWREATTTLFCASDAKAY	60
19	MRVMGIRKNYQHLWREGILLLGILMICSAADNLWVTVYYGVPVWREATTTLFCASDAKAY	60
20	MRVMGIRKNYQHLWREGILLLGILMICSAADNLWVTVYYGVPVWKEATTTLFCASDAKAY	60
21	MRVMGIRKNYQHLWREGILLLGILMICSAADNLWVTVYYGVPVWREATTTLFCASDAKAY	60
22	MRVMGIRKNYQHLWREGILLLGILMICSAADNLWVTVYYGVPVWREATTTLFCASDAKAY	60
23	MRVMGIRKNYQHLWREGILLLGILMICSAADKLWVTVYYGVPVWREATTTLFCASDAKAY	60
24	MRVMGIRKNYQHWWREGILLLGILMICSAADKLWVTVYYGVPVWREATTTLFCASDAKAY	60
25	MRVMGIRKNYQHLWREGILLLGILMICSAADNLWVTVYYGVPVWREATTTLFCASDAKAY	60
26	MRVMGIRKNYQHLWREGILLLGILMICSAADNLWVTVYYGVPVWREATTTLFCASDAKAY	60
27	MRVMGIRKNYQHLWREGILLLGILMICSATDNLWVTVYYGVPVWREATTTLFCASDAKAY	60
28	MRVMGIRKNYQHLWKEGILLLGILMICSAADNLWVTVYYGVPVWREATTTLFCASDAKAY	60
29	MRVMGIRKNYQHLWRKGILLLGILMICSAADNLWVTVYYGVPVWREATTTLFCASDAKAY	60
30	MRVMGIRKNYQHLWRKGILLLGILMICSAADNLWVTVYYGVPVWREATTTLFCASDAKAY	60
31	MRVMGIRKNYQHLWREGILLLGILMICSAADNLWVTVYYGVPVWREATTTLFCASDAKAY	60

DTEAHNVWATHACVPTDPNPQEVELKNVTENFNMWENNMVEQMHEDIISLWDQSLKPCVK

1	DTEAHNVWATHACVPTDPNPQEVELKNVTENFNMWENNMVEQMHEDIISLWDQSLKPCVK	120
2	DTEAHNVWATHACVPTDPNPQEVELKNVTENFNMGKNNMVEQMHEDIISLWDQSLKPCVK	120
3	DTEAHNVWATHACVPTDPNPQEVELKNVTENFNMWENNMVEQMHEDIISLWDQSLKPCVK	120
4	DTEAHNVWATHACVPTDPNPQEVELKNVTENFNMWENNMVEQMHEDIISLWDQSLKPCVK	120
5	DTEAHNVWATHACVPTDPNPQEVELKNVTENFNMWENNMVEQMHEDIISLWDQSLKPCVK	120
6	DTEAHNVWATHACVPTDPNPQEVELKNVTENFNMWENNMVEQMHEDIISLWDQSLKPCVK	120
7	DTEAHNVWATHACVPTDPNPQEVELKNVTENFNMWENNMVEQMHEDIISLWDQSLKPCVK	120
8	DTEAHNVWATHACVPTDPNPQEVELKNVTENFNMWENNMVEQMHEDIISLWDQSLKPCVK	120
9	DTEAHNVWATHACVPTDPNPQEVELKNVTENFNMWENNMVEQMHEDIISLWDQSLKPCVK	120
10	DTEAHNVWATHACVPTDPNPQEVELKNVTENFNMWENNMVEQMHEDIISLWDQSLKPCVK	120
11	DTEAHNGWATHACVPTDPNPQEVELKNVTENFNMWENNMVEQMHEDIISLWDQSLKPCVK	120
12	DTEAHNVWATHACVPTDPNPQEVELKNVTENFNMWENNMVEQMHEDIISLWDQSLKPCVK	120
13	DTEAHNVWATHACVPTDPNPQEVELKNVTENFNMWENNMVEQMHEDIISLWDQSLKPCVK	120
14	DTEAHNVWATHACVPTDPNPQEVELKNVTENFNMWENNMVEQMHEDIISLWDQSLKPCVK	120
15	DTEAHNVWATHACVPTDPNPQEVELKNVTENFNMWENNMVEQMHEDIISLWDQSLKPCVK	120
16	DTEAHNVWATHACVPTDPNPQEVELKNVTENFNMWENNMVEQMHEDIISLWDQSLKPCVK	120
17	DTEAHNVWATHACVPTDPNPQEVELKNVTENFNMWENNMVEQMHEDIISLWDQSLKPCVK	120
18	DTEAHNVWATHACVPTDPNPQEVELKNVTENFNMWENNMVEQMHEDIISLWDQSLKPCVK	120
19	DTEAHNVWATHACVPTDPNPQEVELKNVTENFNMWENNMVEQMHEDIISLWDQSLKPCVK	120
20	DTEAHNVWATHACVPTDPNPQEVELKNVTENFNMWENNMVEQMHEDIISLWDQSLKPCVK	120
21	DTEAHNVWATHACVPTDPNPQEVKLKNVTENFNMWENNMVEQMHEDIISLWDQSLKPCVK	120
22	DTEAHNVWATHACVPTDPNPQEVELKNVTENFNMWENNMVEQMHEDIISLWDQSLKPCVK	120
23	DTEAHNVWATHACVPTDPNPQEVELKNVTENFNMWENNMVEQMHEDIISLWDQSLKPCVK	120
24	DTEAHNVWATHACVPTDPNPQEVELKNVTENFNMWENNMVEQMHEDIISLWDQSLKPCVK	120
25	DTEAHNVWATHACVPTDPNPQEVELKNVTENFNMWENNMVEQMHEDIISLWDQSLKPCVK	120
26	DTEAHNVWATHACVPTDPNPQEVELKNVTENFNMWENNMVEQMHEDIISLWDQSLKPCVK	120
27	DTEAHNVWATHACVPTDPNPQEVELKNVTENFNMGKNNMVEQMHEDIISLWDQSLKPCVK	120
28	DTEAHNVWATHACVPTDPNPQEVELKNVTENFNMWENNMVEQMHEDIISLWDQSLKPCVK	120
29	DTEAHNVWATHACVPTDPNPQEVELKNVTENFNMWENNMVEQMHEDIISLWDQSLKPCVK	120
30	DTEAHNVWATHACVPTDPNPQEVELKNVTENFNMWENNMVEQMHEDIISLWDQSLKPCVK	120
31	DTEAHNVWATHACVPTDPNPQEVELKNVTENFNMWENNMVEQMHEDIISLWDQSLKPCVK	120

	LTPLCVTLNCTDNVTTTTNRNGTNCTDXXNVTXT---------TNXNETLMEKGEVKNCS	
1	LTPLCVTLNCTDLGNVTNTT------------------------NSNGEMMEKGEVKNCS	156
2	LTPLCVTLNCTDNVTTTTNRNGTNCTDLRNVTTT---------TNSNETLMEKGEVKNCS	171
3	LTPLCVTLNCTDNVTTTTNRNGTNCTDSGNVTNT---------TNRNGALMEKGEVKNCS	171
4	LTPLCVTLNCTDSGNGTTTTNRNGTL-----------------MGNETLMMEKGEVKNCS	163
5	LTPLCVTLNCTDNVTTTTNRNGTNCTDLRNVTNT---------TNSNETLMEKGEVKNCS	171
6	LTPLCVTLNCTDNVTTTTNRNGTNCTDLRNVTTT---------TNSNETLMEKGEVKNCS	171
7	LTPLCVTLNCTDLENVTTTTNRNGTNCTDSGNVTPTTNRNGT-LMGNETSMEKGEVKNCS	179
8	LTPLCVTLNCTDLGNVTTTTNRNGTNCTDSGNVTAT-------TNRNETFMEKEEV-NCS	172
9	LTPLCVTLNCTDLRNVTTTTNRNGTNCTDSGNGTTTTNRNGTLMGNETLMMEKGEVKNCS	180
10	LTPLCVTLNCTDNVTTTTNRNGTNCTDSGNVTNT---------TNRNGALMEKGEVKNCS	171
11	LTPLCVTLNCTDNVTTTTNRNGTNCTDLRNVTTT---------TNSNETWMEKGEVKNCS	171
12	LTPLCVTLNCTDNVTTTTNRNGTNCTDSGNVTNT---------TNRNGALMEKGEVKNCS	171
13	LTPLCVTLNCTDNVTTTTNRNGTNCTDSGNVTNT---------TNRNGALMEKGEVKNCS	171
14	LTPLCVTLNCTDNVTTTTNRNGTNCTDLRNVTTT---------TNSNETLMEKGEVKNCS	171
15	LTPLCVTLNCTDNVTTTTNRNGTNCTDLRNVTTT---------TNSNETLMEKGEVKNCS	171
16	LTPLCVTLNCTDNVTTTTNRNGTNCTDSGNVTNT---------TNRNGALMEKGEVKNCS	171
17	LTPLCVTLNCTDNVTTTTNRNGTNCTDLGNVNTT---------TNSNETLMEKGEVKNCS	171
18	LTPLCVTLNCTDNVTTTTNRNGTNCTDLGNVNTT---------TNSNETLMEKGEVKNCS	171
19	LTPLCVTLNCTDNVTTTTNRNGTNCTDSGNVTNT---------TNRNGALMEKGEVKNCS	171
20	LTPLCVTLNCTDVGNVTTIT------------------------NSNGTLMEKGEVKNCS	156
21	LTPLCVTLNCTDDLGNVTTTTNRNGTNCTDSGNVTNT------TNRNGALMEKGEVKNCS	174
22	LTPLCVTLNCTDNVTTTTNRNGTNCTDLRNVTTT---------TNSNETLMEKGEVKNCS	171
23	LTPLCVTLNCTDLENVTTTTNRNGTNCTDSGNVTPTTNRNGT-LMGNETLMEKGEVKNCS	179
24	LTPLCVTLNCTDNVTTTTNRNGTNCTDLRNVTTT---------TNSNETLMEKGEVKNCS	171
25	LTPLCVTLNCTDLENVTTTTNRNGINCTDSGNVTPTTNRNGT-LMGNETLMEKGEVKNCS	179
26	LTPLCVTLNCTDNVTTTTNRNGTNCTDLRNVTTT---------TNSNETLMEKGEVKNCS	171
27	LTPLCVTLNCTDNVTTTTNRNETNCTDLRNVTTT---------TNSNETLMEKGEVKNCS	171
28	LTPLCVTLNCTDNVTTTTNRNGTNCTDSGNVTNT---------TNRNGALMEKGEVKNCS	171
29	LTPLCVTLNCTDNVTTTTNRNGTNCTDLGNVNTT---------TNSNETLMEKGEVKNCS	171
30	LTPLCVTLNCTDNVTTTTNRNGTNCTDSGNVTNT---------TNRNGALMEKGEVKNCS	171
31	LTPLCVTLNCTDLRNVTTTTNRNGTNCTDSGNGTTTTNRNGTLMGNETLMMEKGEVKNCS	180

```
     FRITTDMQDRTRKEYALFYKLDVVPIND-------TRYRLVSCNTSVITQACPKVSFEPI
1    FKITTDIKDRTRKEYALFYKLDVVPIND-------TRYRLVSCNTSVITQACPKVSFEPI  209
2    FMITTDVKDRMRKEYALFYKLDVVPIND-------TRYRLVSCNTSVITQACPKVSFEPI  224
3    FRITTDMQDRTRKEYALFYKLDVVPIND-------TRYRLVSCNTSVITQACPKVSFEPI  224
4    FRITTDMKDRTRKEYALFYKLDVVPIND-------TSYRLVSCNTSVITQACPKVSFEPI  216
5    FMITTDVKDRMRKEYALFYKLDVVPIND-------TRYRLVSCNTSVITQACPKVSFEPI  224
6    FMITTDVKDRMRKEYALFYKLDVVPIND-------TRYRLVSCNTSVITQACPKVSFEPI  224
7    FRITTDMQDRTRKEYALFYKLDVVPIND-------TRYRLVSCNTSVITQACPKVSFEPI  232
8    FRITTDMQDRTRKEYALFYKLDVVPIND-------TRYRLVSCNTSVITQACPKVSFEPI  225
9    FRITTDMKDRTRKEYALFYKLDVVPIND-------TSYRLVSCNTSVITQACPKVSFEPI  233
10   FRITTDMQDRTRKEYALFYKLDVVPIND-------TRYRLVSCNTSVITQACPKVSFEPI  224
11   FRITTDMQDRTRKEYALFYKLDVVPIND-------TRYRLVSCNTSVITQACPKVSFEPI  224
12   FRITTDMQDRTRKEYALFYKLDVVPIND-------TRYRLVSCNTSVITQACPKVSFEPI  224
13   FRITTDMQDRTRKEYALFYKLDVVPIND-------TRYRLVSCNTSVITQACPKVSFEPI  224
14   FMITTDMQDRTRKEYALFYKLDVVPIND-------TRYRLVSCNTSVITQACPKVSFEPI  224
15   FMITTDVKDRMRKEYALFYKLDVVPIND-------TRYRLVSCNTSVITQACPKVSFEPI  224
16   FRITTDMQDRTRKEYALFYKLDVVPIND-------TRYRLVSCNTSVITQACPKVSFEPI  224
17   FMITTDVKDRMRKEYALFYKLDVVPIND-------TRYRLVSCNTSVITQACPKVSFEPI  224
18   FMITTDVKDRMRKEYALFYKLDVVPIND-------TRYRLVSCNTSVITQACPKVSFEPI  224
19   FRITTDMQDRTRKEYALFYKLDVVPIND-------TRYRLVSCNTSVITQACPKVSFEPI  224
20   FKITTDTRDRMRKEYALFYKLDVVPIND-------TRYRFVSCNTSVITQACPKVSFEPI  209
21   FRITTDMQDRTRKEYALFYKLDVVPIND-------TSYRLVSCNTSVITQACPKVSFEPI  227
22   FMITTDMQDRTRKEYALFYKLDVVPIND-------TRYRLVSCNTSVITQACPKVSFEPI  224
23   FRITTDMQDRTRKEYALFYKLDVVPIND-------TRYRLVSCNTSVITQACPKVSFEPI  232
24   FMITTDVKDRMRKEYALFYKLDVVPIND-------TRYRLVSCNTSVITQACPKVSFEPI  224
25   FRITTDMQDRTRKEYALFYKLDVVPIND-------TRYRLVSCNTSVITQACPKVSFEPI  232
26   FMITTDVKDRMRKEYALFYKLDVVPIND-------TRYRLVSCNTSVITQACPKVSFEPI  224
27   FMITTDVKDRMRKEYALFYKLDVVPIND-------TRYRLVSCNTSVLTQACPKVSFEPI  224
28   FRITTDMQDRTRKEYALFYKLDVVPIND-------TRYRLVSCNTSVITQACPKVSFEPI  224
29   FRITTDMQDRTRKEYALFYKLDVVPIND-------TRYRLVSCNTSVITQACPKVSFEPI  224
30   FRITTDMQDRTRKEYALFYKLDVVPIND-------TRYRLVSCNTSVITQACPKVSFEPI  224
31   FRITTDMKDRTRKEYALFYKLDVVPIND-------TSYRLVSCNTSVITQACPKVSFEPI  233
```

	Sequence	
	**PIHYCAPAGFAILKCNDKQFNGTGPCTNVSTVQCTHGIRPVVSTQLLLNGSLAEEEVVIR**	
1	PIHYCAPAGFAILKCNDKQFIGTGPCTNVSTVQCTHGIRPVVSTQLLLNGSLAEEEVVIR	269
2	PIHYCAPAGFAILKCNDKQFNGTGPCTNVSTVQCTHGIRPVVSTQLLLNGSLAEEEVVIR	284
3	PIHYCAPAGFAILKCNDKQFNGTGPCTNVSTVQCTHGIRPVVSTQLLLNGSLAEEEVVIR	284
4	PIHYCAPAGFAILKCNDKQFNGTGPCTNVSTVQCTHGIRPVVSTQLLLNGSLAEEEVVIR	276
5	PIHYCAPAGFAILKCNDKQFNGTGPCTNVSTVQCTHGIRPVVSTQLLLNGSLAEEEVVIR	284
6	PIHYCAPAGFAILKCNDKQFNGTGPCTNVSTVQCTHGIRPVVSTQLLLNGSLAEEEVVIR	284
7	PIHYCAPAGFAILKCNDKQFNGTGPCTNVSTVQCTHGIRPVVSTQLLLNGSLAEEEVVIR	292
8	PIHYCAPAGFAILKCNDKQFNGTGPCTNVSTVQCTHGIRPVVSTQLLLNGSLAEEEVVIR	285
9	PIHYCAPAGFAILKCNDKQFNGTGPCTNVSTVQCTHGIRPVVSTQLLLNGSLAEEEVVIR	293
10	PIHYCAPAGFAILKCNDKQFNGTGPCTNVSTVQCTHGIRPVVSTQLLLNGSLAEEEVVIR	284
11	PIHYCAPAGFAILKCNDKQFNGTGPCTNVSTVQCTHGIRPVVSTQLLLNGSLAEEEVVIR	284
12	PIHYCAPAGFAILKCNDKQFNGTGPCTNVSTVQCTHGIRPVVSTQLLLNGSLAEEEVVIR	284
13	PIHYCAPAGFAILKCNDKQFNGTGPCTNVSTVQCTHGIRPVVSTQLLLNGSLAEEEVVIR	284
14	PIHYCAPAGFAILKCNDKQFNGTGPCTNVSTVQCTHGIRPVVSTQLLLNGSLAEEEVVIR	284
15	PIHYCAPAGFAILKCNDKQFNGTGPCTNVSTVQCTHGIRPVVSTQLLLNGSLAEEEVVIR	284
16	PIHYCAPAGFAILKCNDKQFNGTGPCTNVSTVQCTHGIRPVVSTQLLLNGSLAEEEVVIR	284
17	PIHYCAPAGFAILKCNDKQFNGTGPCTNVSTVQCTHGIRPVVSTQLLLNGSLAEEEVVIR	284
18	PIHYCAPAGFAILKCNDKQFNGTGPCTNVSTVQCTHGIRPVVSTQLLLNGSLAEEEVVIR	284
19	PIHYCAPAGFAILKCNDKQFNGTGPCTNVSTVQCTHGIRPVVSTQLLLNGSLAEEEVVIR	284
20	PIHYCAPAGFAILKCNDKQFNGTGPCTNVSTVQCTHGIRPVVSTQLLLNGSLAEEEVVIR	269
21	PIHYCAPAGFAILKCNDKQFNGTGPCTNVSTVQCTHEIRPVVSTQLLLNGSLAEEEVVIR	287
22	PIHYCAPAGFAILKCNDKQFNGTGPCTNVSTVQCTHGIRPVVSTQLLLNGSLAEEEVVIR	284
23	PIHYCAPAGFAILKCNDKQFNGTGPCTNVSTVQCTHGIRPVVSTQLLLNGSLAEEEVVIR	292
24	PIHYCAPAGFAILKCNDKQFNGTGPCTNVSTVQCTHGIRPVVSTQLLLNGSLAEEEVVIR	284
25	PIHYCAPAGFAILKCNDKQFNGTGPCTNVSTVQCTHGIRPVVSTQLLLNGSLAEEEVVIR	292
26	PIHYCAPAGFAILKCNDKQFNGTGPCTNVSTVQCTHGIRPVVSTQLLLNGSLAEEEVVIR	284
27	PIHYCAPAGFAILKCNDKQFNGTGPCTNVSTVQCTHGIRPVVSTQLLLNGSLAEEEVVIR	284
28	PIHYCAPAGFAILKCNDKQFNGTGPCTNVSTVQCTHGIRPVVSTQLLLNGSLAEEEVVIR	284
29	PIHYCAPAGFAILKCNDKQFNGTGPCTNVSTVQCTHGIRPVVSTQLLLNGSLAEEEVVIR	284
30	PIHYCAPAGFAILKCNDKQFNGTGPCTNVSTVQCTHGIRPVVSTQLLLNGSLAEEEVVIR	284
31	PIHYCAPAGFAILKCNDKQFNGTGPCTNVSTVQCTHGIRPVVSTQLLLNGSLAEEEVVIR	293

```
    SVNFSDNAKTIIVQLNKSVEINCTRPNNNTRKSIHMGPGKAFYARGAIIGDIRKAHCNIS
1   SVNFSDNAKTIIVQLNKSVEITCTRPNNNTRKSIPMGPGKAFYARGDITGDIRKAYCEIN  329
2   SVNFSDNAKTIIVQLNKSVEINCTRPNNNTRKSIPMGPGKAFYARGAIIGDIRKAHCNIS  344
3   SVNFSDNAKTIIVQLNKSVEINCTRPNNNTRKSIHMGPGKAFYARGGIIGDIRKAHCNIS  344
4   SVNFSDNAKTIIVQLNKSVEINCTRP--NTRKSIPMGPGKAFYARGAIIGDIRKAHCNIS  334
5   SVNFSDNAKTIIVQLNKSVEINCTRPNNNTRKSIPMGPGKAFYARGAIIGDIRKAHCNIS  344
6   SVNFSDNAKTIIVQLNKSVEINCTRPNNNTRKSIPMGPGKAFYARGAIIGDIRKAHCNIS  344
7   SVNFSDNAKTIIVQLNKSVEINCTRPNNNTRKSIHMGPGKAFYARGAIIGDIRKAHCNIS  352
8   SVNFSDNAKTIIVQLNKSVEINCTRPNNNTRKSIHMGPGKAFYARGAIIGDIRKAHCNIS  345
9   SVNFSDNAKTIIVQLNKSVEINCTRPNNNTRKSIHMGPGKAFYARGAIIGDIRKAHCNIS  353
10  SVNFSDNAKTIIVQLNKSVEINCTRPNNNTRKSIHMGPGKAFYARGGIIGDIRKAHCNIS  344
11  SVNFSDNAKTIIVQLNKSVEINCTRPNNNTRKSIPMGPGKAFYARGAIIGDIRKAHCNIS  344
12  SVNFSDNAKTIIVQLNKSVEINCTRPNNNTRKSIHMGPGKAFYARGGIIGDIRKAHCNIS  344
13  SVNFSDNAKTIIVQLNKSVEINCTRPNNNTRKSIHMGPGKAFYARGGIIGDIRKAHCNIS  344
14  SVNFSDNAKTIIVQLNKSVEINCTRPNNNTRKSIHMGPGKAFYARGGIIGDIRKAHCNIS  344
15  SVNFSDNAKTIIVQLNKSVEINCTRPNNNTRKSIPMGPGKAFYARGAIIGDIRKAHCNIS  344
16  SVNFSDNAKTIIVQLNKSVEINCTRPNNNTRKSIHMGPGKAFYARGGIIGDIRKAHCNIS  344
17  SVNFSDNAKTIIVQLNKSVEINCTRPNNNTRKSIPMGPGKAFYARGAIIGDIRKAHCNIS  344
18  SVNFSDNAKTIIVQLNKSVEINCTRPNNNTRKSIPMGPGKAFYARGAIIGDIRKAHCNIS  344
19  SVNFSDNAKTIIVQLNKSVEINCTRPNNNTRKSIHMGPGKAFYARGGIIGDIRKAHCNIS  344
20  SVNFSDNVKTIIVQLNKSVEINCTRPNNNTRKSIPMGPGKAFYARGNIIGDIRKAYCNIS  329
21  SVNFSDNAKTIIVQLNKSVEINCTRPNNNTRKSIHMGPGKAFYARGGIIGDIRKAYCNVS  347
22  SVNFSDNAKTIIVQLNKSVEINCTRPNNNTRKSIHMGPGKAFYARGGIIGDIRKAHCNIS  344
23  SVNFSDNAKTIIVQLNKSVEINCTRPNNNTRKSIHMGPGKAFYARGAIIGDIRKAHCNIS  352
24  SVNFSDNAKTIIVQLNKSVEINCTRPNNNTRKSIPMGPGKAFYARGAIIGDIRKAHCNIS  344
25  SVNFSDNAKTIIVQLNKSVEINCTRPNNNTRKSIHMGPGKAFYARGAIIGDIRKAHCNIS  352
26  SVNFSDNAKTIIVQLNKSVEINCTRPNNNTRKSIPMGPGKAFYARGAIIGDIRKAHCNIS  344
27  SVNFSDNAKTIIVQLNKSVEINCTRPNNNTRKSIHMGPGKAFYARGGIIGDIRKAHCNIS  344
28  SVNFSDNAKTIIVQLNKSVEINCTRPNNNTRKSIHMGPGKAFYARGGIIGDIRKAHCNIS  344
29  SVNFSDNAKTIIVQLNKSVEINCTRPNNNTRKSIHMGPGKAFYARGGIIGDIRKAH-NIS  343
30  SVNFSDNAKTIIVQLNKSVEINCTRPNNNTRKSIHMGPGKAFYARGAIIGDIRKAHCNIS  344
31  SVNFSDNAKTIIVQLNKSVEINCTRPNNNTRKSIHMGPGKAFYARGAIIGDIRKAHCNIS  353
```

```
    GTEWHSTLKLVVEKLREQY-NKTIVFNHSSGGDPEIVMYSFNCGGEFFYCNSTKLFNSTW
1   GTEWHSTLKLVVEKLREQY-NKTIVFNRSSGGDPEIVMYSFNCGGEFFYCNSTKLFNSTW  388
2   RAEWHSTLKLVAEKLREQY-NKTIVFNHSSGGDPEIVMYSFNCGGEFFYCNSTKLFNSTW  403
3   GTEWHSTLKLVVKKLREQY-NKTIVFNHSSGGDPEIVMYSFNCGGEFFYCNSTKLFNSTW  403
4   GTEWHSTLKLVVEKLREKY-NKTIVFNHSSGGDPEIVMYSFNCGGEFFYCNSTKLFNSTW  393
5   GTEWHNTLKLVVEKLREQY-NKTIVFNHSSGGDPEIVMYSFNCGGEFFYCNSTKLFNSTW  403
6   RAEWHSTLKLVAEKLREQY-NKTIVFNHSSGGDPEIVMYSFNCGGEFFYCNSTKLFNSTW  403
7   GTEWHNTLKLVVEKLREQY-NKTIVFNHSSGGDPEIVMYSFNCGGEFFYCNSTKLFNSTW  411
8   RAEWHSTLKLVAEKLREQY-NKTIVFNHSSGGDPEIVMYSFNCGGEFFYCNSTKLFNSTW  404
9   GTEWHNTLKLVVEKLREQY-NKTIVFNHSSGGDPEIVMYSFNCGGEFFYCNSTKLFNSTW  412
10  GTEWHSTLKLVVKKLREQY-NKTIVFNHSSGGDPEIVMYSFNCGGEFFYCNSTKLFNSTW  403
11  RAEWHSTLKLVAEKLREQY-NKTIVFNHSSGGDPEIVMYSFNCGGEFFYCNSTKLFNSTW  403
12  GTEWHSTLKLVVKKLREQY-NKTIVFNHSSGGDPEIVMYSFNCGGEFFYCNSTKLFNSTW  403
13  GTEWHSTLKLVVKKLREQY-NKTIVFNHSSGGDPEIVMYSFNCGGEFFYCNSTKLFNSTW  403
14  GTEWHSTLKLVGEKLREQY-NKTIVFNHSSGGDPEIVMYSFNCGGEFFYCNSTKLFNSTW  403
15  RAEWHSTLKLVAEKLREQY-NKTIVFNHSSGGDPEIVMYSFNCGGEFFYCNSTKLFNSTW  403
16  GTEWHSTLKLVVKKLREQY-NKTIVFNHSSGGDPEIVMYSFNCGGEFFYCNSTKLFNSTW  403
17  GTEWHNTLKLVVEKLREQY-NKTIVFNHSSGGDPEIVMYSFNCGGEFFYCNSTKLFNSTW  403
18  GTEWHNTLKLVVEKLREQY-NKTIVFNHSSGGDPEIVMYSFNCGGEFFYCNSTKLFNSTW  403
19  RTEWHSTLKLVAEKLREQY-NKTIVFNHSSGGDPEIVMYSFNCGGEFFYCNSTKLFNSTW  403
20  GPEWYSTLKLVVEKLREQY-NKTIVFNHSSGGDPEIVMYSFNCGGEFFYCNSTKLFNSTW  388
21  RTEWHSTLKLVVKKLREKY-NKTIVFNHSSGGDPEIVMYSFNCGGEFFYCNSTKLFNSTW  406
22  RAEWHSTLKLVAEKLREQY-NKTIVFNHSSGGDPEIVMYSFNCGGEFFYCNSTKLFNSTW  403
23  GTEWHNTLKLVVEKLREQY-NKTIVFNHSSGGDPEIVMYSFNCGGEFFYCNSTKLFNSTW  411
24  GTEWHNTLKLVVEKLREQY-NKTIVFNHSSGGDPEIVMYSFNCGGEFFYCNSTKLFNSTW  403
25  GTEWHNTLKLVVEKLREQY-NKTIVFNHSSGGDPEIVMYSFNCGGEFFYCNSTKLFNSTW  411
26  GTEWHNTLKLVVEKLREQY-NKTIVFNHSSGGDPEIVMYSFNCGGEFFYCNSTKLFNSTW  403
27  GTEWHSTLKLVGEKLREQY-NKTIVFNHSSGGDPEIVMYSFNCGGEFFYCNSTKLFNSTW  403
28  GTEWHSTLKLVVKKLREQY-NKTIVFNHSSGGDPEIVMYSFNCGGEFFYCNSTKLFNSTW  403
29  GTEWHSTLKLVVKKLREQY-NKTIVFNHSSGGDPEIVMYSFNCGGEFFYCNSTKLFNSTW  402
30  GTEWHNTLKLVVEKLREQY-NKTIVFNHSSGGDPEIVMYSFNCGGEFFYCNSTKLFNSTW  403
31  RAEWHNTLKLVVEKLREQY-NKTIVFNHSSGGDPEIVMYSFNCGGEFFYCNSTKLFNSTW  412
```

	PWND--TXGSHDTNGTLTLPCRIKQIINMWQGVGKAMYAPPIEGIIKCSSNITGLLLTRD	
1	PWND--TKGSHDTNGTLILPCKIKQIINMWQGVGKAMYAPPIEGKIRCSSNITGLLLTRD	446
2	PWND--TQGSHDTNGTLTLPCRIKQIINMWQEVGKAMYAPPIEGIIKCSSNITGLLLTRD	461
3	PWND--TEGSHDTNGTLTLPCRIKQIINMWQGVGKAMYAPPIEGIIKCSSNITGLLLTRD	461
4	PWND--TEGSHDTNGTLTLPCRIKQIINMWQGVGKAMYAPPIEGIIKCSSNITGLLLTRD	451
5	PWNG--TNGSHDTNGTLTLPCRIKQIINMWQGVGKAMYAPPIEGIIKCSSNITGLLLTRD	461
6	PWND--TNGSHDTNGTLTLPCRIKQIINMWQEVGKAMYAPPIEGIIKCSSNITGLLLTRD	461
7	PGND--TEGSHNTNGTLTLPCRIKQIINMWQEVGKAMYAPPIEGIIKCSSNITGLLLTRD	469
8	--------SGMILKGHMTL---MKQIINMWQEVGKAMYAPPIEGIIKCSSNITGLLLTRD	453
9	PGND--TNGSHDINGTLTLPCRIKQIINMWQGVGKAMYAPPIEGIIKCSSNITGLLLTRD	470
10	PWNDTDTEGSHDTNGTLTLPCRIKQIINMWQGVGKAMYAPPIEGIIKCSSNITGLLLTRD	463
11	PWND--TNGSHDTNGTLTLPCRIKQIINMWQGVGKAMYAPPIEGIIKCSSNITGLLLTRD	461
12	PWNDTDTEGSHDTNGTLTLPCRIKQIINMWQGVGKAMYAPPIEGIIKCSSNITGLLLTRD	463
13	PWNDTDTGGSHDTNGTLTLPCRIKQIINMWQGVGKAMYAPPIEGIIKCSSNITGLLLTRD	463
14	PWND--TNGSHDTNGTLTLPCRIKQIINMWQGVGKAMYAPPIEGIIKCSSNITGLLLTRD	461
15	PWND--TNGSHDTNGTLTLPCRIKQIINMWQGVGKAMYAPPIEGIIKCSSNITGLLLTRD	461
16	PWNDTDTEGSHDTNGTLTLPCRIKQIINMWQGVGKAMYAPPIEGIIKCSSNITGLLLTRD	463
17	PGND--TKGSHDNNGTLTLPCRIKQIINMWQGVGKAMYAPPIEGIIKCSSNITGLLLTRD	461
18	PGND--TNGSHDTNGTLTLPCRIKQIINMWQGVGKAMYAPPIEGIIKCSSNITGLLLTRD	461
19	PWND--TEGSHDTNGTLTLPCRIKQIINMWQGVGKAMYAPPIEGIIKCSSNITGLLLTRD	461
20	PWND--TNGSYDTNDTLMLPCKIKQIINMWQGVGKAMYAPPIEGEIRCSSNITGLLLTRD	446
21	PWND--TNGSHDTNGTLTLPCRIKQIINMWQGVGKAMYAPPIEGIIKCSSNITGLLLTRD	464
22	PWND--TQGSHDTNGTLTLPCRIKQIINMWQEVGKAMYAPPIEGIIKCSSNITGLLLTRD	461
23	PGND--TEGSHNTNGTLTLPCRIKQIINMWQEVGKAMYAPPIEGIIKCSSNITGLLLTRD	469
24	PWNG--TNGSHDTNGTLTLPCRIKQIINMWQGVGKAMYAPPIEGIIKCSSNITGLLLTRD	461
25	PGND--TEGSHNTNGTLTLPCRIKQIINMWQEVGKAMYAPPIEGIIKCSSNITGLLLTRD	469
26	PWNG--TNGSHDTNGTLTLPCRIKQIINMWQGVGKAMYAPPIEGIIKCSSNITGLLLTRD	461
27	PWND--TEGSHDTNGTLTLPCRIKQIINMWQEVGKAMYAPPIEGIIKCSSNITGLLLTRD	461
28	PWNDTDTEGSHDTNGTLTLPCRIKQIINMWQGVGKAMYAPPIEGIIKCSSNITGLLLTRD	463
29	PWNDTDTEGSHDTNGTLTLPCRIKQIINMWQGVGKAMYAPPIEGIIKCSSNITGLLLTRD	462
30	PGND--TEGSHNTNGTLTLPCRIKQIINMWQEVGKAMYAPPIEGIIKCSSNITGLLLTRD	461
31	PGND--TNGSHDINGTLTLPCRIKQIINMWQGVGKAMYAPPIEGIIKCSSNITGLLLTRD	470

	GG-YESNKTDEIFRPGGGDMRDNWRSELYKYKVVKIEPLGVAPTKAKRRVVQREKRAFG-	
1	GG-YESNETDEIFRPGGGDMRDNWRSELYKYKVVKIEPLGVAPTKAKRRVVQREKRAFG-	504
2	GG-YESNKTDEIFRPGGGDMRDNWRSELYKYKVVKIEPLGVAPTKAKRRVVQREKRAFG-	519
3	GG-YESNKTDEIFRPGGGDMRDNWRSELYKYKVVKIEPLGVAPTKAKRRVVQREKRAFG-	519
4	GG-YESNKTDEIFRPGGGDMRDNWRSELYKYKVVKIEPLGVAPTKAKRRVVQREKRAFG-	509
5	GG-YESNKTDEIFRPGGGDMRDNWRSELYKYKVVKIEPLGVAPTKAKRRVVQREKRAFG-	519
6	GG-YESNKTDEIFRPGGGDMRDNWRSELYKYKVVKIEPLGVAPTKAKRRVVQREKRAFG-	519
7	GG-YESNKTDEIFRPGGGDMRDNWRSELYKYKVVKIEPLGVAPTKAKRRVVQREKRAFG-	527
8	GG-YESNKTDEIFRPGGGDMRDNWRSELYKYKVVKIEPLGVAPTKAKRRVVQREKRAFG-	511
9	GG-YESNKTDEIFRPGGGDMRDNWRSELYKYKVVKIEPLGVAPTKAKRRVVQREKRAFG-	528
10	GG-YESNKTDEIFRPGGGDMRDNWRSELYKYKVVKIEPLGVAPTKAKRRVVQREKRAFG-	521
11	GG-YESNKTDEIFRPGGGDMRDNWRSELYKYKVVKIEPLGVAPTKAKRRVVQREKRAFG-	519
12	GG-YESNKTDEIFRPGGGDMRDNWRSELYKYKVVKIEPLGVAPTKAKRRVVQREKRAFG-	521
13	GG-YESNKTDEIFRPGGGDMRDNWRSELYKYKVVKIEPLGVAPTKAKRRVVQREKRAFG-	521
14	GG-YESNKTDEIFRPGGGDMRDNWRSELYKYKVVKIEPLGVAPTKAKRRVVQREKRAFG-	519
15	GG-YESNKTDEIFRPGGGDMRDNWRSELYKYKVV-IEPLGVAPTKAKRRVVQREKRAFG-	518
16	GG-YESNKTDEIFRPGGGDMRDNWRSELYKYKVVKIEPLGVAPTKAKRRVVQREKRAFG-	521
17	GG-YESNKTDEIFRPGGGDMRDNWRSELYKYKVVKIEPLGVAPTKAKRRVVQREKRAFG-	519
18	GG-YESNKTDEIFRPGGGDMRDNWRSELYKYKVVKIEPLGVAPTKAKRRVVQREKRAFG-	519
19	GG-YESNKTDEIFRPGGGDMRDNWRSELYKYKVVKIEPLGVAPTKAKRRVVQREKRAFG-	519
20	GG-YESNETDEIFRPGGGDMRDNWRSELYKYKVVKIEPLGVAPTKAKRRVVQREKRAFG-	504
21	GG-YESNKTDEIFRPGGGDMRDNWRSELYKYKVVKIEPLGVAPAKAKRRVVQREKRAFG-	522
22	GG-YESNKTDEIFRPGGGDMRDNWRSELYKYKVVKIEPLGVAPTKAKRRVVQREKRAFG-	519
23	GG-YESNKTDEIFRPGGGDMRDNWRSELYKYKVVKIEPLGVAPTKAKRRVVQREKRAFG-	527
24	GG-YESNKTDEIFRPGGGDMRDNWRSELYKYKVVKIEPLGVAPTKAKRRVVQREKRAFG-	519
25	GG-YESNKTDEIFRPGGGDMRDNWRSELYKYKVVKIEPLGVAPTKAKRRVVQREKRAFG-	527
26	GG-YESNKTDEIFRPGGGDMRDNWRSELYKYKVVKIEPLGVAPTKAKRRVVQREKRAFG-	519
27	GG-YESNKTDEIFRPGGGDMRDNWRSELYKYKVVKIEPLGVAPTKAKRRVVQREKRAFG-	519
28	GG-YESNKTDEIFRPGGGDMRDNWRSELYKYKVVKIEPLGVAPTKAKRRVVQREKRAFG-	521
29	GG-YESNKTDEIFRPGGGDMRDNWRSELYKYKVVKIEPLGVAPTKAKRRVVQREKRAFG-	520
30	GG-YESNKTDEIFRPGGGDMRDNWRSELYKYKVVKIEPLGVAPTKAKRRVVQREKRAFG-	519
31	GG-YESNKTDEIFRPGGGDMRDNWRSELYKYKVVKIEPLGVAPTKAKRRVVQREKRAFG-	528

	**LGAVFLGFLGAAGSTMGAASITLTVQARQLLSGIVQQQNNLLRAIEAQQHLLQLTVWGIK**	
1	LGAVFLGFLGAAGSTMGAASITLTVQARQLLSGIVQQQNNLLRAIEAQQHLLQLTVWGIK	564
2	LGAVFLGFLGAAGSTMGAASITLTVQARQLLSGIVQQQNNLLRAIEAQQHLLQLTVWGIK	579
3	LGAVFLGFLGAAGSTMGAASITLTVQARQLLSGIVQQQNNLLRAIEAQQHLLQLTVWGIK	579
4	LGAVFLGFLGAAGSTMGAASITLTVQARQLLSGIVQQQNNLLRAIEAQQHLLQLTVWGIK	569
5	LGAVFLGFLGAAGSTMGAASITLTVQARQLLSGIVQQQNNLLRAIEAQQHLLQLTVWGIK	579
6	LGAVFLGFLGAAGSTMGAASITLTVQARQLLSGIVQQQNNLLRAIEAQQHLLQLTVWGIK	579
7	LGAVFLGFLGAAGSTMGAASITLTVQARQLLSGIVQQQNNLLRAIEAQQHLLQLTVWGIK	587
8	LGAVFLGFLGAAGSTMGAASITLTVQARQLLSGIVQQQNNLLRAIEAQQHLLQLTVWGIK	571
9	LGAVFLGFLGAAGSTMGAASITLTVQARQLLSGIVQQQNNLLRAIEAQQHLLQLTVWGIK	588
10	LGAVFLGFLGAAGSTMGAASITLTVQARQLLSGIVQQQNNLLRAIEAQQHLLQLTVWGIK	581
11	LGAVFLGFLGAAGSTMGAASITLTVQARQLLSGIVQQQNNLLRAIEAQQHLLQLTVWGIK	579
12	LGAVFLGFLGAAGSTMGAASITLTVQARQLLSGIVQQQNNLLRAIEAQQHLLQLTVWGIK	581
13	LGAVFLGFLGAAGSTMGAASITLTVQARQLLSGIVQQQNNLLRAIEAQQHLLQLTVWGIK	581
14	LGAVFLGFLGAAGSTMGAASITLTVQARQLLSGIVQQQNNLLRAIEAQQHLLQLTVWGIK	579
15	LGAVFLGFLGAAGSTMGAASITLTVQARQLLSGIVQQQNNLLRAIEAQQHLLQLTVWGIK	578
16	LGAVFLGFLGAAGSTMGAASITLTVQARQLLSGIVQQQNNLLRAIEAQQHLLQLTVWGIK	581
17	LGAVFLGFLGAAGSTMGAASITLTVQARQLLSGIVQQQNNLLRAIEAQQHLLQLTVWGIK	579
18	LGAVFLGFLGAAGSTMGAASITLTVQARQLLSGIVQQQNNLLRAIEAQQHLLQLTVWGIK	579
19	LGAVFLGFLGAAGSTMGAASITLTVQARQLLSGIVQQQNNLLRAIEAQQHLLQLTVWGIK	579
20	LGAVFLGFLGAAGSTMGAASITLTVQARQLLSGIVQQQNNLLRAIEAQQHLLQLTVWGIK	564
21	LGAVFLGFLGAAGSTMGAASITLTVQARQLLSGIVQQQNNLLRTIEAQQHLLQLTVWGIK	582
22	LGAVFLGFLGAAGSTMGAASITLTVQARQLLSGIVQQQNNLLRAIEAQQHLLQLTVWGIK	579
23	LGAVFLGFLGAAGSTMGAASITLTVQARQLLSGIVQQQNNLLRAIEAQQHLLQLTVWGIK	587
24	LGAVFLGFLGAAGSTMGAASITLTVQARQLLSGIVQQQNNLLRAIEAQQHLLQLTVWGIK	579
25	LGAVFLGFLGAAGSTMGAASITLTVQARQLLSGIVQQQNNLLRAIEAQQHLLQLTVWGIK	587
26	LGAVFLGFLGAAGSTMGAASITLTVQARQLLSGIVQQQNNLLRAIEAQQHLLQLTVWGIK	579
27	LGAVFLGFLGAAGSTMGAASITLTVQARQLLSGIVQQQNNLLRAIEAQQHLLQLTVWGIK	579
28	LGAVFLGFLGAAGSTMGAASITLTVQARQLLSGIVQQQNNLLRAIEAQQHLLQLTVWGIK	581
29	LGAVFLGFLGAAGSTMGAASITLTVQARQLLSGIVQQQNNLLRAIEAQQHLLQLTVWGIK	580
30	LGAVFLGFLGAAGSTMGAASITLTVQARQLLSGIVQQQNNLLRAIEAQQHLLQLTVWGIK	579
31	LGAVFLGFLGAAGSTMGAASITLTVQARQLLSGIVQQQNNLLRAIEAQQHLLQLTVWGIK	588

	QLQARVLAVERYLKDQQLLGIWGCSGKLICTTTVPWNTSWSNKSLEQIWDNMTWMEWERE	
1	QLQARVLAVERYLKDQQLLGIWGCSGKLICTTTVPWNTSWSNKSLEQIWDNMTWMEWERE	624
2	QLQARVLAVERYLKDQQLLGIWGCSGKLICTTTVPWNTSWSNKSLEQIWDNMTWMEWEKE	639
3	QLQARVLAVERYLKDQQLLGIWGCSGKLICTTTVPWNTSWSNKSLEQIWDNMTWMEWERE	639
4	QLQARVLAVERYLKDQQLLGIWGCSGKLICTTTVPWNTSWSNKSLEQIWDNMTWMEWERE	629
5	QLQARVLAVERYLKDQQLLGIWGCSGKLICTTTVPWNTSWSNKSLEQIWDNMTWMEWERE	639
6	QLQARVLAVERYLKDQQLLGIWGCSGKLICTTTVPWNTSWSNKSLEQIWDNMTWMEWERE	639
7	QLQARVLAVERYLKDQQLLGIWGCSGKLICTTTVPWNTSWSNKSLEQIWDKMTWMEWERE	647
8	QLQARVLAVERYLKDQQLLGIWGCSGKLICTTTVPWNTSWSNKSLEQIWDNMTWMEWERE	631
9	QLQARVLAVERYLKDQQLLGIWGCSGKLICTTTVPWNTSWSNKSLEQIWDKMTWMEWERE	648
10	QLQARVLAVERYLKDQQLLGIWGCSGKLICTTTVPWNTSWSNKSLEQIWDNMTWMEWERE	641
11	QLQARVLAVERYLKDQQLLGIWGCSGKLICTTTVPWNTSWSNKSLEQIWDNMTWMEWERE	639
12	QLQARVLAVERYLKDQQLLGIWGCSGKLICTTTVPWNTSWSNKSLEQIWDKMTWMEWERE	641
13	QLQARVLAVERYLKDQQLLGIWGCSGKLICTTTVPWNTSWSNKSLEQIWDKMTWMEWERE	641
14	QLQARVLAVERYLKDQQLLGIWGCSGKLICTTTVPWNTSWSNKSLEQIWDNMTWMEWERE	639
15	QLQARVLAVERYLKDQQLLGIWGCSGKLICTTTVPWNTSWSNKSLEQIWDNMTWMEWERE	638
16	QLQARVLAVERYLKDQQLLGIWGCSGKLICTTTVPWNTSWSNKSLEQIWDKMTWMEWERE	641
17	QLQARVLAVERYLKDQQLLGIWGCSGKLICTTTVPWNTSWSNKSLEQIWDNMTWMEWERE	639
18	QLQARVLAVERYLKDQQLLGIWGCSGKLICTTTVPWNTSWSNKSLEQIWDNMTWMEWERE	639
19	QLQARVLAVERYLKDQQLLGIWGCSGKLICTTTVPWNTSWSNKSLEQIWDNMTWMEWERE	639
20	QLQARVLAVERYLKDQQLLGIWGCSGKLICTTTVPWNTSWSNKSLEQIWDNMTWMEWERE	624
21	QLQARVLAVERYLKDQQLLGIWGCSGKLICTTTVPWNTSWSNKSLEQIWDNMTWMEWKRE	642
22	QLQARVLAVERYLKDQQLLGIWGCSGKLICTTTVPWNTSWSNKSLEQIWDNMTWMEWERE	639
23	QLQARVLAVERYLKDQQLLGIWGCSGKLICTTTVPWNTSWSNKSLEQIWDNMTWMEWERE	647
24	QLQARVLAVERYLKDQQLLGIWGCSGKLICTTTVPWNTSWSNKSLEQIWDNMTWMEWERE	639
25	QLQARVLAVERYLKDQQLLGIWGCSGKLICTTTVPWNTSWSNKSLEQIWDKMTWMEWERE	647
26	QLQARVLAVERYLKDQQLLGIWGCSGKLICTTTVPWNTSWSNKSLEQIWDKMTWMEWERE	639
27	QLQARVLAVERYLKDQQLLGIWGCSGKLICTTTVPWNTSWSNKSLEQIWDKMTWMEWERE	639
28	QLQARVLAVERYLKDQQLLGIWGCSGKLICTTTVPWNTSWSNKSLEQIWDNMTWMEWERE	641
29	QLQARVLAVERYLKDQQLLGIWGCSGKLICTTTVPWNTSWSNKSLEQIWDKMTWMEWERE	640
30	QLQARVLAVERYLKDQQLLGIWGCSGKLICTTTVPWNTSWSNKSLEQIWDKMTWMEWERE	639
31	QLQARVLAVERYLKDQQLLGIWGCSGKLICTTTVPWNTSWSNKSLEQIWDKMTWMEWERE	648

	**IDNYTGYIYQLIEESQNQQEKNEQELLALDKWASLWNWFDITNWLWYIKIFIMIVGGLIG**	
1	IDNYTGYIYQLIEESQNQQEKNEQELLALDKWASLWNWFDITNWLWYIKIFIMIVGGLIG	684
2	INNYTGYIYQLIEESQNQQEKNEQELLAWDKWASLWNWFDITNWLWYIKIFIMIVGGLIG	699
3	INNYTGYIYQLIEESQNQQEKNEQELLAWDKWASLWNWFDITNWLWYIKIFIMIVGGLIG	699
4	IDNYTGYIYQLIEESQNQQEKNEQELLALDKWASLWNWFDITNWLWYIKIFIMIVGGLIG	689
5	INNYTGYIYQLIEESQNQQEKNEQELLAWDKWASLWNWFDITNWLWYIKIFIMIVGGLIG	699
6	INNYTGYIYQLIEESQNQQEKNEQELLALDKWASLWNWFDITNWLWYIKIFIMIVGGLIG	699
7	IDNYTDYIYQLIEESQNQQEKNEQELLALDKWASLWNWFDITNWLWYIKIFIMIVGGLIG	707
8	INNYTGYIYQLIEESQNQQEKNEQELLALDKWASLWNWFDITNWLWYIKIFIMIVGGLIG	691
9	IDNYTDYIYQLIEESQNQQEKNEQELLALDKWASLWNWFDITNWLWYIKIFIMIVGGLIG	708
10	IDNYTDYIYQLIEESQNQQEKNEQELLALDKWASLWNWFDITKWLWYIKIFIMIVGGLIG	701
11	INNYTGYIYQLIEESQNQQEKNEQELLALDKWASLWNWFDITNWLWYIKIFIMIVGGLIG	699
12	IDNYTDYIYQLIEESQNQQEKNEQELLALDKWASLWNWFDITNWLWYIKIFIMIVGGLIG	701
13	INNYTGYIYQLIEESQNQQEKNEQELLALDKWASLWNWFDITNWLWYIKIFIMIVGGLIG	701
14	INNYTGYIYQLIEESQNQQEKNEQELLALDKWASLWNWFDITNWLWYIKIFIMIVGGLIG	699
15	INNYTGYIYQLIEESQNQQEKNEQELLAWDKWASLWNWFDITNWLWYIKIFIMIVGGLIG	698
16	IDNYTDYIYQLIEESQNQQEKNEQELLALDKWASLWNWFDITNWLWYIKIFIMIVGGLIG	701
17	INNYTGYIYQLIEESQNQQEKNEQELLAWDKWASLWNWFDITNWLWYIKIFIMIVGGLIG	699
18	INNYTGYIYQLIEESQNQQEKNEQELLAWDKWASLWNWFDITNWLWYIKIFIMIVGGLIG	699
19	INNYTGYIYQLIEESQNQQEKNEQELLAWDKWASLWNWFDITNWLWYIKIFIMIVGGLIG	699
20	IDNYTGYIYQLIEESQNQQEKNEQELLALDKWASLWNWFDITNWLWYIKIFIMIVGGLIG	684
21	INNYTGYIYQLIEESQNQQEKNEQELLAWDKWASL-NWFDITNWLWYIKIFIMIVGGLIG	701
22	INNYTGYIYQLIEESQNQQEKNEQELLALDKWASLWNWFDITNWLWYIKIFIMIVGGLIG	699
23	IDNYTGYIYQLIEESQNQQEKNEQELLALDKWASLWNWFDITNWLWYIKIFIMIVGGLIG	707
24	INNYTGYIYQLIEESQNQQEKNEQELLAWDKWASLWNWFDITNWLWYIKIFIMIVGGLIG	699
25	IDNYTDYIYQLIEESQNQQEKNEQELLALDKWASLWNWFDITNWLWYIKIFIMIVGGLIG	707
26	IDNYTDYIYQLIEESQNQQEKNEQELLALDKWASLWNWFDITNWLWYIKIFIMIVGGLIG	699
27	IDNYTDYIYQLIEESQNQQEKNEQELLALDKWASLWNWFDITNWLWYIKIFIMIVGGLIG	699
28	IDNYTGYIYQLIEESQNQQEKNEQELLAWDKWASLWNWFDITNWLWYIKIFIMIVGGLIG	701
29	IDNYTDYIYQLIEESQNQQEKNEQELLALDKWASLWNWFDITNWLWYIKIFIMIVGGLIG	700
30	IDNYTDYIYQLIEESQNQQEKNEQELLALDKWASLWNWFDITNWLWYIKIFIMIVGGLIG	699
31	IDNYTDYIYQLIEESQNQQEKNEQELLALDKWASLWNWFDITNWLWYIKIFIMIVGGLIG	708

	LRIVFTVLSIVNRVRQGYSPLSFQTHLPAQRGPDRPEGIGEEGGERDRDRSDPLVNGFLT	
1	LRIVFTVLSIVNRVRQGYSPLSFQTHLPAQRGPDRPEGIGEEGGERDRDRSDPLVNGFLA	744
2	LRIVFTVLSIVNRVRQGYSPLSFQTHLPAQRGPDRPEGIGEEGGERDRDRSDPLVNGFLT	759
3	LRIVFTVLSIVNRVRQGYSPLSFQTHLPAQRGPDRPEGIGEEGGERDRDRSDPLVNGFLT	759
4	LRIVFTVLSIVNRVRQGYSPLSFQTHLPAQRGLDRPEGIGEEGGERDRDRSDPLVNGFLT	749
5	LRIVFTVLSIVNRVRQGYSPLSFQTHLPAQRGPDRPEGIGEEGGERDRDRSDPLVNGFLT	759
6	LRIVFTVLSIVNRVRQGYSPLSFQTHLPAQRGPDRPEGIGEEGGERDRDRSDPLVNGFLT	759
7	LRIVFTVLSIVNRVRQGYSPLSFQTHLPAQRGPDRPEGIGEEGGERDRDRSDPLVNGFLT	767
8	LRIVFTVLSIVNRVRQGYSPLSFQTHLPAQRGPDRPEGIGEEGGERDRDRSDPLVNGFLT	751
9	LRIVFTVLSIVNRVRQGYSPLSFQTHLPAQRGPDRPEGIGEEGGERDRDRSDPLVNGFLT	768
10	LRIVFTVLSIVNRVRQGYSPLSFQTHFPAQRGPDRPEGIGEEGGERDRDRSDPLVNGFLT	761
11	LRIVFTVLSIVNRVRQGYSPLSFQTHFPAQRGPDRPEGIGEEGGERDRDRSDPLVNGFLT	759
12	LRIVFTVLSIVNRVRQGYSPLSFQTHFPAQRGPDRPEGIGEEGGERDRDRSDPLVNGFLT	761
13	LRIVFTVLSIVNRVRQGYSPLSFQTHLPAQRGPDRPEGIGEEGGERDRDRSDPLVNGFLT	761
14	LRIVFTVLSIVNRVRQGYSPLSFQTHLPAQRGPDRPEGIGEEGGERDRDRSAPLVNGFLT	759
15	LRIVFTILSIVNRVRQGYSPLSFQTHLPAQREPDRPEGIGEEGGERDRDRSDPLVNGFLT	758
16	LRIVFTVLSIVNRVRQGYSPLSFQTHFPAQRGPDRPEGIGEEGGERDRDRSDPLVNGFLT	761
17	LRIVFTVLSIVNRVRQGYSPLSFQTHLPAQRGPDRPEGIGEEGGERDRDRSDPLVNGFLT	759
18	LRIVFTVLSIVNRVRQGYSPLSFQTHLPAQRGPDRPEGIGEEGGERDRDRSDPLVNGFLT	759
19	LRIVFTVLSIVNRVRQGYSPLSFQTHFPAQRGPDRPEGIGEEGGERDRDRSDPLVNGFLT	759
20	LRIVFTVLSIVNRVRQGYSPLSFQTHLPAQRGPDRPEGIGEEGGERDRDRSDPLVNGFLT	744
21	LRIVFTVLSIVNRVRQGYSPLSFQTHFPAQRGPDRPEEIGEDGGERDRDRSDPLVNGFLT	761
22	LRIVFTVLSIVNRVRQGYSPLSFQTHLPAQRGPDRPEGIGEEGGERDRDRSDPLVNGFLT	759
23	LRIVFTVLSIVNRVRQGYSPLSFQTHFPAQRGPDRPEGIGEEGGERDRDRSDPLVNGFLT	767
24	LRIVFTVLSIVNRVRQGYSPLSFQTHLPAQRGPDRPEGIGEEGGERDRDRSDPLVNGFLT	759
25	LRIVFTVLSIVNRVRQGYSPLSFQTHLPAQRGPDRPEGIGEEGGERDRDRSDPLVNGFLT	767
26	LRIVFTVLSIVNRVRQGYSPLSFQTHFPAQRGPDRPEGIGEEGGERDRDRSDPLVNGFLT	759
27	LRIVFTVLSIVNRVRQGYSPLSFQTHFPAQRGPDRPEGIGEEGGERDRDRSDPLVNGFLT	759
28	LRIVFTVLSVVNRVRQGYSPLSFQTHLPAQREPDRPEGIGEEGGERDRDRSDPLVNGFLT	761
29	LRIVFTVLSIVNRVRQGYSPLSFQTHLPAQRGPDRPEGIGEEGGERDRDRSDPLVNGFLT	760
30	LRIVFTVLSIVNRVRQGYSPLSFQTHLPAQRGPDRPEGIGEEGGERDRDRSDPLVNGFLT	759
31	LRIVFTVLSIVNRVRQGYSPLSFQTHLPAQRGPDRPEGIGEEGGERDRDRSDPLVNGFLT	768

```
    LIWSDLRSLCLFSYHHLRDLLLIVTRIVELLGR-------RGWEVLKYWWNLLQYWSQEL
1   LIWSDLRSLCLFSYHRLRDLLLIVTRIVELLGR-------RGWEVLKYWWNLLQYWSQEL 797
2   LIWSDLRSLCLFSYHRLRDLLLIATRIVELLGR-------RGWEVLKYWWNLLQYWSQEL 812
3   LIWSDLRSLCLFSYHHLRDLLLIVTRIVELLGR-------RGWEVLKYWWNLLQYWSQEL 812
4   LIWSDLRSLCLFSYHHLRDLLLIVTRIVELLGR-------RGWEVLKYWWNLLQYWSQEL 802
5   LIWSDLRSLCLFSYHHLRDLLLIVTRIVELLGR-------RGWEVLKYWWNLLQYWSQEL 812
6   LIWSDLRSLCLFSYHRLRDLLLIATRIVELLGR-------RGWEVLKYWWNLLQYWSQEL 812
7   LIWSDLRSLCLFSYHRLRDLLLIATRIVELLGR-------RGWEVLKYWWNLLQYWSQEL 820
8   LIWSDLRSLCLFSYHHLRDLLLIVTRIVELLGR-------RGWEVLKYWWNLLQYWSQEL 804
9   LIWSDLRSLCLFSYHHLRDLLLIVTRIVELLGR-------RGWEVLKYWWNLLQYWSQEL 821
10  LIWSDLRSLCLFSYHRLRDLLLIATRIVELLGR-------RGWEVLKYWWNLLQYWSQEL 814
11  LIWSDLRSLCLCSYHRLRDLLLIATRIVELLGR-------RGWEVLKYWWNLLQYWSQEL 812
12  LIWSDLRSLCLFSYHRLRDLLLIATRIVELLGR-------RGWEVLKYWWNLLQYWSQEL 814
13  LIWSDLRSLCLFSYHHLRDLLLIVTRIVELLGR-------RGWEVLKYWWNLLQYWSQEL 814
14  LIWSDLRSLCLFSYHHLRDSLLIVTRIVELLGR-------RGWEVLKYWWNLLQYWSQEI 812
15  LIWSDLRSLCLFSYHRLRDLLLIATRIVELLGR-------RGWEVLKYWWNLLQYWSQEL 811
16  LIWSDLRSLCLFSYHHLRDLLLIVTRIVELLGR-------RGWEVLKYWWNLLQYWSQEL 814
17  LIWSDLRSLCLFSYHRLRDLLLIATRIVELLGR-------RGWEVLKYWWNLLQYWSQEL 812
18  LIWSDLRSLCLFSYHRLRDLLLIATRIVELLGR-------RGWEVLKYWWNLLQYWSQEL 812
19  LIWSDLRSLCLFSYHRLRDLLLIATRIVELLGR-------RGWEVLKYWWNLLQYWSQEL 812
20  LIWSDLRSLCLFSYHHLRDLLLIVTRIVELLGR-------RGWEVLKYWWNLLQYWSQEL 797
21  LIWSDLRSLCLFSYHRLRDLLLIATRIVELLGH-------RGWEVLKYWWNLLQYWSQEL 814
22  LIWSDLRSLCLFSYHHLRDLLLIVTRIVELLGR-------RGWEVLKYWWNLLQYWSQEL 812
23  LIWSDLRSLCLFSYHRLRDLLLIATRIVELLGR-------RGWEVLKYWWNLLQYWSQEL 820
24  LIWSDLRSLCLFSYHHLRDLLLIVTRIVELLGR-------RGWEVLKYWWNLLQYWSQEL 812
25  LIWSDLRSLCLFSYHRLRDLLLIATRIVELLGR-------RGWEVLKYWWNLLQYWSQEL 820
26  LIWSDLRSLCLFSYHHLRDLLLIVTRIVELLGR-------RGWEVLKYWWNLLQYWSQEI 812
27  LIWSDLRSLCLFSYHHLRDLLLIVTRIVELLGR-------RGWEVLKYWWNLLQYWSQEL 812
28  LIWSDLRSLCLFSYHHLRDLLLIVTRIVELLGR-------RGWEVLKYWWNLLQYWSQEI 814
29  LIWSDLRSLCLFSYHRLRDLLLIATRIVELLGR-------RGWEVLKYWWNLLQYWSQEL 813
30  LIWSDLRSLCLFSYHHLRDLLLIVTRIVELLGR-------RGWEVLKYWWNLLQYWSQEL 812
31  LIWSDLRSLCLFSYHHLRDLLLIVTRIVELLGR-------RGWEVLKYWWNLLQYWSQEL 821
```

	KNSAVSLLNATAIAVAEGTDRVIEVVQRAGRAIRHIPRRIRQGLERALL	
1	KNSAVSLLNATAIAVAEGTDRVIEVVQRACRAILHIPRRIRQGLERALL	846
2	KKSAVSLLNATAIAVAEGTDRVIEVVQRAGRAIRHIPRRIRQGLERALL	861
3	KNSAISLLNATAIAVAEGTDRVIEVVQRAGRAIRHIPRRIRQGLERALL	861
4	KNSAVSLFNATAIAVAEGTDRVIEVVQRAGRAIRHIPRRIRQGLERALL	851
5	KDSAVSLLNATAIAVAEGTDRVIEVVQRAGRAIRHIPRRIRQGLERALL	861
6	KNSAVSLLNATAIAVAEGTDRVIEVVQRAGRAIRHIPRRIRQGLERALL	861
7	KNSAVSLLNATAIAVAEGTDRVIEVVQRAGRAIRHIPRRIRQGLERALL	869
8	KKSAVSLLNATAIAVAEGTDRVIEVVQRAGRAIRHIPRRIRQGLERALL	853
9	KNSAVSLLNATAIAVAEGTDRVIEVVQRAGRAIRHIPRRIRQGLERALL	870
10	KSSAVSLLNATAIAVAEGTDRVIEVVQRAGRAIRHIPRRIRQGLERALL	863
11	KSSAVSLLNATAIAVAEGTDRVIEVVQRAGRAIRHIPRRIRQGLERALL	861
12	KSSAVSLLNATAIAVAEGTDRVIEVVQRAGRAIRHIPRRIRQGLERALL	863
13	KNSAVSLLNATAIAVAEGTDRVIEVVQRAGRAIRHIPRRIRQGLERALL	863
14	KNSAVSLLNATATAVAEGTDRVIEVVQRAGRAIRHIPRRIRQGLERALL	861
15	KDSAVSLLNATATAVAEGTDRVIEVVQRAGRAIRHIPRRIRQGLERALL	860
16	KNSAVSLLNATAIAVAEGTDRVIEVVQRAGRAIRHIPRRIRQGLERALL	863
17	KNSAVSLFNATAIAVAEGTDRVIEVVQRAGRAIRHIPRRIRQGLERALL	861
18	KNSAVSLLNATAIAVAEGTDRVIEVVQRAGRAIRHIPRRIRQGLERALL	861
19	KNSAGSLLNATAIAVAEGTDRVIEVVQRAGRAIRHIPRRIRQGLERALL	861
20	KNSAVSLLNATAIAVAEGTDRVIEVVQRAGRAIRHIPRRIRQGLERALL	846
21	KKSAVSLLNATAIAVAEGTDRVIEVVQRAGRAIRHIPRRIRQGLERALL	863
22	KNSAVSLLNATAIAVAEGTDRVIEVVQRAGRAIRHIPRRIRQGLERVLL	861
23	RNSAVSLLNATAIAVAEGTDRVIEVVQRAGRAIRHIPRRIRQGLERALL	869
24	KDSAVSLLNATAIAVAEGTDRVIEVVQRAGRAIRHIPRRIRQGLERALL	861
25	KNSAVSLLNATAIAVAEGTDRVIEVVQRAGRAIRHIPRRIRQGLERALL	869
26	KNSAISLLNATAIAVAEGTDRVIEVVQRAGRAIRHIPRRIRQGLERALL	861
27	KNSAVNLLNATAIAVAEGTDRVIEVVQRAGRAIRHIPRRIRQGLERALL	861
28	KNSAVSLLNATAIAVAEGTDRVIEVVQRAGRAIRHIPRRIRQGLERALL	863
29	KSSAVSLLNATAIAVAEGTDRVIEVVQRAGRAIRHIPRRIRQGLERALL	862
30	KNSAVNLLNATAIAIAEGTDRVIEVVQRAGRAIRHIPRRIRQGLERALL	861
31	KNSAVSLLNATAIAVAEGTDRVIEVVQRAGRAIRHIPRRIRQGLERALL	870

Consensus

1. B.US.2006.700010040_C9_4520.EU289193
2. B.US.2010.CH0040_3_d1597_ipe032_15_28.MG900512
3. B.US.2010.CH0040_3_d1597_ipe032_15_29.MG900513
4. B.US.2010.CH0040_3_d1597_ipe032_15_34.MG900514
5. B.US.2010.CH0040_3_d1597_ipe032_15_38.MG900515
6. B.US.2010.CH0040_3_d1597_ipe032_15_40.MG900516
7. B.US.2010.CH0040_3_d1597_ipe032_15_42.MG900517
8. B.US.2010.CH0040_3_d1597_ipe032_15_43.MG900518
9. B.US.2010.CH0040_3_d1597_ipe032_15_44.MG900519
10. B.US.2010.CH0040_3_d1597_ipe032_15_46.MG900520
11. B.US.2010.CH0040_3_d1597_ipe032_15_49.MG900521
12. B.US.2010.CH0040_3_d1597_ipe032_15_50.MG900522
13. B.US.2010.CH0040_3_d1597_ipe032_15_52.MG900523
14. B.US.2010.CH0040_3_d1597_ipe032_15_53.MG900524
15. B.US.2010.CH0040_3_d1597_ipe032_15_54.MG900525
16. B.US.2010.CH0040_3_d1597_ipe032_15_55.MG900526
17. B.US.2010.CH0040_3_d1597_ipe032_15_56.MG900527
18. B.US.2010.CH0040_3_d1597_ipe032_15_57.MG900528
19. B.US.2010.CH0040_3_d1597_ipe032_15_60.MG900529
20. B.US.2010.CH0040_3_d1597_ipe032_15_65.MG900530
21. B.US.2010.CH0040_3_d1597_ipe032_15_66.MG900531
22. B.US.2010.CH0040_3_d1597_ipe032_15_68.MG900532
23. B.US.2010.CH0040_3_d1597_ipe032_15_69.MG900533
24. B.US.2010.CH0040_3_d1597_ipe032_15_70.MG900534
25. B.US.2010.CH0040_3_d1597_ipe032_25_10.MG900535
26. B.US.2010.CH0040_3_d1597_ipe032_25_13.MG900536
27. B.US.2010.CH0040_3_d1597_ipe032_25_21.MG900537
28. B.US.2010.CH0040_3_d1597_ipe032_25_22.MG900538
29. B.US.2010.CH0040_3_d1597_ipe032_25_26.MG900539
30. B.US.2010.CH0040_3_d1597_ipe032_25_27.MG900540
31. B.US.2010.CH0040_3_d1597_ipe032_25_4.MG900541
32. B.US.2010.CH0040_3_d1597_ipe032_25_5.MG900542
33. B.US.2010.CH0040_3_d1597_ipe032_25_6.MG900543
34. B.US.2010.CH0040_3_d1597_ipe032_25_8.MG900544
35. B.US.2010.CH0040_3_d1597_ipe032_27_01.MG900545

	MRVMGIRKNYQHLWREGILLLGILMICSAADNLWVTVYYGVPVWREATTTLFCASDAKAY	
1	MRVMGIRKNYQHLWREGILLLGILMICSAADNLWVTVYYGVPVWREATTTLFCASDAKAY	60
2	MRVMGIRKNYQHWWREGILLLGILMICSAADKLWVTVYYGVPVWREATTTLFCASDAKAY	60
3	MRVMGIRKNYQHLWREGILLLGILMICSAADNLWVTVYYGVPVWREATTTLFCASDAKAY	60
4	MRVMGIRKNYQHLWREGILLLGILMICSAADNLWVTVYYGVPVWREATTTLFCASDAKAY	60
5	MRVMGIRKNYQHWWREGILLLGILMICSAADKLWVTVYYGVPVWREATTTLFCASDAKAY	60
6	MRVMGIRKNYQHLWREGILLLGILMICSAADNLWVTVYYGVPVWREATTTLFCASDAKAY	60
7	MRVMGIRKNYQHLWREGILLLGILMICSAADNLWVTVYYGVPVWREATTTLFCASDAKAY	60
8	MRVMGIRKNYQHLWREGILLLGILMICSAADNLWVTVYYGVPVWREATTTLFCASDAKAY	60
9	MRVMGIRKNYQHLWREGILLLGILMICSAADNLWVTVYYGVPVWREATTTLFCASDAKAY	60
10	MRVMGIRKNYQHLWREGILLLGILMICSAADNLWVTVYYGVPVWREATTTLFCASDAKAY	60
11	MRVMGIRKNYQHWWREGILLLGILMICSAADKLWVTVYYGVPVWREATTTLFCASDAKAY	60
12	MRVMGIRKNYQHWWREGILLLGILMICSAADKLWVTVYYGVPVWREATTTLFCASDAKAY	60
13	MRVMGIRKNYQHLWREGILLLGILMICSAADNLWVTVYYGVPVWREATTTLFCASDAKAY	60
14	MRVMGIRKNYQHLWREGILLLGILMICSATDNLWVTVYYGVPVWREATTTLFCASDAKAY	60
15	MRVMGIRKNYQHLWREGILLLGILMICSAADDLWVTVYYGVPVWREATTTLFCASDAKAY	60
16	MRVMGIRKNYQHLWRKGILLLGILMICSATNNLWVTVYYGVPVWREATTTLFCASDAKAY	60
17	MRVMGIRKNYQHLWREGILLLGILMICSAADNLWVTVYYGVPVWREATTTLFCASDAKAY	60
18	MRVMGIRKNYQHWWREGILLLGILMICSAADKLWVTVYYGVPVWREATTTLFCASDAKAY	60
19	MRVMGIRKNYQHLWREGILLLGILMICSAADNLWVTVYYGVPVWREATTTLFCASDAKAY	60
20	MRVMGIRKNYQHLWREGILLLGILMICSAADNLWVTVYYGVPVWREATTTLFCASDAKAY	60
21	MRVMGIRKNYQHLWRKGILLLGILMICSATNNLWVTVYYGVPVWREATTTLFCASDAKAY	60
22	MRVMGIRKNYQHLWREGILLLGILMICSAADNLWVTVYYGVPVWREATTTLFCASDAKAY	60
23	MRVMGIRKNYQHLWREGILLLGILMICSAADNLWVTVYYGVPVWREATTTLFCASDAKAY	60
24	MRVMGIRKNYQHLWREGILLLGILMICSAADNLWVTVYYGVPVWREATTTLFCASDAKAY	60
25	MRVMGIRKNYQHLWRKGILLLGILMICSAADKLWVTVYYGVPVWREATTTLFCASDAKAY	60
26	MRVMGIRKNYQHLWREGILLLGILMICSATDNLWVTVYYGVPVWREATTTLFCASDAKAY	60
27	MRVMGIRKNYQHLWREGILLLGILMICSAADNLWVTVYYGVPVWREATTTLFCASDAKAY	60
28	MRVMGIRKNYQHLWREGILLLGILMICSAADNLWVTVYYGVPVWREATTTLFCASDAKAY	60
29	MRVMGIRKNYQHLWREGILLLGILMICSAADNLWVTVYYGVPVWREATTTLFCASDAKAY	60
30	MRVMGIRKNYQHLWREGILLLGILMICSAADNLWVTVYYGVPVWREATTTLFCASDAKAY	60
31	MRVMGIRKNYQHLWREGILLLGILMICSAADNLWVTVYYGVPVWREATTTLFCASDAKAY	60
32	MRVMGIRKNYQHLWREGILLLGILMICSAADNLWVTVYYGVPVWREATTTLFCASDAKAY	60
33	MRVMGIRKNYQHLWREGILLLGILMICSAADDLWVTVYYGVPVWREATTTLFCASDAKAY	60
34	MRVMGIRKNYQHLWREGILLLGILMICSAADNLWVTVYYGVPVWREATTTLFCASDAKAY	60
35	MRVMGIRKNYQHLWRGGILLLGILMICSAADNLWVTVYYGVPVWREATTTLFCASDAKAY	60

	Sequence	
	DTEAHNVWATHACVPTDPNPQEVELKNVTENFNMWENNMVEQMHEDIISLWDQSLKPCVK	
1	DTEAHNVWATHACVPTDPNPQEVELKNVTENFNMWENNMVEQMHEDIISLWDQSLKPCVK	120
2	DTEAHNVWATHACVPTDPNPQEVE--NVTENFNMWENNMVEQMHEDIISLWDQSLKPCVK	118
3	DTEAHNVWATHACVPTDPNPQEVELKNVTENFNMWENNMVEQMHEDIISLWDQSLKPCVK	120
4	DTEAHNVWATHACVPTDPNPQEVELKNVTENFNMWENNMVEQMHEDIISLWDQSLKPCVK	120
5	DTEAHNVWATHACVPTDPNPQEVELKNVTENFNMWENNMVEQMHEDIISLWDQSLKPCVK	120
6	DTEAHNVWATHACVPTDPNPQEVELKNVTENFNMWENNMVEQMHEDIISLWDQSLKPCVK	120
7	DTEAHNVWATHACVPTDPNPQEVELKNVTENFNMWENNMVEQMHEDIISLWDQSLKPCVK	120
8	DTEAHNVWATHACVPTDPNPQEVELKNVTENFNMWENNMVEQMHEDIISLWDQSLKPCVK	120
9	DTEAHNVWATHACVPTDPNPQEVELKNVTENFNMWENNMVEQMHEDIISLWDQSLKPCVK	120
10	DTEAHNVWATHACVPTDPNPQEVELKNVTENFNMWENNMVEQMHEDIISLWDQSLKPCVK	120
11	DTEAHNVWATHACVPTDPNPQEVELKNVTENFNMWENNMVEQMHEDIISLWDQSLKPCVK	120
12	DTEAHNVWATHACVPTDPNPQEVELKNVTENFNMWENNMVEQMHEDIISLWDQSLKPCVK	120
13	DTEAHNVWATHACVPTDPNPQEVELKNVTENFNMWENNMVEQMHEDIISLWDQSLKPCVK	120
14	DTEAHNVWATHACVPTDPNPQEVELKNVTENFNMWENNMVEQMHEDIISLWDQSLKPCVK	120
15	DTEAHNVWATHACVPTDPNPQEVELKNVTENFNMWENNMVEQMHEDIISLWDQSLKPCVK	120
16	DTEAHNVWATHACVPTDPNPQEVELKNVTENFNMWENNMVEQMHEDIISLWDQSLKPCVK	120
17	DTEAHNVWATHACVPTDPNPQEVELKNVTENFNMWENNMVEQMHEDIISLWDQSLKPCVK	120
18	DTEAHNVWATHACVPTDPNPQEVELKNVTENFNMWENNMVEQMHEDIISLWDQSLKPCVK	120
19	DTEAHNVWATHACVPTDPNPQEVELKNVTENFNMWENNMVEQMHEDIISLWDQSLKPCVK	120
20	DTEAHNVWATHACVPTDPNPQEVELKNVTENFNMWENNMVEQMHEDIISLWDQSLKPCVK	120
21	DTEAHNVWATHACVPTDPNPQEVELKNVTENFNMWENNMVEQMHEDIISLWDQSLKPCVK	120
22	DTEAHNVWATHACVPTDPNPQEVELKNVTENFNMWENNMVEQMHEDIISLWDQSLKPCVK	120
23	DTEAHNVWATHACVPTDPNPQEVELKNVTENFNMWENNMVEQMHEDIISLWEQSLKPCVK	120
24	DTEAHNVWATHACVPTDPNPQEVELKNVTENFNMWENNMVEQMHEDIISLWDQSLKPCVK	120
25	DTEAHNVWATHACVPTDPNPQEVELKNVTENFNMWENNMVEQMHEDIISLWDQSLKPCVK	120
26	DTEAHNVWATHACVPTDPNPQEVELKNVTENFNMWENNMVEQMHEDIISLWDQSLKPCVK	120
27	DTEAHNVWATHACVPTDPNPQEVELKNVTENFNMWENNMVEQMHEDIISLWDQSLKPCVK	120
28	DTEAHNVWATHACVPTDPNPQEVELKNVTENFNMWENNMVEQMHEDIISLWDQSLKPCVK	120
29	DTEAHNVWATHACVPTDPNPQEVELKNVTENFNMWENNMVEQMHEDIISLWDQSLKPCVK	120
30	DTEAHNVWATHACVPTDPNPQEVELKNVTENFNMWENNMVEQMHEDIISLWDQSLKPCVK	120
31	DTEAHNVWATHACVPTDPNPQEVELKNVTENFNMGKNNMVEQMHEDIISLWDQSLKPCVK	120
32	DTEAHNVWATHACVPTDPNPQEVELKNVTENFNMWENNMVEQMHEDIISLWDQSLKPCVK	120
33	DTEAHNVWATHACVPTDPNPQEVELKNVTENFNMWENNMVEQMHEDIISLWDQSLKPCVK	120
34	DTEAHNVWATHACVPTDPNPQEVELKNVTENFNMWENNMVEQMHEDIISLWDQSLKPCVK	120
35	DTEAHNVWATHACVPTDPNPQEVELKNVTENFNMGKNNMVEQMHEDIISLWDQSLKPCVK	120

	Sequence	
	LTPLCVTLNCTDNVTTTTNRNGTNCTDSGNVTNTTNRNGTLMGNETLMMEKGEVKNCSFR	
1	LTPLCVTLNCTDLGNVTNTT----------------------NSNGEMMEKGEVKNCSFK	158
2	LTPLCVTLNCTDNVTTTTNRNGTNCTDSGNVTNTTNRNGTLMGNETLMMEKGEVKNCSFR	178
3	LTPLCVTLNCTDNVTTTTNRNGTNCTDSGNVTNTTNRNGTLMGNETLMMEKGEVKNCSFR	180
4	LTPLCVTLNCTDNVTTTTNRNGTNCTDSGNVTNTTNRNGTLMGNETLMMEKGEVKNCSFR	180
5	LTPLCVTLNCTDNVTTTTNRNGTNCTDSGNVTNTTNRNGTLMGNETLMMEKGEVKNCSFR	180
6	LTPLCVTLNCTDNVTTTTNRNGTNCTDSGNVTNTTNRNGTLMGNETLMMEKGEVKNCSFR	180
7	LTPLCVTLNCTDNVTTTTNRNGTNCTDSGNVTNTTNRNGTLMGNETLMMEKGEVKNCSFR	180
8	LTPLCVTLNCTDNVTTTTNRNGTNCTDSGNVTNTTNRNGTLMGNETLMMEKGEVKNCSFR	180
9	LTPLCVTLNCTDNVTTTTNRNGTNCTDSGNVTNTTNRNGTLMGNETLMMEKGEVKNCSFR	180
10	LTPLCVTLNCTDNVTTTTNRNGTNCTDSGNVTNTTNRNGTLMGNETLMMEKGEVKNCSFR	180
11	LTPLCVTLNCTDNVTTTTNRNGTNCTDSGNVTNTTNRNGTLMGNETLMMEKGEVKNCSFR	180
12	LTPLCVTLNCTDNVTNTTNRNGTL-----------------MGNETLMMEKGEVKNCSFR	163
13	LTPLCVTLNCTDNVTTTTNRNGTNCTDSGNVTNTTNRNGTLMGNETLMMEKGEVKNCSFR	180
14	LTPLCVTLNCTDNVTTTTNRNGTNCTDSGNVTNTTNRNGTLMGNETLMMEKGEVKNCSFR	180
15	LTPLCVTLNCTDNVTTTTNRNGTNCTDSGNVTNTTNRNGTLMGNETLMMEKGEVKNCSFR	180
16	LTPLCVTLNCTDNVTTTTNRNGTNCTDSGNVTNTTNRNGTLMGNETLMMEKGEVKNCSFR	180
17	LTPLCVTLNCTDNVTTTTNRNGTNCTDSGNVTNTTNRNGTLMGNETLMMEKGEVKNCSFR	180
18	LTPLCVTLNCTDNVTTTTNRNGTNCTDSGNVTNT-------TNRNGALMEKGEVKNCSFR	173
19	LTPLCVTLNCTDNVTTTTNRNGTNCTDSGNVTNTTNRNGTLMGNETLMMEKGEVKNCSFR	180
20	LTPLCVTLNCTDNVTTTTNRNGTNCTDSGNVTNTTNRNGTLMGNETLMMEKGEVKNCSFR	180
21	LTPLCVTLNCTDNVTTTTNRNGTNCTDSGNVTNTTNRNGTLMGNETLMMEKGEVKNCSFR	180
22	LTPLCVTLNCTDNVTTTTNRNGTNCTDSGNVTNTTNRNGTLMGNETLMMEKGEVKNCSFR	180
23	LTPLCVTLNCTDNVTTTTNRNGTNCTDSGNVTNTTNRNGTLMGNETLMMEKGEVKNCSFR	180
24	LTPLCVTLNCTDNVTTTTNRNGTNCTDSGNVTNTTNRNGTLMGNETLMMEKGEVKNCSFR	180
25	LTPLCVTLNCTDNVTTTTNRNGTNCTDSGNVTNTTNRNGTLMGNETLMMEKGEVKNCSFR	180
26	LTPLCVTLNCTDNVTTTTNRNGTNCTDSGNVTNTTNRNGTLMGNETLMMEKGEVKNCSFR	180
27	LTPLCVTLNCTDNVTTTTNRNGTNCTDSGNVTNTTNRNGTLMGNETLMMEKGEVKNCSFR	180
28	LTPLCVTLNCTDNVTTTTNRNGTNCTDSGNVTNTTNRNGTLMGNETLMMEKGEVKNCSFR	180
29	LTPLCVTLNCTDNVTTTTNRNGTNCTDSGNVTNTTNRNGTLMGNETLMMEKGEVKNCSFR	180
30	LTPLCVTLNCTDNVTTTTNRNGTNCTDSGNVTNTTNRNGTLMGNETLMMEKGEVKNCSFR	180
31	LTPLCVTLNCTDNVTTTTNRNGTNYTDSGNVTNT-------TNRNGALMEKGEVKNCSFR	173
32	LTPLCVTLNCTDNVTTTTNRNGTNCTDSGNVTNTTNRNGTLMGNETLMMEKGEVKNCSFR	180
33	LTPLCVTLNCTDNVTTTTNRNGTNCTDSGNVTNTTNRNGTLMGNETLMMEKGEVKNCSFR	180
34	LTPLCVTLNCTDNVTTTTNRNGTNCTDSGNVTNTTNRNGTLMGNETLMMEKGEVKNCSFR	180
35	LTPLCVTLNCTDLGNVTTTTNRNGTNCTDSGNVTAT-----TNRNETFMEKEEV-NCSFR	174

	Sequence	
	ITTDMQDRTRKEYALFYKLDVVPIND-------TSYRLVSCNTSVITQACPKVSFEPIPI	
1	ITTDIKDRTRKEYALFYKLDVVPIND-------TRYRLVSCNTSVITQACPKVSFEPIPI	211
2	ITTDMQDRTRKEYALFYKLDVVPIND-------TRYRLVSCNTSVITQACPKVSFEPIPI	231
3	ITTDMQDRTRKEYALFYKLDVVPIND-------TSYRLVSCNTSVITQACPKVSFEPIPI	233
4	ITTDMQDRTRKEYALFYKLDVVPIND-------TSYRLVSCNTSVITQACPKVSFEPIPI	233
5	ITTDMQDRTRKEYALFYKLDVVPIND-------TSYRLVSCNTSVITQACPKVSFEPIPI	233
6	ITTDMQDRTRKEYALFYKLDVVPIND-------TSYRLVSCNTSVITQACPKVSFEPIPI	233
7	ITTDMQDRTRKEYALFYKLDVVPIND-------TSYRLVSCNTSVITQACPKVSFEPIPI	233
8	ITTDMQDRTRKEYALFYKLDVVPIND-------TSYRLVSCNTSVITQACPKVSFEPIPI	233
9	ITTDMQDRTRKEYALFYKLDVVPIND-------TSYRLVSCNTSVITQACPKVSFEPIPI	233
10	ITTDMQDRTRKEYALFYKLDVVPIND-------TSYRLVSCNTSVITQACPKVSFEPIPI	233
11	ITTDMQDRTRKEYALFYKLDVVPIND-------TSYRLVSCNTSVITQACPKVSFEPIPI	233
12	ITTDMQDRTRKEYALFYKLDVVPIND-------TSYRLVSCNTSVITQACPKVSFEPIPI	216
13	ITTDMQDRTRKEYALFYKLDVVPIND-------TSYRLVSCNTSVITQACPKVSFEPIPI	233
14	ITTDMQDRTRKEYALFYKLDVVPIND-------TSYRLVSCNTSVITQACPKVSFEPIPI	233
15	ITTDMQDRTRKEYALFYKLDVVPIND-------TSYRLVSCNTSVITQACPKVSFEPIPI	233
16	ITTDMQDRTRKEYALFYKLDVVPIND-------TSYRLVSCNTSVITQACPKVSFEPIPI	233
17	ITTDMQDRTRKEYALFYKLDVVPIND-------TSYRLVSCNTSVITQACPKVSFEPIPI	233
18	ITTDMQDRTRKEYALFYKLDVVPIND-------TRYRLVSCNTSVITQACPKVSFEPIPI	226
19	ITTDMQDRTRKEYALFYKLDVVPIND-------TSYRLVSCNTSVITQACPKVSFEPIPI	233
20	ITTDMQDRTRKEYALFYKLDVVPIND-------TSYRLVSCNTSVITQACPKVSFEPIPI	233
21	ITTDMQDRTRKEYALFYKLDVVPIND-------TSYRLVSCNTSVITQACPKVSFEPIPI	233
22	ITTDMQDRTRKEYALFYKLDVVPIND-------TSYRLVSCNTSVITQACPKVSFEPIPI	233
23	ITTDMQDRTRKEYALFYKLDVVPIND-------TSYRLVSCNTSVIAQACPKVSFEPIPI	233
24	ITTDMQDRTRKEYALFYKLDVVPIND-------TSYRLVSCNTSVITQACPKVSFEPIPI	233
25	ITTDMQDRTRKEYALFYKLDVVPIND-------TSYRLVSCNTSVITQACPKVSFEPIPI	233
26	ITTDMQDRTRKEYALFYKLDVVPIND-------TSYRLVSCNTSVITQACPKVSFEPIPI	233
27	ITTDMQDRTRKEYALFYKLDVVPIND-------TSYRLVSCNTSVITQACPKVSFEPIPI	233
28	ITTDMQDRTRKEYALFYKLDVVPIND-------TSYRLVSCNTSVITQACPKVSFEPIPI	233
29	ITTDMQDRTRKEYALFYKLDVVPIND-------TSYRLVSCNTSVITQACPKVSFEPIPI	233
30	ITTDMQDRTRKEYALFYKLDVVPIND-------TSYRLVSCNTSVITQACPKVSFEPIPI	233
31	ITTDMQDRTRKEYALFYKLDVVPIND-------TRYRLVSCNTSVITQACPKVSFEPIPI	226
32	ITTDMQDRTRKEYALFYKLDVVPIND-------TSYRLVSCNTSVITQACPKVSFEPIPI	233
33	ITTDMQDRTRKEYALFYKLDVVPIND-------TSYRLVSCNTSVITQACPKVSFEPIPI	233
34	ITTDMQDRTRKEYALFYKLDVVPIND-------TSYRLVSCNTSVITQACPKVSFEPIPI	233
35	ITTDMQDRTRKEYALFYKLDVVPIND-------TRYRLVSCNTSVITQACPKVSFEPIPI	227

	Sequence	
	HYCAPAGFAILKCNDKQFNGTGPCTNVSTVQCTHGIRPVVSTQLLLNGSLAEEEVVIRSV	
1	HYCAPAGFAILKCNDKQFIGTGPCTNVSTVQCTHGIRPVVSTQLLLNGSLAEEEVVIRSV	271
2	HYCAPAGFAILKCNDKQFNGTGPCTNVSTVQCTHGIRPVVSTQLLLNGSLAEEEVVIRSV	291
3	HYCAPAGFAILKCNDKQFNGTGPCTNVSTVQCTHGIRPVVSTQLLLNGSLAEEEVVIRSV	293
4	HYCAPAGFAILKCNDKQFNGTGPCTNVSTVQCTHGIRPVVSTQLLLNGSLAEEEVVIRSV	293
5	HYCAPAGFAILKCNDKQFNGTGPCTNVSTVQCTHGIRPVVSTQLLLNGSLAEEEVVIRSV	293
6	HYCAPAGFAILKCNDKQFNGTGPCTNVSTVQCTHGIRPVVSTQLLLNGSLAEEEVVIRSV	293
7	HYCAPAGFAILKCNDKQFNGTGPCTNVSTVQCTHGIRPVVSTQLLLNGSLAEEEVVIRSV	293
8	HYCAPAGFAILKCNDKQFNGTGPCTNVSTVQCTHGIRPVVSTQLLLNGSLAEEEVVIRSV	293
9	HYCAPAGFAILKCNDKQFNGTGPCTNVSTVQCTHGIRPVVSTQLLLNGSLAEEEVVIRSV	293
10	HYCAPAGFAILKCNDKQFNGTGPCTNVSTVQCTHGIRPVVSTQLLLNGSLAEEEVVIRSV	293
11	HYCAPAGFAILKCNDKQFNGTGPCTNVSTVQCTHGIRPVVATQLLLNGSLAEEEVVIRSV	293
12	HYCAPAGFAILKCNDKQFNGTGPCTNVSTVQCTHGIRPVVSTQLLLNGSLAEEEVIIRSV	276
13	HYCAPAGFAILKCNDKQFNGTGPCTNVSTVQCTHGIRPVVSTQLLLNGSLAEEEVVIRSV	293
14	HYCAPAGFAILKCNDKQFNGTGPCTNVSTVQCTHGIRPVVSTQLLLNGSLAEEEVVIRSV	293
15	HYCAPAGFAILKCNDKQFNGTGPCTNVSTVQCTHGIRPVVSTQLLLNGSLAEEEVVIRSV	293
16	HYCAPAGFAILKCNDKQFNGTGPCTNVSTVQCTHGIRPVVSTQLLLNGSLAEEEVVIRSV	293
17	HYCAPAGFAILKCNDKQFNGTGPCTNVSTVQCTHGIRPVVSTQLLLNGSLAEEEVVIRSV	293
18	HYCAPAGFAILKCNDKQFNGTGPCTNVSTVQCTHGIRPVVSTQLLLNGSLAEEEVVIRSV	286
19	HYCAPAGFAILKCNDKQFNGTGPCTNVSTVQCTHGIRPVVSTQLLLNGSLAEEEVVIRSV	293
20	HYCAPAGFAILKCNDKQFNGTGPCTNVSTVQCTHGIRPVVSTQLLLNGSLAEEEVVIRSV	293
21	HYCAPAGFAILKCNDKQFNGTGPCTNVSTVQCTHGIRPVVSTQLLLNGSLAEEEVVIRSV	293
22	HYCAPAGFAILKCNDKQFNGTGPCTNVSTVQCTHGIRPVVSTQLLLNGSLAEEEVVIRSV	293
23	HYCAPAGFAILKCNDKQFNGTGPCTNVSTVQCTHGIRPVVSTQLLLNGSLAEEEVVIRSV	293
24	HYCAPAGFAILKCNDKQFNGTGPCTNVSTVQCTHGIRPVVSTQLLLNGSLAEEEVVIRSV	293
25	HYCAPAGFAILKCNDKQFNGTGPCTNVSTVQCTHGIRPVVSTQLLLNGSLAEEEVVIRSV	293
26	HYCAPAGFAILKCNDKQFNGTGPCTNVSTVQCTHGIRPVVSTQLLLNGSLAEEEVVIRSV	293
27	HYCAPAGFAILKCNDKQFNGTGPCTNVSTVQCTHGIRPVVSTQLLLNGSLAEEEVVIRSV	293
28	HYCAPAGFAILKCNDKQFNGTGPCTNVSTVQCTHGIRPVVSTQLLLNGSLAEEEVVIRSV	293
29	HYCAPAGFAILKCNDKQFNGTGPCTNVSTVQCTHGIRPVVSTQLLLNGSLAEEEVVIRSV	293
30	HYCAPAGFAILKCNDKQFNGTGPCTNVSTVQCTHGIRPVVSTQLLLNGSLAEEEVVIRSV	293
31	HYCAPAGFAILKCNDKQFNGTGPCTNVSTVQCTHGIRPVVSTQLLLNGSLAEEEVVIRSV	286
32	HYCAPAGFAILKCNDKQFNGTGPCTNVSTVQCTHGIRPVVSTQLLLNGSLAEEEVVIRSV	293
33	HYCAPAGFAILKCNDKQFNGTGPCTNVSTVQCTHGIRPVVSTQLLLNGSLAEEEVVIRSV	293
34	HYCAPAGFAILKCNDKQFNGTGPCTNVSTVQCTHGIRPVVSTQLLLNGSLAEEEVVIRSV	293
35	HYCAPAGFAILKCNDKQFNGTGPCTNVSTVQCTHGIRPVVSTQLLLNGSLAEEEVVIRSV	287

	NFSDNAKTIIVQLNKSVEINCTRPNNNTRKSIHMGPGKAFYARGGIIGDIRKAHCNISGT	
1	NFSDNAKTIIVQLNKSVEITCTRPNNNTRKSIPMGPGKAFYARGDITGDIRKAYCEINGT	331
2	NFSDNAKTIIVQLNKSVEINCTRPNNNTRKSIHMGPGKAFYARGAIIGDIRKAHCNISGT	351
3	NFSDNAKTIIVQLNKSVEINCTRPNNNTRKSIHMGPGKAFYARGGIIGDIRKAHCNISGT	353
4	NFSDNAKTIIVQLNKSVEINCTRPNNNTRKSIHMGPGKAFYARGGIIGDIRKAHCNISGT	353
5	NFSDNAKTIIVQLNKSVEINCTRPNNNTRKSIHMGPGKAFYARGGIIGDIRKAHCNISGT	353
6	NFSDNAKTIIVQLNKSVEINCTRPNNNTRKSIHMGPGKAFYARGGIIGDIRKAHCNISGT	353
7	NFSDNAKTIIVQLNKSVEINCTRPNNNTRKSIHMGPGKAFYARGGIIGDIRKAHCNISGT	353
8	NFSDNAKTIIVQLNKSVEINCTRPNNNTRKSIHMGPGKAFYARGGIIGDIRKAHCNISGT	353
9	NFSDNAKTIIVQLNKSVEINCTRPNNNTRKSIHMGPGKAFYARGGIIGDIRKAHCNISGT	353
10	NFSDNAKTIIVQLNKSVEINCTRPNNNTRKSIHMGPGKAFYARGGIIGDIRKAHCNISGT	353
11	NFSDNAKTIIVQLNKSVEINCTRPNNNTRKSIHMGPGKAFYARGAIIGDIRKAHCNISRA	353
12	NFSDNAKTIIVQLNKSVEINCTRPNNNTRKSIHMGPGKAFYARGGIIGDIRKAHCNISGT	336
13	NFSDNAKTIIVQLNKSVEINCTRPNNNTRKSIHMGPGKAFYARGAIIGDIRKAHCNISGT	353
14	NFSDNAKTIIVQLNKSVEINCTRPNNNTRKSIHMGPGKAFYARGGIIGDIRKAHCNISGT	353
15	NFSDNAKTIIVQLNKSVEINCTRPNNNTRKSIHMGPGKAFYARGAIIGDIRKAHCNISGT	353
16	NFSDNAKTIIVQLNKSVEINCTRPNNNTRKSIHMGPGKAFYARGAIIGDIRKAHCNISGT	353
17	NFSDNAKTIIVQLNKSVEINCTRPNNNTRKSIHMGPGKAFYARGGIIGDIRKAHCNISGT	353
18	NFSDNAKTIIVQLNKSVEINCTRPNNNTRKSIHMGPGKAFYARGGIIGDIRKAHCNISGT	346
19	NFSDNAKTIIVQLNKSVEINCTRPNNNTRKSIHMGPGKAFYARGGIIGDIRKAHCNISGT	353
20	NFSDNAKTIIVQLNKSVEINCTRPNNNTRKSIHMGPGKAFYARGGIIGDIRKAHCNISGT	353
21	NFSDNAKTIIVQLNKSVEINCTRPNNNTRKSIHMGPGKAFYARGAIIGDIRKAHCNISGT	353
22	NFSDNAKTIIVQLNKSVEINCTRPNNNTRKSIHMGPGKAFYARGAIIGDIRKAHCNISGT	353
23	NFSDNAKTIIVQLNKSVEINCTRPNNNTRKSIHMGPGKAFYARGGIIGDIRKAHCNISGT	353
24	NFSDNAKTIIVQLNKSVEINCTRPNNNTRKSIHMGPGKAFYARGAIIGDIRKAHCNISGT	353
25	NFSDNAKTIIVQLNKSVEINCTRPNNNTRKSIHMGPGKAFYARGGIIGDIRKAHCNISGT	353
26	NFSDNAKTIIVQLNKSVEINCTRPNNNTRKSIHMGPGKAFYARGGIIGDIRKAHCNISGT	353
27	NFSDNAKTIIVQLNKSVEINCTRPNNNTRKSIHMGPGKAFYARGGIIGDIRKAHCNISGT	353
28	NFSDNAKTIIVQLNKSVEINCTRPNNNTRKSIHMGPGKAFYARGGIIGDIRKAHCNISGT	353
29	NFSDNAKTIIVQLNKSVEINCTRPNNNTRKSIHMGPGKAFYARGGIIGDIRKAHCNISGT	353
30	NFSDNAKTIIVQLNKSVEINCTRPNNNTRKSIHMGPGKAFYARGAIIGDIRKAHCNISGT	353
31	NFSDNAKTIIVQLNKSVEINCTRPNNNTRKSIHMGPGKAFYARGGIIGDIRKAHCNISGT	346
32	NFSDNAKTIIVQLNKSVEINCTRPNNNTRKSIHMGPGKAFYARGGIIGDIRKAHCNISGT	353
33	NFSDNAKTIIVQLNKSVEINCTRPNNNTRKSIHMGPGKAFYARGGIIGDIRKAHCNISGT	353
34	NFSDNAKTIIVQLNKSVEINCTRPNNNTRKSIHMGPGKAFYARGAIIGDIRKAHCNISGT	353
35	NFSDNAKTIIVQLNKSVEINCTRPNNNTRKSIHMGPGKAFYARGGIIGDIRKAHCNISGT	347

	EWHNTLKLVVEKLREQY-NKTIVFNHSSGGDPEIVMYSFNCGGEFFYCNSTKLFNSTWPG	
1	EWHSTLKLVVEKLREQY-NKTIVFNRSSGGDPEIVMYSFNCGGEFFYCNSTKLFNSTWPW	390
2	EWHNTLKLVVEKLREQY-NKTIVFNHSSGGDPEIVMYSFNCGGEFFYCNSTKLFNSTWPG	410
3	EWHNTLKLVVEKLREQY-NKTIVFNHSSGGDPEIVMYSFNCGGEFFYCNSTKLFNSTWPG	412
4	EWHNTLKLVVAKLREQY-NKTIVFNHSSGGDPEIVMYSFNCGGEFFYCNSTKLFNSTWPG	412
5	EWHNTLKLVVEKLREQY-NKTIVFNHSSGGDPEIVMYSFNCGGEFFYCNSTKLFNSTWPG	412
6	EWHNTLKLVVEKLREQY-NKTIVFNHSSGGDPEIVMYSFNCGGEFFYCNSTKLFNSTWPG	412
7	EWHNTLKLVVEKLREQY-NKTIVFNHSSGGDPEIVMYSFNCGGEFFYCNSTKLFNSTWPG	412
8	EWHNTLKLVVKKLREQY-NKTIVFNHSSGGDPEIVMYSFNCGGEFFYCNSTKLFNSTWPG	412
9	EWHNTLKLVVEKLREQY-NKTIVFNHSSGGDPEIVMYSFNCGGEFFYCNSTKLFNSTWPG	412
10	EWHNTLKLVVEKLREQY-NKTIVFNHSSGGDPEIVMYSFNCGGEFFYCNSTKLFNSTWPG	412
11	EWHSTLKLVAEKLREQY-NKTIVFNHSSGGDPEIVMYSFNCGGEFFYCNSTKLFNSTWSW	412
12	EWHNTLKLVVEKLREQY-NKTIVFNHSSGGDPEIVMYSFNCGGEFFYCNSTKLFNSTWPG	395
13	EWHNTLKLVVEKLREQY-NKTIVFNHSSGGDPEIVMYSFNCGGEFFYCNSTKLFNSTWPG	412
14	EWHNTLKLVVEKLREQY-NKTIVFNHSSGGDPEIVMYSFNCGGEFFYCNSTKLFNSTWPW	412
15	EWHNTLKLVVEKLREQY-NKTIVFNHSSGGDPEIVMYSFNCGGEFFYCNSTKLFNSTWPG	412
16	EWHNTLKLVVEKLREQY-NKTIVFNHSSGGDPEIVMYSFNCGGEFFYCNSTKLFNSTWPR	412
17	EWHNTLKLVVEKLREQY-NKTIVFNHSSGGDPEIVMYSFNCGGEFFYCNSTKLFNSTWPG	412
18	EWHSTLKLVVKKLREQY-NKTIVFNHSSGGDPEIVMYSFNCGGEFFYCNSTKLFNSTWPW	405
19	EWHNTLKLVVEKLREQY-NKTIVFNHSSGGDPEIVMYSFNCGGEFFYCNSTKLFNSTWPG	412
20	EWHNTLKLVVEKLREQY-NKTIVFNHSSGGDPEIVMYSFNCGGEFFYCNSTKLFNSTWPG	412
21	EWHNTLKLVVEKLREQY-NKTIVFNHSSGGDPEIVMYSFNCGGEFFYCNSTKLFNSTWPR	412
22	EWHNTLKLVVEKLREQY-NKTIVFNHSSGGDPEIVMYSFNCGGEFFYCNSTKLFNSTWPG	412
23	EWHNTLKLVVEKLREQY-NKTIVFNHSSGGDPEIVMYSFNCGGEFFYCNSTKLFNSTWPG	412
24	EWHNTLKLVVEKLREQY-NKTIVFNHSSGGDPEIVMYSFNCGGEFFYCNSTKLFNSTWPG	412
25	EWHNTLKLVVEKLREQY-NKTIVFNHSSGGDPEIVMYSFNCGGEFFYCNSTKLFNSTWPR	412
26	EWHNTLKLVVEKLREQY-NKTIVFNHSSGGDPEIVMYSFNCGGEFFYCNSTKLFNSTWPG	412
27	EWHNTLKLVVEKLREQY-NKTIVFNHSSGGDPEIVMYSFNCGGEFFYCNSTKLFNSTWPG	412
28	EWHSTLKLVVKKLREQY-NKTIVFNHSSGGDPEIVMYSFNCGGEFFYCNSTKLFNSTWPG	412
29	EWHNTLKLVVEKLREQY-NKTIVFNHSSGGDPEIVMYSFNCGGEFFYCNSTKLFNSTWPG	412
30	EWHNTLKLVVEKLREQY-NKTIVFNHSSGGDPEIVMYSFNCGGEFFYCNSTKLFNSTWPG	412
31	EWHNTLKLVVEKLREQY-NKTIVFNHSSGGDPEIVMYSFNCGGEFFYCNSTKLFNSTWPG	405
32	EWHNTLKLVVEKLREQY-NKTIVFNHSSGGDPEIVMYSFNCGGEFFYCNSTKLFNSTWPG	412
33	EWHNTLKLVVEKLREQY-NKTIVFNHSSGGDPEIVMYSFNCGGEFFYCNSTKLFNSTWPG	412
34	EWHNTLKLVVEKLREQY-NKTIVFNHSSGGDPEIVMYSFNCGGEFFYCNSTKLFNSTWPG	412
35	EWHNTLKLVVEKLREQY-NKTIVFNHSSGGDPEIVMYSFNCGGEFFYCNSTKLFNSTWPG	406

	Sequence	
	ND--TEGSHNTNGTLTLPCRIKQIINMWQGVGKAMYAPPIEGIIKCSSNITGLLLTRDGG	
1	ND--TKGSHDTNGTLILPCKIKQIINMWQGVGKAMYAPPIEGKIRCSSNITGLLLTRDGG	448
2	ND--TEGSHNTNGTLTLPCRIKQIINMWQEVGKAMYAPPIEGIIKCSSNITGLLLTRDGG	468
3	ND--TEGSHDTNGTLTLPCRIKQIINMWQEVGKAMYAPPIEGIIKCSSNITGLLLTRDGG	470
4	ND--TEGSHNTNGTLTLPCRIKQIINMWQGVGKAMYAPPIEGIIKCSSNITGLLLTRDGG	470
5	ND--TEGSHNTNGTLTLPCRIKQIINMWQGVGKAMYAPPIEGIIKCSSNITGLLLTRDGG	470
6	ND--TEGSHNTNGTLTLPCRIKQIINMWQGVGKAMYAPPIEGIIKCSSNITGLLLTRDGG	470
7	ND--TEGSHNTNGTLTLPCRIKQIINMWQGVGKAMYAPPIEGIIKCSSNITGLLLTRDGG	470
8	ND--TEGSHNTNGTLTLPCRIKQIINMWQEVGKAMYAPPIEGIIKCSSNITGLLLTRDGG	470
9	ND--TEGSHNTNGTLTLPCRIKQIINMWQGVGKAMYAPPIEGIIKCSSNITGLLLTRDGG	470
10	ND--TEGSHNTNGTLTLPCRIKQIINMWQGVGKAMYAPPIEGIIKCSSNITGLLLTRDGG	470
11	ND--TQGSHDTNGTLTLPCRIKQIINMWQEVGKAMYAPPIEGIIKCSSNITGLLLTRDGG	470
12	ND--TEGSHNTNGTLTLPCRIKQIINMWQGVGKAMYAPPIEGIIKCSSNITGLLLTRDGG	453
13	ND--TEGSHNTNGTLTLPCRIKQIINMWQEVGKAMYAPPIEGIIKCSSNITGLLLTRDGG	470
14	NND-TEGSHDTNGTLTLPCRIKQIINMWQEVGKAMYAPPIEGIIKCSSNITGLLLTRDGG	471
15	ND--TEGSHNTNGTLTLPCRIKQIINMWQGVGKAMYAPPIEGIIKCSSNITGLLLTRDGG	470
16	NA--TRGSPDTNGTLTLPCRIKQIINMWQEVGKAMYAPPIEGIIKCSSNITGLLLTRDGG	470
17	ND--TEGSHNTNGALTLPCRIKQIINMWQGVGKAMYAPPIEGIIKCSSNITGLLLTRDGG	470
18	NDTDTEGSHDTNGTLTLPCRIKQIINMWQEVGKAMYAPPIEGIIKCSSNITGLLLTRDGG	465
19	ND--TEGSHNTNGTLTLPCRIKQIINMWQGVGKAMYAPPIEGIIKCSSNITGLLLTRDGG	470
20	ND--TEGSHNTNGTLTLPCRIKQIINMWQGVGKAMYAPPIEGIIKCSSNITGLLLTRDGG	470
21	NA--TRGSPDTNGTLTLPCRIKQIINMWQEVGKAMYAPPIEGIIKCSSNITGLLLTRDGG	470
22	NA--TEGSHDTNGTLTLPCRIKQIINMWQEVGKAMYAPPIEGIIKCSSNITGLLLTRDGG	470
23	ND--TEGSHNTNGTLTLPCRIKQIINMWQGVGKAMYAPPIEGIIKCSSNITGLLLTRDGG	470
24	ND--TEGSHNTNGTLTLPCRIKQIINMWQEVGKAMYAPPIEGIIKCSSNITGLLLTRDGG	470
25	ND--TEGSHNTNGTLTLPCRIKQIINMWQEVGKAMYAPPIEGIIKCSSNITGLLLTRDGG	470
26	ND--TEGSHNTNGTLTLPCRIKQIINMWQGVGKAMYAPPIEGIIKCSSNITGLLLTRDGG	470
27	ND--TEGSHNTNGTLTLPCRIKQIINMWQGVGKAMYAPPIEGIIKCSSNITGLLLTRDGG	470
28	ND--TEGSHNTNGTLTLPCRIKQIINMWQGVGKAMYAPPIEGIIKCSSNITGLLLTRDGG	470
29	ND--TEGSHNTNGTLTLPCRIKQIINMWQGVGKAMYAPPIEGIIKCSSNITGLLLTRDGG	470
30	ND--TEGSHNTNGTLTLPCRIKQIINMWQEVGKAMYAPPIEGIIKCSSNITGLLLTRDGG	470
31	ND--TEGSHNTNGTLTLPCRIKQIINMWQGVGKAMYAPPIEGIIKCSSNITGLLLTRDGG	463
32	ND--TEGSHNTNGTLTLPCRIKQIINMWQGVGKAMYAPPIEGIIKCSSNITGLLLTRDGG	470
33	ND--TEGSHNTNGTLTLPCRIKQIINMWQGVGKAMYAPPIEGIIKCSSNITGLLLTRDGG	470
34	ND--TEGSHNTNGTLTLPCRIKQIINMWQEVGKAMYAPPIEGIIKCSSNITGLLLTRDGG	470
35	ND--TEGSHNTNGTLTLPCRIKQIINMWQEVGKAMYAPPIEGIIKC---------TRDGG	455

	-YESNKTDEIFRPGGGDMRDNWRSELYKYKVVKIEPLGVAPTKAKRRVVQREKRAFG-LG	
1	-YESNETDEIFRPGGGDMRDNWRSELYKYKVVKIEPLGVAPTKAKRRVVQREKRAFG-LG	506
2	-YESNKTDEIFRPGGGDMRDNWRSELYKYKVVKIEPLGVAPTKAKRRVVQREKRAFG-LG	526
3	-YESNKTDEIFRPGGGDMRDNWRSELYKYKVVKIEPLGVAPTKAKRRVVQREKRAFG-LG	528
4	-YESNKTDEIFRPGGGDMRDNWRSELYKYKVVKIEPLGVAPTKAKRRVVQREKRAFG-LG	528
5	-YESNKTDEIFRPGGGDMRDNWRSELYKYKVVKIEPLGVAPTKAKRRVVQREKRAFG-LG	528
6	-YESNKTDEIFRPGGGDMRDNWRSELYKYKVVKIEPLGVAPTKAKRRVVQREKRAFG-LG	528
7	-YESNKTDEIFRPGGGDMRDNWRSELYKYKVVKIEPLGVAPTKAKRRVVQREKRAFG-LG	528
8	-YESNKTDEIFRPGGGDMRDNWRSELYKYKVVKIEPLGVAPTKAKRRVVQREKRAFG-LG	528
9	-YESNKTDEIFRPGGGDMRDNWRSELYKYKVVKIEPLGVAPTKAKRRVVQREKRAFG-LG	528
10	-YESNKTDEIFRPGGGDMRDNWRSELYKYKVVKIEPLGIAPTKAKRRVVQREKRAFG-LG	528
11	-YESNKTDEIFRPGGGDMRDNWRSELYKYKVVKIEPLGVAPTKAKRRVVQREKRAFG-LG	528
12	-YESNKTDEIFRPGGGDMRDNWRSELYKYKVVKIEPLGVAPTKAKRRVVQREKRAFG-LG	511
13	-YESNKTDEIFRPGGGDMRDNWRSELYKYKVVKIEPLGVAPTKAKRRVVQREKRAFG-LG	528
14	-YESNKTDEIFRPGGGDMRDNWRSELYKYKVVKIEPLGVAPTKAKRRVVQREKRAFG-LG	529
15	-YESNKTDEIFRPGGGDMRDNWRSELYKYKVVKIEPLGVAPTKAKRRVVQREKRAFG-LG	528
16	-YESNKTDEIFRPGGGDMRDNWRSELYKYKVVKIEPLGVAPTKAKRRVVQREKRAFG-LG	528
17	-YESNKTDEIFRPGGGDMRDNWRSELYKYKVVKIEPLGVAPTKAKRRVVQREKRAFG-LG	528
18	-YESNKTDEIFRPGGGDMRDNWRSELYKYKVVKIEPLGVAPTKAKRRVVQREKRAFG-LG	523
19	-YESNKTDEIFRPGGGDMRDNWRSELYKYKVVKIEPLGVAPTKAKRRVVQREKRAFG-LG	528
20	-YESNKTDEIFRPGGGDMRDNWRSELYKYKVVKIEPLGVAPTKAKRRVVQREKRAFG-LG	528
21	-YESNKTDEIFRPGGGDMRDNWRSELYKYKVVKIEPLGVAPTKAKRRVVQREKRAFG-LG	528
22	-YESNKTDEIFRPGGGDMRDNWRSELYKYKVVKIEPLGVAPTKAKRRVVQREKRAFG-LG	528
23	-YESNKTDEIFRPGGGDMRDNWRSELYKYKVVKIEPLGVAPTKAKRRVVQREKRAFG-LG	528
24	-YESNKTDEIFRPGGGDMRDNWRSELYKYKVVKIEPLGVAPTKAKRRVVQREKRAFG-LG	528
25	-YESNKTDEIFRPGGGDMRDNWRSELYKYKVVKIEPLGVAPTKAKRRVVQREKRAFG-LG	528
26	-YESNKTDEIFRPGGGDMRDNWRSELYKYKVVKIEPLGVAPTKAKRRVVQREKRAFG-LG	528
27	-YESNKTDEIFRPGGGDMRDNWRSELYKYKVVKIEPLGVAPTKAKRRVVQREKRAFG-LG	528
28	-YESNKTDEIFRPGGGDMRDNWRSELYKYKVVKIEPLGVAPTKAKRRVVQREKRAFG-LG	528
29	-YESNKTDEIFRPGGGDMRDNWRSELYKYKVVKIEPLGVAPTKAKRRVVQREKRAFG-LG	528
30	-YESNKTDEIFRPGGGDMRDNWRSELYKYKVVKIEPLGVAPTKAKRRVVQREKRAFG-LG	528
31	-YESNKTDEIFRPGGGDMRDNWRSELYKYKVVKIEPLGVAPTKAKRRVVQREKRAFG-LG	521
32	-YESNKTDEIFRPGGGDMRDNWRSELYKYKVVKIEPLGVAPTKAKRRVVQREKRAFG-LG	528
33	-YESNKTDEIFRPGGGDMRDNWRSELYKYKVVKIEPLGVAPTKAKRRVVQREKRAFG-LG	528
34	-YESNKTDEIFRPGGGDMRDNWRSELYKYKVVKIEPLGVAPTKAKRRVVQREKRAFG-LG	528
35	-YESNKTDEIFRPGGGDMRDNWRSELYKYKVVKIEPLGVAPTKAKRRVVQREKRAFG-LG	513

	**AVFLGFLGAAGSTMGAASITLTVQARQLLSGIVQQQNNLLRAIEAQQHLLQLTVWGIKQL**	
1	AVFLGFLGAAGSTMGAASITLTVQARQLLSGIVQQQNNLLRAIEAQQHLLQLTVWGIKQL	566
2	AVFLGFLGAAGSTMGAASITLTVQARQLLSGIVQQQNNLLRAIEAQQHLLQLTVWGIKQL	586
3	AVFLGFLGAAGSTMGAASITLTVQARQLLSGIVQQQNNLLRAIEAQQHLLQLTVWGIKQL	588
4	AVFLGFLGAAGSTMGAASITLTVQARQLLSGIVQQQNNLLRAIEAQQHLLQLTVWGIKQL	588
5	AVFLGFLGAAGSTMGAASITLTVQARQLLSGIVQQQNNLLRAIEAQQHLLQLTVWGIKQL	588
6	AVFLGFLGAAGSTMGAASITLTVQARQLLSGIVQQQNNLLRAIEAQQHLLQLTVWGIKQL	588
7	AVFLGFLGAAGSTMGAASITLTVQARQLLSGIVQQQNNLLRAIEAQQHLLQLTVWGIKQL	588
8	AVFLGFLGAAGSTMGAASITLTVQARQLLSGIVQQQNNLLRAIEAQQHLLQLTVWGIKQL	588
9	AVFLGFLGAAGSTMGAASITLTVQARQLLSGIVQQQNNLLRAIEAQQHLLQLTVWGIKQL	588
10	AVFLGFLGAAGSTMGAASITLTVQARQLLSGIVQQQNNLLRAIEAQQHLLQLTVWGIKQL	588
11	AVFLGFLGAAGSTMGAASITLTVQARQLLSGIVQQQNNLLRAIEAQQHLLQLTVWGIKQL	588
12	AVFLGFLGAAGSTMGAASITLTVQARQLLSGIVQQQNNLLRAIEAQQHLLQLTVWGIKQL	571
13	AVFLGFLGAAGSTMGAASITLTVQARQLLSGIVQQQNNLLRAIEAQQHLLQLTVWGIKQL	588
14	AVFLGFLGAAGSTMGAASITLTVQARQLLSGIVQQQNNLLRAIEAQQHLLQLTVWGIKQL	589
15	AVFLGFLGAAGSTMGAASITLTVQARQLLSGIVQQQNNLLRAIEAQQHLLQLTVWGIKQL	588
16	AVFLGFLGAAGSTMGAASITLTVQARQLLSGIVQQQNNLLRAIEAQQHLLQLTVWGIKQL	588
17	AVFLGFLGAAGSTMGAASITLTVQARQLLSGIVQQQNNLLRAIEAQQHLLQLTVWGIKQL	588
18	AVFLGFLGAAGSTMGAASITLTVQARQLLSGIVQQQNNLLRAIEAQQHLLQLTVWGIKQL	583
19	AVFLGFLGAAGSTMGAASITLTVQARQLLSGIVQQQNNLLRAIEAQQHLLQLTVWGIKQL	588
20	AVFLGFLGAAGSTMGAASITLTVQARQLLSGIVQQQNNLLRAIEAQQHLLQLTVWGIKQL	588
21	AVFLGFLGAAGSTMGAASITLTVQARQLLSGIVQQQNNLLRAIEAQQHLLQLTVWGIKQL	588
22	AVFLGFLGAAGSTMGAASITLTVQARQLLSGIVQQQNNLLRAIEAQQHLLQLTVWGIKQL	588
23	AVFLGFLGAAGSTMGAASITLTVQARQLLSGIVQQQNNLLRAIEAQQHLLQLTVWGIKQL	588
24	AVFLGFLGAAGSTMGAASITLTVQARQLLSGIVQQQNNLLRAIEAQQHLLQLTVWGIKQL	588
25	AVFLGFLGAAGSTMGAASITLTVQARQLLSGIVQQQNNLLRAIEAQQHLLQLTVWGIKQL	588
26	AVFLGFLGAAGSTMGAASITLTVQARQLLSGIVQQQNNLLRAIEAQQHLLQLTVWGIKQL	588
27	AVFLGFLGAAGSTMGAASITLTVQARQLLSGIVQQQNNLLRAIEAQQHLLQLTVWGIKQL	588
28	AVFLGFLGAAGSTMGAASITLTVQARQLLSGIVQQQNNLLRAIEAQQHLLQLTVWGIKQL	588
29	AVFLGFLGAAGSTMGAASITLTVQARQLLSGIVQQQNNLLRAIEAQQHLLQLTVWGIKQL	588
30	AVFLGFLGAAGSTMGAASITLTVQARQLLSGIVQQQNNLLRAIEAQQHLLQLTVWGIKQL	588
31	AVFLGFLGAAGSTMGAASITLTVQARQLLSGIVQQQNNLLRAIEAQQHLLQLTVWGIKQL	581
32	AVFLGFLGAAGSTMGAASITLTVQARQLLSGIVQQQNNLLRAIEAQQHLLQLTVWGIKQL	588
33	AVFLGFLGAAGSTMGAASITLTVQARQLLSGIVQQQNNLLRAIEAQQHLLQLTVWGIKQL	588
34	AVFLGFLGAAGSTMGAASITLTVQARQLLSGIVQQQNNLLRAIEAQQHLLQLTVWGIKQL	588
35	AVFLGFLGAAGSTMGAASITLTVQARQLLSGIVQQQNNLLRAIEAQQHLLQLTVWGIKQL	573

	**QARVLAVERYLKDQQLLGIWGCSGKLICTTTVPWNTSWSNKSLEQIWDNMTWMEWEREIN**	
1	QARVLAVERYLKDQQLLGIWGCSGKLICTTTVPWNTSWSNKSLEQIWDNMTWMEWEREID	626
2	QARVLAVERYLKDQQLLGIWGCSGKLICTTTVPWNTSWSNKSLEQIWDNMTWMEWEREIN	646
3	QARVLAVERYLKDQQLLGIWGCSGKLICTTTVPWNTSWSNKSLEQIWDNMTWMEWEREIN	648
4	QARVLAVERYLKDQQLLGIWGCSGKLICTTTVPWNTSWSNKSLEQIWDNMTWMEWEREIN	648
5	QARVLAVERYLKDQQLLGIWGCSGKLICTTTVPWNTSWSNKSLEQIWDNMTWMEWEREIN	648
6	QARVLAVERYLKDQQLLGIWGCSGKLICTTTVPWNTSWSNKSLEQIWDNMTWMEWEREIN	648
7	QARVLAVERYLKDQQLLGIWGCSGKLICTTTVPWNTSWSNKSLEQIWDNMTWMEWEREIN	648
8	QARVLAVERYLKDQQLLGIWGCSGKLICTTTVPWNTSWSNKSLEQIWDNMTWMEWEREIN	648
9	QARVLAVERYLKDQQLLGIWGCSGKLICTTTVPWNTSWSNKSLEQIWDNMTWMEWEREIN	648
10	QARVLAVERYLKDQQLLGIWGCSGKLICTTTVPWNTSWSNKSLEQIWDNMTWMEWEREIN	648
11	QARVLAVERYLKDQQLLGIWGCSGKLICTTTVPWNTSWSNKSLEQIWDNMTWMEWEREIN	648
12	QARVLAVERYLKDQQLLGIWGCSGKLICTTTVPWNTSWSNKSLEQIWDNMTWMEWEREIN	631
13	QARVLAVERYLKDQQLLGIWGCSGKLICTTTVPWNTSWSNKSLEQIWDNMTWMEWEREIN	648
14	QARVLAVERYLKDQQLLGIWGCSGKLICTTTVPWNTSWSNKSLEQIWDKMTWMEWEREID	649
15	QARVLAVERYLKDQQLLGIWGCSGKLICTTTVPWNTSWSNKSLEQIWDNMTWMEWEREIN	648
16	QARVLAVERYLKDQQLLGIWGCSGKLICTTTVPWNTSWSNKSLEQIWDNMTWMEWEREIN	648
17	QARVLAVERYLKDQQLLGIWGCSGKLICTTTVPWNTSWSNKSLEQIWDNMTWMEWEREIN	648
18	QARVLAVERYLKDQQLLGIWGCSGKLICTTTVPWNTSWSNKSLEQIWDNMTWMEWEREIN	643
19	QARVLAVERYLKDQQLLGIWGCSGKLICTTTVPWNTSWSNKSLEQIWDNMTWMEWEREIN	648
20	QARVLAVERYLKDQQLLGIWGCSGKLICTTTVPWNTSWSNKSLEQIWDNMTWMEWEREIN	648
21	QARVLAVERYLKDQQLLGIWGCSGKLICTTTVPWNTSWSNKSLEQIWDNMTWMEWEREIN	648
22	QARVLAVERYLKDQQLLGIWGCSGKLICTTTVPWNTSWSNKSLEQIWDNMTWMEWEREIN	648
23	QARVLAVERYLKDQQLLGIWGCSGKLICTTTVPWNTSWSNKSLEQIWDNMTWMEWEREIN	648
24	QARVLAVERYLKDQQLLGIWGCSGKLICTTTVPWNTSWSNKSLEQIWDNMTWMEWEREIN	648
25	QARVLAVERYLKDQQLLGIWGCSGKLICTTTVPWNTSWSNKSLEQIWDNMTWMEWEREIN	648
26	QARVLAVERYLKDQQLLGIWGCSGKLICTTTVPWNTSWSNKSLEQIWDKMTWMEWEREID	648
27	QARVLAVERYLKDQQLLGIWGCSGKLICTTTVPWNTSWSNKSLEQIWDNMTWMEWEREIN	648
28	QARVLAVERYLKDQQLLGIWGCSGKLICTTTVPWNTSWSNKSLEQIWDNMTWMEWEREIN	648
29	QARVLAVERYLKDQQLLGIWGCSGKLICTTTVPWNTSWSNKSLEQIWDNMTWMEWEREIN	648
30	QARVLAVERYLKDQQLLGIWGCSGKLICTTTVPWNTSWSNKSLEQIWDNMTWMEWEREIN	648
31	QARVLAVERYLKDQQLLGIWGCSGKLICTTTVPWNTSWSNKSLEQIWDNMTWMEWEREIN	641
32	QARVLAVERYLKDQQLLGIWGCSGKLICTTTVPWNTSWSNKSLEQIWDNMTWMEWEREIN	648
33	QARVLAVERYLKDQQLLGIWGCSGKLICTTTVPWNTSWSNKSLEQIWDNMTWMEWEREIN	648
34	QARVLAVERYLKDQQLLGIWGCSGKLICTTTVPWNTSWSNKSLEQIWDNMTWMEWEREIN	648
35	QARVLAVERYLKDQQLLGIWGCSGKLICTTTVPWNTSWSNKSLEQIWDNMTWIEWEREIN	633

	Sequence	
	NYTGYIYQLIEESQNQQEKNEQELLAWDKWASLWNWFDITNWLWYIKIFIMIVGGLIGLR	
1	NYTGYIYQLIEESQNQQEKNEQELLALDKWASLWNWFDITNWLWYIKIFIMIVGGLIGLR	686
2	NYTGYIYQLIEESQNQQEQNEQELLAWDKWASLWNWFDITNWLWYIKIFIMIVGGLIGLR	706
3	NYTGYIYQLIEESQNQQEKNEQELLALDKWASLWNWFDITNWLWYIKIFIMIVGGLIGLR	708
4	NYTGYIYQLIEESQNQQEKNEQELLAWDKWASLWNWFDITNWLWYIKIFIMIVGGLIGLR	708
5	NYTGYIYQLIEESQNQQEKNEQELLAWDKWASLWNWFDITNWLWYIKIFIMIVGGLIGLR	708
6	NYTGYIYQLIEESQNQQEKNEQELLAWDKWASLWNWFDITNWLWYIKIFIMIVGGLIGLR	708
7	NYTGYIYQLIEESQNQQEKNEQELLAWDKWASLWNWFDITNWLWYIKIFIMIVGGLIGLR	708
8	NYTGYIYQLIEESQNQQEKNEQELLAWDKWASLWNWFDITNWLWYIKIFIMIVGGLIGLR	708
9	NYTGYIYQLIEESQNQQEKNEQELLAWDKWASLWNWFDITNWLWYIKIFIMIVGGLIGLR	708
10	NYTGYIYQLIEESQNQQEKNEQELLAWDKWASLWNWFDITNWLWYIKIFIMIVGGLIGLR	708
11	NYTGYIYQLIEESQNQQEKNEQELLALDKWASLWNWFDITNWLWYIKIFIMIVGGLIGLR	708
12	NYTGYIYQLIEESQNQQEKNEQELLAWDKWASLWNWFDITNWLWYIKIFIMIVGGLIGLR	691
13	NYTGYIYQLIEESQNQQEKNEQELLAWDKWAGLWNWFDITNWLWYIKIFIMIVGGLIGLR	708
14	NYTDYIYQLIEESQNQQEKNEQELLALDKWASLWNWFDITNWLWYIKIFIMIVGGLIGLR	709
15	NYTGYIYQLIEESQNQQEKNEQELLAWDKWASLWNWFDITNWLWYIKIFIMIVGGLIGLR	708
16	NYTGYIYQLIEESQNQQEKNEQELLAWDKWASLWNWFDITNWLWYIKIFIMIVGGLIGLR	708
17	NYTGYIYQLIEESQNQQEKNEQELLAWDKWASLWNWFDITNWLWYIKIFIMIVGGLIGLR	708
18	NYTGYIYQLIEESQNQQEKNEQELLAWDKWASLWNWFDITNWLWYIKIFIMIVGGLIGLR	703
19	NYTGYIYQLIEESQNQQEKNEQELLAWDKWASLWNWFDITNWLWYIKIFIMIVGGLIGLR	708
20	NYTGYIYQLIEESQNQQEKNEQELLAWDKWASLWNWFDITNWLWYIKIFIMIVGGLIGLR	708
21	NYTGYIYQLIEESQNQQEKNEQELLAWDKWASLWNWFDITNWLWYIKIFIMIVGGLIGLR	708
22	NYTGYIYQLIEESQNQQEKNEQELLAWDKWASLWNWFDITNWLWYIKIFIMIVGGLIGLR	708
23	NYTGYIYQLIEESQNQQEKNEQELLAWDKWASLWNWFDITNWLWYIKIFIMIVGGLIGLR	708
24	NYTGYIYQLIEESQNQQEKNEQELLAWDKWASLWNWFDITNWLWYIKIFIMIVGGLIGLR	708
25	NYTGYIYQLIEESQNQQEKNEQELLAWDKWASLWNWFDITNWLWYIKIFIMIVGGLIGLR	708
26	NYTDYIYQLIEESQNQQEKNEQELLAWDKWASLWNWFDITNWLWYIKIFIMIVGGLIGLR	708
27	NYTGYIYQLIEESQNQQEKNEQELLAWDKWASLWNWFDITNWLWYIKIFIMIVGGLIGLR	708
28	NYTGYIYQLIEESQNQQEKNEQELLAWDKWASLWNWFDITNWLWYIKIFIMIVGGLIGLR	708
29	NYTGYIYQLIEESQNQQEKNEQELLAWDKWASLWNWFDITNWLWYIKIFIMIVGGLIGLR	708
30	NYTGYIYQLIEESQNQQEKNEQELLAWDKWASLWNWFDITNWLWYIKIFIMIVGGLIGLR	708
31	NYTGYIYQLIEESQNQQEKNEQELLAWDKWASLWNWFDITNWLWYIKIFIMIVGGLIGLR	701
32	NYTGYIYQLIEESQNQQEKNEQELLAWDKWASLWNWFDITNWLWYIKIFIMIVGGLIGLR	708
33	NYTGYIYQLIEESQNQQEKNEQELLAWDKWASLWNWFDITNWLWYIKIFIMIVGGLIGLR	708
34	NYTGYIYQLIEESQNQQEKNEQELLAWDKWASLWNWFDITNWLWYIKIFIMIVGGLIGLR	708
35	NYTGYIYQLIEESQNQQEKNEQELLAWDKWASLWNWFDITNWLWYIKIFIMIVGGLIGLR	693

	**IVFTVLSIVNRVRQGYSPLSFQTHFPAQRGPDRPEGIGEEGGERDRDRSDPLVNGFLTLI**	
1	IVFTVLSIVNRVRQGYSPLSFQTHLPAQRGPDRPEGIGEEGGERDRDRSDPLVNGFLALI	746
2	IVFTVLSIVNRVRQGYSPLSFQTHFPAQRGPDRPEGIGEEGGERDRDRSDPLVNGFLTLI	766
3	IVFTVLSIVNRVRQGYSPLSFQTHFPAQRGPDRPEGIGEEGGERDRDRSEPLVNGFLTLI	768
4	IVFTVLSIVNRVRQGYSPLSFQTHFPAQRGPDRPEGIGEEGGERDRDRSDPLVNGFLTLI	768
5	IVFTVLSIVNRVRQGYSPLSFQTHFPAQRGPDRPEGIGEEGGERDRDRSDPLVNGFLTLI	768
6	IVFTVLSIVNRVRQGYSPLSFQTHFPAQRGPDRPEGIGEEGGERDRDRSDPLVNGFLTLI	768
7	IVFTVLSIVNRVRQGYSPLSFQTHFPAQRGPDRPEGIGEEGGERDRDRSDPLVNGFLTLI	768
8	IVFTVLSIVNRVRQGYSPLSFQTHFPAQRGPDRPEGIGEEGGERDRDRSDPLVNGFLTLI	768
9	IVFTVLSIVNRVRQGYSPLSFQTHFPAQRGPDRPEGIGEEGGERDRDRSDPLVNGFLTLI	768
10	IVFTVLSIVNRVRQGYSPLSFQTHFPAQRGPDRPEGIGEEGGERDRDRSDPLVNGFLTLI	768
11	IVFTVLSIVNRVRQGYSPLSFQTHFPAQRGPDRPEGIGEEGGERDRDRSDPLVNGFLTLI	768
12	IVFTVLSIVNRVRQGYSPLSFQTHLPAQRGPDRPEGIGEEGGERDRDRSDPLVNGFLTLI	751
13	IVFTVLSIVNRVRQGYSPLSFQTHFPAQRGPDRPEGIGEEGGERDRDRSDPLVNGFLTLI	768
14	IVFTVLSIVNRVRQGYSPLSFQTHFPAQRGPDRPEGIGEEGGERDRDRSDPLVNGFLTLI	769
15	IVFTVLSIVNRVRQGYSPLSFQTHFPAQRGPDRPEGIGEEGGERDRDRSDPLVNGFLTLI	768
16	IVFTVLSIVNRVRQGYSPLSFQTHFPAQRGPDRPEGIGEEGGERDRDRSDPLVNGFLTLI	768
17	IVFTVLSIVNRVRQGYSPLSFQTHFPAQRGPDRPEGIGEEGGERDRDRSDPLVNGFLTLI	768
18	IVFTVLSIVNRVRQGYSPLSFQTHLPAQRGPDRPEGIGEEGGERDRDRSDPLVNGFLTLI	763
19	IVFTVLSIVNRVRQGYSPLSFQTHFPAQRGPDRPEGIGEEGGERDRDRSDPLVNGFLTLI	768
20	IVFTVLSIVNRSRQGYSPLSFQTHFPAQRGPDRPEGIGEEGGERDRDRSDPLVNGFLTLI	768
21	IVFTVLSIVNRVRQGYSPLSFQTHFPAQRGPDRPEGIGEEGGERDRDRSDPLVNGFLTLI	768
22	IVFTVLSIVNRVRQGYSPLSFQTHFPAQRGPDRPEGIGEEGGERDRDRSDPLVNGFLTLI	768
23	IVFTVLSIVNRVRQGYSPLSFQTHFPAQRGPDRPEGIGEEGGERDRDRSDPLVNGFLTLI	768
24	IVFTVLSIVNRVRQGYSPLSFQTHFPAQRGPDRPEGIGEEGGERDRDRSDPLVNGFLTLI	768
25	IVFTVLSIVNRVRQGYSPLSFQTHFPAQRGPDRPEGIGEEGGERDRDRSDPLVNGFLTLI	768
26	IVFTVLSIVNRVRQGYSPLSFQTHFPAQRGPDRPEGIGEEGGERDRDRSDPLVNGFLTLI	768
27	IVFTILSIVNRVRQGYSPLSFQTHLPAQRGPDRPEGIGEEGGERDRDRSDPLVNGFLTLI	768
28	IVFTVLSIVNRVRQGYSPLSFQTHFPAQRGPDRPEGIGEEGGERDRDRSDPLVNGFLTLI	768
29	IVFTVLSIVNRVRQGYSPLSFQTHFPAQRGPDRPEGIGEEGGERDRDRSDPLVNGFLTLI	768
30	IVFTVLSIVNRVRQGYSPLSFQTHFPAQRGPDRPEGIGEEGGERDRDRSDPLVNGFLTLI	768
31	IVFTVLSIVNRVRQGYSPLSFQTHFPAQRGPDRPEGIGEEGGERDRDRSDPLVNGFLTLI	761
32	IVFTVLSIVNRVRQGYSPLSFQTHFPAQRGPDRPEGIGEEGGERDRDRSDPLVNGFLTLI	768
33	IVFTVLSIVNRVRQGYSPLSFQTHFPAQRGPDRPEGIGEEGGERDRDRSDPLVNGFLTLI	768
34	IVFTVLSIVNRVRQGYSPLSFQTHFPAQRGPDRPEGIGEEGGERDRDRSDPLVNGFLTLI	768
35	IVFTVLSIVNRVRQGYSPLSFQTHFPAQRGPDRPEGIGEEGGERDRDRSDPLVNGFLTLI	753

	WSDLRSLCLFSYHRLRDLLLIATRIVELLGR-------RGWEVLKYWWNLLQYWSQELKK	
1	WSDLRSLCLFSYHRLRDLLLIVTRIVELLGR-------RGWEVLKYWWNLLQYWSQELKN	799
2	WSDLRSLCLFSYHHLRDLLLIVTRIVELLGR-------RGWEVLKYWWNLLQYWSQELKD	819
3	WSDLRSLCLFSYHHLRDLLLIVTRIVELLGR-------RGWEVLKYWWNLLQYWSQELKK	821
4	WSDLRSLCLFSYHRLRDLLLIATRIVELLGR-------RGWEVLKYWWNLLQYWSQELKK	821
5	WSDLRSLCLFSYHRLRDLLLIATRIVELLGR-------RGWEVLKYWWNLLQYWSQELKK	821
6	WSDLRSLCLFSYHRLRDLLLIATRIVELLGR-------RGWEVLKYWWNLLQYWSQELKK	821
7	WSDLRSLCLFSYHRLRDLLLIATRIVELLGR-------RGWEVLKYWWNLLQYWSQELKK	821
8	WSDLRSLCLFSYHRLRDLLLIATRIVELLGR-------RGWEVLKYWWNLLQYWSQELKK	821
9	WSDLRSLCLFSYHRLRDLLLIATRIVELLGR-------RGWEVLKYWWNLLQYWSQELKK	821
10	WSDLRSLCLFSYHRLRDLLLIATRIVELLGR-------RGWEVLKYWWNLLQYWSQELKK	821
11	WSDLRSLCLFSYHHLRDLLLIVTRIVELLGR-------RGWEVLKYWWNLLQYWSQELKK	821
12	WSDLRSLCLFSYHRLRDLLLIATRIVELLGR-------RGWEVLKYWWNLLQYWSQELKK	804
13	WSDLRSLCLFSYHHLRDLLLIVTRIVELLGR-------RGWEVLKYWWNLLQYWGQELKN	821
14	WSDLRSLCLFSYHRLRDLLLIATRIVELLGR-------RGWEVLKYWWNLLQYWSQELKK	822
15	WSDLRSLCLFSYHRLRDLLLIATRIVELLGR-------RGWEVLKYWWNLLQYWSQELKK	821
16	WSDLRSLCLFSYHRLRDLLLIATRIVELLGR-------RGWEILKYWWNLLQYWSQELKK	821
17	WSDLRSLCLFSYHRLRDLLLIATRIVELLGR-------RGWEVLKYWWNLLQYWSQELKK	821
18	WSDLRSLCLFSYHHLRDLLLIVTRIVELLGR-------RGWEVLKYWWNLLQYWSQELKD	816
19	WSDLRSLCLFSYHRLRDLLLIATRIVELLGR-------RGWEVLKYWWNLLQYWSQELKK	821
20	WSDLRSLCLFSYHRLRDLLLIATRIVELLGR-------RGWEVLKYWWNLLQYWSQELKK	821
21	WSDLRSLCLFSYHRLRDLLLIATRIVELLGR-------RGWEILKYWWNLLQYWSQELKK	821
22	WSDLRSLCLFSYHRLRDLLLIATRIVELLGR-------RGWEVLKYWWNLLQYWSQELKK	821
23	WSDLRSLCLFSYHRLRDLLLIATRIVELLGR-------RGWEVLKYWWNLLQYWSQELKK	821
24	WSDLRSLCLFSYHRLRDLLLIATRIVELLGR-------RGWEVLKYWWNLLQYWSQELKK	821
25	WSDLRSLCLFSYHRLRDLLLIATRIVELLGR-------RGWEVLKYWWNLLQYWSQELKK	821
26	WSDLRSLCLFSYHRLRDLLLIATRIVELLGR-------RGWEVLKYWWNLLQYWSQELKK	821
27	WSDLRSLCLFSYHHLRDLLLIATRIVELLGR-------RGWEVLKYWWNLLQYWSQELKH	821
28	WSDLRSLCLFSYHRLRDLLLIATRIVELLGR-------RGWEVLKYWWNLLQYWSQELKK	821
29	WSDLRSLCLFSYHRLRDLLLIATRIVELLGR-------RGWEVLKYWWNLLQYWSQELKK	821
30	WSDLRSLCLFSYHHLRDLLLIVTRIVELLGR-------RGWEVLKYWWNLLQYWSQELKK	821
31	WSDLRSLCHFSYHRLRDLLLIATRIVELLGR-------RGWEVLKYWWNLLQYWSQELKN	814
32	WSDLRSLCLFSYHRLRDLLLIATRIVELLGR-------RGWEILKYWWNLIQYWSQELKK	821
33	WSDLRSLCLFSYHRLRDLLLIATRIVELLGR-------RGWEVLKYWWNLLQYWGQELKN	821
34	WSDLRSLCLFSYHRLRDLLLIATRIVELLGR-------RGWEVLKYWWNLLQYWSQELKK	821
35	WSDLRSLCLFSYHHLRDLLLIATRIVELLGR-------RGWEVLKYWWNLLQYWSQELKK	806

	Sequence	
	SAVSLLNATAIAVAEGTDRVIEVVQRAGRAIRHIPRRIRQGLERALL	
1	SAVSLLNATAIAVAEGTDRVIEVVQRACRAILHIPRRIRQGLERALL	846
2	SAVSLLNATAIAVAEGTDRVIEVVQRAGRAIRHIPRRIRQGLERALL	866
3	SAVSLLNATAIAVAEGTDRVIEVVQRAGRAIRHIPRRIRQGLERALL	868
4	SAVSLLNATAIAVAEGTDRVIEVVQRAGRAIRHIPRRIRQGLERALL	868
5	SAVSLLNATAIAVAEGTDRVIEVVQRAGRAIRHIPRRIRQGLERALL	868
6	SAVSLLNATAIAVAEGTDRVIEVVQRAGRAIRHIPRRIRQGLERALL	868
7	SAVSLLNATAIAVAEGTDRVIEVVQRAGRAIRHIPRRIRQGLERALL	868
8	SAVSLLNATAIAVAEGTDRVIEVVQRAGRAIRHIPRRIRQGLERALL	868
9	SAVSLLNATAIAVAEGTDRVIEVVQRAGRAIRHIPRRIRQGLERALL	868
10	SAVSLLNATAIAVAEGTDRVIEVVQRAGRTIRHIPRRIRQGLERALL	868
11	SAVSLLNATAIAVAEGTDRVIEVVQRAGRAIRHIPRRIRQGLERALL	868
12	SAVSLLNATAVAVAEGTDRVIEVVQRAGRTIRHIPRRIRQGLERALL	851
13	SAVSLLNATAIAVAEGTDRVIEVVQRAGRAIRHIPRRIRQGLERALL	868
14	SAVSLLNATAIAVAEGTDRVIEVVQRAGRAIRHIPRRIRQGLERALL	869
15	SAVSLLNATAIAVAEGTDRVIEVVQRAGRAIRHIPRRIRQGLERALL	868
16	SAVSLLNATAIAVAEGTDRVIEVVQRAGRAIRHIPRRIRQGLERALL	868
17	SAVSLLNATAIAVAEGTDRVIEVVQRAGRTIRHIPRRIRQGLERALL	868
18	SAVSLLNATAIAVAEGTDRVIEVVQRAGRAIRHIPRRIRQGLERALL	863
19	SAVSLLNATAIAVAEGTDRVIEVVQRAGRAIRHIPRRIRQGLERALL	868
20	SAVSLLNATAIAVAEGTDRVIEVVQRAGRAIRHIPRRIRQGLERALL	868
21	SAVSLLNATAIAVAEGTDRVIEVVQRAGRAIRHIPRRIRQGLERALL	868
22	SAVSLLNATATAVAEGTDRVIEVVQRAGRAIRHIPRRIRQGLERALL	868
23	SAVSLLNATAIAVAEGTDRVIEVVQRAGRAIRHIPRRIRQGLERALL	868
24	SAVSLLNATAIAVAEGTDRVIEVVQRAGRAIRHIPRRIRQGLERALL	868
25	SAVSLLNATAIAVAEGTDRVIEVVQRAGRAIRHIPRRIRQGLERALL	868
26	SAVSLLNATAIAVAEGTDRVIEVVQRAGRAIRHIPRRIRQGLERALL	868
27	SAVSLLNATAIAVAEGTDRVIEVVQRAGRAIRHIPRRIRQGLERALL	868
28	SAVSLLNATAIAVAEGTDRVIEVVQRAGRAIRHIPRRIRQGLERALL	868
29	SAVSLLNATAIAVAEGTDRVIEVVQRAGRAIRHIPRRIRQGLERALL	868
30	SAVSLLNATAIAVAEGTDRVIEVVQRAGRAIRHIPRRIRQGLERALL	868
31	SAVSLLNATAIAVAEGTDRVIEVVQRAGRAIRHIPRRIRQGLERALL	861
32	SAVSLLNATAIAVAEGTDRVIEVVQRAGRAIRHIPRRIRQGLERALL	868
33	SAVSLLNATAIAVAEGTDRIIEVVQRAGRAIRHIPRRIRQGLERALL	868
34	SAVSLLNATAIAVAEGTDRVIEVVQRAGRAIRHIPRRIRQGLERALL	868
35	SAVSLLNATAIAVAEGTDRVIEVVQRAGRAIRHIPRRIRQGLERALL	853

Consensus

1. B.US.2006.700010040_C9_4520.EU289193
2. B.US.2011.CH0040_3_d1737_ipe032_25_13.MG900546
3. B.US.2011.CH0040_3_d1737_ipe032_25_14.MG900547
4. B.US.2011.CH0040_3_d1737_ipe032_25_19.MG900548
5. B.US.2011.CH0040_3_d1737_ipe032_25_20.MG900549
6. B.US.2011.CH0040_3_d1737_ipe032_25_21.MG900550
7. B.US.2011.CH0040_3_d1737_ipe032_25_22.MG900551
8. B.US.2011.CH0040_3_d1737_ipe032_25_24.MG900552
9. B.US.2011.CH0040_3_d1737_ipe032_25_25.MG900553
10. B.US.2011.CH0040_3_d1737_ipe032_25_26.MG900554
11. B.US.2011.CH0040_3_d1737_ipe032_25_27.MG900555
12. B.US.2011.CH0040_3_d1737_ipe032_25_28.MG900556
13. B.US.2011.CH0040_3_d1737_ipe032_25_31.MG900557
14. B.US.2011.CH0040_3_d1737_ipe032_25_33.MG900558
15. B.US.2011.CH0040_3_d1737_ipe032_25_34.MG900559
16. B.US.2011.CH0040_3_d1737_ipe032_25_37.MG900560
17. B.US.2011.CH0040_3_d1737_ipe032_25_38.MG900561
18. B.US.2011.CH0040_3_d1737_ipe032_25_41.MG900562
19. B.US.2011.CH0040_3_d1737_ipe032_25_44.MG900563
20. B.US.2011.CH0040_3_d1737_ipe032_25_45.MG900564
21. B.US.2011.CH0040_3_d1737_ipe032_25_62.MG900565
22. B.US.2011.CH0040_3_d1737_ipe032_25_63.MG900566
23. B.US.2011.CH0040_3_d1737_ipe032_25_66.MG900567
24. B.US.2011.CH0040_3_d1737_ipe032_25_67.MG900568
25. B.US.2011.CH0040_3_d1737_ipe032_25_69.MG900569
26. B.US.2011.CH0040_3_d1737_ipe032_25_71.MG900570
27. B.US.2011.CH0040_3_d1737_ipe032_25_72.MG900571
28. B.US.2011.CH0040_3_d1737_ipe032_25_73.MG900572
29. B.US.2011.CH0040_3_d1737_ipe032_25_74.MG900573
30. B.US.2011.CH0040_3_d1737_ipe032_25_77a.MG900574
31. B.US.2011.CH0040_3_d1737_ipe032_25_78.MG900575
32. B.US.2011.CH0040_3_d1737_ipe032_25_79.MG900576
33. B.US.2011.CH0040_3_d1737_ipe032_25_80.MG900577
34. B.US.2011.CH0040_3_d1737_ipe032_25_81.MG900578
35. B.US.2011.CH0040_3_d1737_ipe032_25_82.MG900579
36. B.US.2011.CH0040_3_d1737_ipe032_25_86.MG900580
37. B.US.2011.CH0040_3_d1737_ipe032_25_87.MG900581
38. B.US.2011.CH0040_3_d1737_ipe032_27_09.MG900582
39. B.US.2011.CH0040_3_d1737_ipe032_27_12.MG900583
40. B.US.2011.CH0040_3_d1737_ipe032_9_01.MG900584
41. B.US.2011.CH0040_3_d1737_ipe032_9_04.MG900585
42. B.US.2011.CH0040_3_d1737_ipe032_9_05.MG900586

	MRVMGIRKNYQHLWREGILLLGILMICSAADXLWVTVYYGVPVWREATTTLFCASDAKAY	
1	MRVMGIRKNYQHLWREGILLLGILMICSAADNLWVTVYYGVPVWREATTTLFCASDAKAY	60
2	MRVMGIRKNYQHLWREGTLLLGILMICSAEDNLWVTVYYGVPVWREATTTLFCASDAKAY	60
3	MRVMGIRKNYQHLWREGILLLGILMICSAADNLWVTVYYGVPVWREATTTLFCASDAKAY	60
4	MRVMGIRKNYQHLWREGILLLGILMICSAAGNLWVTVYYGVPVWREATTTLFCASDAKAY	60
5	MRVMGIRKNYQHLWREGILFLGILMICSAADNLWVTVYYGVPVWKEATTTLFCASDAKAY	60
6	MRVMGIRKNYQHLWRKGILLLGILMICSAADKLWVTVYYGVPVWREATTTLFCASDAKAY	60
7	MRVMGIRKNYQHWWREGILLLGILMICSAADKLWVTVYYGVPVWREATTTLFCASDAKAY	60
8	MRVMGIRKNYQHLWREGILLLGILMICSAADNLWVTVYYGVPVWREATTTLFCASDAKAY	60
9	MRVMGIRKNYQHLWREGTLLLGILMICSAEDNLWVTVYYGVPVWREATTTLFCASDAKAY	60
10	MRVMGIRKNYQHLWREGILLLGILMICSAADNLWVTVYYGVPVWREATTTLFCASDAKAY	60
11	MRVMGIRKNYQHLWREGILLLGILMICSAADNSWVTVYYGVPVWKEATTTLFCASDAKAY	60
12	MRVMGIRKNYQHWWREGILLLGILMICSAADKLWVTVYYGVPVWREATTTLFCASDAKAY	60
13	MRVMGIRKNYQHLWRKGILLLGILMICSAADKLWVTVYYGVPVWREATTTLFCASDAKAY	60
14	MRVMGIRKNYQHLWRKGILLLGILMICSAADKLWVTVYYGVPVWREATTTLFCASDAKAY	60
15	MRVMGIRKNYQHLWRKGILLLGILMICSAADKLWVTVYYGVPVWREATTTLFCASDAKAY	60
16	MRVMGIRKNYQHLWREGILLLGILMICSAADNLWVTVYYGVPVWKEATTTLFCASDAKAY	60
17	MRVMGIRKNYQHLWREGILLLGILMICSAADNLWVTVYYGVPVWREATTTLFCASDAKAY	60
18	MRVMGIRKNYQHLWREGILLLGILMICSAADDLWVTVYYGVPVWREATTTLFCASDAKAY	60
19	MRVMGIRKNYQHLWREGILLLGILMICSAADNLWVTVYYGVPVWKEATTTLFCASDAKAY	60
20	MRVMGIRKNYQHWWREGILLLGILMICSAADKLWVTVYYGVPVWREATTTLFCASDAKAY	60
21	MRVMGIRKNYQHLWREGILLLGILMICSAADNLWVTVYYGVPVWKEATTTLFCASDAKAY	60
22	MRVMGIRKNYQHLWREGILLLGILMICSAADNLWVTVYYGVPVWREATTTLFCASDAKAY	60
23	MRVMGIRKNYQHLWRKGILLLGILMICSAADKLWVTVYYGVPVWREATTTLFCASDAKAY	60
24	MRVMGIRKNYQHLWREGILLLGILMICSAADNLWVTVYYGVPVWREATTTLFCASDAKAY	60
25	MRVMGIRKNYQHLWRKGILLLGILMICSAADKLWVTVYYGVPVWREATTTLFCASDAKAY	60
26	MRVMGIRKNYQHLWREGILLLGILMICSAADDLWVTVYYGVPVWREATTTLFCASDAKAY	60
27	MRVMGIRKNYQHWWREGILLLGILMICSAADKLWVTVYYGVPVWREATTTLFCASDAKAY	60
28	MRVMGIRKNYQHWWREGILLLGILMICSAADKLWVTVYYGVPVWREATTTLFCASDAKAY	60
29	MRVMGIRKNYQHLWRKGILLLGILMICSAADKLWVTVYYGVPVWREATTTLFCASDAKAY	60
30	MRVMGIRKNYQHWWREGILLLGILMICSAADKLWVTVYYGVPVWREATTTLFCASDAKAY	60
31	MRVMGIRKNYQHLWRKGILLLGILMICSAADKLWVTVYYGVPVWREATTTLFCASDAKAY	60
32	MRVMGIRKNYQHWWREGILLLGILMICSAADKLWVTVYYGVPVWREATTTLFCASDAKAY	60
33	MRVMGIRKNYQHLWREGILLLGILMICSAADNLWVTVYYGVPVWKEATTTLFCASDAKAY	60
34	MRVMEIRKNYQHWWREGILLLGILMICSAADKLWVTVYYGVPVWREATTTLFCASDAKAY	60
35	MRVMGIRKNYQHLWREGILLLGILMICSAADNLWVTVYYGVPVWREATTTLFCASDAKAY	60
36	MRVMGIRKNYQHLWRKGILLLGILMICSAADKLWVTVYYGVPVWREATTTLFCASDAKAY	60
37	MRVMGIRKNYQHWWREGILLLGILMICSAADKLWVTVYYGVPVWREATTTLFCASDAKAY	60
38	MRVMGIRKNYQHWWREGILLLGILMICSAADKLWVTVYYGVPVWREATTTLFCASDAKAY	60
39	MRVMGIRKNYQHLWREGILLLGILMICSAADNLWVTVYYGVPVWREATTTLFCASDAKAY	60
40	MRVMGIRKNYQHLWREGILLLGILMICSAADNLWVTVYYGVPVWREATTTLFCASDAKAY	60
41	MRVMGIRKNYQHLWRKGILLLGILMICSAADKLWVTVYYGVPVWREATTTLFCASDAKAY	60
42	MRVMGIRKNYQHLWREGILLLGILMICSAADNLWVTVYYGVPVWREATTTLFCASDAKAY	60

	DTEAHNVWATHACVPTDPNPQEVELKNVTENFNMWENNMVEQMHEDIISLWDQSLKPCVK	
1	DTEAHNVWATHACVPTDPNPQEVELKNVTENFNMWENNMVEQMHEDIISLWDQSLKPCVK	120
2	DTEAHNVWATHACVPTDPNPQEVELKNVTENFNMWENNMVEQMHEDIISLWDQSLKPCVK	120
3	DTEAHNVWATHACVPTDPNPQEVELKNVTENFNMWENNMVEQMHEDIISLWDQSLKPCVK	120
4	DTEAHNVWATHACVPTDPNPQEVELKNVTENFNMWENNMVEQMHEDIISLWDQSLKPCVK	120
5	DTEAHNVWATHACVPTDPNPQEVELKNVTENFNMWKNNMVEQMHEDIISLWDQSLKPCVK	120
6	DTEAHNVWATHACVPTDPNPQEVELKNVTENFNMWENNMVEQMHEDIISLWDQSLKPCVK	120
7	DTEAHNVWATHACVPTDPNPQEVELKNVTENFNMWENNMVEQMHEDIISLWDQSLKPCVK	120
8	DTEAHNVWATHACVPTDPNPQEVELKNVTENFNMWENNMVEQMHEDTISLWDQSLKPCVK	120
9	DTEAHNVWATHACVPTDPNPQEVELKNVTENFNMWENNMVEQMHEDIISLWDQSLKPCVK	120
10	DTEAHNVWATHACVPTDPNPQEVELKNVTENFNMWENNMVEQMHEDIISLWDQSLKPCVK	120
11	DTEAHNVWATHACVPTDPNPQEVELKNVTENFNMWKNNMVEQMHEDIISLWDQSLKPCVK	120
12	DTEAHNVWATHACVPTDPNPQEVELKNVTENFNMWENNMVEQMHEDIISLWDQSLKPCVK	120
13	DTEAHNVWATHACVPTDPNPQEVELRNVTENFNMWENNMVEQMHEDIISLWDQSLKPCVK	120
14	DTEAHNVWATHACVPTDPNPQEVELKNVTENFNMWENNMVEQMHEDIISLWDQSLKPCVK	120
15	DTEAHNVWATHACVPTDPNPQEVELKNVTENFNMWENNMVEQMHEDIISLWDQSLKPCVK	120
16	DTEAHNVWATHACVPTDPNPQEVELKNVTENFNMWKNNMVEQMHEDIISLWDQSLKPCVK	120
17	DTEAHNVWATHACVPTDPNPQEVELKNVTENFNMWENNMVEQMHEDIISLWDQSLKPCVK	120
18	DTEAHNVWATHACVPTDPNPQEVELKNVTENFNMWENNMVEQMHEDIISLWDQSLKPCVK	120
19	DAEAHNVWATHACVPTDPNPQEVELKNVTENFNMWKNNMVEQMHEDIISLWDQSLKPCVK	120
20	DTEAHNVWATHACVPTDPNPQEVELKNVTENFNMWENNMVEQMHEDIISLWDQSLKPCVK	120
21	DTEAHNVWATHACVPTDPNPQEVELKNVTENFNMWKNNMVEQMHEDIISLWDQSLKPCVK	120
22	DTEAHNVWATHACVPTDPNPQEVELKNVTENFNMWENNMVEQMHEDIISLWDQSLKPCVK	120
23	DTEAHNVWATHACVPTDPNPQEVELKNVTENFNMWENNMVEQMHEDIISLWDQSLKPCVK	120
24	DTEAHNVWATHACVPTDPNPQEVELKNVTENFNMWENNMVEQMHEDIISLWDQSLKPCVK	120
25	DTEAHNVWATHACVPTDPNPQEVELKNVTENFNMWENNMVEQMHEDIISLWDQSLKPCVK	120
26	DTEAHNVWATHACVPTDPNPQEVELKNVTENFNMWENNMVEQMHEDIISLWDQSLKPCVK	120
27	DTEAHNVWATHACVPTDPNPQEVELKNVTENFNMWENNMVEQMHEDIISLWDQSLKPCVK	120
28	DTEAHNVWATHACVPTDPNPQEVELKNVTENFNMWENNMVEQMHEDIISLWDQSLKPCVK	120
29	DTEAHNVWATHACVPTDPNPQEVELKNVTENFNMWENNMVEQMHEDIISLWDQSLKPCVK	120
30	DTEAHNVWATHACVPTDPNPQEVELKNVTENFNMWENNMVEQMHEDIISLWDQSLKPCVK	120
31	DTEAHNVWATHACVPTDPNPQEVELKNVTENFNMWENNMVEQMHEDIISLWDQSLKPCVK	120
32	DTEAHNVWATHACVPTDPNPQEVELKNVTENFNMWENNMVEQMHEDIISLWDQSLKPCVK	120
33	DTEAHNVWATHACVPTDPNPQEVELKNVTENFNMWKNNMVEQMHEDIISLWDQSLKPCVK	120
34	DTEAHNVWATHACVPTDPNPQEVELKNVTENFNMWENNMVEQMHEDIISLWDQSLKPCVK	120
35	DTEAHNVWATHACVPTDPNPQEVELKNVTENFNMWENNMVEQMHEDIISLWDQSLKPCVK	120
36	DTEAHNVWATHACVPTDPNPQEVELKNVTENFNMWENNMVEQMHEDIISLWDQSLKPCVK	120
37	DTEAHNVWATHACVPTDPNPQEVELKNVTENFNMWENNMVEQMHEDIISLWDQSLKPCVK	120
38	DTEAHNVWATHACVPTDPNPQEVELKNVTENFNMWENNMVEQMHEDIISLWDQSLKPCVK	120
39	DTEAHNVWATHACVPTDPNPQEVELKNVTENFNMWKNNMVEQMHEDIISLWDQSLKPCVK	120
40	DTEAHNVWATHACVPTDPNPQEVELKNVTENFNMWENNMVEQMHEDIISLWDQSLKPCVK	120
41	DTEAHNVWATHACVPTDPNPQEVELKNVTENFNMWENNMVEQMHEDIISLWDQSLKPCVK	120
42	DTEAHNVWATHACVPTDPNPQEVELKNVTENFNMWENNMVEQMHEDIISLWDQSLKPCVK	120

```
    LTPLCVTLNCTDXXXXTTTTXXXNXTXXXXXXNXTXXXXXX---XXXNEXMMEKGEVKNC
1   LTPLCVTLNCTDLGNVTNTT-------------------------NSNGEMMEKGEVKNC   155
2   LTPLCVTLNCTDNVTTTTNRNGTNCTDSGNVTNTTNRNGTL---MGNETLMMEKGEVKNC   177
3   LTPLCVTLNCTDLGNVTTTT-------------------------DRNEKMMEKEEV-NC   154
4   LTPLCVTLNCTDLGNVTTTT-------------------------DRNEKMMEKEEV-NC   154
5   LTPLCVTLNCTDLENVTATTN------------------------SNGTLMEEEGEVKNC   156
6   LTPLCVTLNCTDDLDNVTTTTNRNGTNCTDSGNVTPTTNRNGT-LMGNETLMEKGEVKNC   179
7   LTPLCVTLNCTDNVTTTTNRNGTNCTDSGNVTNTTNRNGTL---MGNETLMMERGEVKNC   177
8   LTPLCVTLNCTDLGNVTTTT-------------------------DRNEKMMEKEEV-NC   154
9   LTPLCVTLNCTDNVTTTTNRNGTNCTDSGNVTNTTNRNGTL---MGNETLMMERGEVKNC   177
10  LTPLCVTLNCTDLGNVTTTT-------------------------DRNEKMMEKEEV-NC   154
11  LTPLCVTLNCTDLENVTATTN------------------------SNGTLMEEEGEVKNC   156
12  LTPLCVTLNCTDDLDNVTTTTNRNGTNCTDSGNVTPTTNRNGT-LMGNETLMEKGEVKNC   179
13  LTPLCVTLNCTDDLDNVTTTTNRNGTNCTDSGNVTPTTNRNGT-LMGNETLMEKGEVKNC   179
14  LTPLCVTLNCTDDLDNVTTTTNRNGTNCTDSGNVTPTTNRNGT-LMGNETLMEKGEVKNC   179
15  LTPLCVTLNCTDDLDNVTTTTNRNGTNCTDSGNVTPTTNRNGT-LMGNETLMEKGEVKNC   179
16  LTPLCVTLNCTDLENDTITT-------------------------NSNGTSMEEGEVKNC   155
17  LTPLCVTLNCTDLGNVTTTT-------------------------DRNEKMMEKEEV-NC   154
18  LTPLCVTLNCTDNVTTTTNRNGTNCTDSGNVTNTTNRNGTL---MGNETLMMEKGEVKNC   177
19  LTPLCVTLNCTDTTTTN-----------------------------SNGTSMEEGEVKNC   151
20  LTPLCVTLNCTDNVTTTTNRNGTNCTDSGNVTNTTNRNGTL---MGNETLMMERGEVKNC   177
21  LTPLCVTLNCTDTTTTN-----------------------------SNGTSMEEGEVKNC   151
22  LTPLCVTLNCTDLGNVTTTT-------------------------DRNEKMMEKEEV-NC   154
23  LTPLCVTLNCTDDLDNVTTTTNRNGTNCTDSGNVTPTTNRNGT-LMGNETLMEKGEVKNC   179
24  LTPLCVTLNCTDLGNVTTTT-------------------------DRNEKMMEKEEV-NC   154
25  LTPLCVTLNCTDNVTTTTNRNGTNCTDSGNVTNTTNRNGTL---MGNETLMMERGEVKNC   177
26  LTPLCVTLNCTDDLDNVTTTTNRNGTNCTDSGNVTPTTNRNGT-LMGNETLMEKGEVKNC   179
27  LTPLCVTLNCTDDLDNVTTTTNRNGTNCTDSGNVTNTTNRNGTLMGNETLMMERGEVKNC   180
28  LTPLCVTLNCTDNVTTTTNRNGTNCTDLRNDTAT----------TNSNETLMEKGEVKNC   170
29  LTPLCVTLNCTDDLDNVTTTTNRNGTNCTDSGNVTPTTNRNGT-LMGNETLMEKGEVKNC   179
30  LTPLCVTLNCTDNVTTTTNRNGTNCTDSGNVTNTTNRNGTL---MGNETLMMEEGEVKNC   177
31  LTPLCVTLNCTDNVTTTTNRNGTNCTDSGNVTNTTNRNGTL---MGNETLMMERGEVKNC   177
32  LTPLCVTLNCTDNVTTTTNRNGTNCTDSGNVTNTTNRNGTL---MGNETLMMERGEVKNC   177
33  LTPLCVTLNCTDLENDTTTT---------------------------NGTSMEEGEVKNC   153
34  LTPLCVTLNCTDNVTTTTNRNGTNCTDSGNVTNTTNRNGTL---MGNETLMMERGEVKNC   177
35  LTPLCVTLNCTDLGNVTTTT-------------------------DRNEKMMEKEEV-NC   154
36  LTPLCVTLNCTDDLDNVTTTTNRNGTNCTDSGNVTPTTNRNGT-LMGNETLMEKGEVKNC   179
37  LTPLCVTLNCTDDLDNVTTTTNRNGTNCTDSGNVTPTTNRNGT-LMGNETLMEKGEVKNC   179
38  LTPLCVTLNCTDDLDNVTTTTNRNGTNCTDSGNVTPTTNRNGT-LMGNETLMEKGEVKNC   179
39  LTPLCVTLNCTDLENDTTTI-------------------------NSNGTSMEEGEVKNC   155
40  LTPLCVTLNCTDLGNVTTTT-------------------------DRNEKMMEKEEV-NC   154
41  LTPLCVTLNCTDDLDNVTTTTNRNGTNCTDSGNVTPTTNRNGT-LMGNETLMEKGEVKNC   179
42  LTPLCVTLNCTDLGNVTTTT-------------------------DRNEKMMEKEEV-NC   154
```

	Sequence	
	SFRITTDMQDRTRKEYALFYKLDVVPIND-------TRYRLVSCNTSVITQACPKVSFEP	
1	SFKITTDIKDRTRKEYALFYKLDVVPIND-------TRYRLVSCNTSVITQACPKVSFEP	208
2	SFRITTDMQDRTRKEYALFYKLDVVPIND-------TSYRLVSCNTSVITQACPKVSFEP	230
3	SFKITTDVKDRMRKEYALFYKLDVVPIND-------TRYRLISCNTSVITQACPKVSFEP	207
4	SFKITTDVKDRMRKEYALFYKLDVVPIND-------TRYRLISCNTSVITQACPKVSFEP	207
5	SFKITTEIKDRTRKEYALFYKLDVVPIND-------TRYRLVSCNTSVITQACPKVSFEP	209
6	SFRITTDMQDRTRKEYALFYKLDVVPIND-------TRYRLVSCNTSVITQACPKVSFEP	232
7	SFRITTDMQDRTRKEYALFYKLDVVPIND-------TSYRLVSCNTSVITQACPKVSFEP	230
8	SFKITTDVKDRMRKEYALFYKLDVVPIND-------TRYRLISCNTSVITQACPKVSFEP	207
9	SFRITTDMQDRTRKEYALFYKLDVVPIND-------TSYRLVSCNTSVITQACPKVSFEP	230
10	SFKITTDVKDRMRKEYALFYKLDVVPIND-------TRYRLISCNTSVITQACPKVSFEP	207
11	SFKITTEIKDRTRKEYALFYKLDVVPIND-------TRYRLVSCNTSVITQACPKVSFEP	209
12	SFRITTDMQDRTRKEYALFYKLDVVPIND-------TRYRLVSCNTSVITQACPKVSFEP	232
13	SFRITTDMQDRTRKEYALFYKLDVVPIND-------TRYRLVSCNTSVITQACPKVSFEP	232
14	SFRITTDMQDRTRKEYALFYKLDVVPIND-------TSYRLVSCNTSVITQACPKVSFEP	232
15	SFRITTDMQDRTRKEYALFYKLDVVPIND-------TRYRLVSCNTSVITQACPKVSFEP	232
16	SFKITTEIKDRTRKEYALFYKLDVVPIND-------TRYRLVSCNTSVITQACPKVSFEP	208
17	SFKITTDVKDRMRKEYALFYKLDVVPIND-------TRYRLISCNTSVITQACPKVSFEP	207
18	SFRITTDMQDRTRKEYALFYKLDVVPIND-------TSYRLVSCNTSVITQACPKVSFEP	230
19	SFKITTEMKDRTRKEYALFYKLDVVPIND-------TRYRLVSCNTSVLTQACPKVSFEP	204
20	SFRITTDMQDRTRKEYALFYKLDVVPIND-------TSYRLVSCNTSVITQACPKVSFEP	230
21	SFKITTEMKDRTRKEYALFYKLDVVPIND-------TRYRLVSCNTSVLTQACPKVSFEP	204
22	SFKITTDVKDRMRKEYALFYKLDVVPIND-------TRYRLISCNTSVITQACPKVSFEP	207
23	SFRITTDMQDRTRKEYALFYKLDVVPIND--TPINDTSYRLVSCNTSVITQACPKVSFEP	237
24	SFKITTDVKDRMRKEYALFYKLDVVPIND-------TRYRLISCNTSVITQACPKVSFEP	207
25	SFRITTDMQDRTRKEYALFYKLDVVPIND-------TSYRLVSCNTSVITQACPKVSFEP	230
26	SFRITTDMQDRTRKEYALFYKLDVVPIND-------TRYRLVSCNTSVITQACPKVSFEP	232
27	SFRITTDMQDRTRKEYALFYKLDVVPIND-------TSYRLVSCNTSVITQACPKVSFEP	233
28	SFRITTDMQDRTRKEYALFYKLDVVPIND--TPINDTSYRLVSCNTSVITQACPKVSFEP	228
29	SFMITTDMQDRTRKEYALFYKLDVVPIND-------TRYRLVSCNTSVITQACPKVSFEP	232
30	SFRITTDMQDRTRKEYALFYKLDVVPIND-------TSYRLVSCNTSVITQACPKVSFEP	230
31	SFRITTDMQDRTRKEYALFYKLDVVPIND-------TSYRLVSCNTSVITQACPKVSFEP	230
32	SFRITTDMQDRTRKEYALFYKLDVVPIND-------TSYRLVSCNTSVITQACPKVSFEP	230
33	SFKITTEMKDRTRKEYALFYKLDVVPIND-------TRYRLVSCNTSVLTQACPKVSFEP	206
34	SFRITTDMQDRTRKEYALFYKLDVVPIND-------TSYRLVSCNTSVITQACPKVSFEP	230
35	SFKITTDVKDRMRKEYALFYKLDVVPIND-------TRYRLISCNTSVITQACPKVSFEP	207
36	SFRITTDMQDRTRKEYALFYKLDVVPIND-------TRYRLVSCNTSVITQACPKVSFEP	232
37	SFRITTDMQDRTRKEYALFYKLDVVPIND-------TRYRLVSCNTSVITQACPKVSFEP	232
38	SFRITTDMQDRTRKEYALFYKLDVVPIND-------TRYRLVSCNTSVITQACPKVSFEP	232
39	SFKITTEIRDRTRKEYALFYKLDVVPIND-------TRYRLVSCNTSVITQACPKVSFEP	208
40	SFKITTDVKDRMRKEYALFYKLDVVPIND-------TRYRLISCNTSVITQACPKVSFEP	207
41	SFRITTDMQDRTRKEYALFYKLDVVPIND-------TRYRLVSCNTSVITQACPKVSFEP	232
42	SFKITTDVKDRMRKEYALFYKLDVVPIND-------TRYRLISCNTSVITQACPKVSFEP	207

	IPIHYCAPAGFAILKCNDKQFNGTGPCTNVSTVQCTHGIRPVVSTQLLLNGSLAEEEVVI	
1	IPIHYCAPAGFAILKCNDKQFIGTGPCTNVSTVQCTHGIRPVVSTQLLLNGSLAEEEVVI	268
2	IPIHYCAPAGFAILKCNDKQFNGTGPCTNVSTVQCTHGIRPVVSTQLLLNGSLAEEEVVI	290
3	IPIHYCAPAGFAILKCNDKQFNGTGPCTNVSAVQCTHGIRPVVSTQLLLNGSLAEEEVVI	267
4	IPIHYCAPAGFAILKCNDKQFNGTGPCTNVSTVQCTHGIRPVVSTQLLLNGSLAEEEVVI	267
5	IPIHYCAPAGFAILKCNDKQFNGTGPCTNVSTVQCTHGIRPVVSTQLLLNGSLAEEEVVI	269
6	IPIHYCAPAGFAILKCNDKQFNGTGPCTNVSTVQCTHGIRPVVSTQLLLNGSLAEEEVVI	292
7	IPIHYCAPAGFAILKCNDKQFNGTGPCTNVSTVQCTHGIRPVVSTQLLLNGSLAEEEVVI	290
8	IPIHYCAPAGFAILKCNDKQFNGTGPCTNVSTVQCTHGIRPVVSTQLLLNGSLAEEEVVI	267
9	IPIHYCAPAGFAILKCNDKQFNGTGPCTNVSTVQCTHGIRPVVSTQLLLNGSLAEEEVVI	290
10	IPIHYCAPAGFAILKCNDKQFNGTGPCTNVSTVQCTHGIRPVVSTQLLLNGSLAEEEVVI	267
11	IPIHYCAPAGFAILKCNDKQFNGTGPCTNVSTVQCTHGIRPVVSTQLLLNGSLAEEEVVI	269
12	IPIHYCAPAGFAILKCNDKQFNGTGPCTNVSTVQCTHGIRPVVSTQLLLNGSLAEEEVVI	292
13	IPIHYCAPAGFAILKCNDKQFNGTGPCTNVSTVQCTHGIRPVVSTQLLLNGSLAEEEVVI	292
14	IPIHYCAPAGFAILKCNDKQFNGTGPCTNVSTVQCTHGIRPVVSTQLLLNGSLAEEEVVI	292
15	IPIHYCAPAGFAILKCNDKQFNGTGPCTNVSTVQCTHGIRPVVSTQLLLNGSLAEEEVVI	292
16	IPIHYCAPAGFAILKCNDKQFNGTGPCTNVSTVQCTHGIRPVVSTQLLLNGSLAEEEVVI	268
17	IPIHYCAPAGFAILKCNDKQFNGTGPCTNVSTVQCTHGIRPVVSTQLLLNGSLAEEEVVI	267
18	IPIHYCAPAGFAILKCNDKQFNGTGPCTNVSTVQCTHGIRPVVSTQLLLNGSLAEEEVVI	290
19	IPIHYCAPAGFAILKCNDKQFNGTGPCTNVSTVQCTHGIRPVVSTQLLLNGSLAEEEVVI	264
20	IPIHYCAPAGFAILKCNDKQFNGTGPCTNVSTVQCTHGIRPVVSTQLLLNGSLAEEEVVI	290
21	IPIHYCAPAGFAILKCNDKQFNGTGPCTNVSTVQCTHGIRPVVSTQLLLNGSLAEEEVVI	264
22	IPIHYCAPAGFAILKCNDKQFNGTGPCTNVSTVQCTHGIRPVVSTQLLLNGSLAEEEVVI	267
23	IPIHYCAPAGFAILKCNDKQFNGTGPCTNVSTVQCTHGIRPVVSTQLLLNGSLAEEEVVI	297
24	IPIHYCAPAGFAILKCNDKQFNGTGPCTNVSTVQCTHGIRPVVSTQLLLNGSLAEEEVVI	267
25	IPIHYCAPAGFAILKCNDKQFNGTGPCTNVSTVQCTHGIRPVVSTQLLLNGSLAEEEVVI	290
26	IPIHYCAPAGFAILKCNDKQFNGTGPCTNVSTVQCTHGIRPVVSTQLLLNGSLAEEEVVI	292
27	IPIHYCAPAGFAILKCNDKQFNGTGPCTNVSTVQCTHGIRPVVSTQLLLNGSLAEEEVVI	293
28	IPIHYCAPAGFAILKCNDKQFNGTGPCTNVSTVQCTHGIRPVVSTQLLLNGSLAEEEVVI	288
29	IPIHYCAPAGFAILKCNDKQFNGTGPCTNVSTVQCTHGIRPVVSTQLLLNGSLAEEEVVI	292
30	IPIHYCAPAGFAILKCNDKQFNGTGPCTNVSTVQCTHGIRPVVSTQLLLNGSLAEEEVVL	290
31	IPIHYCAPAGFAILKCNDKQFNGTGPCTNVSTVQCTHGIRPVVSTQLLLNGSLAEEEVVI	290
32	IPIHYCAPAGFAILKCNDKQFNGTGPCTNVSTVQCTHGIRPVVSTQLLLNGSLAEEEVVI	290
33	IPIHYCAPAGFAILKCNDKQFNGTGPCTNVSTVQCTHGIRPVVSTQLLLNGSLAEEEVVI	266
34	IPIHYCAPAGFAILKCNDKQFNGTGPCTNVSTVQCTHGIRPVVSTQLLLNGSLAEEEVVI	290
35	IPIHYCAPAGFAILKCNDKQFNGTGPCTNVSTVQCTHGIRPVVSTQLLLNGSLAEEEVVI	267
36	IPIHYCAPAGFAILKCNDKQFNGTGPCTNVSTVQCTHGIRPVVSTQLLLNGSLAEEEVVI	292
37	IPIHYCAPAGFAILKCNDKQFNGTGPCTNVSTIQCTHGIRPVVSTQLLLNGSLAEEEVVI	292
38	IPIHYCAPAGFAILKCNDKQFNGTGPCTNVSTVQCTHGIRPVVSTQLLLNGSLAEEEVVI	292
39	IPIHYCAPAGFAILKCNDKQFNGTGPCTNVSTVQCTHGIRPVVSTQLLLNGSLAEEEVVI	268
40	IPIHYCAPAGFAILKCNDKQFNGTGPCTNVSTVQCTHGIRPVVSTQLLLNGSLAEEEVVI	267
41	IPIHYCAPAGFAILKCNDKQFNGTGPCTNVSTVQCTHGIRPVVSTQLLLNGSLAEEEVVI	292
42	IPIHYCAPAGFAILKCNDKQFNGTGPCTNVSTVQCTHGIRPVVSTQLLLNGSLAEEEVVI	267

	Sequence	
	RSVNFSDNAKTIIVQLNKSVEINCTRPNNNTRKSIHMGPGKAFYARGXIIGDIRKAHCNI	
1	RSVNFSDNAKTIIVQLNKSVEITCTRPNNNTRKSIPMGPGKAFYARGDITGDIRKAYCEI	328
2	RSVNFSDNAKTIIVQLNKSVEINCTRPNNNTRKSIHMGPGKAFYARGAIIGDIRKAHCNI	350
3	RSVNFSDNAKTIIVQLNKSVEMTCTRPNNNTRKSIPMGPGKVFYARGDIIGDIRKAHCNI	327
4	RSVNFSDNAKTIIVQLNKSVEMTCTRPNNNTRKSIPMGPGKVFYARGDIIGDIRKAHCNI	327
5	RSVNFSDNAKTIIVQLNKSVEINCTRPHNNTRKSIPMGPGKAFYARGNIIGDIRKAYCNI	329
6	RSVNFSDNAKTIIVQLNKSVEINCTRPNNNTRKSIHMGPGKAFYARGAIIGDIRKAHCNI	352
7	RSVNFSDNAKTIIVQLNKSVEINCTRPNNNTRKSIHMGPGKAFYARGGIIGDIRKAHCNI	350
8	RSVNFSDNAKTIIVQLNKSVEMTCTRPNNNTRKSIPMGPGKVFYARGDIIGDIRKAHCNI	327
9	RSVNFSDNAKTIIVQLNKSVEINCTRPNNNTRKSIHMGPGKAFYARGGIIGDIRKAHCNI	350
10	RSVNFSDNAKTIIVQLNKSVEMTCTRPNNNTRKSIPMGPGKVFYARGDIIGDIRKAHCNI	327
11	RSVNFSDNAKTIIVQLNKSVEINCTRPHNNTRKSIPMGPGKAFYARGNIIGDIRKAYCNI	329
12	RSVNFSDNTKTIIVQLNKSVEINCTRPNNNTRKSIHMGPGKAFYARGAIIGDIRKAHCNI	352
13	RSVNFSDNAKTIIVQLNKSVEINCTRPNNNTRKSIHMGPGKAFYARGAIIGDIRKAHCNI	352
14	RSVNFSDNAKTIIVQLNKSVEINCTRPNNNTRKSIHMGPGKAFYARGAIIGDIRKAHCNI	352
15	RSVNFSDNAKTIIVQLNKSVEINCTRPNNNTRKSIHMGPGKAFYARGAIIGDIRKAHCNI	352
16	RSVNFSDNAKTIIVQLNKSVEINCTRPHNNTRKSIPMGPGKAFYARGGIIGDIRKAYCNI	328
17	RSVNFSDNAKTIIVQLNKSVEMTCTRPNNNTRKSIPMGPGKVFYARGDIIGDIRKAHCNI	327
18	RSVNFSDNAKTIIVQLNKSVEINCTRPNNNTRKSIHMGPGKAFYARGGIIGDIRKAHCNI	350
19	RSVNFSDNAKTIIVQLNKSVEINCTRPNNNTRKSIPMGPGKAFYARGGIIGDIRKAYCNI	324
20	RSVNFSDNAKTIIVQLNKSVKINCTRPNNNTRKSIHMGPGKAFYARGGIIGDIRKAHCNI	350
21	RSVNFSDNAKTIIVQLNKSVEINCTRPHNNTRKSIPMGPGKAFYARGNIIGDIRKAYCNI	324
22	RSVNFSDNAKTIIVQLNKSVEMTCTRPNNNTRKSIPMGPGKVFYARGDIIGDIRKAHCNI	327
23	RSVNFSDNAKTIIVQLNKSVEINCTRPNNNTRKSIHMGPGKAFYARGGIIGDIRKAHCNI	357
24	RSVNFSDNAKTIIVQLNKSVEMTCTRPNNNTRKSIPMGPGKVFYARGDIIGDIRKAHCNI	327
25	RSVNFSDNAKTIIVQLNKSVEINCTRPNNNTRKSIHMGPGRAFYARGGIIGDIRKAHCNI	350
26	RSVNFSDNAKTIIVQLNKSVEINCTRPNNNTRKSIHMGPGKAFYARGAIIGDIRKAHCNI	352
27	RSVNFSDNAKTIIVQLNKSVEINCTRPNNNTRKSIHMGPGKAFYARGAIIGDIRKAHCNI	353
28	RSVNFSDNAKTIIVQLNKSVEINCTRPNNNTRKSIHMGPGKAFYARGAIIGDIRKAHCNI	348
29	RSVNFSDNAKTIIVQLNKSVEINCTRPNNNTRKSIHMGPGKAFYARGAIIGDIRKAHCNI	352
30	RSVNFSDNAKTIIVQLNKSVEINCTRPNNNTRKSIHMGPGKAFYARGAIIGDIRKAHCNI	350
31	RSVNFSDNAKTIIVQLNKSVEINCTRPNNNTRKSIHMGPGKAFYARGGIIGDIRKAHCNI	350
32	RSVNFSDNAKTIIVQLNKSVEINCTRPNNNTRKSIHMGPGKAFYARGGIIGDIRKAHCNI	350
33	RSVNFSDNAKTIIVQLNKSVEINCTRPHNNTRKSIPMGPGKAFYARGNIIGDIRKAYCNI	326
34	RSVNFSDNAKTIIVQLNKSVEINCTRPNNNTRKSIHMGPGKAFYARGGIIGDIRKAHCNI	350
35	RSVNFSDNAKTIIVQLNKSVEMTCTRPNNNTRKSIPMGPGKVFYARGDIIGDIRKAHCNI	327
36	RSVNFSDNAKTIIVQLNKSVEINCTRPNNNTRKSIHMGPGKAFYARGAIIGDIRKAHCNI	352
37	RSVNFSDNAKTIIVQLNKSVEINCTRPNNNTRKSIHMGPGKAFYARGAIIGDIRKAHCNI	352
38	RSVNFSDNAKTIIVQLNKSVEINCTRPNNNTRKSIHMGPGKAFYARGAIIGDIRKAHCNI	352
39	RSVNFSDNAKTIIVQLNKSVEINCTRPHNNTRKSIPMGPGKAFYARGGIIGDIRKAYCNI	328
40	RSVNFSDNAKTIIVQLNKSVEMTCTRPNNNTRKSIPMGPGKVFYARGDIIGDIRKAHCNI	327
41	RSVNFSDNAKTIIVQLNKSVEINCTRPNNNTRKSIHMGPGKAFYARGAIIGDIRKAHCNI	352
42	RSVNFSDNAKTIIVQLNKSVEMTCTRPNNNTRKSIPMGPGKVFYARGDIIGDIRKAHCNI	327

	Sequence	
	SGTEWHSTLKLVVEKLREQY-NKTIVFNHSSGGDPEIVMYSFNCGGEFFYCNSTKLFNST	
1	NGTEWHSTLKLVVEKLREQY-NKTIVFNRSSGGDPEIVMYSFNCGGEFFYCNSTKLFNST	387
2	SRAEWYSTLKLVAEKLREQY-HKTIVFNHSSGGDPEIVMYSFNCGGEFFYCNSTKLFNST	409
3	SGTEWHSTLKLVVGKLREQY-NKTIVFNHSSGGDPEIVMYSFNCGGEFFYCNSTKLFNST	386
4	SGTEWHSTLKLVVGKLREQY-NKTIVFNHSSGGDPEIVMYSFNCGGEFFYCNSTKLFNST	386
5	SRPEWHSTLKLVVEKLRKQY-NKTIVFNHSSGGDPEIVMYSFNCGGEFFYCNSTKLFNST	388
6	SGTEWHNTLKLVVEKLKEQY-NKTIVFNHSSGGDPEIVMYSFNCGGEFFYCNSTKLFNST	411
7	SGTEWHNTLKLVVEKLREQY-NKTIVFNHSSGGDPEIVMYSFNCGGEFFYCNSTKLFNST	409
8	SGTEWHSTLKLVVGKLREQY-NKTIVFNHSSGGDPEIVMYSFNCGGEFFYCNSTKLFNST	386
9	SRTEWHSTLKLVAEKLREQY-KKTIVFNHSSGGDPEIVMYSFNCGGEFFYCNSTKLFNST	409
10	SGTEWHSTLKLVVGKLREQY-NKTIVFNHSSGGDPEIVMYSFNCGGEFFYCNSTKLFNST	386
11	SRPEWHSTLKLVVEKLRKQY-NKTIVFNHSSGGDPEIVMYSFNCGGEFFYCNSTKLFNST	388
12	SGTEWHNTLKLVVEKLREQY-NKTIVFNHSSGGDPEIVMYSFNCGGEFFYCNSTKLFNST	411
13	SGTEWHNTLKLVVEKLREQY-NKTIVFNHSSGGDPEIVMYSFNCGGEFFYCNSTKLFNST	411
14	SGTEWHNTLTLVVEKLREQF-NKTIVFNHSSGGDPEIVMYSFNCGGEFFYCNSTKLFNST	411
15	SGTEWHNTLKLVVEKLREQY-NKTIVFNHSSGGDPEIVMYSFNCGGEFFYCNSTKLFNST	411
16	SRAEWHSTLKLVVEKLREQY-NKTIVFNHSSGGDPEIVMYSFNCGGEFFYCNSTKLFNST	387
17	SGTEWHSTLKLVVGKLREQY-NKTIVFNHSSGGDPEIVMYSFNCGGEFFYCNSTKLFNST	386
18	SGTEWHNTLKLVVEKLREQY-NKTIVFNHSSGGDPEIVMYSFNCGGEFFYCNSTKLFNST	409
19	SRAEWHSTLKLVVEKLREQY-NKTIVFNHSSGGDPEIVMYSFNCGGEFFYCNSTKLFNST	383
20	SGTEWHSTLKLVVKKLREQY-NKTIVFNHSSGGDPEIVMYSFNCGGEFFYCNSTKLFNST	409
21	SGPEWHSTLKLVVEKLRAQY-NKTIVFNHSSGGDPEIVMYSFNCGGEFFYCNSTKLFNST	383
22	SGTEWHSTLKLVVGKLREQY-NKTIVFNHSSGGDPEIVMYSFNCGGEFFYCNSTKLFNST	386
23	SGTEWHSTLKLVVEKLREKY-NKTIVFNHSSGGDPEIVMYSFNCGGEFFYCNSTKLFNST	416
24	SGTEWHSTLKLVVGKLREQY-NKTIVFNHSSGGDPEIVMYSFNCGGEFFYCNSTKLFNST	386
25	SGTEWHSTLKLVVKKLREQY-NKTIVFNHSSGGDPEIVMYSFNCGGEFFYCNSTKLFNST	409
26	SGTEWHNTLKLVVEKLREQY-NKTIVFNHSSGGDPEIVMYSFNCGGEFFYCNSTKLFNST	411
27	SGTEWHNTLKLVVEKLREQY-NKTIVFNHSSGGDPEIVMYSFNCGGEFFYCNSTKLFNST	412
28	SGTEWHNTLKLVVKKLREQY-NKTIVFNHSSGGDPEIVMYSFNCGGEFFYCNSTKLFNST	407
29	SGTEWHNTLKLVVKKLREQY-NKTIVFNHSSGGDPEIVMYSFNCGGEFFYCNSTKLFNST	411
30	SGTEWHNTLKLVVKKLREQY-NKTIVFNHSSGGDPEIVMYSFNCGGEFFYCNSTKLFNST	409
31	SGTEWHNTLKLVVEKLREQY-NKTIVFNHSSGGDPEIVMYSFNCGGEFFYCNSTKLFNST	409
32	SGTEWHNTLKLVVEKLREQY-NKTIVFNHSSGGDPEIVMYSFNCGGEFFYCNSTKLFNST	409
33	SGPDWHSTLKLVVEKLREQY-NKTIVFNHSSGGDPEIVMYSFNCGGEFFYCNSTKLFNST	385
34	SGTEWHNTLKLVVKKLREQY-NKTIVFNHSSGGDPEIVMYSFNCGGEFFYCNSTKLFNST	409
35	SGTEWHSTLKLVVGKLREQY-NKTIVFNHSSGGDPEIVMYSFNCGGEFFYCNSTKLFNST	386
36	SGTEWHNTLKLVVEKLREQY-NKTIVFNHSSGGDPEIVMYSFNCGGEFFYCNSTKLFNST	411
37	SGTEWHNTLKLVVEKLREQY-NKTIVFNHSSGGDPEIVMYSFNCGGEFFYCNSTKLFNST	411
38	SGTEWHNTLKLVVEKLREQY-NKTIVFNHSSGGDPEIVMYSFNCGGEFFYCNSTKLFNST	411
39	SRAEWHSTLKLVVEKLREQY-NKTIVFNHSSGGDPEIVMYSFNCGGEFFYCNSTKLFNST	387
40	SGTEWHSTLKLVVGKLREQY-NKTIVFNHSSGGDPEIVMYSFNCGGEFFYCNSTKLFNST	386
41	SGTEWHNTLKLVVKKLREQY-NKTIVFNHSSGGDPEIVMYSFNCGGEFFYCNSTKLFNST	411
42	SGTEWHSTLKLVVGKLREQY-NKTIVFNHSSGGDPEIVMYSFNCGGEFFYCNSTKLFNST	386

```
     WPXNX--TXGSHDTNGTLTLPCRIKQIINMWQEVGKAMYAPPIEGIIKCSSNITGLLLTR
1    WPWND--TKGSHDTNGTLILPCKIKQIINMWQGVGKAMYAPPIEGKIRCSSNITGLLLTR  445
2    WPGND--TEGSHDTNGTLTLPCRIKQIINMWQEVGKAMYAPPIEGIIKCSSNITGLLLTR  467
3    WPWNG--TNGTHDTNGTLTLPCRIKQIINMWQEVGKAMYAPPIEGIIKCSSNITGLLLTR  444
4    WPWNG--TNGSHDTNGTLTLPCRIKQIINMWQEVGKAMYAPPIEGIIKCSSNITGLLLTR  444
5    WPWND--TNRSHDTNDTLMLPCKIKQII-TWQGVGKAMYAPPIEGEIRCSSNITGLLLTR  445
6    WPRNA--TKGSPDTNGTLTLPCRIKQIINMWQEVGKAMYAPPIEGIIKCSSNITGLLLTR  469
7    WHGND--TEGSHNTNGTLTLPCRIKQIINMWQEVGKAMYAPPIEGIIKCSSNITGLLLTR  467
8    WPWNG--TNGSHDTNGTLTLPCRIKQIINMWQEVGKAMYAPPIEGIIKCSSNITGLLLTR  444
9    WPGSD--TEGSHNTNGTLTLPCRIKQIINMWQEVGKAMYAPPIEGIIKCSSNITGLLLTR  467
10   WPWNG--TNGSHDTNGTLTLPCRIKQIINMWQEVGKAMYAPPIEGIIKCSSNITGLLLTR  444
11   WPWND--TNRSHDTNDTLMLPCKIKQIINMWQGVGKAMYAPPIEGEIRCSSNITGLLLTR  446
12   WPRNA--IRGSPDTNGTLTLPCRIKQIINMWQEVGKAMYAPPIEGIIKCSSNITGLLLTR  469
13   WHGND--TEGSHNTNGTLTLPCRIKQIINMWQEVGKAMYAPPIEGIIKCSSNITGLLLTR  469
14   WHRNA--TRGSPDTNGTLTLPCRIKQIINMWQEVGKAMYAPPIEGIIKCSSNITGLLLTR  469
15   WPGNG--TNGSPDTNGTLTLPCRIKQIINMWQEVGKAMYAPPIEGIIKCSSNITGLLLTR  469
16   WPRND--TQGSHDTNDTLMLPCKIKQIINMWQGVGKAMYAPPIEGEIRCSSNITGLLLTR  445
17   WPWNG--TNGSHDTNGTLTLPCRIKQIINMWQEVGKAMYAPPIKGIIKCSSNITGLLLTR  444
18   WLGND--TEGSHNTNGTLTLPCRIKQIINMWQGVGKAMYAPPIEGIIKCSSNITGLLLTR  467
19   WPRNA--TQGSHDTNDTLMLPCKIKQIINMWQGVGKAMYAPPIEGEIRCSSNITGLLLTR  441
20   WPGND--TEGSHNTNGTLTLPCRIKQIINMWQEVGKAMYAPPIEGIIKCSSNITGLLLTR  467
21   WPWND--TNRSHDTNDTLMLPCKIKQIINMWQGVGKAMYAPPIEGEIRCSSNITGLLLTR  441
22   WPWNG--TNGSHDTNGTLTLPCRIKQIINMWQEVGKAMYAPPIEGIIKCSSNITGLLLTR  444
23   WPRNA--TKGSPDTNGTLTLPCRIKQIINMWQEVGKAMYAPPIEGIIKCSSNITGLLLTR  474
24   WPWNG--TNGSHDTNGTLTLPCRIKQIINMWQEVGKAMYAPPIEGIIKCSSNITGLLLTR  444
25   WPWNDNDTEGSHDTNGTLTLPCRIKQIINMWQGVGKAMYAPPIEGIIKCSSNITGLLLTR  469
26   WHGND--TEGSHNTNGTLTLPCRIKQIINMWQEVGKAMYAPPIEGIIKCSSNITGLLLTR  469
27   WHGND--TEGSHNTNGTLTLPCRIKQIINMWQEVGKAMYAPPIEGIIKCSSNITGLLLTR  470
28   WPWNG--TQGSHDTNGTLTLPCRIKQIINMWQGVGKAMYAPPIEGIIKCSSNITGLLLTR  465
29   WPWNND-TEGSHDTNGTLTLPCRIKQIINMWQEVGKAMYAPPIEGIIKCSSNITGLLLTR  470
30   WPGND--TNGSHDTNGTLTLPCRIKQIINMWQGVGKAMYAPPIEGIIKCSSNITGLLLTR  467
31   WPRNT--TRGSPDTNGTLTLPCRIKQIINMWQEVGKAMYAPPIEGIIKCSSNITGLLLTR  467
32   WHGND--TEGSHNTNGTLTLPCRIKQIINMWQEVGKAMYAPPIEGIIKCSSNITGLLLTR  467
33   WPRND--TKRSHDTNDTLMLPCKIKQIINMWQGVGKAMYAPPIEGEIRCSSNITGLLLTR  443
34   WPGND--TEGSHNTNGTLTLPCRIKQIINMWQGVGKAMYAPPIEGIIKCSSNITGLLLTR  467
35   WPWNG--TNGSHDTNGTLTLPCRIKQIINMWQEVGKAMYAPPIEGIIKCSSNITGLLLTR  444
36   WTRNA--TRGSPDTNGTLTLPCRIKQIINMWQEVGKAMYAPPIEGIIKCSSNITGLLLTR  469
37   WPRNT--TRGSPDTNGTLTLPCRIKQIINMWQEVGKAMYAPPIEGIIKCSSNITGLLLTR  469
38   WPRNT--TRGSPDTNGTLTLPCRIKQIINMWQEVGKAMYAPPIEGIIKCSSNITGLLLTR  469
39   WSWNG--TKGSHDTNDTLMLPCKIKQIINMWQGVGKAMYAPPIEGEIRCSSNITGLLLTR  445
40   WPWNG--TNGSHDTNGTLTLPCRIKQIINMWQEVGKAMYAPPIEGIIKCSSNITGLLLTR  444
41   WPWNG--TNGSHDTNGTLTLPCRIKQIINMWQGVGKAMYAPPIEGIIKCSSNITGLLLTR  469
42   WPWNG--TNGSHDTNGTLTLPCRIKQIINMWQEVGKAMYAPPIEGIIKCSSNITGLLLTR  444
```

	DGG-YESNKTDEIFRPGGGDMRDNWRSELYKYKVVKIEPLGVAPTKAKRRVVQREKRAFG	
1	DGG-YESNETDEIFRPGGGDMRDNWRSELYKYKVVKIEPLGVAPTKAKRRVVQREKRAFG	504
2	DGG-YESNKTDEIFRPGGGDMRDNWRSELYKYKVVKIEPLGVAPTKAKRRVVQREKRAFG	526
3	DGG-YESNETDEIFRPGGGDMRDNWRSELYKYKVVKIEPLGVAPTKAKRRVVQREKRAFG	503
4	DGG-YESNETDEIFRPGGGDMRDNWRSELYKYKVVKIEPLGVAPTKAKRRVVQREKRAFG	503
5	DGG-YESNETDEIFRPGGGDMRDNWRSELYKYKVVKIEPLGVAPTRAKRRVVQREKRAFG	504
6	DGG-YESNKTDEIFRPGGGDMRDNWRSELYKYKVVKIEPLGVAPTKAKRRVVQREKRAFG	528
7	DGG-YESNKTDEIFRPGGGDMRDNWRSELYKYKVVKIEPLGVAPTKAKRRVVQREKRAFG	526
8	DGG-YESNETDEIFRPGGGDMRDNWRSELYKYKVVKIEPLGVAPTKAKRRVVQREKRAFG	503
9	DGG-YESNKTDEIFRPGGGDMRDNWRSELYKYKVVKIEPLGVAPTKAKRRVVQREKRAFG	526
10	DGG-YESNETDEIFRPGGGDMRDNWRSELYKYKVVKIEPLGVAPTKAKRRVVQREKRAFG	503
11	DGG-YESNETDEIFRPGGGDMRDNWRSELYKYKVVKIEPLGVAPTKAKRRVVQREKRAFG	505
12	DGG-YESNKTDEIFRPGGGDMRDNWRSELYKYKVVKIEPLGVAPTKAKRRVVQREKRAFG	528
13	DGG-YESNKTDEIFRPGGGDMRDNWRSELYKYKVVKIEPLGVAPTKAKRRVVQREKRAFG	528
14	DGG-YESNKTDEIFRPGGGDMRDNWRSELYKYKVVKIEPLGVAPTKAKRRVVQREKRAFG	528
15	DGG-YESNKTDEIFRPGGGDMRDNWRSELYKYKVVKIEPLGVAPTKAKRRVVQREKRAFG	528
16	DGG-YESNETDEIFRPGGGDMRDNWRSELYKYKVVKIEPLGVAPTKAKRRVVQREKRAFG	504
17	DGG-YESNETDEIFRPGGGDMRDNWRSELYKYKVVKIEPLGVAPTKAKRRVVQREKRAFG	503
18	DGG-YESNKTDEIFRPGGGDMRDNWRSELYKYKVVKIEPLGVAPTKAKRRVVQREKRAFG	526
19	DGG-YESNETDEIFRPGGGDMRDNWRSELYKYKVVKIEPLGVAPTKAKRRVVQREKRAFG	500
20	DGG-YGSNKTDEIFRPGGGDMRDNWRSELYKYKVVKIEPLGVAPTKAKRRVVQREKRAFG	526
21	DGG-YESNETDEIFRPGGGDMRDNWRSELYKYKVVKIEPLGVAPTKAKRRVVQREKRAFG	500
22	DGG-YESNETDEIFRPGGGDMRDNWRSELYKYKVVKIEPLGVAPTKAKRRVVQREKRAFG	503
23	DGG-YESNKTDEIFRPGGGDMRDNWRSELYKYKVVKIEPLGVAPTKAKRRVVQREKRAFG	533
24	DGG-YESNETDEIFRPGGGDMRDNWRSELYKYKVVKIEPLGVAPTKAKRRVVQREKRAFG	503
25	DGG-YESNKTDEIFRPGGGDMRDNWRSELYKYKVVKIEPLGVAPTKAKRRVVQREKRAFG	528
26	DGG-YESNKTDEIFRPGGGDMRDNWRSELYKYKVVKIEPLGVAPTKAKRRVVQREKRAFG	528
27	DGG-YESNKTDEIFRPGGGDMRDNWRSELYKYKVVKIEPLGVAPTKAKRRVVQREKRAFG	529
28	DGG-YESNKTDEIFRPGGGDMRDNWRSELYKYKVVKIEPLGVAPTKAKRRVVQREKRAFG	524
29	DGG-YESNKTDEIFRPGGGDMRDNWRSELYKYKVVKIEPLGVAPTKAKRRVVQREKRAFG	529
30	DGG-YESNKTDETFRPGGGDMRDNWRSELYKYKVVKIEPLGVAPTKAKRRVVQREKRAFG	526
31	DGG-YESNKTDEVFRPGGGDMRDNWRSELYKYKVVKIEPLGVAPTKAKRRVVQREKRAFG	526
32	DGG-YESNKTDEIFRPGGGDMRDNWRSELYKYKVVKIEPLGVAPTKAKRRVVQREKRAFG	526
33	DGG-YESNETDEIFRPGGGDMRDNWRSELYKYKVVKIEPLGVAPTKAKRRVVQREKRAFG	502
34	DGG-YESNKTDEIFRPGGGDMRDNWRSELYKYKVVKIEPLGVAPTKAKRRVVQREKRAFG	526
35	DGG-YESNETDEIFRPGGGDMRDNWRSELYKYKVVKIEPLGVAPTKAKRRVVQREKRAFG	503
36	DGG-YESNKTDEIFRPGGGDMRDNWRSELYKYKVVKIEPLGVAPTKAKRRVVQREKRAFG	528
37	DGG-YESNKTDEIFRPGGGDMRDNWRSELYKYKVVKIEPLGVAPTKAKRRVVQREKRAFG	528
38	DGG-YESNKTDEIFRPGGGDMRDNWRSELYKYKVVKIEPLGVAPTKAKRRVVQREKRAFG	528
39	DGG-YESNETDEIFRPGGGDMRDNWRSELYKYKVVKIEPLGVAPTKAKRRVVQREKRAFG	504
40	DGG-YESNETDEIFRPGGGDMRDNWRSELYKYKVVKIE-LGVAPTKAKRRVVQREKRAFG	502
41	DGG-YESNKTDEIFRPGGGDMRDNWRSELYKYKVVKIEPLGVAPTKAKRRVVQREKRAFG	528
42	DGG-YESNETDEIFRPGGGDMRDNWRSELYKYKVVKIEPLGVAPTKAKRRVVQREKRAFG	503

	**-LGAVFLGFLGAAGSTMGAASITLTVQARQLLSGIVQQQNNLLRAIEAQQHLLQLTVWGI**	
1	-LGAVFLGFLGAAGSTMGAASITLTVQARQLLSGIVQQQNNLLRAIEAQQHLLQLTVWGI	563
2	-LGAVFLGFLGAAGSTMGAASITLTVQARQLLSGIVQQQNNLLRAIEAQQHLLQLTVWGI	585
3	-LGAVFLGFLGAAGSTMGAASITLTVQARQLLSGIVQQQNNLLRAIEAQQHLLQLTVWGI	562
4	-LGAVFLGFLGAAGSTMGAASITLTVQARQLLSGIVQQQNNLLRAIEAQQHLLQLTVWGI	562
5	-LGAVFLGFLGAAGSTMGAASITLTVQARQLLSGIVQQQNNLLRAIEAQQHLLQLTVWGI	563
6	-LGAVFLGFLGAAGSTMGAASITLTVQARQLLSGIVQQQNNLLRAIEAQQHLLQLTVWGI	587
7	-LGAVFLGFLGAAGSTMGAASITLTVQARQLLSGIVQQQNNLLRAIEAQQHLLQLTVWGI	585
8	-LGAVFLGFLGAAGSTMGAASITLTVQARQLLSGIVQQQNNLLRAIEAQQHLLQLTVWGI	562
9	-LGAVFLGFLGAAGSTMGAASITLTVQARQLLSGIVQQQNNLLRAIEAQQHLLQLTVWGI	585
10	-LGAVFLGFLGAAGSTMGAASITLTVQARQLLSGIVQQQNNLLRAIEAQQHLLQLTVWGI	562
11	-LGAVFLGFLGAAGSTMGAASITLTVQARQLLSGIVQQQNNLLRAIEAQQHLLQLTVWGI	564
12	-LGAVFLGFLGAAGSTMGAASITLTVQARQLLSGIVQQQNNLLRAIEAQQHLLQLTVWGI	587
13	-LGAVFLGFLGAAGSTMGAASITLTVQARQLLSGIVQQQNNLLRAIEAQQHLLQLTVWGI	587
14	-LGAVFLGFLGAAGSTMGAASITLTVQARQLLSGIVQQQNNLLRAIEAQQHLLQLTVWGI	587
15	-LGAVFLGFLGAAGSTMGAASITLTVQARQLLSGIVQQQNNLLRAIEAQQHLLQLTVWGI	587
16	-LGAVFLGFLGAAGSTMGAASITLTVQARQLLSGIVQQQNNLLRAIEAQQHLLQLTVWGI	563
17	-LGAVFLGFLGAAGSTMGAASITLTVQARQLLSGIVQQQNNLLRAIEAQQHLLQLTVWGI	562
18	-LGAVFLGFLGAAGSTMGAASITLTVQARQLLSGIVQQQNNLLRAIEAQQHLLQLTVWGI	585
19	-LGAVFLGFLGAAGSTMGAASITLTVQARQLLSGIVQQQNNLLRAIEAQQHLLQLTVWGI	559
20	-LGAVFLGFLGAAGSTMGAASITLTVQARQLLSGIVQQQNNLLRAIEAQQHLLQLTVWGI	585
21	-LGAVFLGFLGAAGSTMGAASITLTVQARQLLSGIVQQQNNLLRAIEAQQHLLQLTVWGI	559
22	-LGAVFLGFLGAAGSTMGAASITLTVQARQLLSGIVQQQNNLLRAIEAQQHLLQLTVWGI	562
23	-LGAVFLGFLGAAGSTMGAASITLTVQARQLLSGIVQQQNNLLRAIEAQQHLLQLTVWGI	592
24	-LGAVFLGFLGAAGSTMGAASITLTVQARQLLSGIVQQQNNLLRAIEAQQHLLQLTVWGI	562
25	-LGAVFLGFLGAAGSTMGAASITLTVQARQLLSGIVQQQNNLLRAIEAQQHLLQLTVWGI	587
26	-LGAVFLGFLGAAGSTMGAASITLTVQARQLLSGIVQQQNNLLRAIEAQQHLLQLTVWGI	587
27	-LGAVFLGFLGAAGSTMGAASITLTVQARQLLSGIVQQQNNLLRAIEAQQHLLQLTVWGI	588
28	-LGAVFLGFLGAAGSTMGAASITLTVQARQLLSGIVQQQNNLLRAIEAQQHLLQLTVWGI	583
29	-LGAVFLGFLGAAGSTMGAASITLTVQARQLLSGIVQQQNNLLRAIEAQQHLLQLTVWGI	588
30	-LGAVFLGFLGAAGSTMGAASITLTVQARQLLSGIVQQQNNLLRAIEAQQHLLQLTVWGI	585
31	-LGAVFLGFLGAAGSTMGAASITLTVQARQLLSGIVQQQNNLLRAIEAQQHLLQLTVWGI	585
32	-LGAVFLGFLGAAGSTMGAASITLTVQARQLLSGIVQQQNNLLRAIEAQQHLLQLTVWGI	585
33	-LGAVFLGFLGAAGSTMGAASITLTVQARQLLSGIVQQQNNLLRAIEAQQHLLQLTAWGI	561
34	-LGAVFLGFLGAAGSTMGAASITLTVQARQLLSGIVQQQNNLLRAIEAQQHLLQLTVWGI	585
35	-LGAVFLGFLGAAGSTMGAASITLTVQARQLLSGIVQQQNNLLRAIEAQQHLLQLTVWGI	562
36	-LGAVFLGFLGAAGSTMGAASITLTVQARQLLSGIVQQQNNLLRAIEAQQHLLQLTVWGI	587
37	-LGAVFLGFLGAAGSTMGAASITLTVQARQLLSGIVQQQNNLLRAIEAQQHLLQLTVWGI	587
38	-LGAVFLGFLGAAGSTMGAASITLTVQARQLLSGIVQQQNNLLRAIEAQQHLLQLTVWGI	587
39	-LGAVFLGFLGAAGSTMGAASITLTVQARQLLSGIVQQQNNLLRAIEAQQHLLQLTVWGI	563
40	-LGAVFLGFLGAAGSTMGAASITLTVQARQLLSGIVQQQNNLLRAIEAQQHLLQLTVWGI	561
41	-LGAVFLGFLGAAGSTMGAASITLTVQARQLLSGIVQQQNNLLRAIEAQQHLLQLTVWGI	587
42	-LGAVFLGFLGAAGSTMGAASITLTVQARQLLSGIVQQQNNLLRAIEAQQHLLQLTVWGI	562

	KQLQARVLAVERYLKDQQLLGIWGCSGKLICTTTVPWNTSWSNKSLEQIWDNMTWMEWER	
1	KQLQARVLAVERYLKDQQLLGIWGCSGKLICTTTVPWNTSWSNKSLEQIWDNMTWMEWER	623
2	KQLQARVLAVERYLKDQQLLGIWGCSGKLICTTTVPWNTSWSNKSLEQIWDNMTWMEWER	645
3	KQLQARVLAVERYLKDQQLLGIWGCSGKLICTTTVPWNTSWSNKSLEQIWDNMTWMEWER	622
4	KQLQARVLAVERYLKDQQLLGIWGCSGKLICTTTVPWNTSWSNKSLEQIWDNMTWMEWER	622
5	KQLQARVLAVERYLKDQQLLGIWGCSGKLICTTTVPWNTSWSNKSLEQIWDNMTWMEWER	623
6	KQLQARVLAVERYLKDQQLLGIWGCSGKLICTTTVPWNTSWSNKSLEQIWDNMTWMEWER	647
7	KQLQARVLAVERYLKDQQLLGIWGCSGKLICTTTVPWNTSWSNKSLEQIWDNMTWMEWER	645
8	KQLQARVLAVERYLKDQQLLGIWGCSGKLICTTTVPWNTSWSNKSLEQIWDNMTWMEWER	622
9	KQLQARVLAVERYLKDQQLLGIWGCSGKLICTTTVPWNTSWSNKSLEQIWDNMTWMEWER	645
10	KQLQARVLAVERYLKDQQLLGIWGCSGKLICTTTVPWNTSWSNKSLEQIWDNMTWMEWER	622
11	KQLQARVLAVERYLKDQQLLGIWGCSGKLICTTTVPWNTSWSNKSLEQIWDNMTWMEWER	624
12	KQLQARVLAVERYLKDQQLLGIWGCSGKLICTTTVPWNTSWSNKSLEQIWDNMTWMEWER	647
13	KQLQARVLAVERYLKDQQLLGIWGCSGKLICTTTVPWNTSWSNKSLEQIWDNMTWMEWER	647
14	KQLQARVLAVERYLKDQQLLGIWGCSGKLICTTTVPWNTSWSNKSLEQIWDNMTWMEWER	647
15	KQLQARVLAVERYLKDQQLLGIWGCSGKLICTTTVPWNTSWSNKSLEQIWDNMTWMEWER	647
16	KQLQARVLAVERYLKDQQLLGIWGCSGKLICTTTVPWNTSWSNKSLEQIWDNMTWMEWER	623
17	KQLQARVLAVERYLKDQQLLGIWGCSGKLICTTTVPWNTSWSNKSLEQIWDNMTWMEWER	622
18	KQLQARVLAVERYLKDQQLLGIWGCSGKLICTTTVPWNTSWSNKSLEQIWDNMTWMEWER	645
19	KQLQARVLAVERYLKDQQLLGIWGCSGKLICTTTVPWNTSWSNKSLEQIWDNITWMEWER	619
20	KQLQARVLAVERYLKDQQLLGIWGCSGKLICTTTVPWNTSWSNKSLEQIWDNMTWMEWER	645
21	KQLQARVLAVERYLKDQQLLGIWGCSGKLICTTTVPWNTSWSNKSLEQIWDNMTWMEWER	619
22	KQLQARVLAVERYLKDQQLLGIWGCSGKLICTTTVPWNTSWSNKSLEQIWDNMTWMEWER	622
23	KQLQARVLAVERYLKDQQLLGIWGCSGKLICTTTVPWNTSWSNKSLEQIWDNMTWMEWER	652
24	KQLQARVLAVERYLKDQQLLGIWGCSGKLICTTTVPWNTSWSNKSLEQIWDNMTWMEWER	622
25	KQLQARVLAVERYLKDQQLLGIWGCSGKLICTTTVPWNTSWSNKSLEQIWDNMTWMEWER	647
26	KQLQARVLAVERYLKDQQLLGIWGCSGKLICTTTVPWNTSWSNKSLEQIWDNMTWMEWER	647
27	KQLQARVLAVERYLKDQQLLGIWGCSGKLICTTTVPWNTSWSNKSLEQIWNNMTWMEWER	648
28	KQLQARVLAVERYLKDQQLLGIWGCSGKLICTTTVPWNTSWSNKSLEQIWDNMTWMEWER	643
29	KQLQARVLAVERYLKDQQLLGIWGCSGKLICTTTVPWNTSWSNKSLEQIWDNMTWMEWER	648
30	KQLQARVLAVERYLKDQQLLGIWGCSGKLICTTTVPWNTSWSNKSLEQIWDNMTWMEWER	645
31	KQLQARVLAVERYLKDQQLLGIWGCSGKLICTTTVPWNTSWSNKSLEQIWDNMTWMEWER	645
32	KQLQARVLAVERYLKDQQLLGIWGCSGKLICTTTVPWNTSWSNKSLEQIWDNMTWMEWER	645
33	KQLQARVLAVERYLKDQQLLGIWGCSGKLICTTTVPWNTSWSNKSLEQIWDNMTWMEWER	621
34	KQLQARVLAVERYLKDQQLLGIWGCSGKLICTTTVPWNTSWSNKSLEQIWDNMTWMEWER	645
35	KQLQARVLAVERYLKDQQLLGIWGCSGKLICTTTVPWNTSWSNKSLEQIWDNMTWMEWER	622
36	KQLQARVLAVERYLKDQQLLGIWGCSGKLICTTTVPWNTSWSNKSLEQIWDNMTWMEWER	647
37	KQLQARVLAVERYLKDQQLLGIWGCSGKLICTTTVPWNTSWSNKSLEQIWDNMTWMEWER	647
38	KQLQARVLAVERYLKDQQLLGIWGCSGKLICTTTVPWNTSWSNKSLEQIWDNMTWMEWER	647
39	KQLQARVLAVERYLKDQQLLGIWGCSGKLICTTTVPWNTSWSNKSLEQIWDNMTWMEWER	623
40	KQLQARVLAVERYLKDQQLLGIWGCSGKLICTTTVPWNTSWSNKSLEQIWDNMTWMEWER	621
41	KQLQARVLAVERYLKDQQLLGIWGCSGKLICTTTVPWNTSWSNKSLEQIWDNMTWMEWER	647
42	KQLQARVLAVERYLKDQQLLGIWGCSGKLICTTTVPWNTSWSNKSLEQIWDNMTWMEWER	622

```
    EINNYTGYIYQLIEESQNQQEKNEQELLALDKWASLWNWFDITNWLWYIKIFIMIVGGLI

1   EIDNYTGYIYQLIEESQNQQEKNEQELLALDKWASLWNWFDITNWLWYIKIFIMIVGGLI  683
2   EINNYTGYIYQLIEESQNQQEKNEQELLAWDKWASLWNWFDITNWLWYIKIVIMIVGGLI  705
3   EIDNYTGYIYQLIEESQNQQEKNEQELLALDKWASLWNWFDITNWLWYIKIFIMIVGGLI  682
4   EISNYTGYIYQLIEESQNQQEKNEQELLALDKWASLWNWFDITNWLWYIKIFIMIVGGLI  682
5   EINNYTGYIYQLIEESQNQQEKNEQELLALDKWASLWNWFDITNWLWYIKIFIMIVGGLI  683
6   EINNYTGYIYQLIEESQNQQEKNEQELLAWDKWASLWNWFDITNWLWYIKIFIMIVGGLI  707
7   EINNYTGYIYQLIEESQNQQEKNEQELLAWDKWASLWNWFDITNWLWYIKIFIMIVGGLI  705
8   EISNYTGYIYQLIEESQNQQEKNEQELLALDKWASLWNWFDITNWLWYIKIFIMIVGGLI  682
9   EINNYTGYIYQLIEESQNQQEKNEQELLALDKWASLWNWFDITNWLWYIKIFIMIVGGLI  705
10  EISNYTGYIYQLIEESQNQQEKNEQELLALDKWASLWNWFDITNWLWYIKIFIMIVGGLI  682
11  EINNYTGYIYQLIEESQNQQEKNEQELLALDKWASLWNWFDITNWLWYIKIFIMIVGGLI  684
12  EINNYTGYIYQLIEESQNQQEKNEQELLAWDKWASLWNWFDITNWLWYIKIFIMIVGGLI  707
13  EINNYTGYIYQLIEESQNQQEKNEQELLAWDKWASLWNWFDITNWLWYIKIFIMIVGGLI  707
14  EINNYTGYIYQLIEESQNQQEKNEQELLALDKWASLWNWFDITNWLWYIKIFIMIVGGLI  707
15  EINNYTGYIYQLIEESQNQQEKNEQELLAWDKWASLWNWFDITNWLWYIKIFIMIVGGLI  707
16  EINNYTGYIYQLIEESQNQQEKNEQELLALDKWASLWNWFDITNWLWYIKIFIMIVGGLI  683
17  EISNYTGYIYQLIEESQNQQEKNEQELLALDKWASLWNWFDITNWLWYIKIFIMIVGGLI  682
18  EINNYTGYIYQLIEESQNQQE-----LLAWDKWASLWNWFDITNWLWYIKIFIMIVGGLI  700
19  EINNYTGYIYQLIEESQNQQEKNEQELLAWDKWASLWNWFDITNWLWYIKIFIMIVGGLI  679
20  EINNYTGYIYQLIEESQNQQEKNEQELLAWDKWASLWNWFDITNWLWYIKIFIMIVGGLI  705
21  EIDNYTGYIYQLIEESQNQQEKNEQELLALDKWASLWNWFDITNWLWYIKIFIMIVGGLI  679
22  EISNYTGYIYQLIEESQNQQEKNEQELLALDKWASLWNWFDITNWLWYIKIFIMIVGGLI  682
23  EINNYTGYIYQLIEESQNQQEKNEQELLALDKWASLWNWFDITNWLWYIKIFIMIVGGLI  712
24  EISNYTGYIYQLIEESQNQQEKNEQELLALDKWASLWNWFDITNWLWYIKIFIMIVGGLI  682
25  EINNYTGYIYQLIEESQNQQEKNEQELLALDKWASLW-WFDITNWLWYIKIFIMIVGGLI  706
26  EINNYTGYIYQLIEESQNQQEKNEQELLAWDKWASLWNWFDITNWLWYIKIFIMIVGGLI  707
27  EINNYTGYIYQLIEESQNQQEKNEQELLALDKWASLWNWFDITNWLWYIKIFIMIVGGLI  708
28  EINNYTGYIYQLIEESQNQQEKNEQELLAWDKWASLWNWFDITNWLWYIKIFIMIVGGLI  703
29  EINNYTGYIYQLIEESQNQQEKNEQELLAWDKWASLWNWFDITNWLWYIKIFIMIVGGLI  708
30  EINNYTGYIYQLIEESQNQQEKNEQELLA-DKWASLWNWFDITNWLWYIKIFIMIVGGLI  704
31  EINNYTGYIYQLIEESQNQQEKNEQELLAWDKWASLWNWFDITNWLWYIKIFIMIVGGLI  705
32  EINNYTGYIYQLIEESQNQQEKNEQELLAWDKWASLWNWFDITNWLWYIKIFIMIVGGLI  705
33  EINNYTGYIYQLIEESQNQQEKNEQELLALDKWASLWNWFDITNWLWYIKIFIMIVGGLI  681
34  EINNYTGYIYQLIEESQNQQEKNEQELLAWDRWASLWNWFDITNWLWYIKIFIMIVGGLI  705
35  EISNYTGYIYQLIEESQNQQEKNEQELLALDKWASLWNWFDITNWLWYIKIFIMIVGGLI  682
36  EINNYTGYIYQLIEESQNQQEKNEQELLAWDKWASLWNWFDITNWLWYIKIFIMIVGGLI  707
37  EINNYTGYIYQLIEESQNQQEKNEQELLALDKWASLWNWFDITNWLWYIKIFIMIVGGLI  707
38  EINNYTGYIYQLIEESQNQQEKNEQELLALDKWASLWNWFDITNWLWYIKIFIMIVGGLI  707
39  EIDNYTGYIYQLIEESQNQQEKNEQELLALDKWASLWNWFDITNWLWYIKIFIMIVGGLI  683
40  EISNYTGYIYQLIEESQNQQEKNEQELLALDKWASLWNWFDITNWLWYIKIFIMIVGGLI  681
41  EINNYTGYIYQLIEESQNQQEKNEQELLALDKWASLWNWFDITNWLWYIKIFIMIVGGLI  707
42  EISNYTGYIYQLIEESQNQQEKNEQELLALDKWASLWNWFDITNWLWYIKIFIMIVGGLI  682
```

#	Sequence	End
	GLRIVFTVLSIVNRVRQGYSPLSFQTHLPAQRGPDRPEGIGEEGGERDRDRSDPLVNGFL	
1	GLRIVFTVLSIVNRVRQGYSPLSFQTHLPAQRGPDRPEGIGEEGGERDRDRSDPLVNGFL	743
2	GLRIVFTVLSIVNRVRQGYSPLSFQTHLPAQRGPDRPEGIGEEGGERDRDRSDPLVNGFL	765
3	GLRIVFTVLSIVNRVRQGYSPLSFQTHLPAQRGPDRPEGIGEEGGERDRDRSDPLVNGFL	742
4	GLRIVFTVLSIVNRVRQGYSPLSFQTHLPAQRGPDRPEGIGEEGGERDRDRSDPLVNGFL	742
5	GLRIVFTVLSIVNRVRQGYSPLSFQTHLPAQRGPDRPEGIGEEGGERDRDRSDPLVNGFL	743
6	GLRIVFTVLSIVNRVRQGYSPLSFQTHFPAQRGPDRPEGIGEEGGERDRDRSAPLVNGFL	767
7	GLRIVFTVLSIVNRVRQGYSPLSFQTHFPAQRGPDRPEGIGEEGGERDRDRSDPLVNGFL	765
8	GLRIVFTVLSIVNRVRQGYSPLSFQTHLPAQRGPDRPEGIGEEGGERDRDRSDPLVNGFL	742
9	GLRIVFTVLSIVNRVRQGYSPLSFQTHLPAQRGPDRPEGIGEEGGERDRDRSDPLVNGFL	765
10	GLRIVFTVLSIVNRVRQGYSPLSFQTHLPAQRGPDRPEGIGEEGGERDRDRSDPLVNGFL	742
11	GLRIVFTVLSIVNRVRQGYSPLSFQTHLPAQRGPDRPEGIGEEGGERDRDRSDPLVNGFL	744
12	GLRIVFTVLSIVNRVRQGYSPLSFQTHLPAQRGPDRPEGIGEEGGERDRDRSDPLVTGFL	767
13	GLRIVFTVLSIVNRVRQGYSPLSFQTHLPAQRGPDRPEGIGEEGGERDRDRSAPLVNGFL	767
14	GLRIVFTVLSIVNRVRQGYSPLSFQTHFPAQRGPDRPEGIGEEGGERDRDRSAPLVNGFL	767
15	GLRIVFTVLSIVNRVRQGYSPLSFQTHFPAQRGPDRPEGIGEEGGERDRDRSAPLVNGFL	767
16	GLRIVFTVLSIVNRVRQGYSPLSFQTHLPAQRGLDRPEGIGEEGGERDRDRSDPLVNGFL	743
17	GLRIVFTVLSIVNRVRQGYSPLSFQTHLPAQRGPDRPEGIGEEGGERDRDRSDPLVNGFL	742
18	GLRIVFAVLSIVNRVRQGYSPLSFQTHLPAQRGPDRPEGIGEEGGERDRDRSDPLVNGFL	760
19	GLRIVFTVLSIVNRVRQGYSPLSFQTHLPAQRGPDRPEGIGEEGGERDRDRSDPLVNGFL	739
20	GLRIVFTVLSIVNRVRQGYSPLSFQTHLPAQRGPDRPEGIGEEGGERDRDRSDPLVNGFL	765
21	GLRIVFTVLSIVNRVRQGYSPLSFQTHLPAQRGLDRPEGIGEEGGERDRDRSDPLVNGFL	739
22	GLRIVFTVLSIVNRVRQGYSPLSFQTHLPAQRGPDRPEGIGEEGGERDRDRSDPLVNGFL	742
23	GLRIVFTVLSIVNRVRQGYSPLSFQTHFPAQRGPDRPEGIGEEGGERDRDRSALLVNGFL	772
24	GLRIVFTVLSIVNRVRQGYSPLSFQTHLPAQRGPDRPEGIGEEGGERDRDRSDPLVNGFL	742
25	GLRIVFTVLSIVNRVRQGYSPLSFQTHFPAQRGPDRPEGIGEEGGERDRDRSDPLVNGFL	766
26	GLRIVFTVLSIVNRVRQGYSPLSFQTHFPAQRGPDRPEGIGEEGGERDRDRSAPLVNGFL	767
27	GLRIVFTVLSIVNRVRQGYSPLSFQTHFPAQRGPDRPEGIGEEGGERDRDRSAPLVNGFL	768
28	GLRIVFTVLSIVNRVRQGYSPLSFQTHFPAQRGPDRPEGIGEEGGERDRDRSAPLVNGFL	763
29	GLRIVFTVLSIVNRVRQGYSPLSFQTHLPAQRGPDRPEGIGEEGGERDRDRSDPLVTGFL	768
30	GLRIVFTVLSIVNRVRQGYSPLSFQTHLPAQRGPDRPEGIGEEGGERDRDRSDPLVTGFL	764
31	GLRIVFTVLSIVNRVRQGYSPLSFQTHFPAQRGPDRPEGIGEEGGERDRDRSDPLVNGFL	765
32	GLRIVFTVLSIVNRVRQGYSPLSFQTHFPAQRGPDRPEGIGEEGGERDRDRSDPLVNGFL	765
33	GLRIVFTVLSIVNRVRQGYSPLSFQTHLPAQRGPDRPEGIGEEGGERDRDRSDPLVNGFL	741
34	GLRIVFTVLSIVNRVRQGYSPLSFQTHLPAQRGPDRPEGIGEEGGERDRDRSDPLVTGFL	765
35	GLRIVFTVLSIVNRVRQGYSPLSFQTHLPAQRGPDRPEGIGEEGGERDRDRSDPLVNGFL	742
36	GLRIVFTVLSIVNRVRQGYSPLSFQTHFPAQRGPDRPEGIGEEGGERDRDRSAPLVNGFL	767
37	GLRIVFTVLSIVNRVRQGYSPLSFQTHLPAQRGPDRPEGIGEEGGERDRDRSAPLVNGFL	767
38	GLRIVFTVLSIVNRVRQGYSPLSFQTHFPAQRGPDRPEGIGEEGGERDRDRSDPLVNGFL	767
39	GLRIVFTVLSIVNRVRQGYSPLSFQTHLPAQRGLDRPEGIGEEGGERDRDRSDPLVNGFL	743
40	GLRIVFTVLSIVNRVRQGYSPLSFQTHLPAQRGPDRPEGIGEEGGERDRDRSDPLVNGFL	741
41	GLRIVFTVLSIVNRVRQGYSPLSFQTHFPAQRGPDRPEGIGEEGGERDRDRSAPLVNGFL	767
42	GLRIVFTVLSIVNRVRQGYSPLSFQTHLPAQRGPDRPEGIGEEGGERDRDRSDPLVNGFL	742

	TLIWSDLRSLCLFSYHRLRDLLLIVTRIVELLGR-------RGWEVLKYWWNLLQYWSQE	
1	ALIWSDLRSLCLFSYHRLRDLLLIVTRIVELLGR-------RGWEVLKYWWNLLQYWSQE	796
2	TFIWSDLRSLCLFSYHHLRDLLLIVTRIVELLGR-------RGWEVLKYWWNLLQYWSQE	818
3	TLIWSDLRSLCLFSYHRLRDLLLIATRIVELLGR-------RGWEVLKYWWNLLQYWSQE	795
4	TLIWSDLRSLCLFSYHRLRDLLLIATRIVELLGR-------RGWEVLKYWWNLLQYWSQE	795
5	TLIWSDLRSLCLFSYHHLRDLLLIVTRIVELLGR-------RGWEVLKYWWNLLQYWSQE	796
6	TLIWSDLRSLCLFSYHRLRDLLLIATRIVELLGR-------RGWEVLKYWWNLLQYWSQE	820
7	TLIWSDLRSLCLFSYHHLRDSLLIVTRIVELLGR-------RGWEVLKYWWNLLQYWSQE	818
8	TLIWSDLRSLCLFSYHRLRDLLLIATRIVELLGR-------RGWEVLKYWWNLLQYWSQE	795
9	TLIWSDLRSLCLFSYHHLRDSLLIVTRIVELLGR-------RGWEVLKYWWNLLQYWSQE	818
10	TIIWSDLRSLCLFSYHRLRDLLLIATRIVELLGR-------RGWEVLKYWWNLLQYWSQE	795
11	TLIWSDLRSLCLFSYHHLRDLLLIVTRIVELLGR-------RGWEVLKYWWNLLQYWSQE	797
12	TLIWSDLRSLCLFSYHRLSDLLLIVTRIVELLGR-------RGWEVLKYWWNLLQYWSQE	820
13	TLIWSDLRSLCLFSYHHLRDSLLIVTRIVELLGR-------RGWEVLKYWWNLLQYWSQE	820
14	TLIWSDLRSLCLFSYHHLRDSLLIVTRIVELLGR-------RGWEVLKYWWNLLQYWSQE	820
15	TFIWSDLRSLCLFSYHHLRDSLLIVTRIVELLGR-------RGWEVLKYWWNLLQYWSQE	820
16	TLIWSDLRSLCLFSYHHLRDLLLIVTRIVELLGR-------RGWEVLKYWWNLLQYWSQE	796
17	TLIWSDLRSLCLFSYHRLRDLLLIATRIVELLGR-------RGWEVLKYWWNLLQYWSQE	795
18	TLIWSDLRSLCLFSYHRLRDLLLIATRIVELLGR-------RGWEVLKYWWNLLQYWSQE	813
19	TLIWSDLRSLGLFSYHHLRDLLLIVTRIVELLGR-------RGWEVLKYWWNLLQYWSQE	792
20	TFIWSDLRSLCLFSYHRLSDLLLIVTRIVELLGR-------RGWEVLKYWWNLLQYWSQE	818
21	TLIWSDLRSLCLFSYHHLRDLLLIVTRIVELLGR-------RGWEVLKYWWNLLQYWSQE	792
22	TLIWSDLRSLCLFSYHRLRDLLLIATRIVELLGR-------RGWEVLKYWWNLLQYWSQE	795
23	TLIWSDLRSLCLFSYHHLRDSLLIVTRIVELLGR-------RGWEVLKYWWNLLQYWSQE	825
24	TLIWSDLRSLCLFSYHRLRDLLLIATRIVELLGR-------RGWEVLKYWWNLLQYWSQE	795
25	TLIWSDLRSLCLFSYHRLRDLLLIATRIVELLGR-------RGWEVLKYWWNLLQYWSQE	819
26	TLIWSDLRSLCLFSYHHLRDLLLIVTRIVELLGR-------RGWEVLKYWWSLLQYWSQE	820
27	TLIWSDLRSLCLFSYHRLRDLLLIATRIVELLGR-------RGWEVLKYWWNLLQYWSQE	821
28	TLIWSDLRSLCLFSYHHLRDSLLIVTRIVELLGR-------RGWEVLKYWWNLLQYWSQE	816
29	TLIWSDLRSLCLFSYHRLSDLLLIVTRIVELLGR-------RGWEVLKYWWNLLQYWSQE	821
30	TLIWSDLRSLCLFSYHRLSDLLLIVTRIVELLGR-------RGWEVLKYWWNLLQYWSQE	817
31	TLIWSDLRSLCLFSYHRLSDLLLIITRIVELLGR-------RGWEVLKYWWNLLQYWSQE	818
32	TFIWSDLRSLCLFSYHRLSDLLLIATRIVELLGR-------RGWEVLKYWWNLLQYWSQE	818
33	TLIWSDLRSLCLFSYHHLRDLLLIVTRIVELLGR-------RGWEVLKYWWNLLQYWSQE	794
34	TLIWSDLRSLCLFSYHRLSDLLLIVTRIVELLGR-------RGWEVLKYWWNLLQYWSQE	818
35	TLIWSDLRSLCLFSYHRLRDLLLIATRIVELLGR-------RGWEVLKYWWNLLQYWSQE	795
36	TLIWSDLRSLCLFSYHRLRDLLLIATRIVELLGR-------RGWEVLKYWWNLLQYWSQE	820
37	TLIWSDLRSLCLFSYHRLRDLLLIATRIVELLGR-------RGWEVLKYWWNLLQYWSQE	820
38	TLIWSDLRSLCLFSYHRLRDLLLIATRIVELLGR-------RGWEVLKYWWNLLQYWSQE	820
39	TLIWSDLRSLCLFSYHHLRDLLLIVTRIVELLGR-------RGWEVLKYWWNLLQYWSQE	796
40	TLIWSDLRSLCLFSYHRLRDLLLIATRIVELLGR-------RGWEVLKYWWNLLQYWSQE	794
41	TLIWSDLRSLCLFSYHHLRDSLLIVTRIVELLGR-------RGWEVLKYWWNLLQYWSQE	820
42	TLIWSDLRSLCLFSYHRLRDLLLIATRIVELLGR-------RGWEVLKYWWNLLQYWSQE	795

	LKNSAVSLLNATAIAVAEGTDRVIEVVQRAGRAIRHIPRRIRQGLERALL	
1	LKNSAVSLLNATAIAVAEGTDRVIEVVQRACRAILHIPRRIRQGLERALL	846
2	LKKSAVSLLNATAIAVAEGTDRVIEVVQRAGRAIRHIPRRIRQGLERALL	868
3	LKNSAVSLFNATAIAVAEGTDRVIEVVQRAGRAIRHIPRRIRQGLERALL	845
4	LRNSAVSLLNATAIAVAEGTDRVIEVVQRAGRAIRHIPRRIRQGLERALL	845
5	LKNSAVSLLNATAIAVAEGTDRVIEVVQRAGRAIRHIPRRIRQGLERALL	846
6	LKKSAVSLLNATAIAVAEGTDRVIEVVQRAGRAIRHIPRRIRQGLERALL	870
7	LKKSAVSLLNATAIAVAEGTDRVIEVVQRTGRAIRHIPRRIRQGLERALL	868
8	LRNSAVSLLNATAIAVAEGTDRVIEVVQRAGRAIRHIPRRIRQGLERALL	845
9	LKKSAVSLLNATAIAVAEGTDRVIEVVQRTGRAIRHIPRRIRQGLERALL	868
10	LRNSAVSLLNATAIAVAEGTDRVIEVVQRAGRAIRHIPRRIRQGLERALL	845
11	LKNSAVSLLNATAIAVAEGTDRVIEVVQRAGRAIRHIPRRIRQGLERALL	847
12	LTNSAVSLLNATAIAVAEGTDRVIEVVQRAGRAVRHIPRRIRQGLERALL	870
13	LKKSAVSLLNATAIAVAEGTDRVIEVAQRTGRAIRHIPRRIRQGLERALL	870
14	LKKSAVSLLNATAIAVAEGTDRVIEVVQRTGRAIRHIPRRIRQGLERALL	870
15	LKKSAVSLLNATAIAVAEGTDRVIEVVQRTGRAIRHIPRRIRQGLERALL	870
16	LKNSTVSLLNATAIAVAEGTDRVIEVVQRAGRAIRHIPRRIRQGLERALL	846
17	LRNSAVSLLNATAIAVAEGTDRVIEVVQRAGRAIRHIPRRIRQGLERALL	845
18	LKKSAVSLLNATAIAVAEGTDRVIEVVQRAGRAIRHIPRRIRQGLERALL	863
19	LKNSAVSLLNATAIAVAEGTDRVIEVVQRAGRAIRHIPRRIRQGLERALL	842
20	LKKSAVSLLNATAIAVAEGTDRVIEVVQRTGRAIRHIPRRIRQGLERALL	868
21	LKNSAVSLLNATAIAVAEGTDRVIEVVQRAGRAIRHIPRRIRQGLERALL	842
22	LRNSAVSLLNATAIAVAEGTDRVIEVVQRAGRAIRHIPRRIRQGLERALL	845
23	LKKSAVSLLNATAIAVAEGTDRVIEVVQRAGRAIRHIPRRIRQGLERALL	875
24	LRNSAVSLLNATAIAVAEGTDRVIEVVQRAGRAIRHIPRRIRQGLERALL	845
25	LKKSAVSLLNATAIAVAEGTDRVIEVVQRAGRAIRHIPRRIRQGLERALL	869
26	LKKSAVSLLNATAIAVAEGTDRVIEVVQRTGRAIRHIPRRIRQGLERALL	870
27	LKKSAVSLLNATAIAVAEGTDRVIEVVQRTGRAIRHIPRRIRQGLERALL	871
28	LKKSAVSLLNATAIAVAEGTDRVIEVVQRTGRAIRHIPRRIRQGLERALL	866
29	LTNSAVSLLNATAIAVAEGTDRVIEVVQRAGRAVRHIPRRIRQGLERALL	871
30	LTNSAVSLLNATAIAVAEGTDRVIEVVQRAGRAVRHIPRRIRQGLERALL	867
31	LTNSAVSLLNATAIAVAEGTDRVIEVVQRAGRAVRHIPRRIRQGLERALL	868
32	LKKSAVSLLNATAIAVAEGTDRVIEVVQRAGRAVRHIPRRIRQGLERALL	868
33	LKNSAVSLLNATAIAVAEGTDRVIEVVQRAGRAIRHIPRRIRQGLERALL	844
34	LTNSAVSLLNATAIAVAEGTDRVIEVVQRAGRAVRHIPRRIRQGLERALL	868
35	LRNSAVSLLNATAIAVAEGTDRVIEVVQRAGRAIRHIPRRIRQGLERALL	845
36	LKKSAVSLLNATAIAVAEGTDRVIEVVQRTGRAIRHIPRRIRQGLERALL	870
37	LKKSAVSLLNATAIAVAEGTDRGIEVVQRTGRAIRHIPRRIRQGLERALL	870
38	LKKSAVSLLNATAIAVAEGTDRVIEVVQRAGRAIRHIPRRIRQGLERALL	870
39	LKNSVVSLLNATAIAVAEGTDRVIEVVQRAGRAIRHIPRRIRQGLERALL	846
40	LRNSAVSLLNATAIAVAEGTDRVIEVVQRAGRAIRHIPRRIRQGLERALL	844
41	LKKSAVSLLNATAIAVAEGTDRVIEVVQRTGRAIRHIPRRIRQGLERALL	870
42	LRNSAVSLLNATAIAVAEGTDRVIEVVQRAGRAIRHIPRRIRQGLERALL	845

Supplementary Table 8. HVTN 704 HIV-1 Envs

#	Env ID	Env clone ID**	Arm	VRC01
1	1	H704_3083_040_EsN	VRC01	Resistant
2	2	H704_2625_090EsN		
3	3	H704_1481_220_RE_p002s		
4	4	H704_2643_210_RE_p001s		
5	6	H704_2536_030_RE_p003s	Placebo	Sensitive
6	7	H704_1747_170_RE_con_s		
7	8	H704_0011_240_RE_pb002_s		
8	9	H704_0011_240_RE_pb003_s		
9	10	H704_0011_240_RE_pb001_s		
10	11	V704_1350_750_RE_pbsga001_s		
11	12	H704_1969_210_RE_pbsga002_s		
12	13	H704_1835_150_RE_p002s_2484A		Resistant
13	14	H704_0445_180_RE_con_s		

** All Env-expressing plasmids (Env clones) of AMP HIV-1 strains were kindly provide by Dr. David Montefiori, Duke University.

a Control

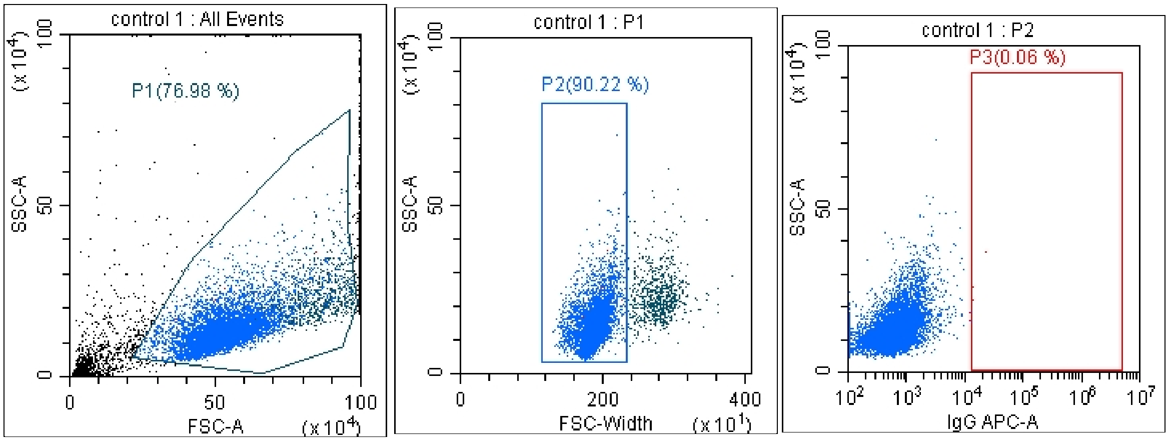

Tube Name: control 1

Sample ID:

Population	Events	% Total	% Parent	Events/µL(V)
All Events	10000	100.00 %	100.00 %	1168.90
P1	7698	76.98 %	76.98 %	899.82
P2	6945	69.45 %	90.22 %	811.80
P3	4	0.04 %	0.06 %	0.47

b N6 bnAb

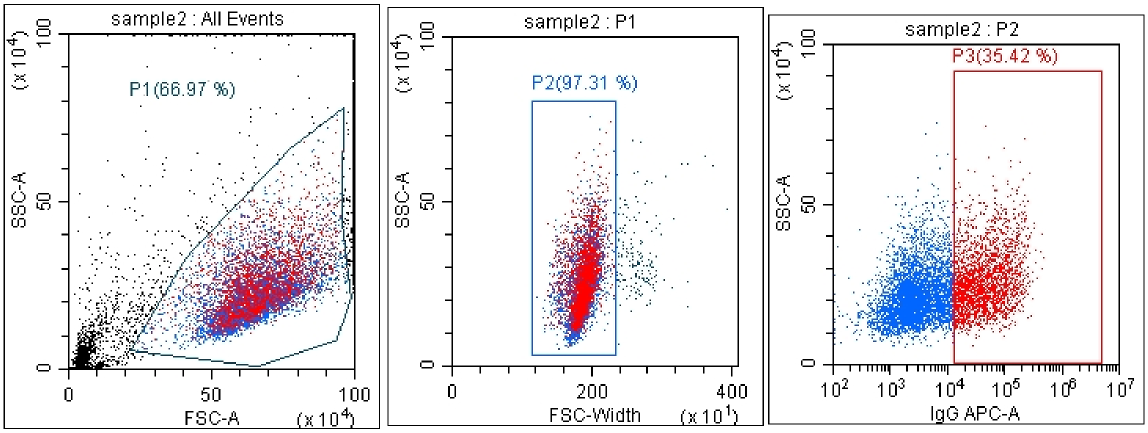

Tube Name: sample2

Sample ID:

Population	Events	% Total	% Parent	Events/µL(V)
All Events	10000	100.00 %	100.00 %	845.01
P1	6697	66.97 %	66.97 %	565.90
P2	6517	65.17 %	97.31 %	550.69
P3	2308	23.08 %	35.42 %	195.03

Supplementary A. flow cytometry gating strategy. 293T cells were transfected with Env-expressing plasmid (HIV-1JRFL△CT or HIV-1JRFL△CT L193R) and binding of different bnAbs to transfected cells was detected with allophycocyanin (APC)-conjugated F(ab′)2 fragment donkey anti-human IgG antibody. Cells were gated first according to side and forward scatter (FSC-A & SSC-A) and then according to SSC-A and FSC-width to exclude doublet cells. Gated 293T cells were then analyzed for the level of APC fluorescence. Control 293T cells (a) and 293T cells transfected with HIV-1JRFL△CT L193R env plasmid and incubated with N6 bnAb (b) are shown.

I. Uncropped scans of gel and blot in Supplementary Fig. 8b.

Lanes are labeled as in the manuscript Supplementary Fig. 8b for 6 gp120 proteins (AMP trial) and AD8 gp120 as control. Lane 1 = protein ladder (Bio-Rad).

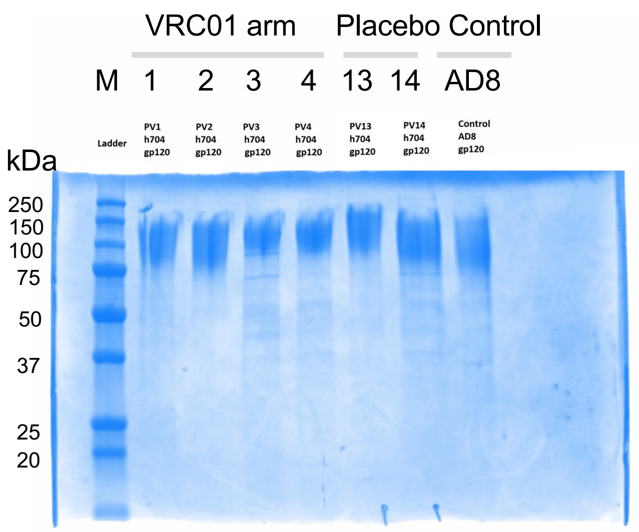

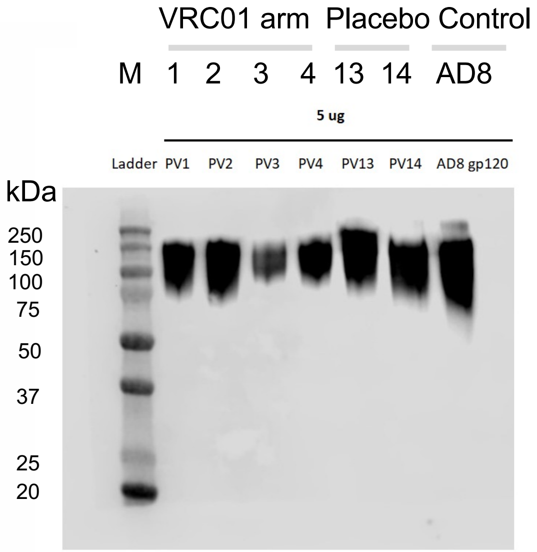

II. An uncropped scan of gel in Supplementary Fig. 11a

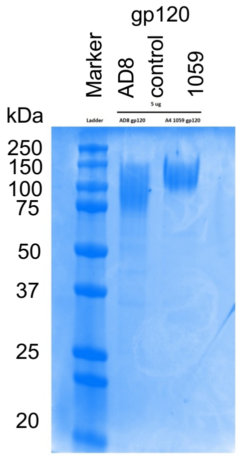

III. An uncropped scan of blot in Supplementary Fig. 12a
(manuscript figure shows only lanes 1-5)

	Signal peptide	Changes	501C-605C	I559P	R6	dMPER	64K	73C-561C	201C-433C	316W	535M	658V
SOSIP.664												
v4.1	Natural	64K, 316W										
DS	Natural	201C-433C										
v5.2.8a	Natural	64K,73C-561C,201C-433C, 316W,658V										
TPA WT	TPA											
TPA P22A	TPA-P22											

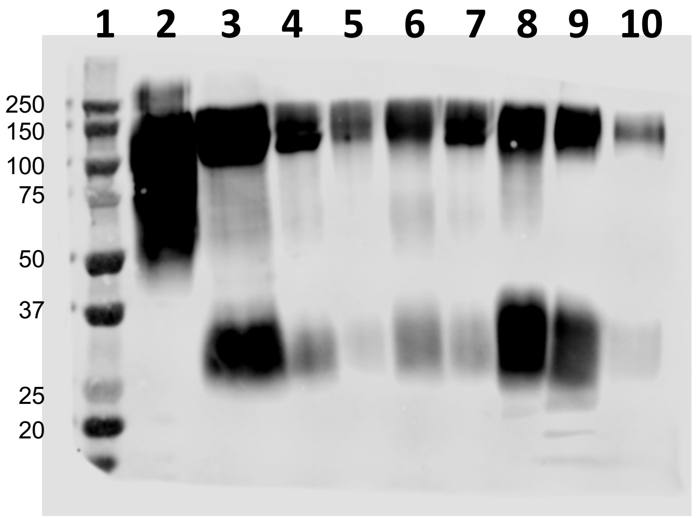

Lanes

1= Protein ladder (Bio-Rad)

2 = gp120 control (HIV-1_{AD8})

3= TPA 1059 SOSIP

4 = TPA P22A 1059 SOSIP

5 = DS 1059 SOSIP

6 = V5.2.8a 1059 SOSIP

7 = v4.1 1059 SOSIP

8 = $HIV_{JRFL\ L193A}$ SOSIP

9 = HIV-1_{BG505} SOSIP

10 = HIV-1_{BG505} SOSIP (in column wash fraction)

TPA, signal peptide of the Tissue Plasminogen Activator.

Supplementary B. Uncropped scans of gels and blots in supplementary figures.
