# Supplementary material for: Conformational flexibility of HIV-1 envelope glycoproteins modulates transmitted/founder sensitivity to broadly neutralizing antibodies
Source: Nat Commun. 2024 Aug 26;15:7334. doi: 10.1038/s41467-024-51656-4 (PMC11347675; doi:10.1038/s41467-024-51656-4)
Supplement: Supplementary file 3 — Description of Additional Supplementary Files [file 41467_2024_51656_MOESM3_ESM.pdf]

Description of Additional Supplementary Files

File name: Supplementary Movie 1a.mov<br>Description: 3D variability analysis of 1059 SOSIP (CryoSPARC). Principal Component (PC)1 topview. Blue, V3 loop; yellow, V1/V2 loop.
File name: Supplementary Movie 1b.mov<br>Description: 3D variability analysis of 1059 SOSIP (CryoSPARC). PC1 sideview. Blue, V3 loop; yellow, V1/V2 loop.
File name: Supplementary Movie 1c.mov<br>Description: 3D variability analysis of 1059 SOSIP (CryoSPARC). PC2 topview. Blue, V3 loop; yellow, V1/V2 loop.
File name: Supplementary Movie 1d.mov<br>Description: 3D variability analysis of 1059 SOSIP (CryoSPARC). PC2 sideview. Blue, V3 loop; yellow, V1/V2 loop.
File name: Supplementary Movie 1e.mov<br>Description: 3D variability analysis of 1059 SOSIP (CryoSPARC). PC3 topview. Blue, V3 loop; yellow, V1/V2 loop.
File name: Supplementary Movie 1f.mov<br>Description: 3D variability analysis of 1059 SOSIP (CryoSPARC). PC3 sideview. Blue, V3 loop; yellow, V1/V2 loop.
File name: Supplementary Movie 2a.mov<br>Description: 3D variability analysis of BG505 SOSIP (CryoSPARC). PC1 topview. Blue, V3 loop; yellow, V1/V2 loop.
File name: Supplementary Movie 2b.mov<br>Description: 3D variability analysis of BG505 SOSIP (CryoSPARC). PC1 sideview. Blue, V3 loop; yellow, V1/V2 loop.
File name: Supplementary Movie 2c.mov<br>Description: 3D variability analysis of BG505 SOSIP (CryoSPARC). PC2 topview. Blue, V3 loop; yellow, V1/V2 loop.
File name: Supplementary Movie 2d.mov<br>Description: 3D variability analysis of BG505 SOSIP (CryoSPARC). PC2 sideview. Blue, V3 loop; yellow, V1/V2 loop.
File name: Supplementary Movie 2e.mov<br>Description: 3D variability analysis of BG505 SOSIP (CryoSPARC). PC3 topview. Blue, V3 loop; yellow, V1/V2 loop.
File name: Supplementary Movie 2f.mov<br>Description: 3D variability analysis of BG505 SOSIP (CryoSPARC). PC3 sideview. Blue, V3 loop; yellow, V1/V2 loop.
