# Supplementary material for: Conformational flexibility of HIV-1 envelope glycoproteins modulates transmitted/founder sensitivity to broadly neutralizing antibodies
Source: Nat Commun. 2024 Aug 26;15:7334. doi: 10.1038/s41467-024-51656-4 (PMC11347675; doi:10.1038/s41467-024-51656-4)
Supplement: Supplementary file 16 — Reporting Summary [file 41467_2024_51656_MOESM16_ESM.pdf]

nature portfolio

Corresponding author(s): Alon Herschhorn

Last updated by author(s): Jul 13, 2024

Reporting Summary

Nature Portfolio wishes to improve the reproducibility of the work that we publish. This form provides structure for consistency and transparency in reporting. For further information on Nature Portfolio policies, see our Editorial Policies and the Editorial Policy Checklist.

Statistics

For all statistical analyses, confirm that the following items are present in the figure legend, table legend, main text, or Methods section.

n/a	Confirmed	
☐	☒	The exact sample size (*n*) for each experimental group/condition, given as a discrete number and unit of measurement
☐	☒	A statement on whether measurements were taken from distinct samples or whether the same sample was measured repeatedly
☐	☒	The statistical test(s) used AND whether they are one- or two-sided<br>*Only common tests should be described solely by name; describe more complex techniques in the Methods section.*
☐	☒	A description of all covariates tested
☒	☐	A description of any assumptions or corrections, such as tests of normality and adjustment for multiple comparisons
☐	☒	A full description of the statistical parameters including central tendency (e.g. means) or other basic estimates (e.g. regression coefficient) AND variation (e.g. standard deviation) or associated estimates of uncertainty (e.g. confidence intervals)
☐	☒	For null hypothesis testing, the test statistic (e.g. *F*, *t*, *r*) with confidence intervals, effect sizes, degrees of freedom and *P* value noted<br>*Give P values as exact values whenever suitable.*
☒	☐	For Bayesian analysis, information on the choice of priors and Markov chain Monte Carlo settings
☒	☐	For hierarchical and complex designs, identification of the appropriate level for tests and full reporting of outcomes
☐	☒	Estimates of effect sizes (e.g. Cohen's *d*, Pearson's *r*), indicating how they were calculated

Our web collection on statistics for biologists contains articles on many of the points above.

Software and code

Policy information about availability of computer code

Data collection	AKTA Go software (Cytiva Unicorn 7);<br>MikroWin 2000 Lite (Berthold Technologies GmbH);<br>Gen5 (BioTek Instruments)<br>CytExpert software 2.2.0.62 (Beckman Coulter)
Data analysis	GraphPad Prism verion 9;<br>3DFSC Processing Server;<br>cryoSPARCv4.01;<br>UCSF ChimeraX 1.6.1;<br>PHENIX 1.20.1-4487;<br>Gatan latitude software GMS 3.5;<br>PyMOL 2.5.5;<br>MODELLER 10.3;<br>Isolde 1.6.0;<br>Coot 0.9.8.8;

For manuscripts utilizing custom algorithms or software that are central to the research but not yet described in published literature, software must be made available to editors and reviewers. We strongly encourage code deposition in a community repository (e.g. GitHub). See the Nature Portfolio guidelines for submitting code & software for further information.

Data

Policy information about availability of data

All manuscripts must include a data availability statement. This statement should provide the following information, where applicable:

- Accession codes, unique identifiers, or web links for publicly available datasets
- A description of any restrictions on data availability
- For clinical datasets or third party data, please ensure that the statement adheres to our policy

Data are available in the manuscript, Extended Data, and supplementary files and movies.
The cryo-EM maps have been deposited in the Electron Microscopy Data Bank (EMDB) under the following accession codes: EMD-41246 for unliganded 1059 SOSIP, EMD-41244 for unliganded BG505 SOSIP. The refined coordinates have been deposited in the RCSB database under the following accession codes: PDB ID 8TGW for unliganded 1059-SOSIP and PDB ID 8TGU for unliganded BG505-SOSIP.

Research involving human participants, their data, or biological material

Policy information about studies with human participants or human data. See also policy information about sex, gender (identity/presentation), and sexual orientation and race, ethnicity and racism.

Reporting on sex and gender	*Use the terms sex (biological attribute) and gender (shaped by social and cultural circumstances) carefully in order to avoid confusing both terms. Indicate if findings apply to only one sex or gender; describe whether sex and gender were considered in study design; whether sex and/or gender was determined based on self-reporting or assigned and methods used. Provide in the source data disaggregated sex and gender data, where this information has been collected, and if consent has been obtained for sharing of individual-level data; provide overall numbers in this Reporting Summary. Please state if this information has not been collected. Report sex- and gender-based analyses where performed, justify reasons for lack of sex- and gender-based analysis.*
Reporting on race, ethnicity, or other socially relevant groupings	*Please specify the socially constructed or socially relevant categorization variable(s) used in your manuscript and explain why they were used. Please note that such variables should not be used as proxies for other socially constructed/relevant variables (for example, race or ethnicity should not be used as a proxy for socioeconomic status). Provide clear definitions of the relevant terms used, how they were provided (by the participants/respondents, the researchers, or third parties), and the method(s) used to classify people into the different categories (e.g. self-report, census or administrative data, social media data, etc.) Please provide details about how you controlled for confounding variables in your analyses.*
Population characteristics	*Describe the covariate-relevant population characteristics of the human research participants (e.g. age, genotypic information, past and current diagnosis and treatment categories). If you filled out the behavioural & social sciences study design questions and have nothing to add here, write "See above."*
Recruitment	*Describe how participants were recruited. Outline any potential self-selection bias or other biases that may be present and how these are likely to impact results.*
Ethics oversight	*Identify the organization(s) that approved the study protocol.*

Note that full information on the approval of the study protocol must also be provided in the manuscript.

Field-specific reporting

Please select the one below that is the best fit for your research. If you are not sure, read the appropriate sections before making your selection.

[x] Life sciences [ ] Behavioural & social sciences [ ] Ecological, evolutionary & environmental sciences

For a reference copy of the document with all sections, see nature.com/documents/nr-reporting-summary-flat.pdf

Life sciences study design

All studies must disclose on these points even when the disclosure is negative.

Sample size	No sample size calculation was performed. We chose 13 out of 20 available T/F HIV-1 strains that showed robust and high infectivity to allow comprehensive and reproducible analysis. We used available 13 Envs from the antibody-mediated prevention trial.
Data exclusions	No data points were excluded but when a technical issue was suspected (e.g. all measurements in the experiment were similar to background) the data of the complete experiment was not included and the experiment was repeated.
Replication	Experiments were independently replicated at least twice
Randomization	Cryo-EM data processing involved splitting the datasets into two random subsets and this procedure is implemented in cryoSPARC v4. Our study is in-vitro biochemical and structural investigation of HIV-1 Env function and conformation; target cells and pseudoviruses are added according to experimental setup without any predefined order.

Blinding	N/A. Our study did not involve human subjects that would require blinding. in-vitro results of our experiments are measured using state-of-the-art equipment that quantified the outcome without bias as they detect and measure luminescence, fluorescence or chemiluminescence.

Reporting for specific materials, systems and methods

We require information from authors about some types of materials, experimental systems and methods used in many studies. Here, indicate whether each material, system or method listed is relevant to your study. If you are not sure if a list item applies to your research, read the appropriate section before selecting a response.

Materials & experimental systems

n/a	Involved in the study
☐	☒ Antibodies
☐	☒ Eukaryotic cell lines
☒	☐ Palaeontology and archaeology
☒	☐ Animals and other organisms
☒	☐ Clinical data
☒	☐ Dual use research of concern
☒	☐ Plants

Methods

n/a	Involved in the study
☒	☐ ChIP-seq
☐	☒ Flow cytometry
☒	☐ MRI-based neuroimaging

Antibodies

Antibodies used

Most antibodies used were from the NIH AIDS Reagent program. In some cases, antibody expressing plasmids were used for expression of antibodies in mammalian cells and the related antibodies were then purified from the cells' supernatant (e.g. JR52 and 19b). Most antibodies were tested in a dose response manner; antibody concentrations used were typically between 100 ug/ml and <0.01 ug/ml and concentrations are specified in the Source Data files.

Antibodies from the NIH AIDS Reagent Program:
N6; catalog number ARP-12968
PG9; catalog number ARP-12149
F240; catalog number ARP-7623
VRC03; catalog number ARP-12032
4E10; catalog number ARP-10091
10E8; catalog number ARP-12294
PG16; catalog number ARP-12150
2F5; catalog number ARP-1475
240-D; catalog number ARP-1242
447-52D; catalog number ARP-4030
F425 B4e8; catalog number ARP-7626
IgG1 b12; catalog number ARP-2640
NIH45-46 G54W; catalog number ARP-12174
7B2-AAA; catalog number ARP-12557
3BNC117; catalog number ARP-12474
246-D; catalog number ARP-1245
48d; catalog number ARP-1756
F105; catalog number ARP-857
50-69; catalog number ARP-531
E51; catalog number ARP-11439
2G12; catalog number ARP-1476
697-30D; catalog number ARP-7371
VRC01; catalog number ARP-12033
39F; catalog number ARP-11437
PGT145; catalog number ARP-12703
PGT126; catalog number ARP-12344
10-1074; catalog number ARP-12477
17b; catalog number ARP-4091
sheep anti-gp120 IgG (1:30,000 dilution; catalog number 288)

Antibodies from research laboratories
JR52 from James Robinson (Tulane University)

Secondary antibodies from Jackson ImmunoResearch Laboratories, West Grove, PA:
horseradish peroxidase (HRP)-conjugated F(ab')2 donkey anti-human IgG, FCgamma; 1:5000 -1:10,000 dilution; catalog number 709-036-098
horseradish peroxidase (HRP)-conjugated rabbit anti-sheep IgG; 1:10,000 dilution; catalog number 313-035-003

	horseradish peroxidase (HRP)-conjugated F(ab')2 Goat anti-mouse IgG FCgamma; 1:10,000 dilution; catalog number 115-036-071<br>Allophycocyanin (APC)-conjugated F(ab')2 fragment donkey anti-human IgG antibody; 1:100 dilution; catalog number 709-136-149
Validation	Antibodies were validated by the NIH AIDS Reagent Program before distribution. There is no information on the program website about how these antibodies are validated but the NIH program is a main and in most cases the only source for these antibodies, and has been distributing them for more than 20 years (https://www.beiresources.org/HIV/ProgramHistory.aspx).<br><br>Secondary antibodies were validated by Jackson ImmunoResearch Laboratories (details on the following websites)<br>https://www.jacksonimmuno.com/catalog/products/115-036-071<br>https://www.jacksonimmuno.com/catalog/products/313-035-003<br>https://www.jacksonimmuno.com/catalog/products/709-036-098<br>https://www.jacksonimmuno.com/catalog/products/709-136-149

Eukaryotic cell lines

Policy information about cell lines and Sex and Gender in Research

Cell line source(s)	Cf2-Th/CD4+CCR5+ and Cf2-Th/CCR5+ cells (Joeseph Sodroski's lab); 293T cells (ATCC); 293F cells (ATCC); TZM-bl cells (NIH AIDS Reagent Program). TZM-bl were derived from HeLa cells, which were originally isolated from a woman. The sex of the donors of the other cell lines is unknown.
Authentication	None of the cell lines was authenticated
Mycoplasma contamination	Cell lines tested negative for mycoplasma
Commonly misidentified lines (See ICLAC register)	No commonly misidentified cell lines were used in the study

Plants

Seed stocks	*Report on the source of all seed stocks or other plant material used. If applicable, state the seed stock centre and catalogue number. If plant specimens were collected from the field, describe the collection location, date and sampling procedures.*
Novel plant genotypes	*Describe the methods by which all novel plant genotypes were produced. This includes those generated by transgenic approaches, gene editing, chemical/radiation-based mutagenesis and hybridization. For transgenic lines, describe the transformation method, the number of independent lines analyzed and the generation upon which experiments were performed. For gene-edited lines, describe the editor used, the endogenous sequence targeted for editing, the targeting guide RNA sequence (if applicable) and how the editor was applied.*
Authentication	*Describe any authentication procedures for each seed stock used or novel genotype generated. Describe any experiments used to assess the effect of a mutation and, where applicable, how potential secondary effects (e.g. second site T-DNA insertions, mosiacism, off-target gene editing) were examined.*

Flow Cytometry

Plots

Confirm that:

- [x] The axis labels state the marker and fluorochrome used (e.g. CD4-FITC).
- [x] The axis scales are clearly visible. Include numbers along axes only for bottom left plot of group (a 'group' is an analysis of identical markers).
- [x] All plots are contour plots with outliers or pseudocolor plots.
- [x] A numerical value for number of cells or percentage (with statistics) is provided.

Methodology

Sample preparation	293T cell line were transfected with Env-expression plasmid and, after 48 hours, detached using 5mM EDTA/PBS and then stained with the specified antibodies. The 293T cells were purchased from the ATCC. Experiment did not involve any tissue processing.
Instrument	CytoFLEX (Beckman Coulter)
Software	CytExpert software 2.2.0.62 (Beckman Coulter)
Cell population abundance	N/A . We used 293T transected cells and abundance was dependent on transfection and growth conditions. We typically detected >60% viable cells according to side and forward scatter.

Gating strategy	Cells were gated according to side (SSC) and forward (FSC) scatters. Gating was based on visual detection of population of cells with clear separation between live and dead cells (please see the example of flow gating strategy in Supplementary Fig. 7 for details).

☒ Tick this box to confirm that a figure exemplifying the gating strategy is provided in the Supplementary Information.
